# Correction: Generation of a transparent killifish line through multiplex CRISPR/Cas9mediated gene inactivation

**DOI:** 10.7554/eLife.112412

**Published:** 2026-06-26

**Authors:** Johannes Krug, Birgit Perner, Carolin Albertz, Hanna Mörl, Vera L Hopfenmüller, Christoph Englert

**Keywords:** Other

Krug J, Perner B, Albertz C, Mörl H, Hopfenmüller VL, Englert C. 2023. Generation of a transparent killifish line through multiplex CRISPR/Cas9mediated gene inactivation. *eLife* **12**:e81549. doi: 10.7554/eLife.81549.

Published 23 February 2023

Prompted by a readers’ request we became aware that due to an oversight we provided two incorrect sequences for oligonucleotides. We sincerely apologize for this oversight and for any confusion this mistake may have caused.

We have corrected the relevant sentence in the Materials and Methods; underline is used to highlight differences.

Corrected text:

To induce a DNA double-strand break in close proximity to the intended site of insertion, the following oligonucleotides for sgRNA synthesis were used: sg_cdkn1a_1: 5’-TAGG-GGGAGTGATATTTCCTTTGA-3’ sg_cdkn1a_2: 5’-AAAC-TCAAAGGAAATATCACTCCC-3’. Synthesis of this sgRNA was done as described above.

Original text:

To induce a DNA double-strand break in close proximity to the intended site of insertion, the following oligonucleotides for sgRNA synthesis were used: sg_cdkn1a_1: 5’-TAGG-AATATCACTCCCCGGATTTC-3’ sg_cdkn1a_2: 5’-AAAC-GAAATCCGGGGAGTGATATT-3’. Synthesis of this sgRNA was done as described above.

The article has been corrected accordingly.

