# Supplementary figures and images for: Generation of a transparent killifish line through multiplex CRISPR/Cas9mediated gene inactivation (part 1 of 2)
Source: eLife. 2023 Feb 23;12:e81549. doi: 10.7554/eLife.81549 (PMC10010688; doi:10.7554/eLife.81549)

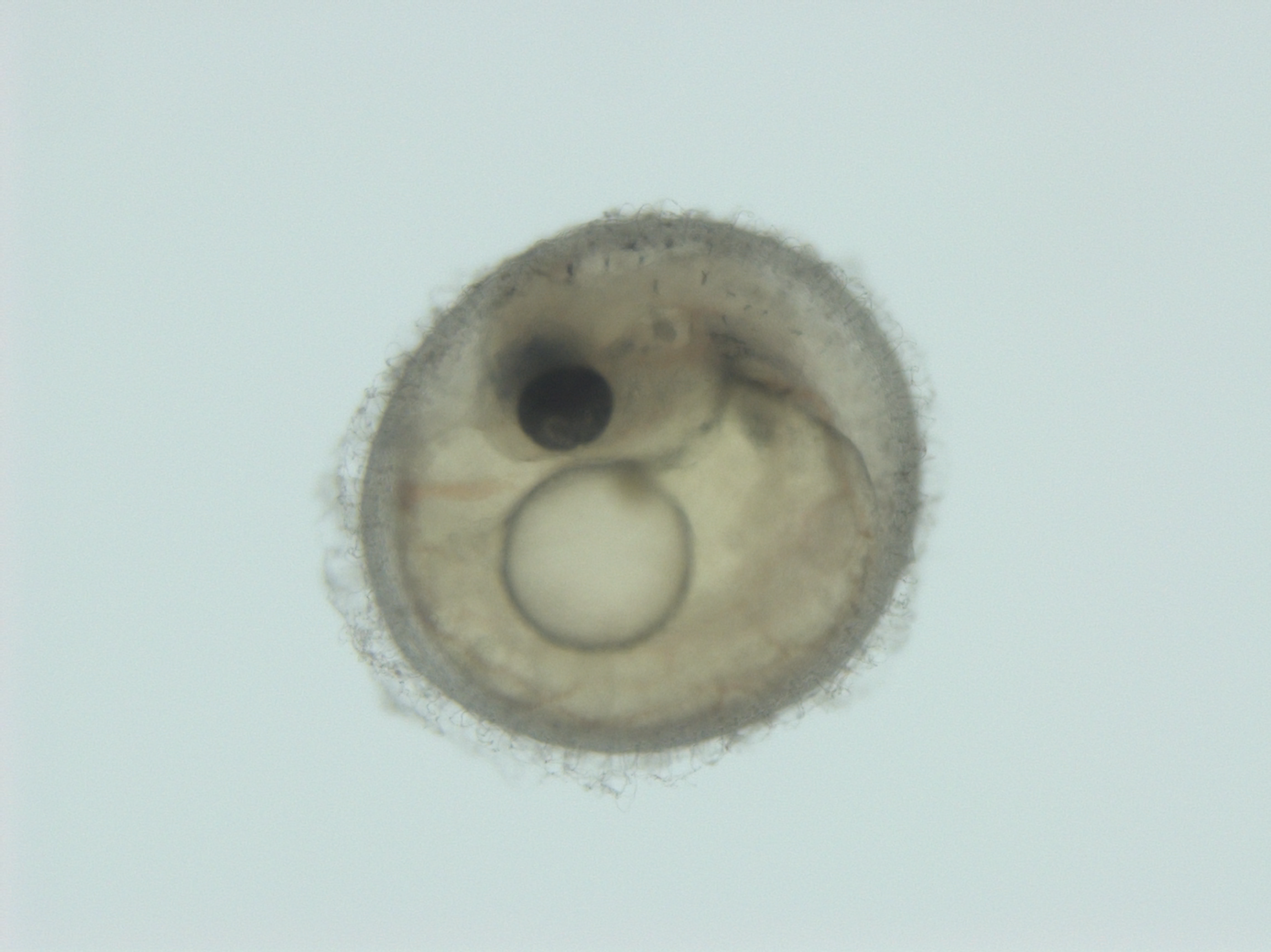

Supplement: Figure 1—source data 1. [file elife-81549-fig1-data1.zip › Figure_1_source_data/Figure_1_panel_D_source_data/GFP-positive_4.tif]

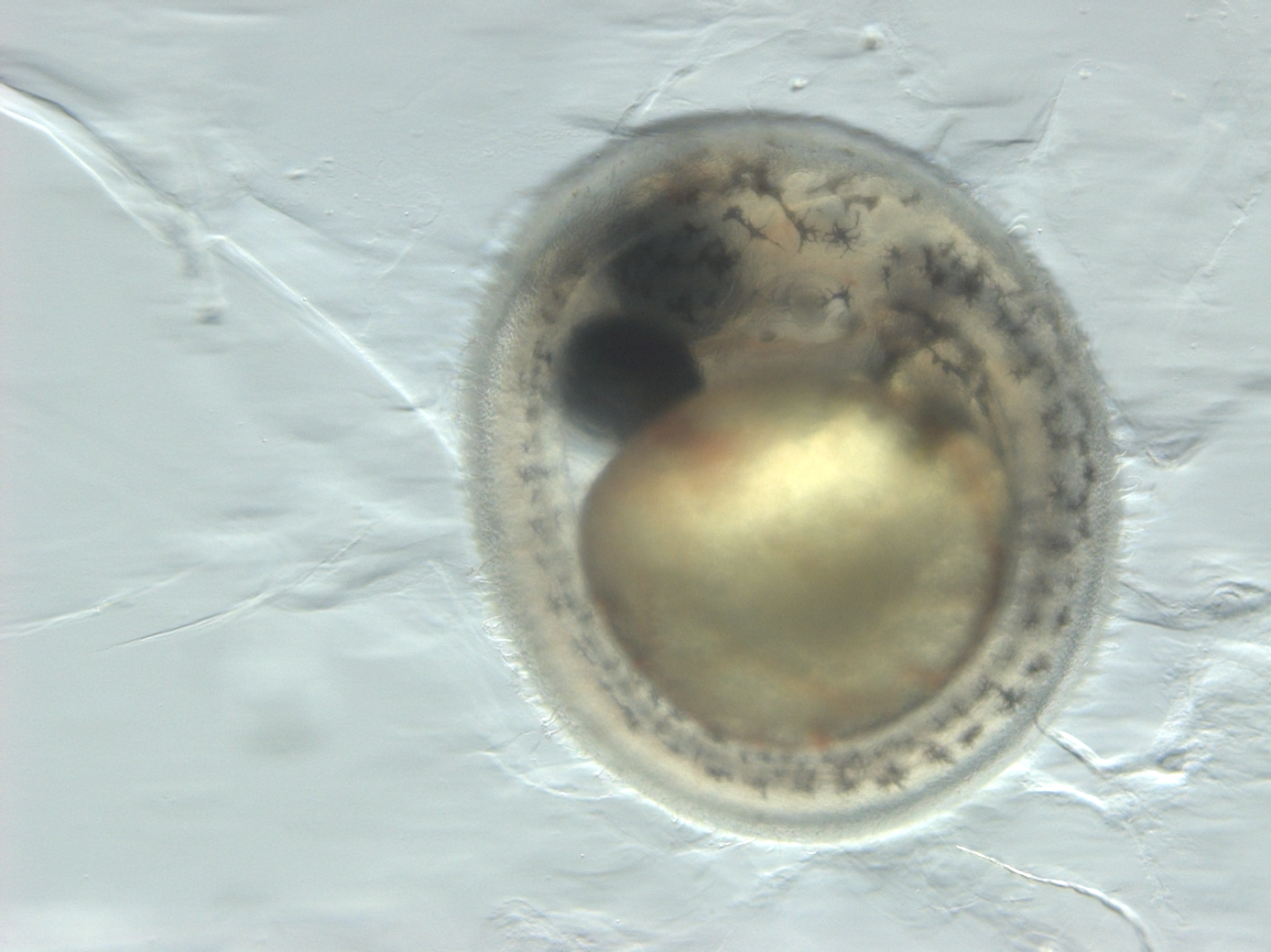

Supplement: Figure 1—source data 1. [file elife-81549-fig1-data1.zip › Figure_1_source_data/Figure_1_panel_D_source_data/GFP_negative_E4.tif]

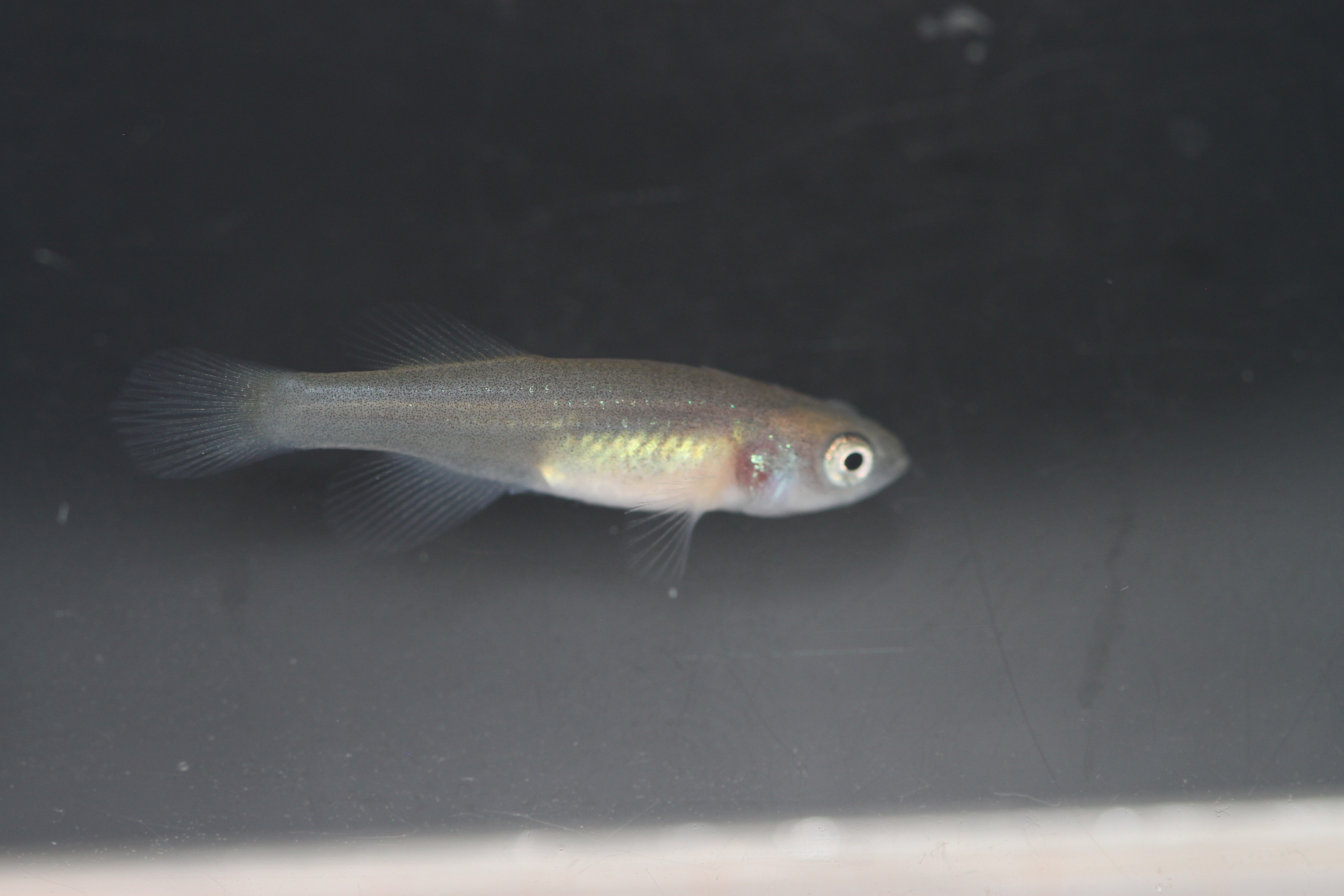

Supplement: Figure 1—source data 1. [file elife-81549-fig1-data1.zip › Figure_1_source_data/Figure_1_panel_EFGHIJK_E ́F ́G ́H ́I ́J ́K ́_source_data/Figure_1_panel_e,f_wild_type/Female_wild_type.JPG]

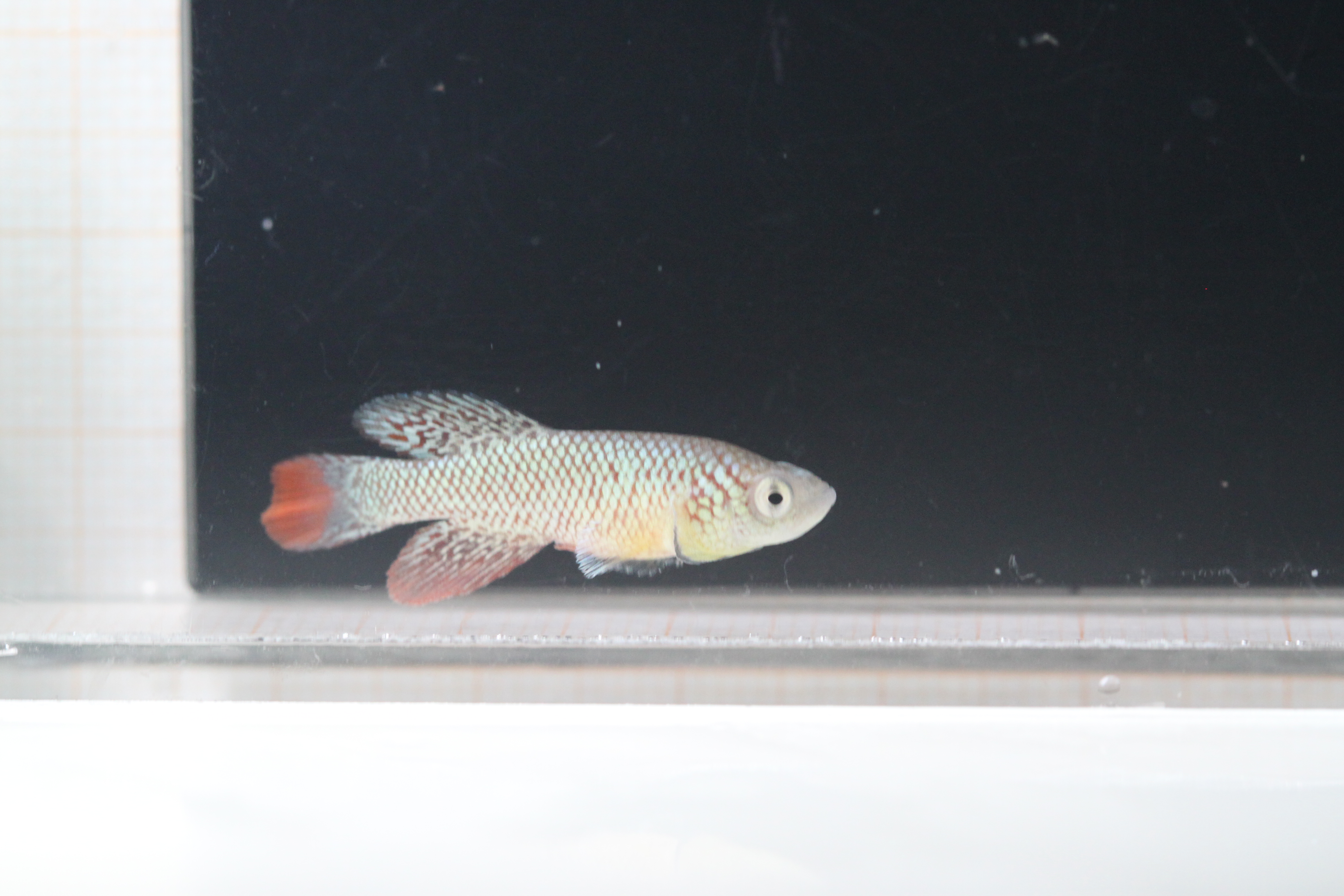

Supplement: Figure 1—source data 1. [file elife-81549-fig1-data1.zip › Figure_1_source_data/Figure_1_panel_EFGHIJK_E ́F ́G ́H ́I ́J ́K ́_source_data/Figure_1_panel_e,f_wild_type/Male_wild_type.JPG]

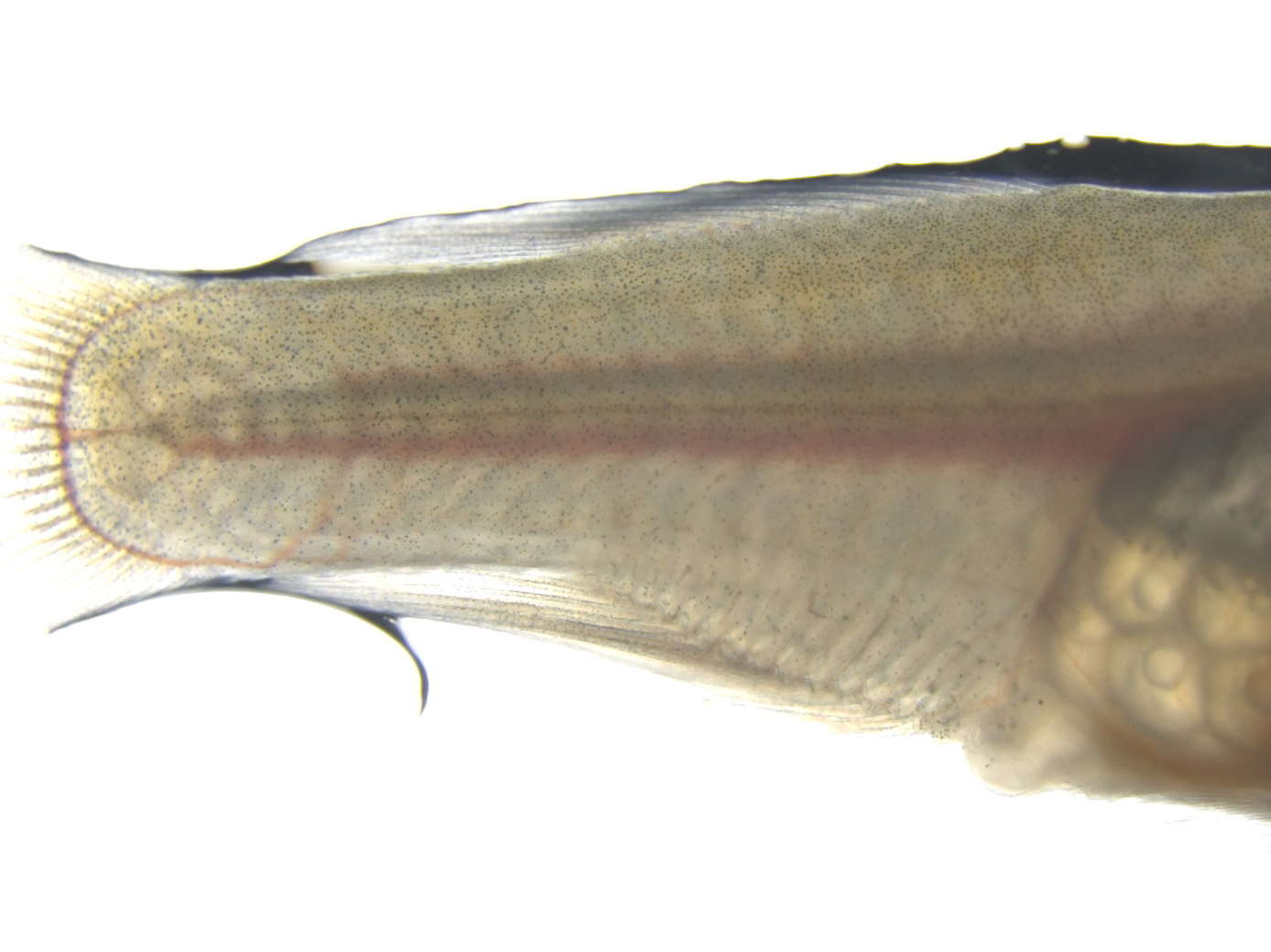

Supplement: Figure 1—source data 1. [file elife-81549-fig1-data1.zip › Figure_1_source_data/Figure_1_panel_EFGHIJK_E ́F ́G ́H ́I ́J ́K ́_source_data/Figure_1_panel_g-j_FO/F0_female_zoom_35dph/Fish 87_Trunk_Lateral-Image Export-01.tif]

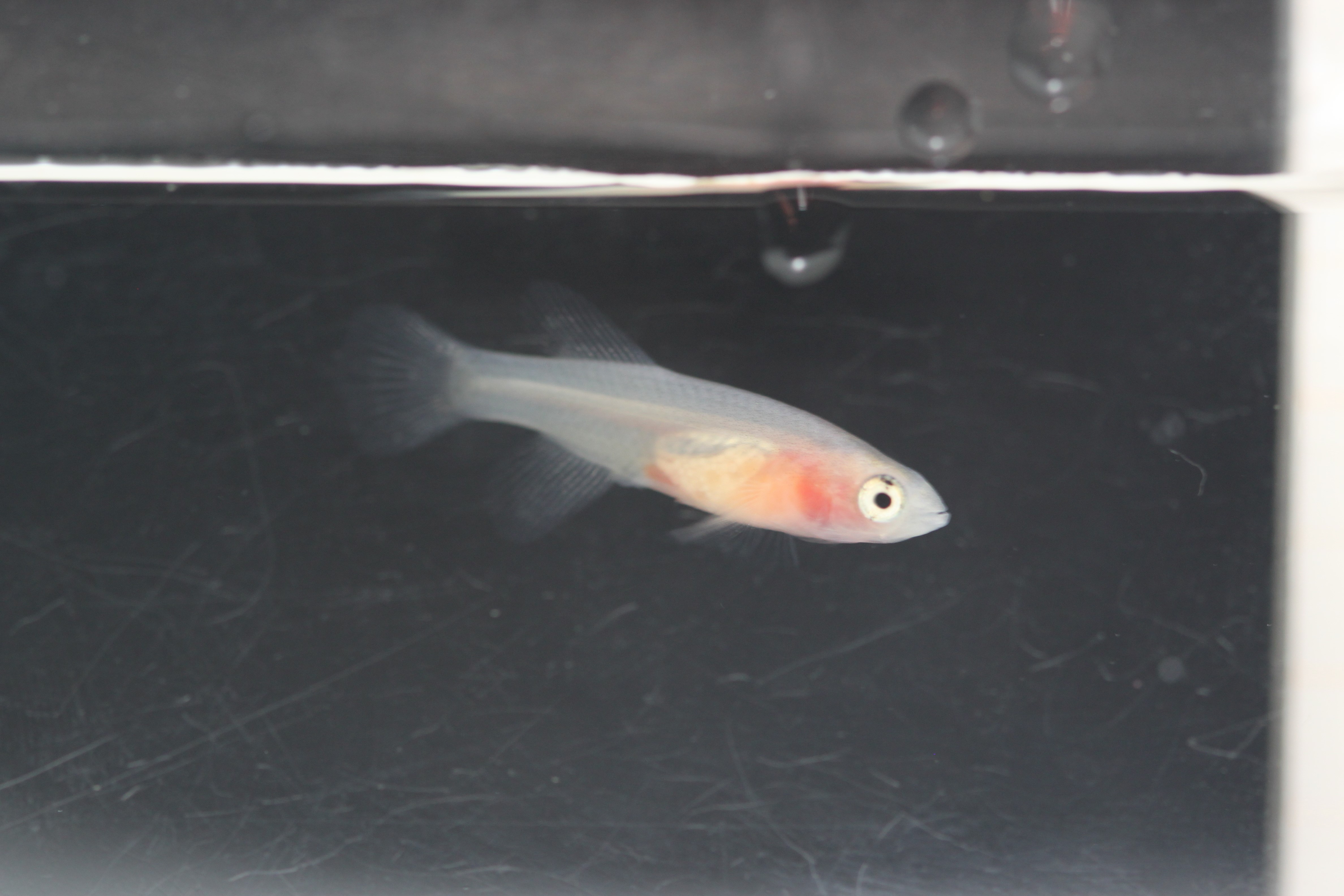

Supplement: Figure 1—source data 1. [file elife-81549-fig1-data1.zip › Figure_1_source_data/Figure_1_panel_EFGHIJK_E ́F ́G ́H ́I ́J ́K ́_source_data/Figure_1_panel_g-j_FO/F0_fish_female_transparent_25dph.jpg]

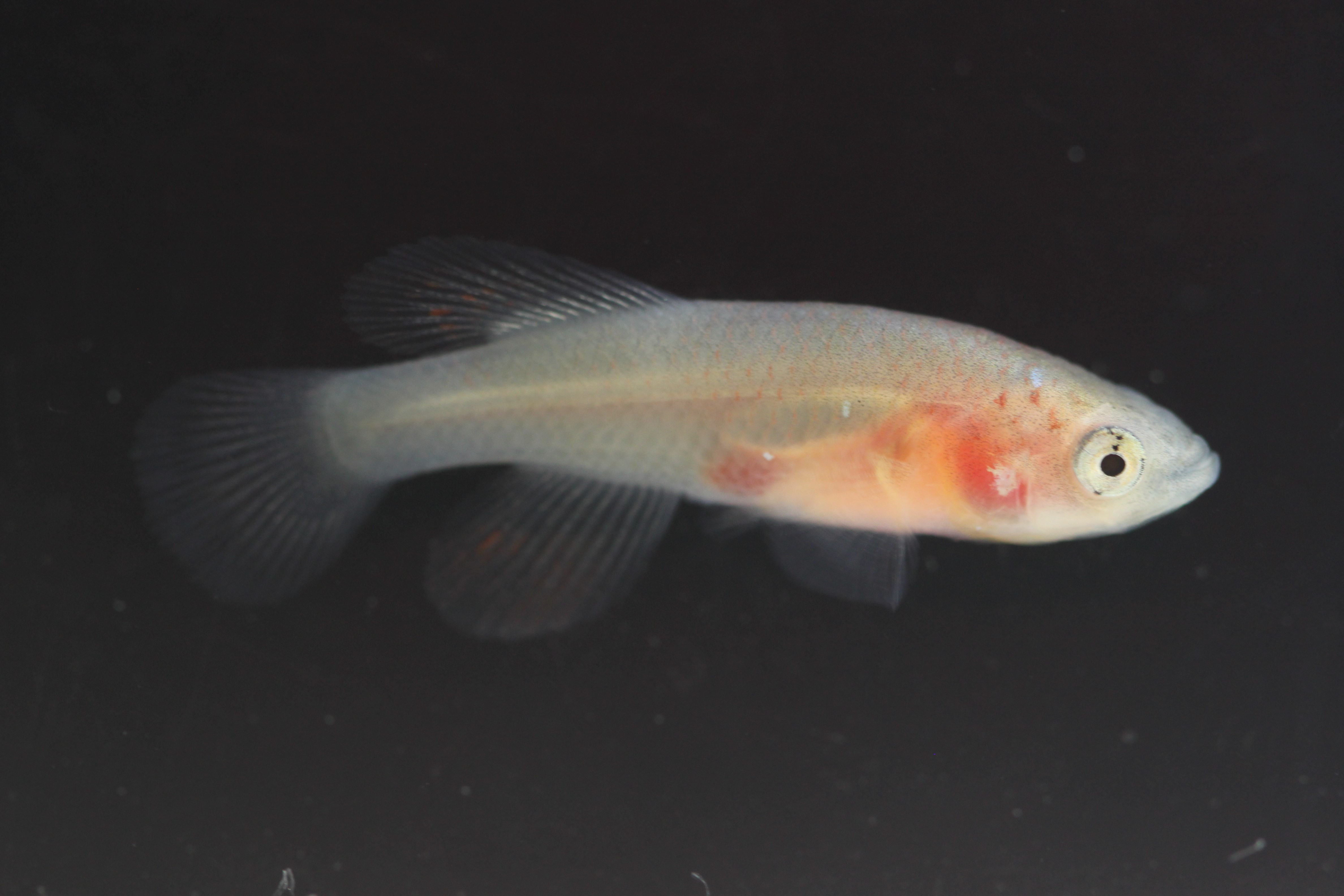

Supplement: Figure 1—source data 1. [file elife-81549-fig1-data1.zip › Figure_1_source_data/Figure_1_panel_EFGHIJK_E ́F ́G ́H ́I ́J ́K ́_source_data/Figure_1_panel_g-j_FO/F0_fish_male_transparent_25dph.jpg]

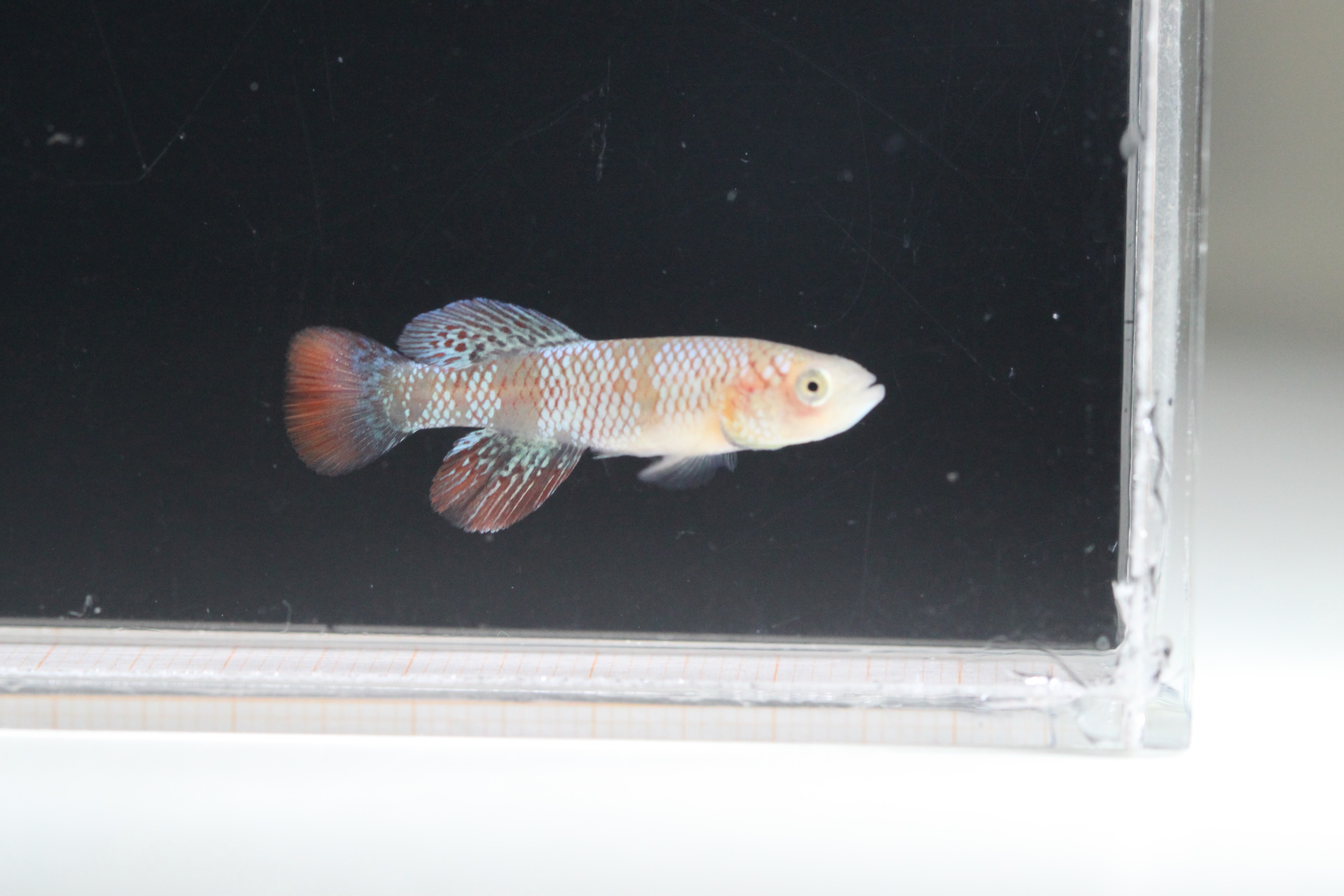

Supplement: Figure 1—source data 1. [file elife-81549-fig1-data1.zip › Figure_1_source_data/Figure_1_panel_EFGHIJK_E ́F ́G ́H ́I ́J ́K ́_source_data/Figure_1_panel_g-j_FO/F0_fish_mosaic_25dph.jpg]

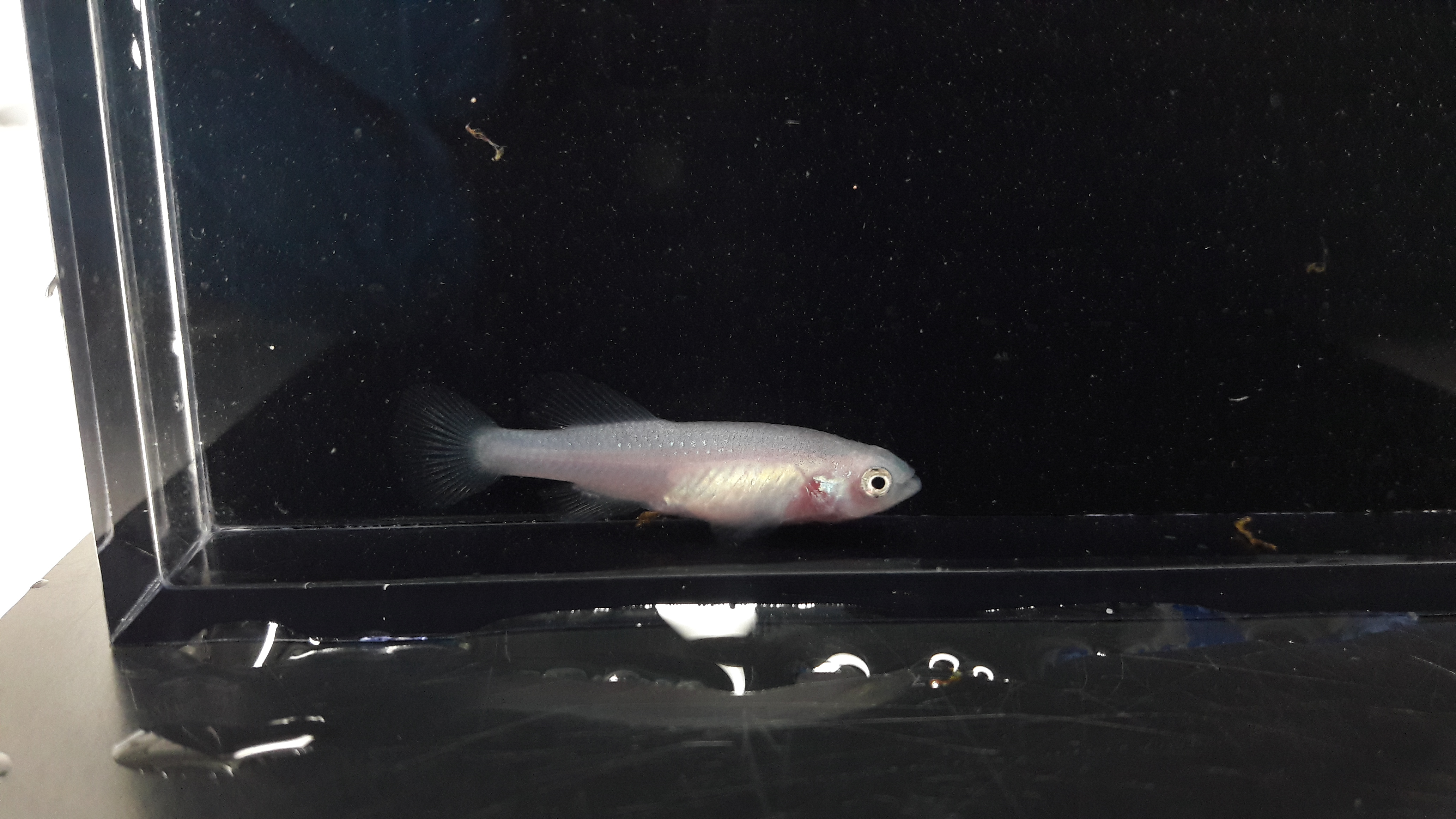

Supplement: Figure 1—source data 1. [file elife-81549-fig1-data1.zip › Figure_1_source_data/Figure_1_panel_EFGHIJK_E ́F ́G ́H ́I ́J ́K ́_source_data/Figure_1_panel_k-l'_F2/Female_panel_k'.jpg]

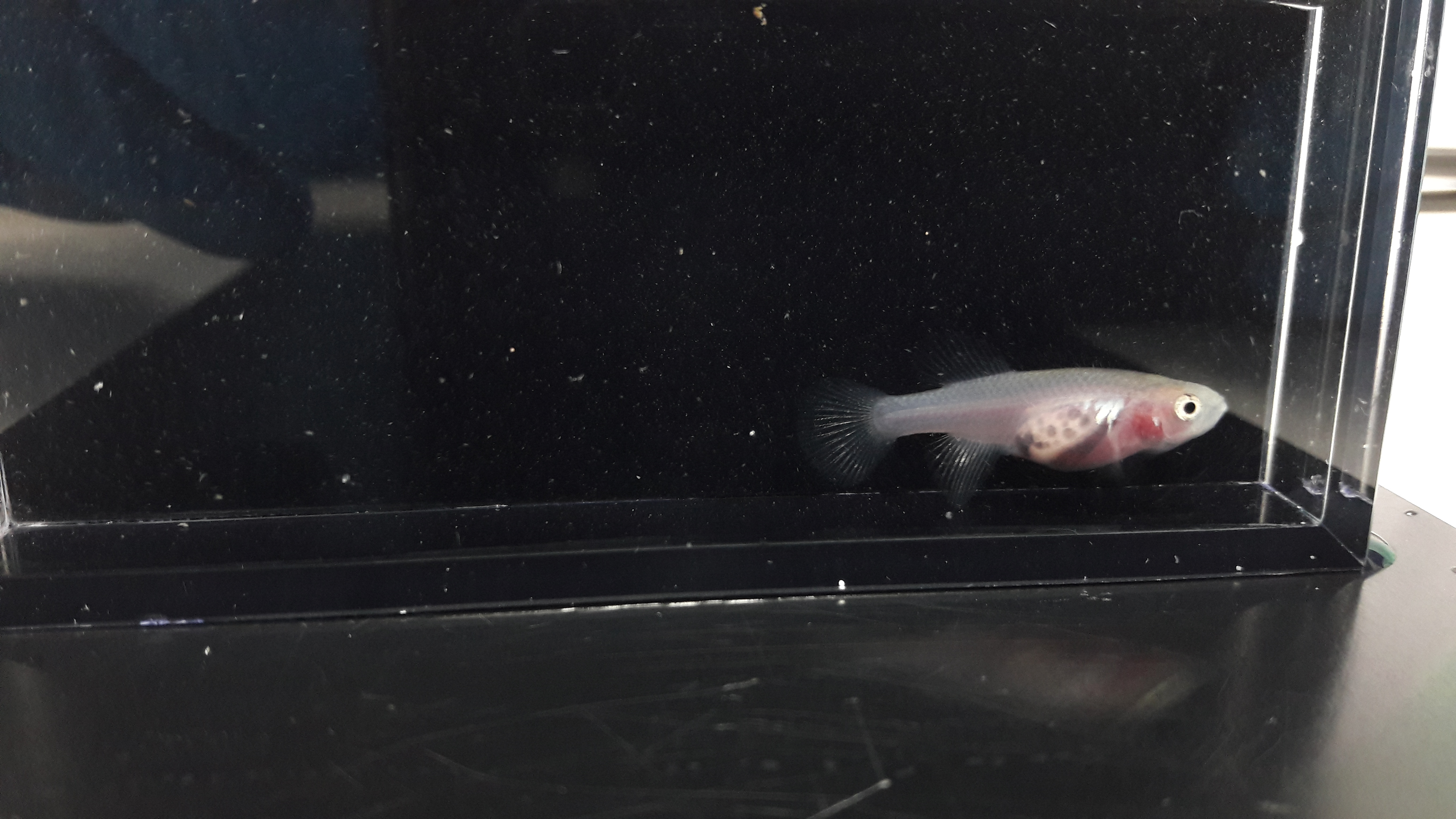

Supplement: Figure 1—source data 1. [file elife-81549-fig1-data1.zip › Figure_1_source_data/Figure_1_panel_EFGHIJK_E ́F ́G ́H ́I ́J ́K ́_source_data/Figure_1_panel_k-l'_F2/Female_panel_l'.jpg]

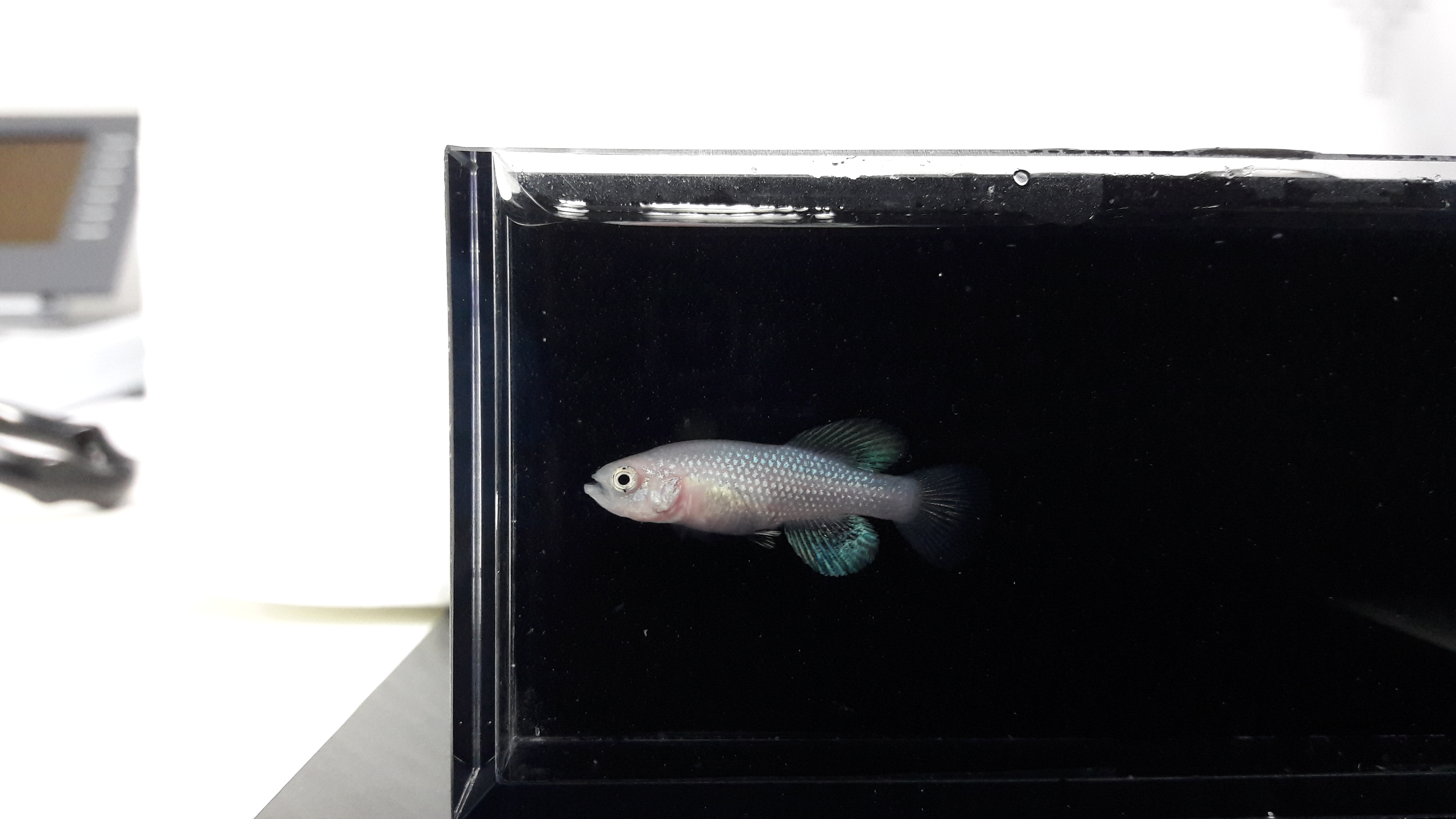

Supplement: Figure 1—source data 1. [file elife-81549-fig1-data1.zip › Figure_1_source_data/Figure_1_panel_EFGHIJK_E ́F ́G ́H ́I ́J ́K ́_source_data/Figure_1_panel_k-l'_F2/Male_panel_k.jpg]

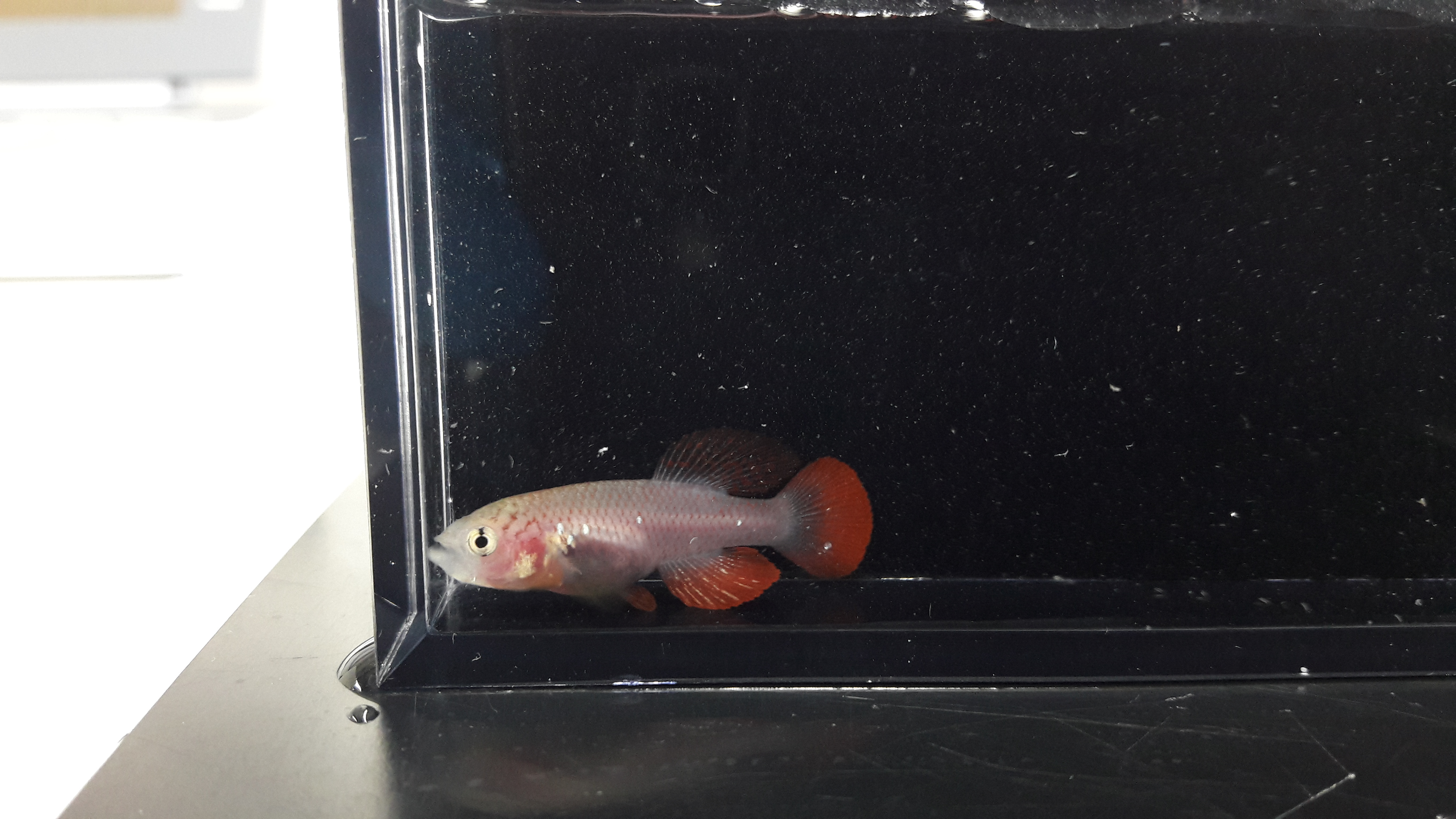

Supplement: Figure 1—source data 1. [file elife-81549-fig1-data1.zip › Figure_1_source_data/Figure_1_panel_EFGHIJK_E ́F ́G ́H ́I ́J ́K ́_source_data/Figure_1_panel_k-l'_F2/Male_panel_l.jpg]

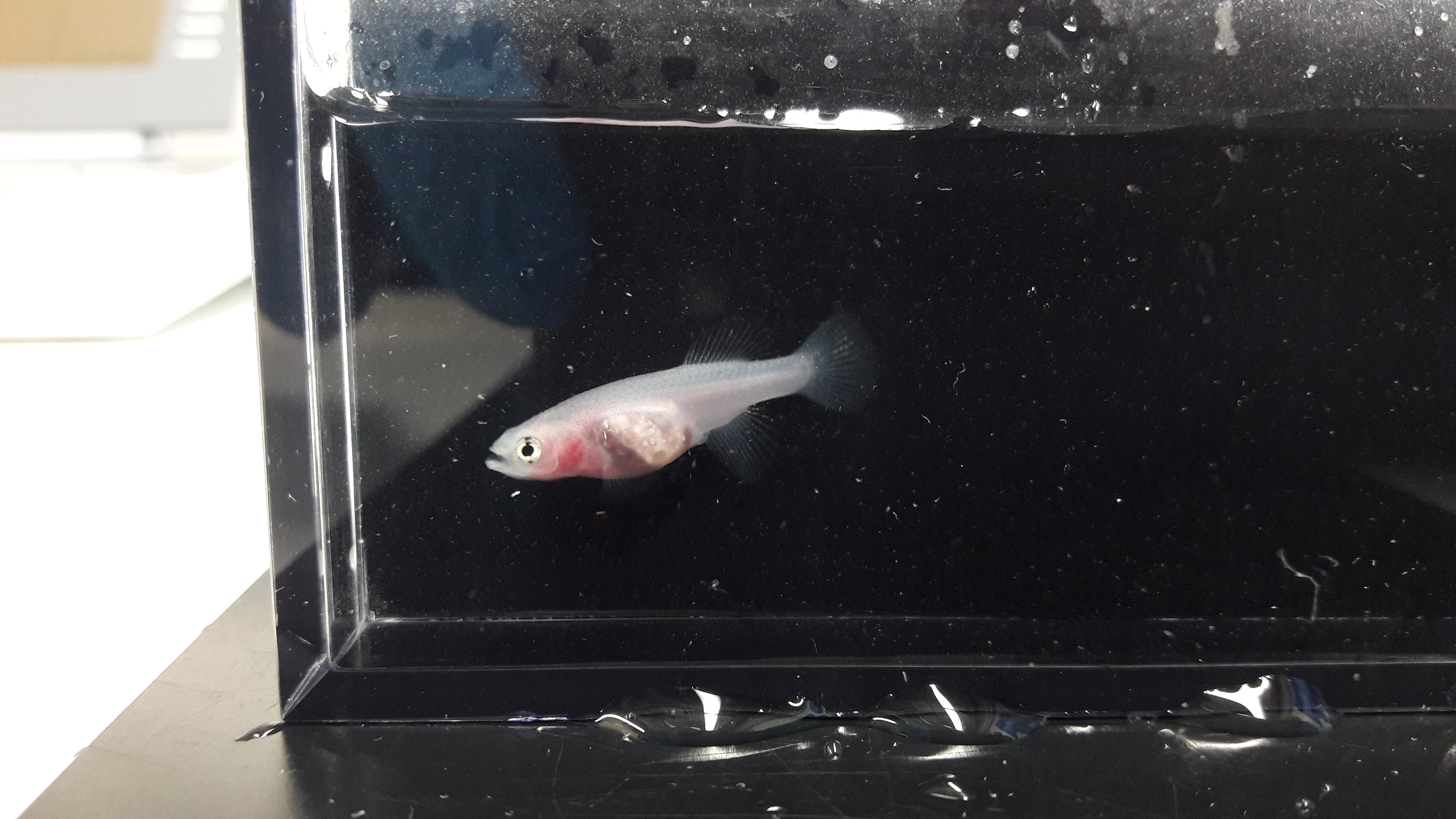

Supplement: Figure 1—source data 1. [file elife-81549-fig1-data1.zip › Figure_1_source_data/Figure_1_panel_EFGHIJK_E ́F ́G ́H ́I ́J ́K ́_source_data/Figure_1_panel_m-m'_F3/Female_panel_m'.jpg]

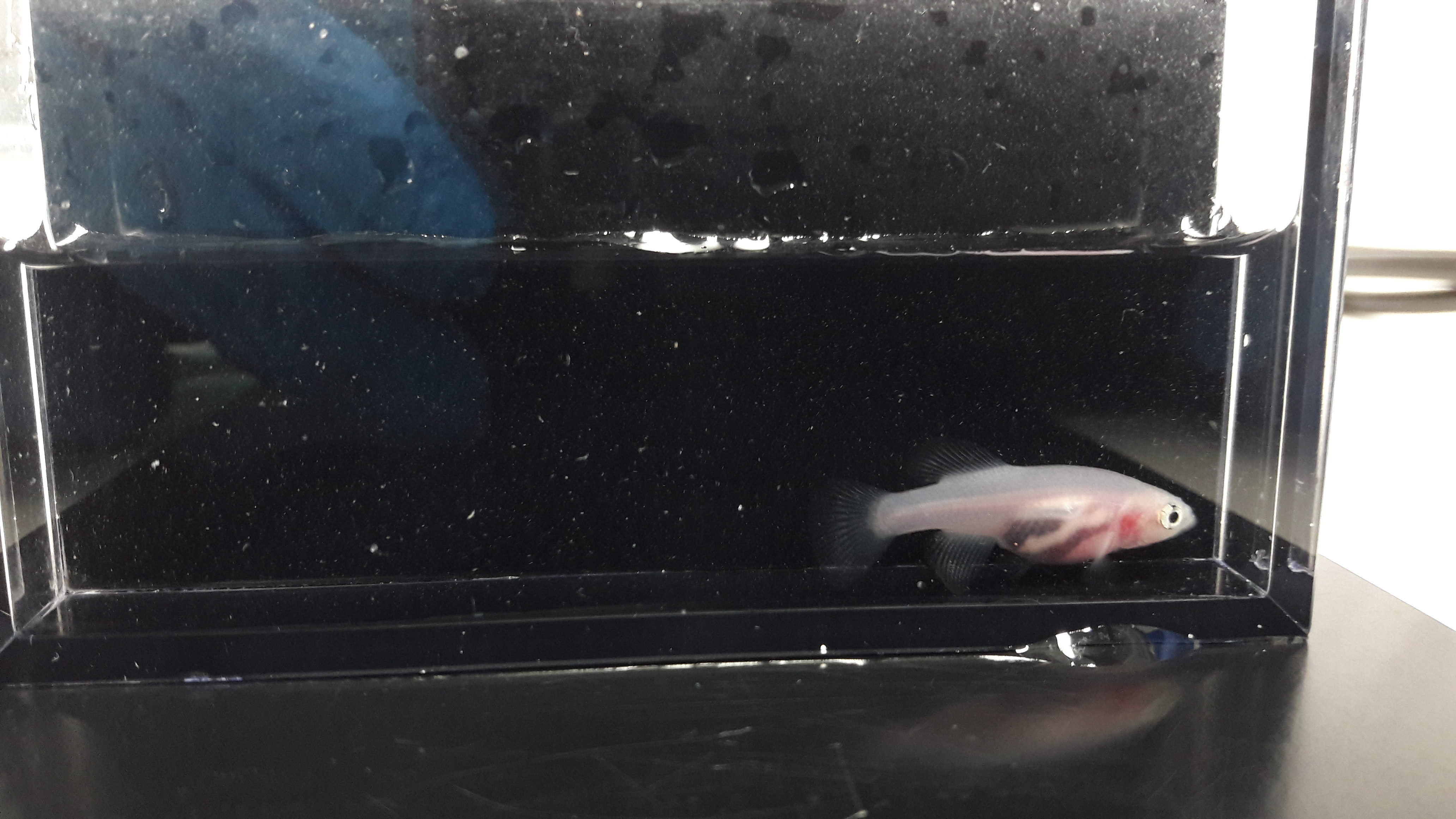

Supplement: Figure 1—source data 1. [file elife-81549-fig1-data1.zip › Figure_1_source_data/Figure_1_panel_EFGHIJK_E ́F ́G ́H ́I ́J ́K ́_source_data/Figure_1_panel_m-m'_F3/Male_panel_m.jpg]

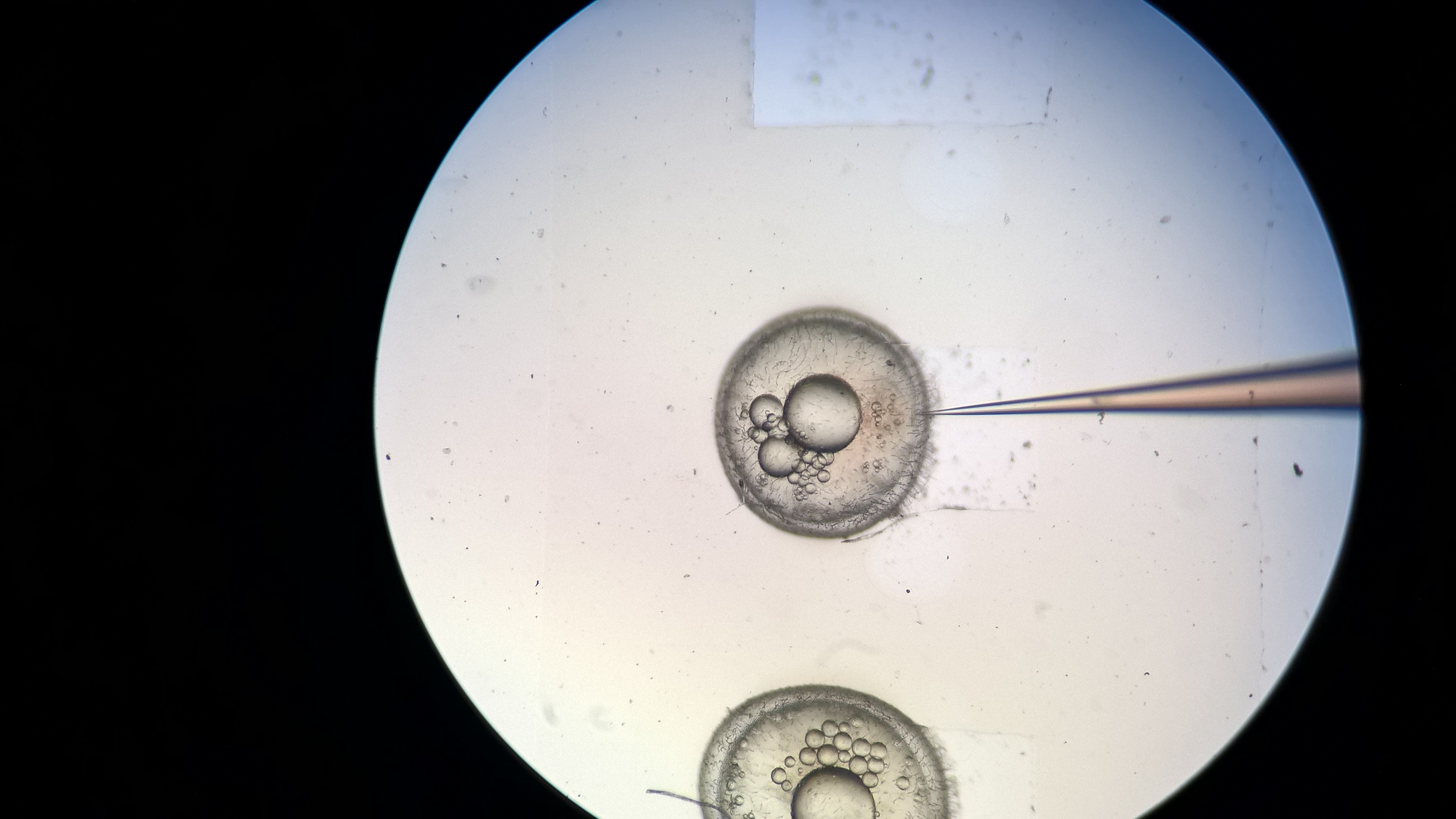

Supplement: Figure 1—figure supplement 1—source data 1. [file elife-81549-fig1-figsupp1-data1.zip › Figure_1_figure_supplement_1_source_data/Figure_1_figure_supplement_1_panel_G_source_data/Injection_egg.jpg]

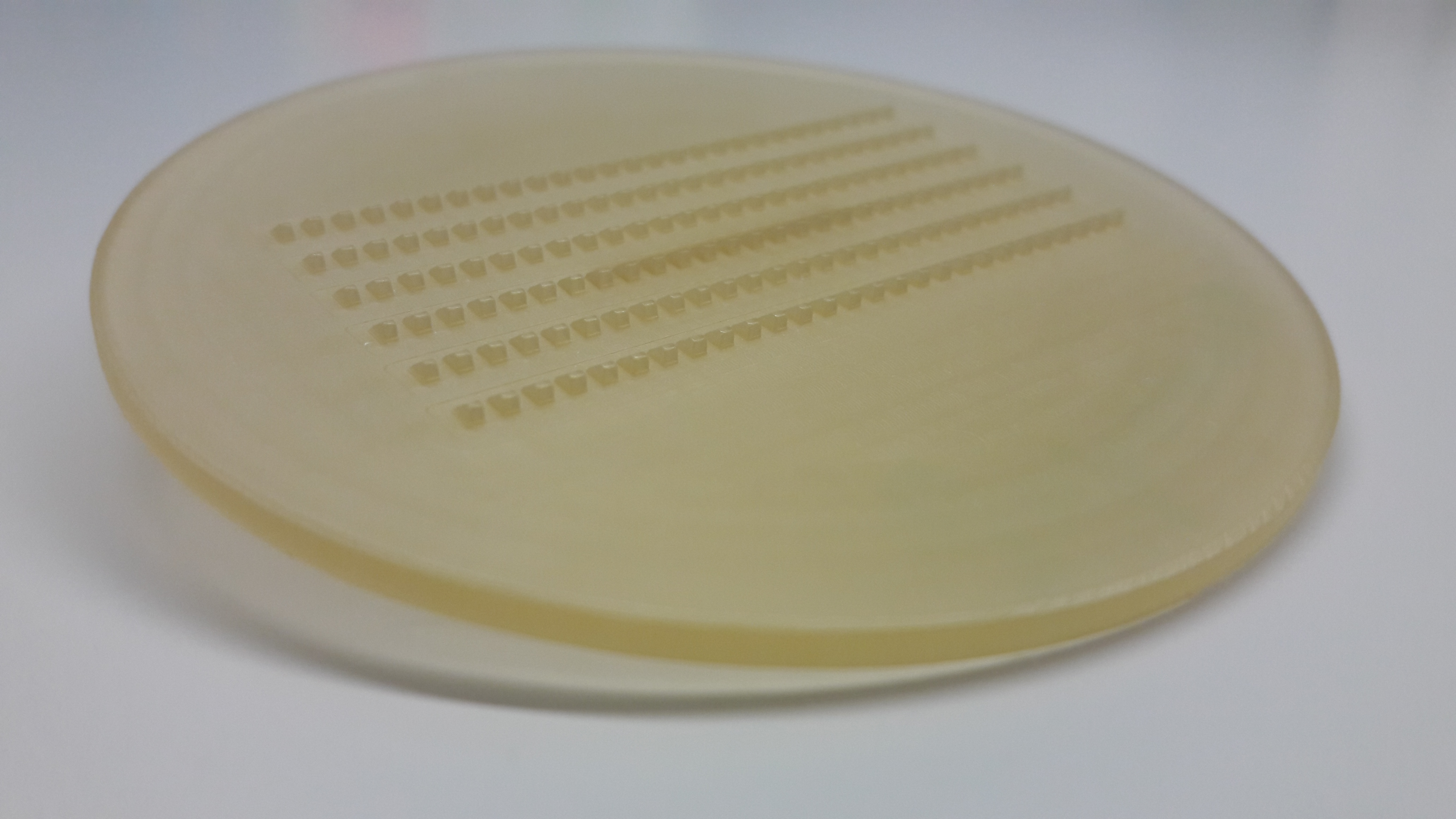

Supplement: Figure 1—figure supplement 1—source data 1. [file elife-81549-fig1-figsupp1-data1.zip › Figure_1_figure_supplement_1_source_data/Figure_1_figure_supplement_1_panel_G_source_data/Injection_mold_stamp.jpg]

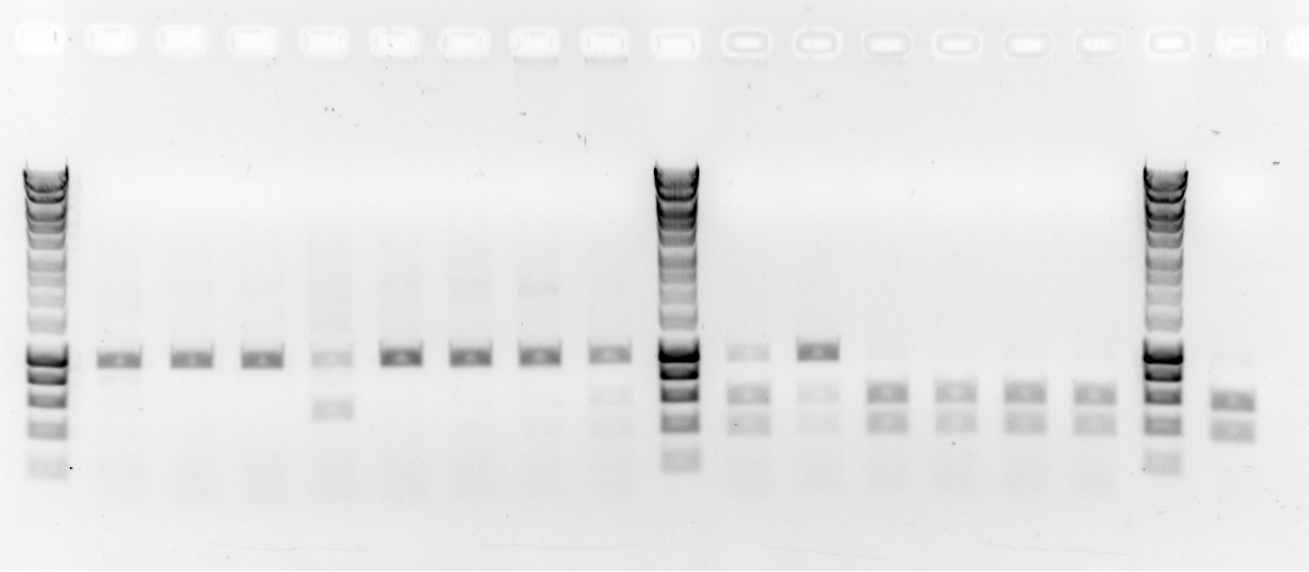

Supplement: Figure 1—figure supplement 2—source data 1. [file elife-81549-fig1-figsupp2-data1.zip › Figure_1_figure_supplement_2_source_data/Figure_1_figure_supplement_2_panel_ABC_source_data/Figure_1_figure_supplement_1_panel_a_source_data.tif]

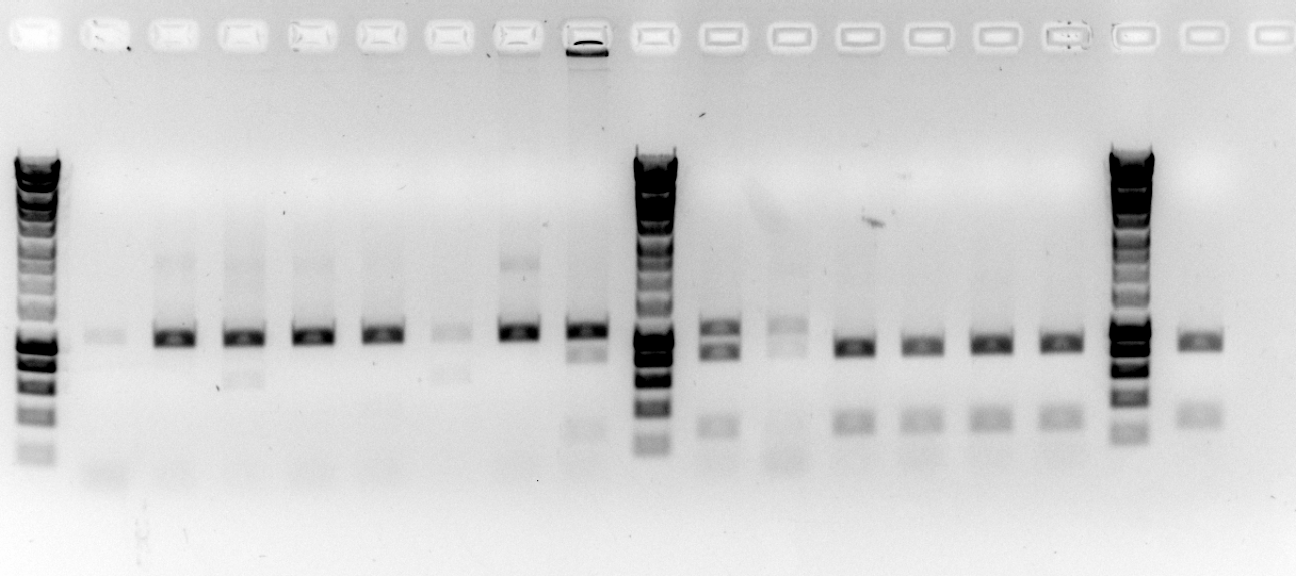

Supplement: Figure 1—figure supplement 2—source data 1. [file elife-81549-fig1-figsupp2-data1.zip › Figure_1_figure_supplement_2_source_data/Figure_1_figure_supplement_2_panel_ABC_source_data/Figure_1_figure_supplement_1_panel_b_source_data.tif]

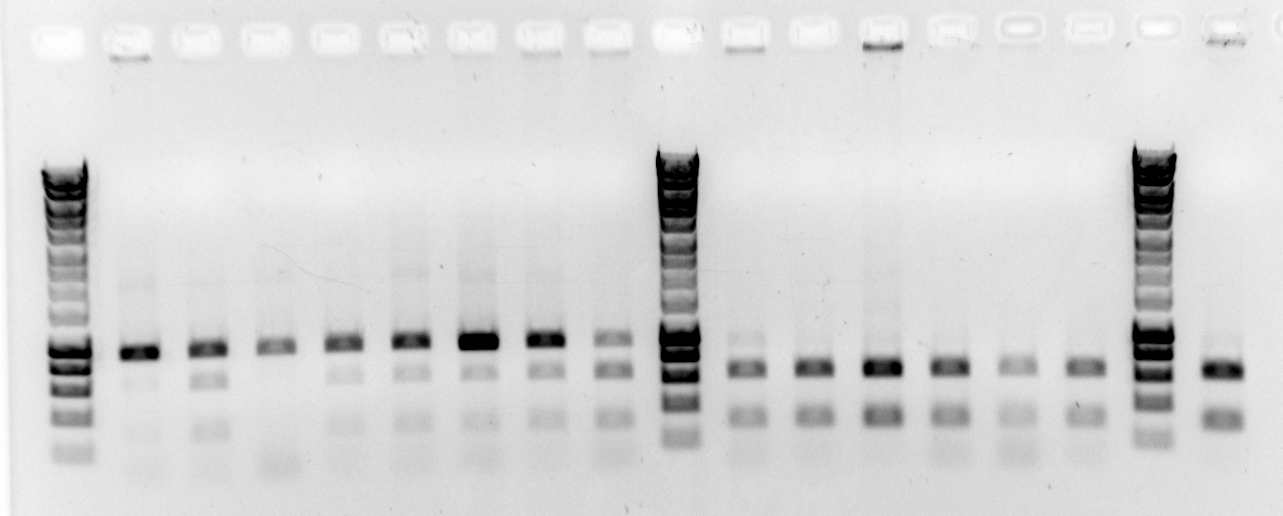

Supplement: Figure 1—figure supplement 2—source data 1. [file elife-81549-fig1-figsupp2-data1.zip › Figure_1_figure_supplement_2_source_data/Figure_1_figure_supplement_2_panel_ABC_source_data/Figure_1_figure_supplement_1_panel_c_source_data.tif]

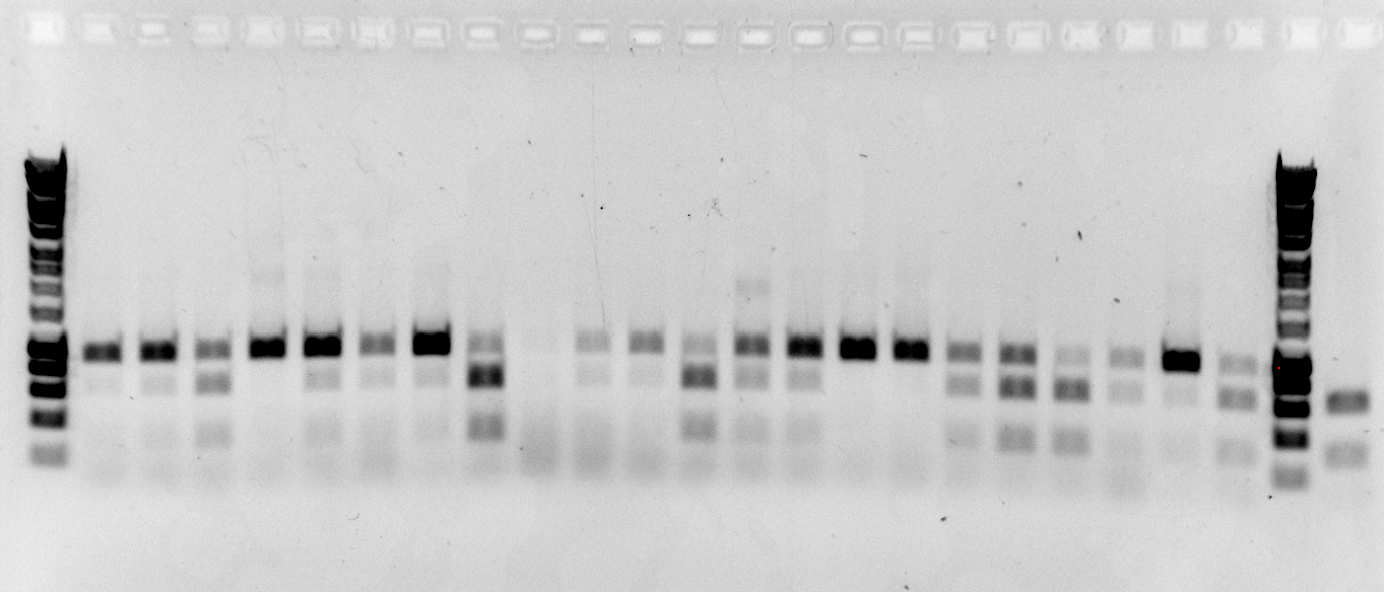

Supplement: Figure 1—figure supplement 2—source data 1. [file elife-81549-fig1-figsupp2-data1.zip › Figure_1_figure_supplement_2_source_data/Figure_1_figure_supplement_2_panel_D_source_data/Gel_pictures/Control digest_transp._fish_#1-41_csf1ra_Gel-oben_15.09.2017.tif]

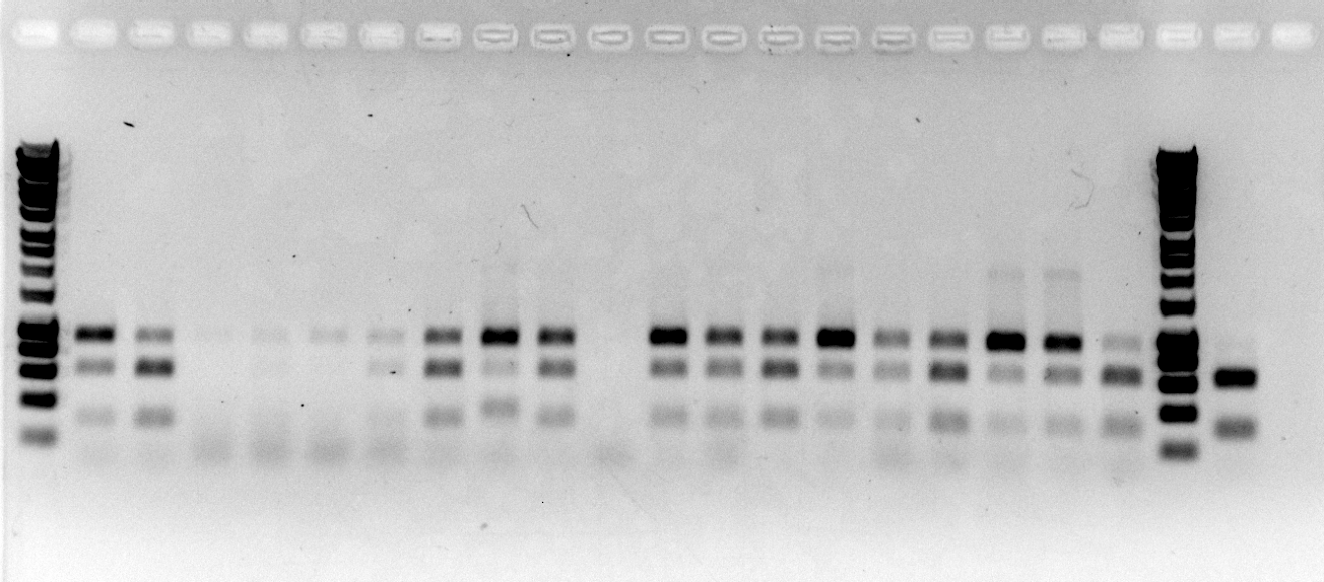

Supplement: Figure 1—figure supplement 2—source data 1. [file elife-81549-fig1-figsupp2-data1.zip › Figure_1_figure_supplement_2_source_data/Figure_1_figure_supplement_2_panel_D_source_data/Gel_pictures/Control digest_transp._fish_#1-41_csf1ra_Gel-unten_15.09.2017.tif]

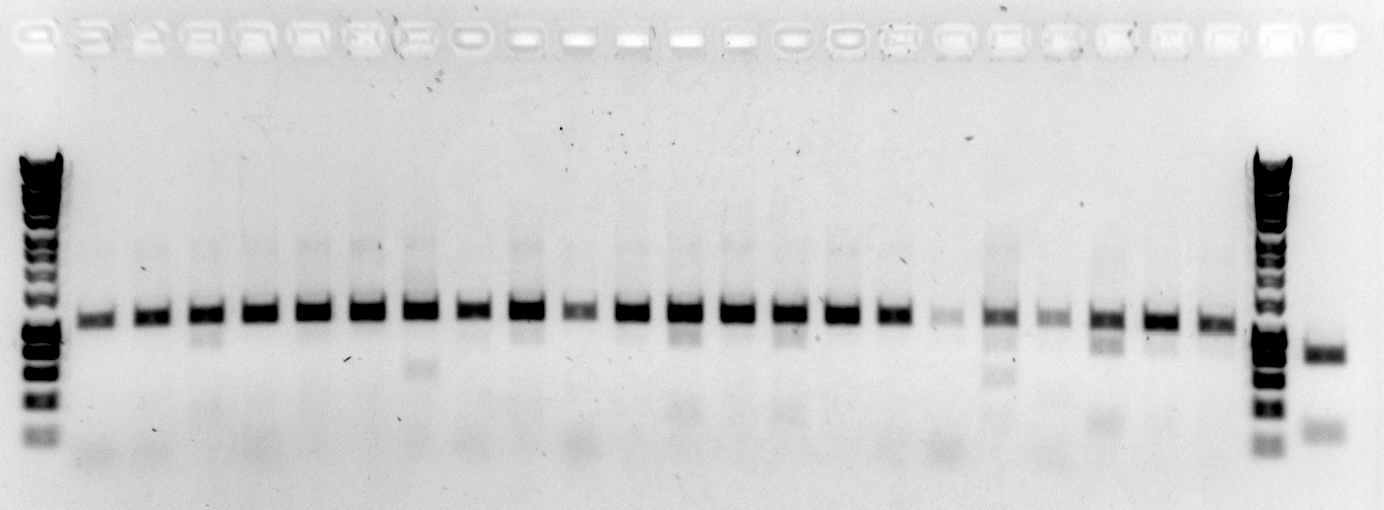

Supplement: Figure 1—figure supplement 2—source data 1. [file elife-81549-fig1-figsupp2-data1.zip › Figure_1_figure_supplement_2_source_data/Figure_1_figure_supplement_2_panel_D_source_data/Gel_pictures/Control digest_transp._fish_#1-41_ltk_Gel-oben_15.09.2017.tif]

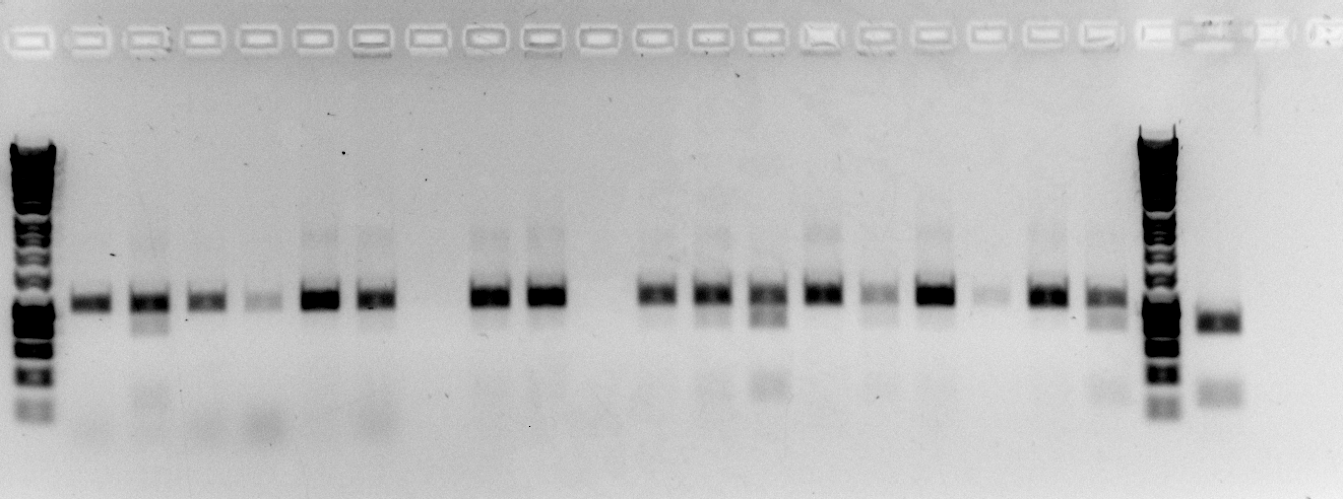

Supplement: Figure 1—figure supplement 2—source data 1. [file elife-81549-fig1-figsupp2-data1.zip › Figure_1_figure_supplement_2_source_data/Figure_1_figure_supplement_2_panel_D_source_data/Gel_pictures/Control digest_transp._fish_#1-41_ltk_Gel-unten_15.09.2017.tif]

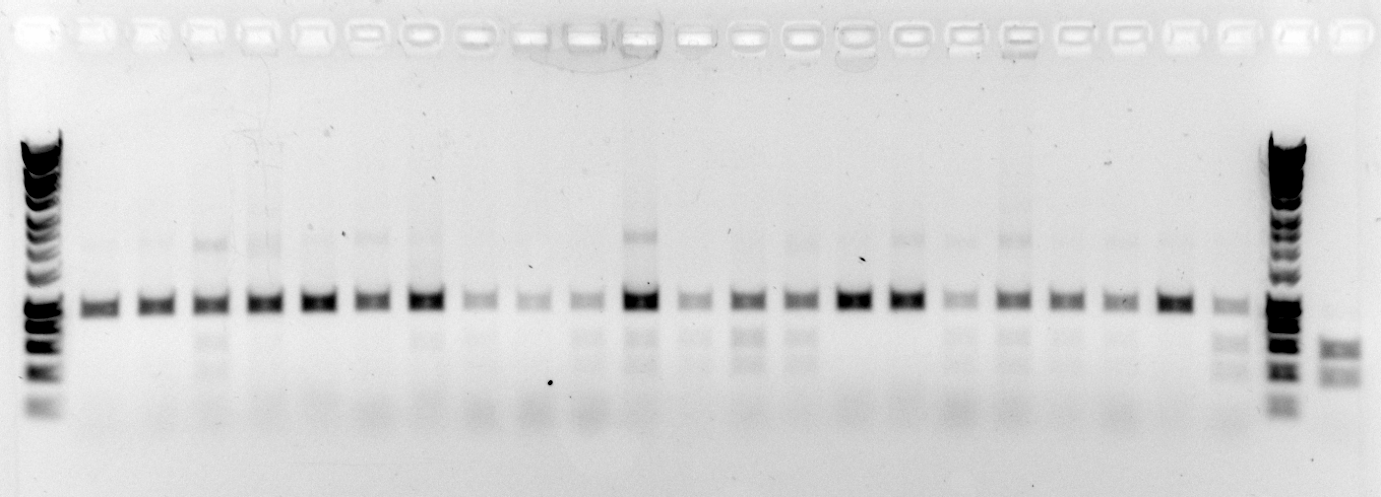

Supplement: Figure 1—figure supplement 2—source data 1. [file elife-81549-fig1-figsupp2-data1.zip › Figure_1_figure_supplement_2_source_data/Figure_1_figure_supplement_2_panel_D_source_data/Gel_pictures/Control digest_transp._fish_#1-41_mitfa_Gel-oben_15.09.2017.tif]

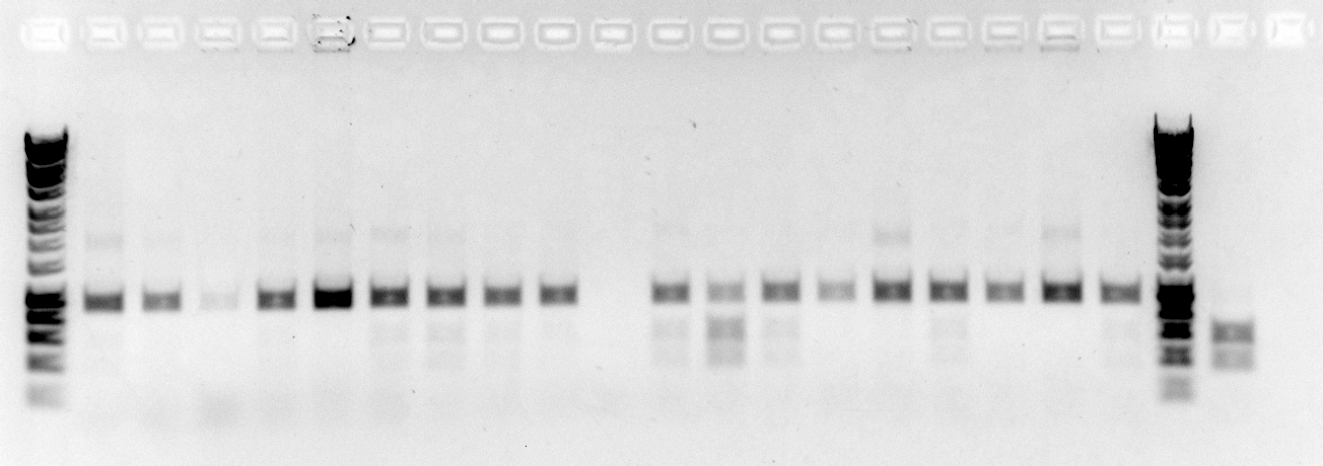

Supplement: Figure 1—figure supplement 2—source data 1. [file elife-81549-fig1-figsupp2-data1.zip › Figure_1_figure_supplement_2_source_data/Figure_1_figure_supplement_2_panel_D_source_data/Gel_pictures/Control digest_transp._fish_#1-41_mitfa_Gel-unten_15.09.2017.tif]

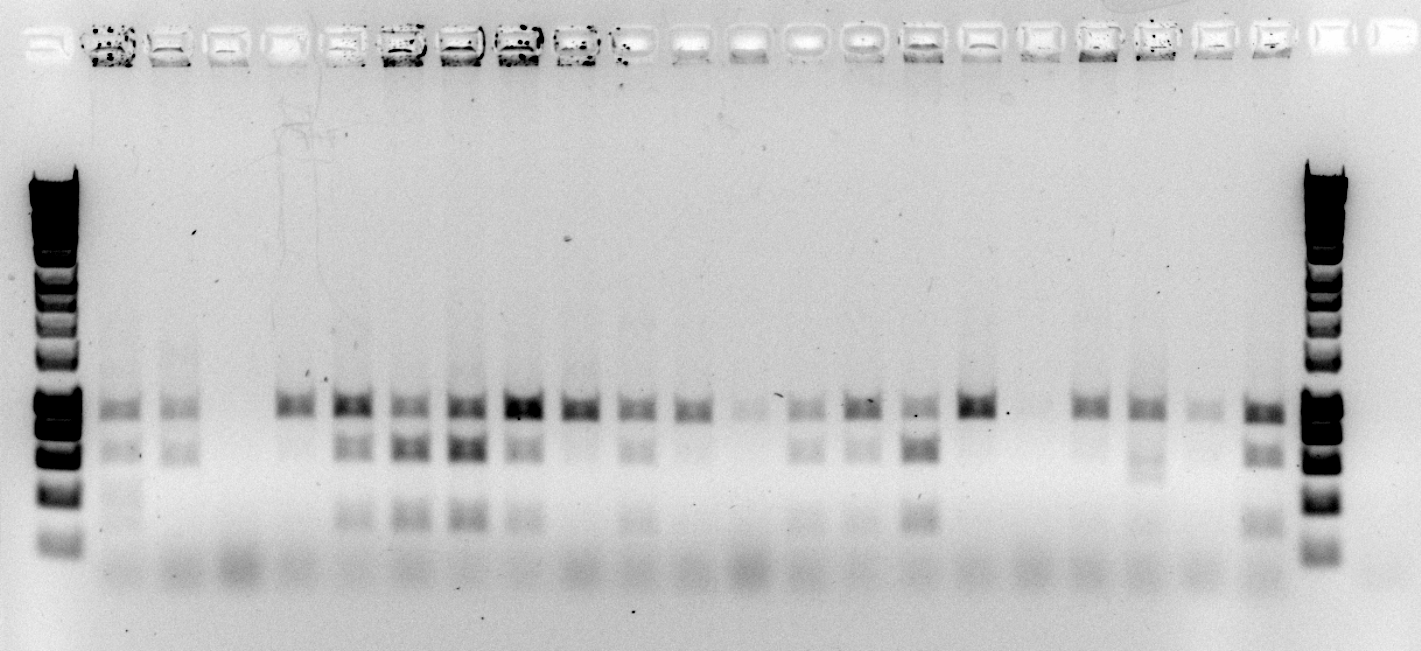

Supplement: Figure 1—figure supplement 2—source data 1. [file elife-81549-fig1-figsupp2-data1.zip › Figure_1_figure_supplement_2_source_data/Figure_1_figure_supplement_2_panel_D_source_data/Gel_pictures/Control digest_transp._fish_#42-89_csf1ra_Gel1-oben_27.09.2017.tif]

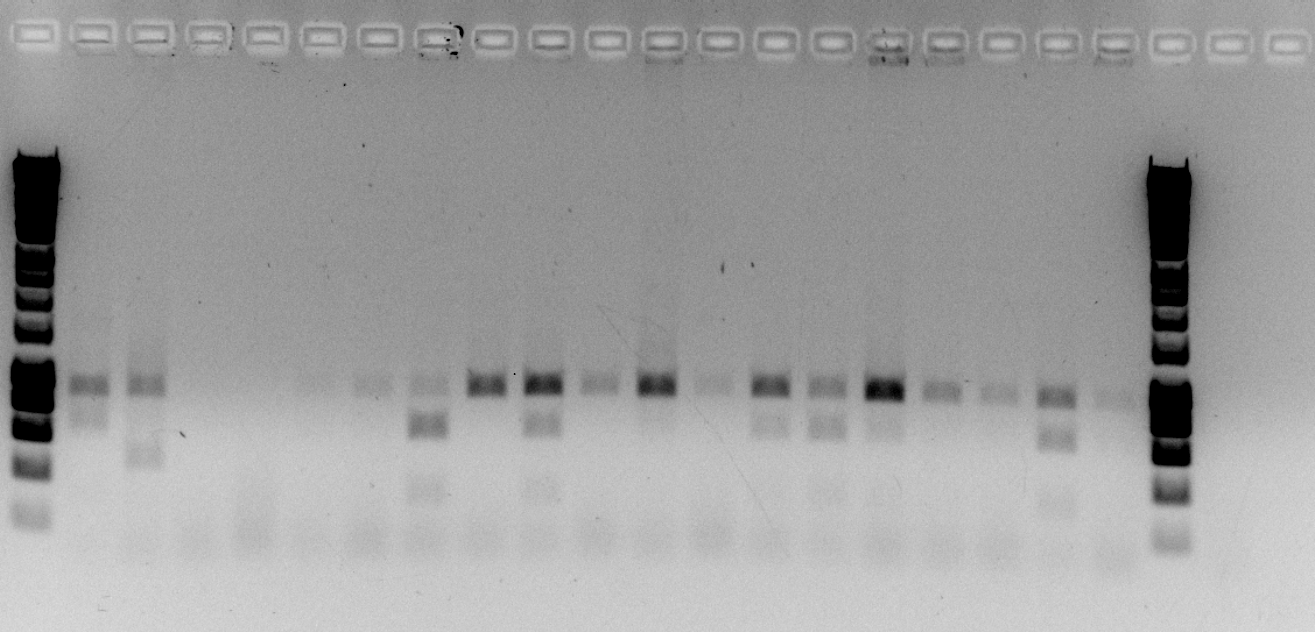

Supplement: Figure 1—figure supplement 2—source data 1. [file elife-81549-fig1-figsupp2-data1.zip › Figure_1_figure_supplement_2_source_data/Figure_1_figure_supplement_2_panel_D_source_data/Gel_pictures/Control digest_transp._fish_#42-89_csf1ra_Gel1-unten_27.09.2017.tif]

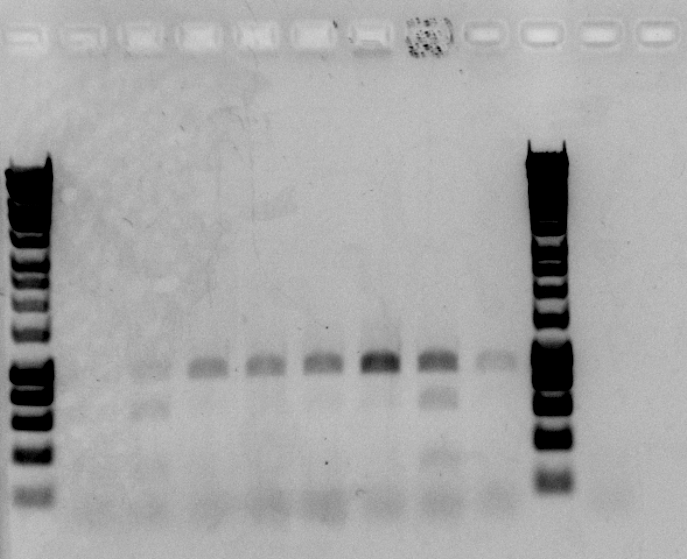

Supplement: Figure 1—figure supplement 2—source data 1. [file elife-81549-fig1-figsupp2-data1.zip › Figure_1_figure_supplement_2_source_data/Figure_1_figure_supplement_2_panel_D_source_data/Gel_pictures/Control digest_transp._fish_#42-89_csf1ra_Gel2_27.09.2017.tif]

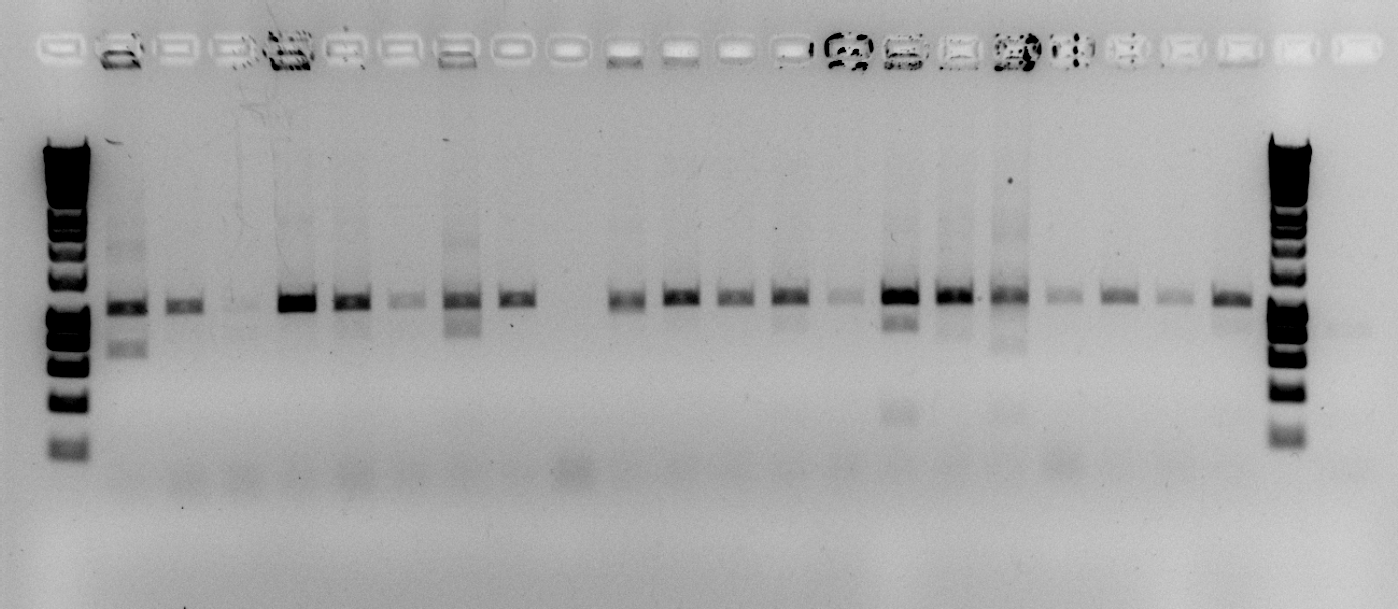

Supplement: Figure 1—figure supplement 2—source data 1. [file elife-81549-fig1-figsupp2-data1.zip › Figure_1_figure_supplement_2_source_data/Figure_1_figure_supplement_2_panel_D_source_data/Gel_pictures/Control digest_transp._fish_#42-89_ltk_Gel1-oben_27.09.2017.tif]

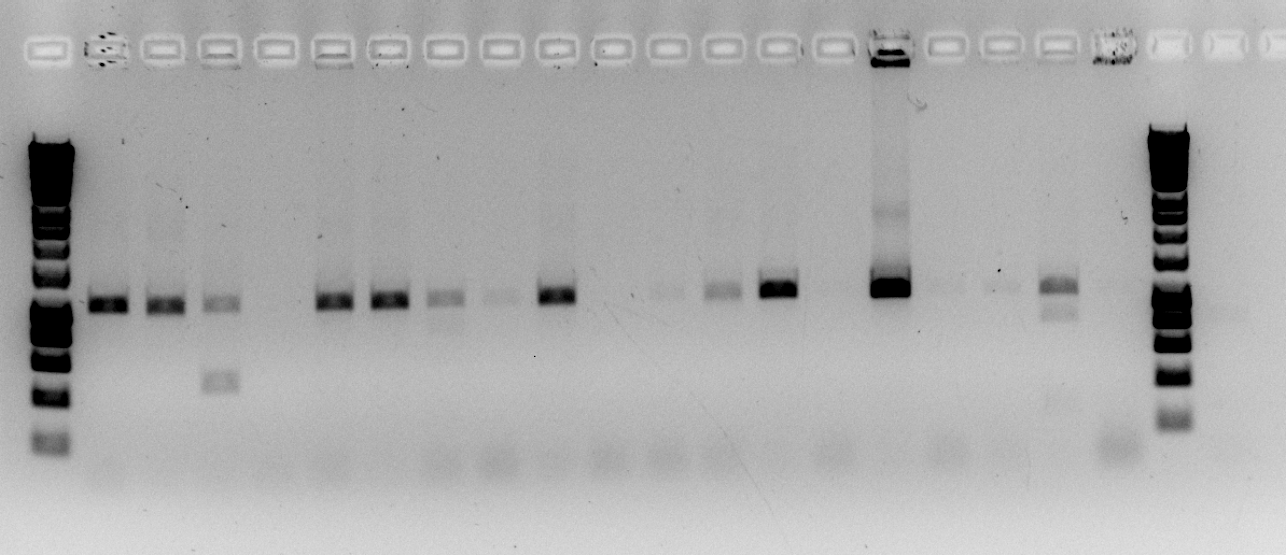

Supplement: Figure 1—figure supplement 2—source data 1. [file elife-81549-fig1-figsupp2-data1.zip › Figure_1_figure_supplement_2_source_data/Figure_1_figure_supplement_2_panel_D_source_data/Gel_pictures/Control digest_transp._fish_#42-89_ltk_Gel1-unten_27.09.2017.tif]

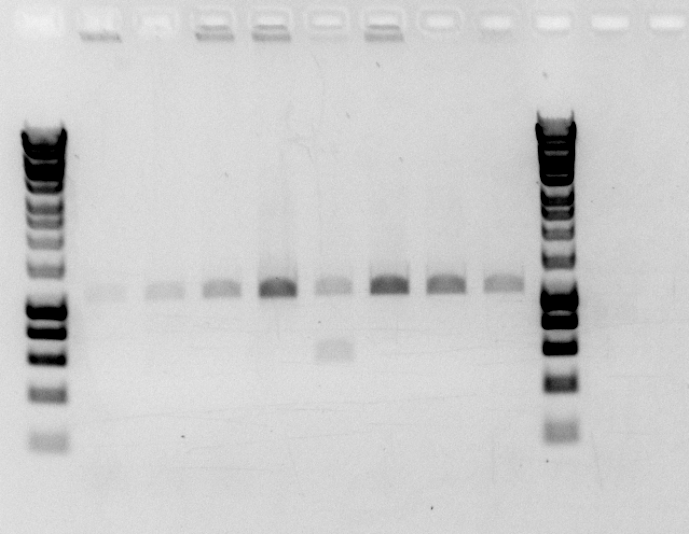

Supplement: Figure 1—figure supplement 2—source data 1. [file elife-81549-fig1-figsupp2-data1.zip › Figure_1_figure_supplement_2_source_data/Figure_1_figure_supplement_2_panel_D_source_data/Gel_pictures/Control digest_transp._fish_#42-89_ltk_Gel2_27.09.2017.tif]

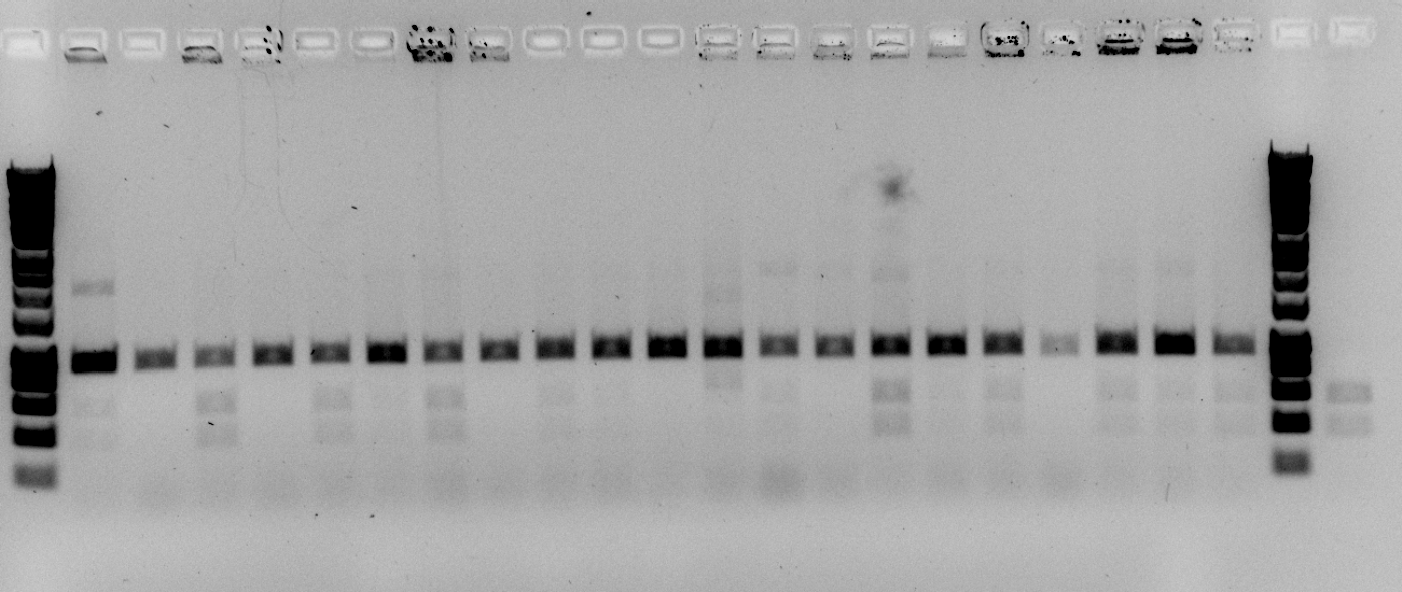

Supplement: Figure 1—figure supplement 2—source data 1. [file elife-81549-fig1-figsupp2-data1.zip › Figure_1_figure_supplement_2_source_data/Figure_1_figure_supplement_2_panel_D_source_data/Gel_pictures/Control digest_transp._fish_#42-89_mitfa_Gel1-oben_27.09.2017.tif]

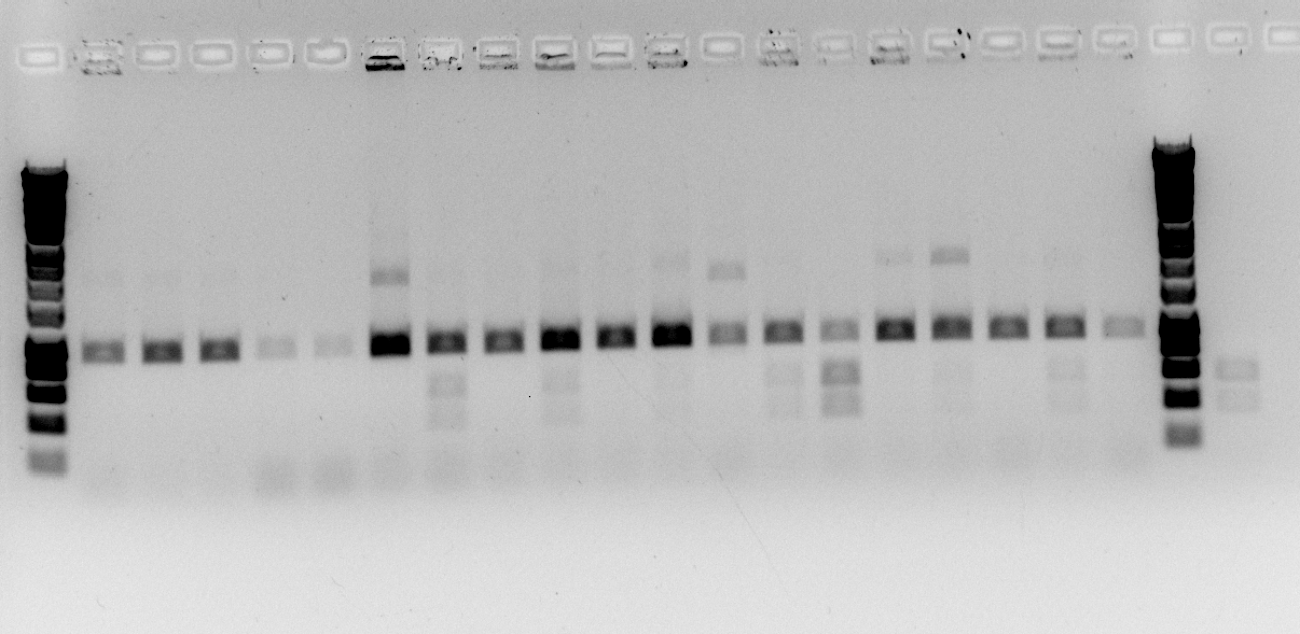

Supplement: Figure 1—figure supplement 2—source data 1. [file elife-81549-fig1-figsupp2-data1.zip › Figure_1_figure_supplement_2_source_data/Figure_1_figure_supplement_2_panel_D_source_data/Gel_pictures/Control digest_transp._fish_#42-89_mitfa_Gel1-unten_27.09.2017.tif]

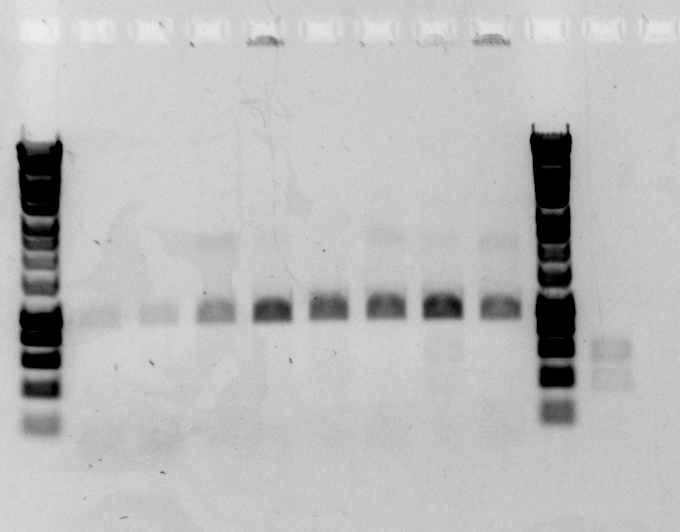

Supplement: Figure 1—figure supplement 2—source data 1. [file elife-81549-fig1-figsupp2-data1.zip › Figure_1_figure_supplement_2_source_data/Figure_1_figure_supplement_2_panel_D_source_data/Gel_pictures/Control digest_transp._fish_#42-89_mitfa_Gel2_27.09.2017.tif]

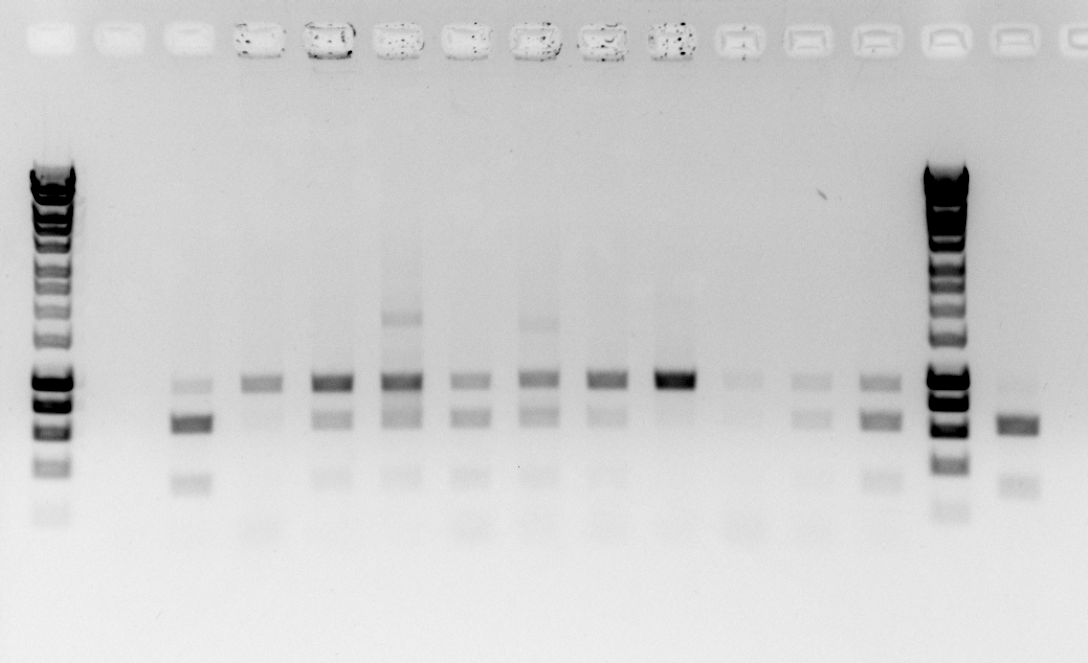

Supplement: Figure 1—figure supplement 2—source data 1. [file elife-81549-fig1-figsupp2-data1.zip › Figure_1_figure_supplement_2_source_data/Figure_1_figure_supplement_2_panel_D_source_data/Gel_pictures/Control digest_transp._fish_#Wdh_csf1ra_29.09.2017.tif]

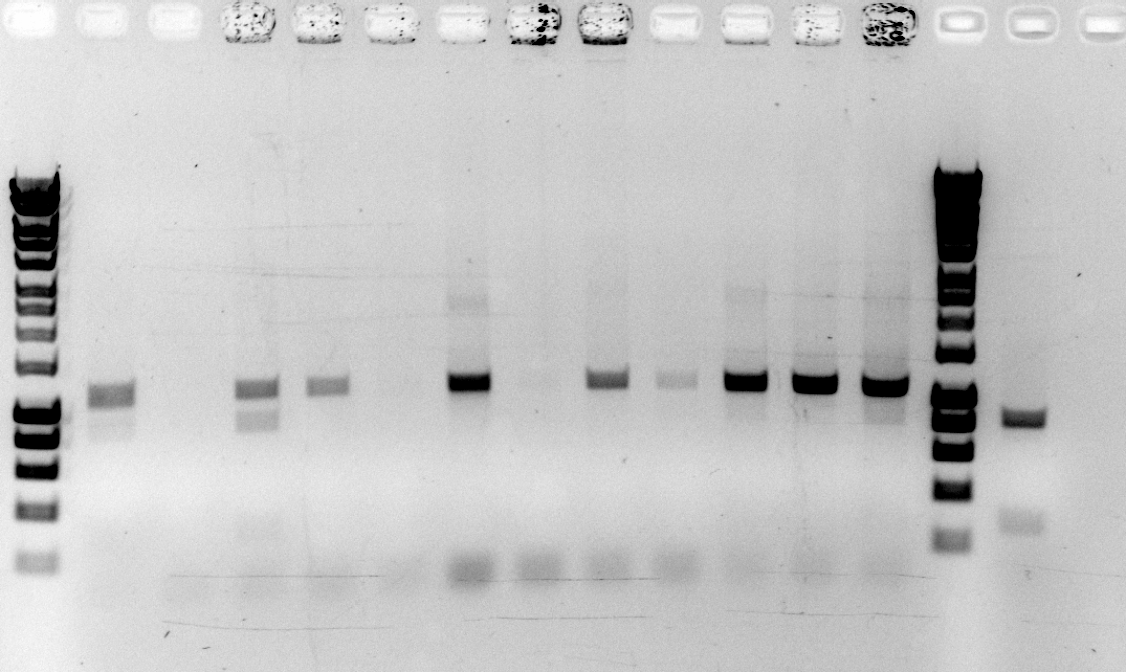

Supplement: Figure 1—figure supplement 2—source data 1. [file elife-81549-fig1-figsupp2-data1.zip › Figure_1_figure_supplement_2_source_data/Figure_1_figure_supplement_2_panel_D_source_data/Gel_pictures/Control digest_transp._fish_#Wdh_ltk_29.09.2017.tif]

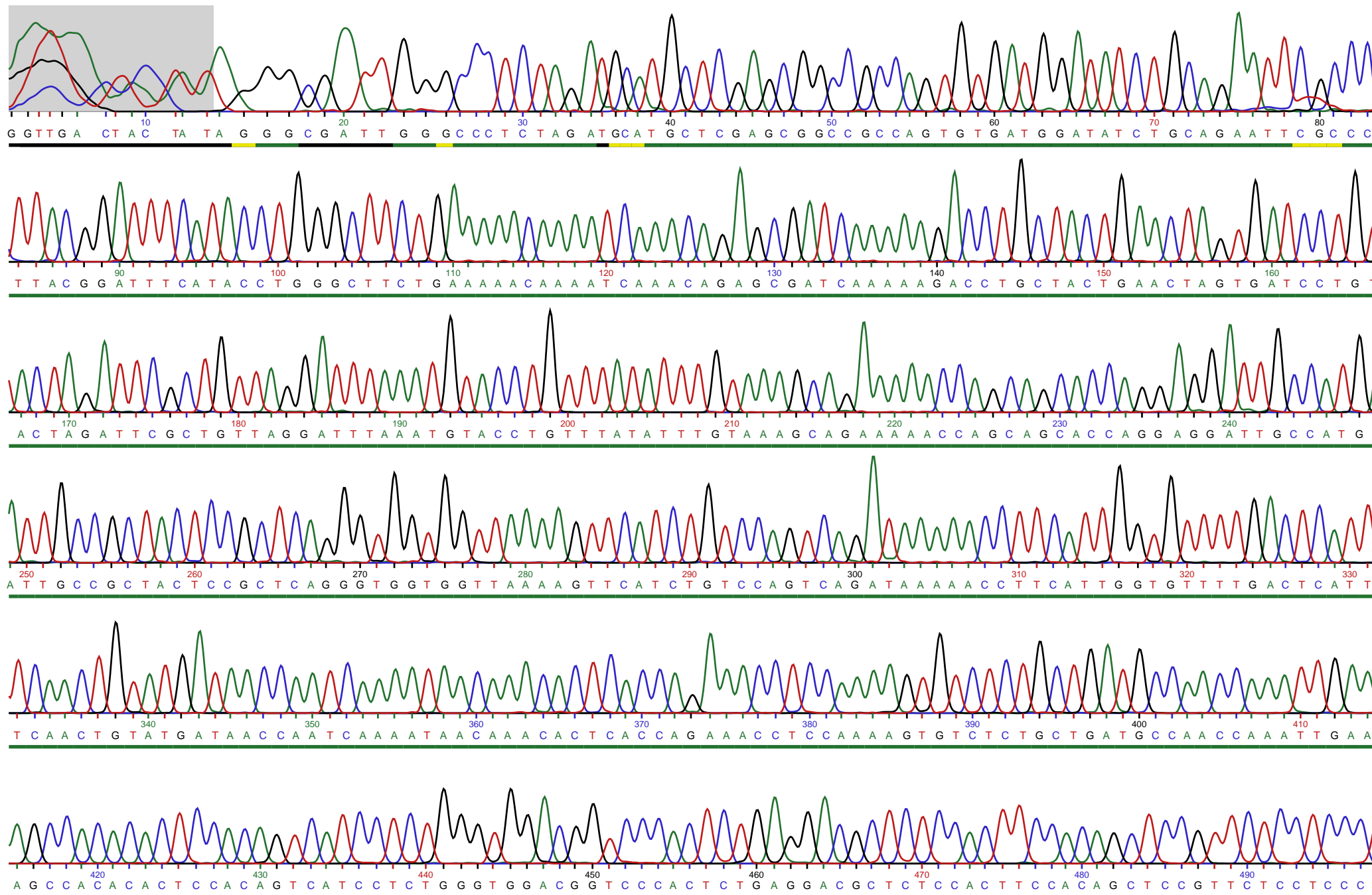

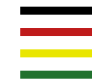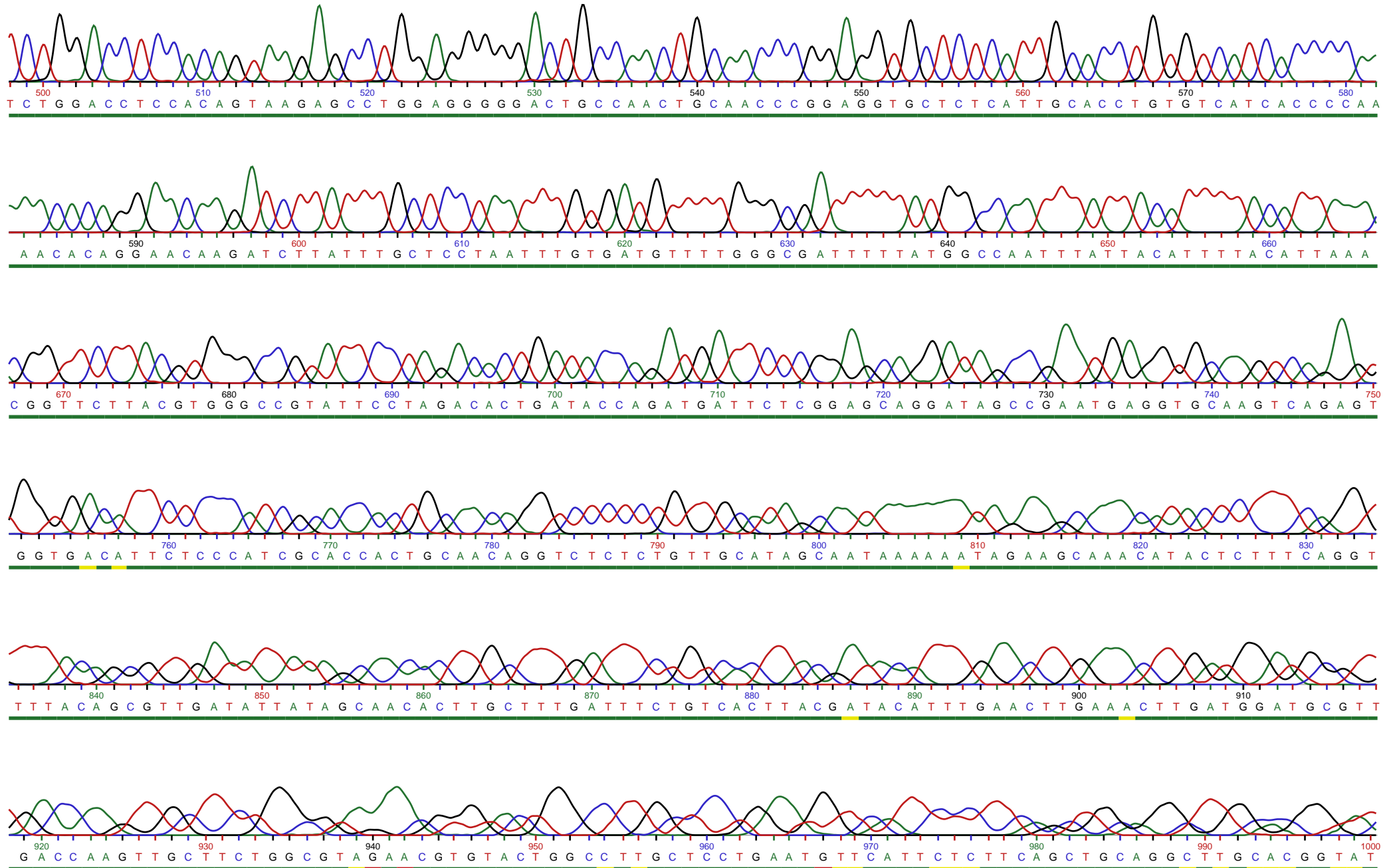

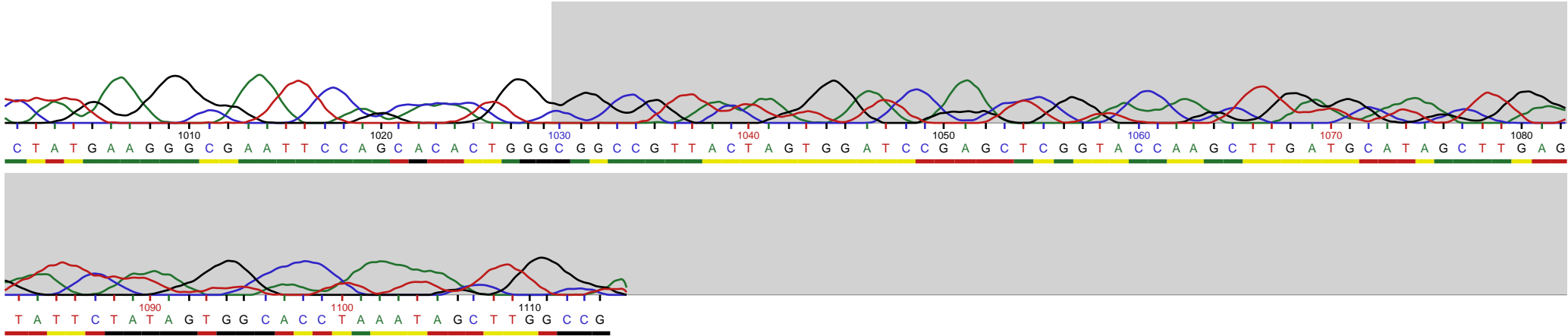

Supplement: Figure 1—figure supplement 3—source data 1. [file elife-81549-fig1-figsupp3-data1.zip › Figure_1_figure_supplement_3_source_data/Figure_1_figure_supplement_3_panel_ABC_source_data/Originals_F1_sequencing/Fish_1/csf1ra/csf #1c_M13uni-21.pdf]

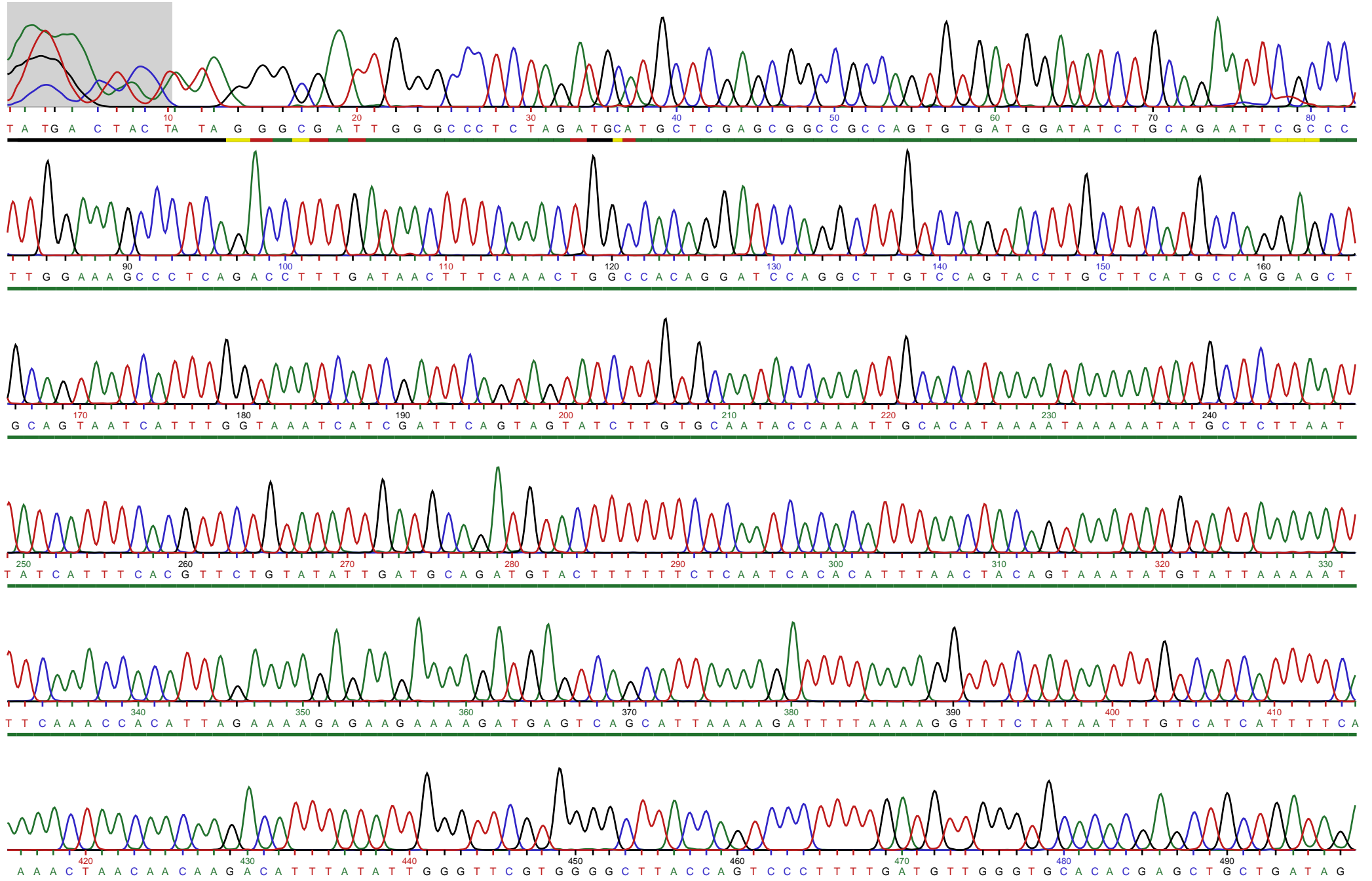

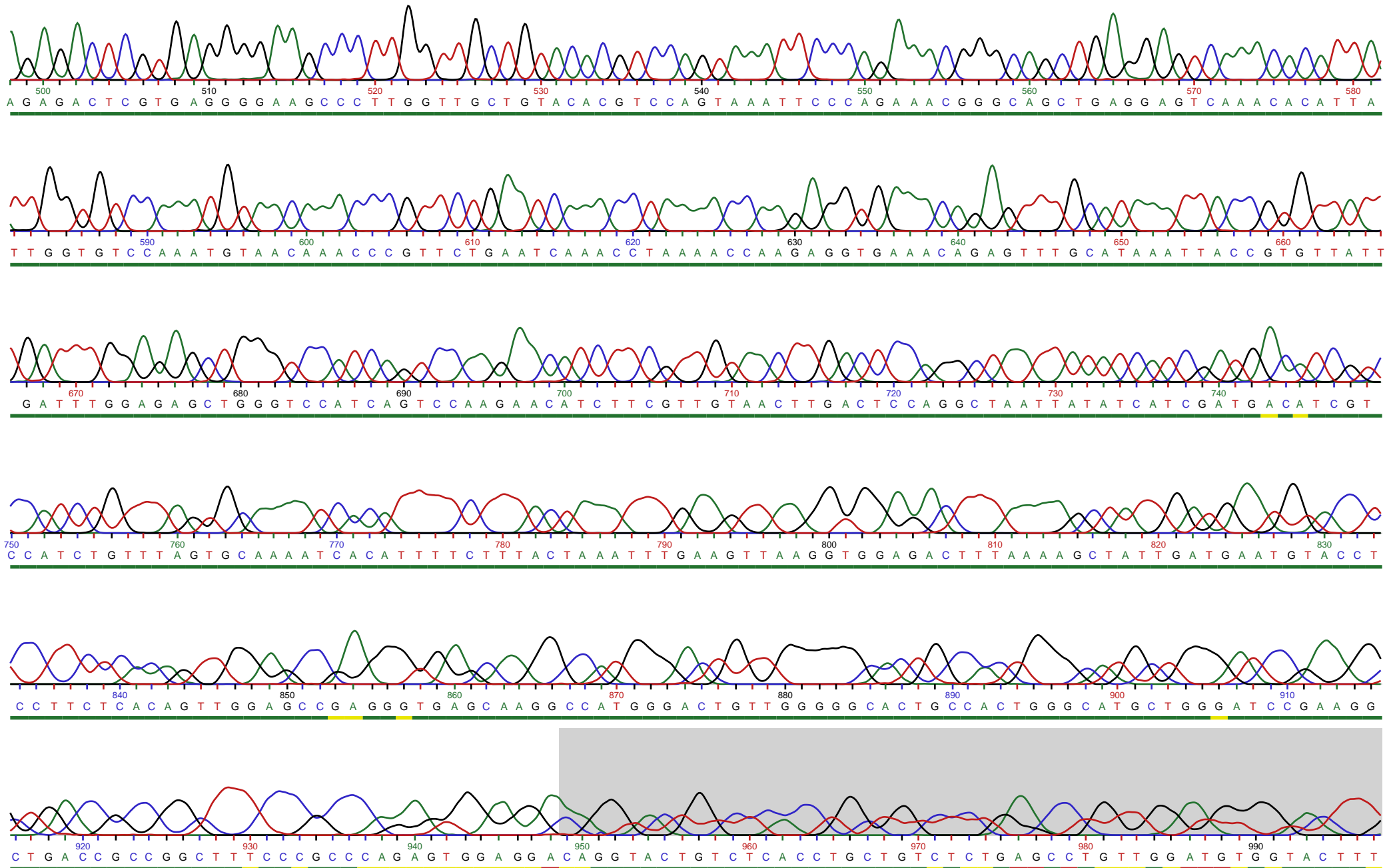

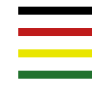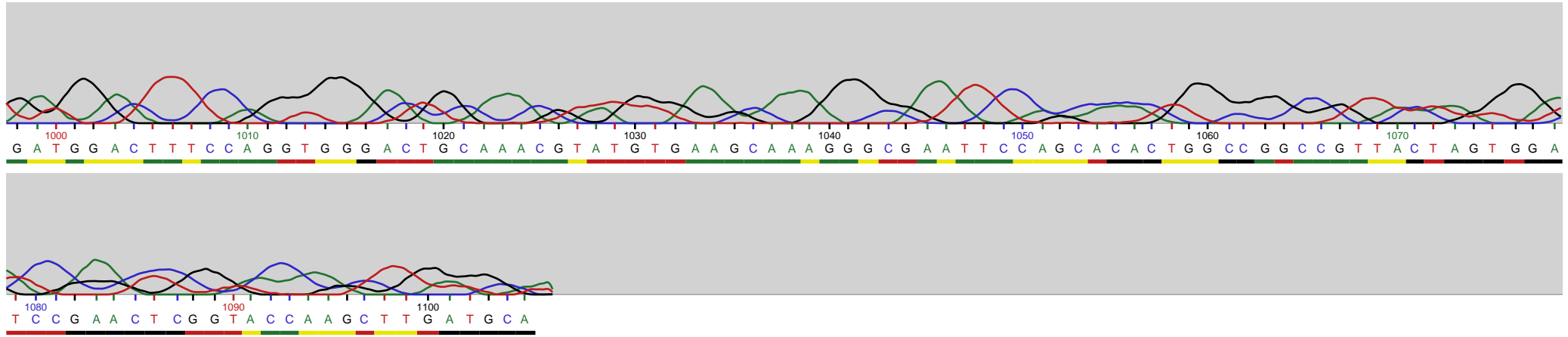

Supplement: Figure 1—figure supplement 3—source data 1. [file elife-81549-fig1-figsupp3-data1.zip › Figure_1_figure_supplement_3_source_data/Figure_1_figure_supplement_3_panel_ABC_source_data/Originals_F1_sequencing/Fish_1/mitfa/mitfa #1b_M13uni-21.pdf]

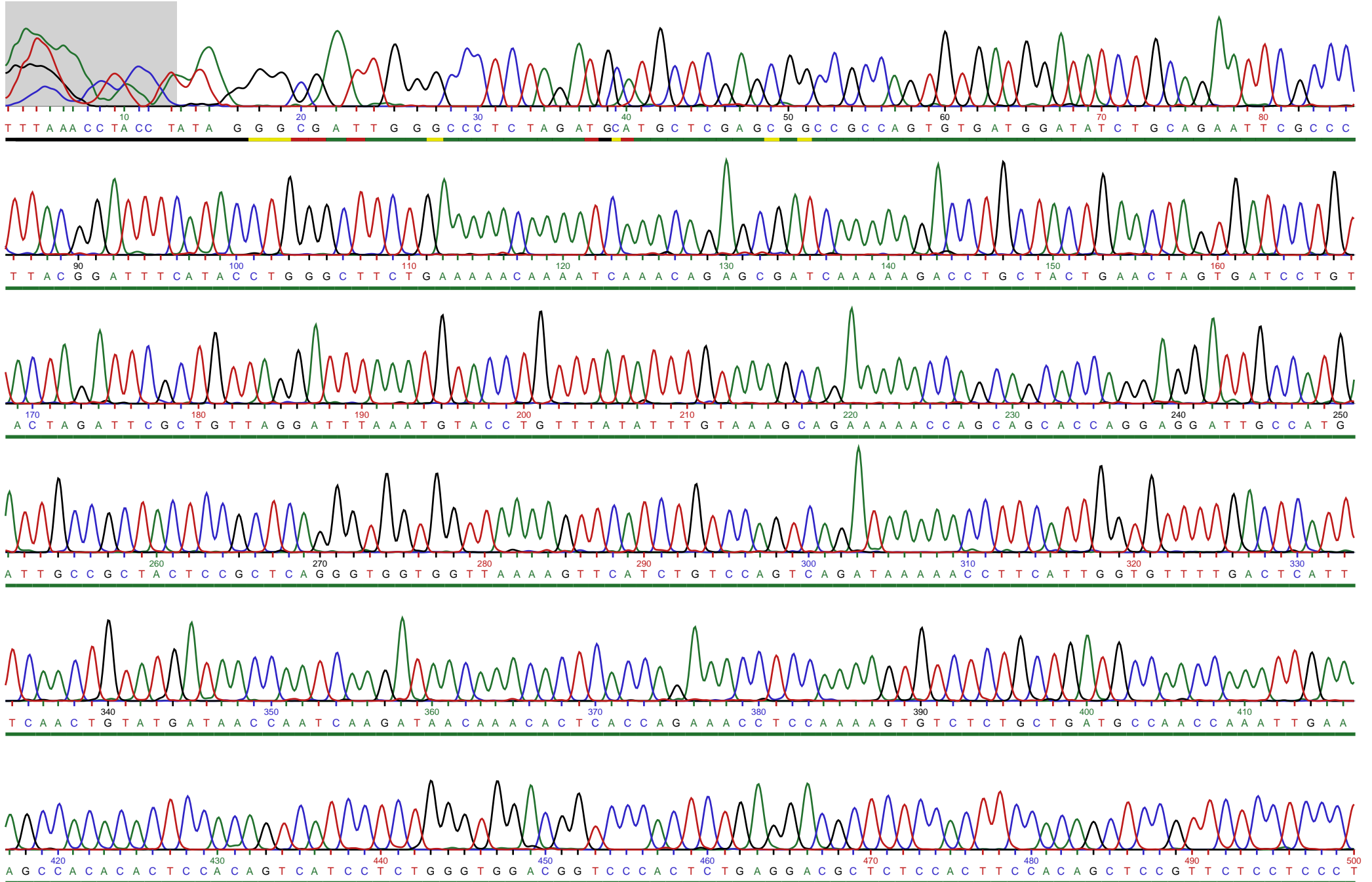

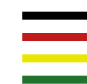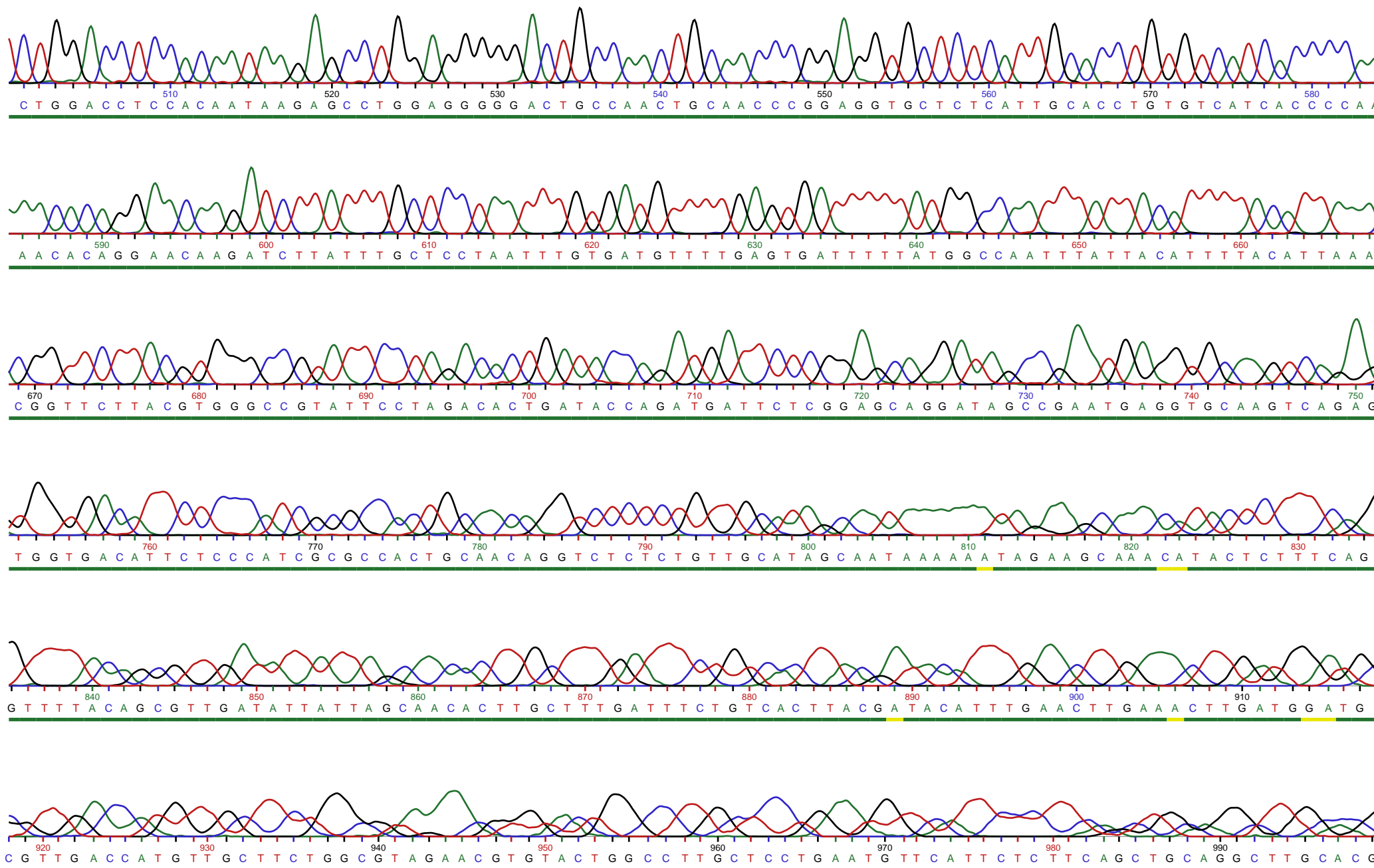

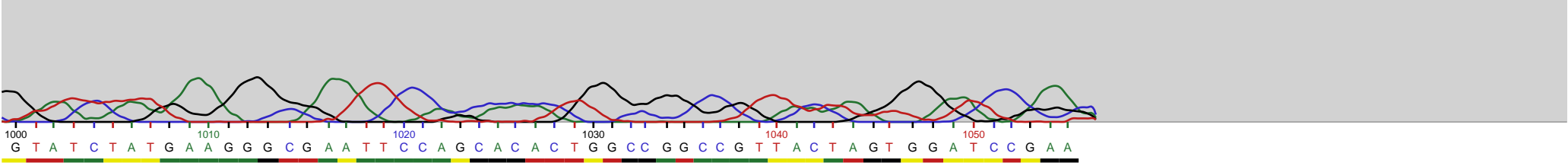

Supplement: Figure 1—figure supplement 3—source data 1. [file elife-81549-fig1-figsupp3-data1.zip › Figure_1_figure_supplement_3_source_data/Figure_1_figure_supplement_3_panel_ABC_source_data/Originals_F1_sequencing/Fish_11/csf1ra/csf #11a_M13uni-21.pdf]

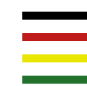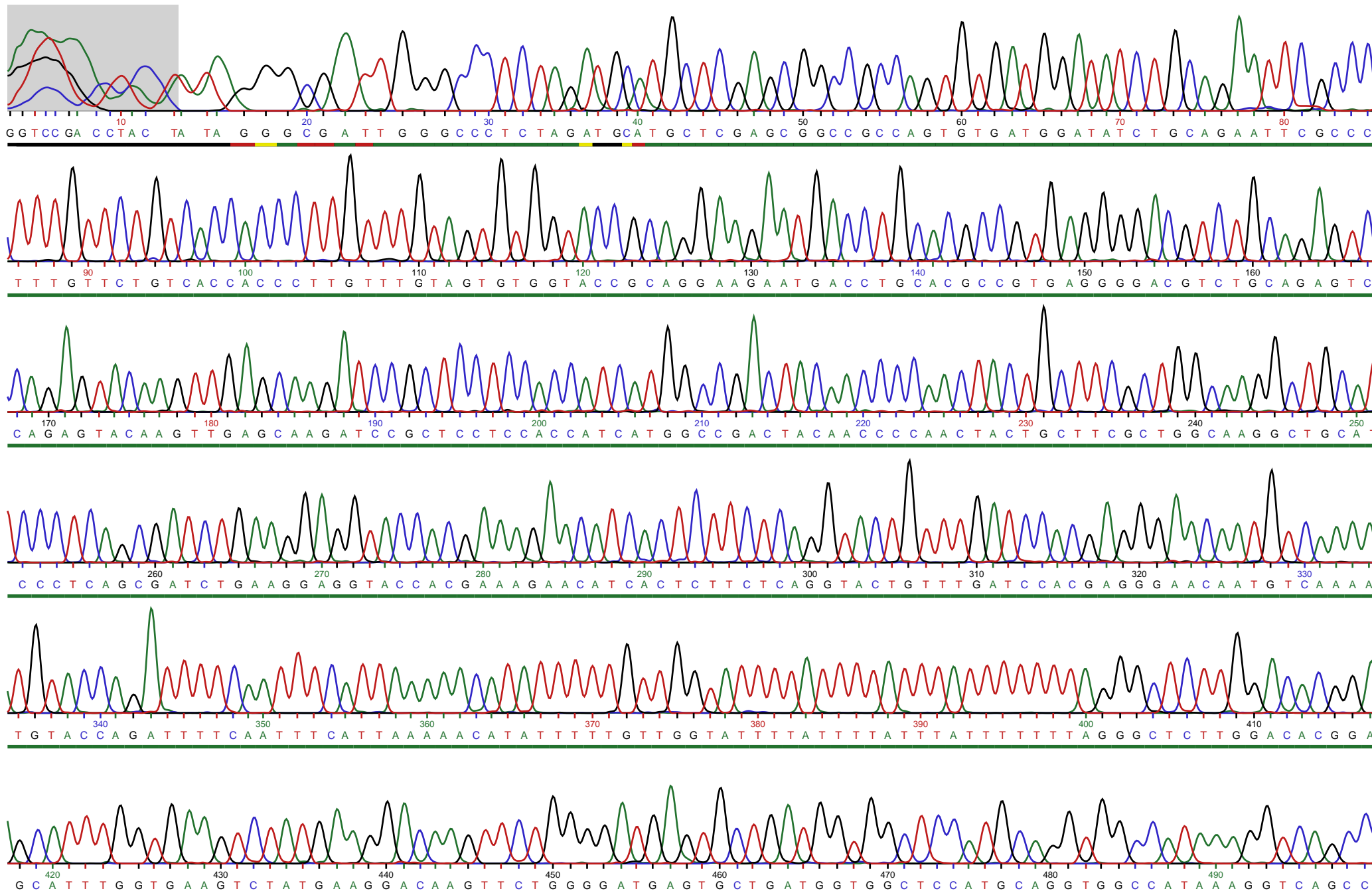

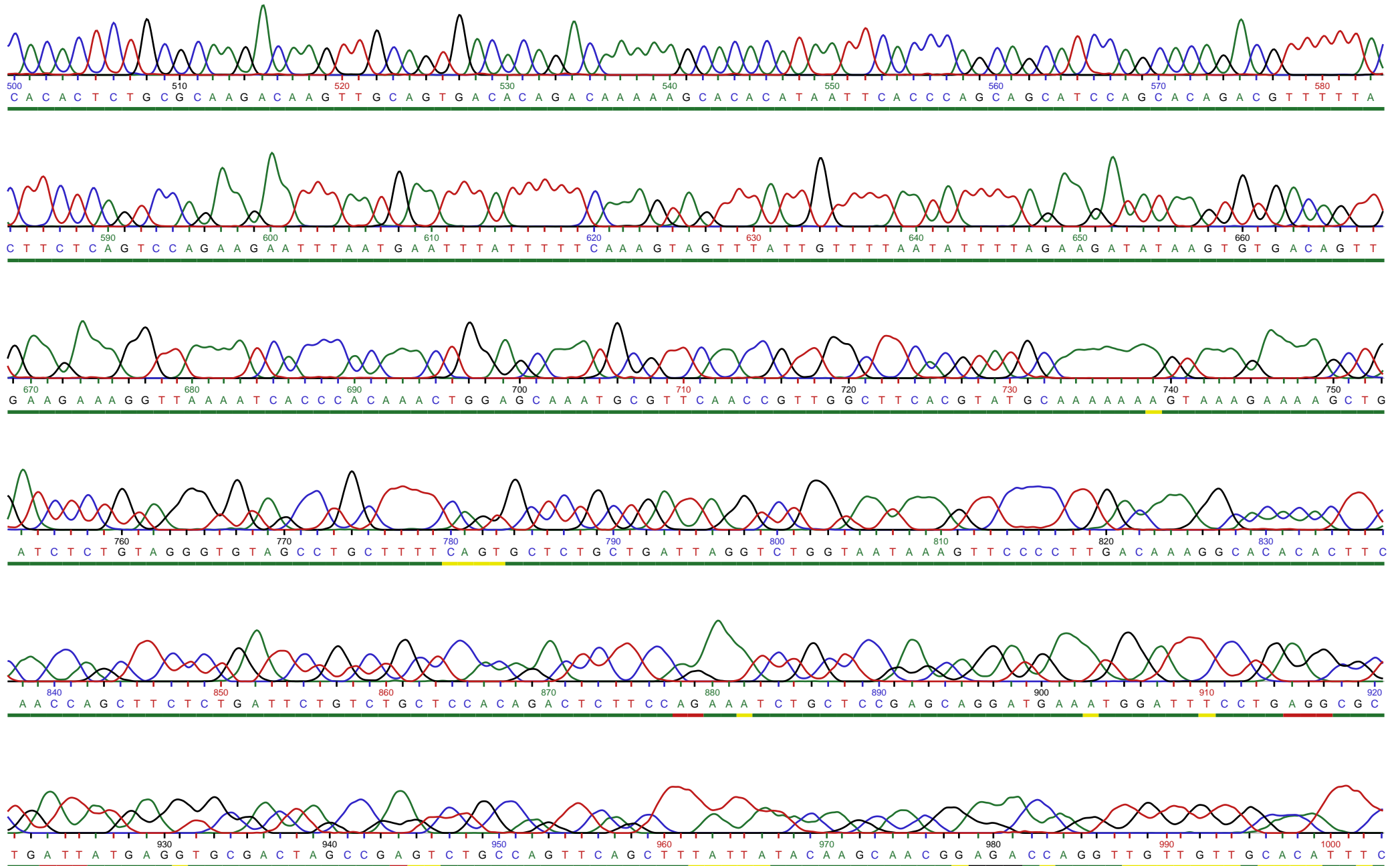

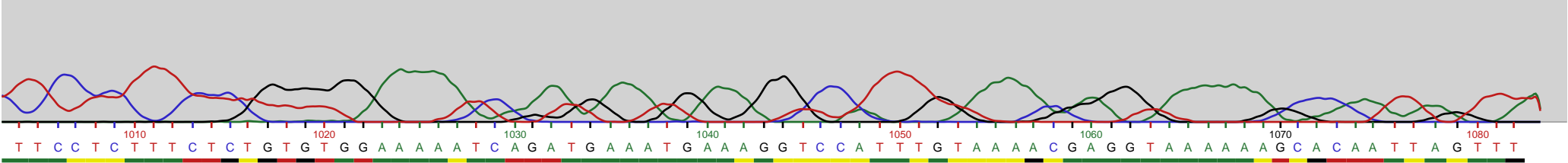

Supplement: Figure 1—figure supplement 3—source data 1. [file elife-81549-fig1-figsupp3-data1.zip › Figure_1_figure_supplement_3_source_data/Figure_1_figure_supplement_3_panel_ABC_source_data/Originals_F1_sequencing/Fish_11/ltk/ltk #11d_M13uni-21.pdf]

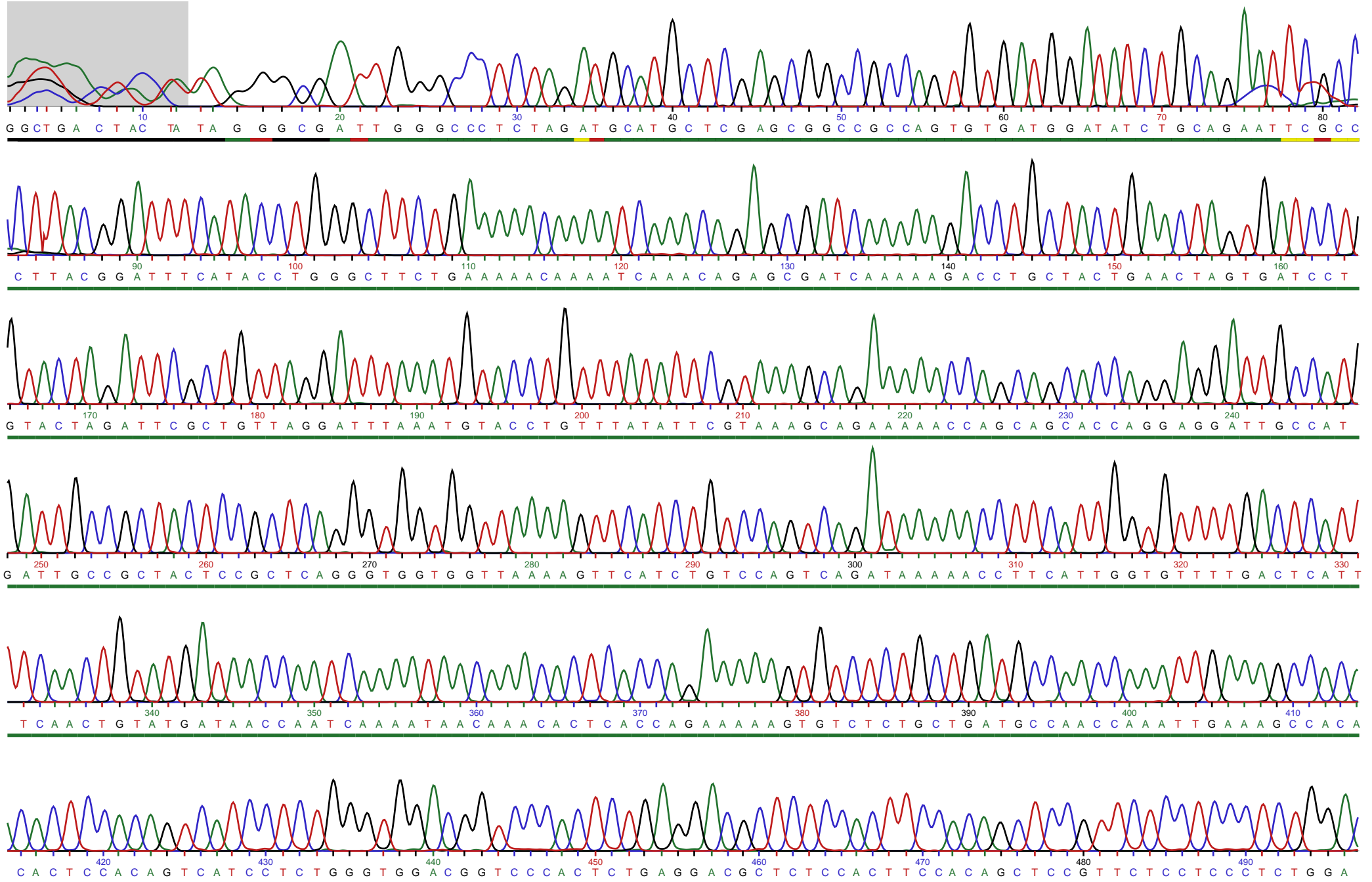

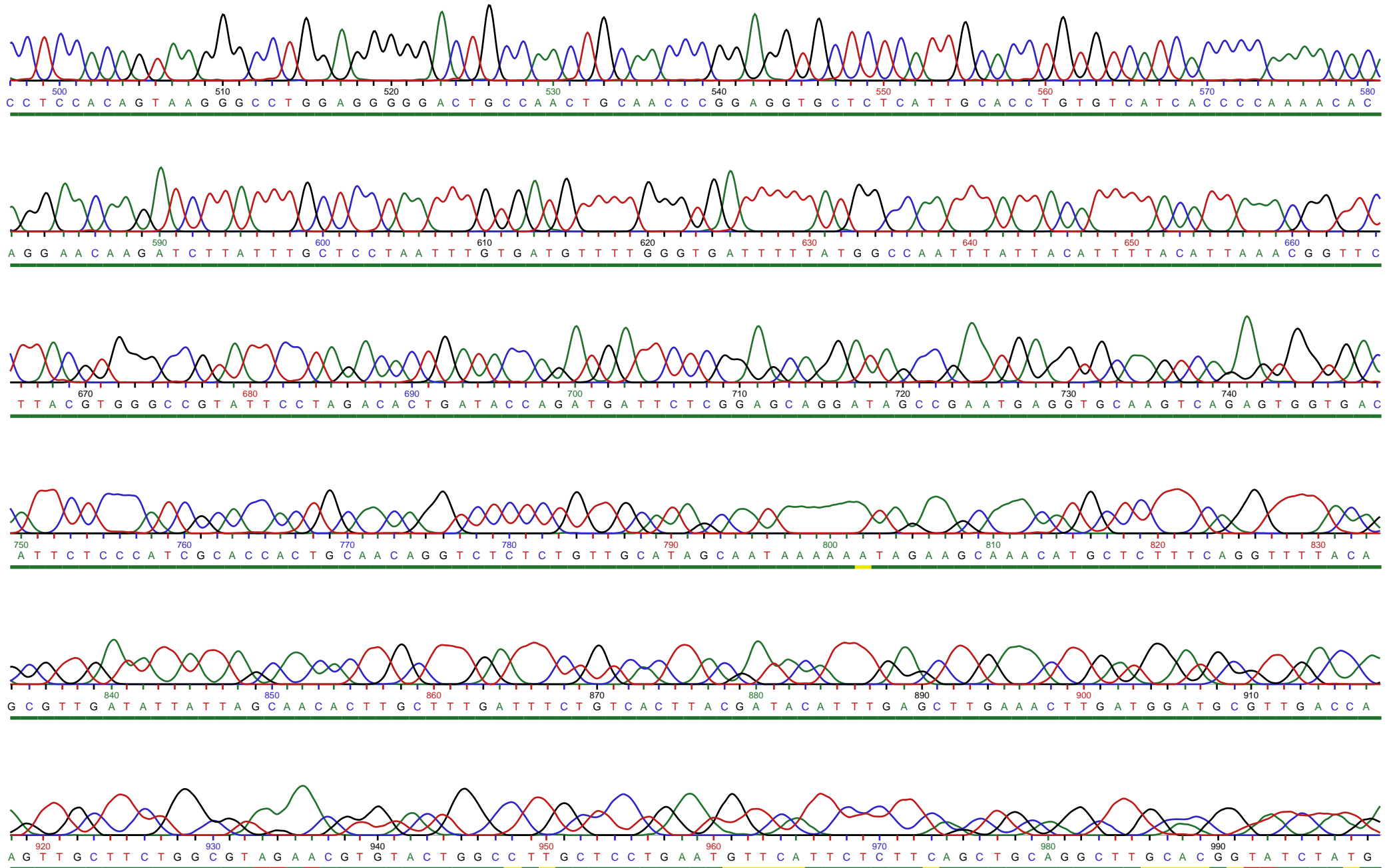

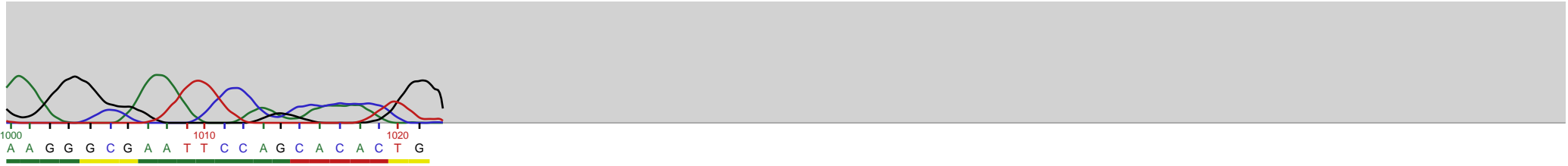

Supplement: Figure 1—figure supplement 3—source data 1. [file elife-81549-fig1-figsupp3-data1.zip › Figure_1_figure_supplement_3_source_data/Figure_1_figure_supplement_3_panel_ABC_source_data/Originals_F1_sequencing/Fish_18/csf1ra/csf #18f_M13uni-21.pdf]

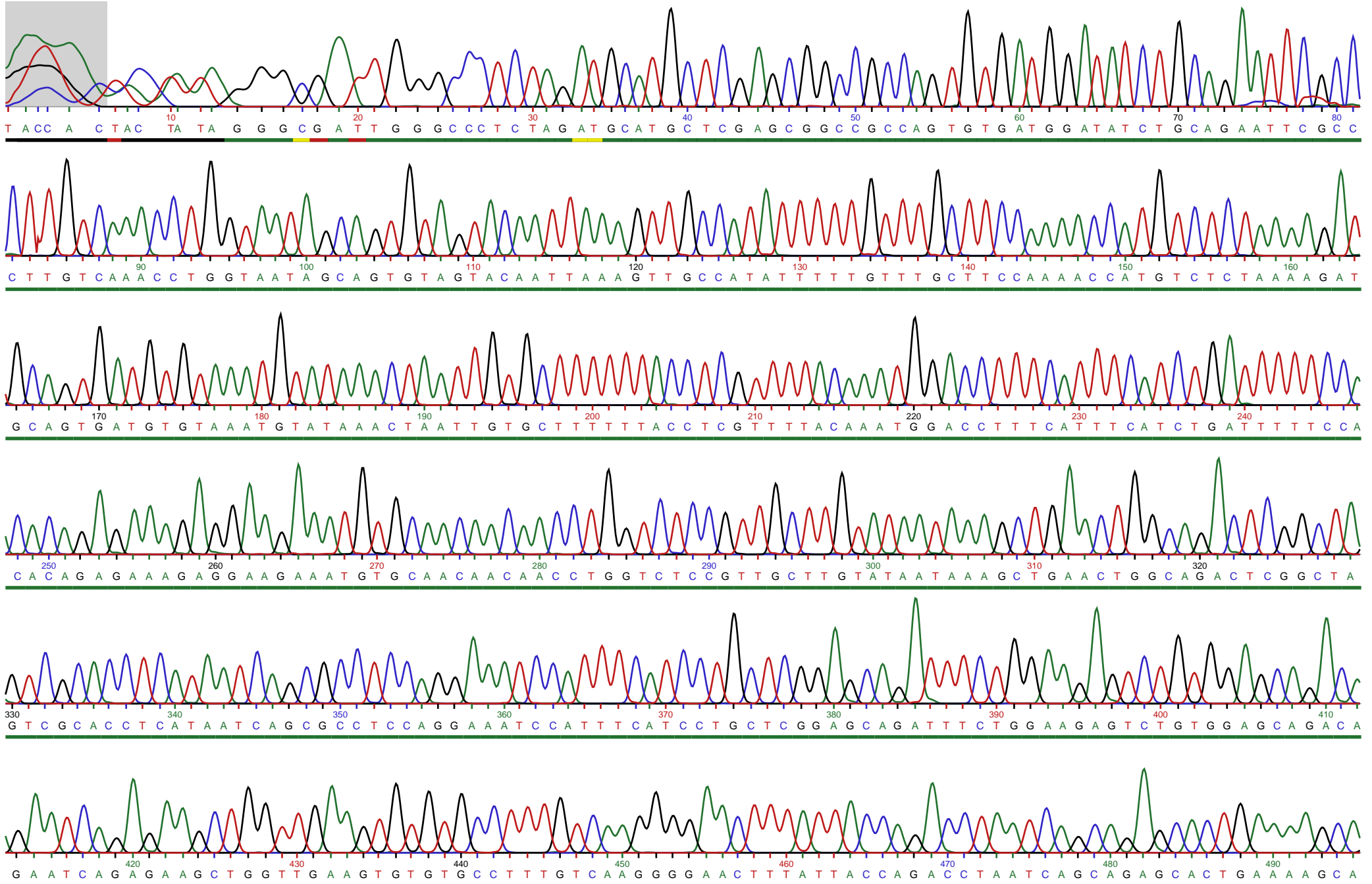

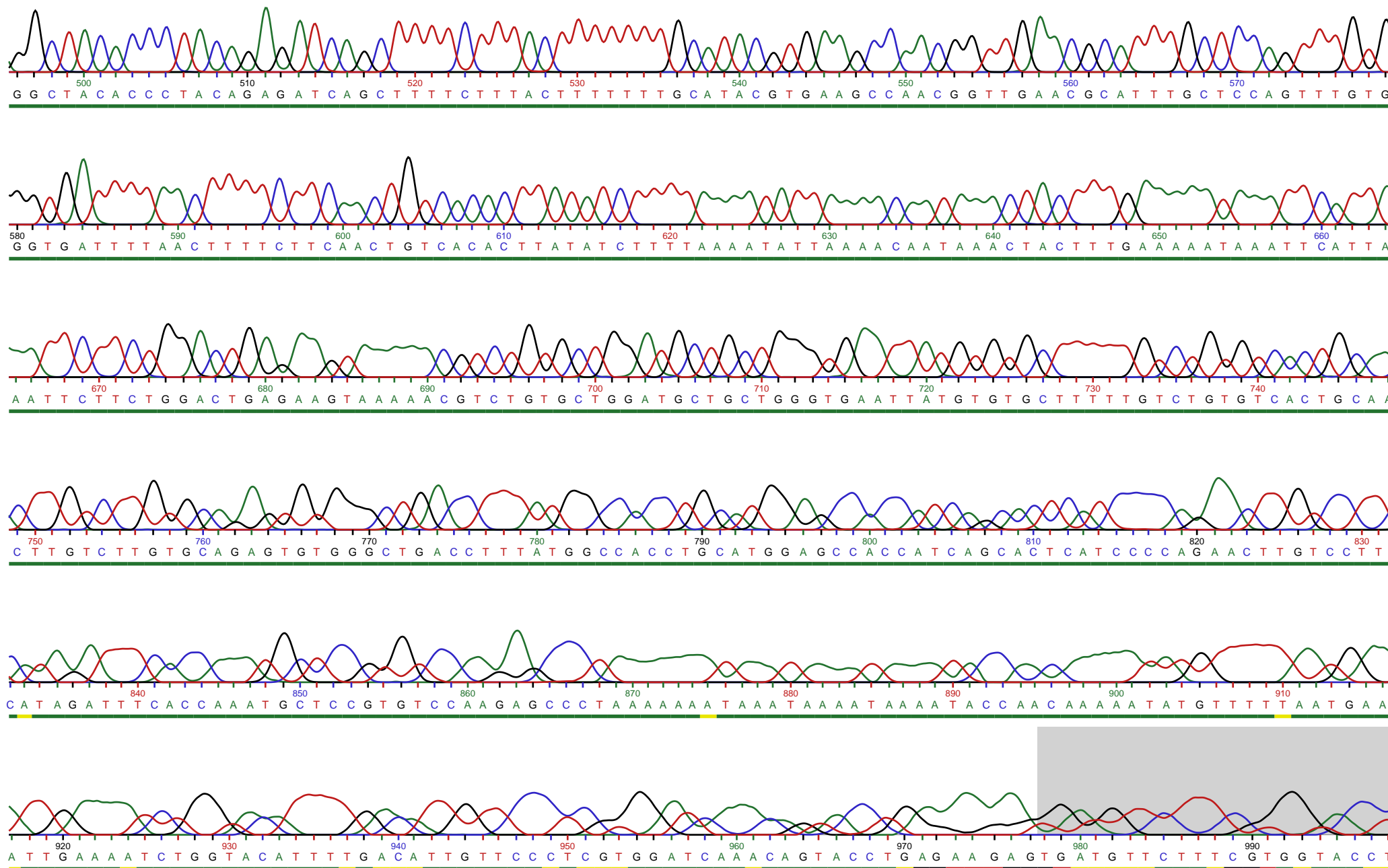

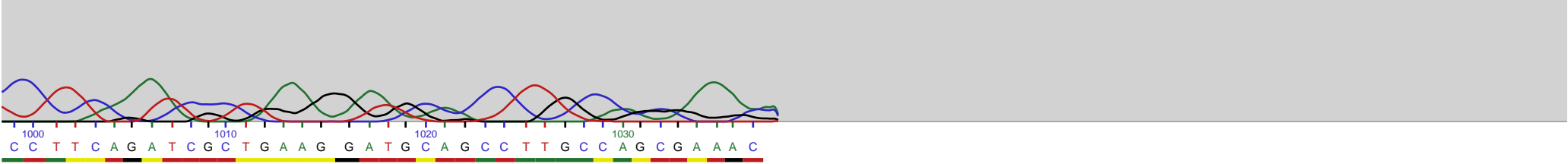

Supplement: Figure 1—figure supplement 3—source data 1. [file elife-81549-fig1-figsupp3-data1.zip › Figure_1_figure_supplement_3_source_data/Figure_1_figure_supplement_3_panel_ABC_source_data/Originals_F1_sequencing/Fish_18/ltk/ltk #18c_M13uni-21.pdf]

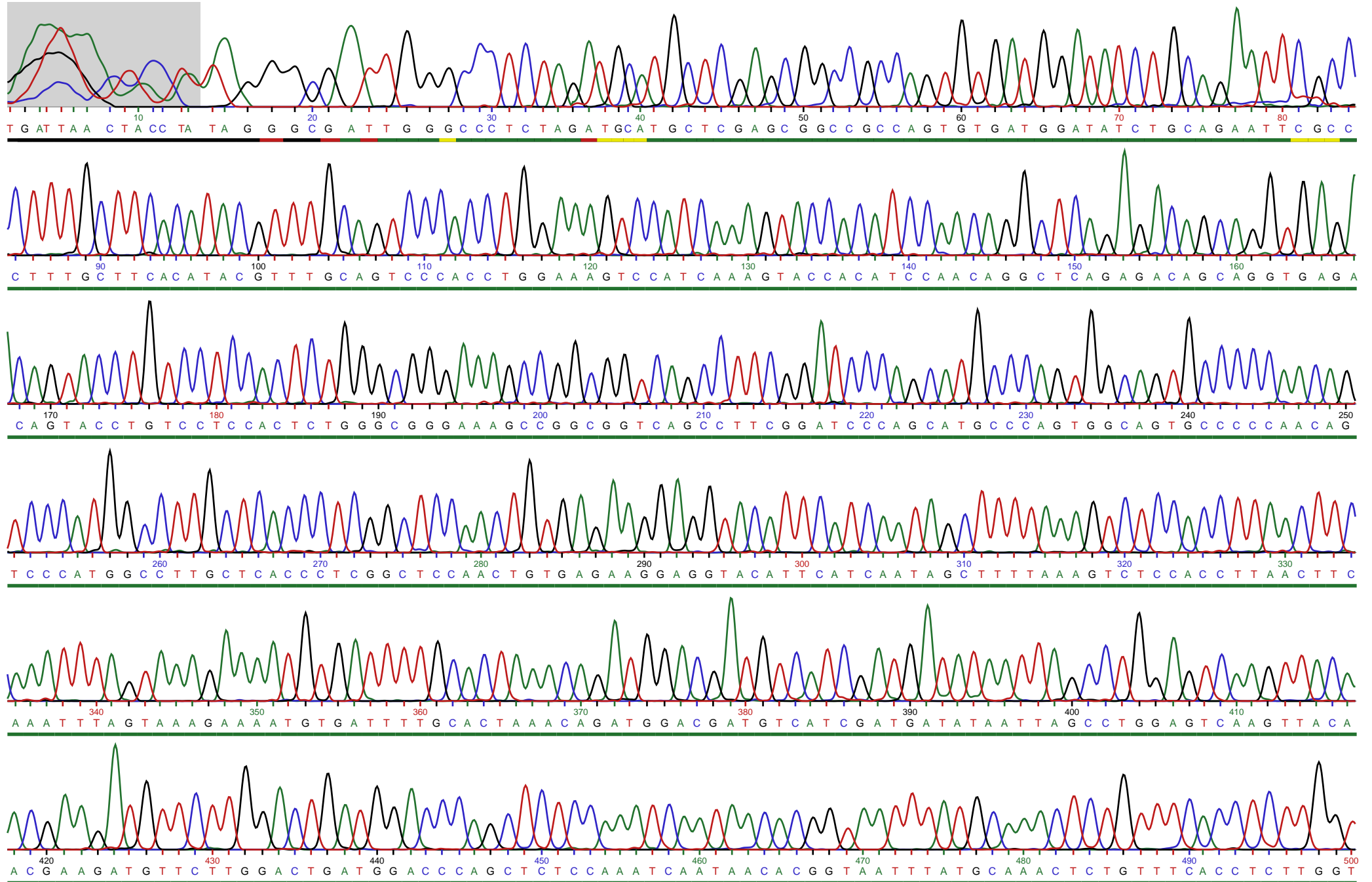

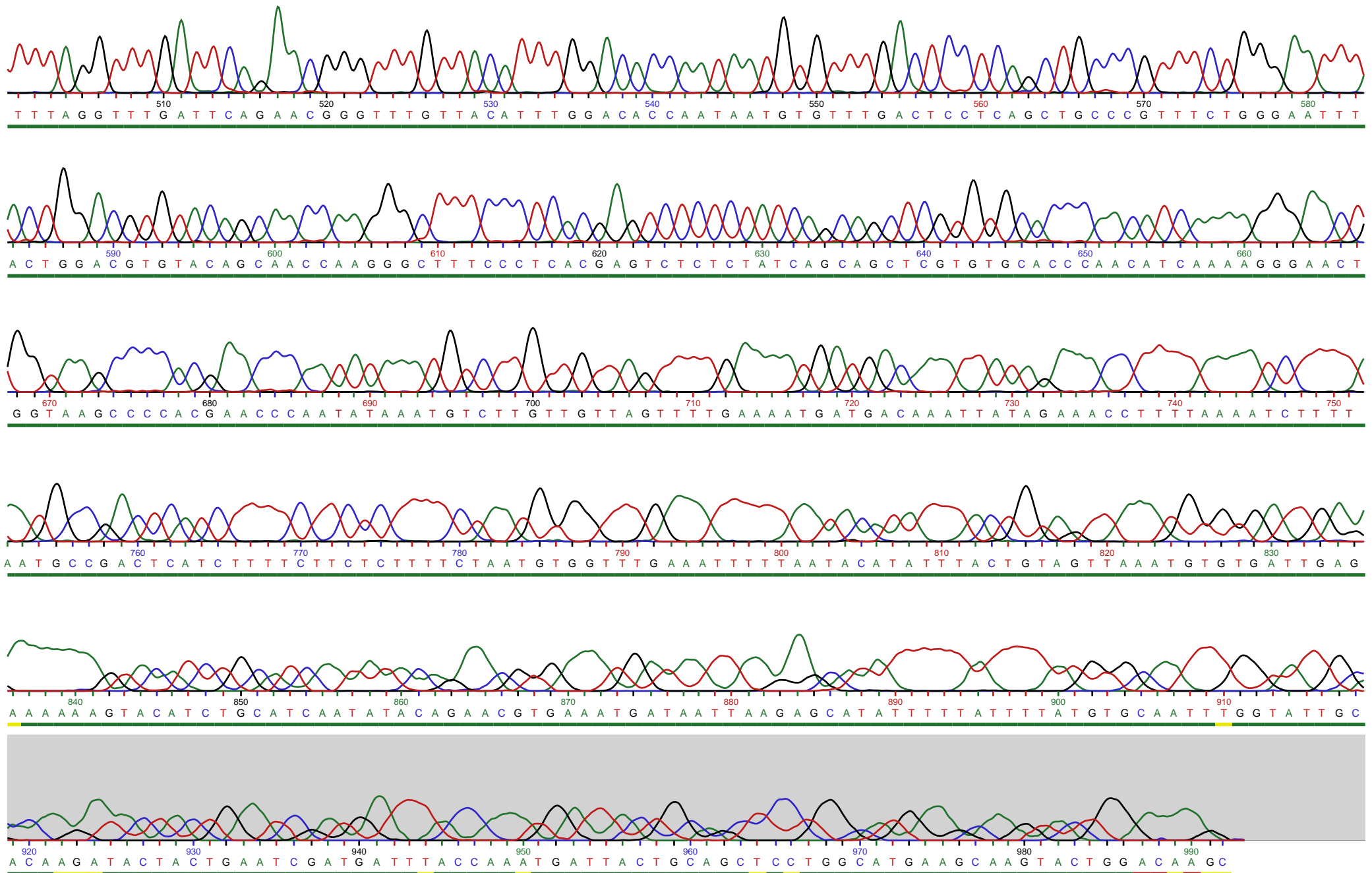

Supplement: Figure 1—figure supplement 3—source data 1. [file elife-81549-fig1-figsupp3-data1.zip › Figure_1_figure_supplement_3_source_data/Figure_1_figure_supplement_3_panel_ABC_source_data/Originals_F1_sequencing/Fish_18/mitfa/mitfa #18d_M13uni-21.pdf]

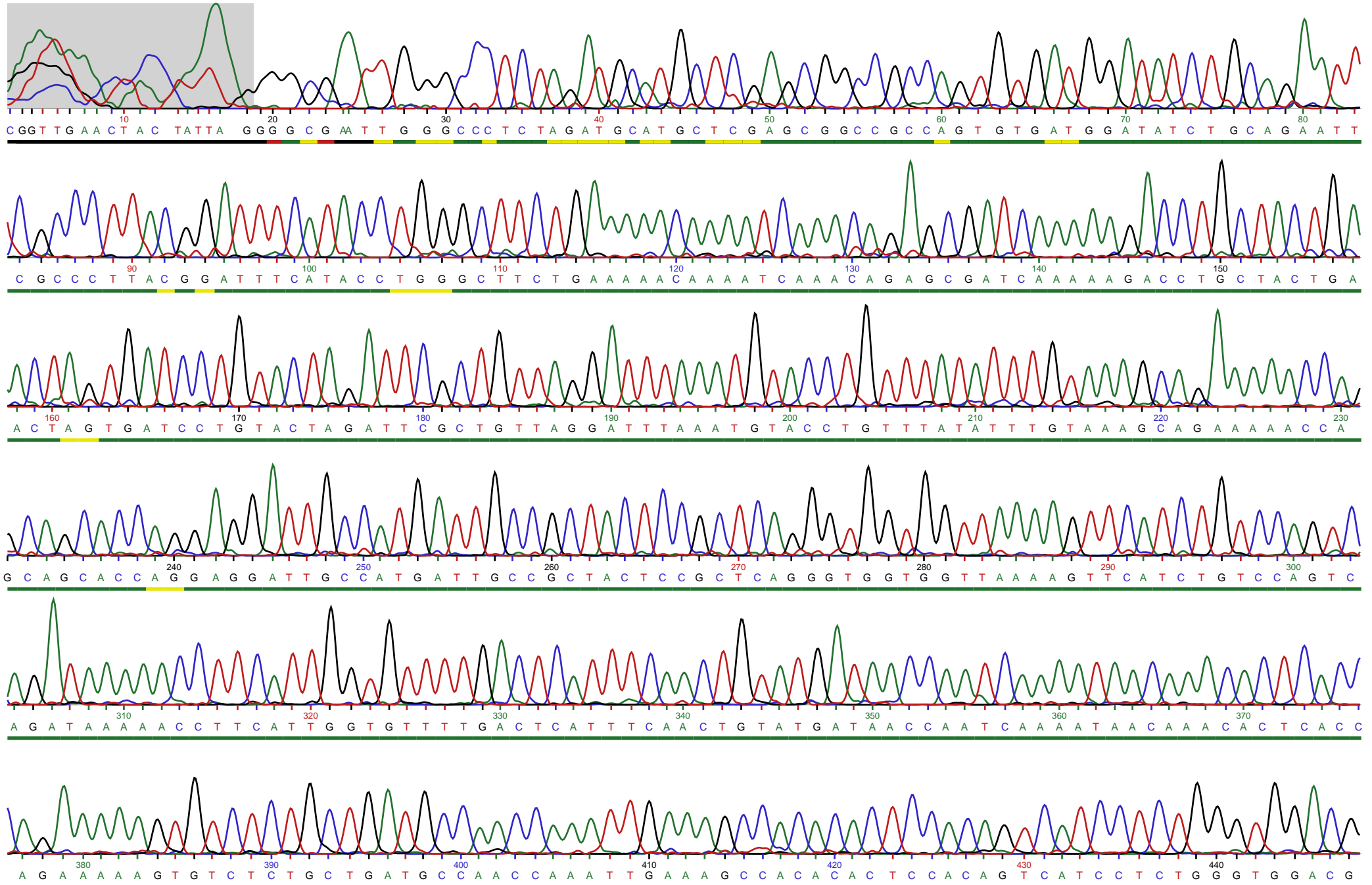

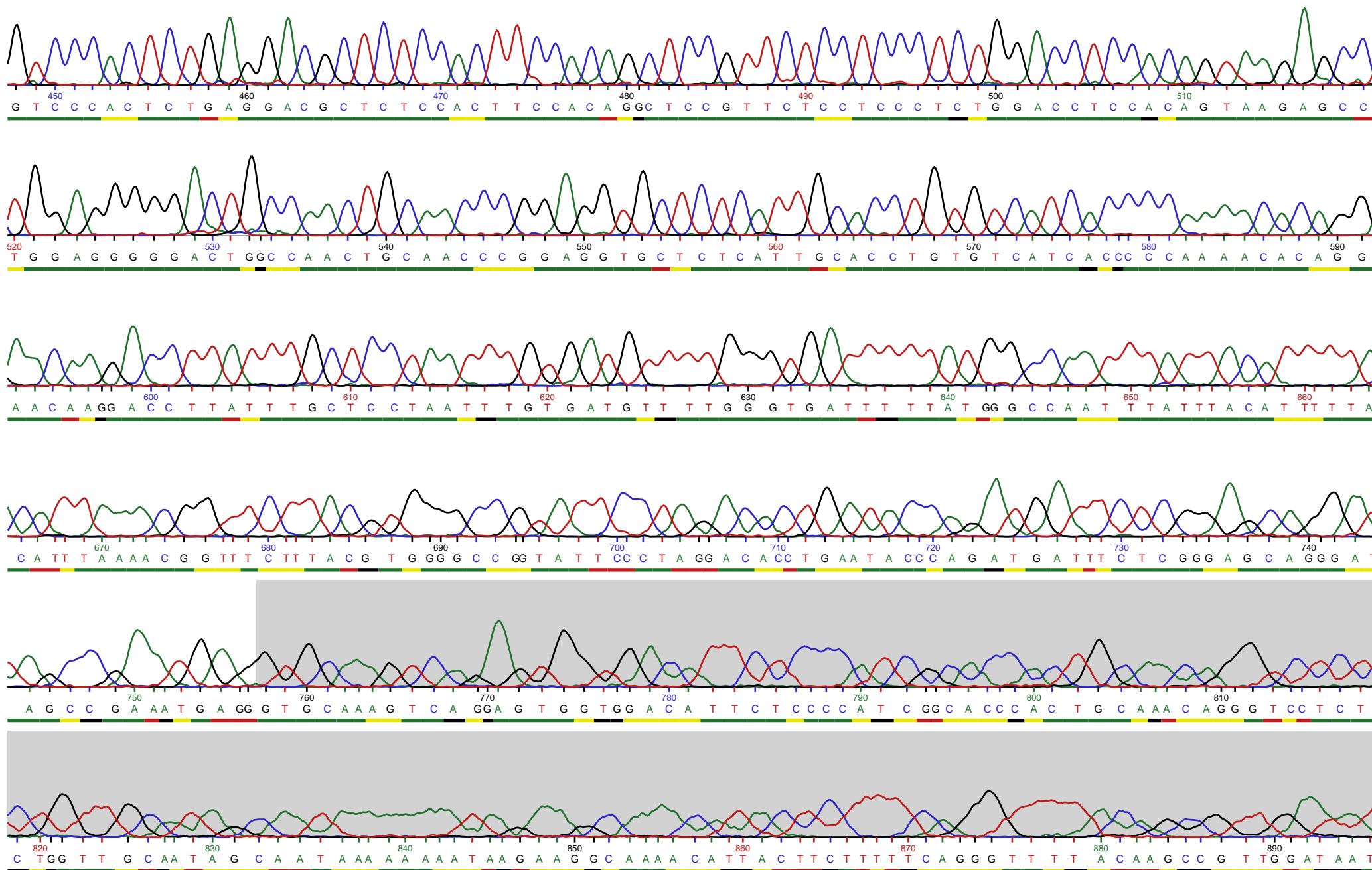

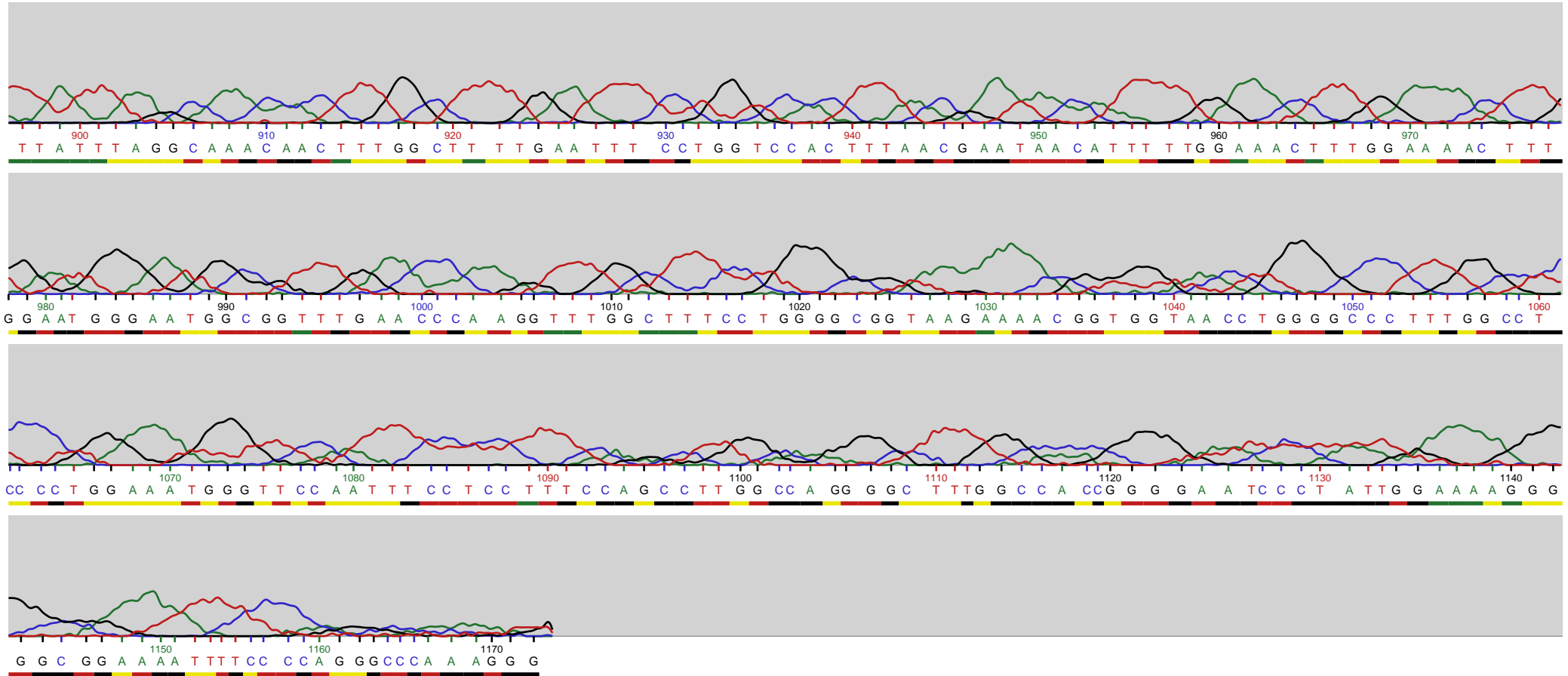

Supplement: Figure 1—figure supplement 3—source data 1. [file elife-81549-fig1-figsupp3-data1.zip › Figure_1_figure_supplement_3_source_data/Figure_1_figure_supplement_3_panel_ABC_source_data/Originals_F1_sequencing/Fish_19/csf1ra/csf #19g_M13uni-21.pdf]

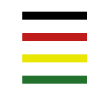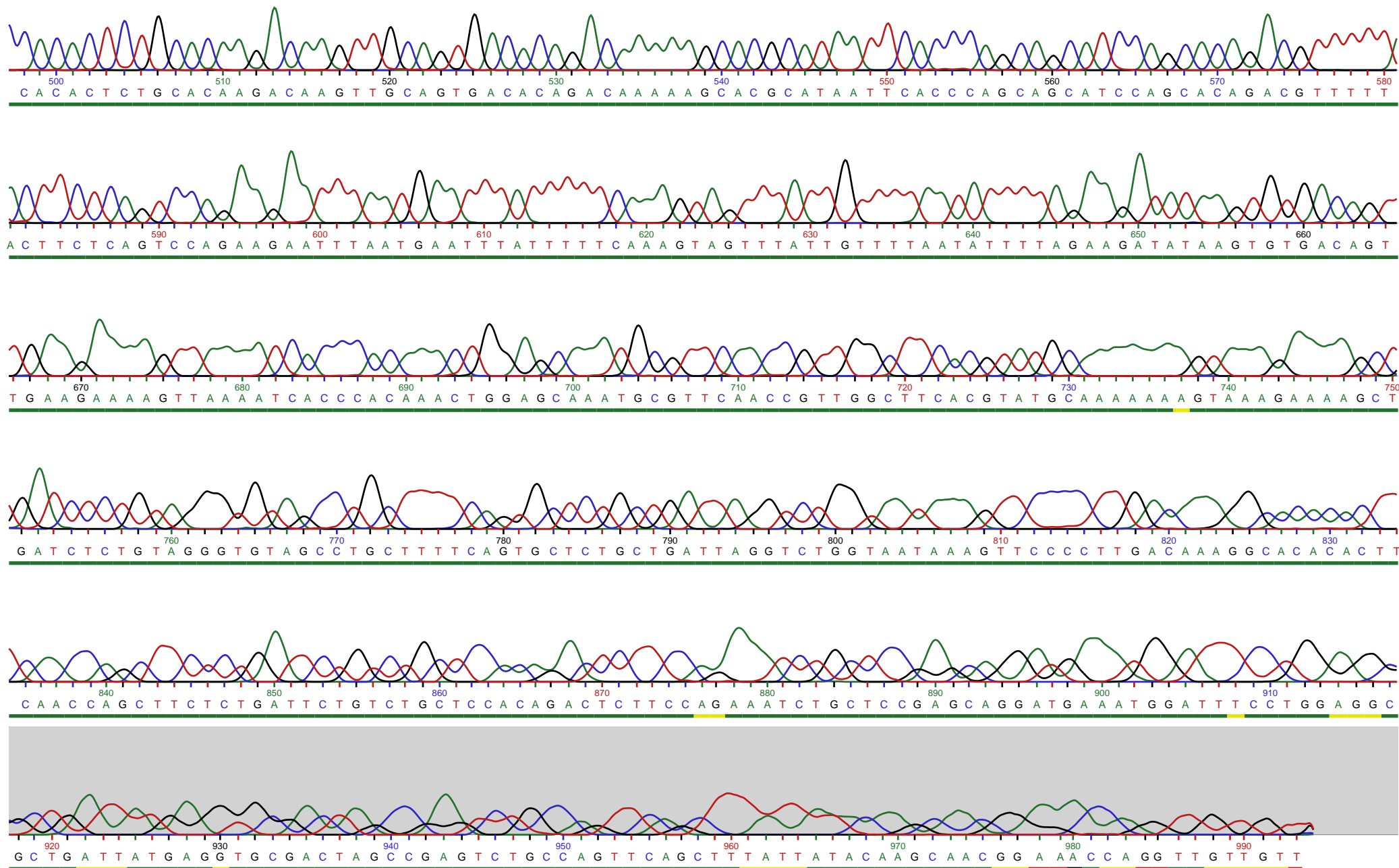

Supplement: Figure 1—figure supplement 3—source data 1. [file elife-81549-fig1-figsupp3-data1.zip › Figure_1_figure_supplement_3_source_data/Figure_1_figure_supplement_3_panel_ABC_source_data/Originals_F1_sequencing/Fish_19/ltk/ltk #19b_M13uni-21.pdf]

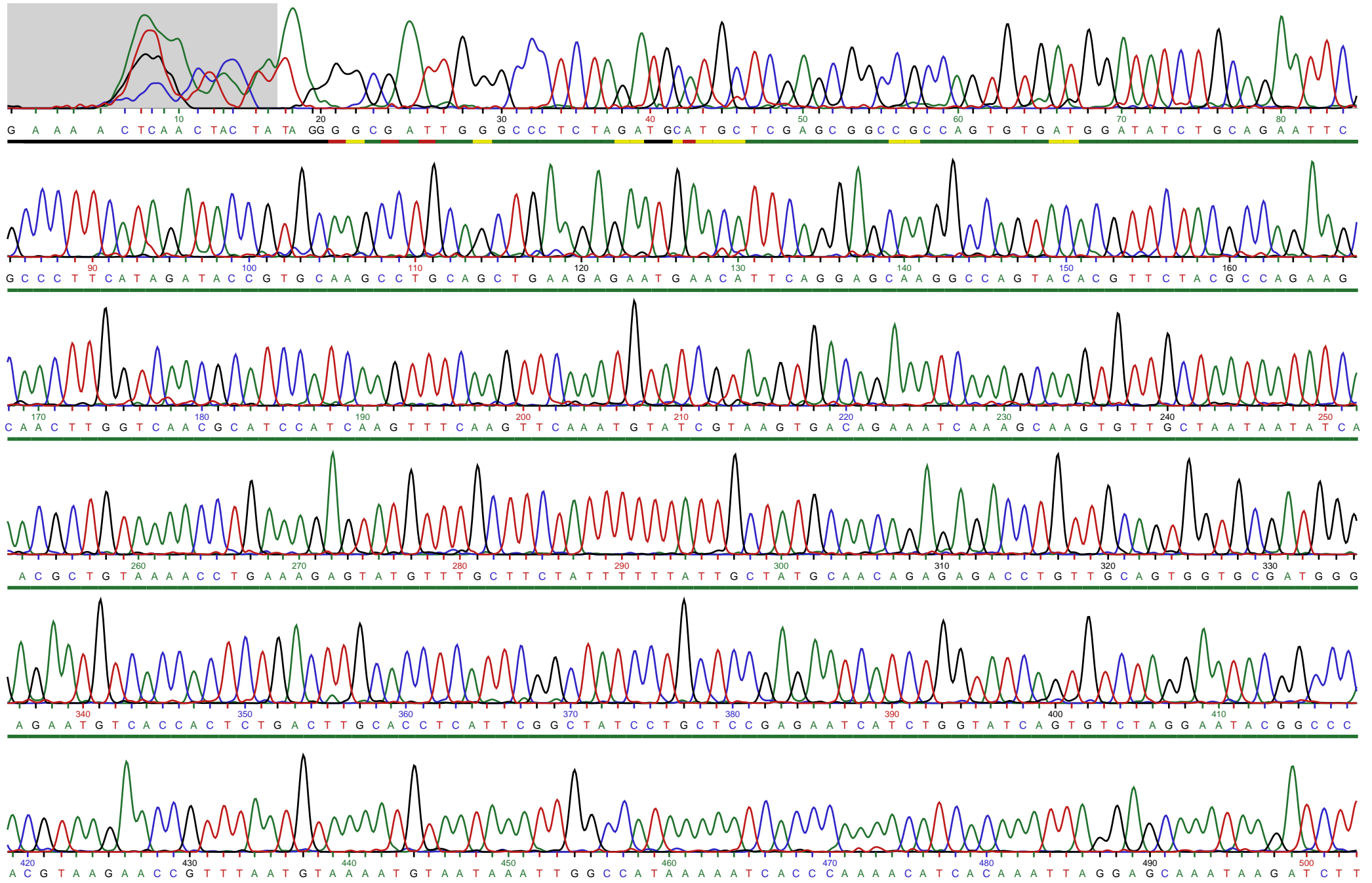

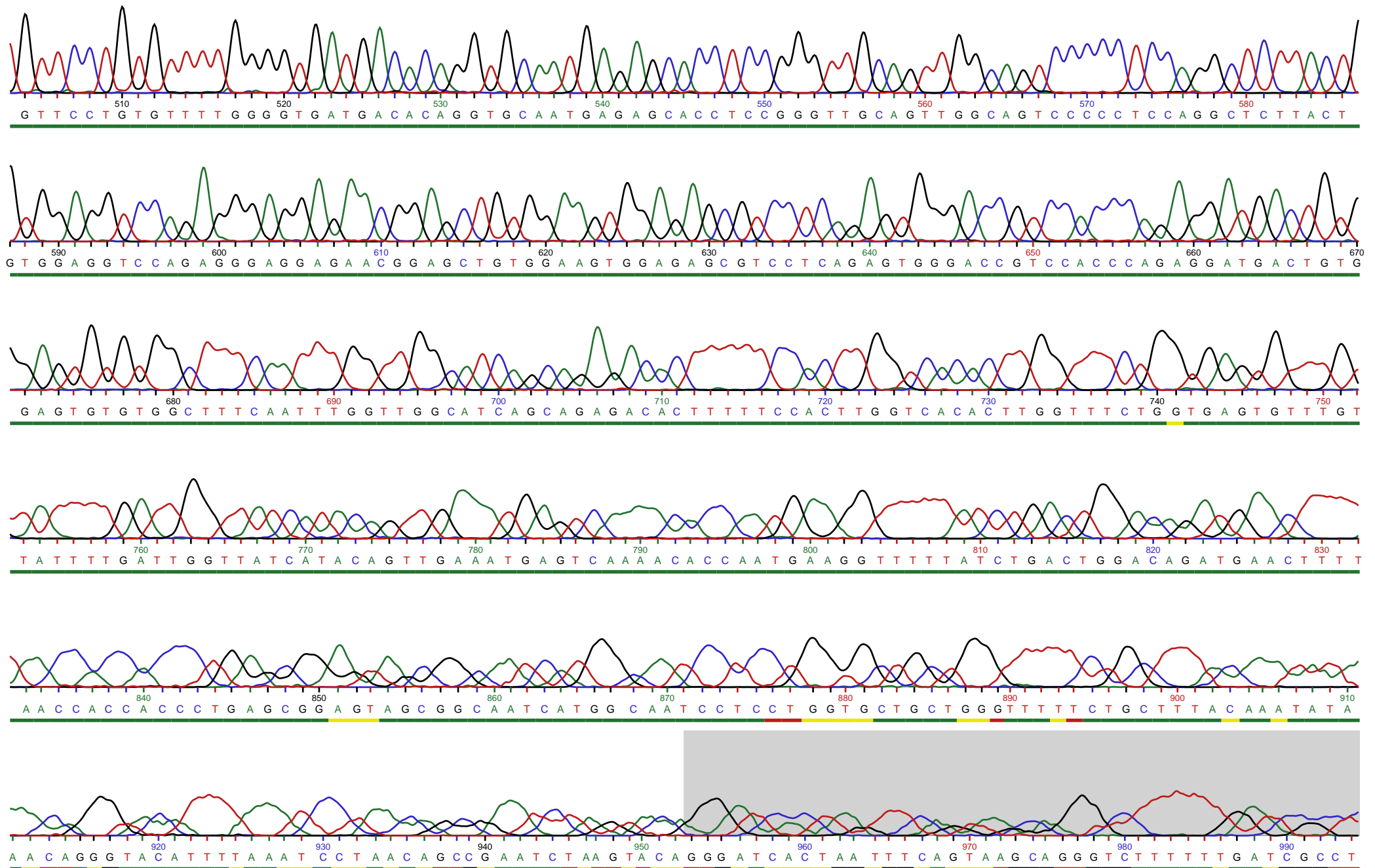

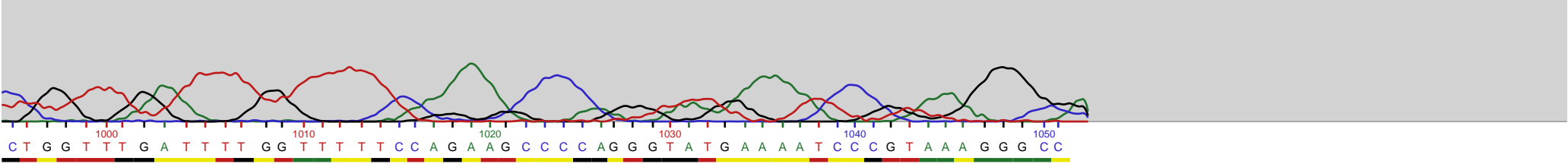

Supplement: Figure 1—figure supplement 3—source data 1. [file elife-81549-fig1-figsupp3-data1.zip › Figure_1_figure_supplement_3_source_data/Figure_1_figure_supplement_3_panel_ABC_source_data/Originals_F1_sequencing/Fish_29/csf1ra/csf #29c_M13uni-21.pdf]

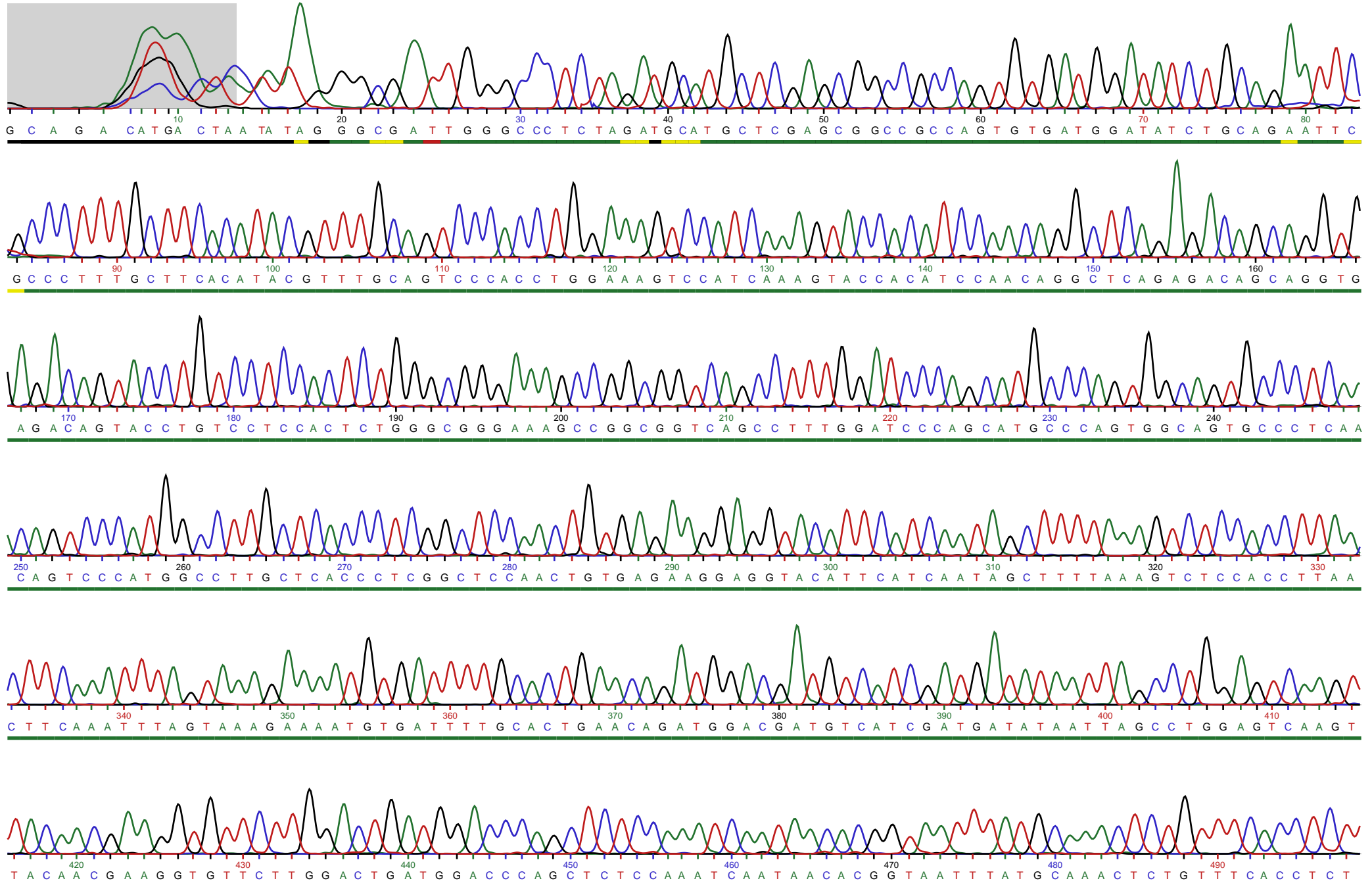

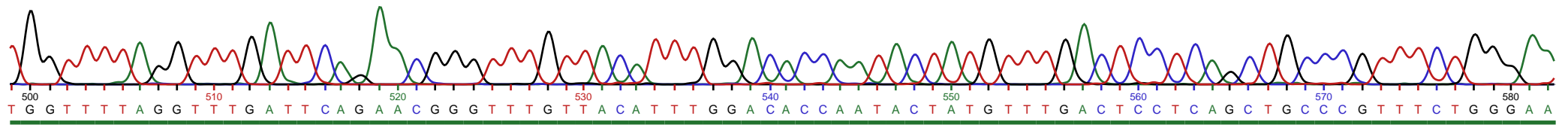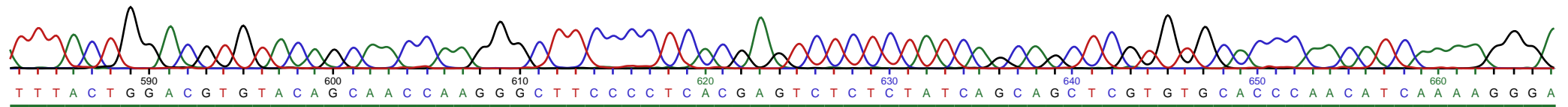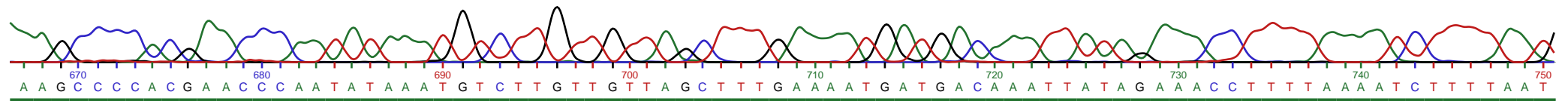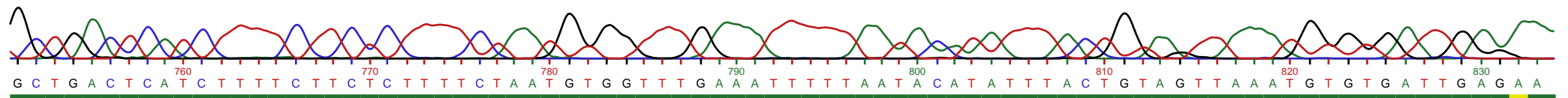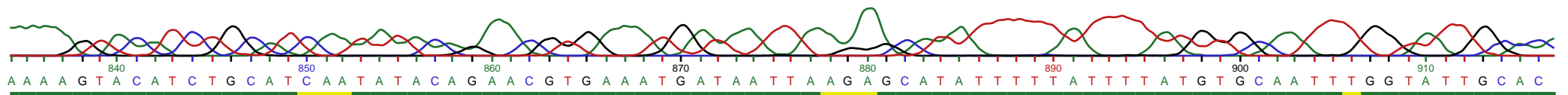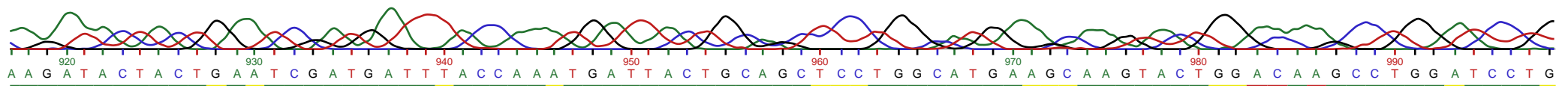

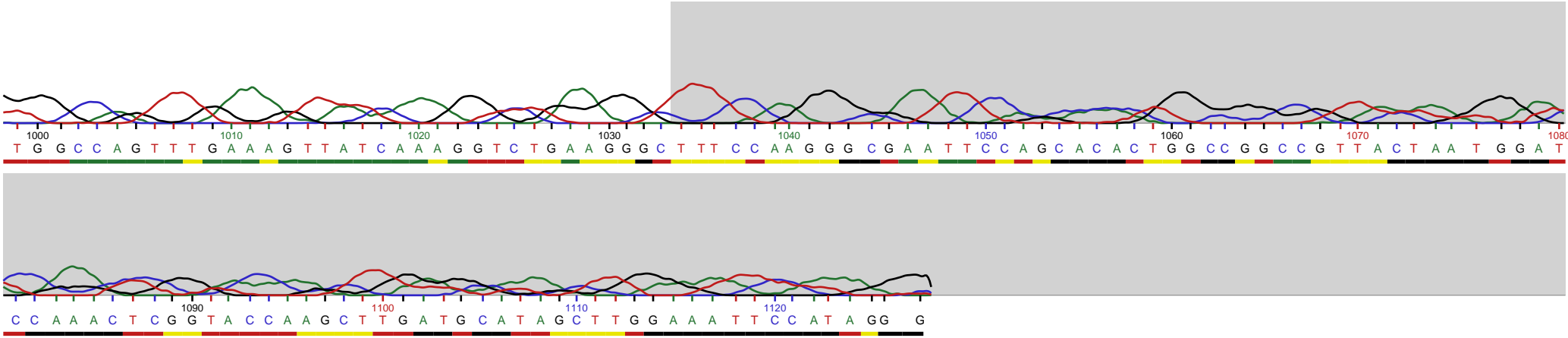

Supplement: Figure 1—figure supplement 3—source data 1. [file elife-81549-fig1-figsupp3-data1.zip › Figure_1_figure_supplement_3_source_data/Figure_1_figure_supplement_3_panel_ABC_source_data/Originals_F1_sequencing/Fish_29/mitfa/mitfa #29a_M13uni-21.pdf]

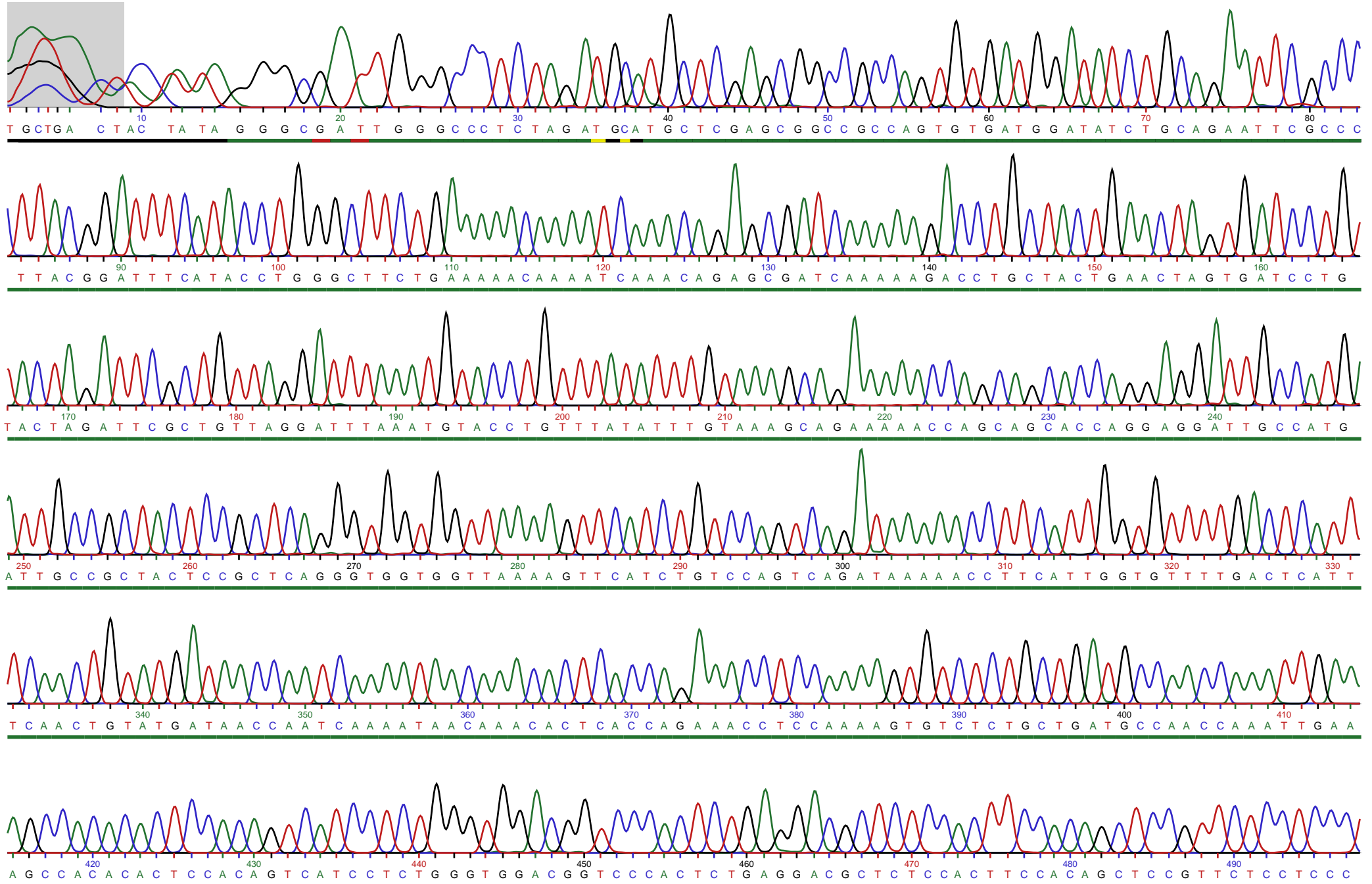

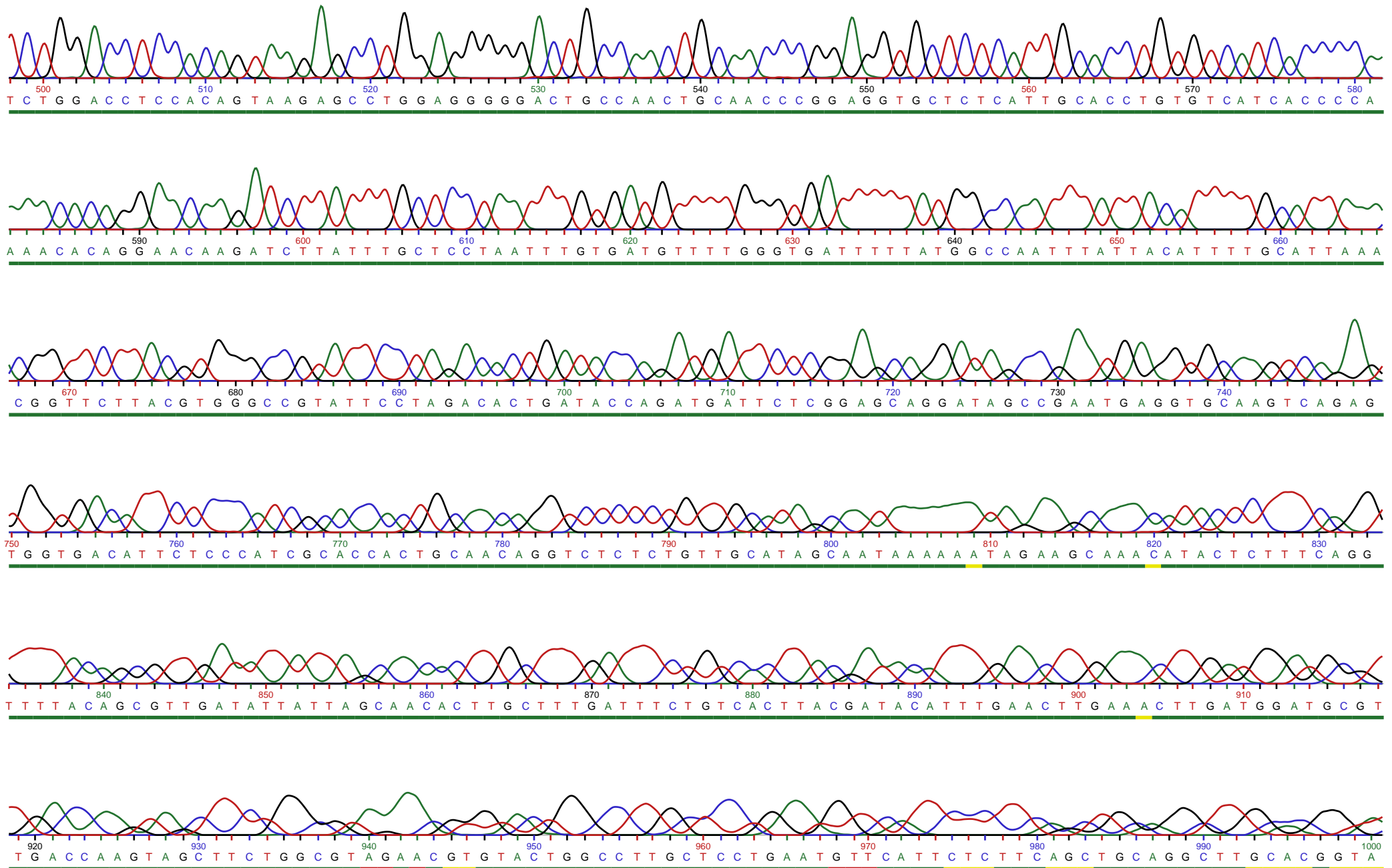

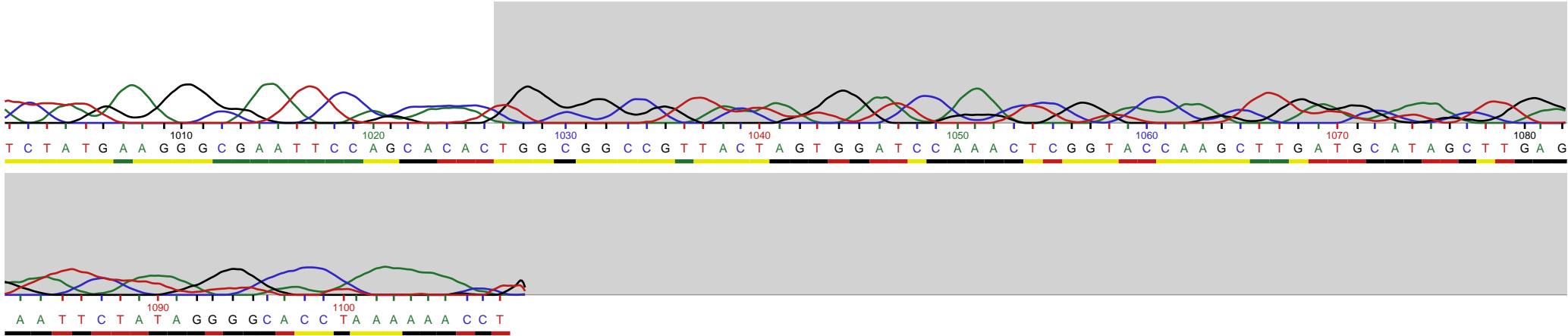

Supplement: Figure 1—figure supplement 3—source data 1. [file elife-81549-fig1-figsupp3-data1.zip › Figure_1_figure_supplement_3_source_data/Figure_1_figure_supplement_3_panel_ABC_source_data/Originals_F1_sequencing/Fish_6/csf1ra/csf #6a_M13uni-21.pdf]

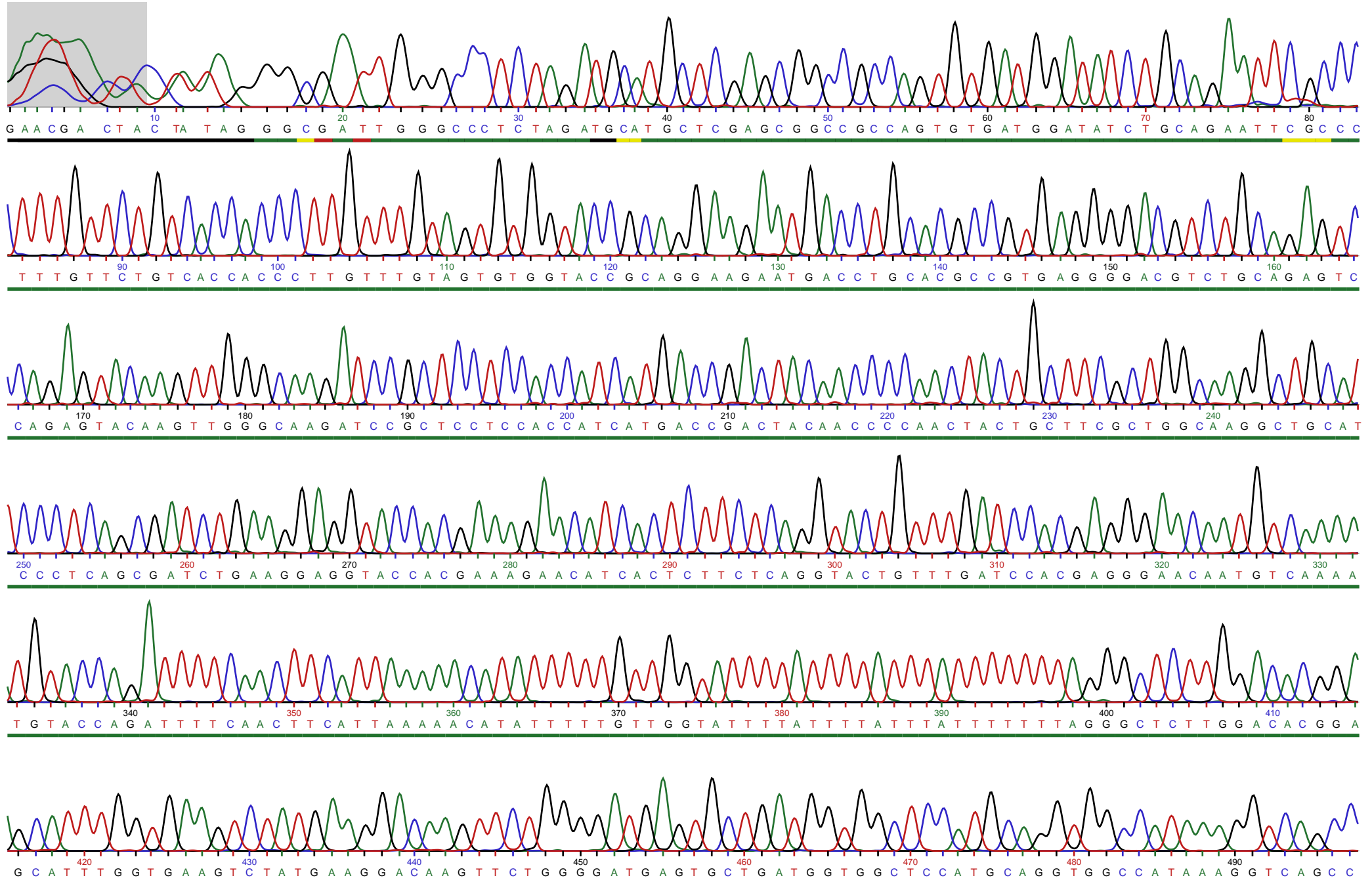

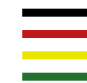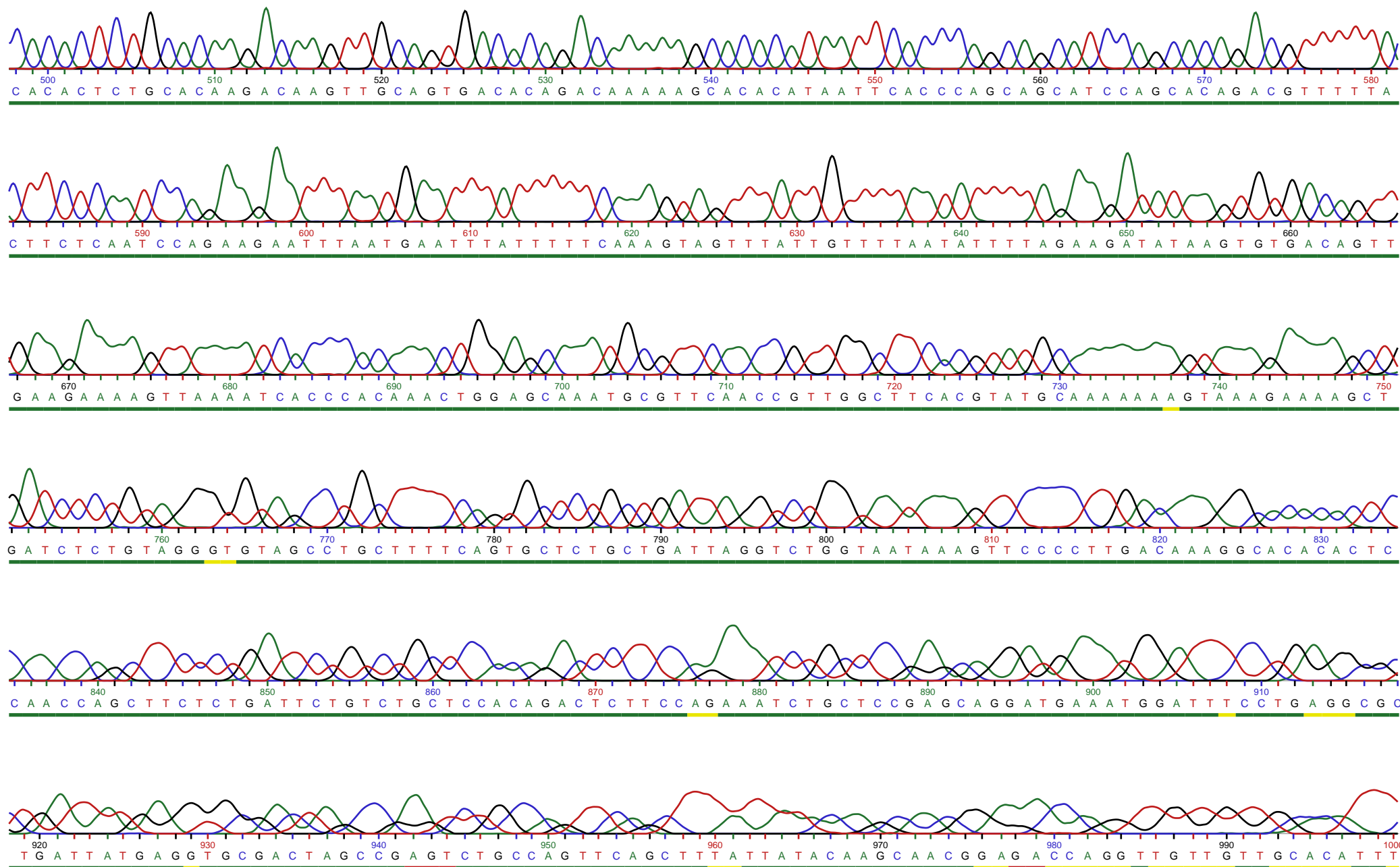

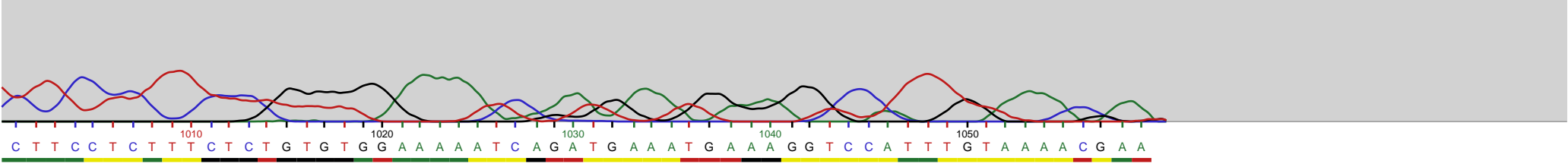

Supplement: Figure 1—figure supplement 3—source data 1. [file elife-81549-fig1-figsupp3-data1.zip › Figure_1_figure_supplement_3_source_data/Figure_1_figure_supplement_3_panel_ABC_source_data/Originals_F1_sequencing/Fish_6/ltk/ltk #6a_M13uni-21.pdf]

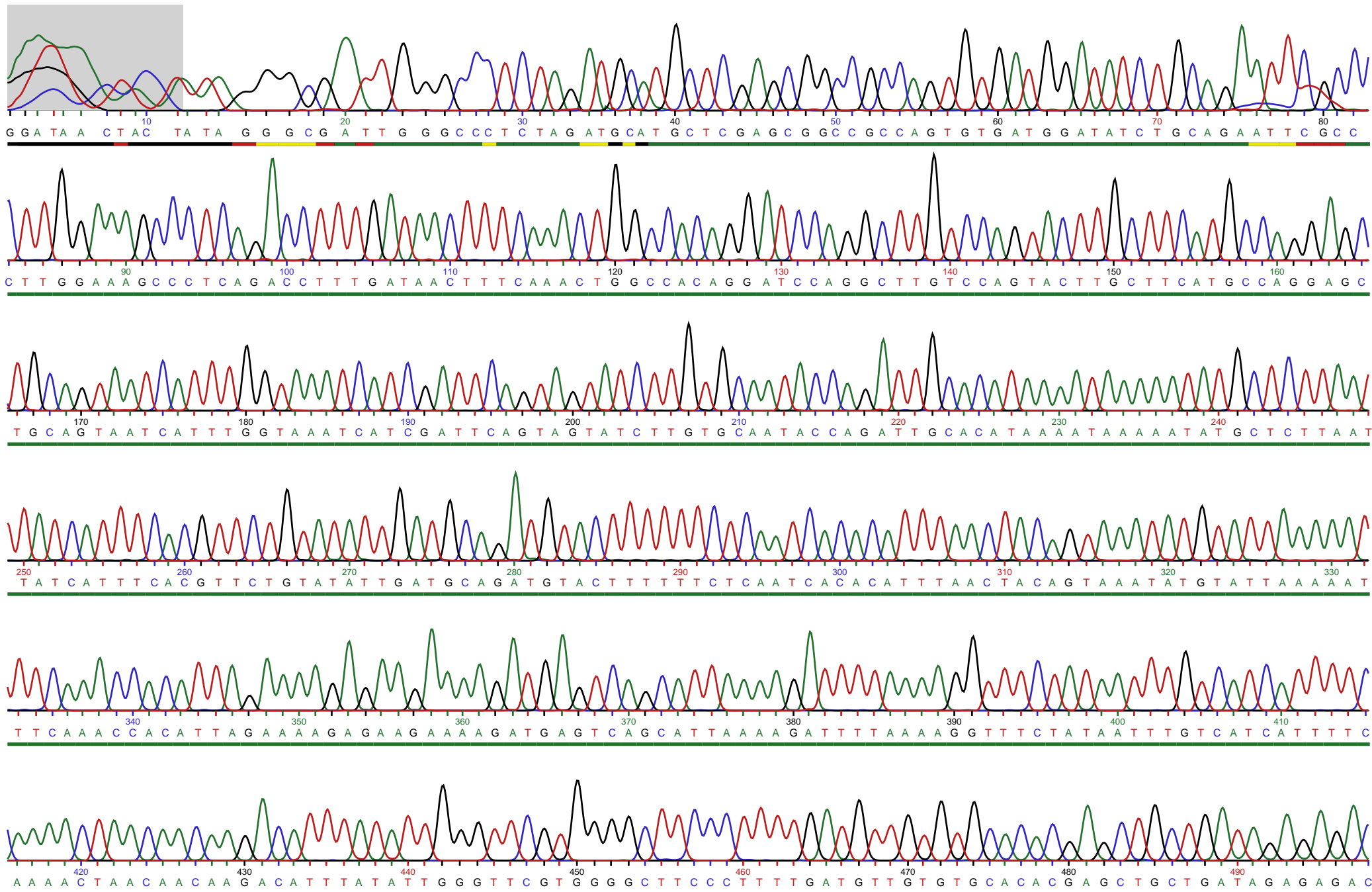

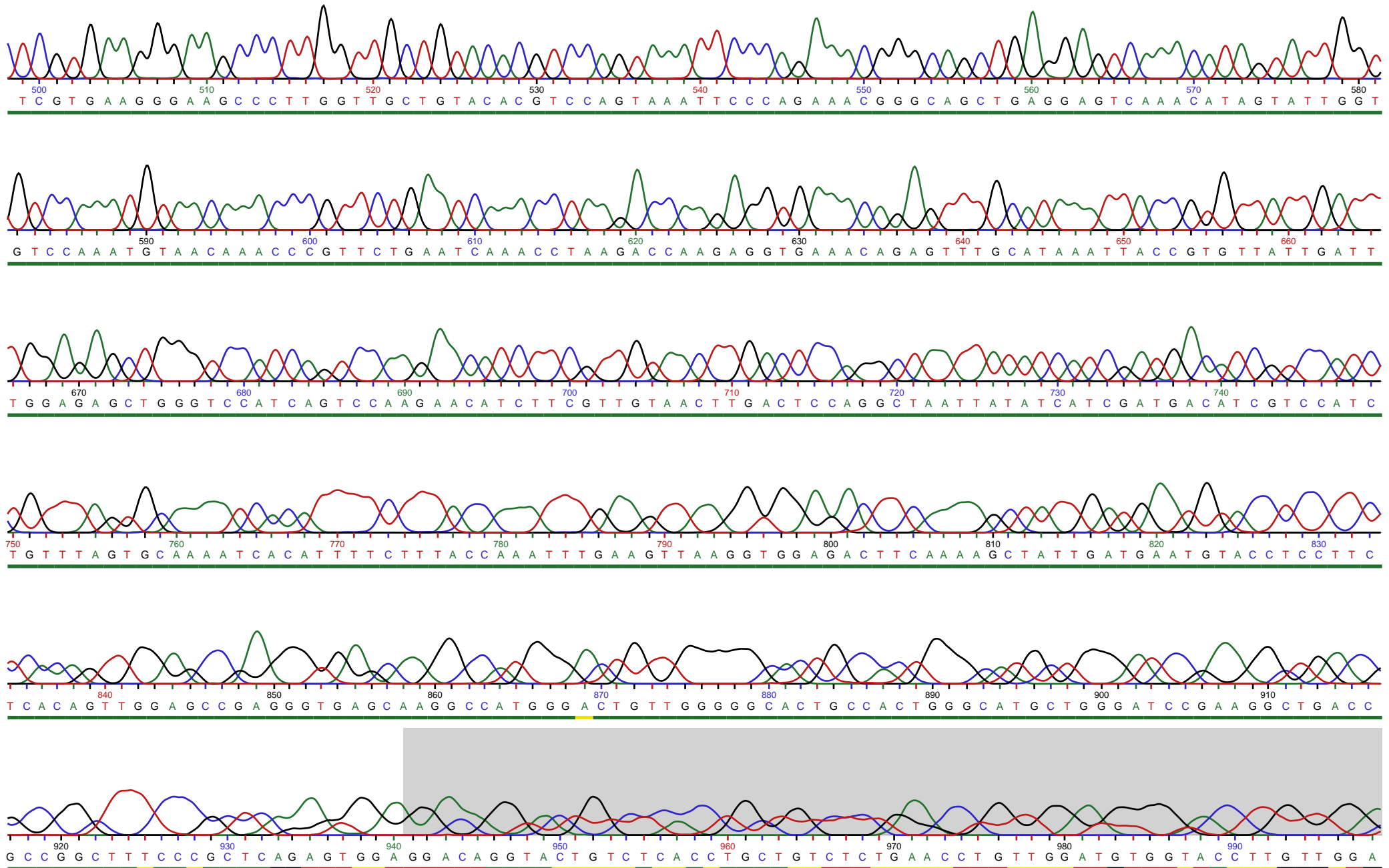

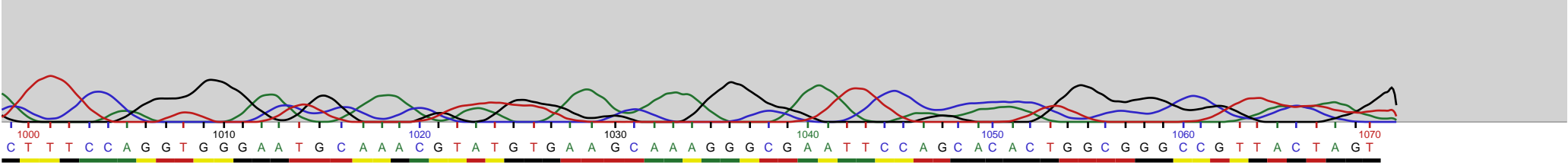

Supplement: Figure 1—figure supplement 3—source data 1. [file elife-81549-fig1-figsupp3-data1.zip › Figure_1_figure_supplement_3_source_data/Figure_1_figure_supplement_3_panel_ABC_source_data/Originals_F1_sequencing/Fish_6/mitfa/mitfa #6c_M13uni-21.pdf]

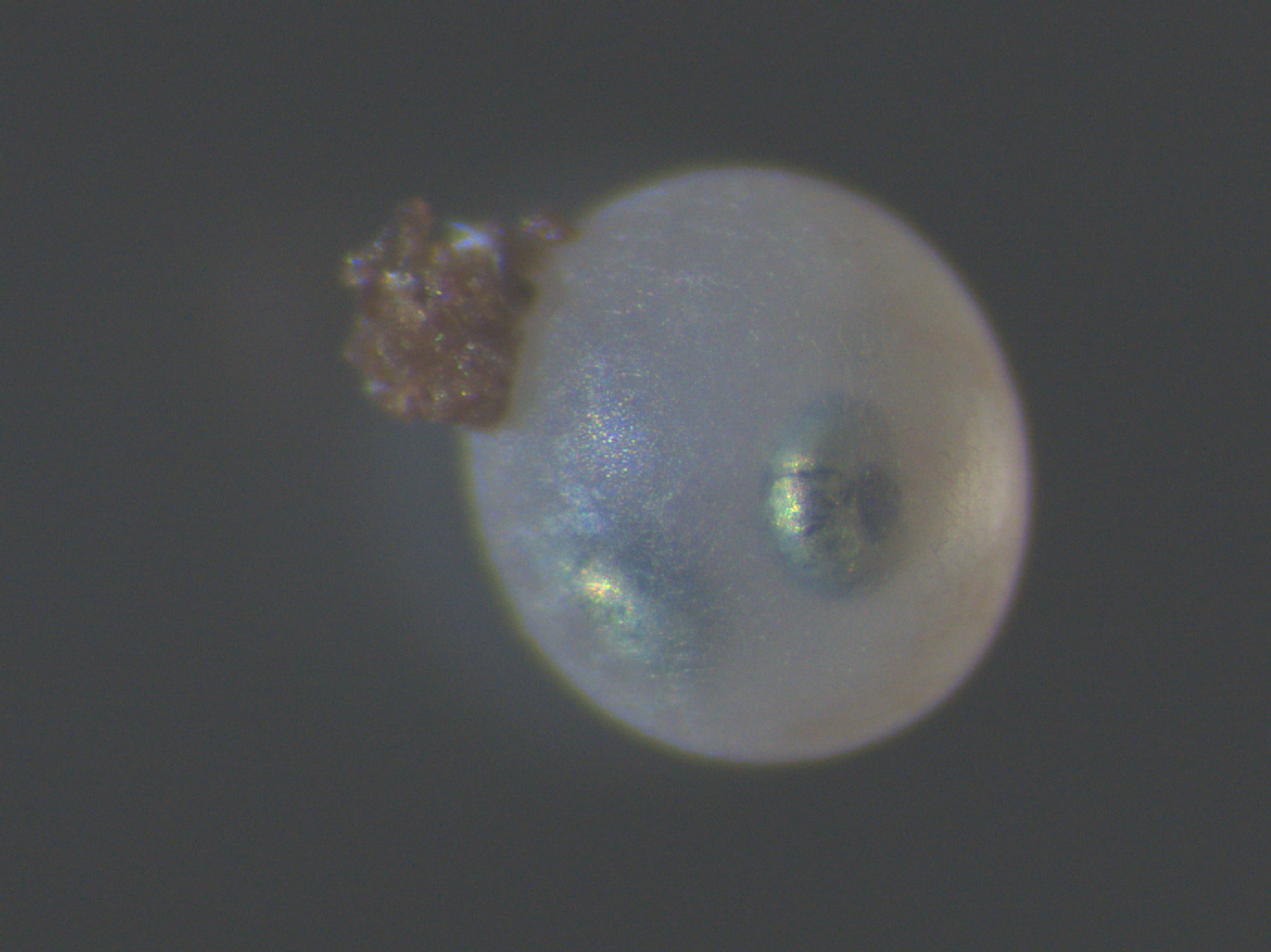

Supplement: Figure 1—figure supplement 3—source data 1. [file elife-81549-fig1-figsupp3-data1.zip › Figure_1_figure_supplement_3_source_data/Figure_1_figure_supplement_3_panel_D_source_data/F2_embryo_#1.3.tif]

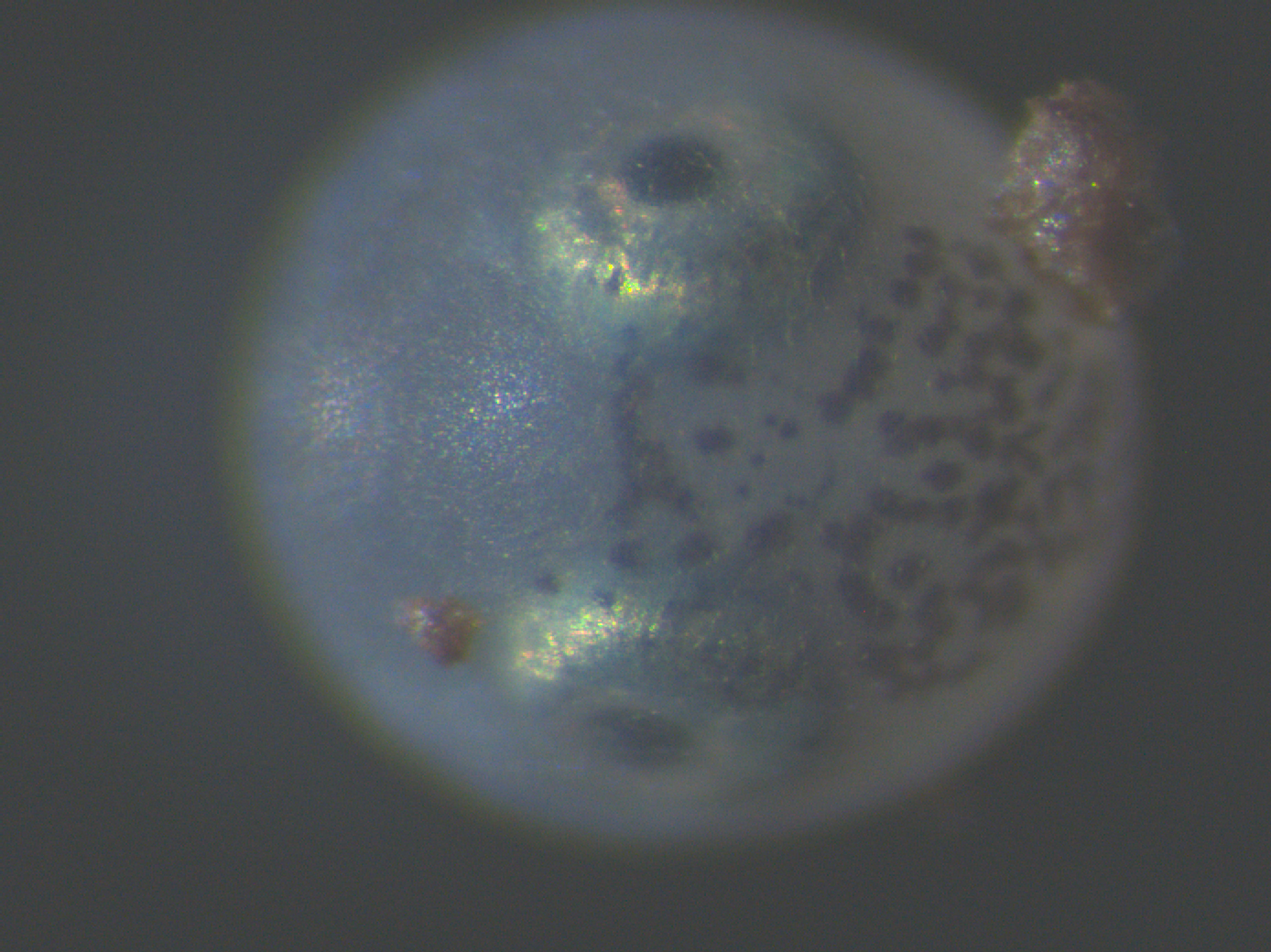

Supplement: Figure 1—figure supplement 3—source data 1. [file elife-81549-fig1-figsupp3-data1.zip › Figure_1_figure_supplement_3_source_data/Figure_1_figure_supplement_3_panel_D_source_data/F2_embryo_#2.5.tif]

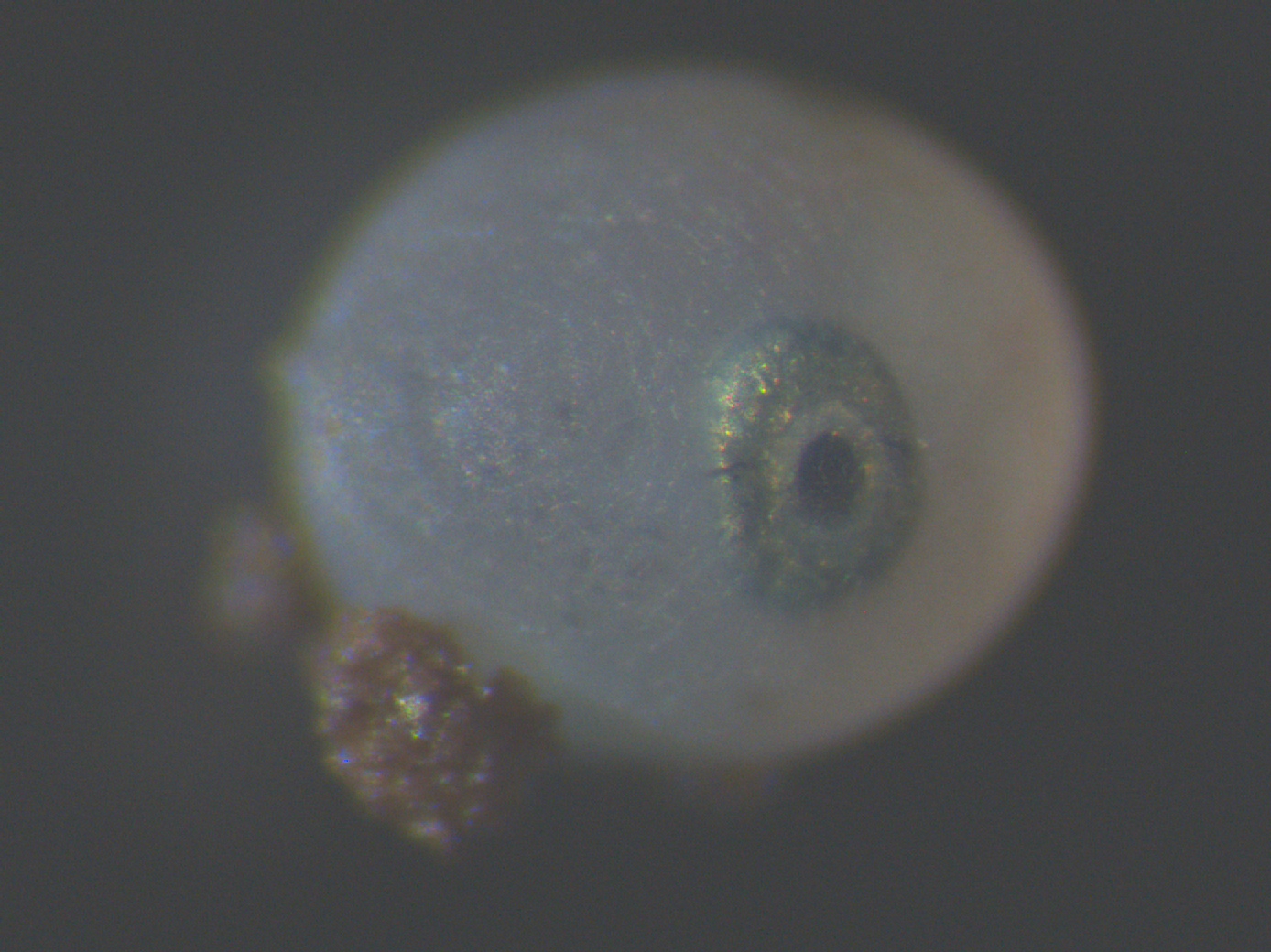

Supplement: Figure 1—figure supplement 3—source data 1. [file elife-81549-fig1-figsupp3-data1.zip › Figure_1_figure_supplement_3_source_data/Figure_1_figure_supplement_3_panel_D_source_data/F2_embryo_#3.2.tif]

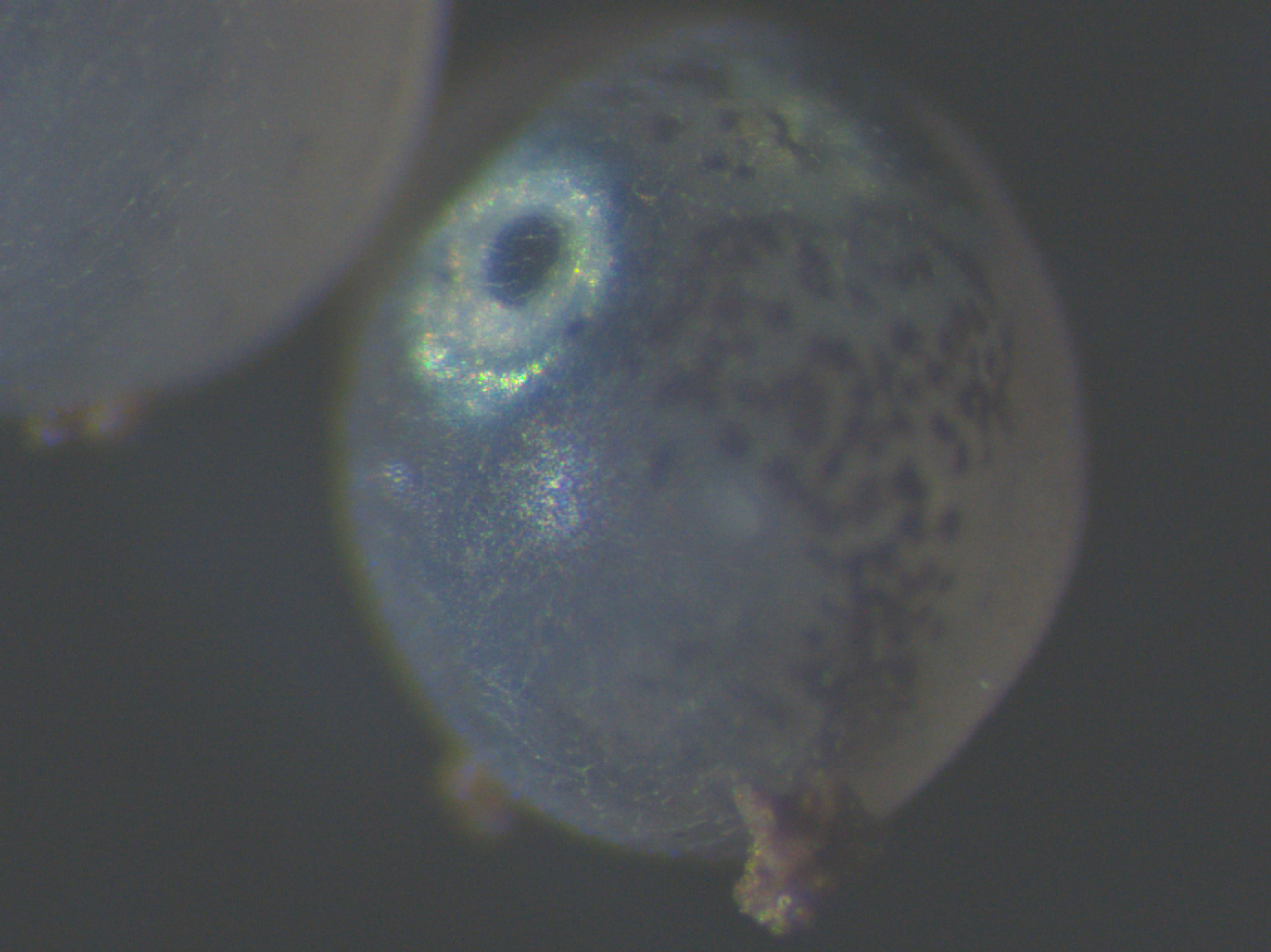

Supplement: Figure 1—figure supplement 3—source data 1. [file elife-81549-fig1-figsupp3-data1.zip › Figure_1_figure_supplement_3_source_data/Figure_1_figure_supplement_3_panel_D_source_data/F2_embryo_#4.2.tif]

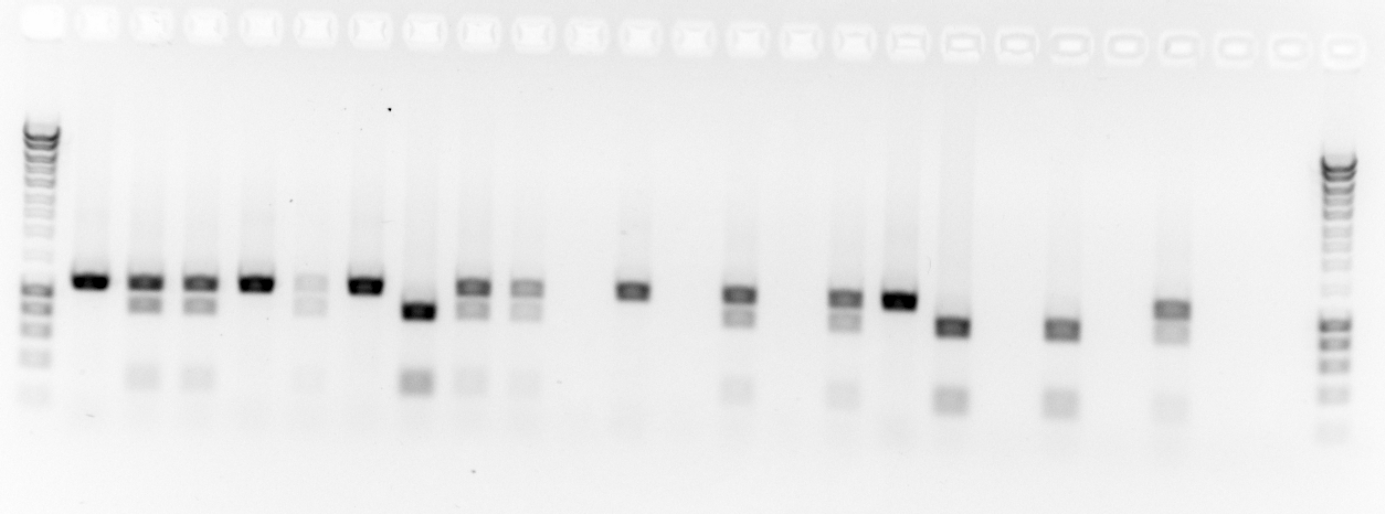

Supplement: Figure 1—figure supplement 3—source data 1. [file elife-81549-fig1-figsupp3-data1.zip › Figure_1_figure_supplement_3_source_data/Figure_1_figure_supplement_3_panel_EF_source_data/Figure_1_figure_supplement_1_panel_e(1)_source_data.tif]

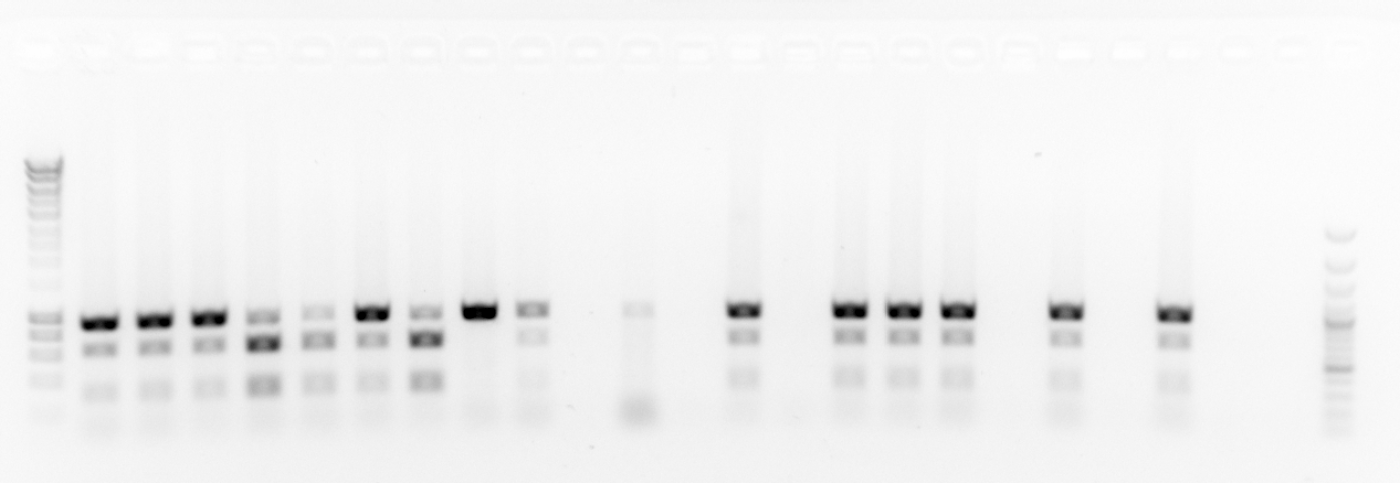

Supplement: Figure 1—figure supplement 3—source data 1. [file elife-81549-fig1-figsupp3-data1.zip › Figure_1_figure_supplement_3_source_data/Figure_1_figure_supplement_3_panel_EF_source_data/Figure_1_figure_supplement_1_panel_e(2)_source_data.tif]

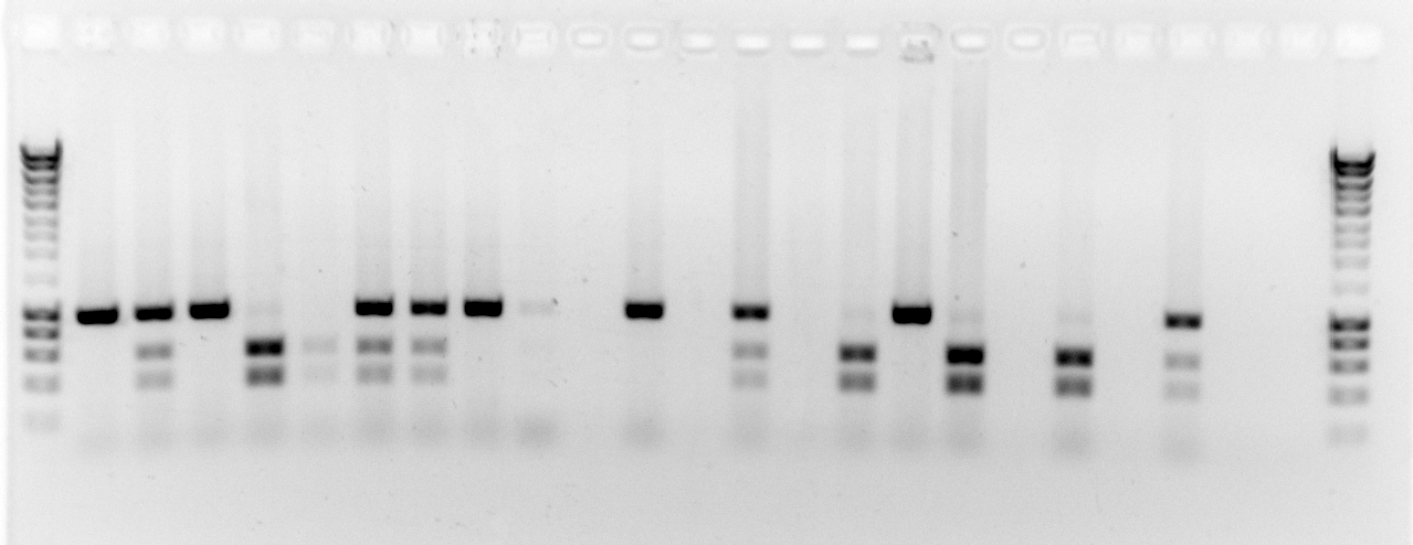

Supplement: Figure 1—figure supplement 3—source data 1. [file elife-81549-fig1-figsupp3-data1.zip › Figure_1_figure_supplement_3_source_data/Figure_1_figure_supplement_3_panel_EF_source_data/Figure_1_figure_supplement_1_panel_e(3)_source_data.tif]

## Figure 2

panel d

*csf1ra*

*rpl13a*

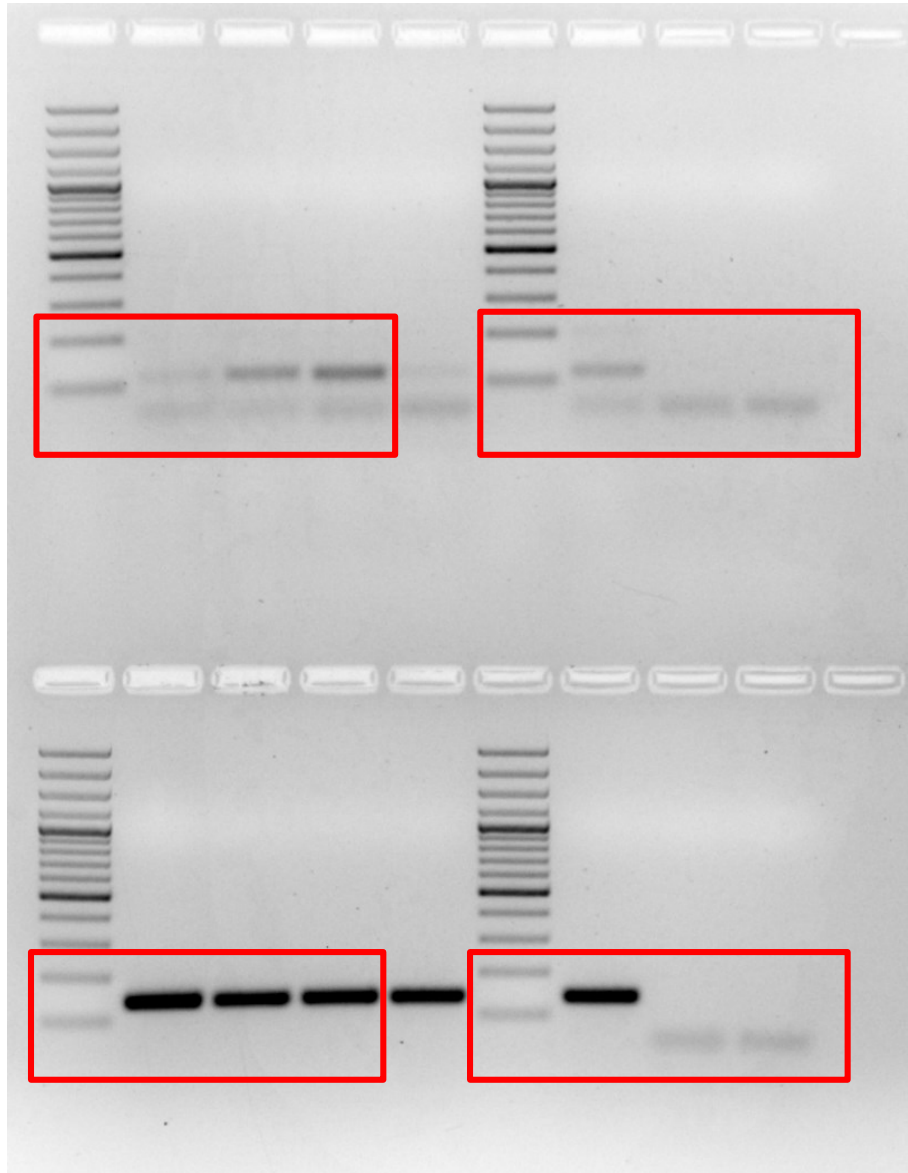

The red box surrounds the lanes shown in the respective panel.

Supplement: Figure 2—source data 1. [file elife-81549-fig2-data1.zip › Figure_2_source_data/Figure_2_panel_A_source_data/Figure_2_panel_a_source_data.pdf]

## Slide 1
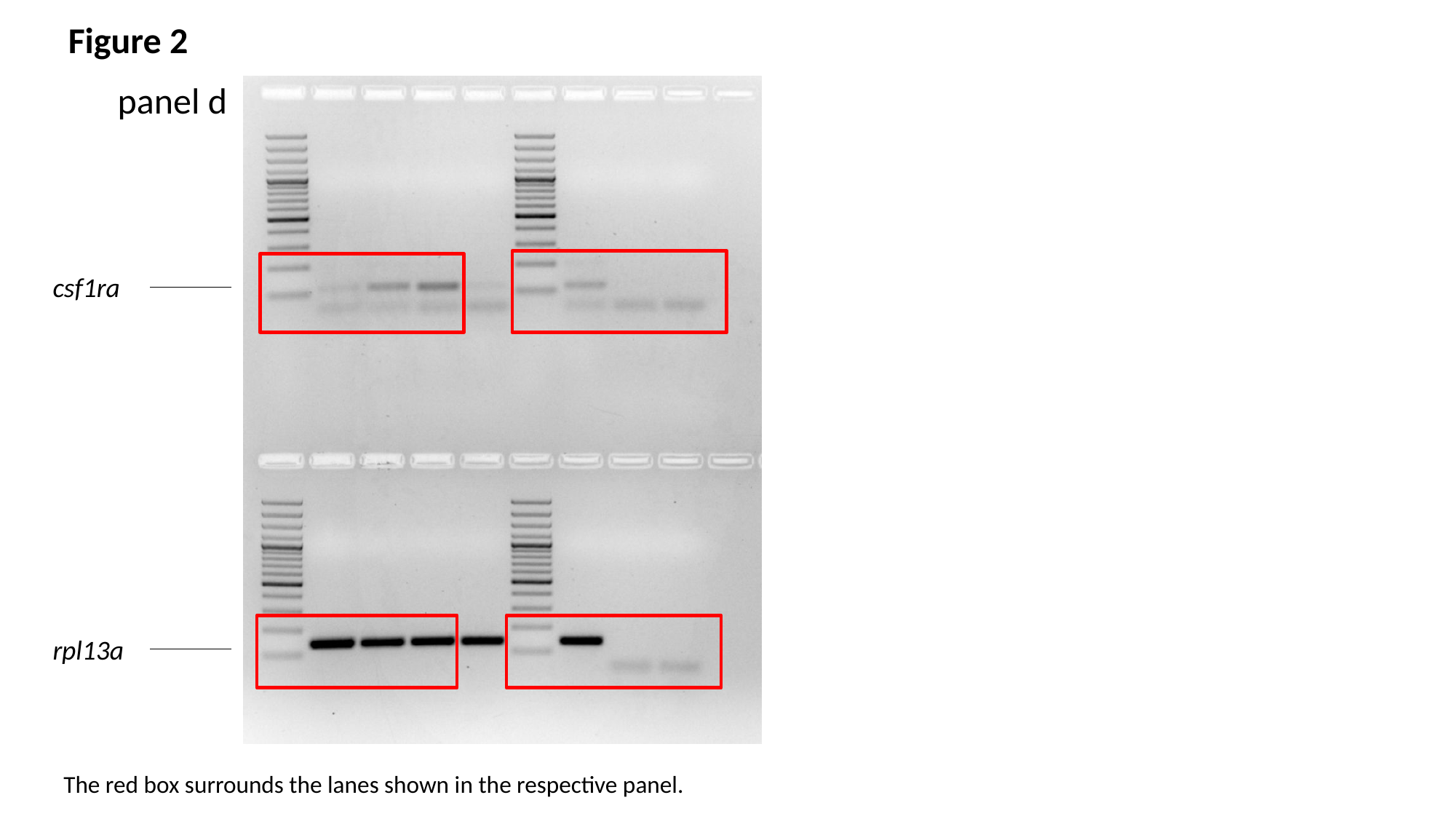

Figure 2
 panel d
csf1ra
rpl13a
The red box surrounds the lanes shown in the respective panel.

Supplement: Figure 2—source data 1. [file elife-81549-fig2-data1.zip › Figure_2_source_data/Figure_2_panel_A_source_data/Figure_2_panel_a_source_data.pptx]

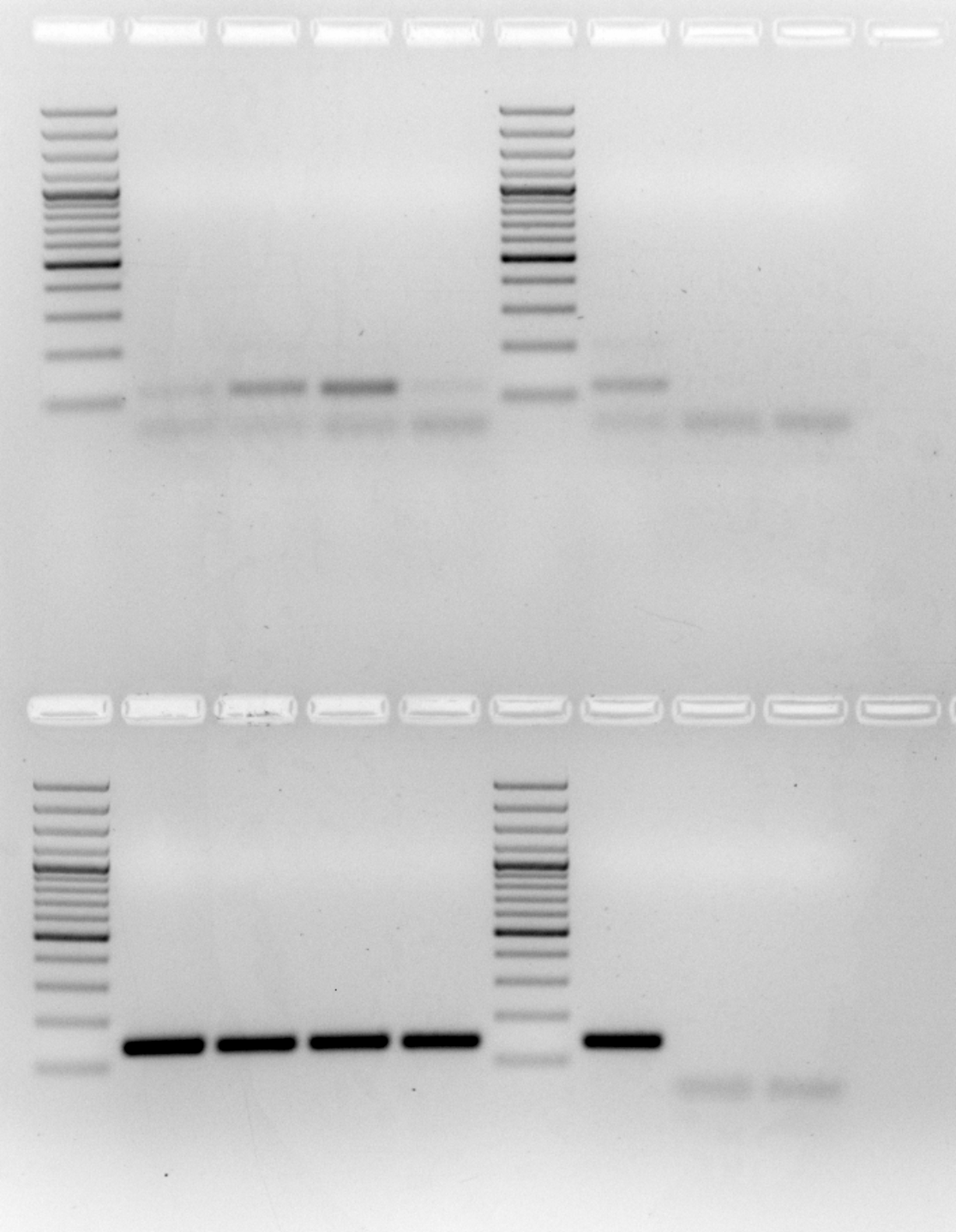

Supplement: Figure 2—source data 1. [file elife-81549-fig2-data1.zip › Figure_2_source_data/Figure_2_panel_A_source_data/Figure_2_panel_a_source_data.tif]

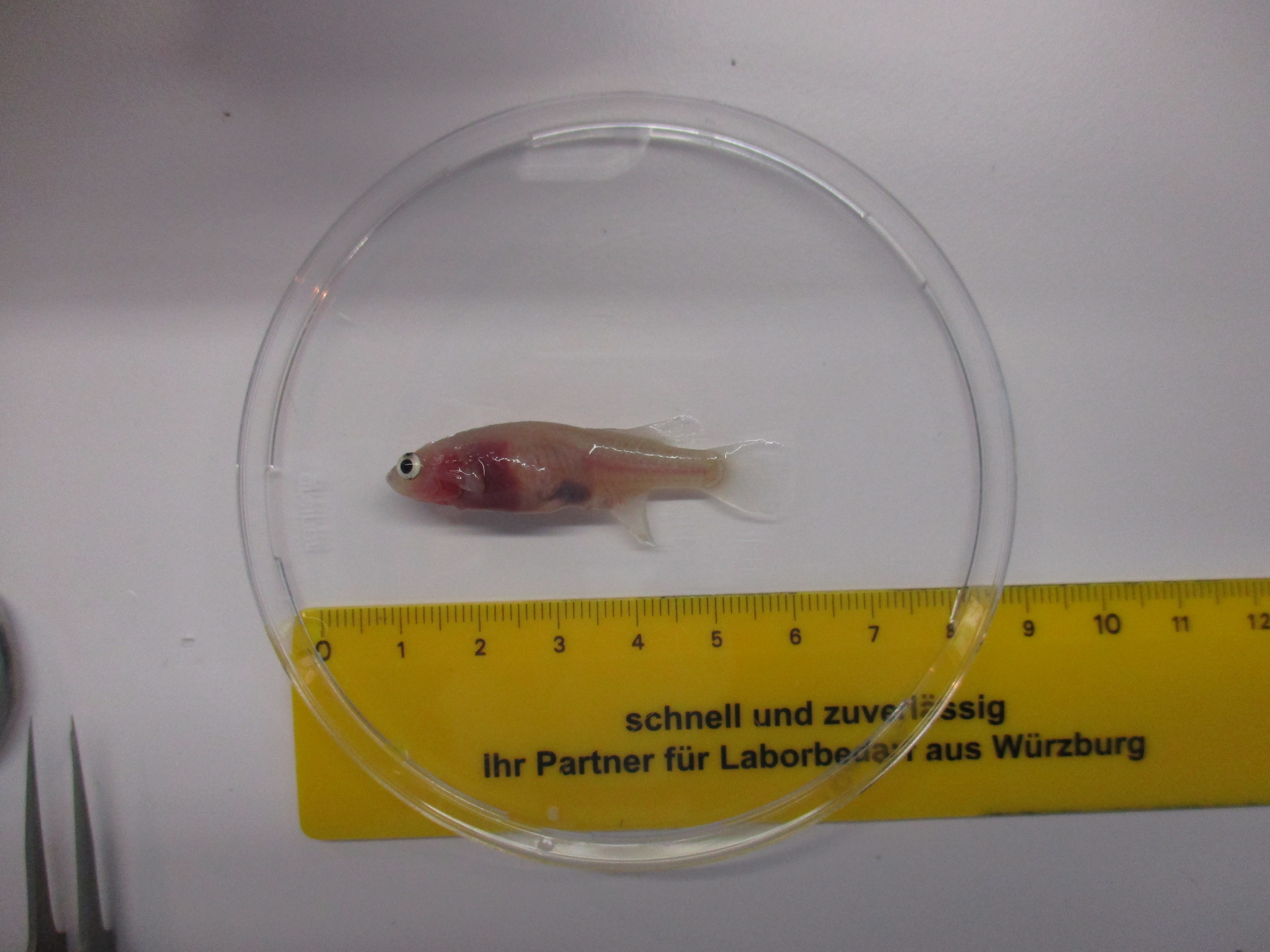

Supplement: Figure 2—source data 1. [file elife-81549-fig2-data1.zip › Figure_2_source_data/Figure_2_panel_CD_C ́D ́_source_data/female_239dph.JPG]

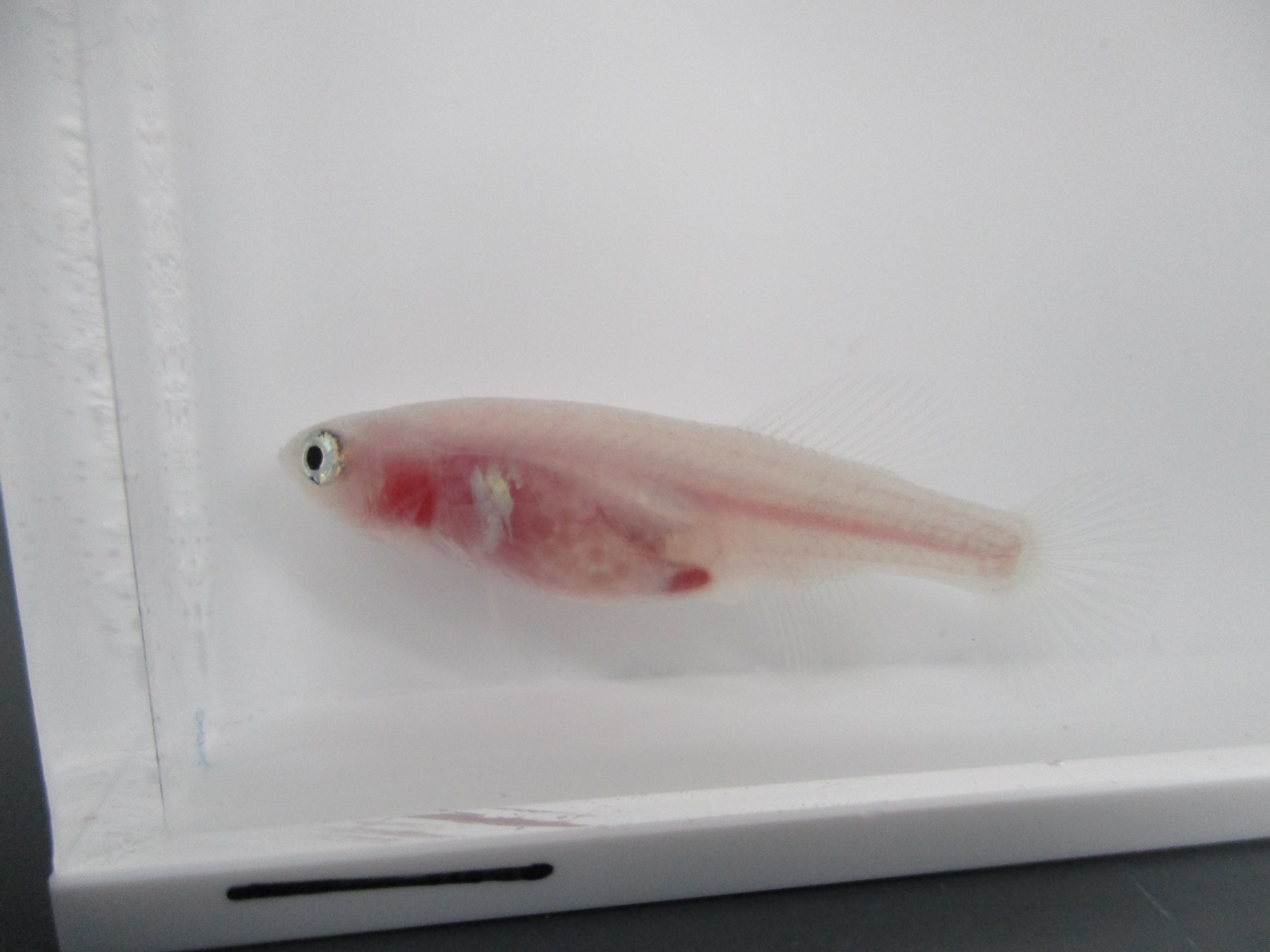

Supplement: Figure 2—source data 1. [file elife-81549-fig2-data1.zip › Figure_2_source_data/Figure_2_panel_CD_C ́D ́_source_data/female_42dph.JPG]

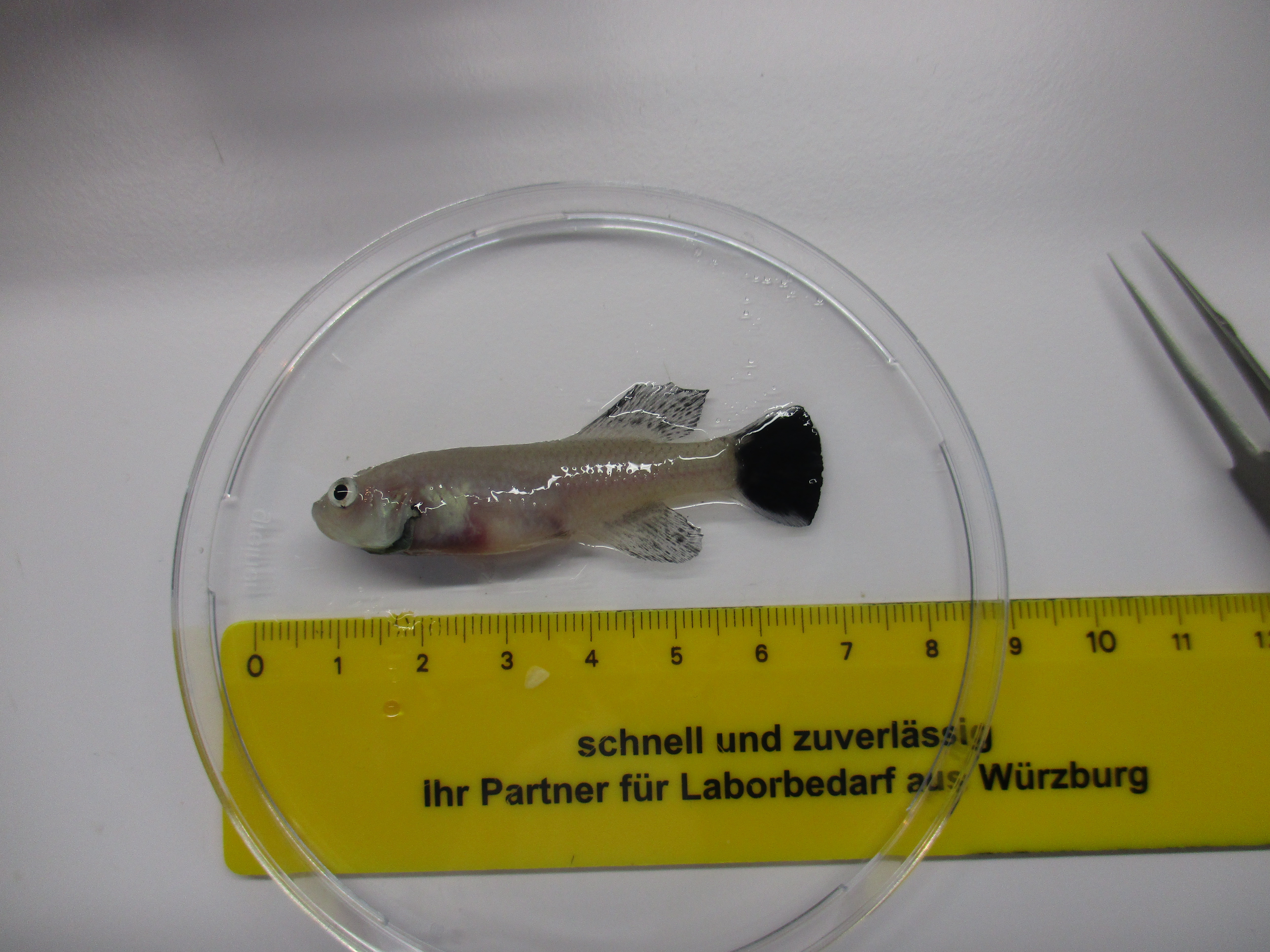

Supplement: Figure 2—source data 1. [file elife-81549-fig2-data1.zip › Figure_2_source_data/Figure_2_panel_CD_C ́D ́_source_data/male_239dph.JPG]

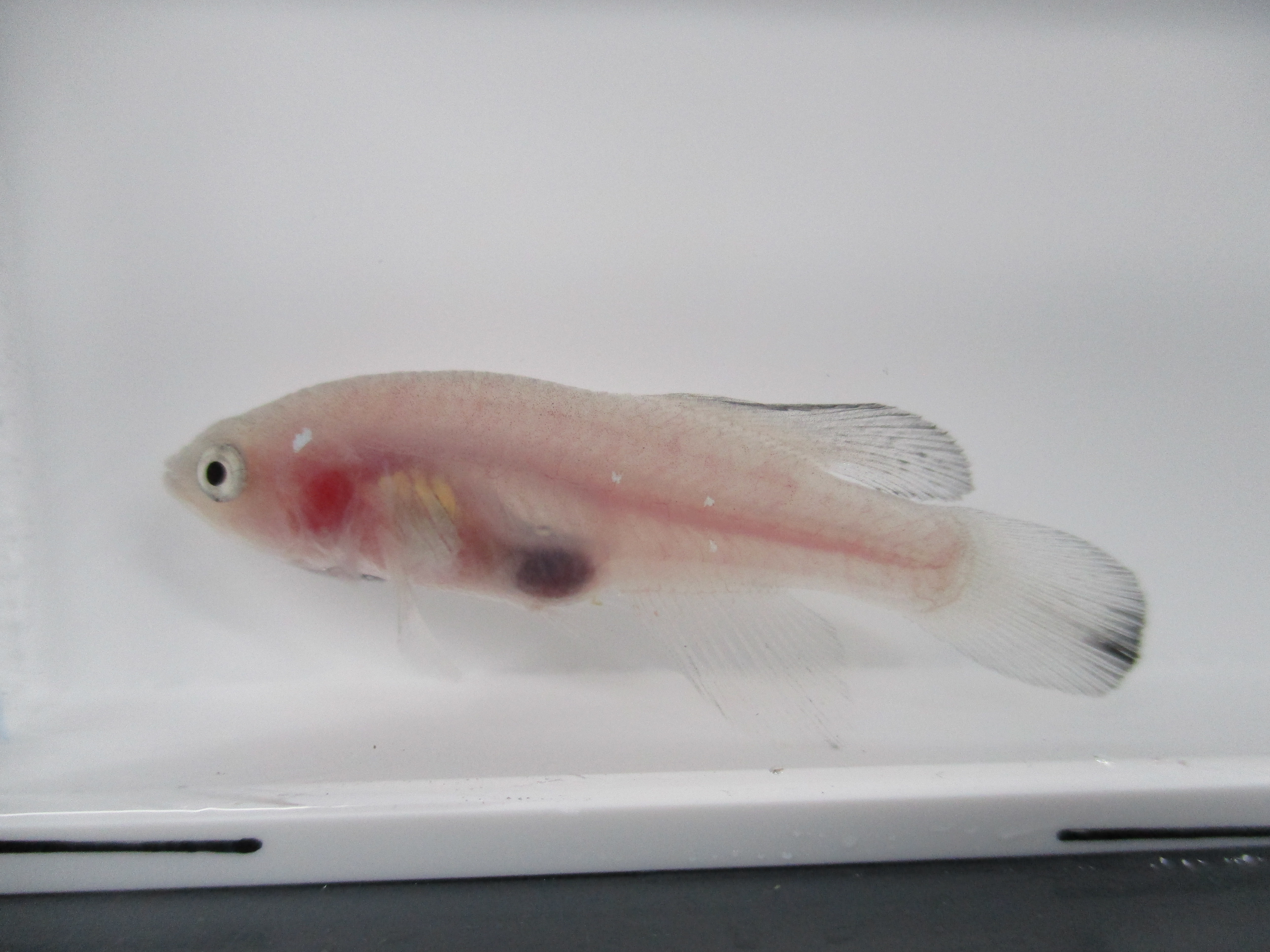

Supplement: Figure 2—source data 1. [file elife-81549-fig2-data1.zip › Figure_2_source_data/Figure_2_panel_CD_C ́D ́_source_data/male_42dph.JPG]

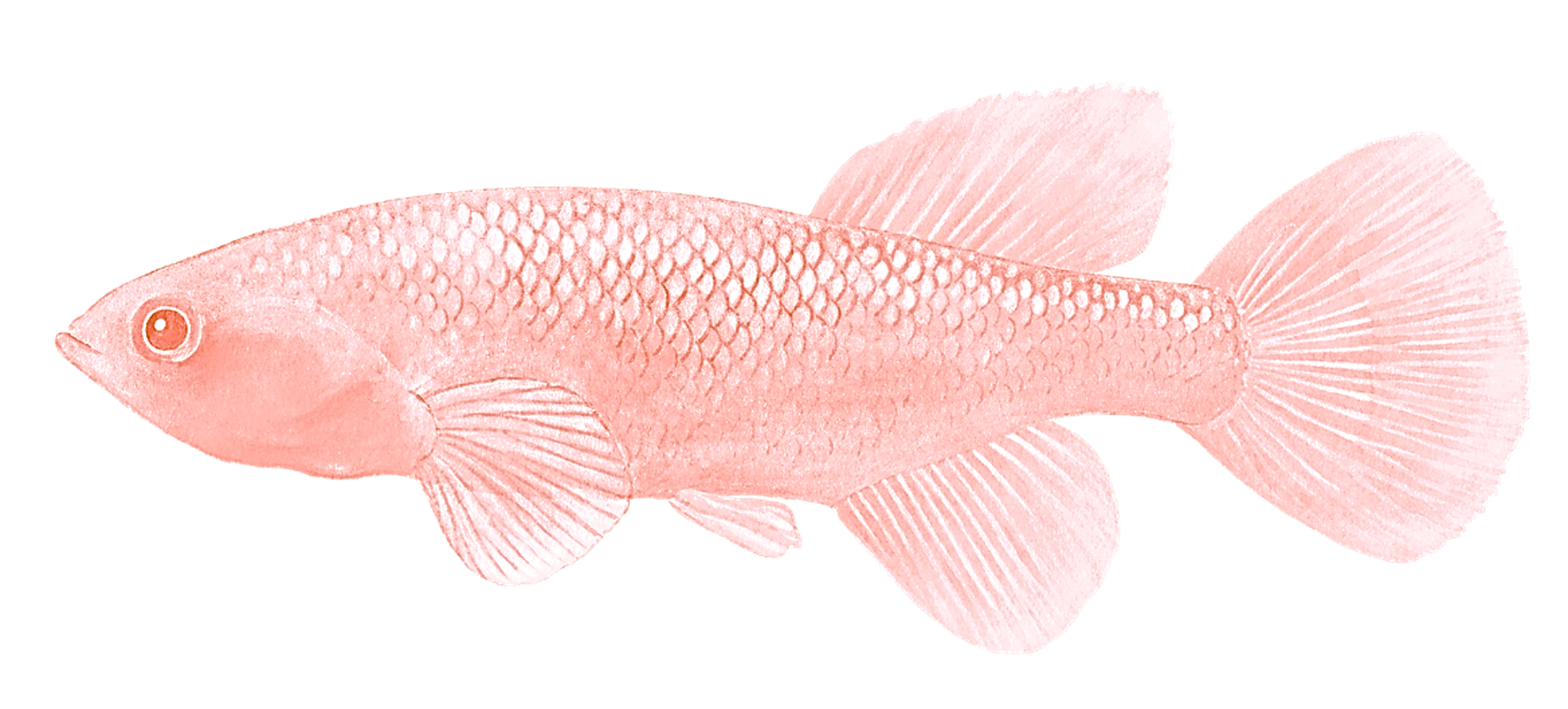

Supplement: Figure 3—source data 1. [file elife-81549-fig3-data1.zip › Figure_3_source_data/Figure_3_panel_B_source_data/female.png]

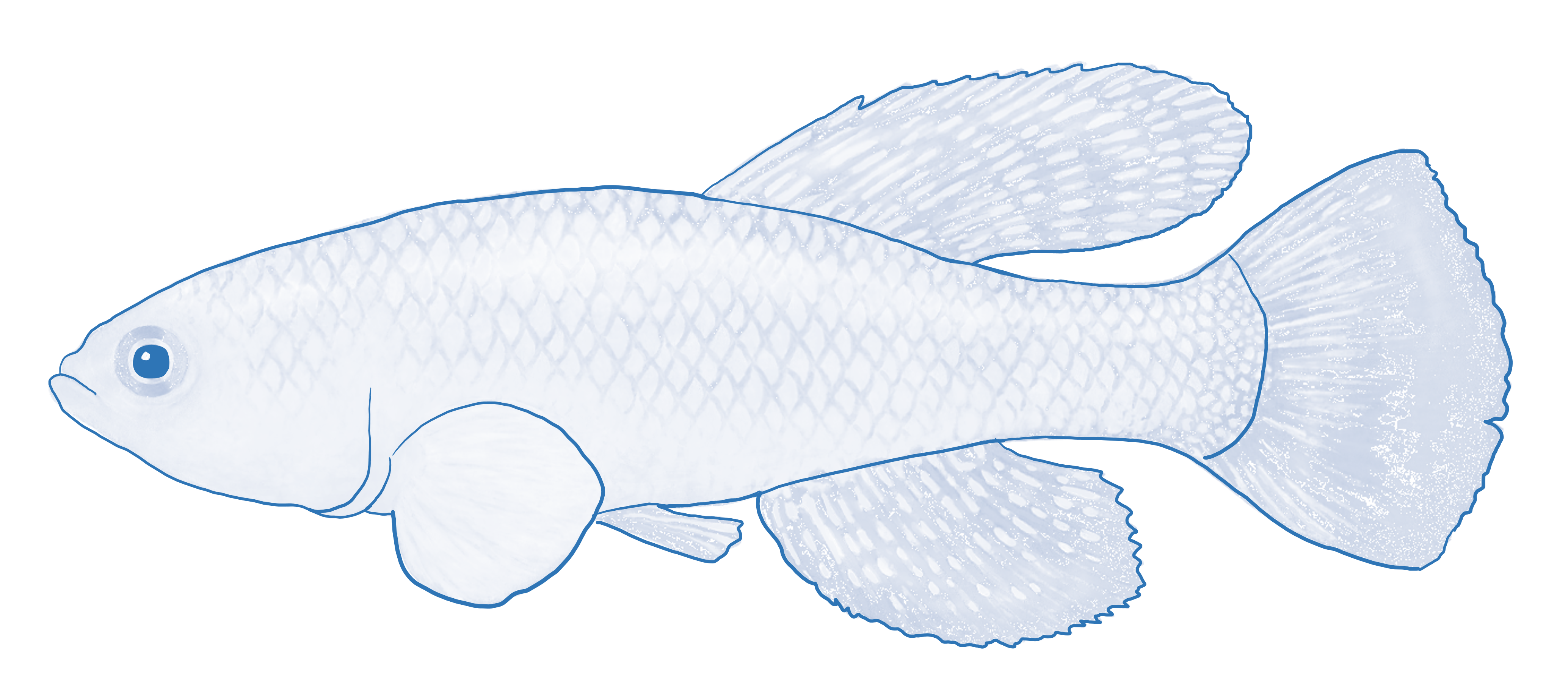

Supplement: Figure 3—source data 1. [file elife-81549-fig3-data1.zip › Figure_3_source_data/Figure_3_panel_B_source_data/Male klara.png]

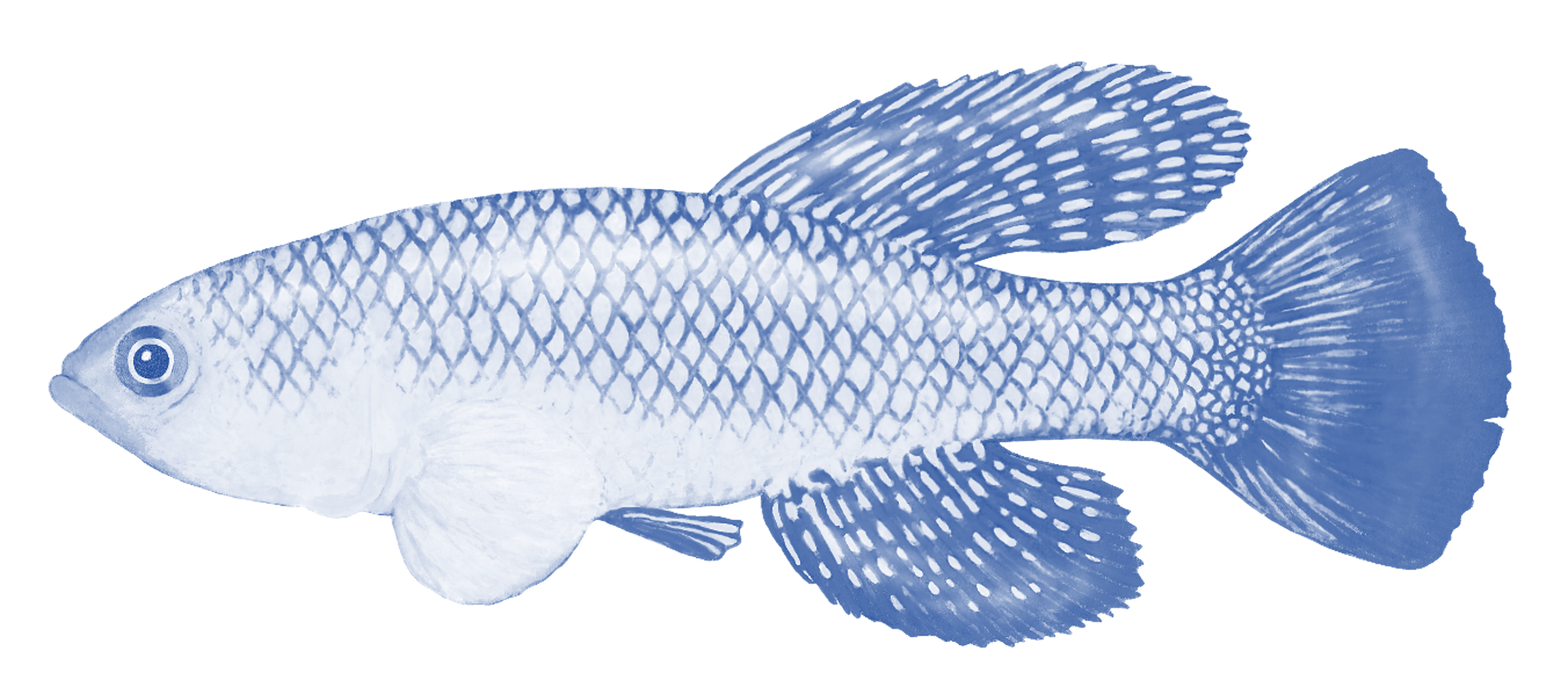

Supplement: Figure 3—source data 1. [file elife-81549-fig3-data1.zip › Figure_3_source_data/Figure_3_panel_B_source_data/Male.png]

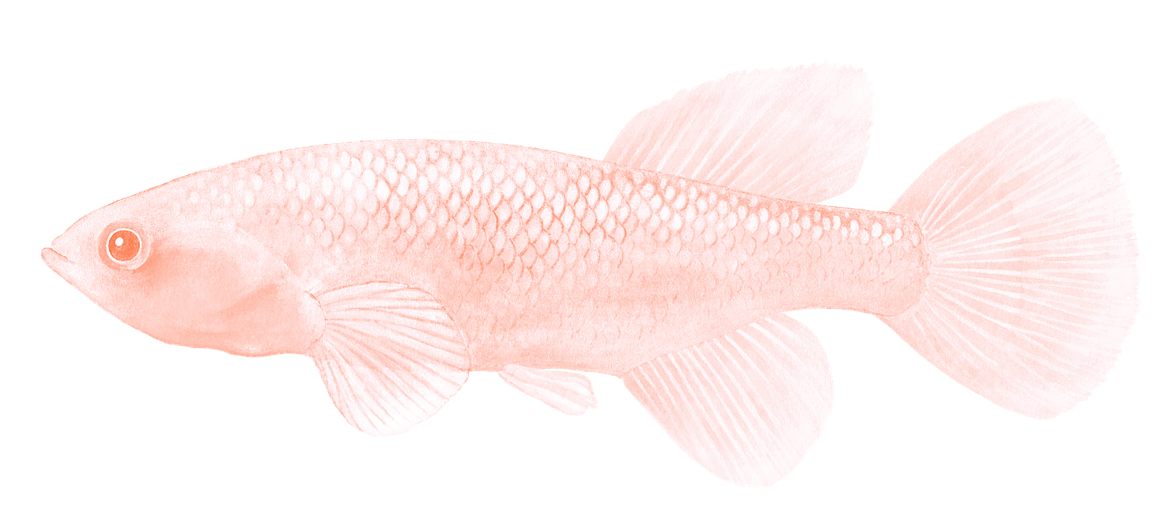

Supplement: Figure 3—source data 1. [file elife-81549-fig3-data1.zip › Figure_3_source_data/Figure_3_panel_B_source_data/Picture1.png]

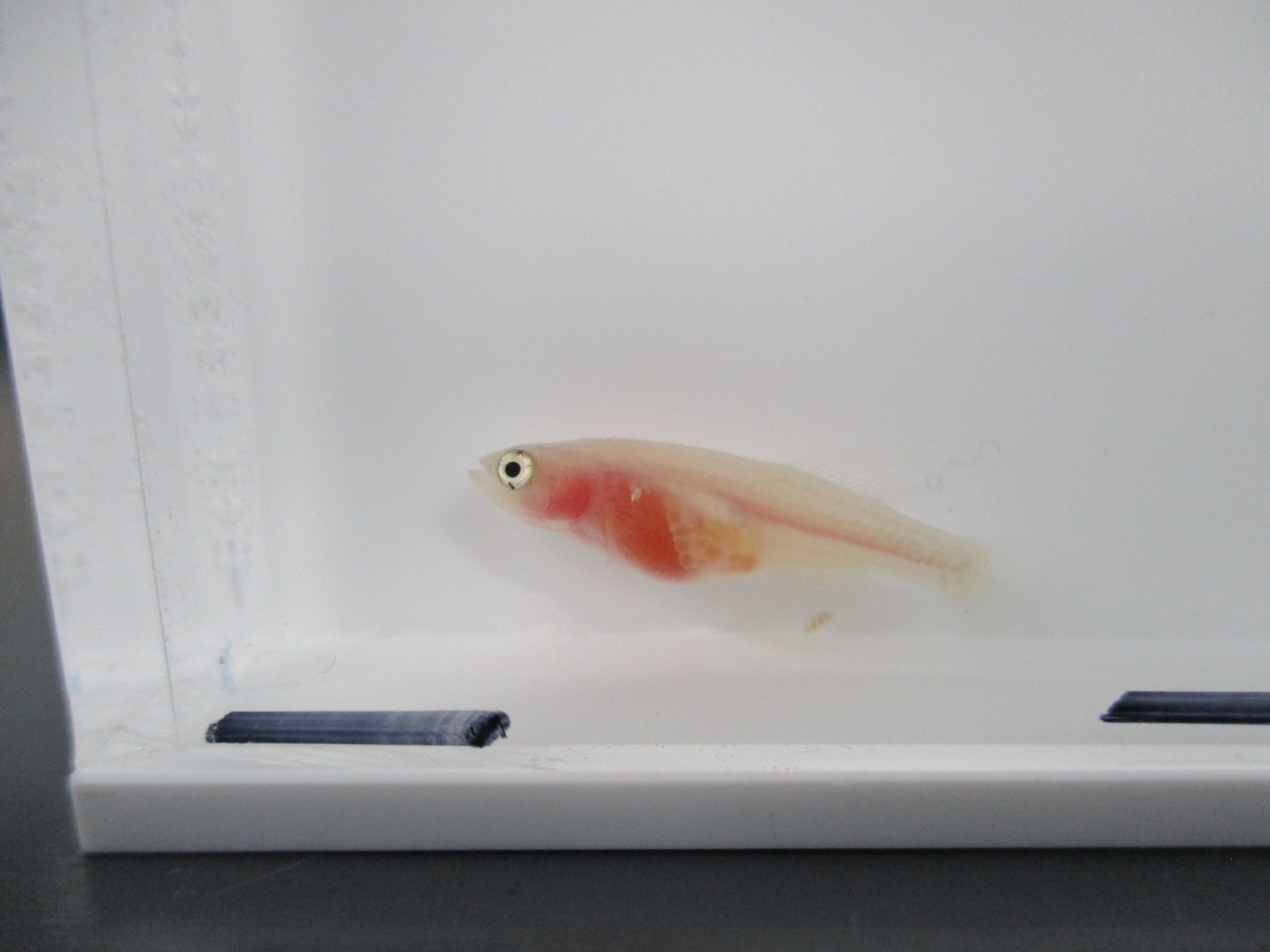

Supplement: Figure 4—source data 1. [file elife-81549-fig4-data1.zip › Figure_4_source_data/Figure_4_panel_A_source_data/female_klara.JPG]

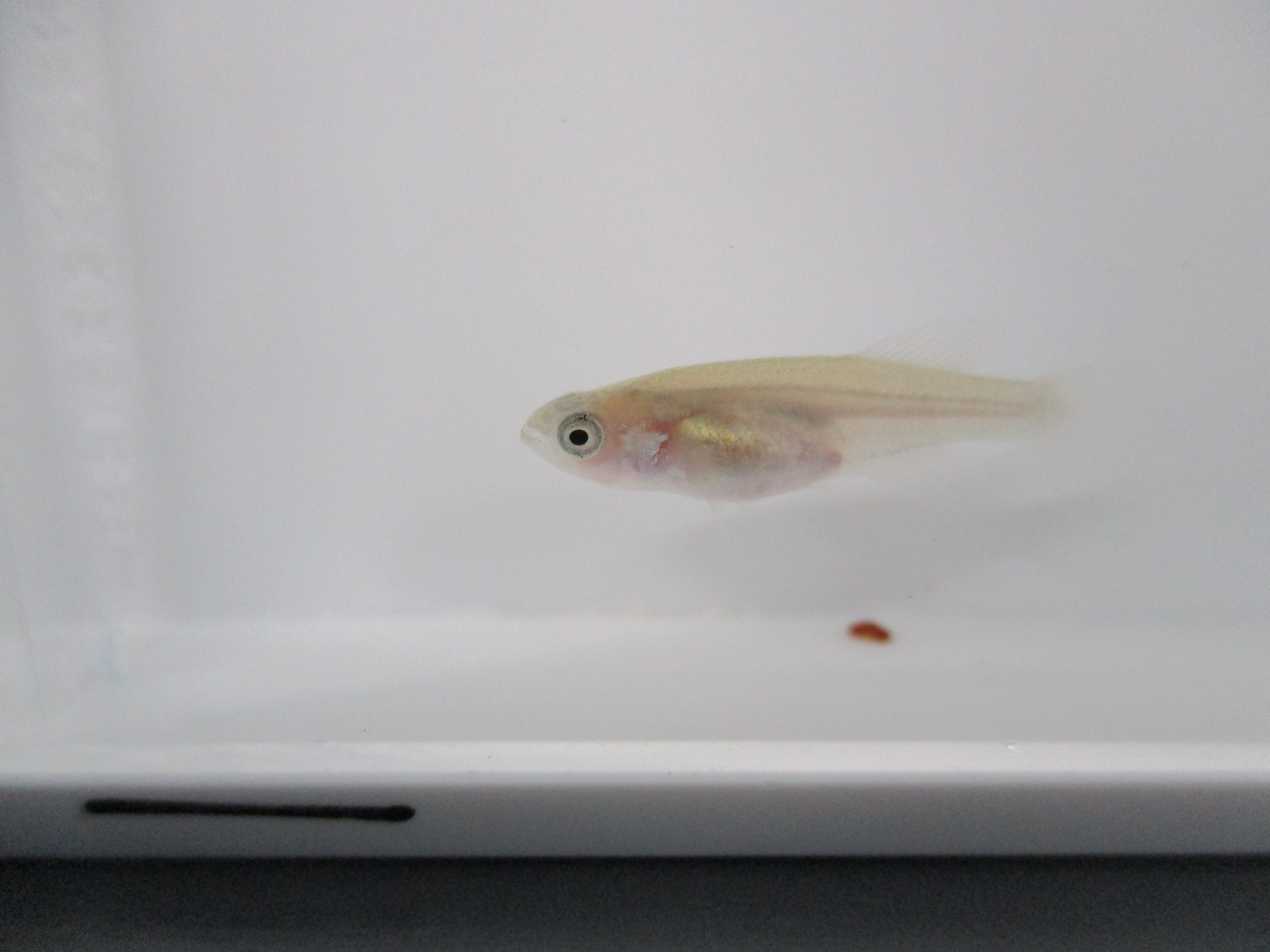

Supplement: Figure 4—source data 1. [file elife-81549-fig4-data1.zip › Figure_4_source_data/Figure_4_panel_A_source_data/female_WT.JPG]

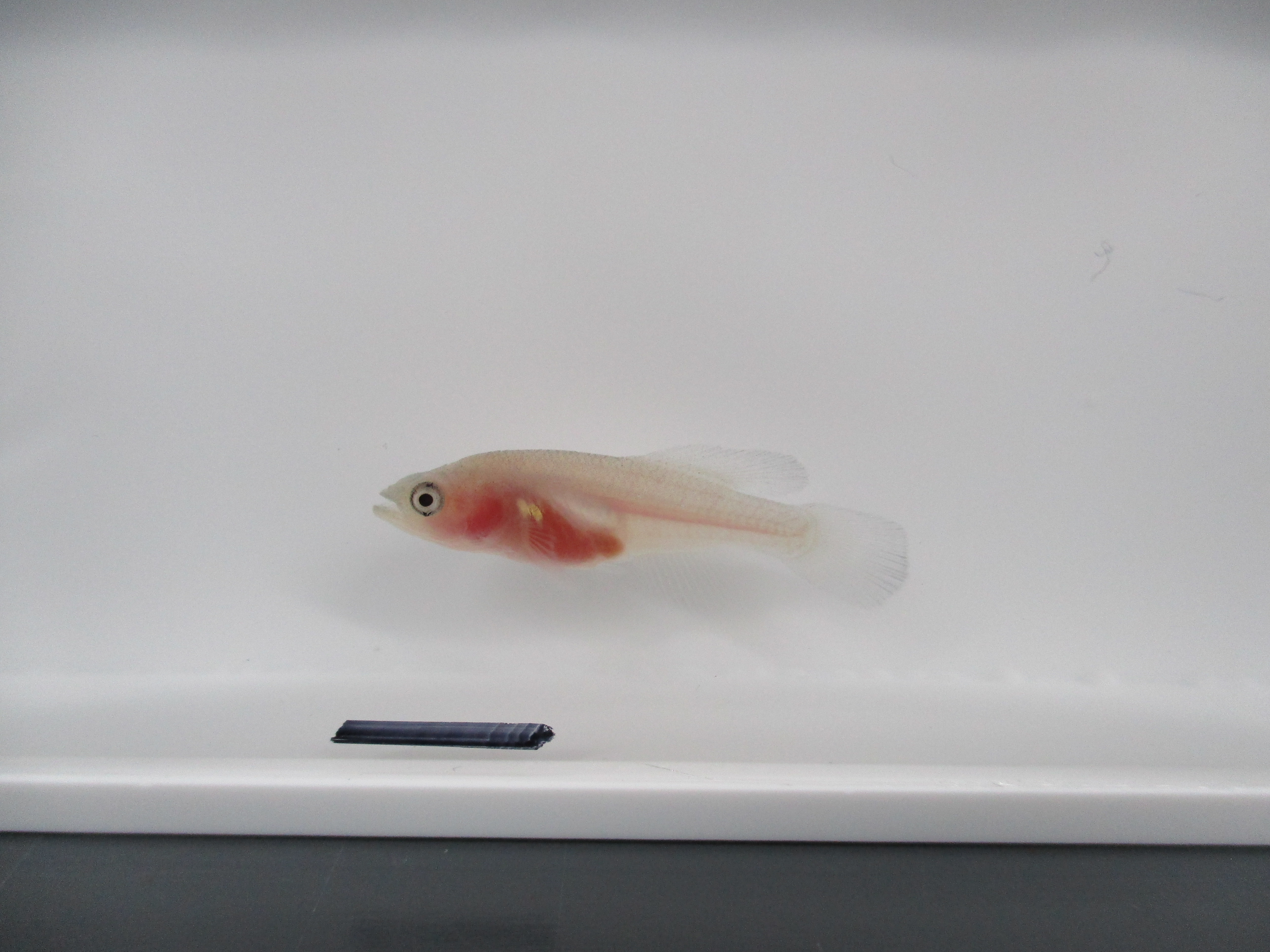

Supplement: Figure 4—source data 1. [file elife-81549-fig4-data1.zip › Figure_4_source_data/Figure_4_panel_A_source_data/male_klara.JPG]

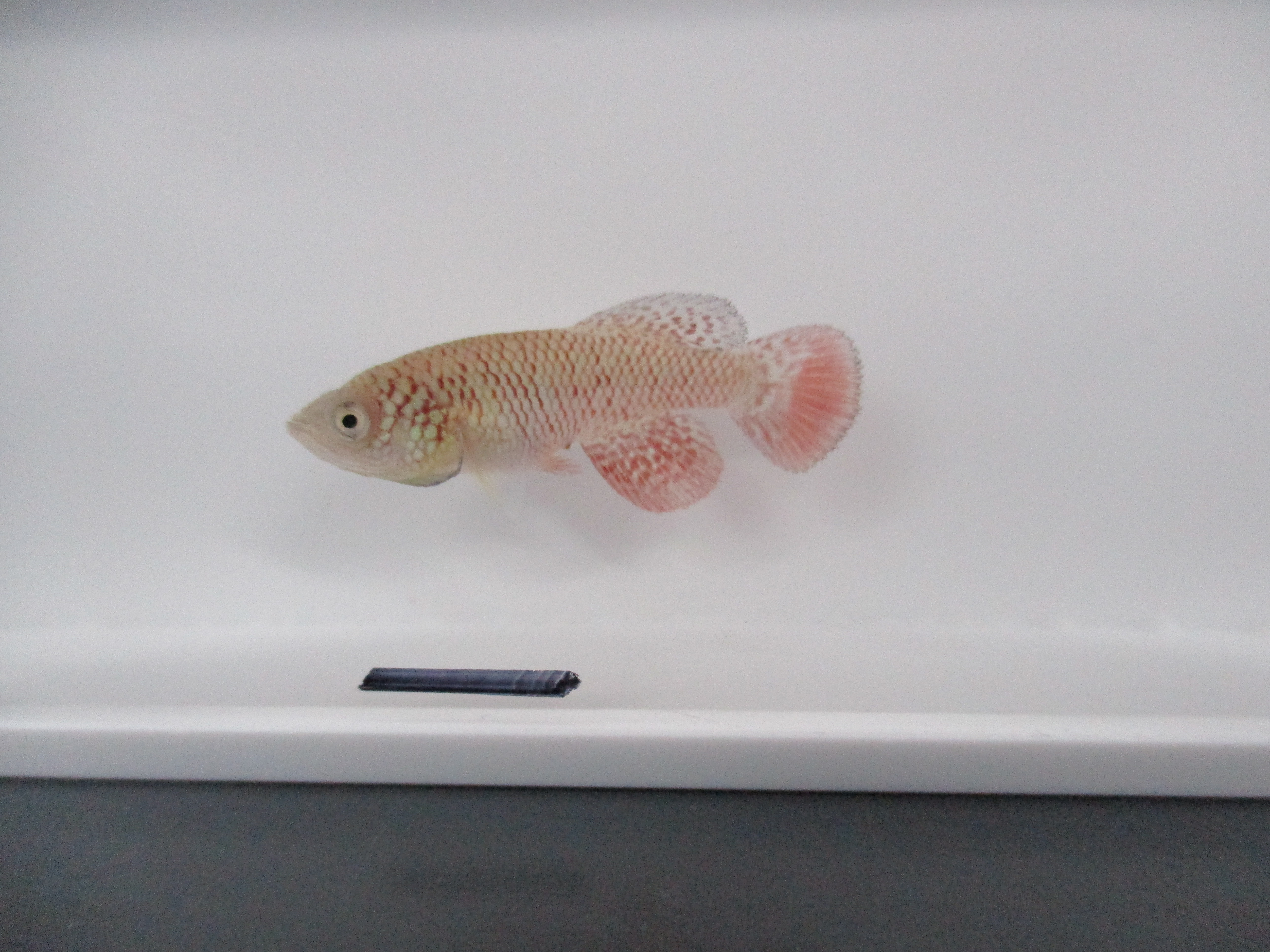

Supplement: Figure 4—source data 1. [file elife-81549-fig4-data1.zip › Figure_4_source_data/Figure_4_panel_A_source_data/male_WT.JPG]

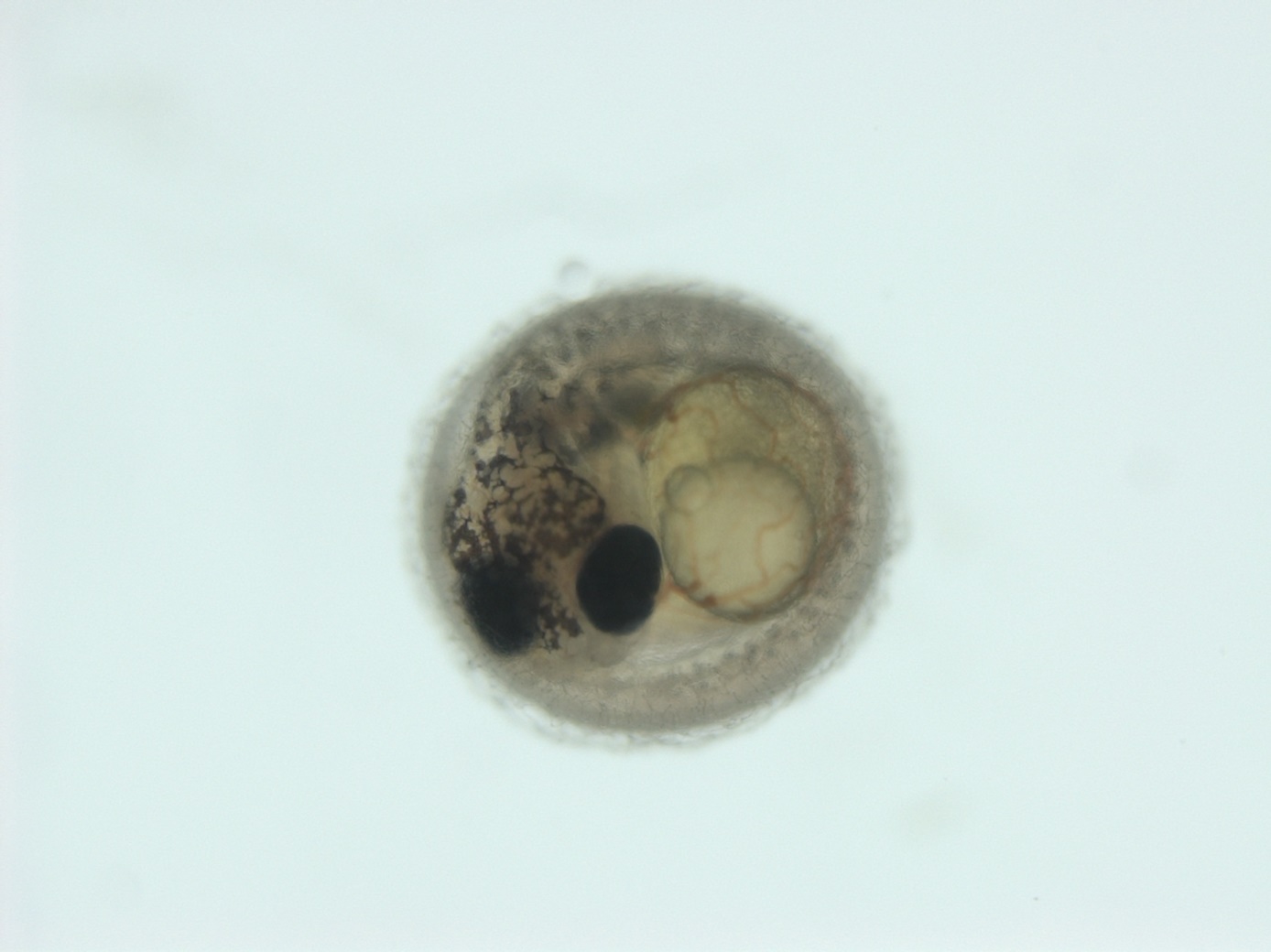

Supplement: Figure 4—source data 1. [file elife-81549-fig4-data1.zip › Figure_4_source_data/Figure_4_panel_B_source_data/Control/Uninjected_H1_1-Image Export-01/Uninjected_H1_1-Image Export-01.jpg]

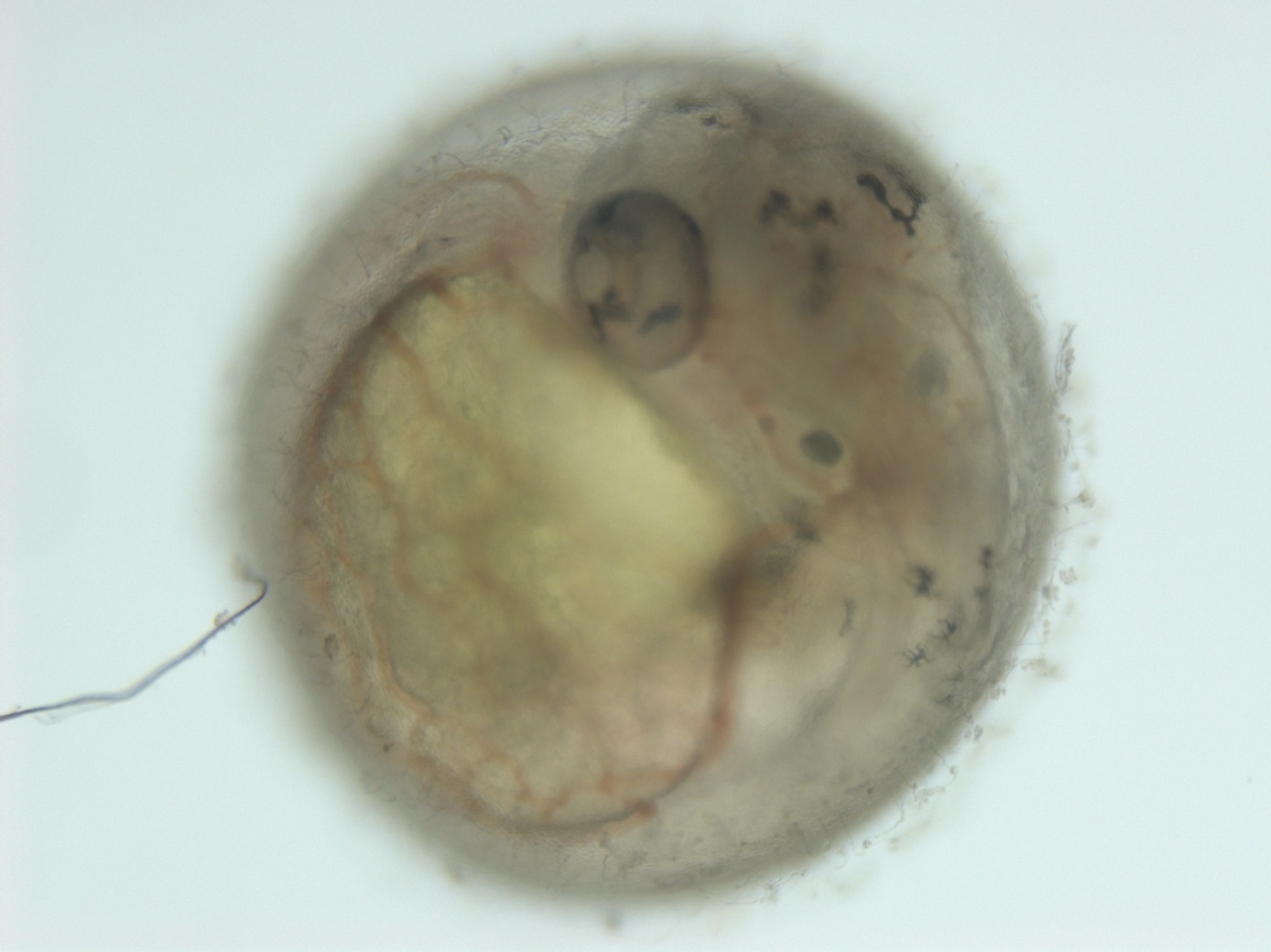

Supplement: Figure 4—source data 1. [file elife-81549-fig4-data1.zip › Figure_4_source_data/Figure_4_panel_B_source_data/GFP-positive/GFP-positive_C6_1-Image Export-03/GFP-positive_C6_1-Image Export-03.jpg]

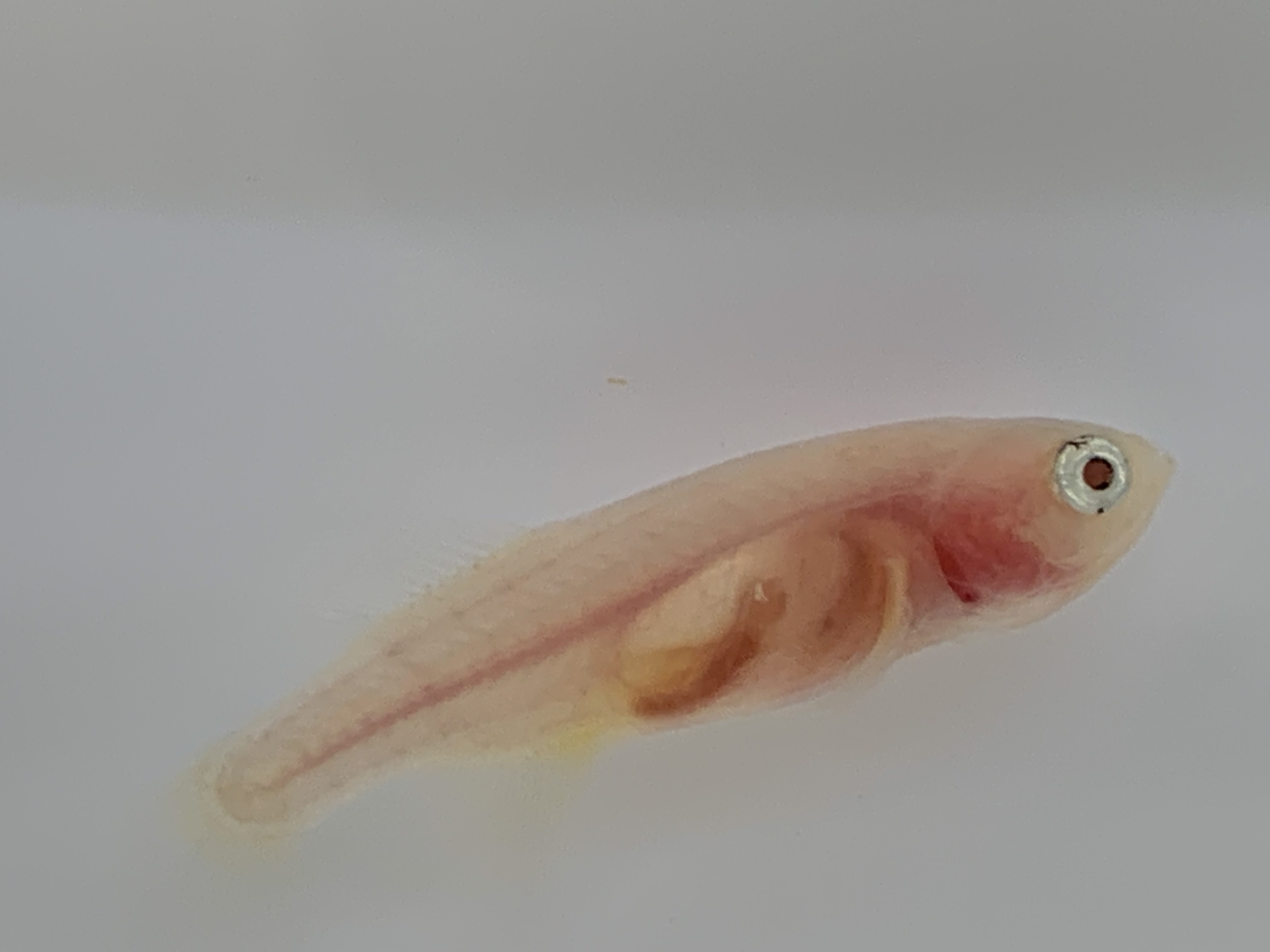

Supplement: Figure 4—source data 1. [file elife-81549-fig4-data1.zip › Figure_4_source_data/Figure_4_panel_C_source_data/F0_fish.JPG]

# Supplement Figure 4

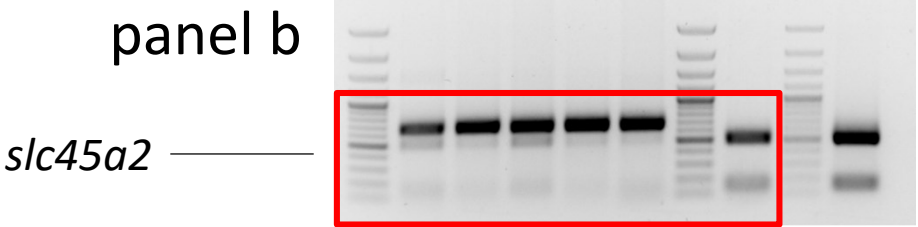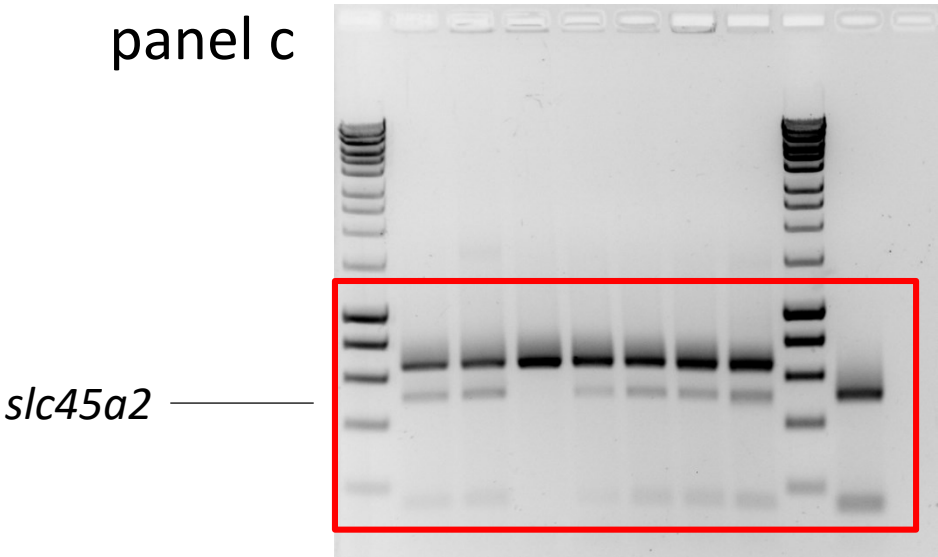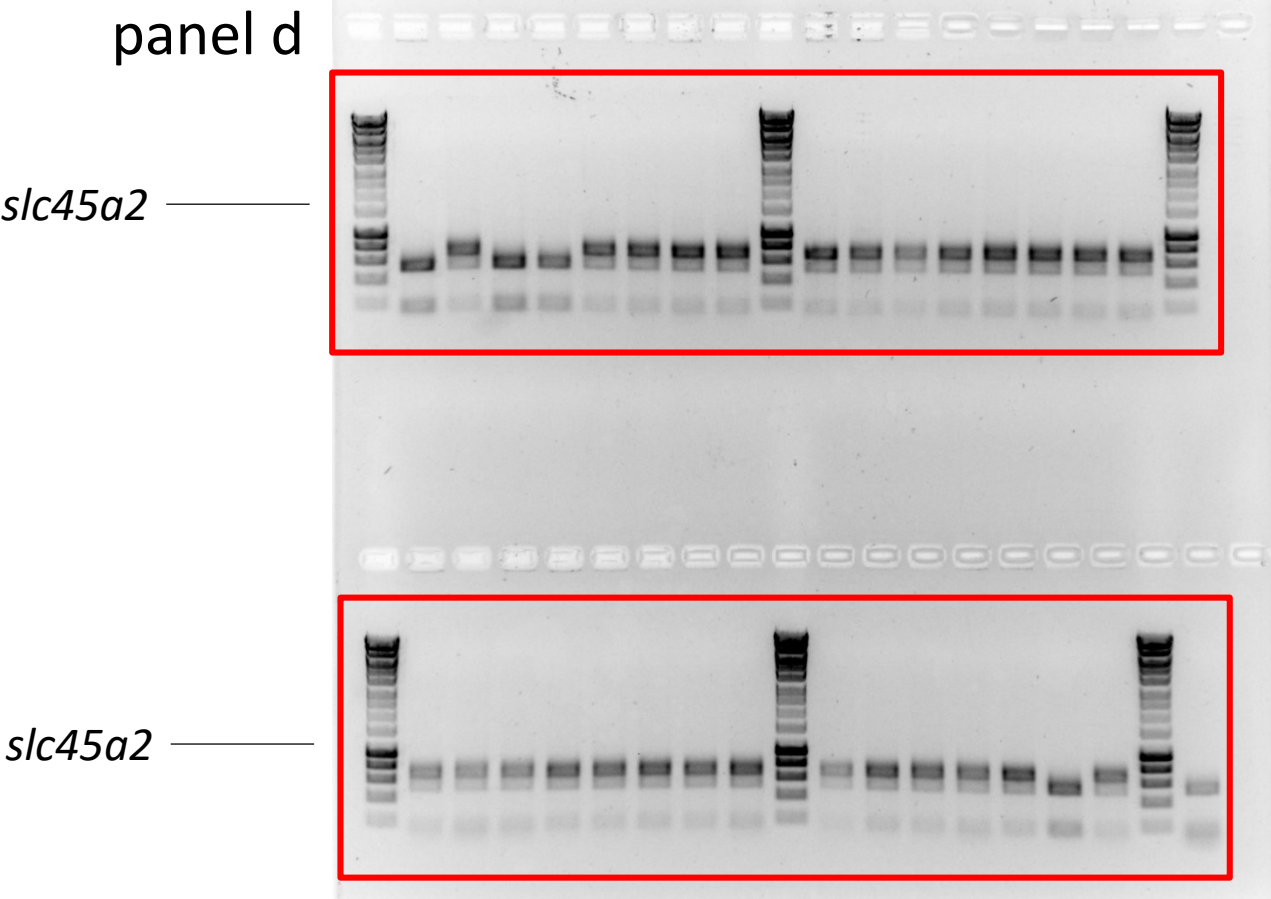

The red box surrounds the lanes shown in the respective panel.

Supplement: Figure 4—figure supplement 1—source data 1. [file elife-81549-fig4-figsupp1-data1.zip › Figure_4_figure_supplement_1_source_data/Figure_4_figure_supplement_1_panel_BCD_source_data/Figure_4_figure_supplement_1_panel_bcd_source_data.pdf]

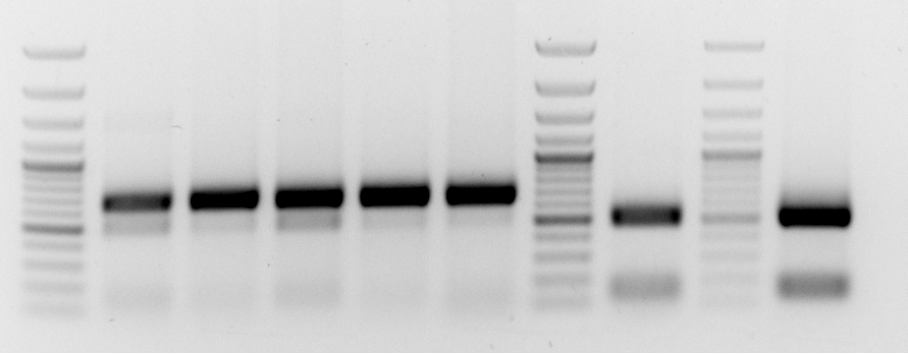

Supplement: Figure 4—figure supplement 1—source data 1. [file elife-81549-fig4-figsupp1-data1.zip › Figure_4_figure_supplement_1_source_data/Figure_4_figure_supplement_1_panel_BCD_source_data/Figure_4_figure_supplement_1_panel_b_source_data.tif]

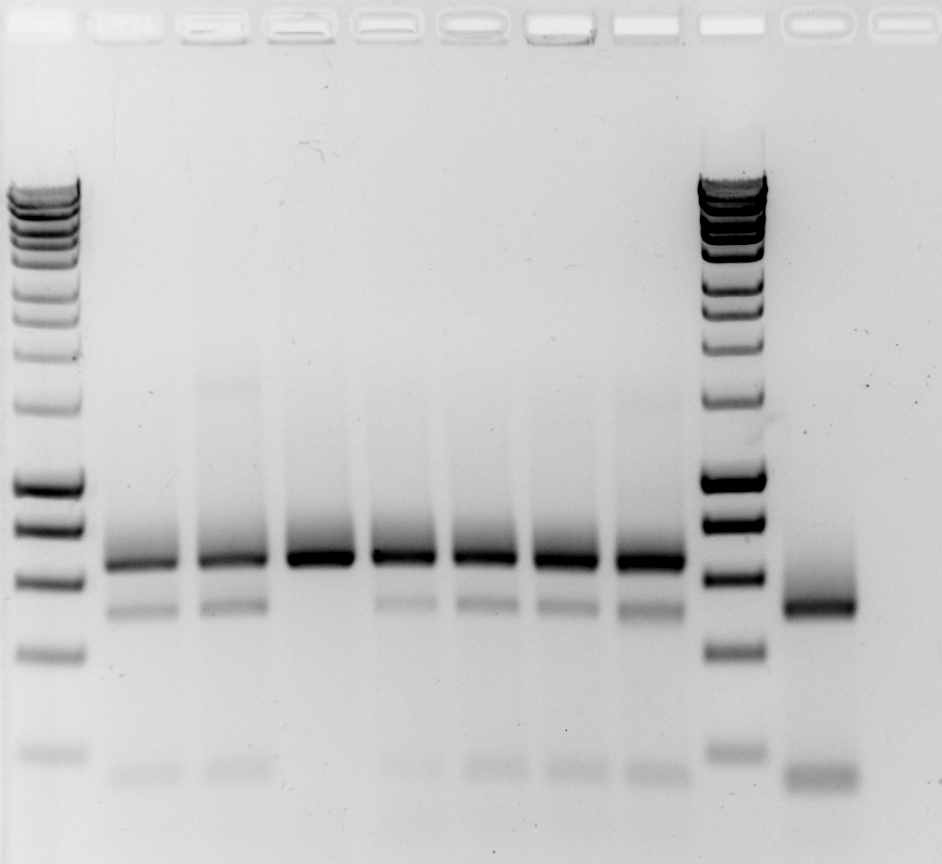

Supplement: Figure 4—figure supplement 1—source data 1. [file elife-81549-fig4-figsupp1-data1.zip › Figure_4_figure_supplement_1_source_data/Figure_4_figure_supplement_1_panel_BCD_source_data/Figure_4_figure_supplement_1_panel_c_source_data.tif]

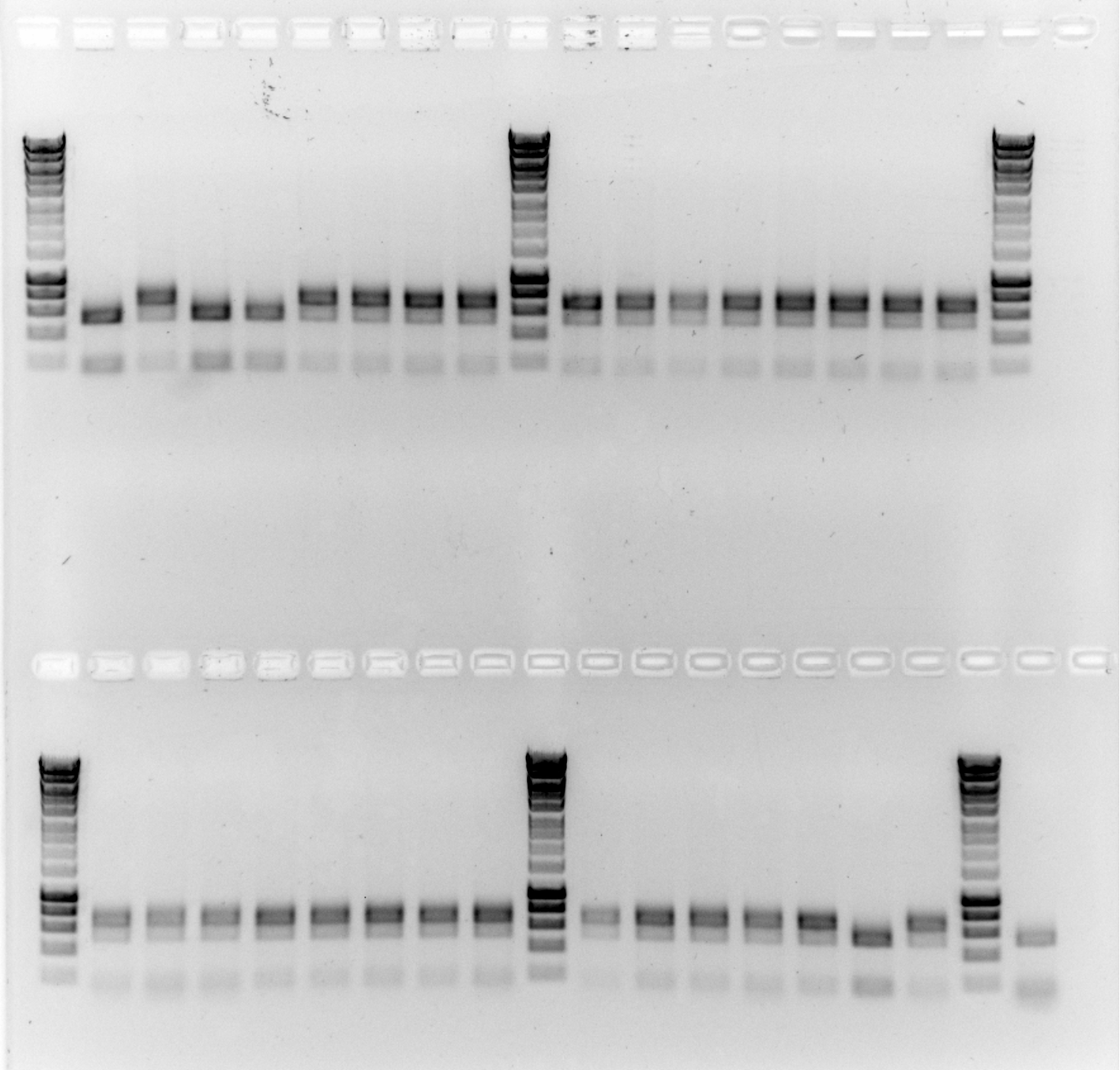

Supplement: Figure 4—figure supplement 1—source data 1. [file elife-81549-fig4-figsupp1-data1.zip › Figure_4_figure_supplement_1_source_data/Figure_4_figure_supplement_1_panel_BCD_source_data/Figure_4_figure_supplement_1_panel_d_source_data.tif]

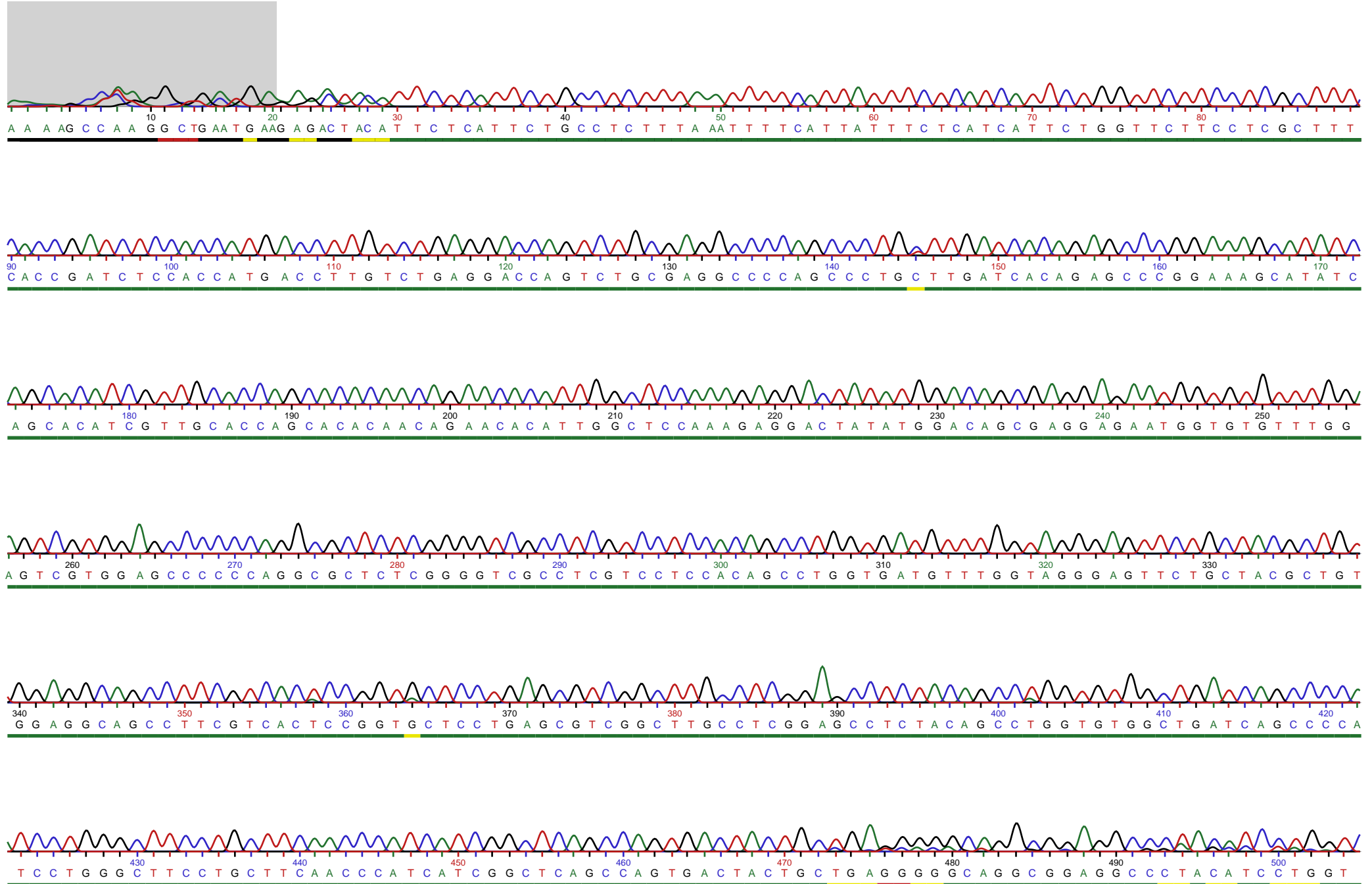

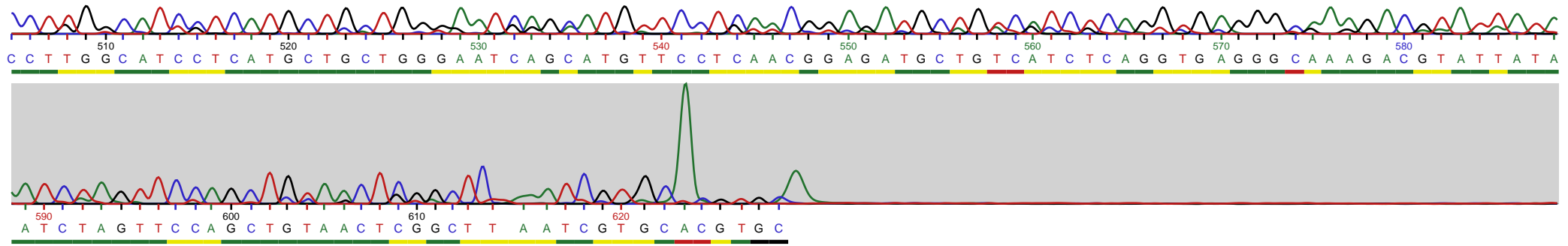

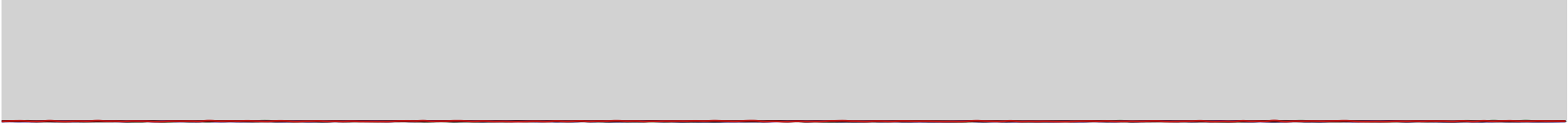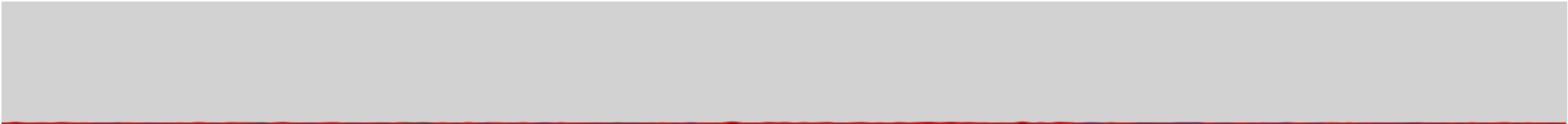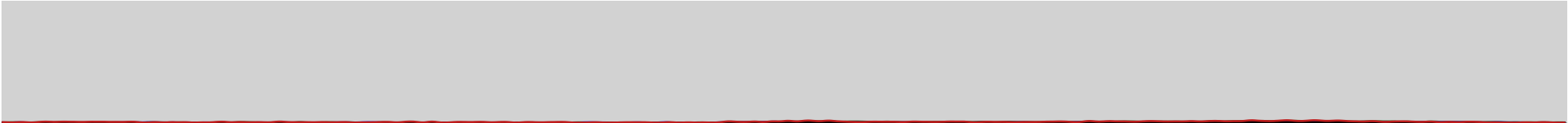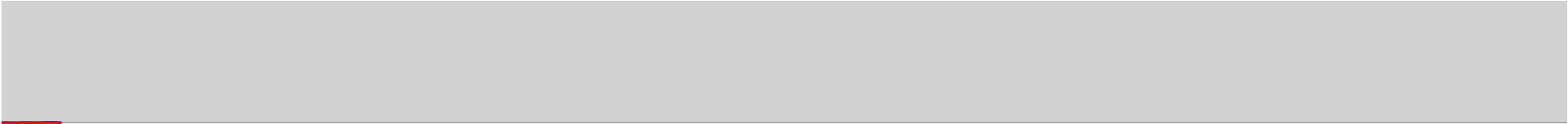

Supplement: Figure 4—figure supplement 1—source data 1. [file elife-81549-fig4-figsupp1-data1.zip › Figure_4_figure_supplement_1_source_data/Figure_4_figure_supplement_1_panel_E_source_data/Originals_Sequencing_data/Fish_9/#9.pdf]

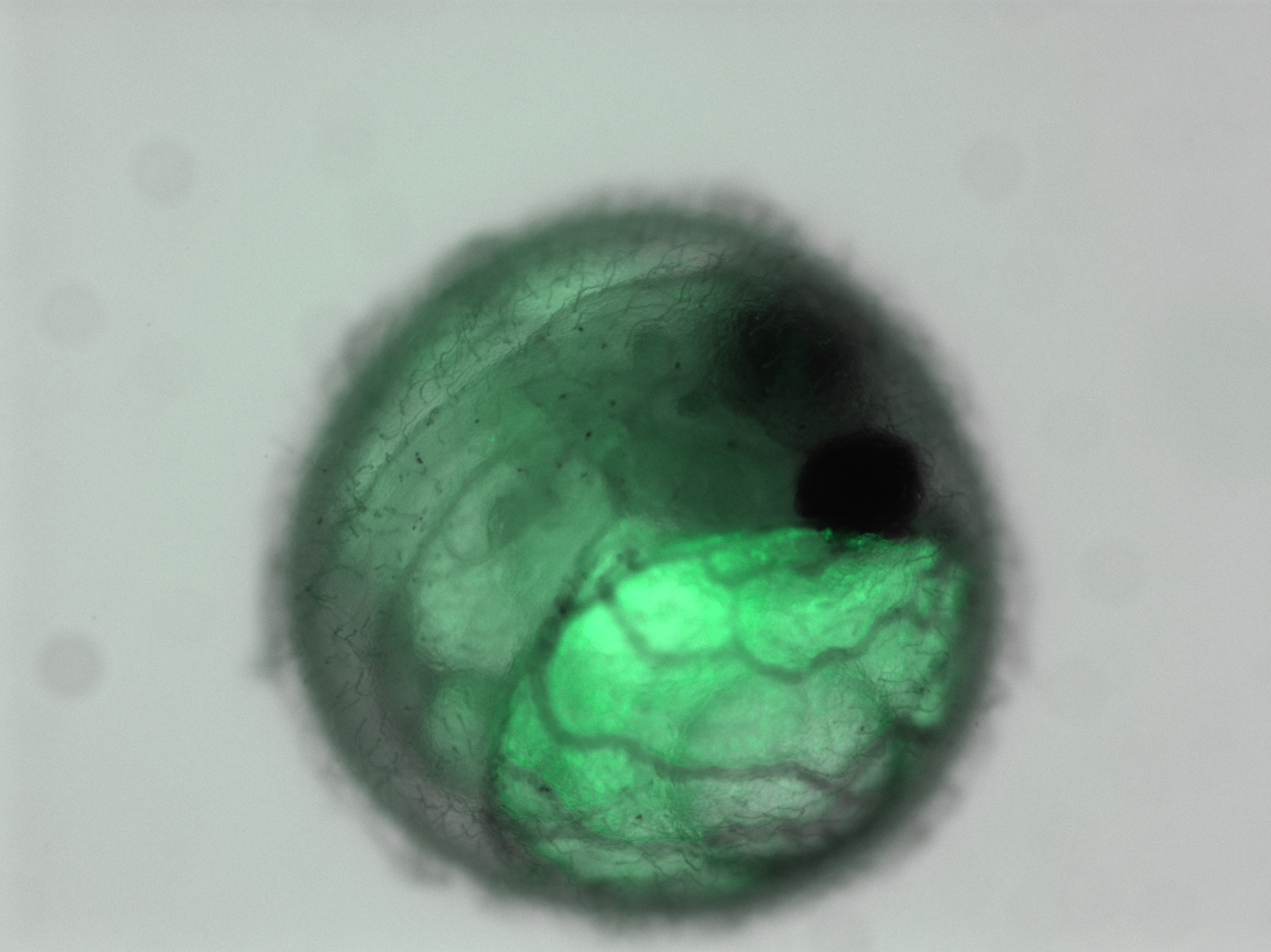

Supplement: Figure 5—source data 1. [file elife-81549-fig5-data1.zip › Figure_5_source_data/Figure_5_panel_CD_source_data/cdkn1a++_24hpi-Image Export-01/cdkn1a++_24hpi-Image Export-01_c1-2.tif]

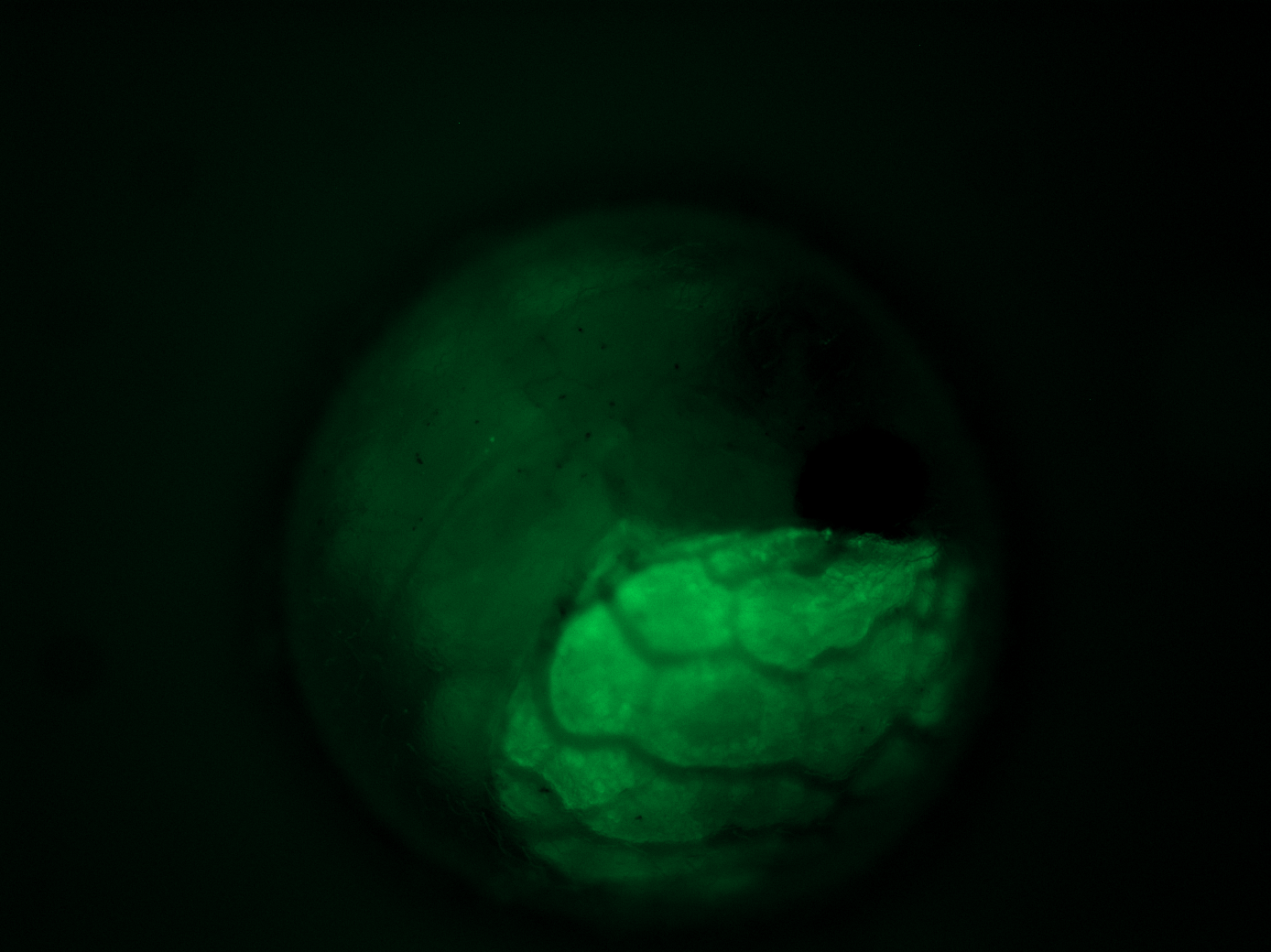

Supplement: Figure 5—source data 1. [file elife-81549-fig5-data1.zip › Figure_5_source_data/Figure_5_panel_CD_source_data/cdkn1a++_24hpi-Image Export-01/cdkn1a++_24hpi-Image Export-01_c1.tif]

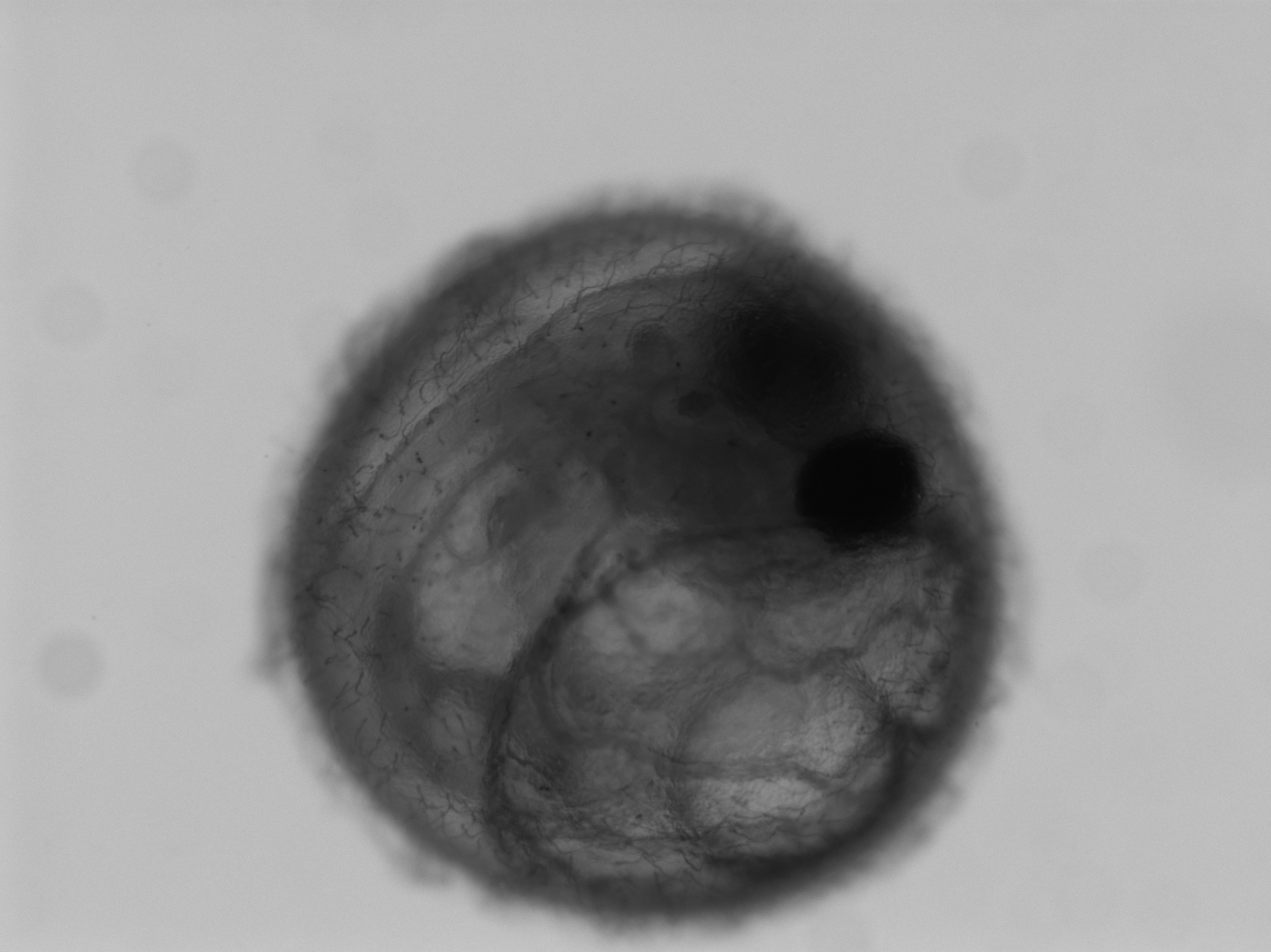

Supplement: Figure 5—source data 1. [file elife-81549-fig5-data1.zip › Figure_5_source_data/Figure_5_panel_CD_source_data/cdkn1a++_24hpi-Image Export-01/cdkn1a++_24hpi-Image Export-01_c2.tif]

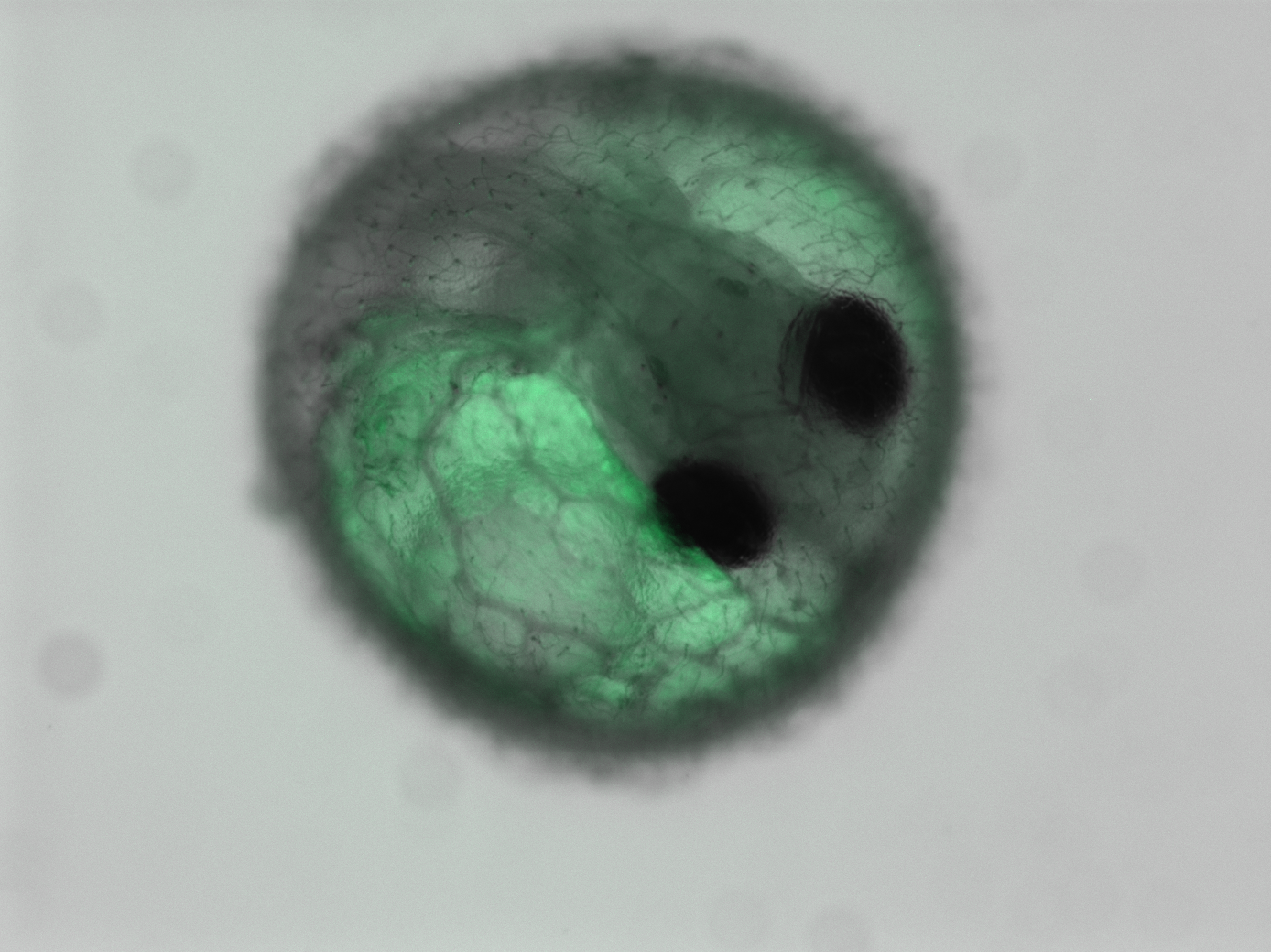

Supplement: Figure 5—source data 1. [file elife-81549-fig5-data1.zip › Figure_5_source_data/Figure_5_panel_CD_source_data/cdkn1a++_before-Image Export-02/cdkn1a++_before-Image Export-02_c1-2.tif]

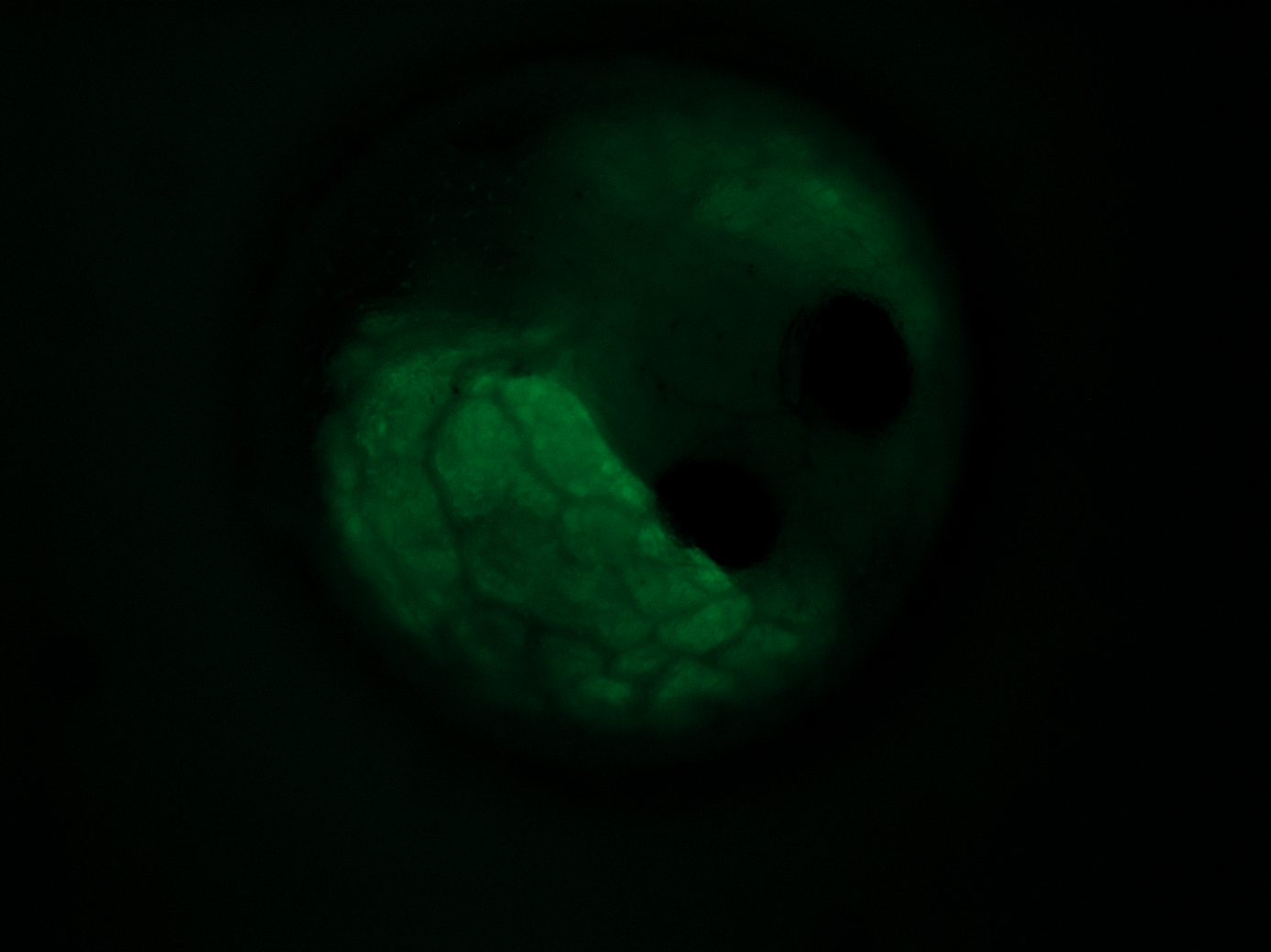

Supplement: Figure 5—source data 1. [file elife-81549-fig5-data1.zip › Figure_5_source_data/Figure_5_panel_CD_source_data/cdkn1a++_before-Image Export-02/cdkn1a++_before-Image Export-02_c1.tif]

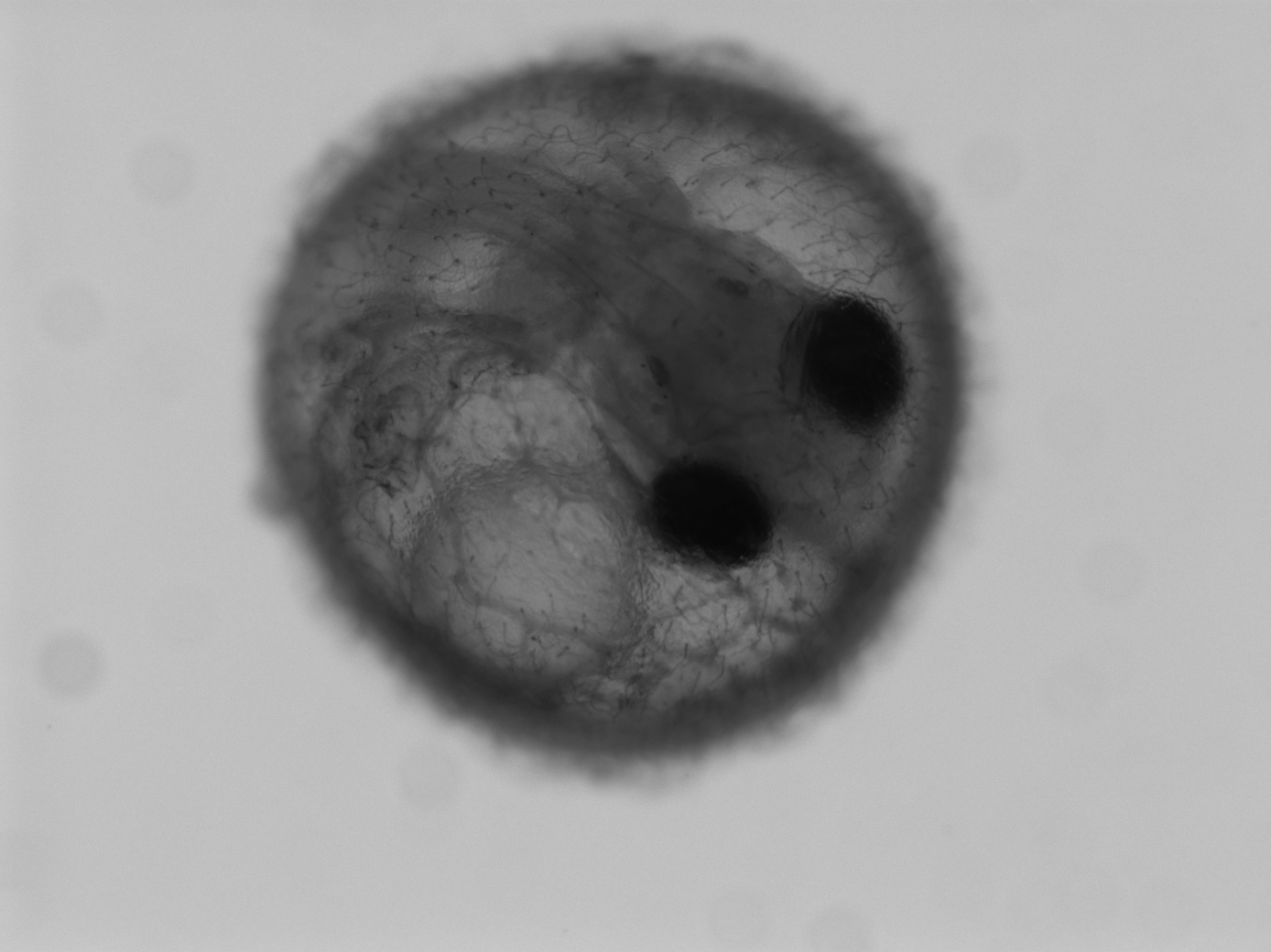

Supplement: Figure 5—source data 1. [file elife-81549-fig5-data1.zip › Figure_5_source_data/Figure_5_panel_CD_source_data/cdkn1a++_before-Image Export-02/cdkn1a++_before-Image Export-02_c2.tif]

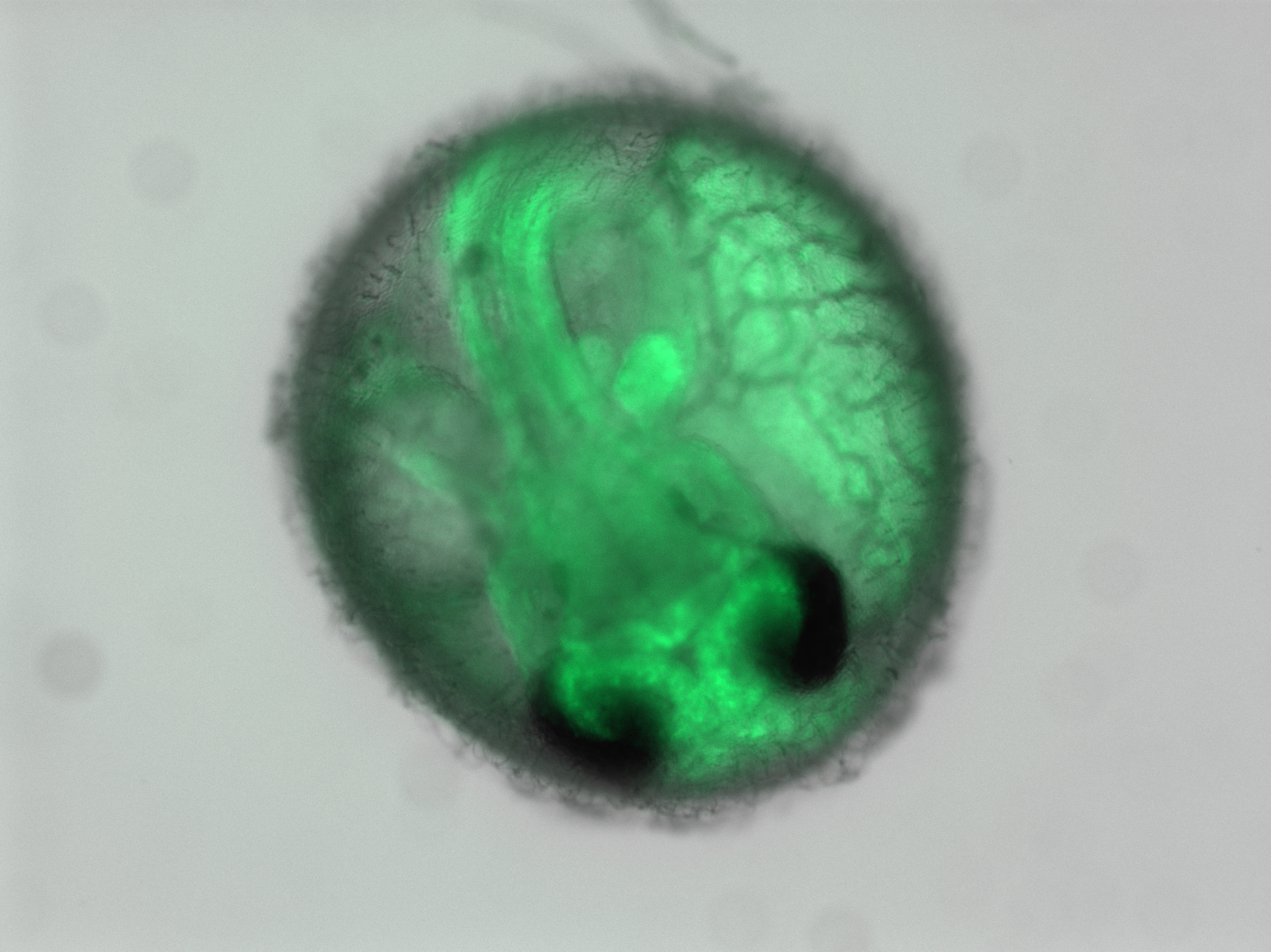

Supplement: Figure 5—source data 1. [file elife-81549-fig5-data1.zip › Figure_5_source_data/Figure_5_panel_CD_source_data/cdkn1aki+_24hpi-Image Export-03/cdkn1aki+_24hpi-Image Export-03_c1-2.tif]

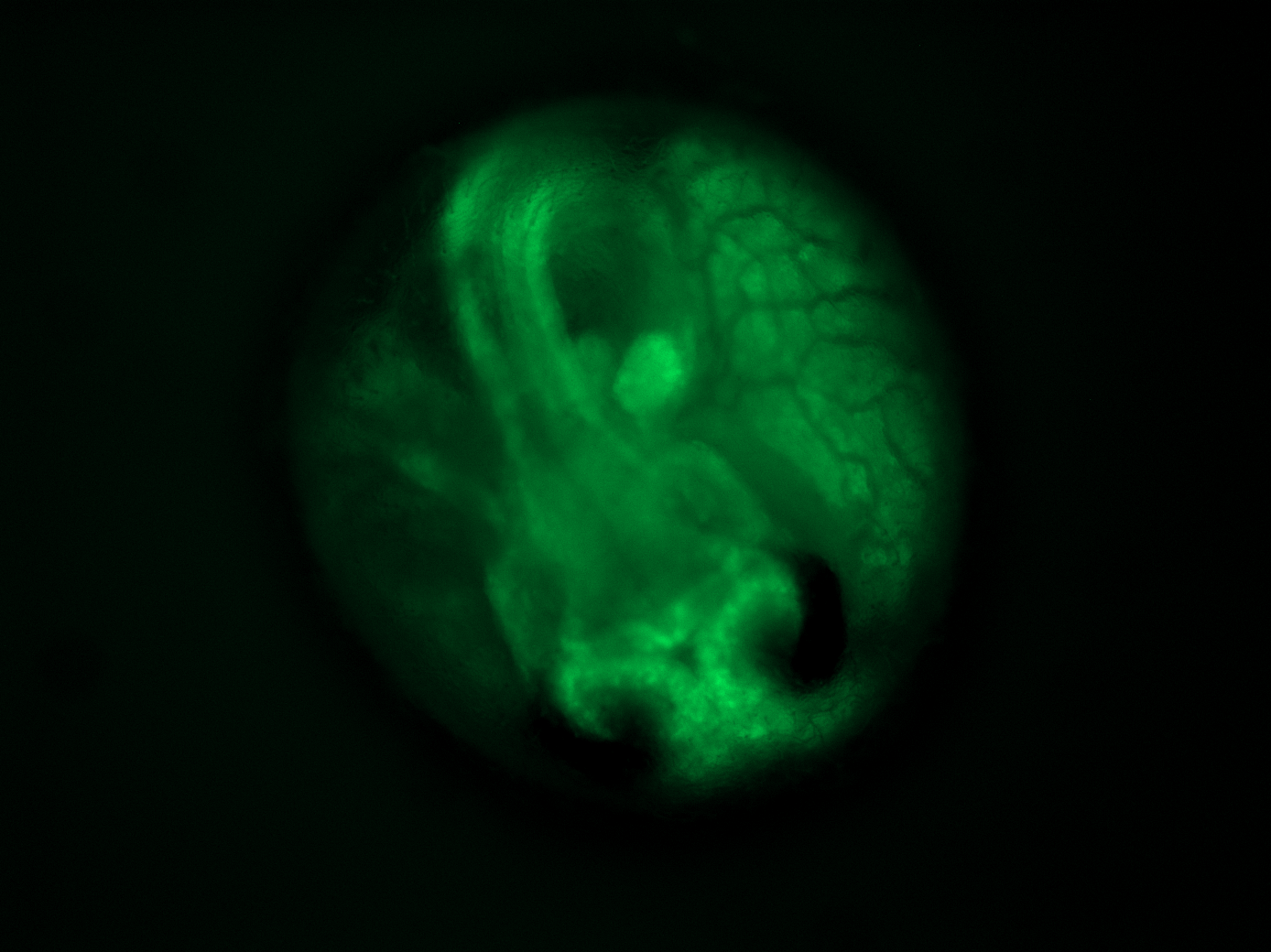

Supplement: Figure 5—source data 1. [file elife-81549-fig5-data1.zip › Figure_5_source_data/Figure_5_panel_CD_source_data/cdkn1aki+_24hpi-Image Export-03/cdkn1aki+_24hpi-Image Export-03_c1.tif]

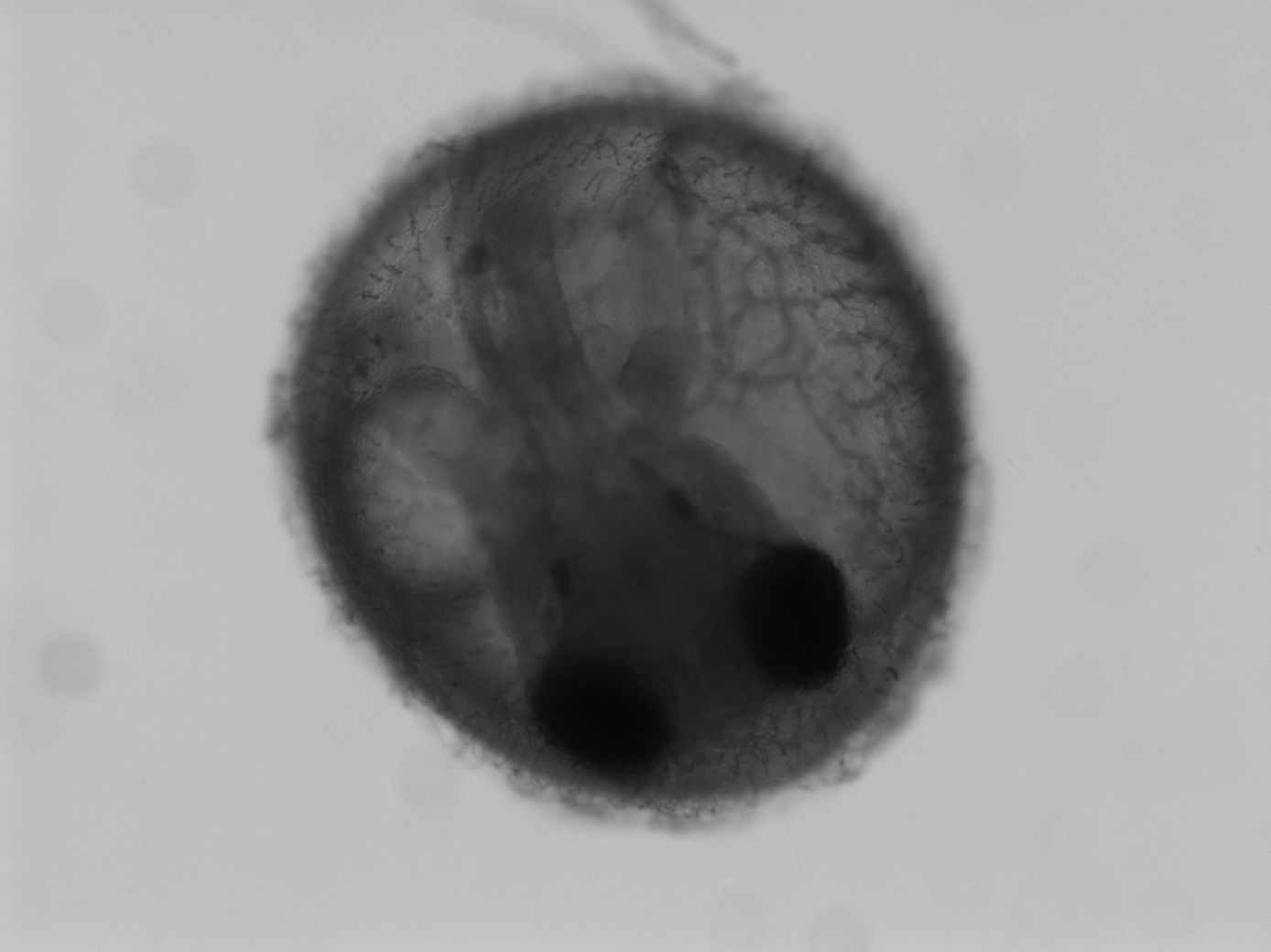

Supplement: Figure 5—source data 1. [file elife-81549-fig5-data1.zip › Figure_5_source_data/Figure_5_panel_CD_source_data/cdkn1aki+_24hpi-Image Export-03/cdkn1aki+_24hpi-Image Export-03_c2.tif]

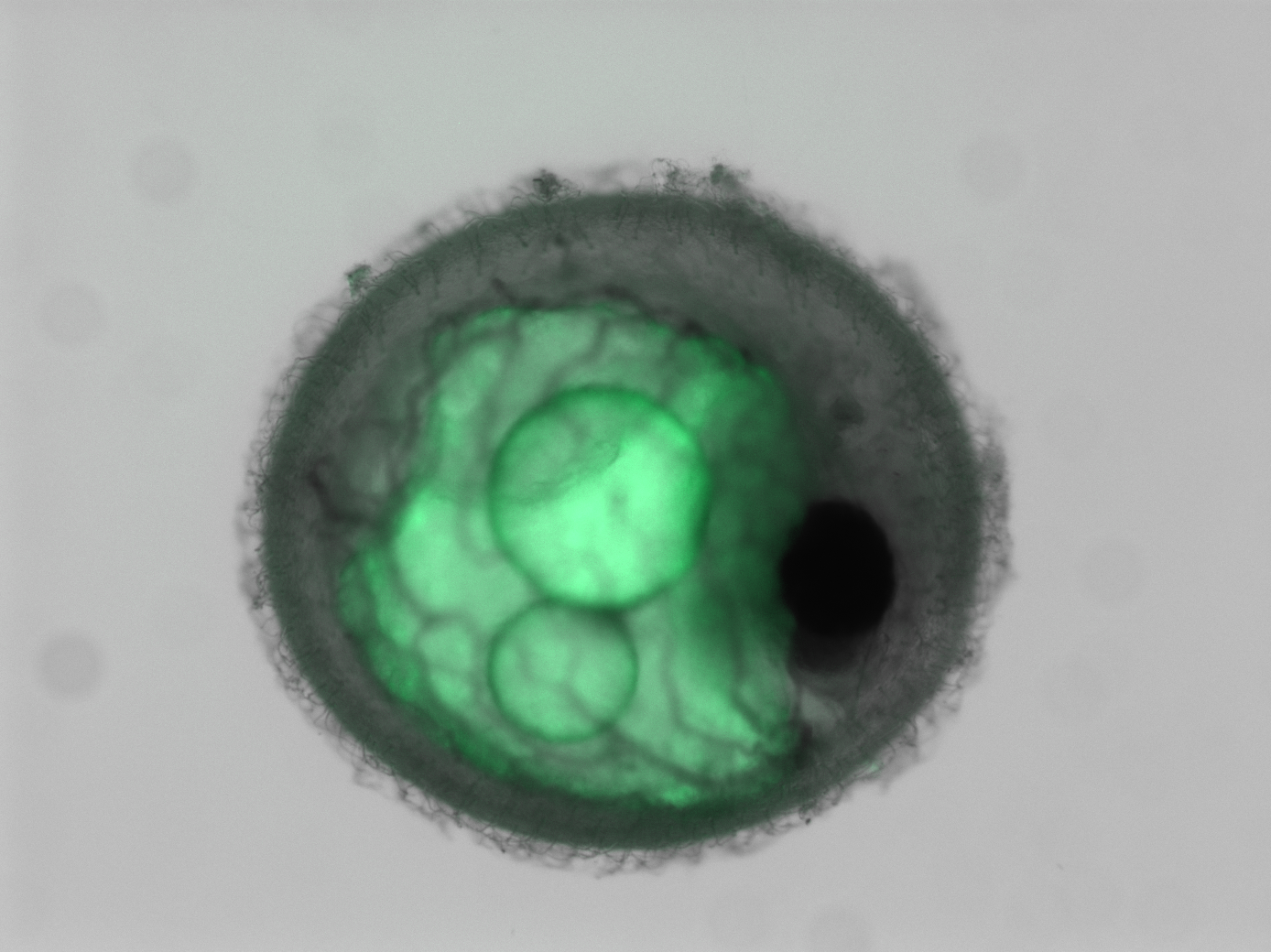

Supplement: Figure 5—source data 1. [file elife-81549-fig5-data1.zip › Figure_5_source_data/Figure_5_panel_CD_source_data/cdkn1aki+_before-Image Export-04/cdkn1aki+_before-Image Export-04_c1-2.tif]

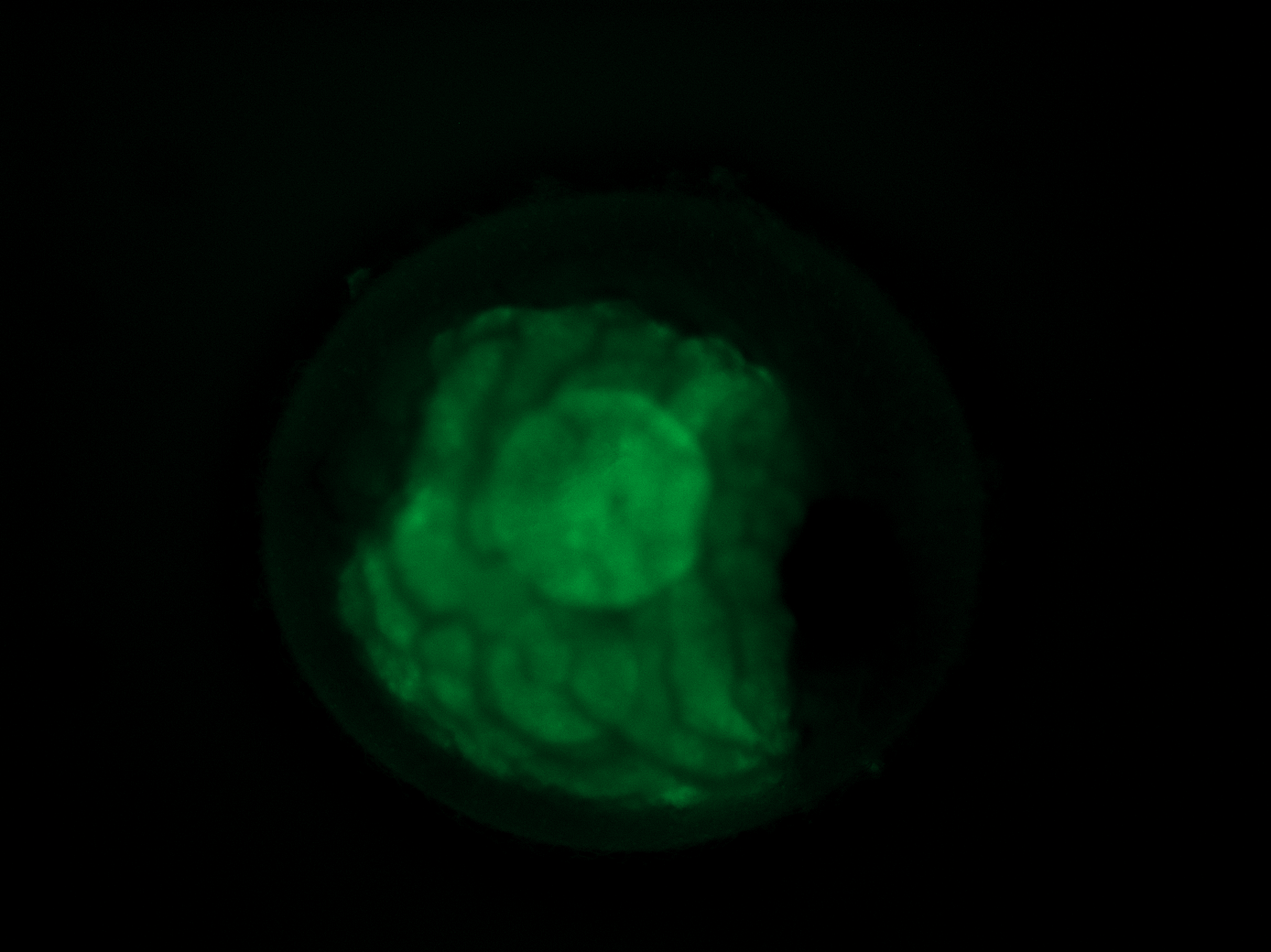

Supplement: Figure 5—source data 1. [file elife-81549-fig5-data1.zip › Figure_5_source_data/Figure_5_panel_CD_source_data/cdkn1aki+_before-Image Export-04/cdkn1aki+_before-Image Export-04_c1.tif]

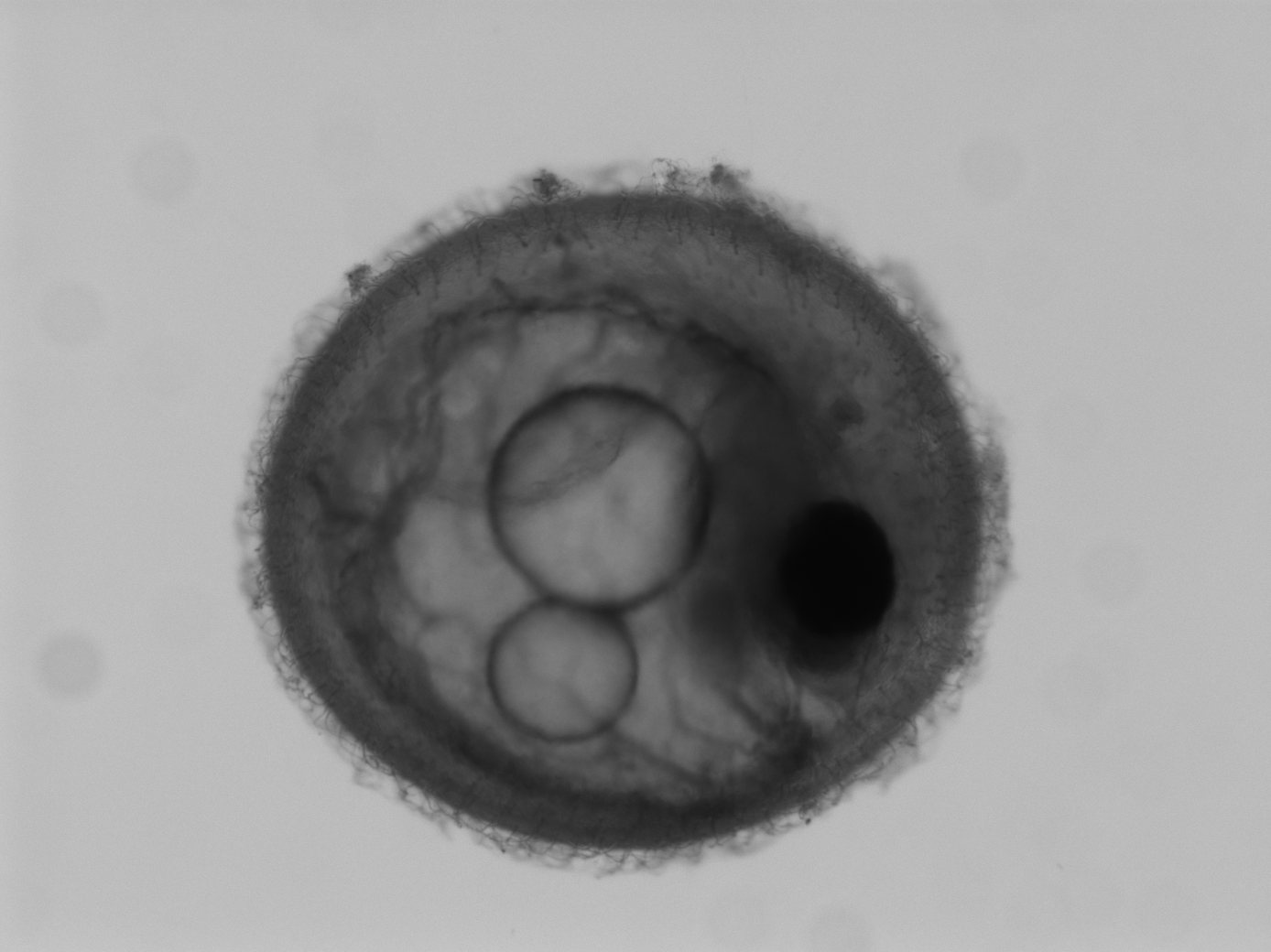

Supplement: Figure 5—source data 1. [file elife-81549-fig5-data1.zip › Figure_5_source_data/Figure_5_panel_CD_source_data/cdkn1aki+_before-Image Export-04/cdkn1aki+_before-Image Export-04_c2.tif]

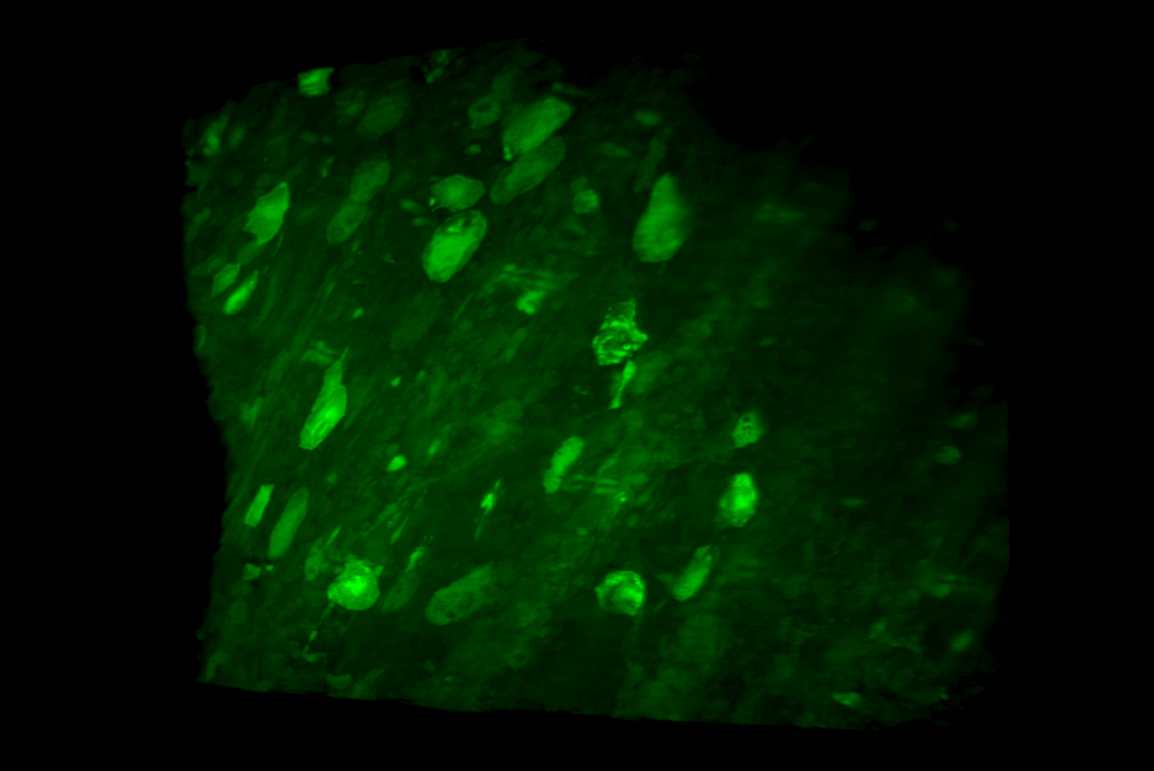

Supplement: Figure 5—source data 1. [file elife-81549-fig5-data1.zip › Figure_5_source_data/Figure_5_panel_JKLM_source_data/17days_ki-ki.tif]

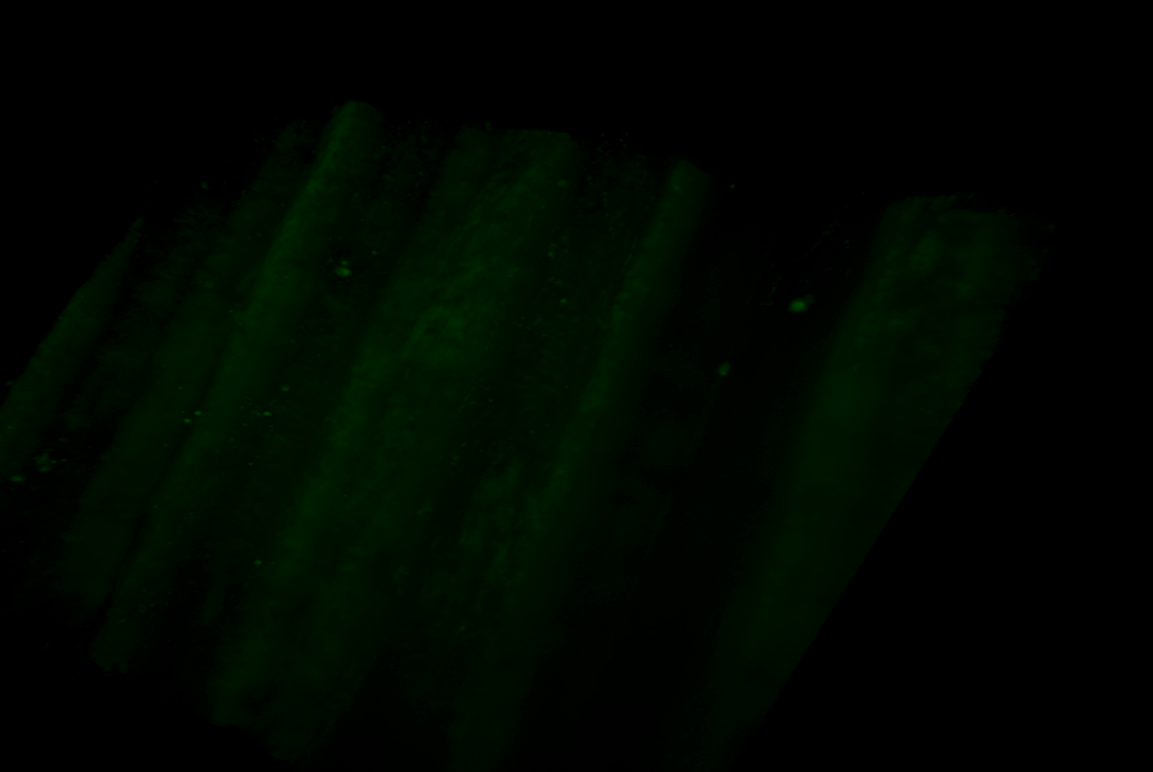

Supplement: Figure 5—source data 1. [file elife-81549-fig5-data1.zip › Figure_5_source_data/Figure_5_panel_JKLM_source_data/17days_wt.tif]

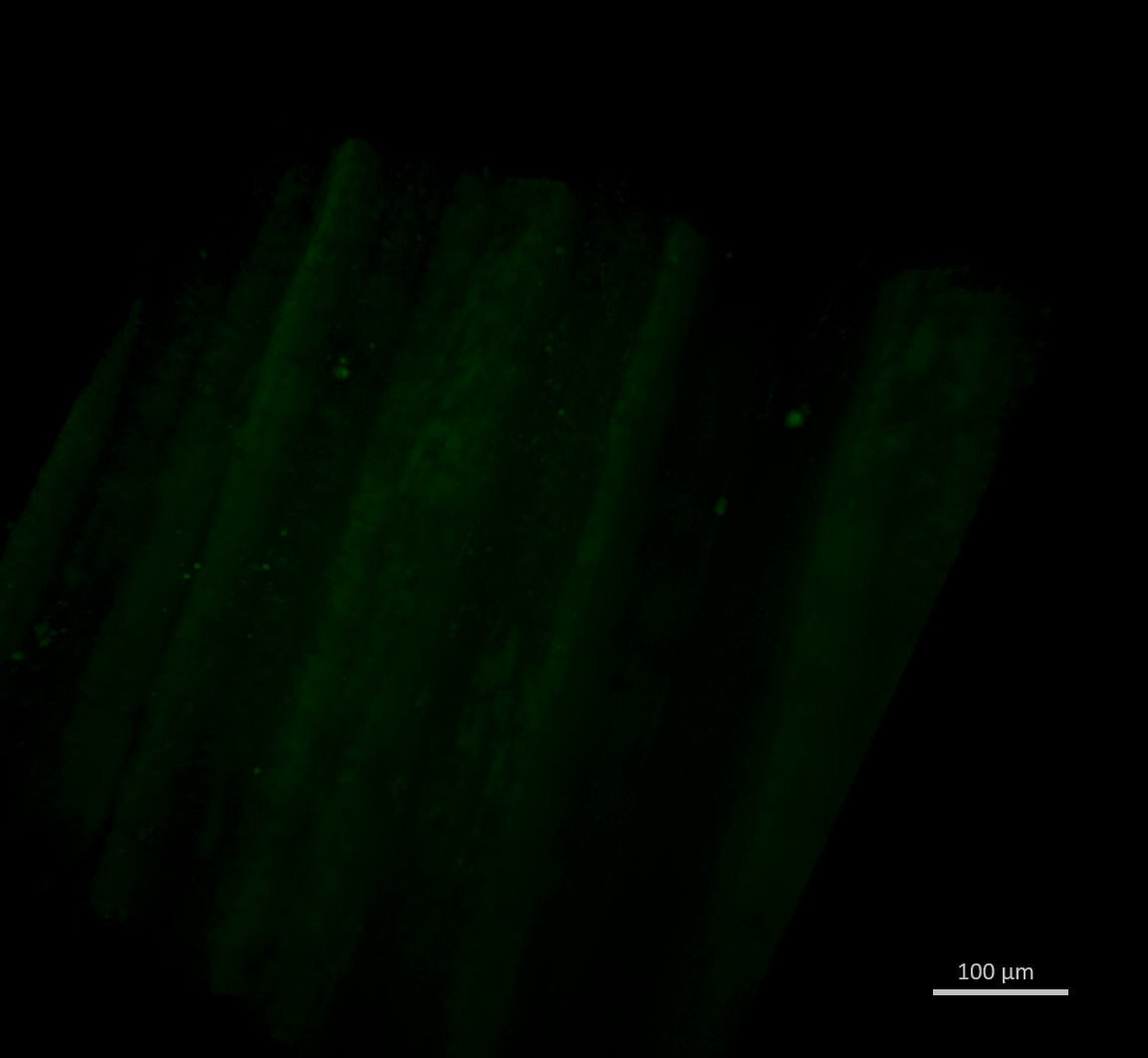

Supplement: Figure 5—source data 1. [file elife-81549-fig5-data1.zip › Figure_5_source_data/Figure_5_panel_JKLM_source_data/17days_wt_withScale.tif]

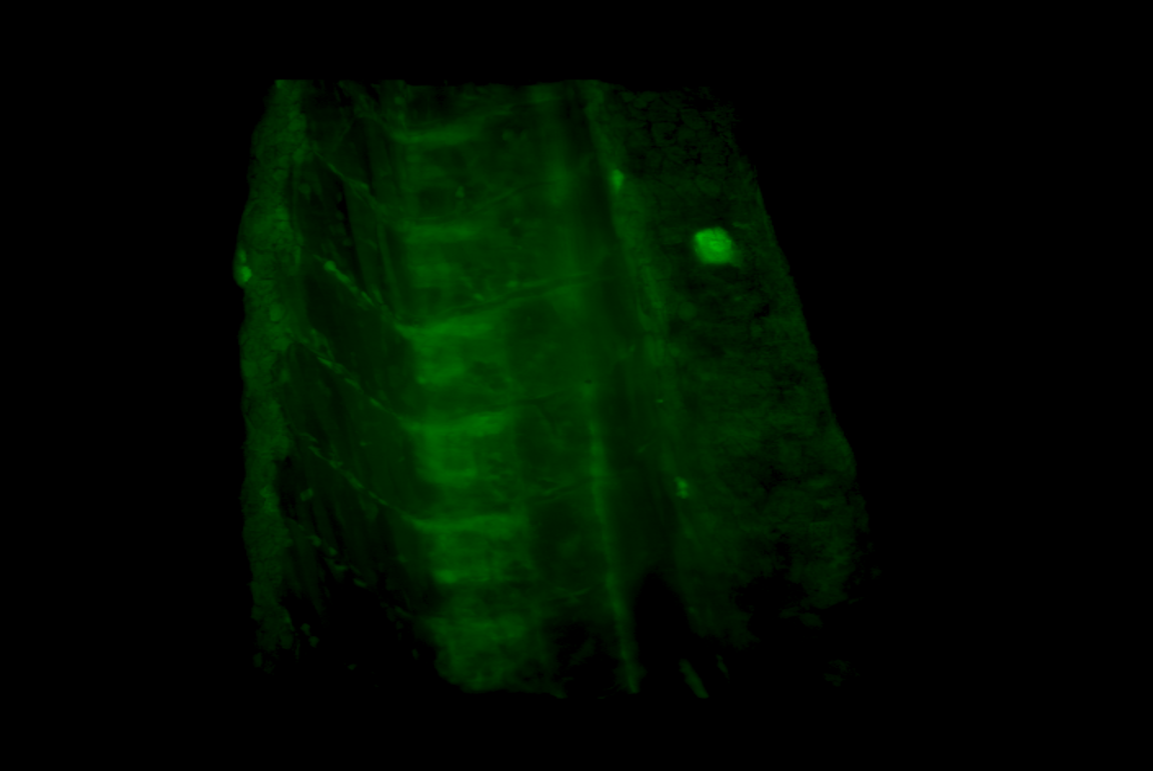

Supplement: Figure 5—source data 1. [file elife-81549-fig5-data1.zip › Figure_5_source_data/Figure_5_panel_JKLM_source_data/4days_ki-ki.tif]

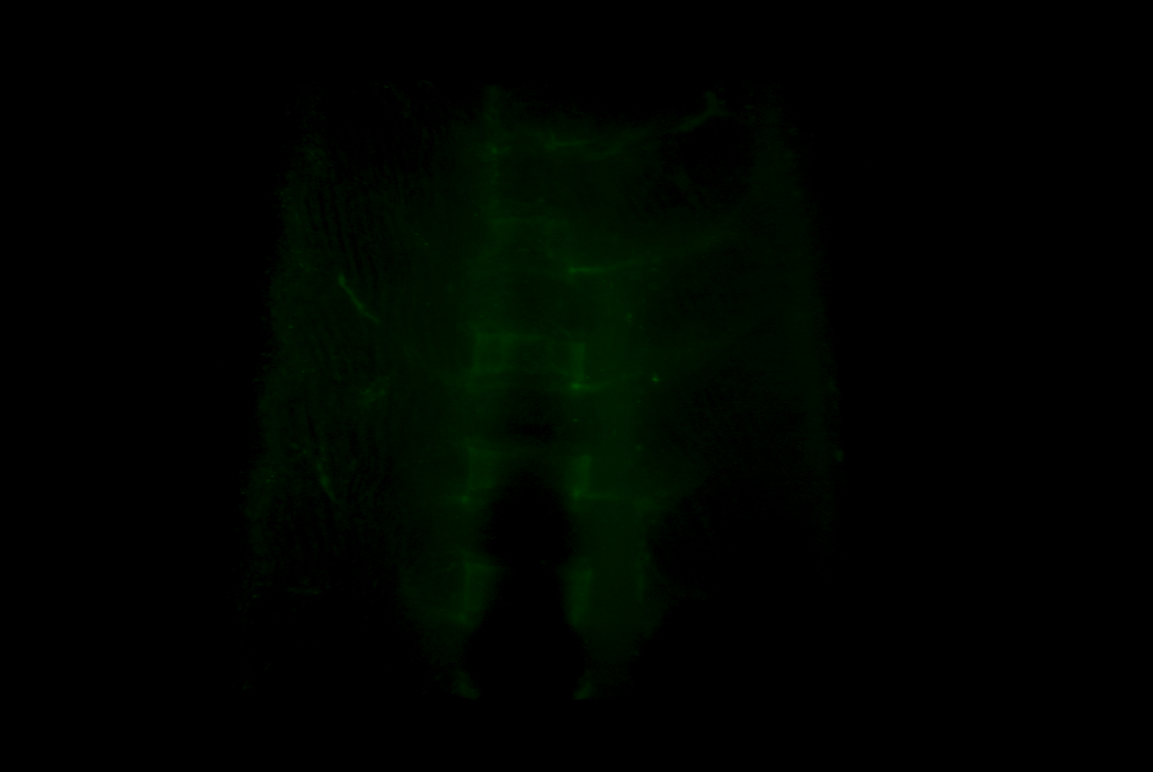

Supplement: Figure 5—source data 1. [file elife-81549-fig5-data1.zip › Figure_5_source_data/Figure_5_panel_JKLM_source_data/4days_wt.tif]

## Supplement Figure 5

panel a

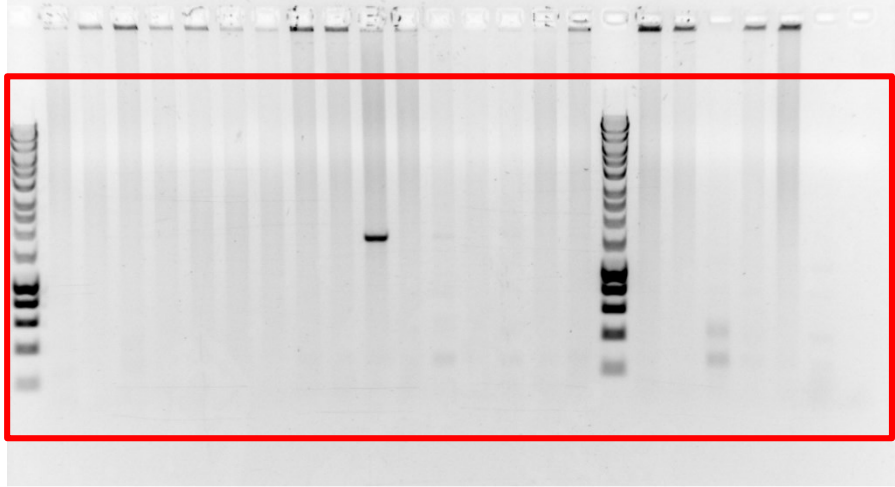

The red box surrounds the lanes shown in the respective panel.

Supplement: Figure 5—figure supplement 1—source data 1. [file elife-81549-fig5-figsupp1-data1.zip › Figure_5_figure_supplement_1_source_data/Figure_5_figure_supplement_1_panel_A_source_data/Figure_5_figure_supplement_1_panel_a_source_data.pdf]

## Slide 1
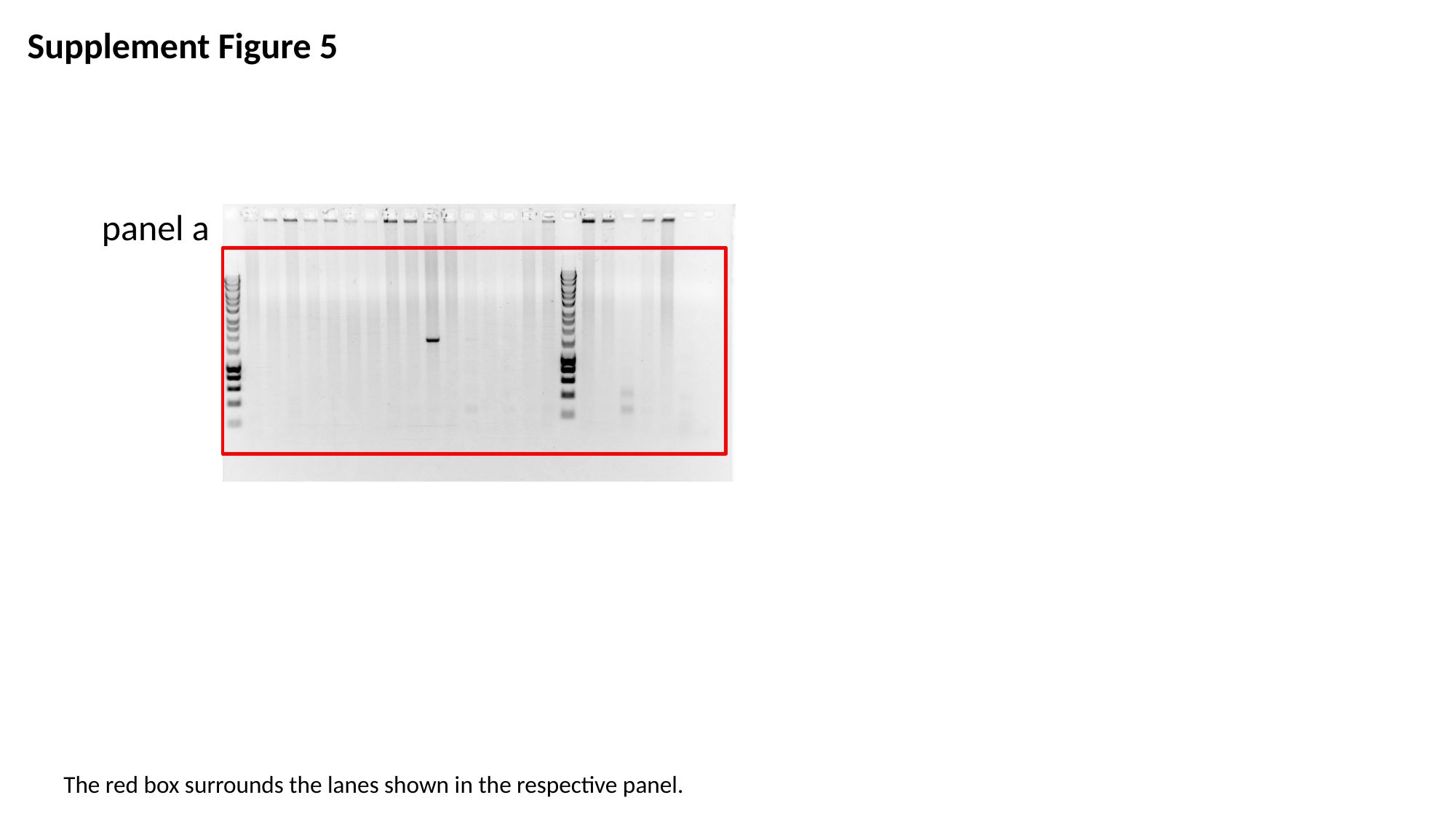

Supplement Figure 5
panel a
The red box surrounds the lanes shown in the respective panel.

Supplement: Figure 5—figure supplement 1—source data 1. [file elife-81549-fig5-figsupp1-data1.zip › Figure_5_figure_supplement_1_source_data/Figure_5_figure_supplement_1_panel_A_source_data/Figure_5_figure_supplement_1_panel_a_source_data.pptx]

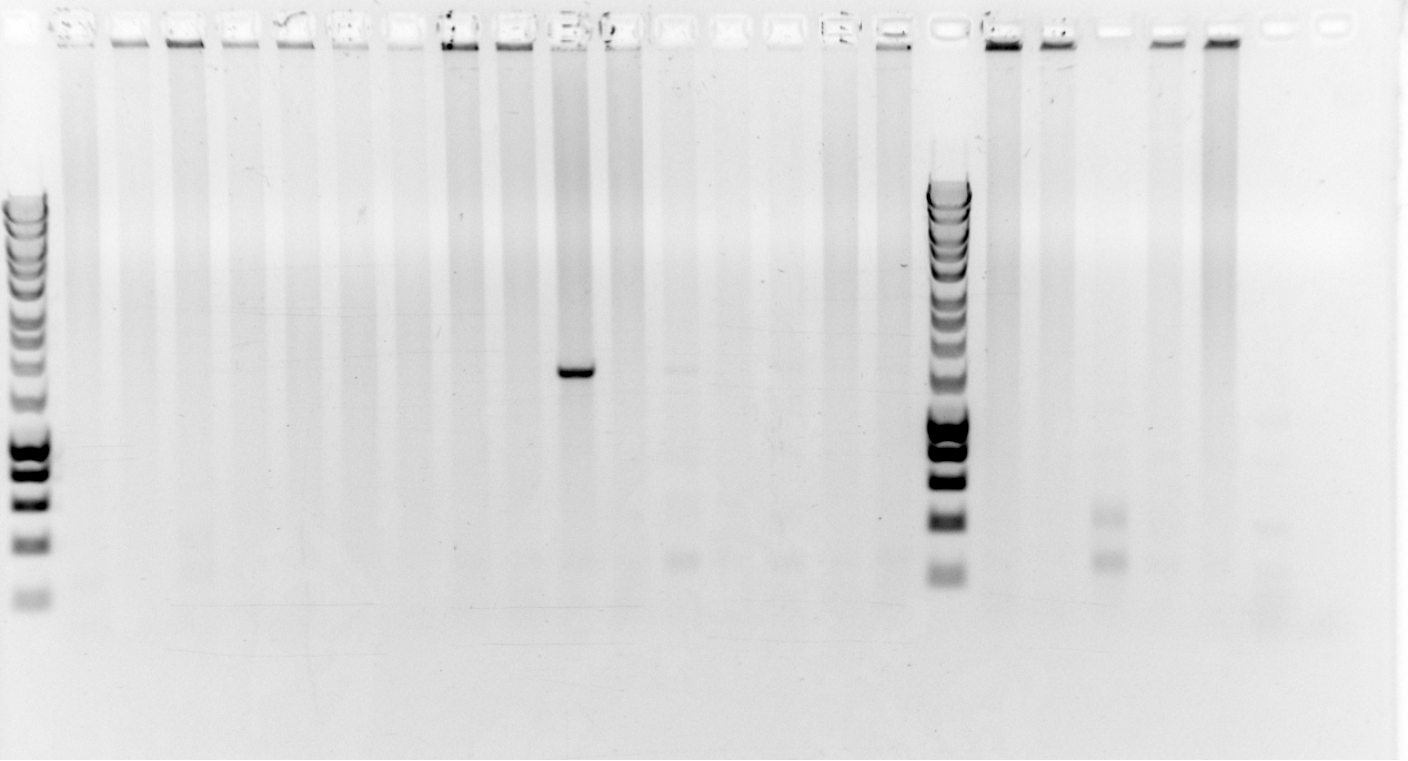

Supplement: Figure 5—figure supplement 1—source data 1. [file elife-81549-fig5-figsupp1-data1.zip › Figure_5_figure_supplement_1_source_data/Figure_5_figure_supplement_1_panel_A_source_data/Figure_5_figure_supplement_1_panel_a_source_data.tif]
