# Supplementary material for: Generation of a transparent killifish line through multiplex CRISPR/Cas9mediated gene inactivation
Source: eLife. 2023 Feb 23;12:e81549. doi: 10.7554/eLife.81549 (PMC10010688; doi:10.7554/eLife.81549)
Supplement: Figure 1—figure supplement 1—source data 1. [file elife-81549-fig1-figsupp1-data1.zip › Figure_1_figure_supplement_1_source_data/Figure_1_figure_supplement_1_panel_G_source_data/Embryohalter_Fischeier_Gezahnt..pdf]

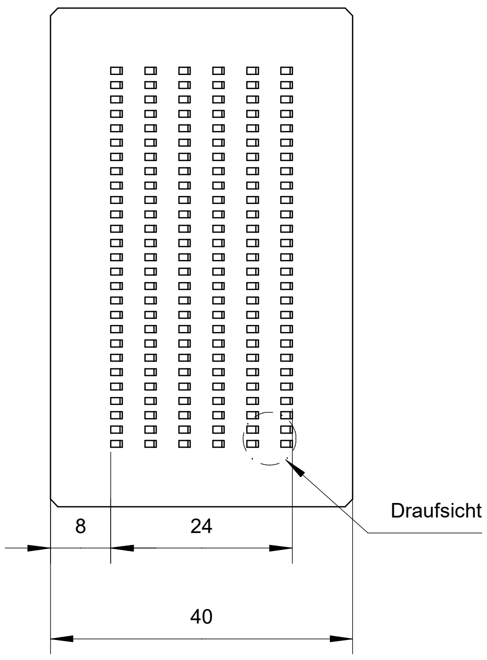

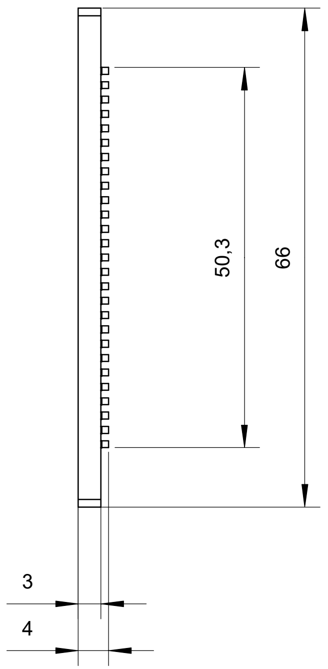

Draufsicht

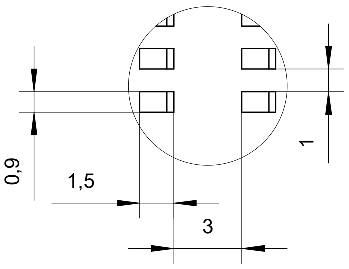

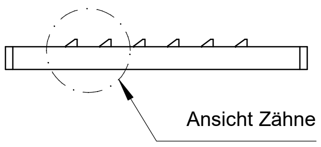

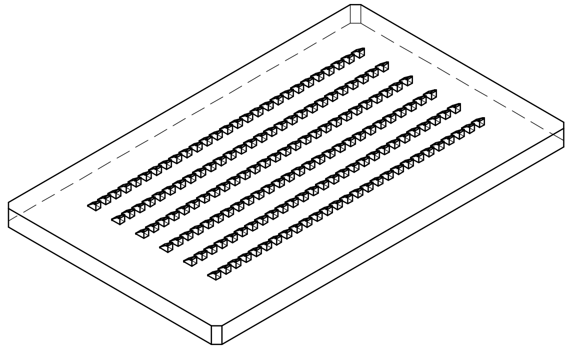

Ansicht Zähne

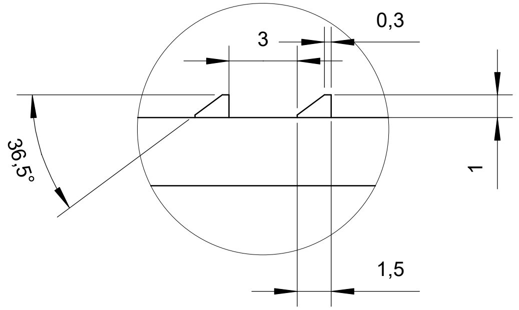

GT - Labortechnik

Gunther Tietsch
Am Artfeld 6
97450 Arnstein - Reuchelheim

Tel.: 09363 - 994754
Fax: 09363 - 995336
Mail: werkstatt@gt-labortechnik.de
Web: www.gt-labortechnik.de

Material: PSU Natur

TITEL

Embryohalter_Fischeier_Gezahnt

GRÖSSE	DATUM	NR	GEP
A4	20.04.2017		ti

MAßSTAB		BLATT
		1
