# Supplementary material for: Generation of a transparent killifish line through multiplex CRISPR/Cas9mediated gene inactivation
Source: eLife. 2023 Feb 23;12:e81549. doi: 10.7554/eLife.81549 (PMC10010688; doi:10.7554/eLife.81549)
Supplement: Figure 1—figure supplement 2—source data 1. [file elife-81549-fig1-figsupp2-data1.zip › Figure_1_figure_supplement_2_source_data/Figure_1_figure_supplement_2_panel_ABC_source_data/Figure_1_figure_supplement_1_panel_abc_source_data.pdf]

Supplement Figure 1

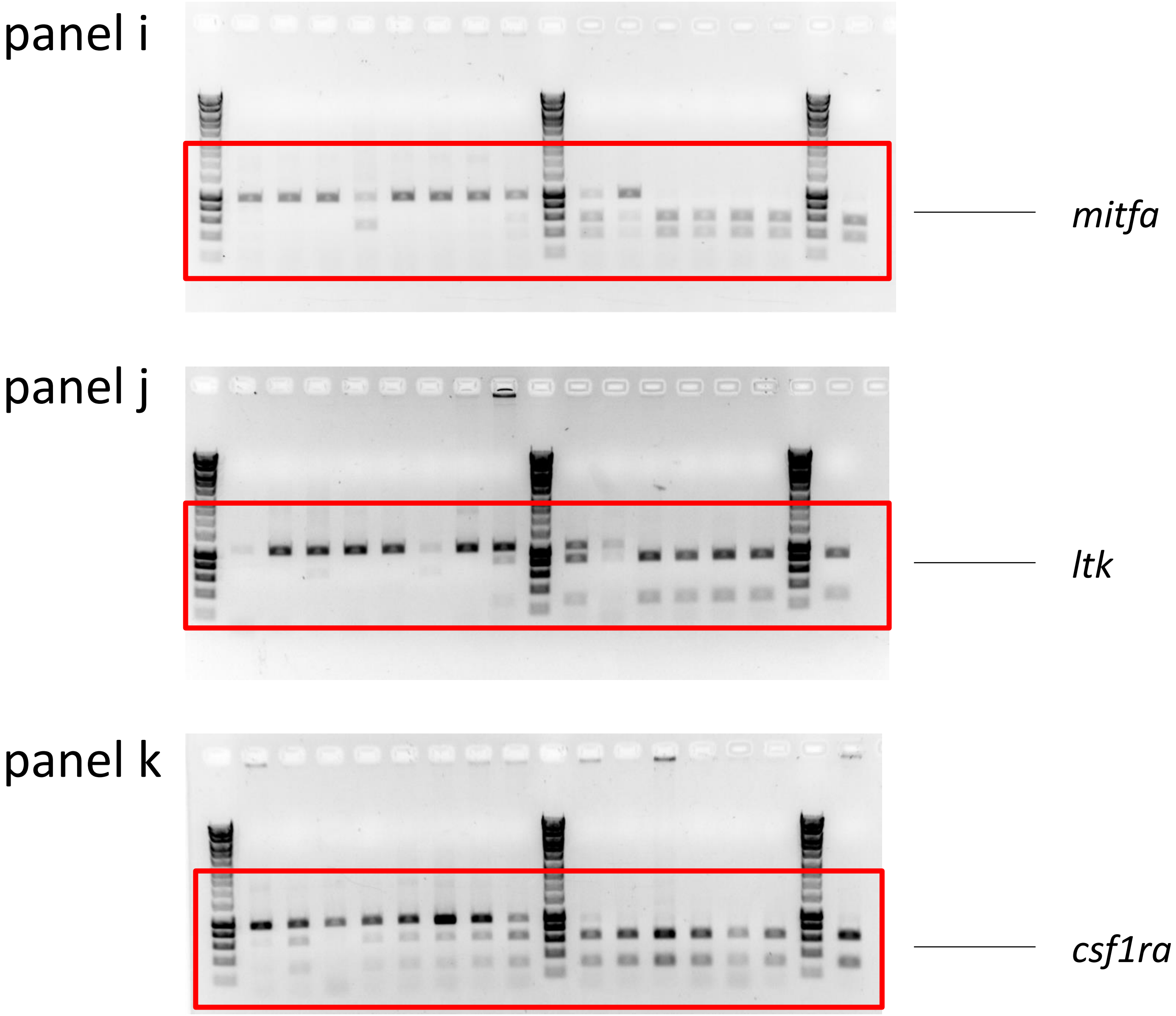

The red box surrounds the lanes shown in the respective panel.
