# Supplementary material for: Generation of a transparent killifish line through multiplex CRISPR/Cas9mediated gene inactivation
Source: eLife. 2023 Feb 23;12:e81549. doi: 10.7554/eLife.81549 (PMC10010688; doi:10.7554/eLife.81549)

Clip. 1 BQ 20 WL 10 Sequence: ltk #1a_M13uni-21

Clipped length:	918	Samples:	12798	Quality: 0 - 9		
Left clip:	80	Bases:	1068	10 - 19		26.02.2018
Right clip:	997	Average spacing:	12.0	20 - 29		
Avg. qual. in clip.:	51.92	Average quality >=	10: 39, 20: 47, 30: 953	>= 30		

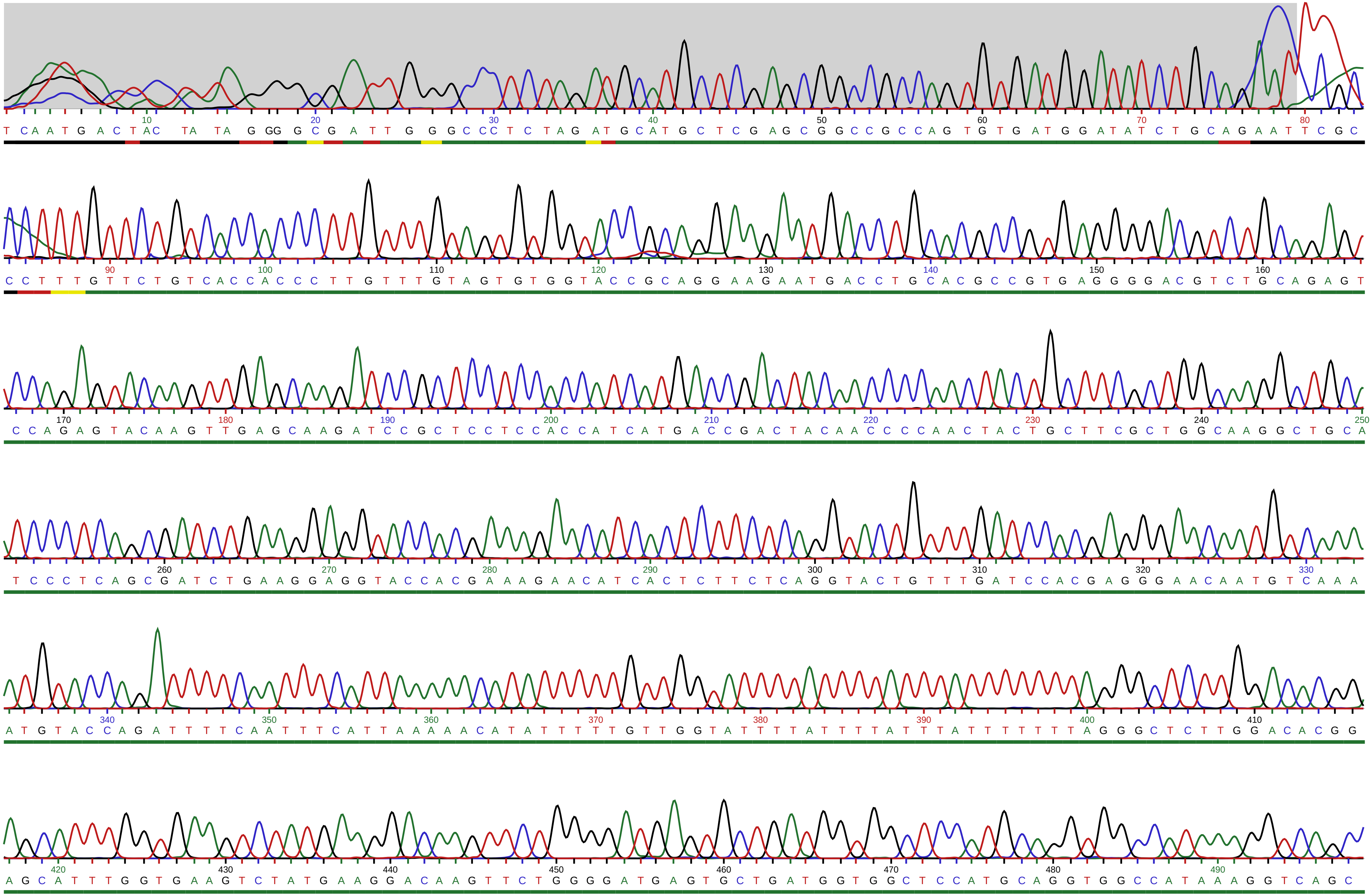

Clip. 1 BQ 20 WL 10 Sequence: ltk #1a_M13uni-21

Clipped length:	918	Samples:	12798	Quality: 0 - 9	
Left clip:	80	Bases:	1068	10 - 19	
Right clip:	997	Average spacing:	12.0	20 - 29	
Avg. qual. in clip.:	51.92	Average quality >=	10: 39, 20: 47, 30: 953	>= 30	

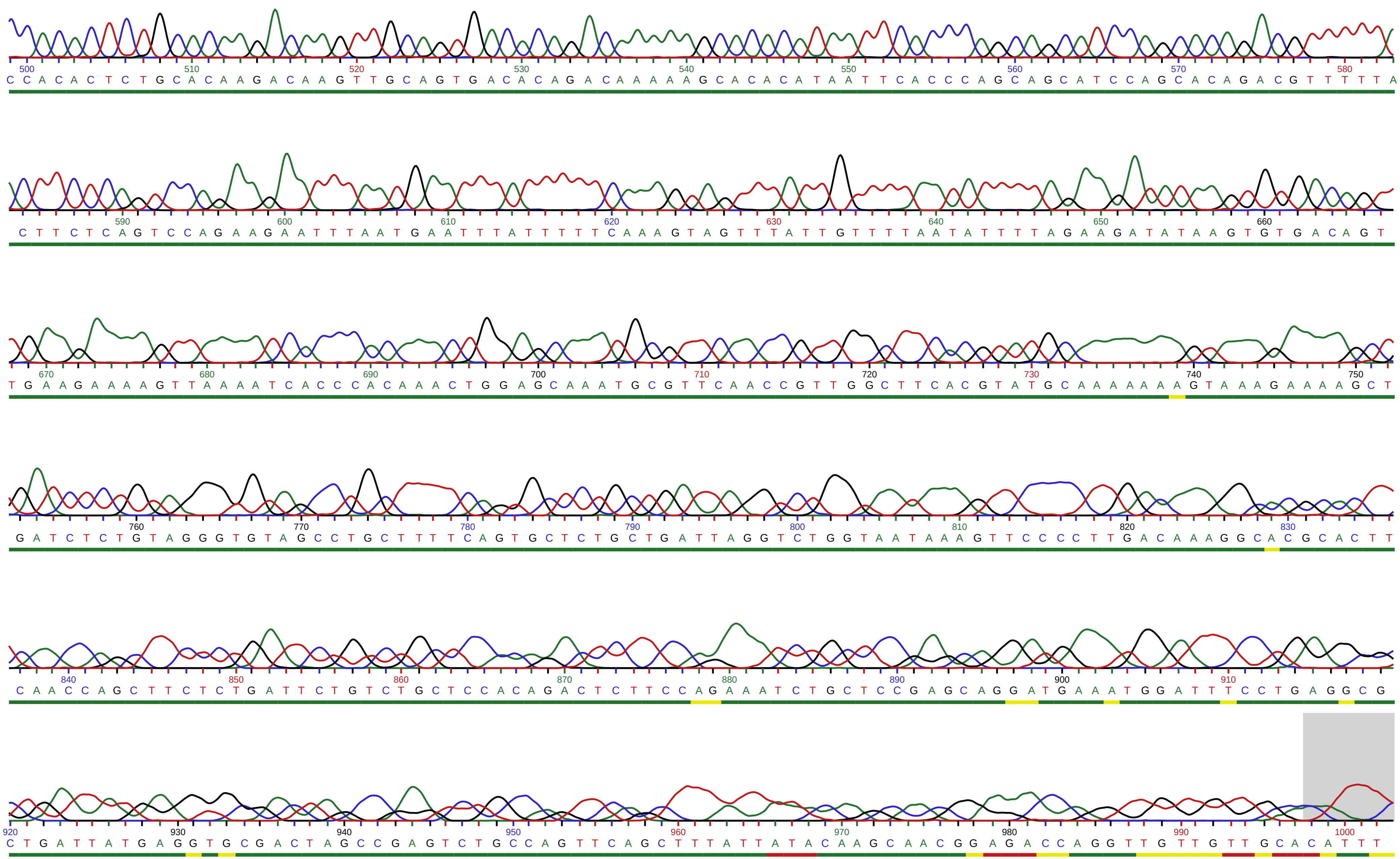

Clip. 1 BQ 20 WL 10 Sequence: ltk #1a_M13uni-21

Clipped length:	918	Samples:	12798	Quality:	0 - 9	
Left clip:	80	Bases:	1068		10 - 19	
Right clip:	997	Average spacing:	12.0		20 - 29	
Avg. qual. in clip.:	51.92	Average quality >=	10: 39, 20: 47, 30: 953		>= 30	

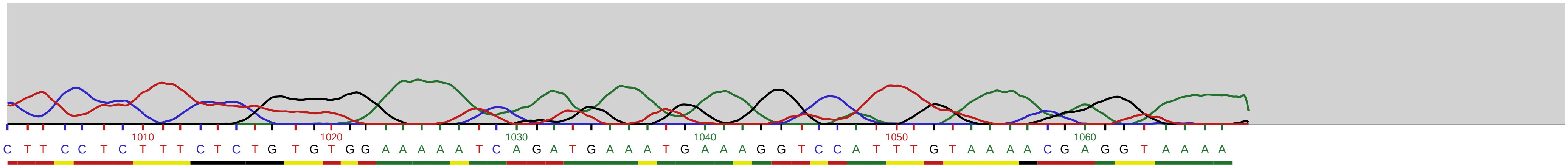

Supplement: Figure 1—figure supplement 3—source data 1. [file elife-81549-fig1-figsupp3-data1.zip › Figure_1_figure_supplement_3_source_data/Figure_1_figure_supplement_3_panel_ABC_source_data/Originals_F1_sequencing/Fish_1/ltk/ltk #1a_M13uni-21.pdf]
