# Supplementary material for: Generation of a transparent killifish line through multiplex CRISPR/Cas9mediated gene inactivation
Source: eLife. 2023 Feb 23;12:e81549. doi: 10.7554/eLife.81549 (PMC10010688; doi:10.7554/eLife.81549)

Clip. 1 BQ 20 WL 10 Sequence: mitfa #11a_M13uni-21

Clipped length:	933	Samples:	13106	Quality:	0 - 9	
Left clip:	8	Bases:	1084		10 - 19	
Right clip:	940	Average spacing:	13.0		20 - 29	
Avg. qual. in clip.:	52.81	Average quality >=	10: 50, 20: 60, 30: 918		>= 30	

26.02.2018

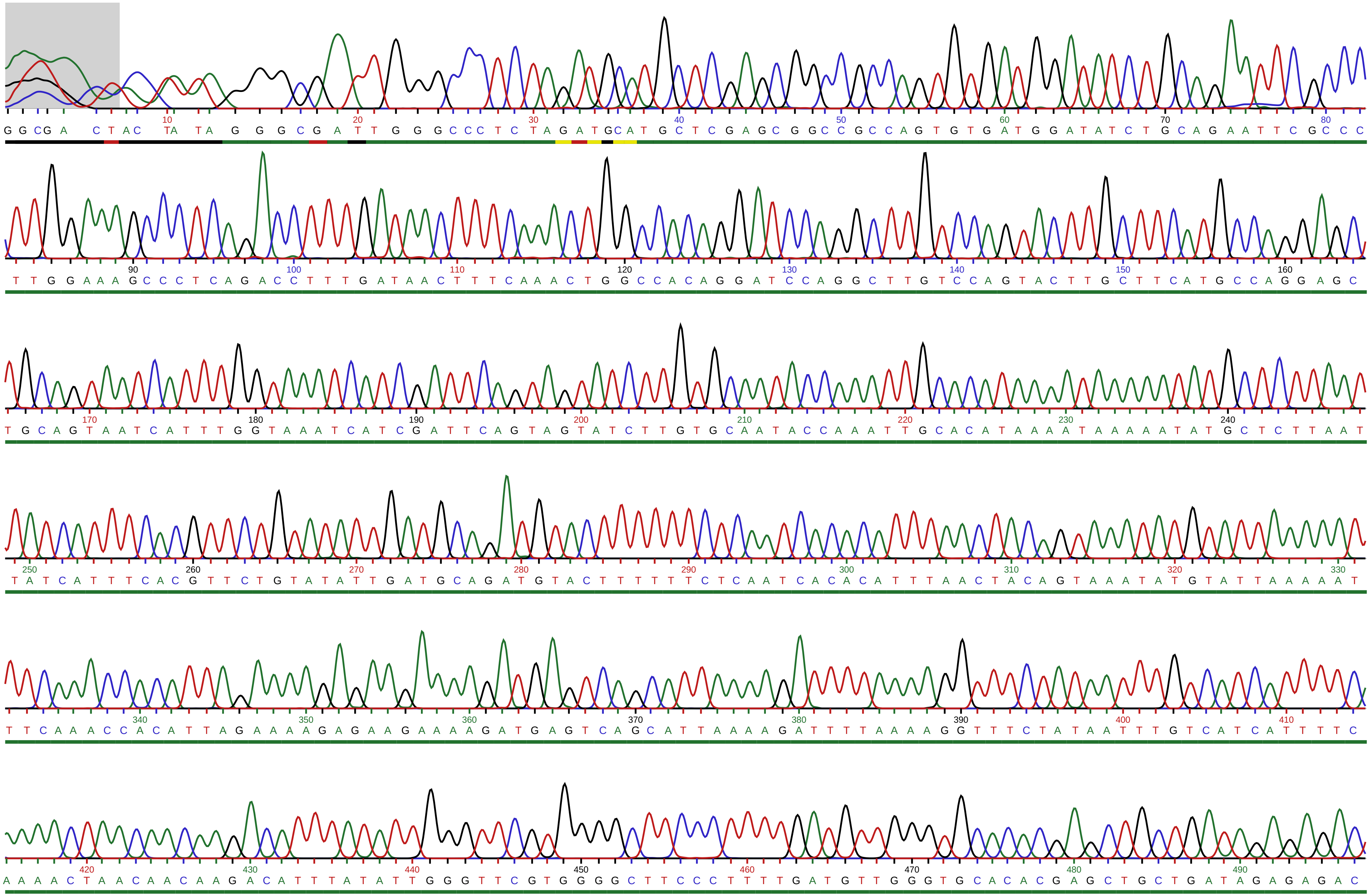

Clip. 1 BQ 20 WL 10

Sequence: mitfa #11a_M13uni-21

Clipped length:	933	Samples:	13106	Quality: 0 - 9	
Left clip:	8	Bases:	1084	10 - 19	
Right clip:	940	Average spacing:	13.0	20 - 29	
Avg. qual. in clip.:	52.81	Average quality >=	10: 50, 20: 60, 30: 918	>= 30	

26.02.2018

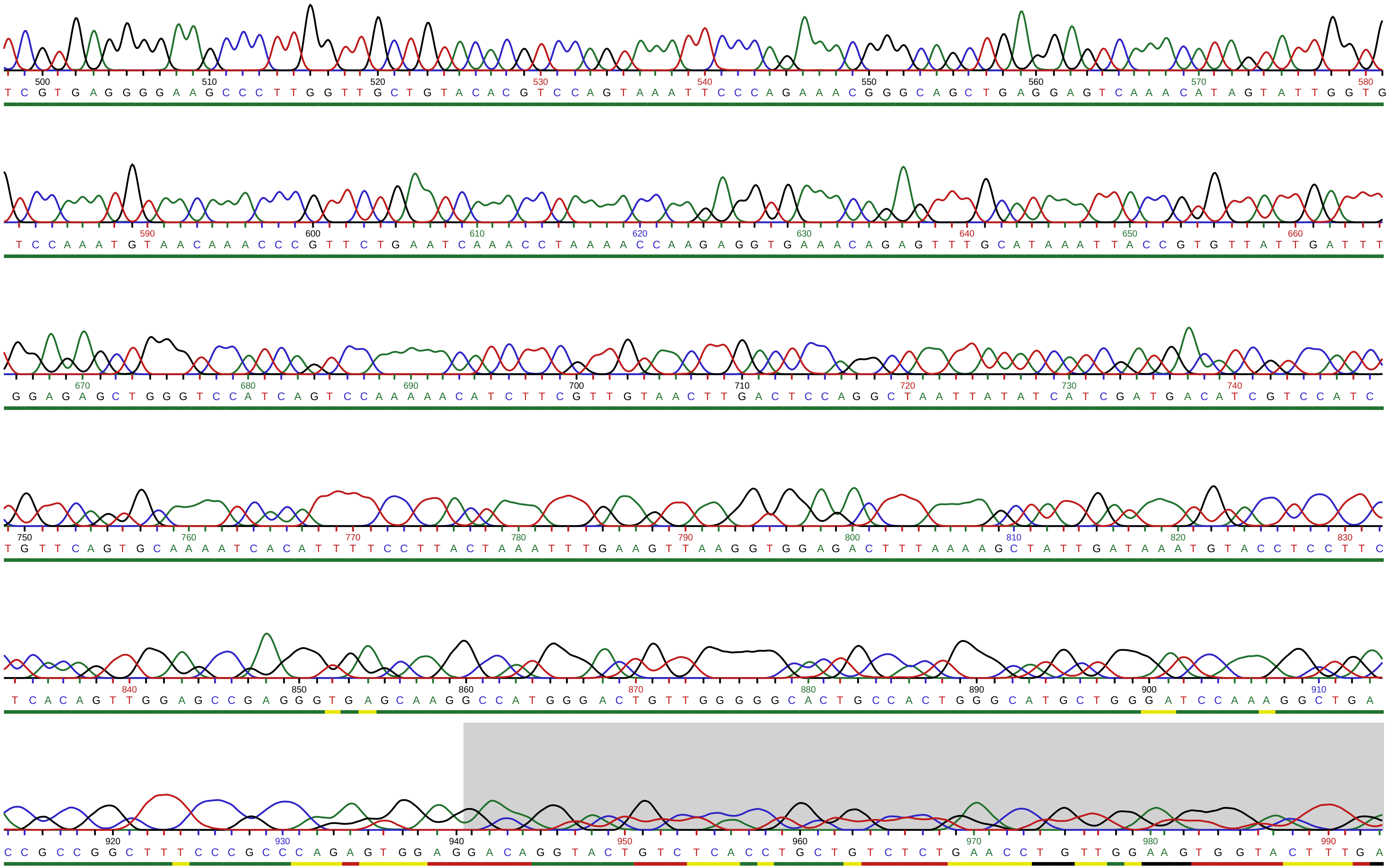

Clip. 1 BQ 20 WL 10 Sequence: mitfa #11a_M13uni-21

Clipped length: 933
Left clip: 8
Right clip: 940
Avg. qual. in clip.: 52.81

Samples: 13106
Bases: 1084
Average spacing: 13.0
Average quality >= 10: 50, 20: 60, 30: 918

Quality: 0 - 9
10 - 19
20 - 29
>= 30

26.02.2018

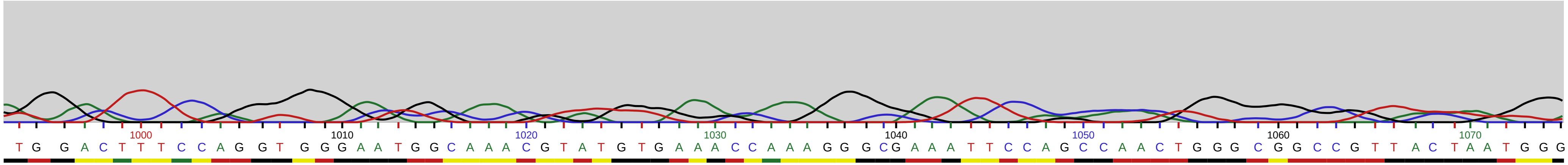

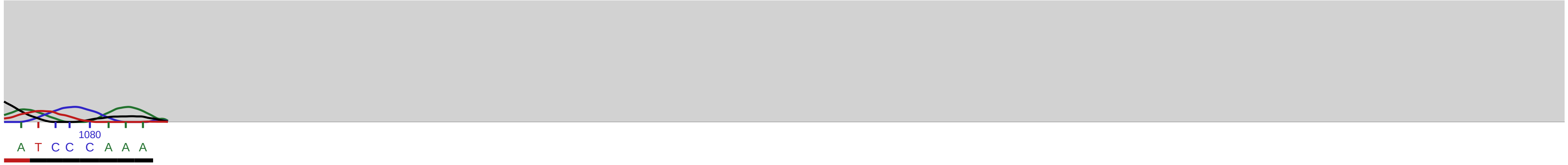

Supplement: Figure 1—figure supplement 3—source data 1. [file elife-81549-fig1-figsupp3-data1.zip › Figure_1_figure_supplement_3_source_data/Figure_1_figure_supplement_3_panel_ABC_source_data/Originals_F1_sequencing/Fish_11/mitfa/mitfa #11a_M13uni-21.pdf]
