# Supplementary material for: Generation of a transparent killifish line through multiplex CRISPR/Cas9mediated gene inactivation
Source: eLife. 2023 Feb 23;12:e81549. doi: 10.7554/eLife.81549 (PMC10010688; doi:10.7554/eLife.81549)
Supplement: Figure 1—figure supplement 3—source data 1. [file elife-81549-fig1-figsupp3-data1.zip › Figure_1_figure_supplement_3_source_data/Figure_1_figure_supplement_3_panel_ABC_source_data/Originals_F1_sequencing/Fish_19/mitfa/mitfa #19a_M13uni-21.pdf]

Clip. 1 BQ 20 WL 10 Sequence: mitfa #19a_M13uni-21

Clipped length: 912
Left clip: 11
Right clip: 922
Avg. qual. in clip.: 51.75

Samples: 11859
Bases: 989
Average spacing: 12.0
Average quality >= 10: 10, 20: 51, 30: 901

Quality: 0 - 9; 10 - 19; 20 - 29; >= 30


26.02.2018

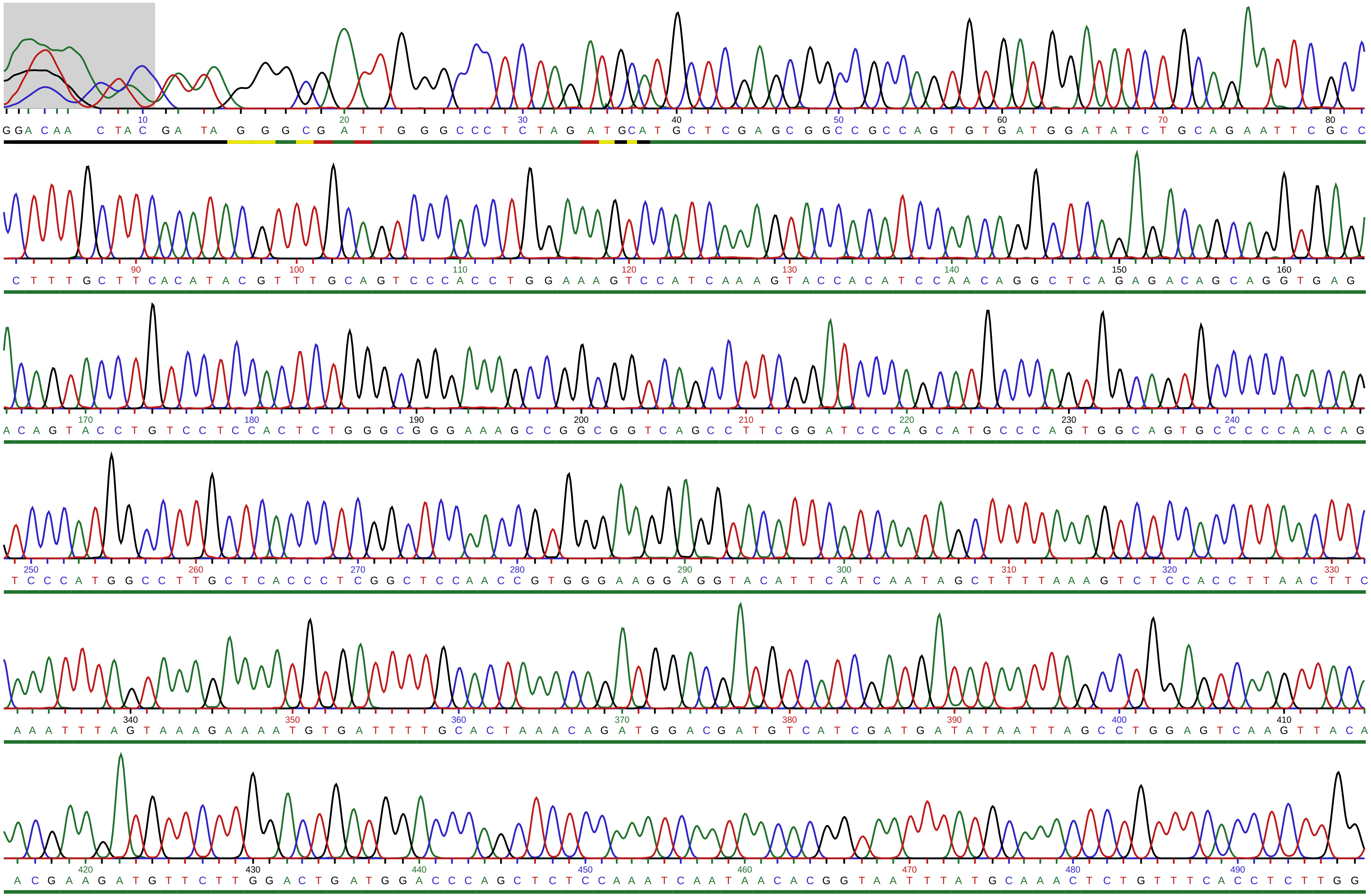

Clip. 1 BQ 20 WL 10 Sequence: mitfa #19a_M13uni-21

Clipped length: 912
Left clip: 11
Right clip: 922
Avg. qual. in clip.: 51.75

Samples: 11859
Bases: 989
Average spacing: 12.0
Average quality >= 10: 10, 20: 51, 30: 901

Quality: 0 - 9; 10 - 19; 20 - 29; >= 30


26.02.2018

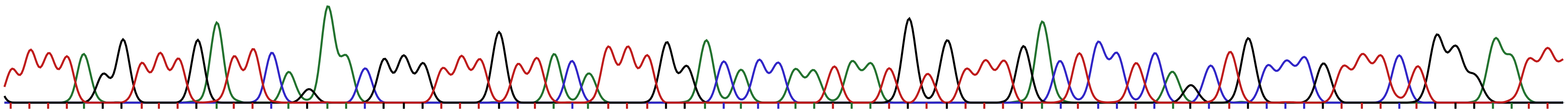

T T T T A G G T T T G A T T C A G A A C G G G T T T G T T A C A T T T G G A C A C C A A T A A T G T G T T T G A C T C C T C A G C T G C C C G T T T C T G G G A A T T

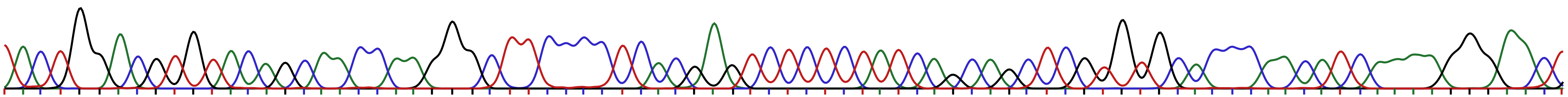

T A C T G G A C G T G T A C A G C A A C C A A G G G C T T C C C C T C A C G A G T C T C T C T A T C A G C A G C T C G T G T G C A C C C A A C A T C A A A A G G G A A C T

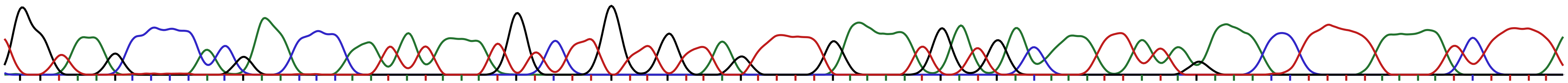

G G T A A G C C C C A C G A A C C C A A T A T A A A T G T C T T G T T G T T A G T T T T G A A A A T G A T G A C A A A T T A T A G A A A C C T T T T A A A A T C T T T T

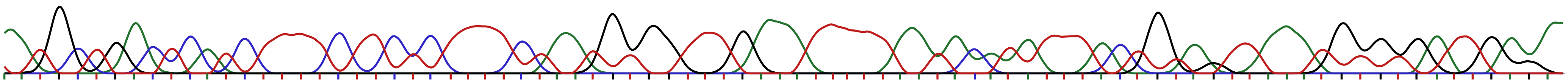

A A T G C T G A C T C A T C T T T T C T T C T C T T T T C T A A T G T G G T T T G A A A T T T T T A A T A C A T A T T T A C T G T A G T T A A A T G T G T G A T T G A G A

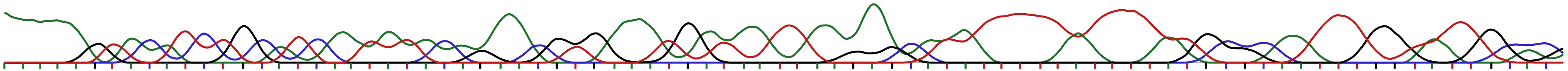

A A A A A G T A C A T C T G C A T C A A T A T A C A G A A C G T G A A A T G A T A A T T A A G A G C A T A T T T T T A T T T T A T G C G C A A T T T G G T A T T G C A C

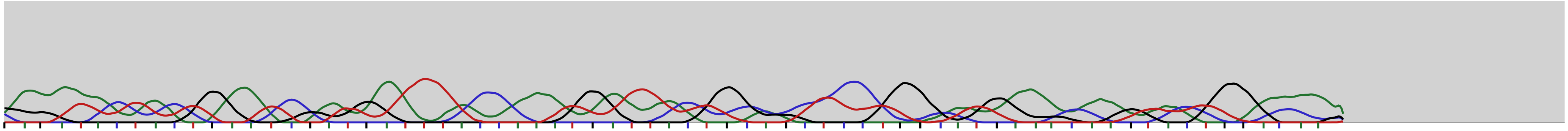

G A G A T A C T A C T G A A T C G A T G A T T T A C C A A A T G A T T A C T G C A G C T C C T G G C A T G A A A C A A G T A C T G G A C A A
