# Supplementary material for: Generation of a transparent killifish line through multiplex CRISPR/Cas9mediated gene inactivation
Source: eLife. 2023 Feb 23;12:e81549. doi: 10.7554/eLife.81549 (PMC10010688; doi:10.7554/eLife.81549)
Supplement: Figure 1—figure supplement 3—source data 1. [file elife-81549-fig1-figsupp3-data1.zip › Figure_1_figure_supplement_3_source_data/Figure_1_figure_supplement_3_panel_ABC_source_data/Originals_F1_sequencing/Fish_29/ltk/ltk #29c_M13uni-21.pdf]

Clip. 1 BQ 20 WL 10

Sequence: ltk #29c_M13uni-21

Clipped length: 971
Left clip: 12
Right clip: 982
Avg. qual. in clip.: 51.6

Samples: 13226
Bases: 1096
Average spacing: 13.0
Average quality >= 10: 44, 20: 69, 30: 934

Quality: 0 - 9, 10 - 19, 20 - 29, >= 30

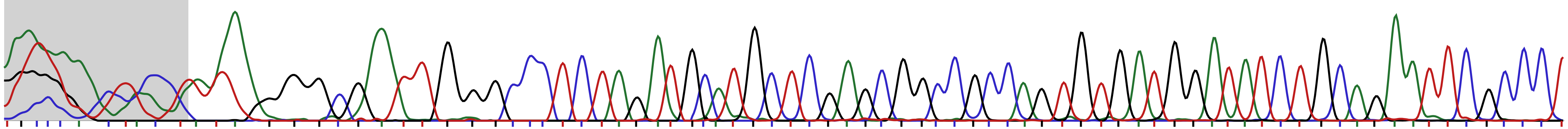

TGCCCACTACTATAGGGCGATTGGGCCCTCTAGATGCATGCTCGAGCGGCCGCCAGTGTGATGGATATCTGCAGAATTCGCCCT (10, 20, 30, 40, 50, 60, 70, 80)

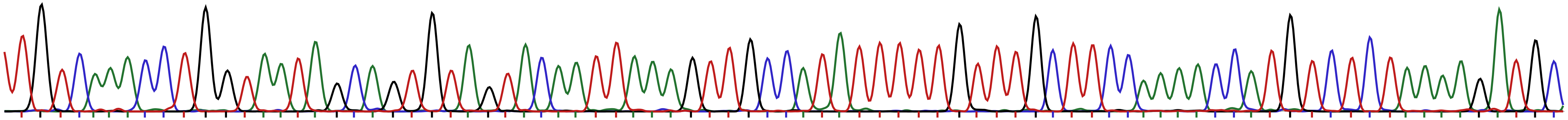

TGTCAAACCTGGTAATAGCAGTGTAGTACAATTAAAGTTGCCATATTTTTGTTTGCTTCCAAAACCATGTCTCTAAAAGATGC (90, 100, 110, 120, 130, 140, 150, 160)

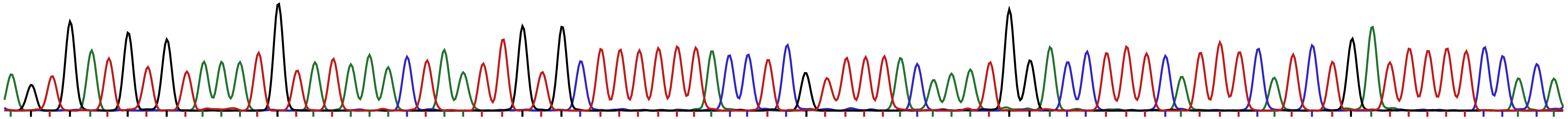

AGTGATGTGTAAATGTATAAACTAATTGTGCTTTTTTACCTCGTTTTACAAATGGACCTTTCATTTCATCTGATTTTTCCACA (170, 180, 190, 200, 210, 220, 230, 240, 250)

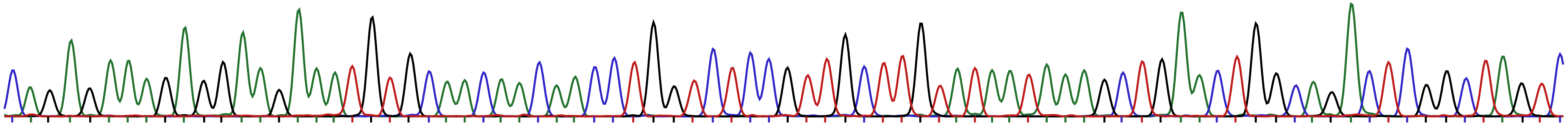

CAGAGAAAGAGGAAGAAATGTGCAACAACAACCTGGTCTCCGTTGCTTGTATAATAAAGCTGAACTGGCAGACTCGGCTAGTC (260, 270, 280, 290, 300, 310, 320, 330)

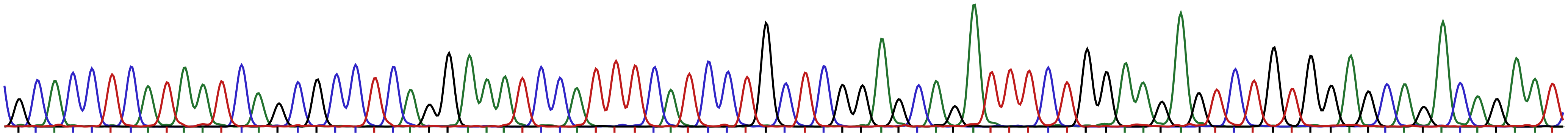

GCACCTCATAATCAGCGCCTCAGGAAATCCATTTCATCCTGCTCGGAGCAGATTTCTGGAAGAGTCTGTGGAGCAGACAGAAT (340, 350, 360, 370, 380, 390, 400, 410)

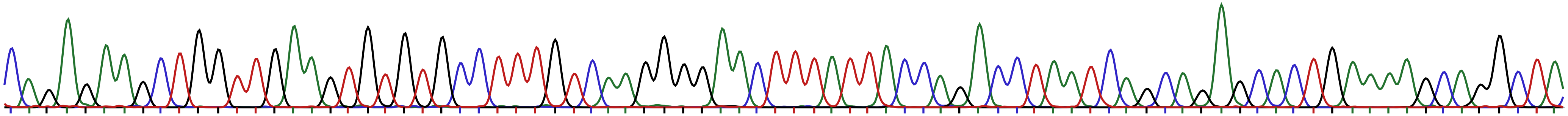

CAGAGAAGCTGGTTGAAGTGTGTGCCTTTGTCAAGGGGAACTTTATTACCAGACCTAATCAGCAGAGCACTGAAAAGCAGGCTA (420, 430, 440, 450, 460, 470, 480, 490, 500)

Clip. 1 BQ 20 WL 10 Sequence: ltk #29c_M13uni-21

Clipped length: 971
Left clip: 12
Right clip: 982
Avg. qual. in clip.: 51.6

Samples: 13226
Bases: 1096
Average spacing: 13.0
Average quality >= 10: 44, 20: 69, 30: 934

Quality: 0 - 9
10 - 19
20 - 29
>= 30

02.03.2018

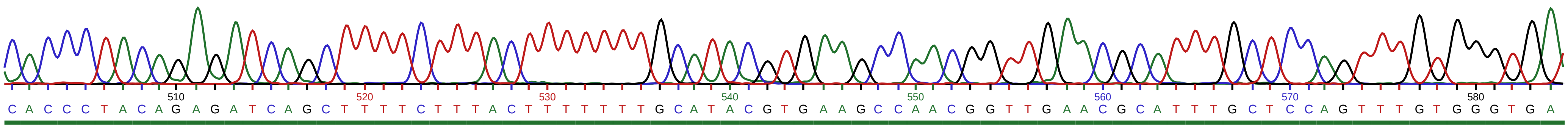

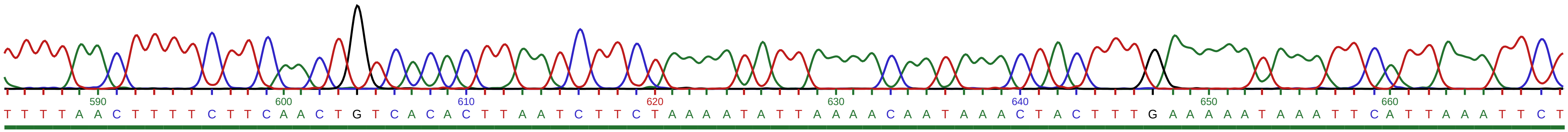

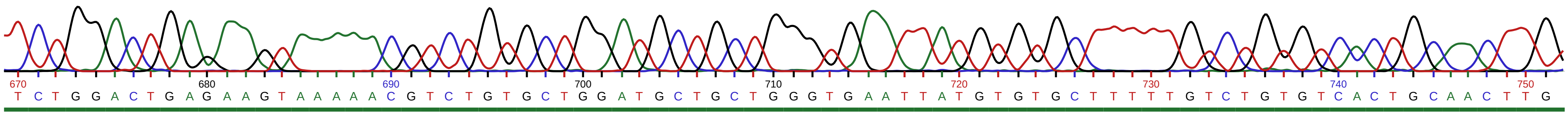

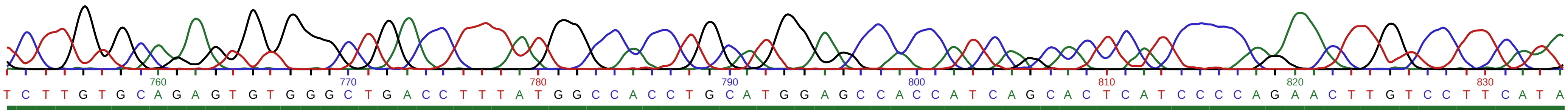

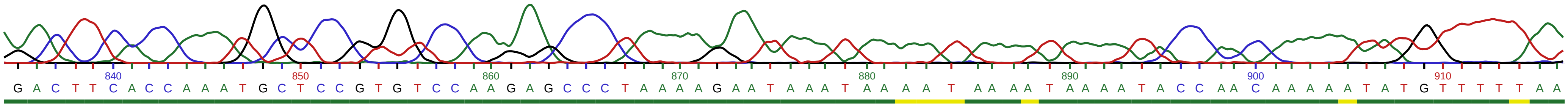

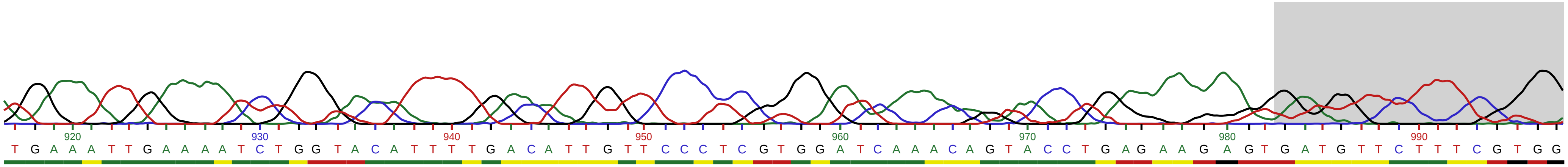

Clip. 1 BQ 20 WL 10 Sequence: ltk #29c_M13uni-21

Clipped length:	971	Samples:	13226	Quality: 0 - 9	
Left clip:	12	Bases:	1096	10 - 19	
Right clip:	982	Average spacing:	13.0	20 - 29	
Avg. qual. in clip.:	51.6	Average quality >=	10: 44, 20: 69, 30: 934	>= 30	

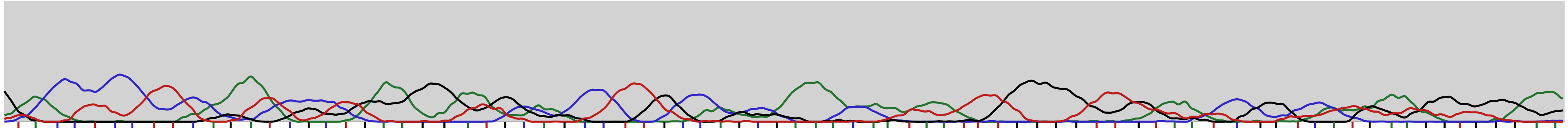

1000 1010 1020 1030 1040 1050 1060 1070

T A C C T C C T T C A G A T C G C T G A A G G A T G C A G C C T T G C C A G C G A A A C A A T A A T T T G G G G T T G T A A T C G G T C A T G A A G G T G G G A A

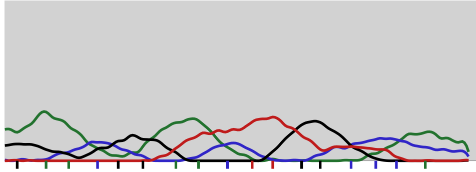

1080 1090

G A A C G G A A C T T G G C C C A
