# Supplementary material for: Generation of a transparent killifish line through multiplex CRISPR/Cas9mediated gene inactivation
Source: eLife. 2023 Feb 23;12:e81549. doi: 10.7554/eLife.81549 (PMC10010688; doi:10.7554/eLife.81549)
Supplement: Figure 1—figure supplement 3—source data 1. [file elife-81549-fig1-figsupp3-data1.zip › Figure_1_figure_supplement_3_source_data/Figure_1_figure_supplement_3_panel_ABC_source_data/Originals_F1_sequencing/Sequencing_F1_fish.docx]

**Sequencing_F1_fish**

*mitfa*

**TGCTTCACATACGTTTGCAG**TCCCACCTGGAAAGTCCAACAAAGTACCACATCCAACAGGCTCAGAGACAGCAGGTGAGACAGTACCTGTCCTCCACTCTGCGGGAAAGCCGGCGGTCAGCCTTCGGATCCCAGCATGCCCAGTGGCAGTGCCCCCAACAGTCCCATGGCCTTGCTCACCCTCGGCTCCAACTGTGAGAAGGAGGTACATTCATCAATAGCTTTTAAAGTCTCCACCTTAACTTCACATTTAGTAAAGAAAATGTGATTTTGCACTGAACAGATGGACGATGTCATCGATGATATAATTAGCCTGGAGTCAAGTTACAACGAAGATGTTCTTGGACTGATGGACCCAGCTCTCCAAATCAATAACACGGTAATTTATGCAAACTCTGTTTCACCTTTTGGTTTTAAGTTTGATTCAAAACGGTTTTGTTACATTTGGACACCAATAATGTGTTTGACTCCTCAGCTGCCCGTTTCTGGGAATTTACTGGACGTGTACAGCAACCAAGGGCTTCCCCTCACGAGTCTCTCTATCAGCAGCTCGTGTGCACCCAACATCAAAAGGGAATTCACTG**GTAAGCCCCATGAACCCAAT**ATAAATGTCTTGTTGTTAGTTTTGAAAATGATGACAAATTATAGAAACCTTTTAAAATCTTTTAATGCTGACTCATCTTTTCTTCTCTTTTCTAATGTGGTTTGAAATTTTTAATACATATTTACTGTAGTTAAATGTGTGATTGAGAAAAAAGTACATCTGCATCAATATACAGAACGTGAAATGATAATTAAGAGCATATTTTTATTTTATGTGCAATTTGGTATTGCACAAGATACTACTGAATCGATGATTTACCAAATGATTACTGCAGCTCCTGGCATGAAGCAAGTACTGGACAAGCCTGGATCCTGTGGCCAGTTTGAAAGTTAT**CAAAGGTCTGAGGGCTTTCC**AGTAGG

JK_mitfa_seq: AGTCTCCACCTTAACTTCACATT

*ltk*

TGTTCTGTCACCACCCTTGTTTGTAGTGTGGTACCGCAGGAAGAATGACCTGCACGCCGTGAGGGGACGTCTGCAGAGTCCAGAGTACAAGTTGAGCAAGATCCGCTCCTCCACCATCATGACCGACTACAACCCCAACTACTGCTTCGCTGGCAAGGCTGCATCCCTCAGCGATCTGAAGGAGGTACCACGAAAGAACATCACTCTTCTCAGGTACTGTTTGATCCACGAGGGAACAATGTCAAAATGTACCAGATTTTCAATTTCATTAAAAACATATTTTTGTTGGTATTTTATTTTATTTTTATTTATTTTTTTAGGGCTCTTGGACACGGAGCATTTGGTGAAGTCTATGAAGGACAAGTTCTGGGGATGAGTGCTGATGGTGGCTCCATGCAGGTGGCCATAAAGGTCAGCCCACACTCTGCACAAGACAAGTTGCAGTGACACAGACAAAAAGCACACATAATTCACCCAGCAGCATCCAGCACAGACGTTTTTACTTCTCAGTCCAGAAGAATTTAATGAATTTATTTTTCAAAGTAGTTTATTGTTTTAATATTTTAGAAGATATAAGTGTGACAGTTGAAGAAAAGTTAAAATCACCCACAAACTGGAGCAAATGCGTTCAACCGTTGGCTTCACGTATGCAAAAAAAGTAAAGAAAAGCTGATCTCTGTAGGGTGTAGCCTGCTTTTCAGTGCTCTGCTGATTAGGTCTGGTAATAAAGTTCCCCTTGACAAAGGCACACACTTCAACCAGCTTCTCTGATTCTGTCTGCTCCACAGACTCTTCCAGAAATCTGCTCCGAGCAGGATGAAATGGATTTCCTGATGGAGGCGCTGATTATGAGGTGCGACTAGCCGAGTCTGCCAGTTCAGCTTTATTATACAAGCAACGGAGACCAGGTTGTTGTTGCACATTTCTTCCTCTTTCTCTGTGTGGAAAAATCAGATGAAATGAAAGGTCCATTTGTAAAACGAGGTAAAAAAGCACAATTAGTTTATACATTTACACATCACTGCATCTTTTAGAGACATGGTTTTGGAAGCAAACAAAAATATGGCAACTTTAATTGTACTACACTGCTATTACCAGGTTTGAC

JK_ltk_seq: AATTCACCCAGCAGCATCCA

*csf1ra*

CATAGATACCGTGCAAGCCTGCAGCTGAAGAGAATGAACATTCAGGAGCAAGGCCAGTACACGTTCTACGCCAGAAGCAACTTGGTCAACGCATCCATCAAGTTTCAAGTTCAAATGTATCGTAAGTGACAGAAATCAAAGCAAGTGTTGCTAATAATATCAACGCTGTAAAACCTGAAAGAGTATGTTTGCTTCTATTTTTTATTGCTATGCAACAGAGAGACCTGTTGCAGTGGTGCGATGGGAGAATGTCACCACTCTGACTTGCACCTCATTCGGCTATCCTGCTCCGAGAATCATCTGGTATCAGTGTCTAGGAATACGGCCCACGTAAGAACCGTTTAATGTAAAATGTAATAAATTGGCCATAAAAATCACCCAAAACATCACAAATTAGGAGCAAATAAGATCTTGTTCCTGTGTTTTGGGGTGATGACACAGGTGCAATGAGAGCACCTCCGGGTTGCAGTTGGCAGTCCCCCTCCAGGCTCTTACTGTGGAGGTCCAGAGGGAGGAGAACGGAGCTGTGGAAGTGGAGAGCGTCCTCAGAGTGGGACCGTCCACCCAGAGGATGACTGTGGAGTGTGTGGCTTTCAATTTGGTTGGCATCAGCAGAGACACTTTTTCCATGGAGGTTTCTGGTGAGTGTTTGTTATTTTGATTGGTTATCATACAGTTGAAATGAGTCAAAGCACCAATGAAGGTTTTTATCTGACTGGACAGATGAACTTTTAACCACCACCCTGAGCGGAGTAGCGGCAATCATGGCAATCCTCCTGGTGCTGCTGGTTTTTCTGCTTTACAAATATAAACAGGTACATTTAAATCCTAACAGCGAATCTAGTACAGGATCACTAGTTCAGTAGCAGGTCTTTTTGATCGCTCTGTTTGATTTTGTTTTTCAGA**AGCCCAGGTATGAAATCCGT**TGGAAGATTATTGAAATGAGTAGTGGAAACAACTACACCTTCATTGACCCCACCCAGCTGCCCTACAACGAGAAGTGGGAGTTCCCCAGAGACAAACTCAAACTAGGTTCAAGCTGCTAAACCCTTATTACAGATCAAACTGTTTCTATGGATTCCATTTGAAATGTCTGCACTCTGAACAGGAAAGATCCTGGGTGCGGGTGCATTCGGAAAAGTGGTTGAAGCAACAGCATATGGTCTTGGGAAAGAGGACAACGCCGTGCGTGTGGCTGTGAAAATGTTAAAAGGTGAAATCTGCTATTTATTACAAATATTTTTTGCCTAAGAAAACCATTCTAAAATATTTAATACCTTAAAGGCCAAGTTCACAGGAAAAC

JK_csf1ra_seq: GTGCGATGGGAGAATGTCAC

**Rev. Complement** of *mitfa* #1b 🡪 -5bp deletion

TCCTCCACTCTGGGCGGGAAAGCCGGCGGTCAGCCTTCGGATCCCAGCATGCCCAGTGGCAGTGCCCCCAACAGTCCCATGGCCTTGCTCACCCTCGGCTCCAACTGTGAGAAGGAGGTACATTCATCAATAGCTTTTAAAGTCTCCACCTTAACTTCAAATTTAGTAAAGAAAATGTGATTTTGCACTAAACAGATGGACGATGTCATCGATGATATAATTAGCCTGGAGTCAAGTTACAACGAAGATGTTCTTGGACTGATGGACCCAGCTCTCCAAATCAATAACACGGTAATTTATGCAAACTCTGTTTCACCTCTTGGTTTTAGGTTTGATTCAGAACGGGTTTGTTACATTTGGACACCAATAATGTGTTTGACTCCTCAGCTGCCCGTTTCTGGGAATTTACTGGACGTGTACAGCAACCAAGGGCTTCCCCTCACGAGTCTCTCTATCAGCAGCTCGTGTGCACCCAACATCAAAAGGGA_____CTGGTAAGCCCCACGAACCCAATATAAATGTCTTGTTGTTAGTTTTGAAAATGATGACAAATTATAGAAACCTTTTAAAATCTTTTAATGCTGACTCATCTTTTCTTCTCTTTTCTAATGTGGTTTGAAATTTTTAATACATATTTACTGTAGTTAAATGTGTGATTGAGAAAAAAGTACATCTGCATCAATATACAGAACGTGAAATGATAATTAAGAGCATATTTTTATTTTATGTGCAATTTGGTATTGCACAAGATACTACTGAATCGATGATTTACCAAATGATTACTGCAGCTCCTGGCATGAAGCAAGTACTGGACAAGCCTGGATCCTGTGGCCAGTTTGAAAGTTATCAAAGGTCTGAGGGCTTTCCAAGGGCGAATTCTGCAGATATCCATCACACTGGCGGCCGCTCGAGCATGCATCTAGAGGGCCCAATCGCCCTAT

*ltk* #1a* 🡪 4bp deletion

TCGCCCTTTGTTCTGTCACCACCCTTGTTTGTAGTGTGGTACCGCAGGAAGAATGACCTGCACGCCGTGAGGGGACGTCTGCAGAGTCCAGAGTACAAGTTGAGCAAGATCCGCTCCTCCACCATCATGACCGACTACAACCCCAACTACTGCTTCGCTGGCAAGGCTGCATCCCTCAGCGATCTGAAGGAGGTACCACGAAAGAACATCACTCTTCTCAGGTACTGTTTGATCCACGAGGGAACAATGTCAAAATGTACCAGATTTTCAATTTCATTAAAAACATATTTTTGTTGGTATTTTATTTTATTTATTTTTTTAGGGCTCTTGGACACGGAGCATTTGGTGAAGTCTATGAAGGACAAGTTCTGGGGATGAGTGCTGATGGTGGCTCCATGCAGGTGGCCATAAAGGTCAGCCCACACTCTGCACAAGACAAGTTGCAGTGACACAGACAAAAAGCACACATAATTCACCCAGCAGCATCCAGCACAGACGTTTTTACTTCTCAGTCCAGAAGAATTTAATGAATTTATTTTTCAAAGTAGTTTATTGTTTTAATATTTTAGAAGATATAAGTGTGACAGTTGAAGAAAAGTTAAAATCACCCACAAACTGGAGCAAATGCGTTCAACCGTTGGCTTCACGTATGCAAAAAAAGTAAAGAAAAGCTGATCTCTGTAGGGTGTAGCCTGCTTTTCAGTGCTCTGCTGATTAGGTCTGGTAATAAAGTTCCCCTTGACAAAGGCACGCACTTCAACCAGCTTCTCTGATTCTGTCTGCTCCACAGACTCTTCCAGAAATCTGCTCCGAGCAGGATGAAATGGATTTCCT(____)GAGGCGCTGATTATGAGGTGCGACTAGCCGAGTCTGCCAGTTCAGCTTTATTATACAAGCAACGGAGACCAGGTTGTTGTTGCA

**Rev. Complement** of *csf1ra* #1c 🡪 5bp deletion

CCCAGTGTGCTGGAATTCGCCCTTCATAGATACCGTGCAAGCCTGCAGCTGAAGAGAATGAACATTCAGGAGCAAGGCCAGTACACGTTCTACGCCAGAAGCAACTTGGTCAACGCATCCATCAAGTTTCAAGTTCAAATGTATCGTAAGTGACAGAAATCAAAGCAAGTGTTGCTATAATATCAACGCTGTAAAACCTGAAAGAGTATGTTTGCTTCTATTTTTTATTGCTATGCAACAGAGAGACCTGTTGCAGTGGTGCGATGGGAGAATGTCACCACTCTGACTTGCACCTCATTCGGCTATCCTGCTCCGAGAATCATCTGGTATCAGTGTCTAGGAATACGGCCCACGTAAGAACCGTTTAATGTAAAATGTAATAAATTGGCCATAAAAATCGCCCAAAACATCACAAATTAGGAGCAAATAAGATCTTGTTCCTGTGTTTTGGGGTGATGACACAGGTGCAATGAGAGCACCTCCGGGTTGCAGTTGGCAGTCCCCCTCCAGGCTCTTACTGTGGAGGTCCAGAGGGAGGAGAACGGAGCTGTGGAAGTGGAGAGCGTCCTCAGAGTGGGACCGTCCACCCAGAGGATGACTGTGGAGTGTGTGGCTTTCAATTTGGTTGGCATCAGCAGAGACACTTTT(_____)GGAGGTTTCTGGTGAGTGTTTGTTATTTTGATTGGTTATCATACAGTTGAAATGAGTCAAAACACCAATGAAGGTTTTTATCTGACTGGACAGATGAACTTTTAACCACCACCCTGAGCGGAGTAGCGGCAATCATGGCAATCCTCCTGGTGCTGCTGGTTTTTCTGCTTTACAAATATAAACAGGTACATTTAAATCCTAACAGCGAATCTAGTACAGGATCACTAGTTCAGTAGCAGGTCTTTTTGATCGCTCTGTTTGATTTTGTTTTTCAGAAGCCCAGGTATGAAATCCGTAAGGGCGAATTCTGCAGATATCCATCACACTGGCGGCCGCTCGAGCATGCATCTAGAGGGCCCAATCGCCCT

**Rev. Complement** of *mitfa* #6c 🡪 11bp deletion

TCCACTCTGAGCGGGAAAGCCGGCGGTCAGCCTTCGGATCCCAGCATGCCCAGTGGCAGTGCCCCCAACAGTCCCATGGCCTTGCTCACCCTCGGCTCCAACTGTGAGAAGGAGGTACATTCATCAATAGCTTTTGAAGTCTCCACCTTAACTTCAAATTTGGTAAAGAAAATGTGATTTTGCACTAAACAGATGGACGATGTCATCGATGATATAATTAGCCTGGAGTCAAGTTACAACGAAGATGTTCTTGGACTGATGGACCCAGCTCTCCAAATCAATAACACGGTAATTTATGCAAACTCTGTTTCACCTCTTGGTCTTAGGTTTGATTCAGAACGGGTTTGTTACATTTGGACACCAATACTATGTTTGACTCCTCAGCTGCCCGTTTCTGGGAATTTACTGGACGTGTACAGCAACCAAGGGCTTCCCTTCACGAGTCTCTCTATCAGCAGCTCGTGTGCACACAACATCAAAAGGGAA(___________)GCCCCACGAACCCAATATAAATGTCTTGTTGTTAGTTTTGAAAATGATGACAAATTATAGAAACCTTTTAAAATCTTTTAATGCTGACTCATCTTTTCTTCTCTTTTCTAATGTGGTTTGAAATTTTTAATACATATTTACTGTAGTTAAATGTGTGATTGAGAAAAAAGTACATCTGCATCAATATACAGAACGTGAAATGATAATTAAGAGCATATTTTTATTTTATGTGCAATCTGGTATTGCACAAGATACTACTGAATCGATGATTTACCAAATGATTACTGCAGCTCCTGGCATGAAGCAAGTACTGGACAAGCCTGGATCCTGTGGCCAGTTTGAAAGTTATCAAAGGTCTGAGGGCTTTCCAAGGGCGAATTCTGCAGATATCCATCACACTGGCGGCCGCTCGAGCATGCATCTAGAGGGCCCAATCGCCCTAT

*ltk* #6a* 🡪 4bp deletion

CTATAGGGCGATTGGGCCCTCTAGATGCATGCTCGAGCGGCCGCCAGTGTGATGGATATCTGCAGAATTCGCCCTTTGTTCTGTCACCACCCTTGTTTGTAGTGTGGTACCGCAGGAAGAATGACCTGCACGCCGTGAGGGGACGTCTGCAGAGTCCAGAGTACAAGTTGGGCAAGATCCGCTCCTCCACCATCATGACCGACTACAACCCCAACTACTGCTTCGCTGGCAAGGCTGCATCCCTCAGCGATCTGAAGGAGGTACCACGAAAGAACATCACTCTTCTCAGGTACTGTTTGATCCACGAGGGAACAATGTCAAAATGTACCAGATTTTCAACTTCATTAAAAACATATTTTTGTTGGTATTTTATTTTATTTATTTTTTTAGGGCTCTTGGACACGGAGCATTTGGTGAAGTCTATGAAGGACAAGTTCTGGGGATGAGTGCTGATGGTGGCTCCATGCAGGTGGCCATAAAGGTCAGCCCACACTCTGCACAAGACAAGTTGCAGTGACACAGACAAAAAGCACACATAATTCACCCAGCAGCATCCAGCACAGACGTTTTTACTTCTCAATCCAGAAGAATTTAATGAATTTATTTTTCAAAGTAGTTTATTGTTTTAATATTTTAGAAGATATAAGTGTGACAGTTGAAGAAAAGTTAAAATCACCCACAAACTGGAGCAAATGCGTTCAACCGTTGGCTTCACGTATGCAAAAAAAGTAAAGAAAAGCTGATCTCTGTAGGGTGTAGCCTGCTTTTCAGTGCTCTGCTGATTAGGTCTGGTAATAAAGTTCCCCTTGACAAAGGCACACACTCCAACCAGCTTCTCTGATTCTGTCTGCTCCACAGACTCTTCCAGAAATCTGCTCCGAGCAGGATGAAATGGATTTCCT(____)GAGGCGCTGATTATGAGGTGCGACTAGCCGAGTCTGCCAGTTCAGCTTTATTATACAAGCAACGGAGACCAGGTTGTTGTTGCACATTTCTTCCTCTTTCTC

**Rev. Complement** of csf1ra #6a 🡪 5bp deletion

GTGTGCTGGAATTCGCCCTTCATAGATACCGTGCAAGCCTGCAGCTGAAGAGAATGAACATTCAGGAGCAAGGCCAGTACACGTTCTACGCCAGAAGCTACTTGGTCAACGCATCCATCAAGTTTCAAGTTCAAATGTATCGTAAGTGACAGAAATCAAAGCAAGTGTTGCTAATAATATCAACGCTGTAAAACCTGAAAGAGTATGTTTGCTTCTATTTTTTATTGCTATGCAACAGAGAGACCTGTTGCAGTGGTGCGATGGGAGAATGTCACCACTCTGACTTGCACCTCATTCGGCTATCCTGCTCCGAGAATCATCTGGTATCAGTGTCTAGGAATACGGCCCACGTAAGAACCGTTTAATGCAAAATGTAATAAATTGGCCATAAAAATCACCCAAAACATCACAAATTAGGAGCAAATAAGATCTTGTTCCTGTGTTTTGGGGTGATGACACAGGTGCAATGAGAGCACCTCCGGGTTGCAGTTGGCAGTCCCCCTCCAGGCTCTTACTGTGGAGGTCCAGAGGGAGGAGAACGGAGCTGTGGAAGTGGAGAGCGTCCTCAGAGTGGGACCGTCCACCCAGAGGATGACTGTGGAGTGTGTGGCTTTCAATTTGGTTGGCATCAGCAGAGACACTTTT(_____)GGAGGTTTCTGGTGAGTGTTTGTTATTTTGATTGGTTATCATACAGTTGAAATGAGTCAAAACACCAATGAAGGTTTTTATCTGACTGGACAGATGAACTTTTAACCACCACCCTGAGCGGAGTAGCGGCAATCATGGCAATCCTCCTGGTGCTGCTGGTTTTTCTGCTTTACAAATATAAACAGGTACATTTAAATCCTAACAGCGAATCTAGTACAGGATCACTAGTTCAGTAGCAGGTCTTTTTGATCGCTCTGTTTGATTTTGTTTTTCAGAAGCCCAGGTATGAAATCCGTAAGGGCGAATTCTGCAGATATCCATCACACTGGCGGCCGCTCGAGCATGCATCTAGAGGGCCCAATCGCCCTATAGT

**Rev. Complement** of *mitfa* #11a 🡪 -11bp

CTCCACTCTGGGCGGGAAAGCCGGCGGTCAGCCTTTGGATCCCAGCATGCCCAGTGGCAGTGCCCCCAACAGTCCCATGGCCTTGCTCACCCTCGGCTCCAACTGTGAGAAGGAGGTACATTTATCAATAGCTTTTAAAGTCTCCACCTTAACTTCAAATTTAGTAAGGAAAATGTGATTTTGCACTGAACAGATGGACGATGTCATCGATGATATAATTAGCCTGGAGTCAAGTTACAACGAAGATGTTTTTGGACTGATGGACCCAGCTCTCCAAATCAATAACACGGTAATTTATGCAAACTCTGTTTCACCTCTTGGTTTTAGGTTTGATTCAGAACGGGTTTGTTACATTTGGACACCAATACTATGTTTGACTCCTCAGCTGCCCGTTTCTGGGAATTTACTGGACGTGTACAGCAACCAAGGGCTTCCCCTCACGAGTCTCTCTATCAGCAGCTCGTGTGCACCCAACATCAAAAGGGAA(___________)GCCCCACGAACCCAATATAAATGTCTTGTTGTTAGTTTTGAAAATGATGACAAATTATAGAAACCTTTTAAAATCTTTTAATGCTGACTCATCTTTTCTTCTCTTTTCTAATGTGGTTTGAAATTTTTAATACATATTTACTGTAGTTAAATGTGTGATTGAGAAAAAAGTACATCTGCATCAATATACAGAACGTGAAATGATAATTAAGAGCATATTTTTATTTTATGTGCAATTTGGTATTGCACAAGATACTACTGAATCGATGATTTACCAAATGATTACTGCAGCTCCTGGCATGAAGCAAGTACTGGACAAGCCTGGATCCTGTGGCCAGTTTGAAAGTTATCAAAGGTCTGAGGGCTTTCCAAGGGCGAATTCTGCAGATATCCATCACACTGGCGGCCGCTCGAGCATGCATCTAGAGGGCCCAATCGCCCTATAGT

*ltk* #11d* 🡪 4bp deletion

ATAGGGCGATTGGGCCCTCTAGATGCATGCTCGAGCGGCCGCCAGTGTGATGGATATCTGCAGAATTCGCCCTTTGTTCTGTCACCACCCTTGTTTGTAGTGTGGTACCGCAGGAAGAATGACCTGCACGCCGTGAGGGGACGTCTGCAGAGTCCAGAGTACAAGTTGAGCAAGATCCGCTCCTCCACCATCATGGCCGACTACAACCCCAACTACTGCTTCGCTGGCAAGGCTGCATCCCTCAGCGATCTGAAGGAGGTACCACGAAAGAACATCACTCTTCTCAGGTACTGTTTGATCCACGAGGGAACAATGTCAAAATGTACCAGATTTTCAATTTCATTAAAAACATATTTTTGTTGGTATTTTATTTTATTTATTTTTTTAGGGCTCTTGGACACGGAGCATTTGGTGAAGTCTATGAAGGACAAGTTCTGGGGATGAGTGCTGATGGTGGCTCCATGCAGGTGGCCATAAAGGTCAGCCCACACTCTGCGCAAGACAAGTTGCAGTGACACAGACAAAAAGCACACATAATTCACCCAGCAGCATCCAGCACAGACGTTTTTACTTCTCAGTCCAGAAGAATTTAATGAATTTATTTTTCAAAGTAGTTTATTGTTTTAATATTTTAGAAGATATAAGTGTGACAGTTGAAGAAAGGTTAAAATCACCCACAAACTGGAGCAAATGCGTTCAACCGTTGGCTTCACGTATGCAAAAAAAGTAAAGAAAAGCTGATCTCTGTAGGGTGTAGCCTGCTTTTCAGTGCTCTGCTGATTAGGTCTGGTAATAAAGTTCCCCTTGACAAAGGCACACACTTCAACCAGCTTCTCTGATTCTGTCTGCTCCACAGACTCTTCCAGAAATCTGCTCCGAGCAGGATGAAATGGATTTCCT(____)GAGGCGCTGATTATGAGGTGCGACTAGCCGAGTCTGCCAGTTCAGCTTTATTATACAAGCAACGGAGACCAGGTTGTTGTTGCACATTTCTTCCTCTTTCTCTGT

*csf1ra*_#11a 🡪 5bp deletion

ATAGGGCGATTGGGCCCTCTAGATGCATGCTCGAGCGGCCGCCAGTGTGATGGATATCTGCAGAATTCGCCCTTACGGATTTCATACCTGGGCTTCTGAAAAACAAAATCAAACAGAGCGATCAAAAAGACCTGCTACTGAACTAGTGATCCTGTACTAGATTCGCTGTTAGGATTTAAATGTACCTGTTTATATTTGTAAAGCAGAAAAACCAGCAGCACCAGGAGGATTGCCATGATTGCCGCTACTCCGCTCAGGGTGGTGGTTAAAAGTTCATCTGTCCAGTCAGATAAAAACCTTCATTGGTGTTTTGACTCATTTCAACTGTATGATAACCAATCAAGATAACAAACACTCACCAGAAACCTCCAAAAGTGTCTCTGCTGATGCCAACCAAATTGAAAGCCACACACTCCACAGTCATCCTCTGGGTGGACGGTCCCACTCTGAGGACGCTCTCCACTTCCACAGCTCCGTTCTCCTCCCTCTGGACCTCCACAATAAGAGCCTGGAGGGGGACTGCCAACTGCAACCCGGAGGTGCTCTCATTGCACCTGTGTCATCACCCCAAAACACAGGAACAAGATCTTATTTGCTCCTAATTTGTGATGTTTTGAGTGATTTTTATGGCCAATTTATTACATTTTACATTAAACGGTTCTTACGTGGGCCGTATTCCTAGACACTGATACCAGATGATTCTCGGAGCAGGATAGCCGAATGAGGTGCAAGTCAGAGTGGTGACATTCTCCCATCGCGCCACTGCAACAGGTCTCTCTGTTGCATAGCAATAAAAAATAGAAGCAAACATACTCTTTCAGGTTTTACAGCGTTGATATTATTAGCAACACTTGCTTTGATTTCTGTCACTTACGATACATTTGAACTTGAAACTTGATGGATGCGTTGACCATGTTGCTTCTGGCGTAGAACGTGTACTGGCCTTGCTCCTGAATGTTCATTCTCTTCAGCTGCAGGCTTGCACGGTATCTATGAAGGGCGAATTCCAGCACACTG

*mitfa* #18d* 🡪 -4bp

TAGGGCGATTGGGCCCTCTAGATGCATGCTCGAGCGGCCGCCAGTGTGATGGATATCTGCAGAATTCGCCCTTTGCTTCACATACGTTTGCAGTCCCACCTGGAAAGTCCATCAAAGTACCACATCCAACAGGCTCAGAGACAGCAGGTGAGACAGTACCTGTCCTCCACTCTGGGCGGGAAAGCCGGCGGTCAGCCTTCGGATCCCAGCATGCCCAGTGGCAGTGCCCCCAACAGTCCCATGGCCTTGCTCACCCTCGGCTCCAACTGTGAGAAGGAGGTACATTCATCAATAGCTTTTAAAGTCTCCACCTTAACTTCAAATTTAGTAAAGAAAATGTGATTTTGCACTAAACAGATGGACGATGTCATCGATGATATAATTAGCCTGGAGTCAAGTTACAACGAAGATGTTCTTGGACTGATGGACCCAGCTCTCCAAATCAATAACACGGTAATTTATGCAAACTCTGTTTCACCTCTTGGTTTTAGGTTTGATTCAGAACGGGTTTGTTACATTTGGACACCAATAATGTGTTTGACTCCTCAGCTGCCCGTTTCTGGGAATTTACTGGACGTGTACAGCAACCAAGGGCTTTCCCTCACGAGTCTCTCTATCAGCAGCTCGTGTGCACCCAACATCAAAAGGGAA(____)CTGGTAAGCCCCACGAACCCAATATAAATGTCTTGTTGTTAGTTTTGAAAATGATGACAAATTATAGAAACCTTTTAAAATCTTTTAATGCCGACTCATCTTTTCTTCTCTTTTCTAATGTGGTTTGAAATTTTTAATACATATTTACTGTAGTTAAATGTGTGATTGAGAAAAAAGTACATCTGCATCAATATACAGAACGTGAAATGATAATTAAGAGCATATTTTTATTTTATGTGCAATTTGGTATTGCACAAGATACTACTGAATCGATGATTTACCAAATGATTACTGCAGCTCCTGGCATGAAGCAAGTACTGGACAAGCC

**Rev. Complement** of *ltk* #18c 🡪 3bp deletion

CTCTTCTCAGGTACTGTTTGATCCACGAGGGAACAATGTCAAAATGTACCAGATTTTCAATTTCATTAAAAACATATTTTTGTTGGTATTTTATTTTATTTATTTTTTTAGGGCTCTTGGACACGGAGCATTTGGTGAAATCTATGAAGGACAAGTTCTGGGGATGAGTGCTGATGGTGGCTCCATGCAGGTGGCCATAAAGGTCAGCCCACACTCTGCACAAGACAAGTTGCAGTGACACAGACAAAAAGCACACATAATTCACCCAGCAGCATCCAGCACAGACGTTTTTACTTCTCAGTCCAGAAGAATTTAATGAATTTATTTTTCAAAGTAGTTTATTGTTTTAATATTTTAAAAGATATAAGTGTGACAGTTGAAGAAAAGTTAAAATCACCCACAAACTGGAGCAAATGCGTTCAACCGTTGGCTTCACGTATGCAAAAAAAGTAAAGAAAAGCTGATCTCTGTAGGGTGTAGCCTGCTTTTCAGTGCTCTGCTGATTAGGTCTGGTAATAAAGTTCCCCTTGACAAAGGCACACACTTCAACCAGCTTCTCTGATTCTGTCTGCTCCACAGACTCTTCCAGAAATCTGCTCCGAGCAGGATGAAATGGATTTCCTG(___)GAGGCGCTGATTATGAGGTGCGACTAGCCGAGTCTGCCAGTTCAGCTTTATTATACAAGCAACGGAGACCAGGTTGTTGTTGCACATTTCTTCCTCTTTCTCTGTGTGGAAAAATCAGATGAAATGAAAGGTCCATTTGTAAAACGAGGTAAAAAAGCACAATTAGTTTATACATTTACACATCACTGCATCTTTTAGAGACATGGTTTTGGAAGCAAACAAAAATATGGCAACTTTAATTGTACTACACTGCTATTACCAGGTTTGACAAGGGCGAATTCTGCAGATATCCATCACACTGGCGGCCGCTCGAGCATGCATCTAGAGGGCCCAATCGCCCTATAGTA

**Rev. Complement** of *csf1ra* #18f 🡪 12bp deletion

CCAGTGTGCTGGAATTCGCCCTTCATAGATACCGTGCAAGCCTGCAGCTGAAGAGAATGAACATTCAGGAGCAAGGCCAGTACACGTTCTACGCCAGAAGCAACTTGGTCAACGCATCCATCAAGTTTCAAGCTCAAATGTATCGTAAGTGACAGAAATCAAAGCAAGTGTTGCTAATAATATCAACGCTGTAAAACCTGAAAGAGCATGTTTGCTTCTATTTTTTATTGCTATGCAACAGAGAGACCTGTTGCAGTGGTGCGATGGGAGAATGTCACCACTCTGACTTGCACCTCATTCGGCTATCCTGCTCCGAGAATCATCTGGTATCAGTGTCTAGGAATACGGCCCACGTAAGAACCGTTTAATGTAAAATGTAATAAATTGGCCATAAAAATCACCCAAAACATCACAAATTAGGAGCAAATAAGATCTTGTTCCTGTGTTTTGGGGTGATGACACAGGTGCAATGAGAGCACCTCCGGGTTGCAGTTGGCAGTCCCCCTCCAGGCCCTTACTGTGGAGGTCCAGAGGGAGGAGAACGGAGCTGTGGAAGTGGAGAGCGTCCTCAGAGTGGGACCGTCCACCCAGAGGATGACTGTGGAGTGTGTGGCTTTCAATTTGGTTGGCATCAGCAGAGACACTTTTTC(____________)TGGTGAGTGTTTGTTATTTTGATTGGTTATCATACAGTTGAAATGAGTCAAAACACCAATGAAGGTTTTTATCTGACTGGACAGATGAACTTTTAACCACCACCCTGAGCGGAGTAGCGGCAATCATGGCAATCCTCCTGGTGCTGCTGGTTTTTCTGCTTTACGAATATAAACAGGTACATTTAAATCCTAACAGCGAATCTAGTACAGGATCACTAGTTCAGTAGCAGGTCTTTTTGATCGCTCTGTTTGATTTTGTTTTTCAGAAGCCCAGGTATGAAATCCGTAAGGGCGAATTCTGCAGATATCCATCACACTGGCGGCCGCTCGAGCATGCATCTAGAGGGCCCAATCGCCCTA

*mitfa* #19a* 🡪 4bp deletion

GATAGGGCGATTGGGCCCTCTAGATGCATGCTCGAGCGGCCGCCAGTGTGATGGATATCTGCAGAATTCGCCCTTTGCTTCACATACGTTTGCAGTCCCACCTGGAAAGTCCATCAAAGTACCACATCCAACAGGCTCAGAGACAGCAGGTGAGACAGTACCTGTCCTCCACTCTGGGCGGGAAAGCCGGCGGTCAGCCTTCGGATCCCAGCATGCCCAGTGGCAGTGCCCCCAACAGTCCCATGGCCTTGCTCACCCTCGGCTCCAACCGTGGGAAGGAGGTACATTCATCAATAGCTTTTAAAGTCTCCACCTTAACTTCAAATTTAGTAAAGAAAATGTGATTTTGCACTAAACAGATGGACGATGTCATCGATGATATAATTAGCCTGGAGTCAAGTTACAACGAAGATGTTCTTGGACTGATGGACCCAGCTCTCCAAATCAATAACACGGTAATTTATGCAAACTCTGTTTCACCTCTTGGTTTTAGGTTTGATTCAGAACGGGTTTGTTACATTTGGACACCAATAATGTGTTTGACTCCTCAGCTGCCCGTTTCTGGGAATTTACTGGACGTGTACAGCAACCAAGGGCTTCCCCTCACGAGTCTCTCTATCAGCAGCTCGTGTGCACCCAACATCAAAAGGGA(____)ACTGGTAAGCCCCACGAACCCAATATAAATGTCTTGTTGTTAGTTTTGAAAATGATGACAAATTATAGAAACCTTTTAAAATCTTTTAATGCTGACTCATCTTTTCTTCTCTTTTCTAATGTGGTTTGAAATTTTTAATACATATTTACTGTAGTTAAATGTGTGATTGAGAAAAAAGTACATCTGCATCAATATACAGAACGTGAAATGATAATTAAGAGCATATTTTTATTTTATGCGCAATTTGGTATTGCACGAGA

*ltk* #19b* 🡪 3bp deletion

ATAGGGCGATTGGGCCCTCTAGATGCATGCTCGAGCGGCCGCCAGTGTGATGGATATCTGCAGAATTCGCCCTTTGTTCTGTCACCACCCTTGTTTGTAGTGTGGTACCGCAGGAAGAATGACCTGCACGCCGTGAGGGGACGTCTGCAGAGTCCAGAGTACAAGTTGAGCAAGATCCGCTCCTCCACCATCATGACCGACTACAACCCCAACTACTGCTTCGCTGGCAAGGCTGCATCCCTCAGCGATCTGAAGGAGGTACCACGAAAGAACATCACTCTTCTCAGGTACTGTTTGATCCACGAGGGAACAATGTCAAAATGTACCAGATTTTCAATTTCATTAAAAACATATTTTTGTTGGTATTTTATTTTATTTATTTTTTTAGGGCTCTTGGACACGGAGCATTTGGTGAAGTCTATGAAGGACAAGTTCTGGGGATGAGTGCTGATGGTGGCTCCATGCAGGTGGCCATAAAGGTCAGCCCACACTCTGCACAAGACAAGTTGCAGTGACACAGACAAAAAGCACGCATAATTCACCCAGCAGCATCCAGCACAGACGTTTTTACTTCTCAGTCCAGAAGAATTTAATGAATTTATTTTTCAAAGTAGTTTATTGTTTTAATATTTTAGAAGATATAAGTGTGACAGTTGAAGAAAAGTTAAAATCACCCACAAACTGGAGCAAATGCGTTCAACCGTTGGCTTCACGTATGCAAAAAAAGTAAAGAAAAGCTGATCTCTGTAGGGTGTAGCCTGCTTTTCAGTGCTCTGCTGATTAGGTCTGGTAATAAAGTTCCCCTTGACAAAGGCACACACTTCAACCAGCTTCTCTGATTCTGTCTGCTCCACAGACTCTTCCAGAAATCTGCTCCGAGCAGGATGAAATGGATTTCCTG(___)GAGGCGCTGATTATGAGGTGCGACTAGCCGAGTCTGCCAGTTCAGCTTTATTATACAAGCAACGGAAACCAGG

*csf1ra*_19g 🡪 12 bp deletion

GGGCGAATTGGGCCCTCTAGATGCATGCTCGAGCGGCCGCCAGTGTGATGGATATCTGCAGAATTCGCCCTTACGGATTTCATACCTGGGCTTCTGAAAAACAAAATCAAACAGAGCGATCAAAAAGACCTGCTACTGAACTAGTGATCCTGTACTAGATTCGCTGTTAGGATTTAAATGTACCTGTTTATATTTGTAAAGCAGAAAAACCAGCAGCACCAGGAGGATTGCCATGATTGCCGCTACTCCGCTCAGGGTGGTGGTTAAAAGTTCATCTGTCCAGTCAGATAAAAACCTTCATTGGTGTTTTGACTCATTTCAACTGTATGATAACCAATCAAAATAACAAACACTCACCAGAAAAAGTGTCTCTGCTGATGCCAACCAAATTGAAAGCCACACACTCCACAGTCATCCTCTGGGTGGACGGTCCCACTCTGAGGACGCTCTCCACTTCCACAGGCTCCGTTCTCCTCCCTCTGGACCTCCACAGTAAGAGCCTGGAGGGGGACTGGCCAACTGCAACCCGGAGGTGCTCTCATTGCACCTGTGTCATCACCCCCAAAACACAGGAACAAGGACCTTATTTGCTCCTAATTTGTGATGTTTTGGGTGATTTTTATGGGCCAATTTATTTACATTTTTACATTTAAAACGGTTTCTTTACGTGGGGCCGGTATTCCCTAGGACACCTGAATACCCAGATGATTTCTCGGGAGCAGGGATAGCCGAAATGAGG

*mitfa* #29a* 🡪 -10bp

ATATAGGGCGATTGGGCCCTCTAGATGCATGCTCGAGCGGCCGCCAGTGTGATGGATATCTGCAGAATTCGCCCTTTGCTTCACATACGTTTGCAGTCCCACCTGGAAAGTCCATCAAAGTACCACATCCAACAGGCTCAGAGACAGCAGGTGAGACAGTACCTGTCCTCCACTCTGGGCGGGAAAGCCGGCGGTCAGCCTTTGGATCCCAGCATGCCCAGTGGCAGTGCCCTCAACAGTCCCATGGCCTTGCTCACCCTCGGCTCCAACTGTGAGAAGGAGGTACATTCATCAATAGCTTTTAAAGTCTCCACCTTAACTTCAAATTTAGTAAAGAAAATGTGATTTTGCACTGAACAGATGGACGATGTCATCGATGATATAATTAGCCTGGAGTCAAGTTACAACGAAGGTGTTCTTGGACTGATGGACCCAGCTCTCCAAATCAATAACACGGTAATTTATGCAAACTCTGTTTCACCTCTTGGTTTTAGGTTTGATTCAGAACGGGTTTGTTACATTTGGACACCAATACTATGTTTGACTCCTCAGCTGCCCGTTTCTGGGAATTTACTGGACGTGTACAGCAACCAAGGGCTTCCCCTCACGAGTCTCTCTATCAGCAGCTCGTGTGCACCCAACATCAAAAGGGA(__________)AAGCCCCACGAACCCAATATAAATGTCTTGTTGTTAGCTTTGAAAATGATGACAAATTATAGAAACCTTTTAAAATCTTTTAATGCTGACTCATCTTTTCTTCTCTTTTCTAATGTGGTTTGAAATTTTTAATACATATTTACTGTAGTTAAATGTGTGATTGAGAAAAAAGTACATCTGCATCAATATACAGAACGTGAAATGATAATTAAGAGCATATTTTTATTTTATGTGCAATTTGGTATTGCACAAGATACTACTGAATCGATGATTTACCAAATGATTACTGCAGCTCCTGGCATGAAGCAAGTACTGGACAAGCCTGGATCCTGTGGCCAGTTTGAAAGTTATCAAAGGTCTGAAGGGC

*ltk*_29c 🡪 4bp deletion

ATAGGGCGATTGGGCCCTCTAGATGCATGCTCGAGCGGCCGCCAGTGTGATGGATATCTGCAGAATTCGCCCTTGTCAAACCTGGTAATAGCAGTGTAGTACAATTAAAGTTGCCATATTTTTGTTTGCTTCCAAAACCATGTCTCTAAAAGATGCAGTGATGTGTAAATGTATAAACTAATTGTGCTTTTTTACCTCGTTTTACAAATGGACCTTTCATTTCATCTGATTTTTCCACACAGAGAAAGAGGAAGAAATGTGCAACAACAACCTGGTCTCCGTTGCTTGTATAATAAAGCTGAACTGGCAGACTCGGCTAGTCGCACCTCATAATCAGCGCCTCAGGAAATCCATTTCATCCTGCTCGGAGCAGATTTCTGGAAGAGTCTGTGGAGCAGACAGAATCAGAGAAGCTGGTTGAAGTGTGTGCCTTTGTCAAGGGGAACTTTATTACCAGACCTAATCAGCAGAGCACTGAAAAGCAGGCTACACCCTACAGAGATCAGCTTTTCTTTACTTTTTTTGCATACGTGAAGCCAACGGTTGAACGCATTTGCTCCAGTTTGTGGGTGATTTTAACTTTTCTTCAACTGTCACACTTAATCTTCTAAAATATTAAAACAATAAACTACTTTGAAAAATAAATTCATTAAATTCTTCTGGACTGAGAAGTAAAAACGTCTGTGCTGGATGCTGCTGGGTGAATTATGTGTGCTTTTTGTCTGTGTCACTGCAACTTGTCTTGTGCAGAGTGTGGGCTGACCTTTATGGCCACCTGCATGGAGCCACCATCAGCACTCATCCCCAGAACTTGTCCTTCATAGACTTCACCAAATGCTCCGTGTCCAAGAGCCCTAAAAGAATAAATAAAATAAAATAAAATACCAACAAAAATATGTTTTTAATGAAATTGAAAATCTGGTACATTTTGACATTGTTCCCTCGTGGATCAAACAGTACCTGAGAAGAGT

*csf1ra*_29c –> 9bp insertion + 4bp exchange

TAGGGGCGATTGGGCCCTCTAGATGCATGCTCGAGCGGCCGCCAGTGTGATGGATATCTGCAGAATTCGCCCTTCATAGATACCGTGCAAGCCTGCAGCTGAAGAGAATGAACATTCAGGAGCAAGGCCAGTACACGTTCTACGCCAGAAGCAACTTGGTCAACGCATCCATCAAGTTTCAAGTTCAAATGTATCGTAAGTGACAGAAATCAAAGCAAGTGTTGCTAATAATATCAACGCTGTAAAACCTGAAAGAGTATGTTTGCTTCTATTTTTTATTGCTATGCAACAGAGAGACCTGTTGCAGTGGTGCGATGGGAGAATGTCACCACTCTGACTTGCACCTCATTCGGCTATCCTGCTCCGAGAATCATCTGGTATCAGTGTCTAGGAATACGGCCCACGTAAGAACCGTTTAATGTAAAATGTAATAAATTGGCCATAAAAATCACCCAAAACATCACAAATTAGGAGCAAATAAGATCTTGTTCCTGTGTTTTGGGGTGATGACACAGGTGCAATGAGAGCACCTCCGGGTTGCAGTTGGCAGTCCCCCTCCAGGCTCTTACTGTGGAGGTCCAGAGGGAGGAGAACGGAGCTGTGGAAGTGGAGAGCGTCCTCAGAGTGGGACCGTCCACCCAGAGGATGACTGTGGAGTGTGTGGCTTTCAATTTGGTTGGCATCAGCAGAGACACTTTTTCCACTTGGTCACACTTGGTTTCTGGTGAGTGTTTGTTATTTTGATTGGTTATCATACAGTTGAAATGAGTCAAAACACCAATGAAGGTTTTTATCTGACTGGACAGATGAACTTTTAACCACCACCCTGAGCGGAGTAGCGGCAATCATGGCAATCCTCCTGGTGCTGCTGGGTTTTTCTGCTTTACAAATATAAACAGGGTACATTTTAAATCCTAACAGCCGAATCTAAGTACA
