# Supplementary material for: Generation of a transparent killifish line through multiplex CRISPR/Cas9mediated gene inactivation
Source: eLife. 2023 Feb 23;12:e81549. doi: 10.7554/eLife.81549 (PMC10010688; doi:10.7554/eLife.81549)
Supplement: Figure 2—source data 1. [file elife-81549-fig2-data1.zip › Figure_2_source_data/Figure_2_panel_B_source_data/WKM_FACS_csf1ra_mutants.pdf]

BD FACSDiva 8.0.1

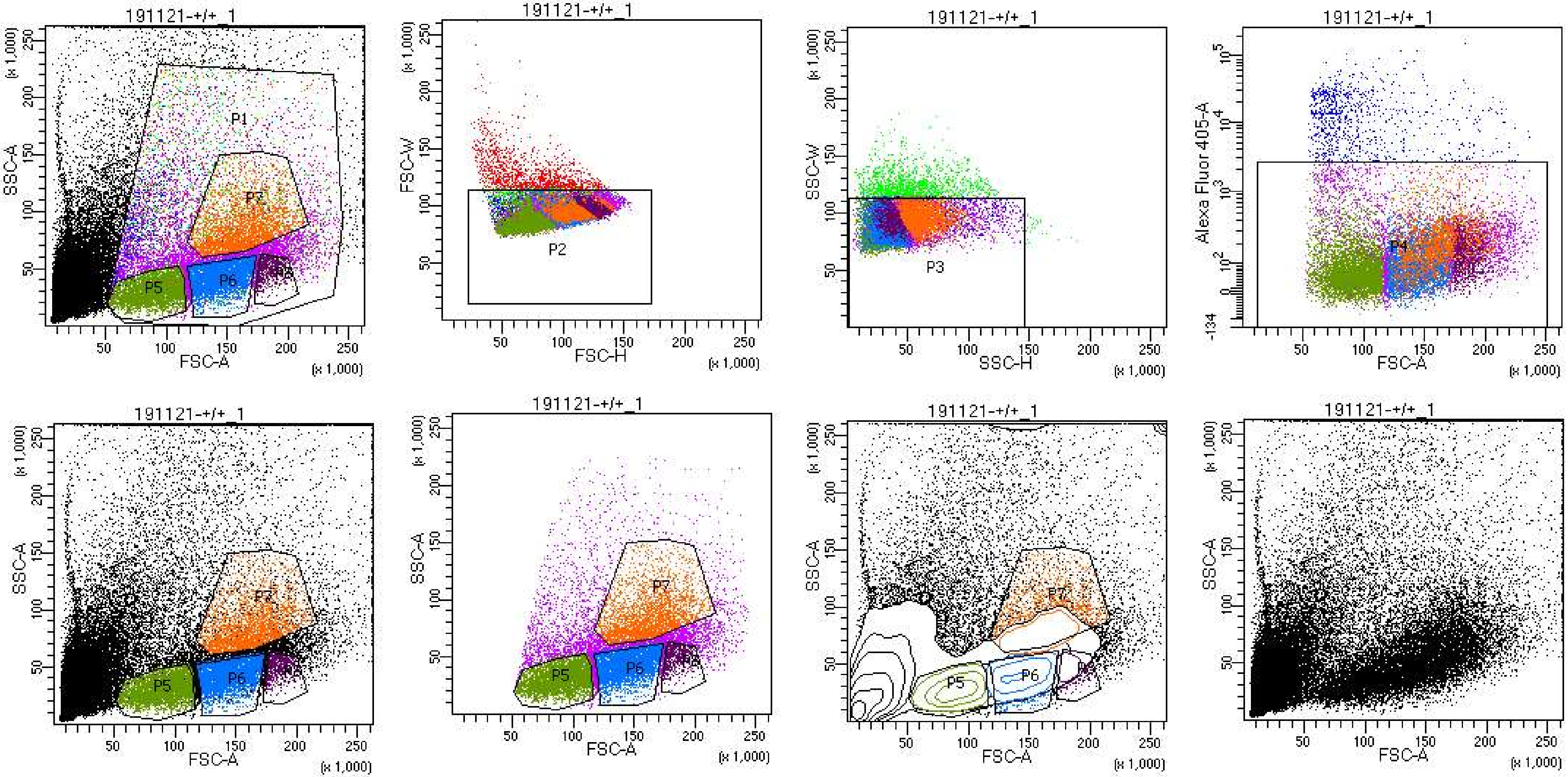

Tube: +/+_1

Population	#Events	%Parent	%Total
All Events	50,000	####	100.0
P1	17,553	35.1	35.1
P2	16,377	93.3	32.8
P3	15,055	91.9	30.1
P4	14,248	94.6	28.5
P5	4,001	28.1	8.0
P6	3,620	25.4	7.2
P7	2,632	18.5	5.3
P8	646	4.5	1.3

BD FACSDiva 8.0.1

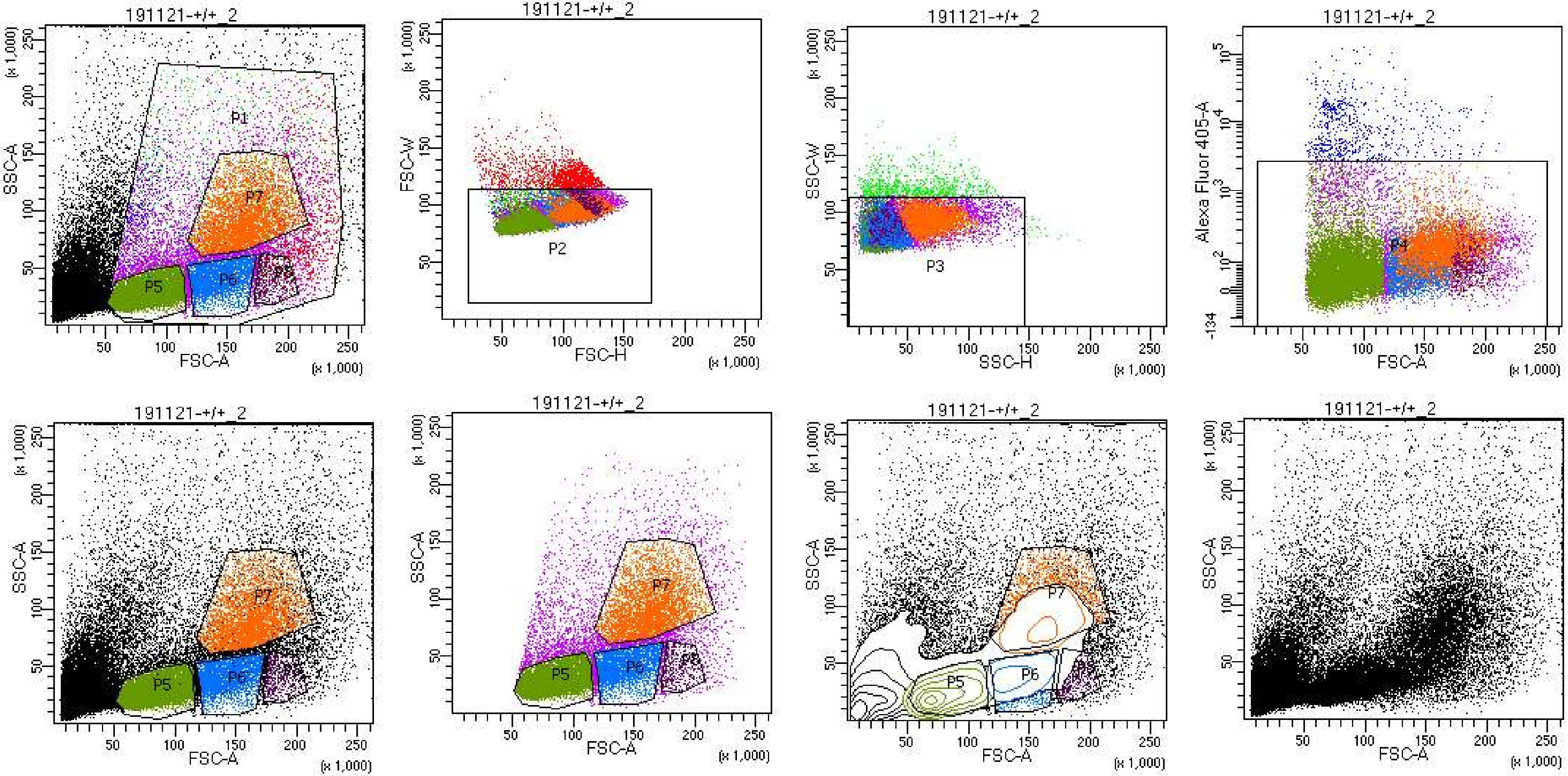

Tube: +/+_2

Population	#Events	%Parent	%Total
All Events	50,000	####	100.0
P1	26,704	53.4	53.4
P2	24,996	93.6	50.0
P3	24,147	96.6	48.3
P4	23,498	97.3	47.0
P5	12,949	55.1	25.9
P6	2,774	11.8	5.5
P7	4,209	17.9	8.4
P8	387	1.6	0.8

BD FACSDiva 8.0.1

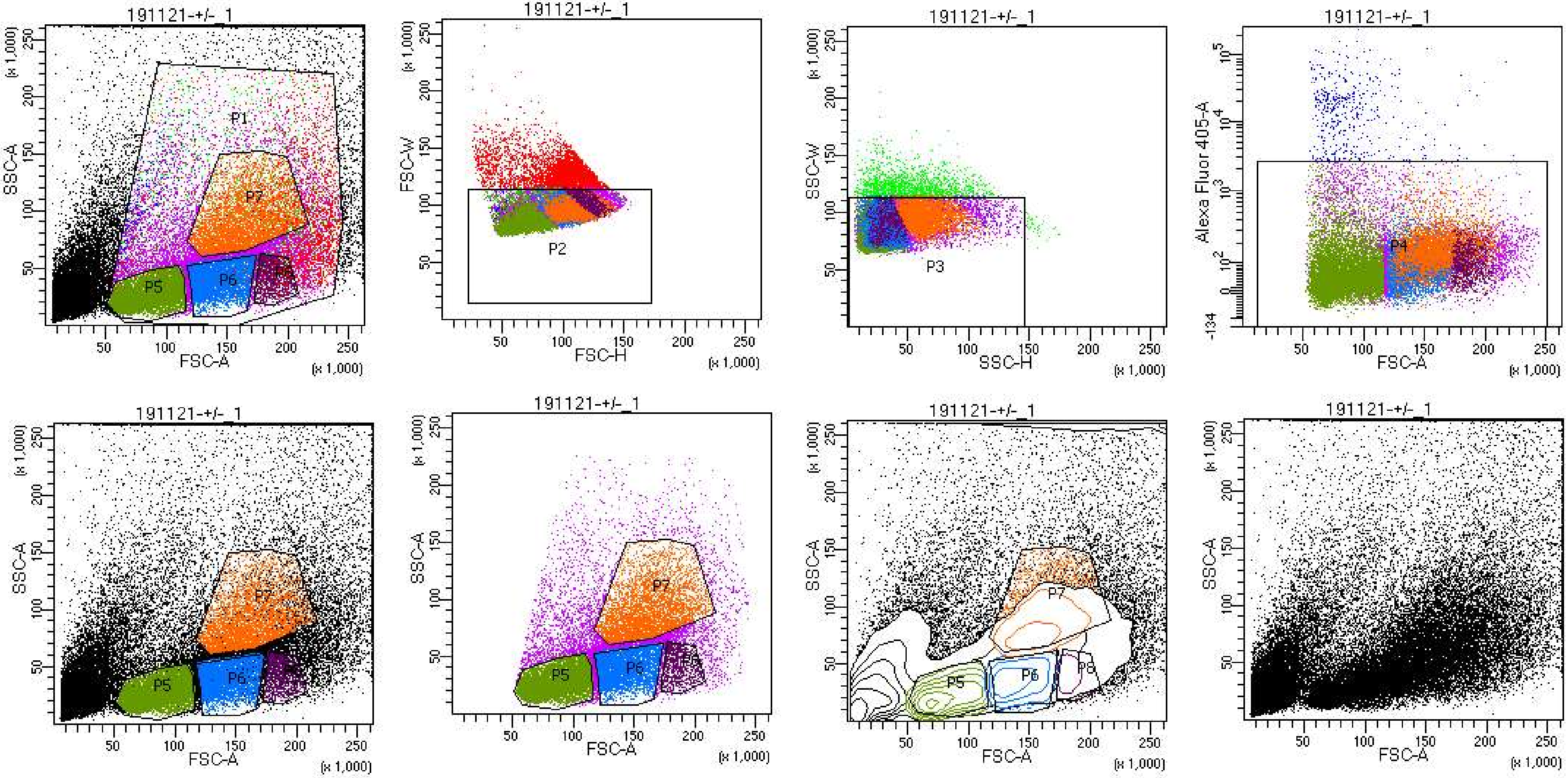

Tube: +/-_1

Population	#Events	%Parent	%Total
All Events	50,000	####	100.0
P1	29,505	59.0	59.0
P2	25,226	85.5	50.5
P3	23,780	94.3	47.6
P4	23,364	98.3	46.7
P5	10,133	43.4	20.3
P6	4,226	18.1	8.5
P7	4,174	17.9	8.3
P8	795	3.4	1.6

BD FACSDiva 8.0.1

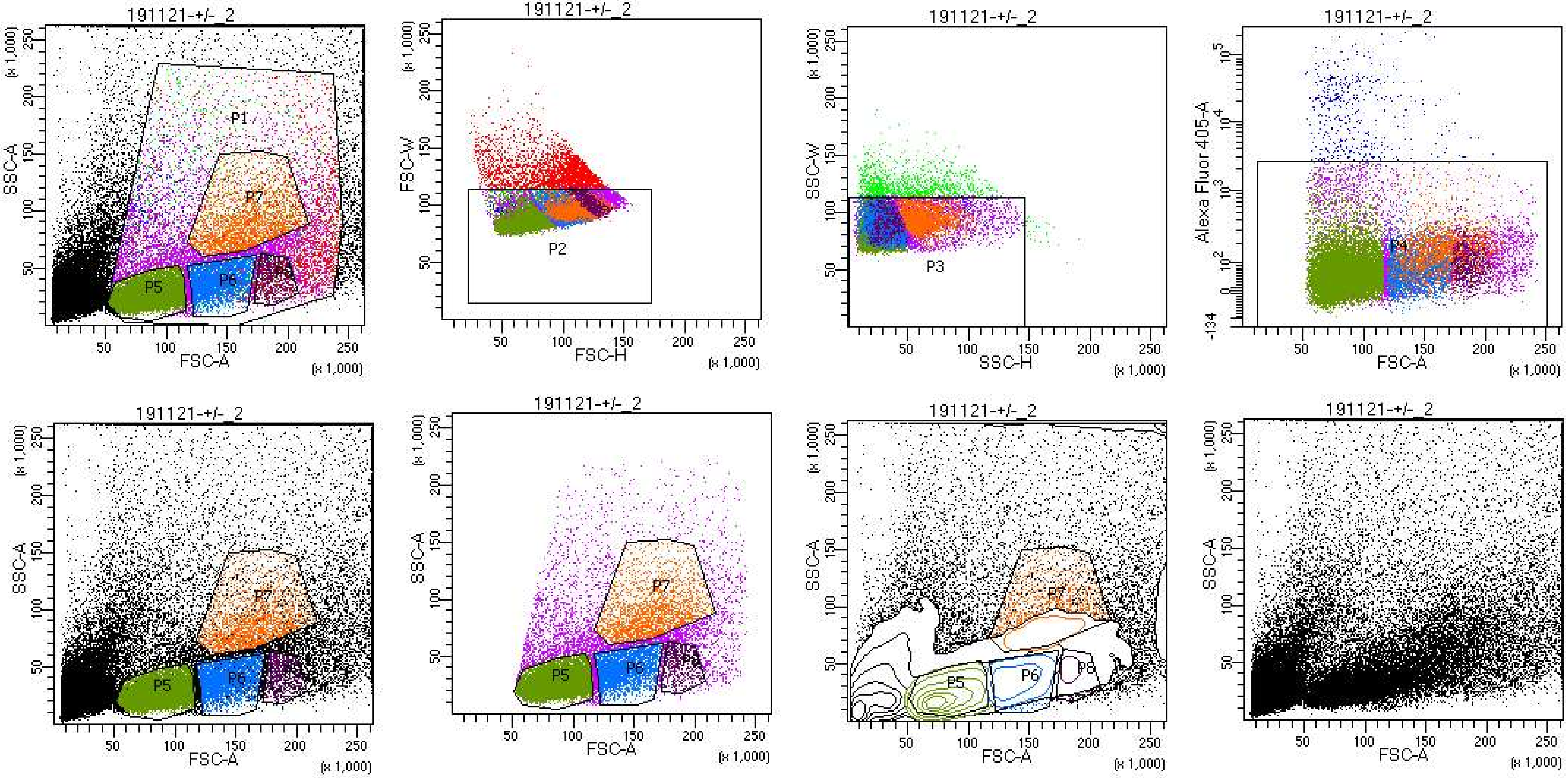

Tube: +/-_2			
Population	#Events	%Parent	%Total
All Events	50,000	####	100.0
P1	25,804	51.6	51.6
P2	23,100	89.5	46.2
P3	22,035	95.4	44.1
P4	21,634	98.2	43.3
P5	13,030	60.2	26.1
P6	2,625	12.1	5.2
P7	1,983	9.2	4.0
P8	554	2.6	1.1

BD FACSDiva 8.0.1

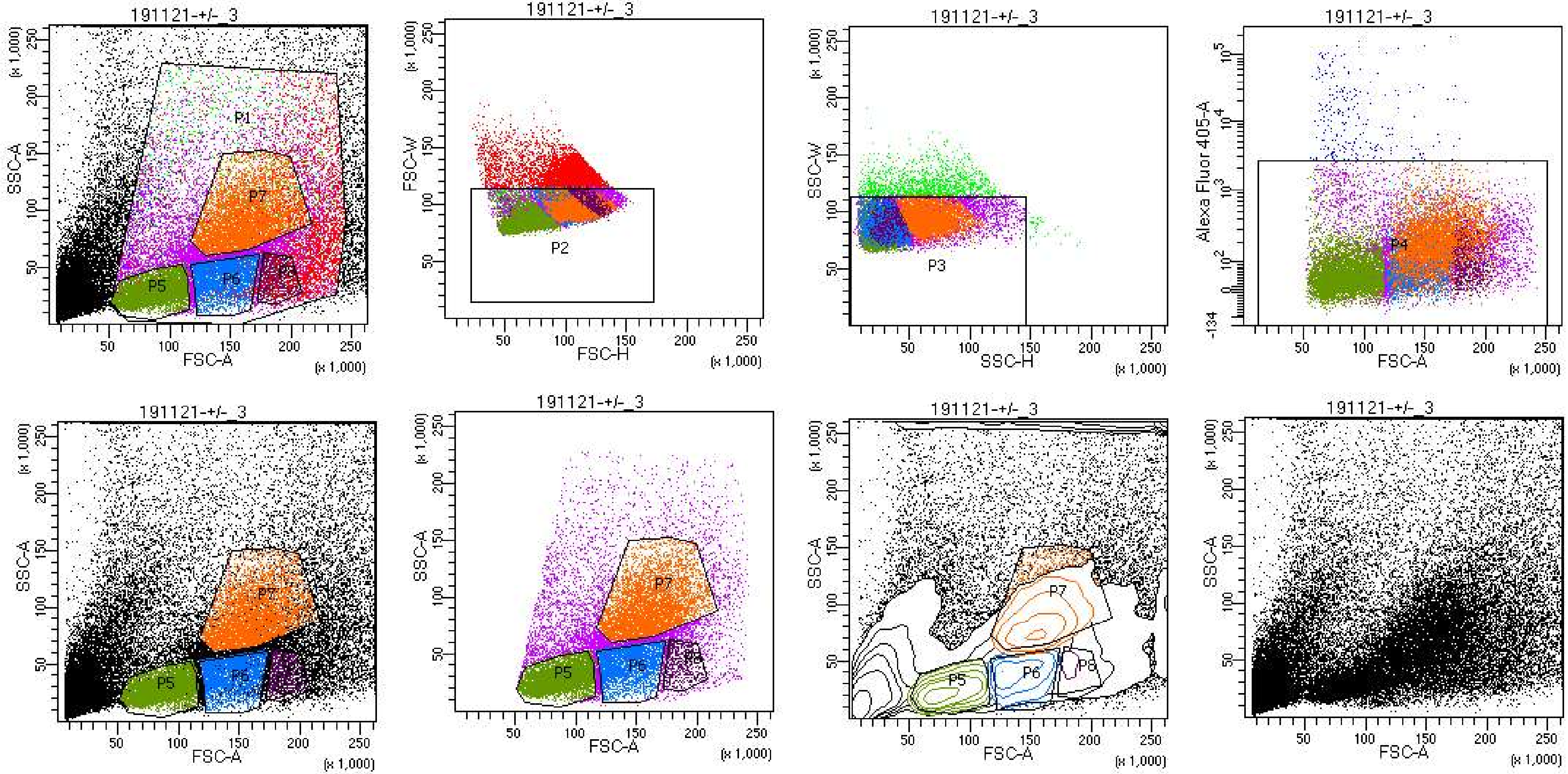

Tube: +/-_3

Population	#Events	%Parent	%Total
All Events	50,000	####	100.0
P1	25,400	50.8	50.8
P2	19,985	78.7	40.0
P3	18,739	93.8	37.5
P4	18,502	98.7	37.0
P5	5,552	30.0	11.1
P6	2,902	15.7	5.8
P7	5,262	28.4	10.5
P8	543	2.9	1.1

BD FACSDiva 8.0.1

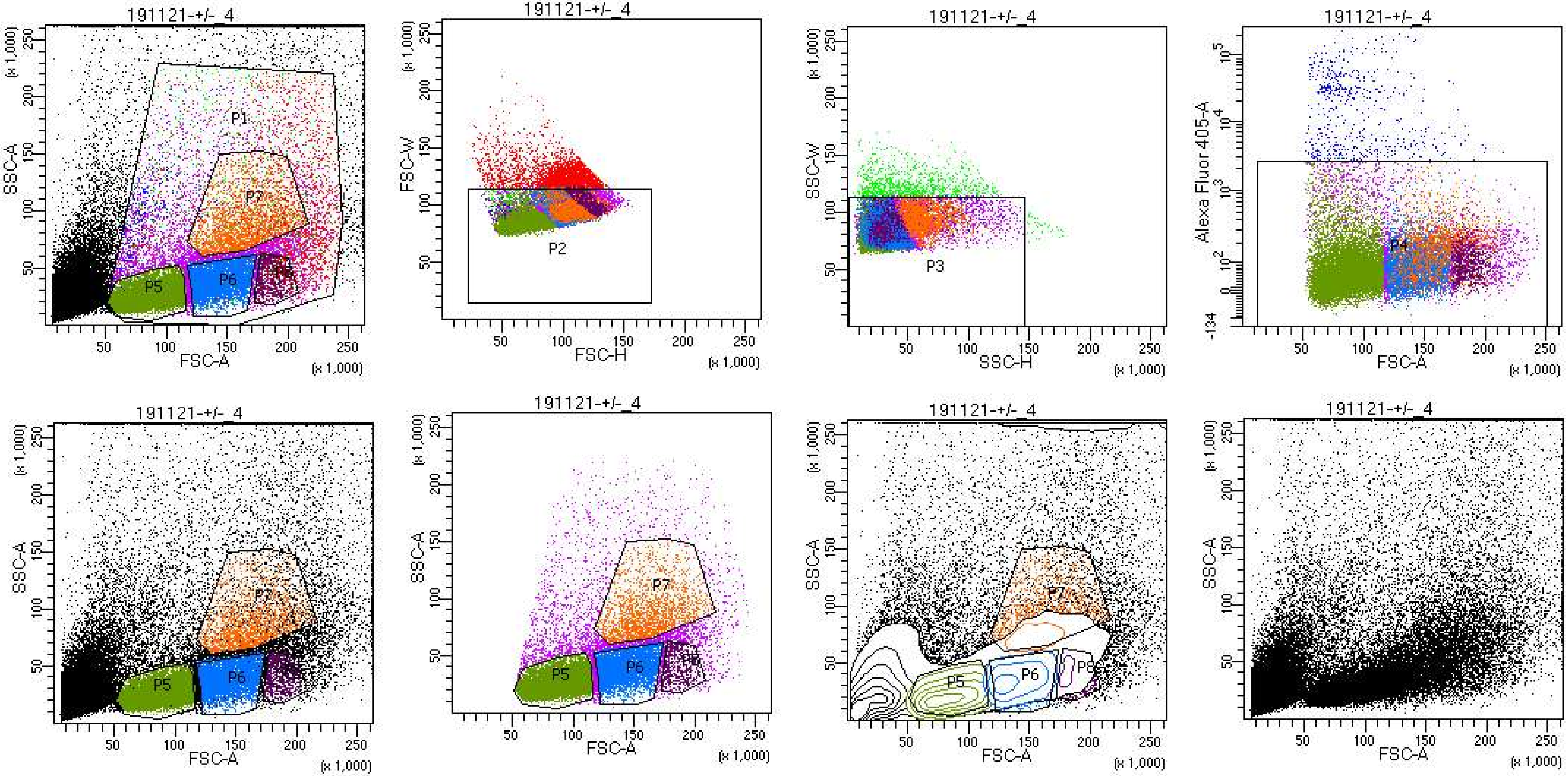

Tube: +/-_4

Population	#Events	%Parent	%Total
All Events	50,000	####	100.0
P1	26,763	53.5	53.5
P2	23,572	88.1	47.1
P3	22,599	95.9	45.2
P4	22,087	97.7	44.2
P5	11,613	52.6	23.2
P6	4,886	22.1	9.8
P7	1,918	8.7	3.8
P8	628	2.8	1.3

BD FACSDiva 8.0.1

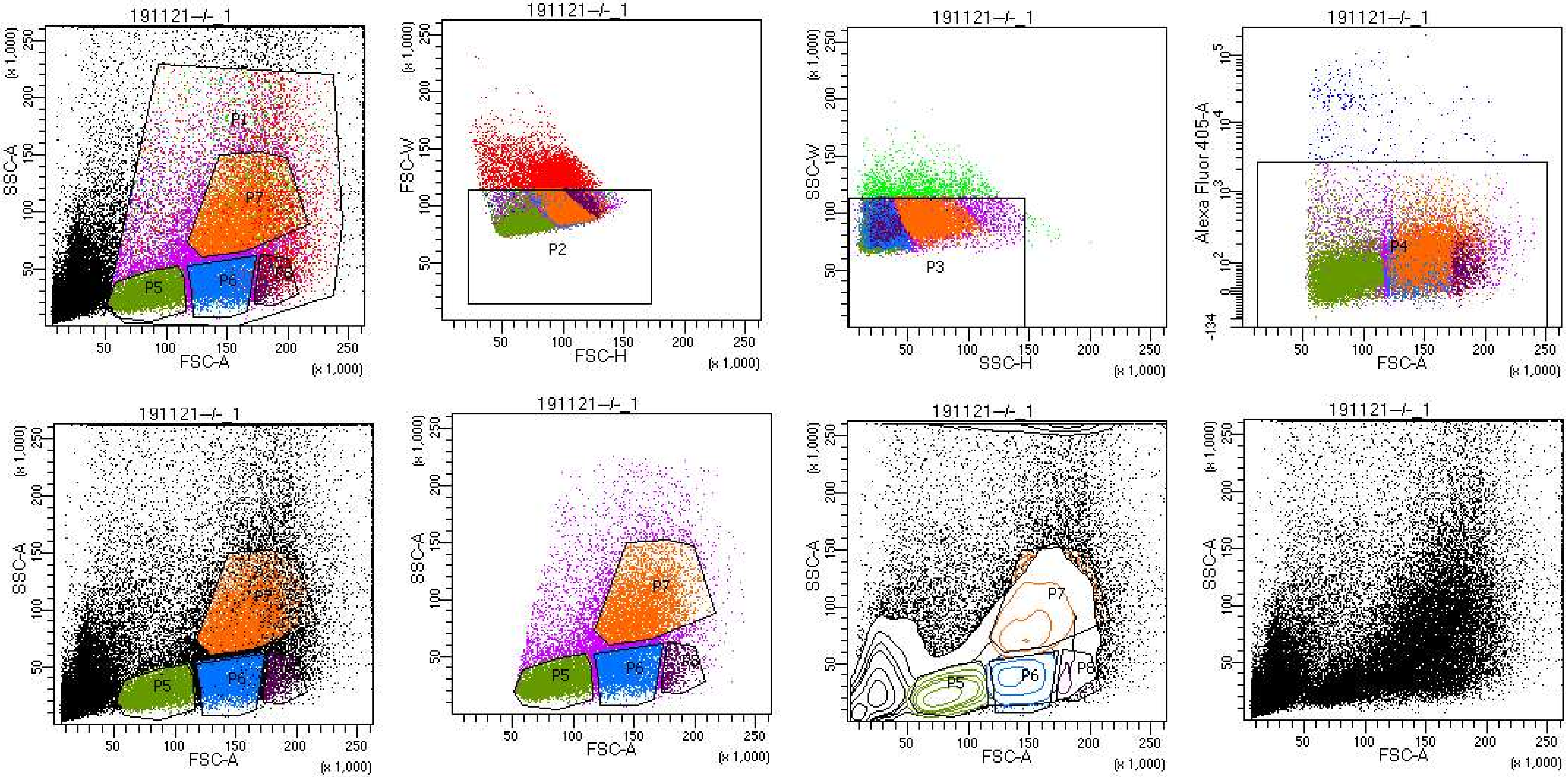

Tube: -/-_1

Population	#Events	%Parent	%Total
All Events	50,000	####	100.0
P1	28,607	57.2	57.2
P2	21,784	76.1	43.6
P3	20,371	93.5	40.7
P4	20,134	98.8	40.3
P5	7,503	37.3	15.0
P6	3,471	17.2	6.9
P7	4,862	24.1	9.7
P8	555	2.8	1.1

BD FACSDiva 8.0.1

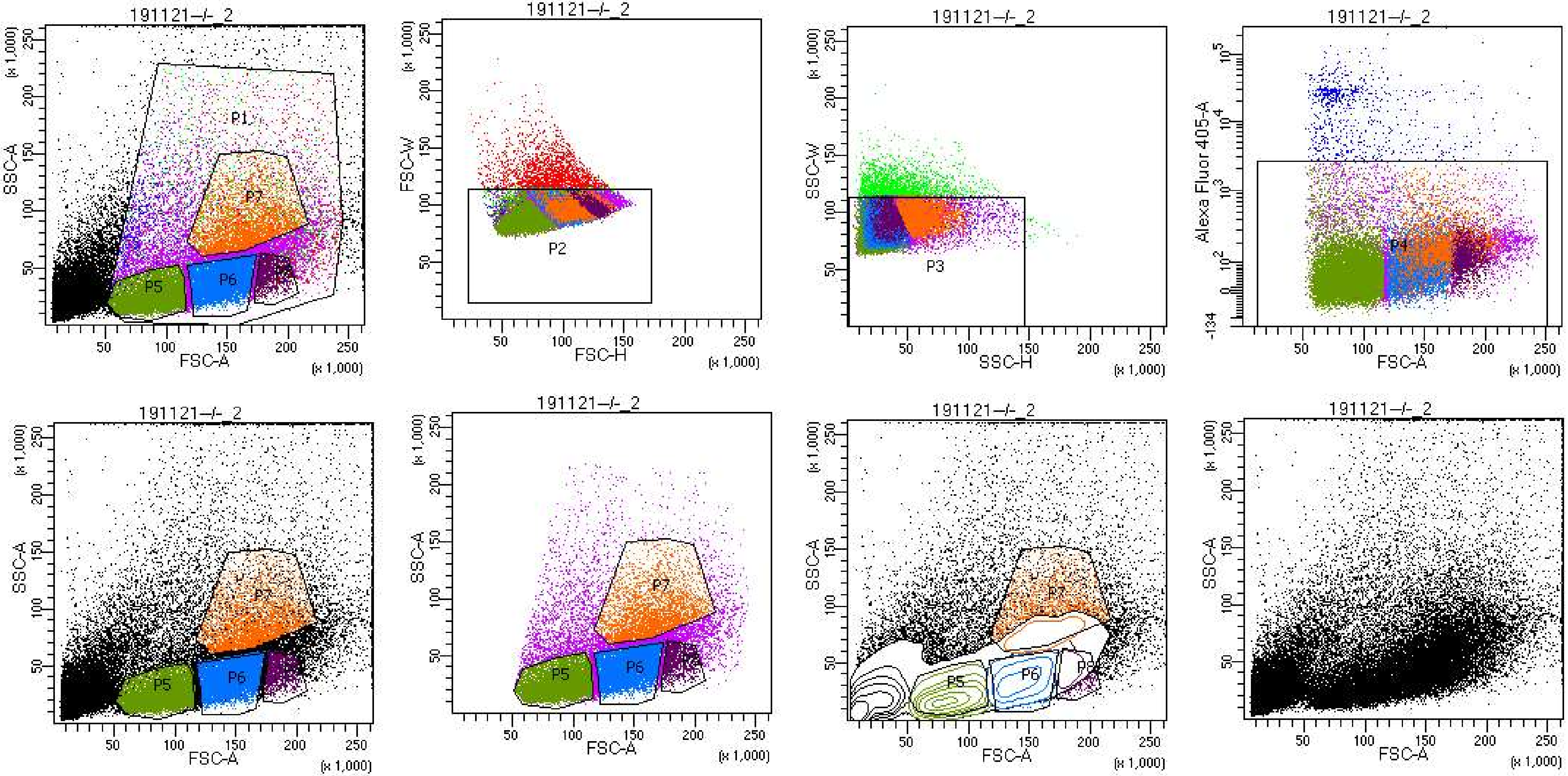

Tube: -/-_2

Population	#Events	%Parent	%Total
All Events	50,000	####	100.0
P1	32,042	64.1	64.1
P2	30,103	93.9	60.2
P3	28,231	93.8	56.5
P4	27,521	97.5	55.0
P5	12,497	45.4	25.0
P6	6,188	22.5	12.4
P7	3,099	11.3	6.2
P8	1,137	4.1	2.3

BD FACSDiva 8.0.1

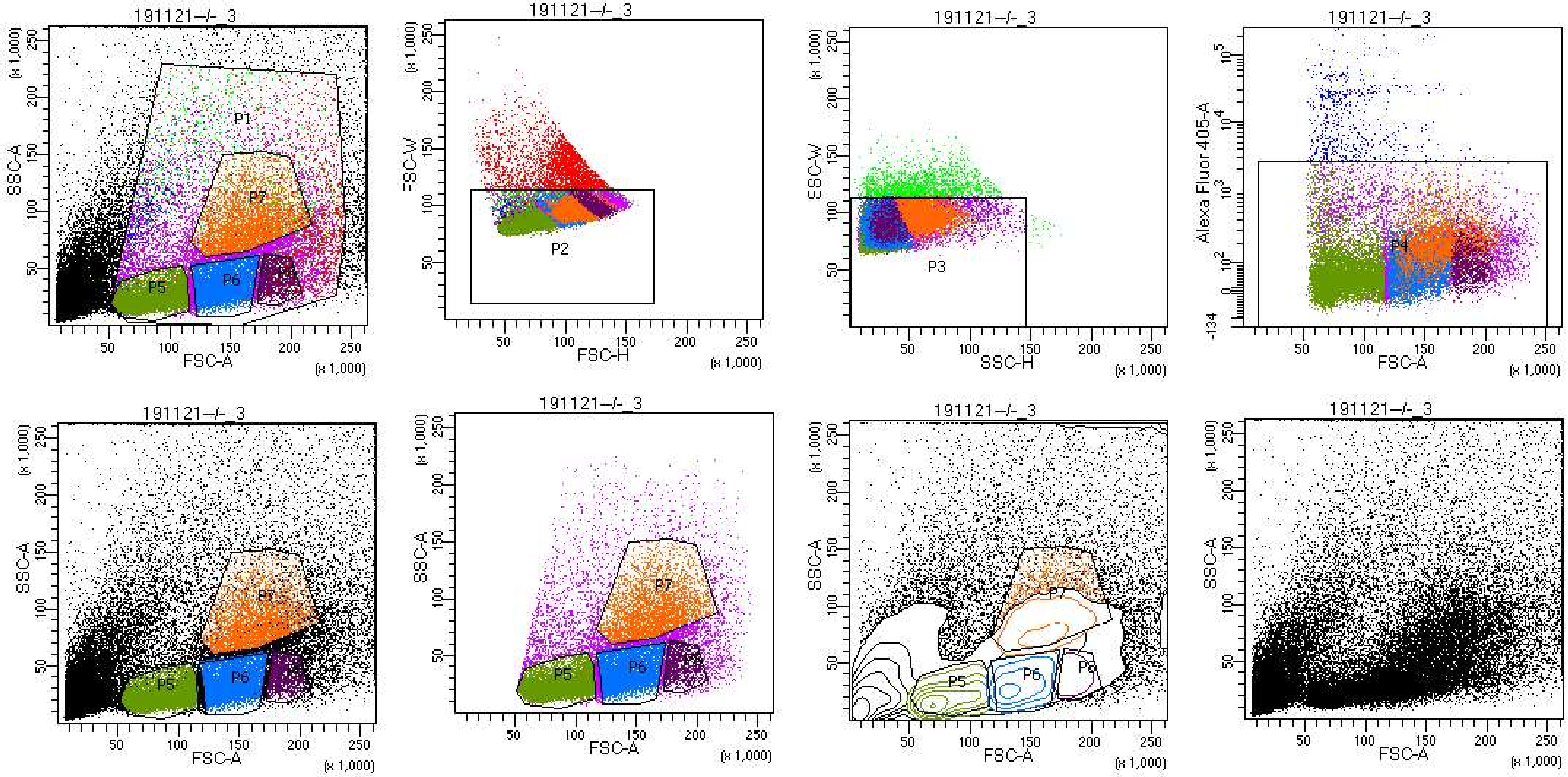

Tube: -/-_3

Population	#Events	%Parent	%Total
All Events	50,000	####	100.0
P1	27,923	55.8	55.8
P2	25,156	90.1	50.3
P3	23,488	93.4	47.0
P4	22,908	97.5	45.8
P5	8,827	38.5	17.7
P6	5,513	24.1	11.0
P7	3,623	15.8	7.2
P8	975	4.3	2.0

BD FACSDiva 8.0.1

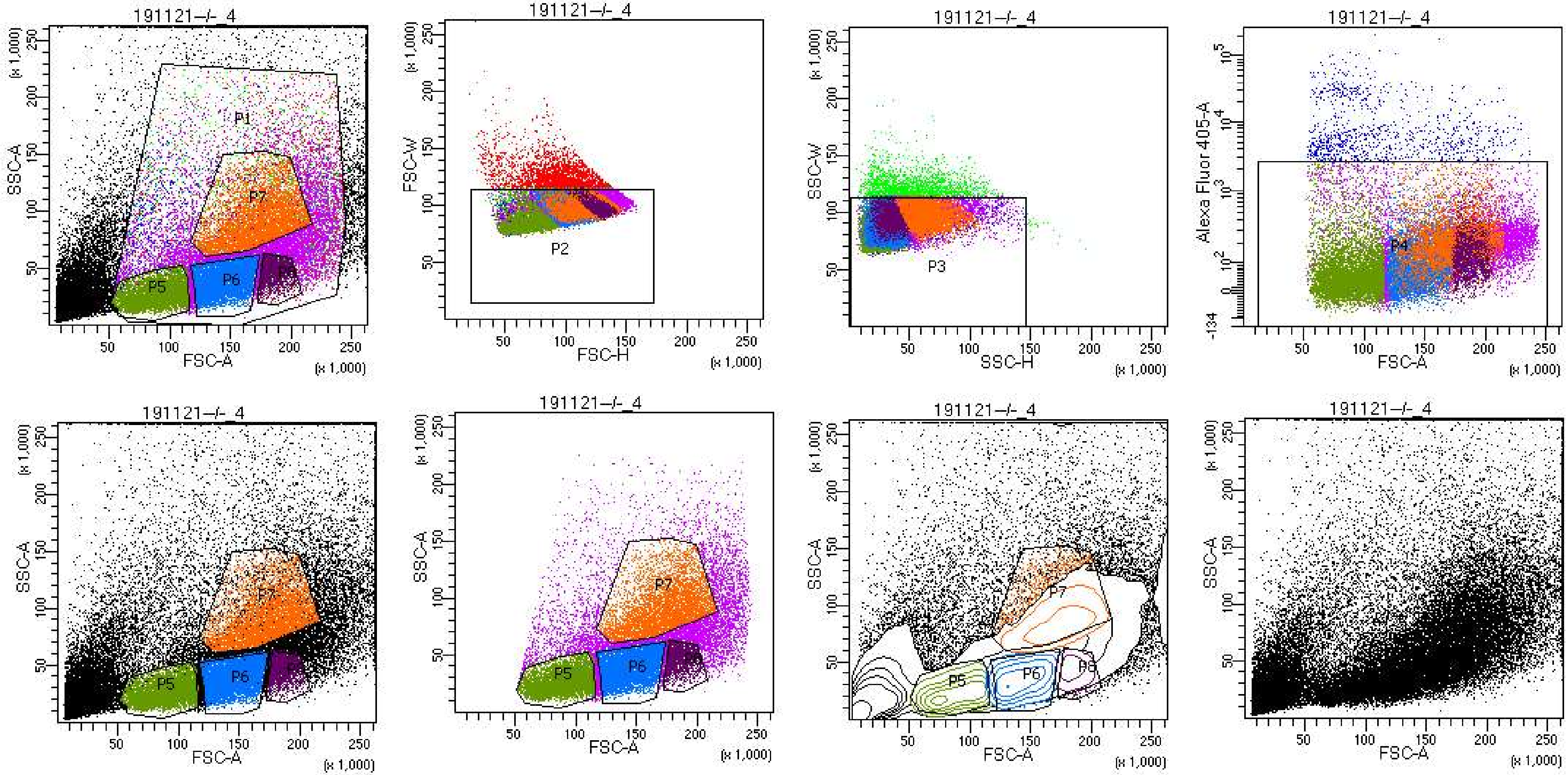

Tube: -/-_4

Population	#Events	%Parent	%Total
All Events	50,000	####	100.0
P1	29,972	59.9	59.9
P2	27,282	91.0	54.6
P3	25,362	93.0	50.7
P4	24,430	96.3	48.9
P5	6,014	24.6	12.0
P6	6,118	25.0	12.2
P7	4,879	20.0	9.8
P8	1,467	6.0	2.9
