# Supplementary material for: Generation of a transparent killifish line through multiplex CRISPR/Cas9mediated gene inactivation
Source: eLife. 2023 Feb 23;12:e81549. doi: 10.7554/eLife.81549 (PMC10010688; doi:10.7554/eLife.81549)
Supplement: Figure 2—figure supplement 1—source data 1. [file elife-81549-fig2-figsupp1-data1.zip › Figure_2_figure_supplement_1_source_data/Figure_2_figure_supplement_1_panel_CD_source_data/FACS_WKM_panel_cd.pdf]

BD FACSDiva 8.0.1

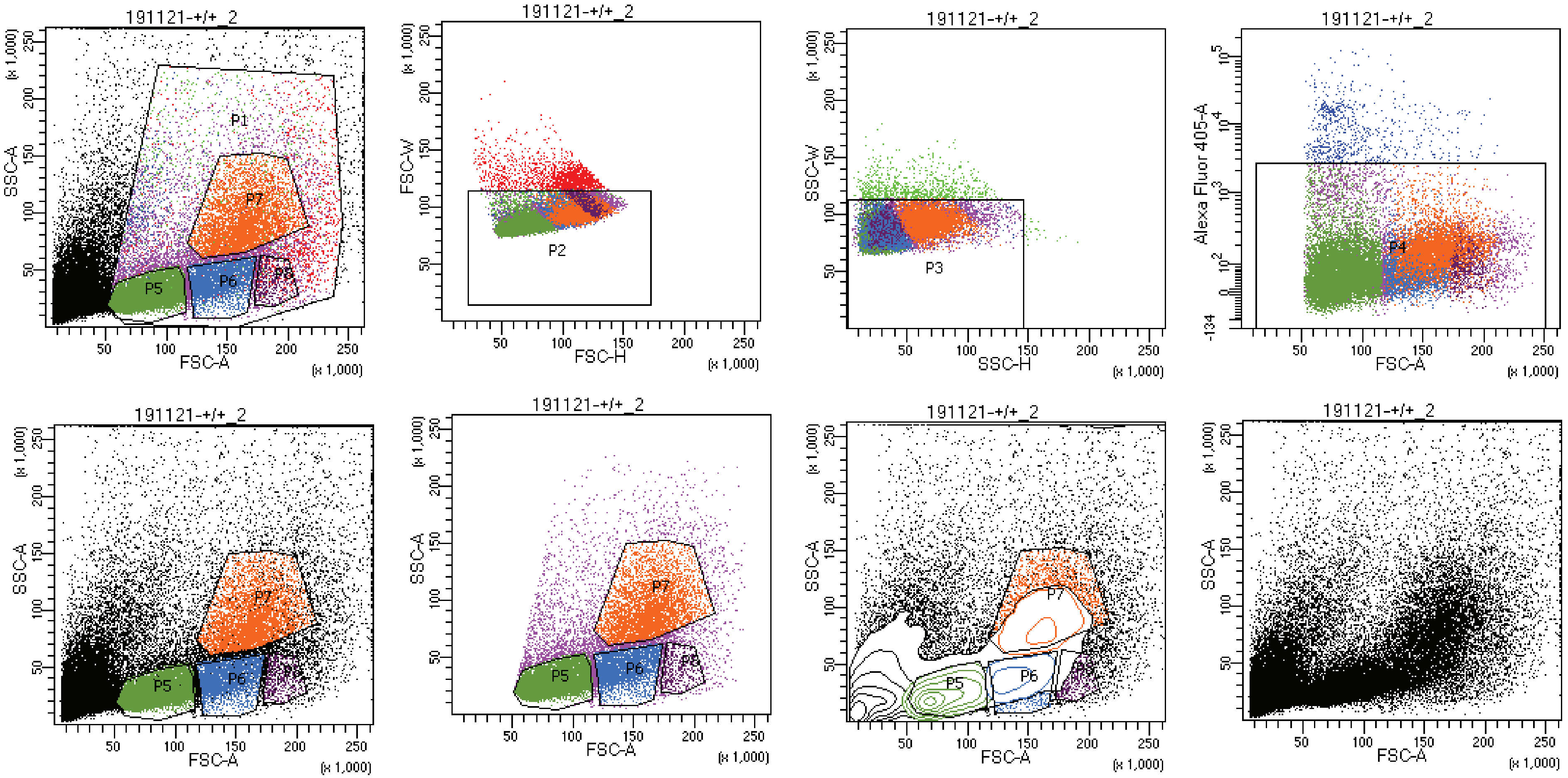

Tube: +/+_2

Population	#Events	%Parent	%Total
All Events	50,000	####	100.0
P1	26,704	53.4	53.4
P2	24,996	93.6	50.0
P3	24,147	96.6	48.3
P4	23,498	97.3	47.0
P5	12,949	55.1	25.9
P6	2,774	11.8	5.5
P7	4,209	17.9	8.4
P8	387	1.6	0.8

BD FACSDiva 8.0.1

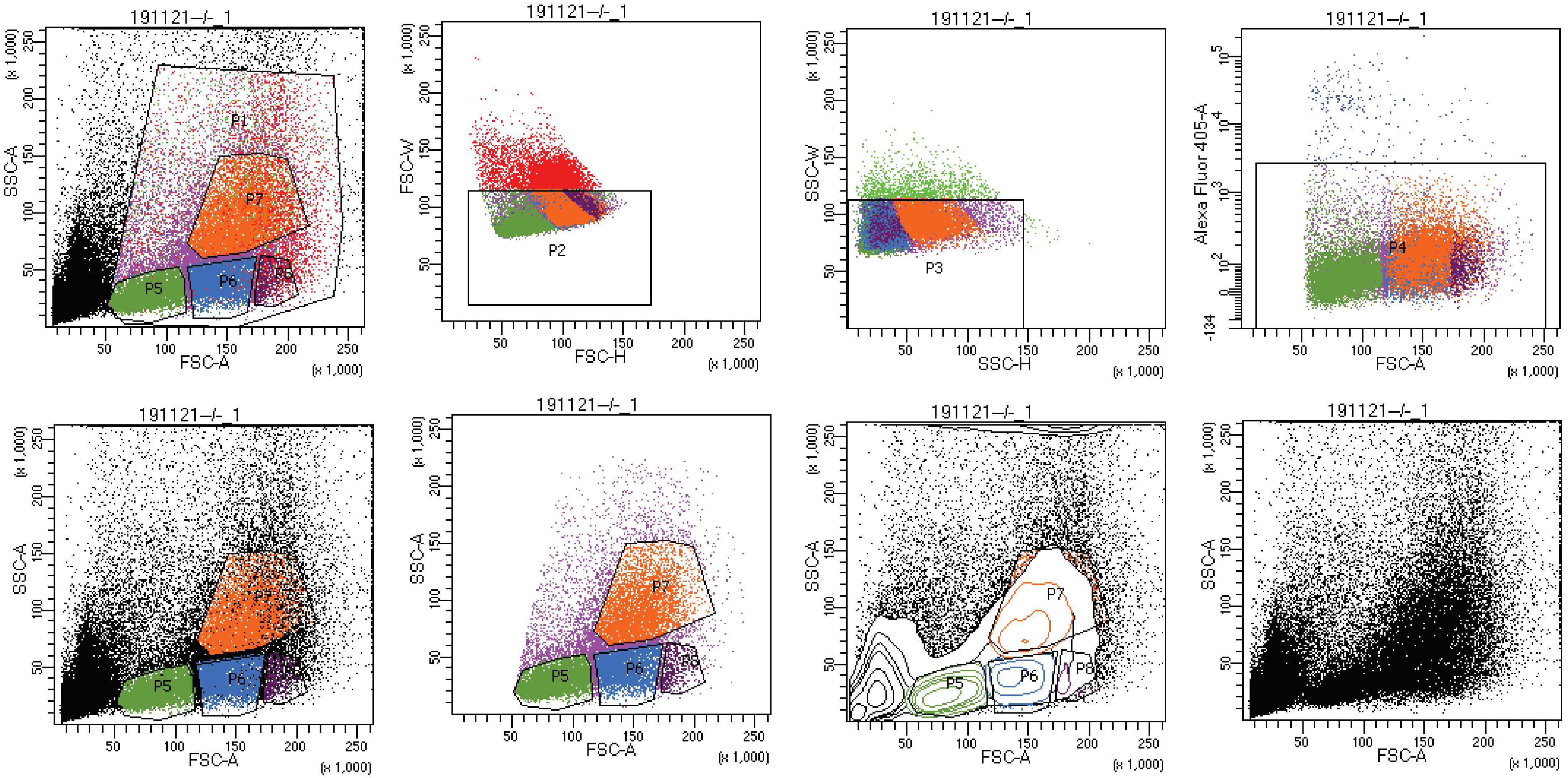

Tube: -/-_1

Population	#Events	%Parent	%Total
All Events	50,000	####	100.0
P1	28,607	57.2	57.2
P2	21,784	76.1	43.6
P3	20,371	93.5	40.7
P4	20,134	98.8	40.3
P5	7,503	37.3	15.0
P6	3,471	17.2	6.9
P7	4,862	24.1	9.7
P8	555	2.8	1.1
