# Supplementary material for: Generation of a transparent killifish line through multiplex CRISPR/Cas9mediated gene inactivation
Source: eLife. 2023 Feb 23;12:e81549. doi: 10.7554/eLife.81549 (PMC10010688; doi:10.7554/eLife.81549)
Supplement: Figure 4—figure supplement 1—source data 1. [file elife-81549-fig4-figsupp1-data1.zip › Figure_4_figure_supplement_1_source_data/Figure_4_figure_supplement_1_panel_BCD_source_data/Figure_4_figure_supplement_1_panel_bcd_source_data.pptx]

## Slide 1
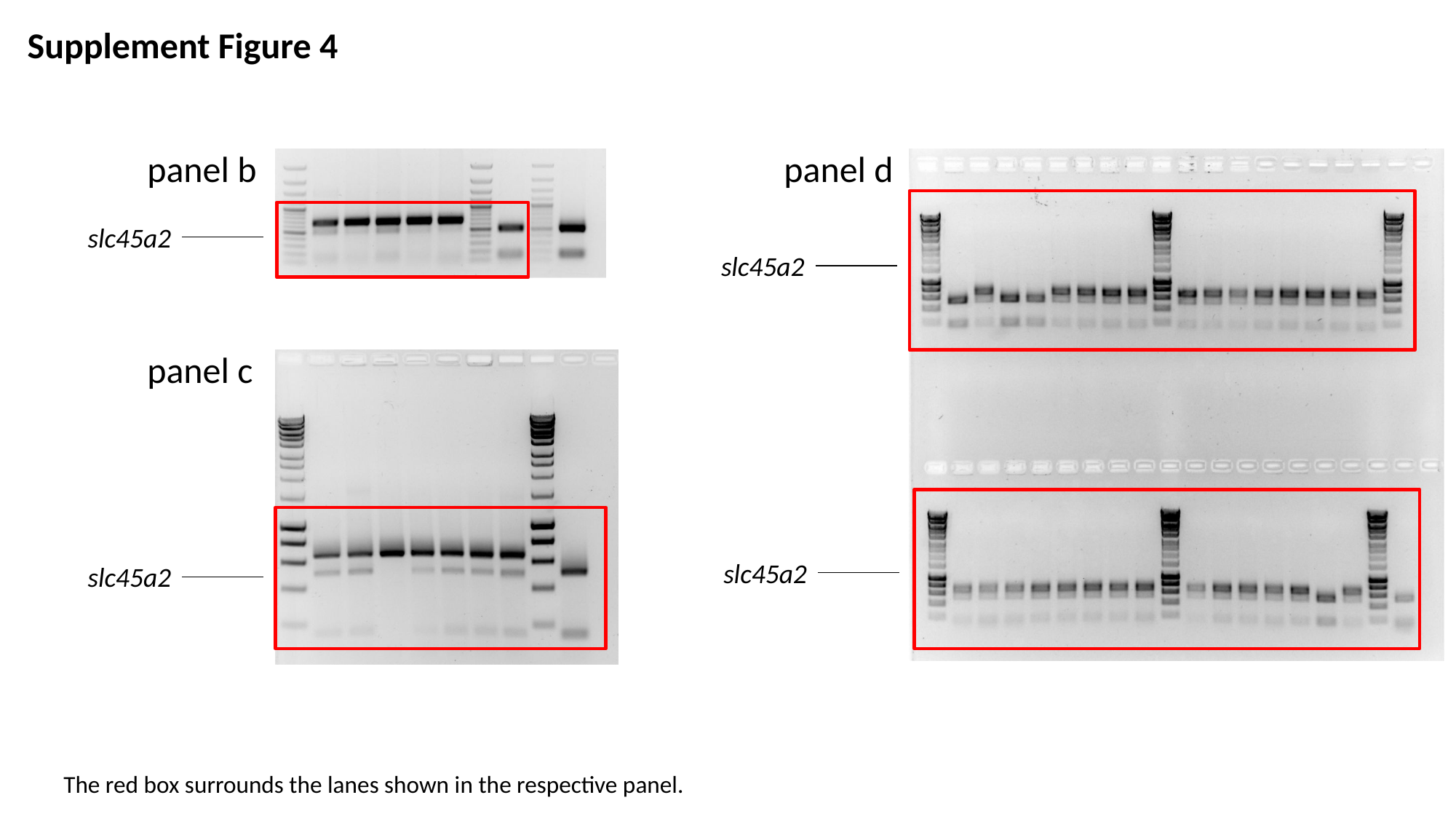

Supplement Figure 4
panel d
panel b
slc45a2
slc45a2
panel c
slc45a2
slc45a2
The red box surrounds the lanes shown in the respective panel.
