# Supplementary material for: Generation of a transparent killifish line through multiplex CRISPR/Cas9mediated gene inactivation
Source: eLife. 2023 Feb 23;12:e81549. doi: 10.7554/eLife.81549 (PMC10010688; doi:10.7554/eLife.81549)

Clip. 1 BQ 20 WL 10 Sequence: #2

Clipped length:	612	Samples:	15898
Left clip:	18	Bases:	630
Right clip:	629	Average spacing:	26.0
Avg. qual. in clip.:	49.18	Average quality >=	10: 43, 20: 88, 30: 482

Quality: 0 - 9, 10 - 19, 20 - 29, >= 30

15.01.2020

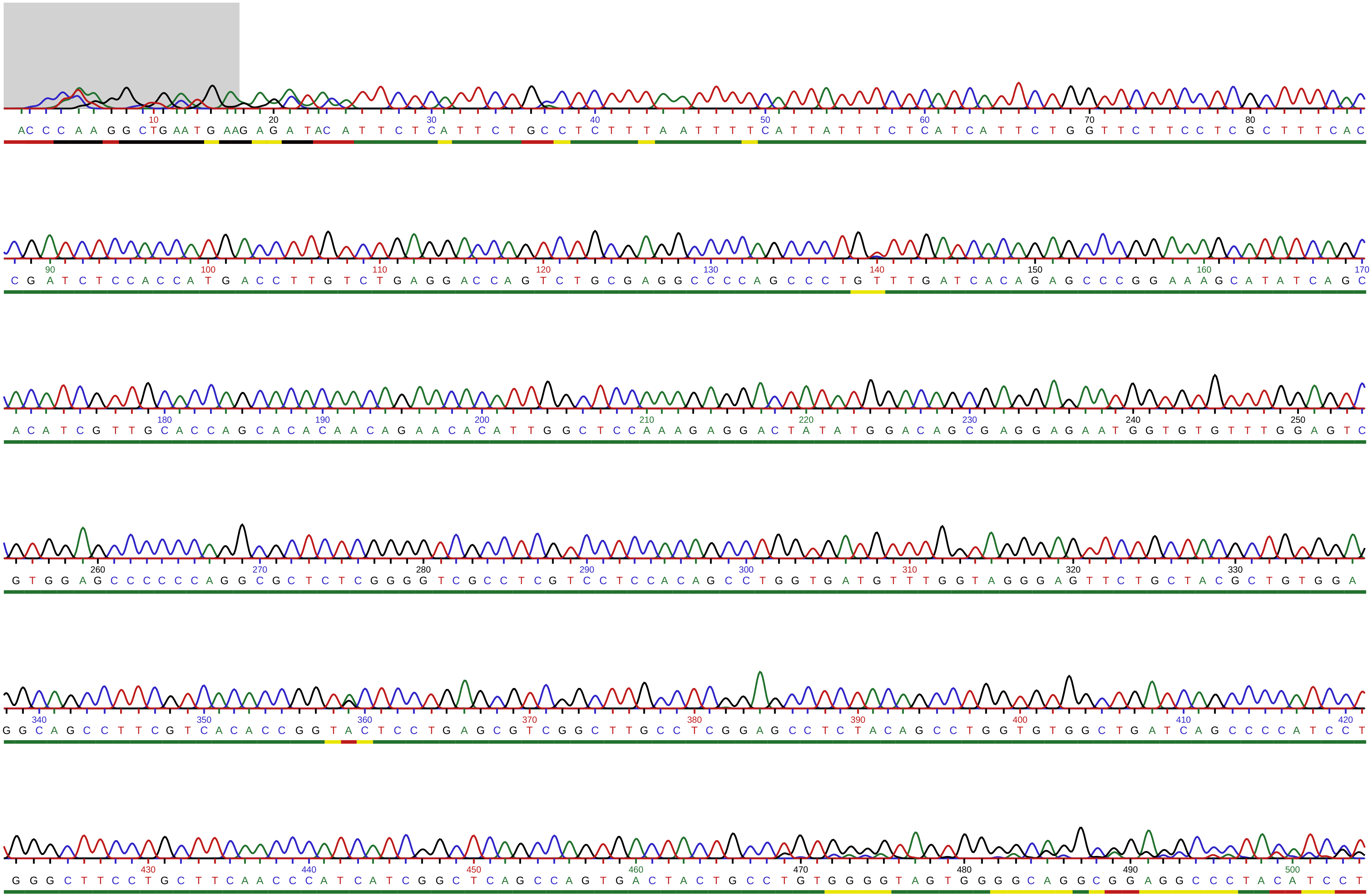

Clip. 1 BQ 20 WL 10 Sequence: #2

Clipped length: 612
Left clip: 18
Right clip: 629
Avg. qual. in clip.: 49.18

Samples: 15898
Bases: 630
Average spacing: 26.0
Average quality >= 10: 43, 20: 88, 30: 482

Quality: 0 - 9
10 - 19
20 - 29
>= 30

15.01.2020

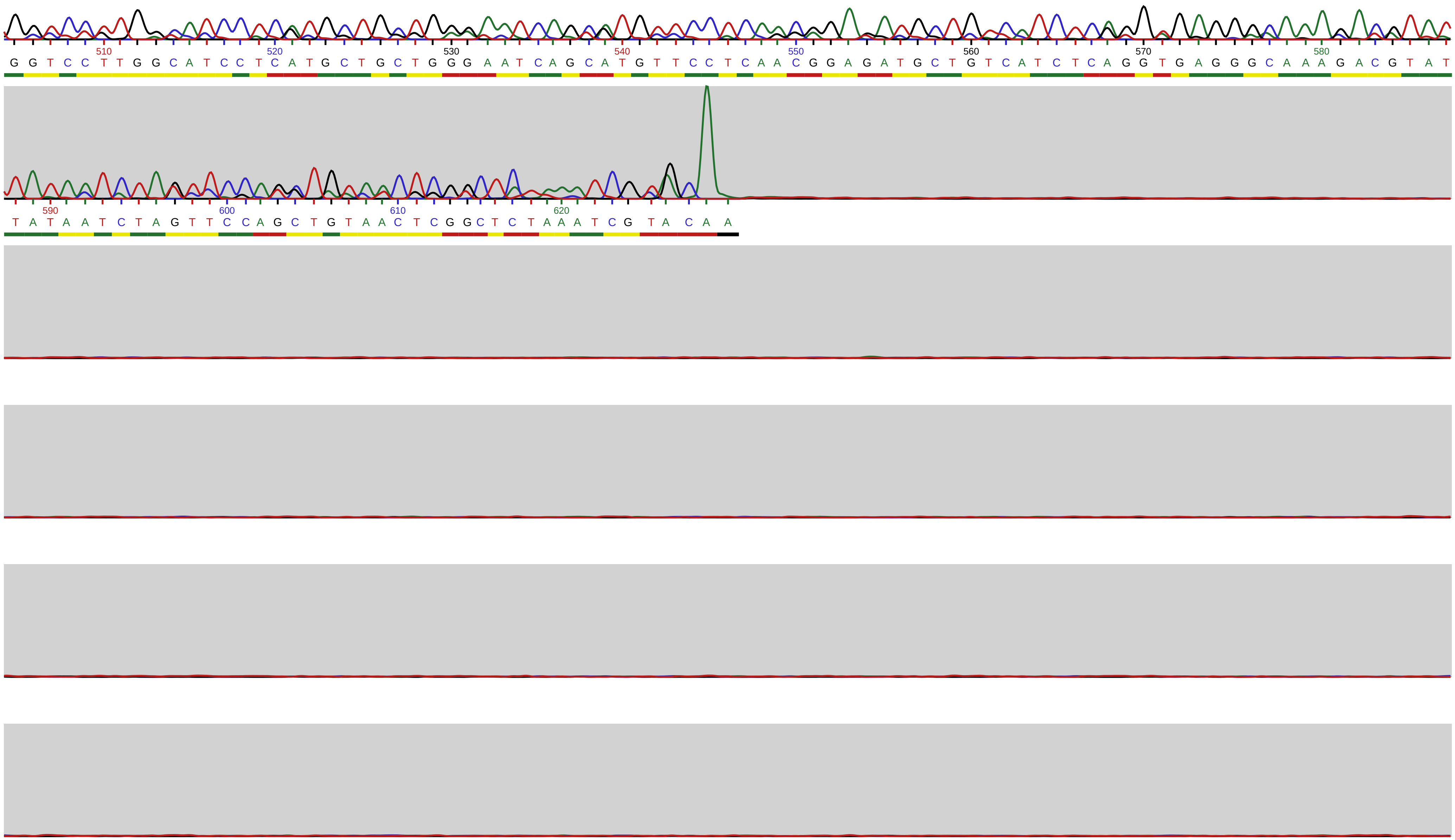

Clip. 1 BQ 20 WL 10 Sequence: #2

Clipped length:	612	Samples:	15898	Quality:	0 - 9	
Left clip:	18	Bases:	630		10 - 19	
Right clip:	629	Average spacing:	26.0		20 - 29	
Avg. qual. in clip.:	49.18	Average quality >=	10: 43, 20: 88, 30: 482		>= 30	

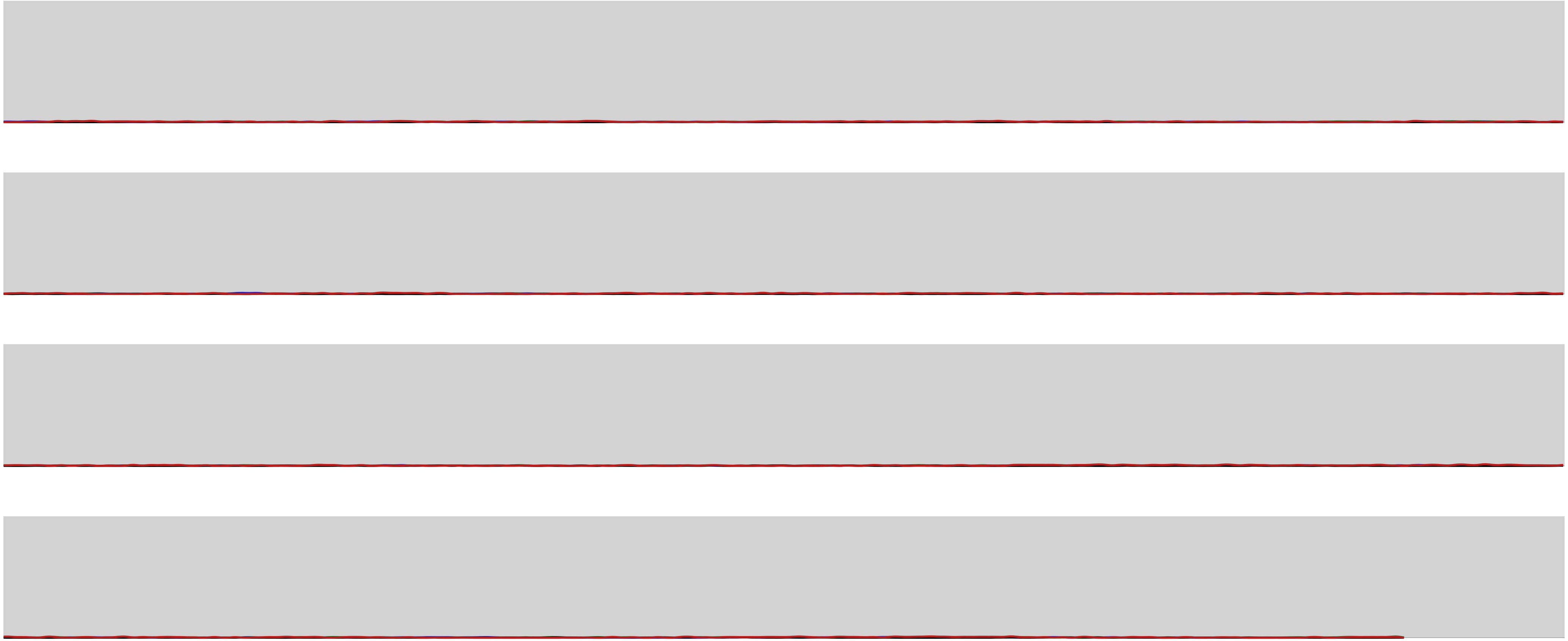

Supplement: Figure 4—figure supplement 1—source data 1. [file elife-81549-fig4-figsupp1-data1.zip › Figure_4_figure_supplement_1_source_data/Figure_4_figure_supplement_1_panel_E_source_data/Originals_Sequencing_data/Fish_2/#2.pdf]
