# Supplementary material for: Generation of a transparent killifish line through multiplex CRISPR/Cas9mediated gene inactivation
Source: eLife. 2023 Feb 23;12:e81549. doi: 10.7554/eLife.81549 (PMC10010688; doi:10.7554/eLife.81549)

Clip. 1 BQ 20 WL 10 Sequence: #29

Clipped length:	611	Samples:	14231	Quality:	0 - 9
Left clip:	21	Bases:	632		10 - 19
Right clip:	631	Average spacing:	23.0		20 - 29
Avg. qual. in clip.:	50.82	Average quality >=	10: 27, 20: 72, 30: 519		>= 30

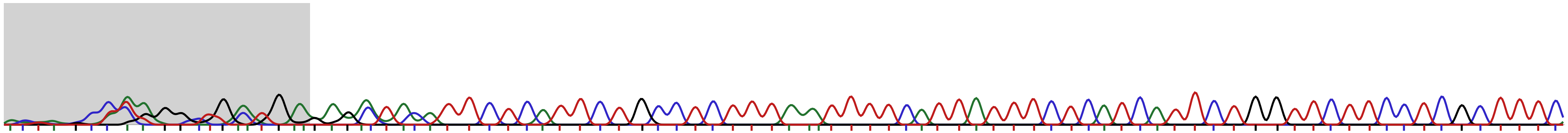

10 20 30 40 50 60 70 80 90

ACTAGCCAAGGCTGAATGAAGAGACTACATTCTCATTCTGCCTCTTTAAATTTTCATTATTTCTCATCATTCTGGTTCTTCCTCGCTTTC

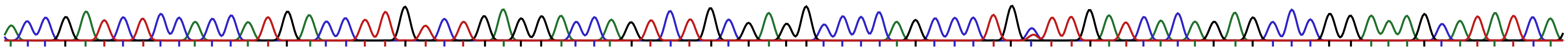

100 110 120 130 140 150 160 170

ACCGATCTCCACCATGACCTTGTCTGAGGACCAGTCTGCGAGGCCCCAGCCCTGCTTGATCACAGAGCCCGGAAAGCATATCA

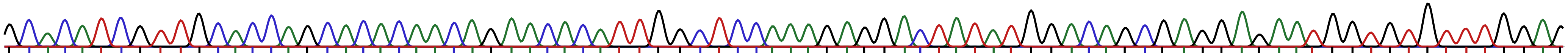

180 190 200 210 220 230 240 250

GCACATCGTTGCACCAGCACACAACAGAACACATTGGCTCCAAAGAGGACTATATGGACAGCGAGGAGAATGGTGTGTTTGGAG

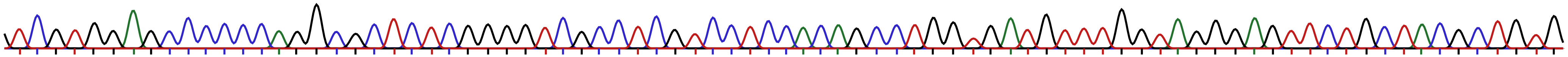

260 270 280 290 300 310 320 330 340

TCGTGGAGCCCCCAGGCGCTCTCGGGGTCGCCTCGTCCTCCACAGCCTGGTGATGTTTGGTAGGGAGTTCTGCTACGCTGTG

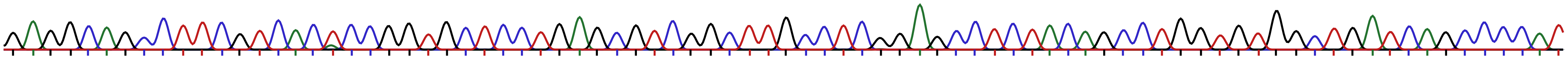

350 360 370 380 390 400 410 420

GAGGCAGCCTTCGTCACTCCGGTGCTCCTGAGCGTCGGCTTGCCTCGGAGCCTCTACAGCCTGGTGTGGCTGATCAGCCCCAT

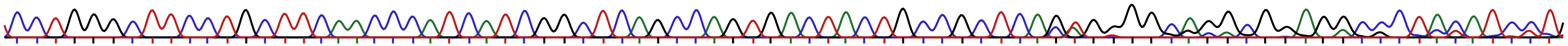

430 440 450 460 470 480 490 500

CCTGGGCTTCCTGCTTCAACCCATCATCGGCTCAGCCAGTGACTACTGCCGCTCAGTGGGCAGGCGGAGGCCCTACATCCT

Clip. 1 BQ 20 WL 10 Sequence: #29

Clipped length:	611	Samples:	14231	Quality: 0 - 9	
Left clip:	21	Bases:	632	10 - 19	
Right clip:	631	Average spacing:	23.0	20 - 29	
Avg. qual. in clip.:	50.82	Average quality >=	10: 27, 20: 72, 30: 519	>= 30	

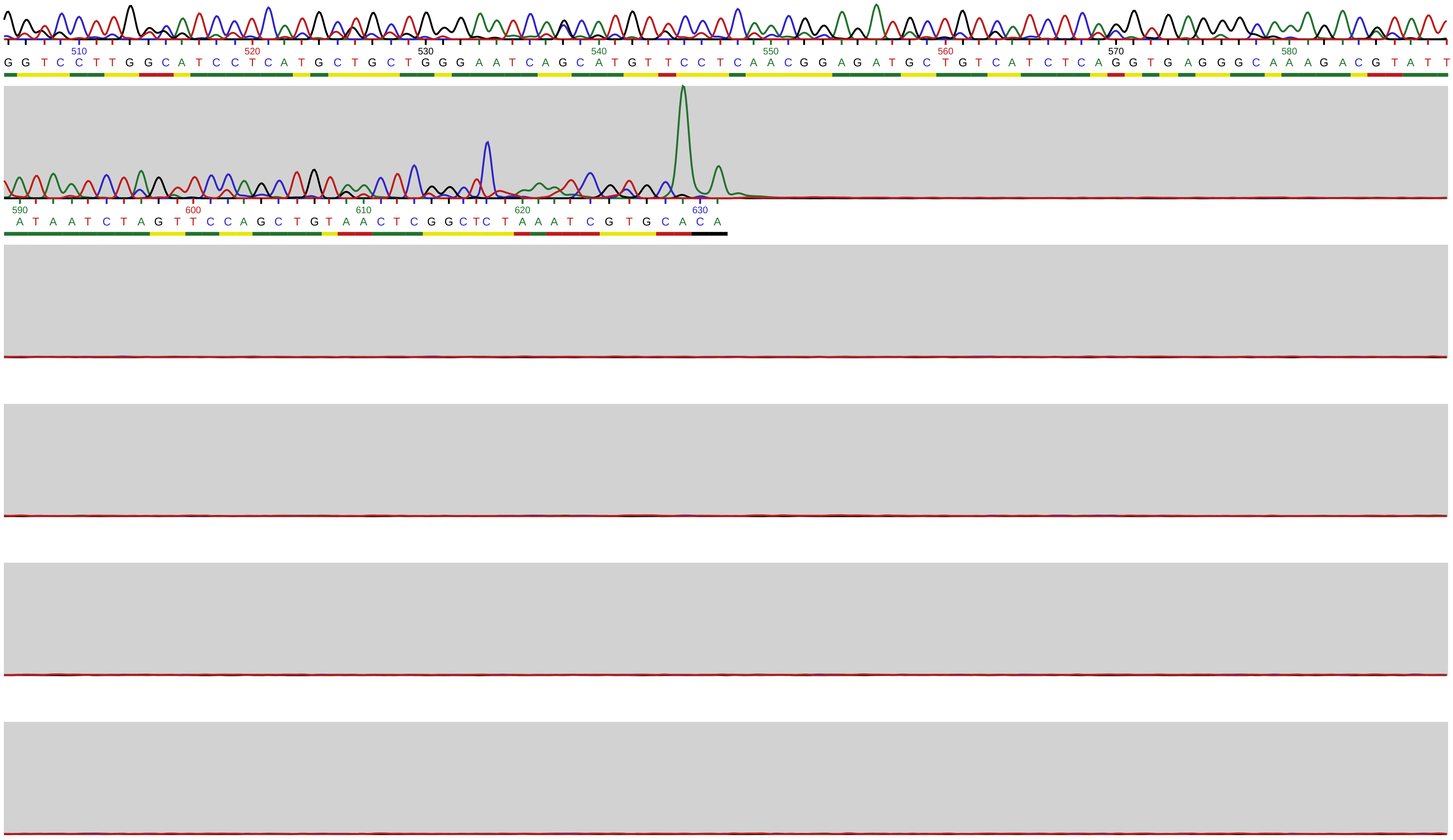

Clip. 1 BQ 20 WL 10 Sequence: #29

Clipped length:	611	Samples:	14231	Quality:	0 - 9	
Left clip:	21	Bases:	632		10 - 19	
Right clip:	631	Average spacing:	23.0		20 - 29	
Avg. qual. in clip.:	50.82	Average quality >=	10: 27, 20: 72, 30: 519		>= 30	

15.01.2020

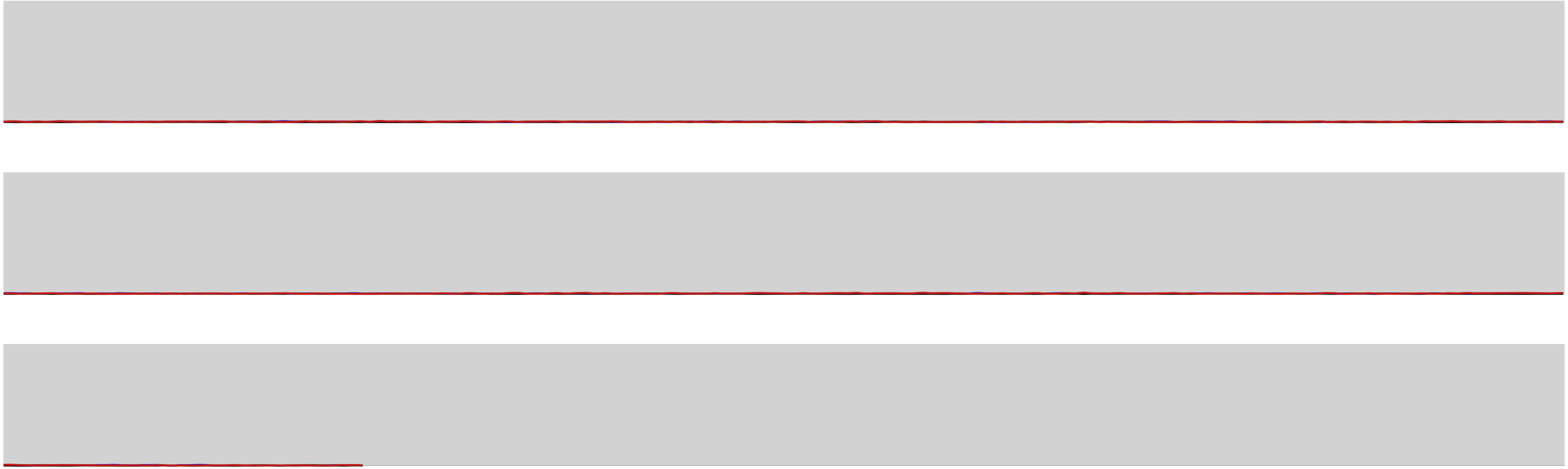

Supplement: Figure 4—figure supplement 1—source data 1. [file elife-81549-fig4-figsupp1-data1.zip › Figure_4_figure_supplement_1_source_data/Figure_4_figure_supplement_1_panel_E_source_data/Originals_Sequencing_data/Fish_29/#29.pdf]
