# Supplementary material for: Generation of a transparent killifish line through multiplex CRISPR/Cas9mediated gene inactivation
Source: eLife. 2023 Feb 23;12:e81549. doi: 10.7554/eLife.81549 (PMC10010688; doi:10.7554/eLife.81549)
Supplement: Figure 4—figure supplement 1—source data 1. [file elife-81549-fig4-figsupp1-data1.zip › Figure_4_figure_supplement_1_source_data/Figure_4_figure_supplement_1_panel_E_source_data/Originals_Sequencing_data/slc45a2_F1_seq.docx]

**Sequencing slc45a2_F1_fish**

>WT

*GGATTTGGTGTTTTGGCCCT*ATATCCTTCACCGGGCTGACTGAGAGACTACATTCTCATTCTGCCTCTTTAAATTTTCATTATTTCTCATCATTCTGGTTCTTCCTCGCTTTCACCGATCTCCACC**ATG**ACCTTGTCTGAGGACCAGTCTGCGAGGCCCCAGCCCTGCTTGATCACAGAGCCCGGAAAGCATATCAGCACATCGTTGCACCAGCACACAACAGAACACATTGGCTTCAAAGAGGACTATATGGACAGCGAGGAGAATGGTGTGTTTGGAGTCGTGGAGCCCCCCAGGCGCTCTCGGGGTCGCCTCGTCCTCCACAGCCTGGTGATGTTTGGTAGGGAGTTCTGCTACGCTGTGGAGGCAGCCTTCGTCACACCGGTACTCCTGAGCGTCGGCTTGCCTCGGAGCCTCTACAGCCTGGTGTGGCTGATCAGCCCCATCCTGGGCTTCCTGCTTCAACCCATCATCGGCTCAGCCAGTGACTACTGCCGCTCACAGTGGGGCAGGCGGAGGCCCTACATCCTGGTCCTTGGCATCCTCATGCTGCTGGGAATCAGCATGTTCCTCAACGGAGATGCTGTCATCTCAGGTGAGGGCAAAGACGTATTATAATCTAGTTCCAGCT*GTAACTCGGCTCTAATCGTGC*

>#2

GAGATACATTCTCATTCTGCCTCTTTAATTTTCATTATTTCTCATCATTCTGGTTCTTCCTCGCTTTCACCGATCTCCACCATGACCTTGTCTGAGGACCAGTCTGCGAGGCCCCAGCCCTGTTTGATCACAGAGCCCGGAAAGCATATCAGCACATCGTTGCACCAGCACACAACAGAACACATTGGCTCCAAAGAGGACTATATGGACAGCGAGGAGAATGGTGTGTTTGGAGTCGTGGAGCCCCCCAGGCGCTCTCGGGGTCGCCTCGTCCTCCACAGCCTGGTGATGTTTGGTAGGGAGTTCTGCTACGCTGTGGAGGCAGCCTTCGTCACACCGGTACTCCTGAGCGTCGGCTTGCCTCGGAGCCTCTACAGCCTGGTGTGGCTGATCAGCCCCATCCTGGGCTTCCTGCTTCAACCCATCATCGGCTCAGCCAGTGACTACTGCCTGTGGGGTAGTGGGGCAGGCGGAGGCCCTACATCCTGGTCCTTGGCATCCTCATGCTGCTGGGAATCAGCATGTTCCTCAACGGAGATGCTGTCATCTCAGGTGAGGGCAAAGACGTATTATAATCTAGTTCCAGCTGTAACTCGGCTCTAAATCGTACAA

>#9

GAGACTACATTCTCATTCTGCCTCTTTAAATTTTCATTATTTCTCATCATTCTGGTTCTTCCTCGCTTTCACCGATCTCCACCATGACCTTGTCTGAGGACCAGTCTGCGAGGCCCCAGCCCTGCTTGATCACAGAGCCCGGAAAGCATATCAGCACATCGTTGCACCAGCACACAACAGAACACATTGGCTCCAAAGAGGACTATATGGACAGCGAGGAGAATGGTGTGTTTGGAGTCGTGGAGCCCCCCAGGCGCTCTCGGGGTCGCCTCGTCCTCCACAGCCTGGTGATGTTTGGTAGGGAGTTCTGCTACGCTGTGGAGGCAGCCTTCGTCACTCCGGTGCTCCTGAGCGTCGGCTTGCCTCGGAGCCTCTACAGCCTGGTGTGGCTGATCAGCCCCATCCTGGGCTTCCTGCTTCAACCCATCATCGGCTCAGCCAGTGACTACTGCTGAGGGGGCAGGCGGAGGCCCTACATCCTGGTCCTTGGCATCCTCATGCTGCTGGGAATCAGCATGTTCCTCAACGGAGATGCTGTCATCTCAGGTGAGGGCAAAGACGTATTATAATCTAGTTCCAGCTGTAACTCGGCTTAATCGTGCACGTGCA

>#29

GAGACTACATTCTCATTCTGCCTCTTTAAATTTTCATTATTTCTCATCATTCTGGTTCTTCCTCGCTTTCACCGATCTCCACCATGACCTTGTCTGAGGACCAGTCTGCGAGGCCCCAGCCCTGCTTGATCACAGAGCCCGGAAAGCATATCAGCACATCGTTGCACCAGCACACAACAGAACACATTGGCTCCAAAGAGGACTATATGGACAGCGAGGAGAATGGTGTGTTTGGAGTCGTGGAGCCCCCCAGGCGCTCTCGGGGTCGCCTCGTCCTCCACAGCCTGGTGATGTTTGGTAGGGAGTTCTGCTACGCTGTGGAGGCAGCCTTCGTCACTCCGGTGCTCCTGAGCGTCGGCTTGCCTCGGAGCCTCTACAGCCTGGTGTGGCTGATCAGCCCCATCCTGGGCTTCCTGCTTCAACCCATCATCGGCTCAGCCAGTGACTACTGCCGCTCAGTGGGGCAGGCGGAGGCCCTACATCCTGGTCCTTGGCATCCTCATGCTGCTGGGAATCAGCATGTTCCTCAACGGAGATGCTGTCATCTCAGGTGAGGGCAAAGACGTATTATAATCTAGTTCCAGCTGTAACTCGGCTCTAAATCGTGCACA
