# Supplementary material for: Generation of a transparent killifish line through multiplex CRISPR/Cas9mediated gene inactivation
Source: eLife. 2023 Feb 23;12:e81549. doi: 10.7554/eLife.81549 (PMC10010688; doi:10.7554/eLife.81549)
Supplement: Figure 5—source data 1. [file elife-81549-fig5-data1.zip › Figure_5_source_data/Figure_5_panel_I_source_data/FACS_data_analysis/kidney_female-Batch_Analysis_23012023124322.pdf]

BD FACSDiva 8.0

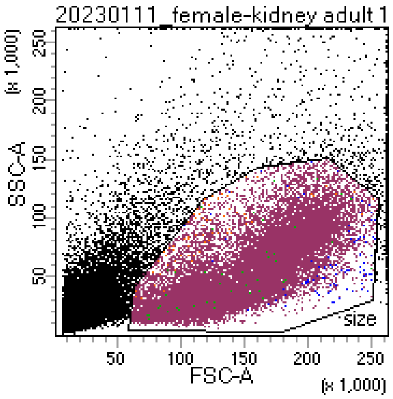

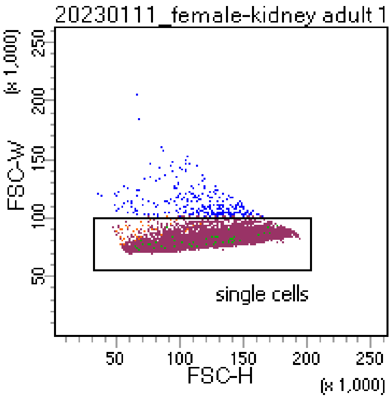

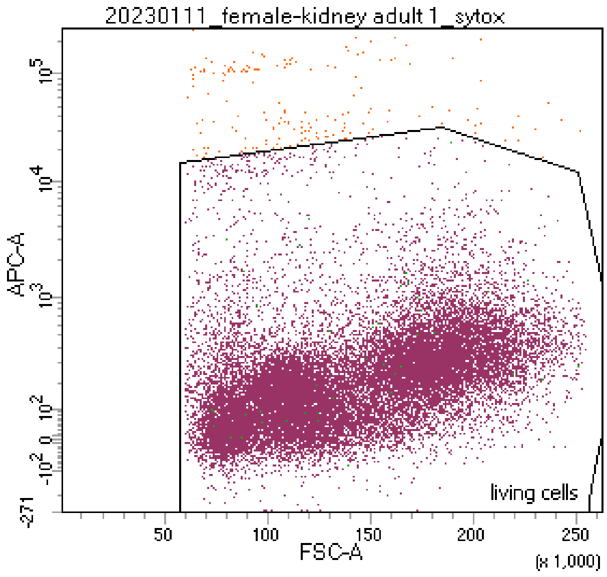

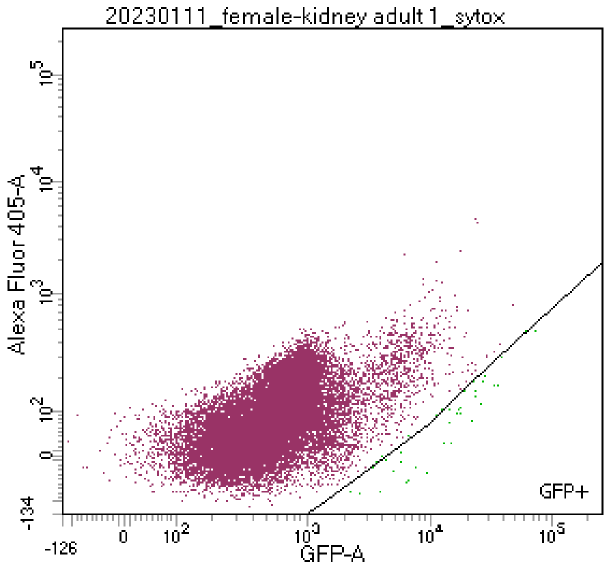

Tube: kidney adult 1_sytox

Population	#Events	%Parent	%Total
All Events	48,620	####	100.0
size	23,828	49.0	49.0
single cells	23,566	98.9	48.5
living cells	23,393	99.3	48.1
GFP+	42	0.2	0.1

Experiment Name: Johannes_Notho kidney
Specimen Name: 20230111_female
Tube Name: kidney adult 1_sytox

Population	#Events	%Parent	%Total	GFP-A Mean	GFP-A %CV
GFP+	42	0.1795	0.0864	16,693	90.9
living cells	23,393	99.2659	48.1139	844	191.1

BD FACSDiva 8.0

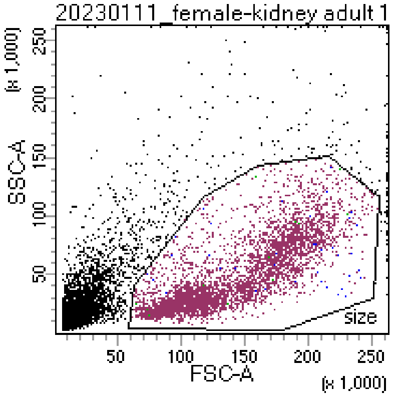

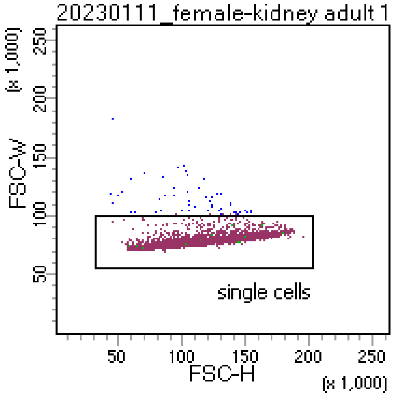

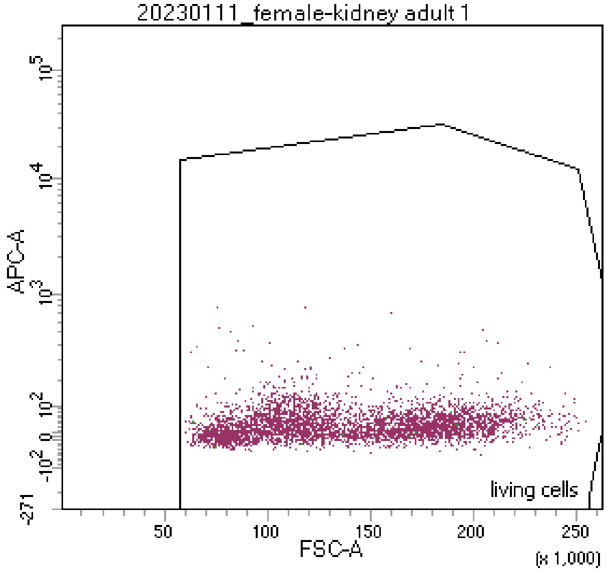

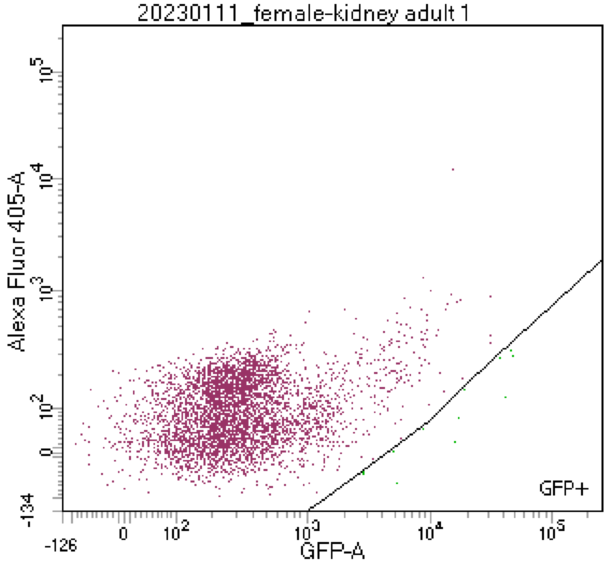

Tube: kidney adult 1

Population	#Events	%Parent	%Total
All Events	8,320	####	100.0
size	4,211	50.6	50.6
single cells	4,162	98.8	50.0
living cells	4,162	100.0	50.0
GFP+	12	0.3	0.1

Experiment Name: Johannes_Notho kidney
Specimen Name: 20230111_female
Tube Name: kidney adult 1

Population	#Events	%Parent	%Total	GFP-A Mean	GFP-A %CV
GFP+	12	0.2883	0.1442	20,714	85.0
living cells	4,162	100.0000	50.0240	641	315.2

BD FACSDiva 8.0

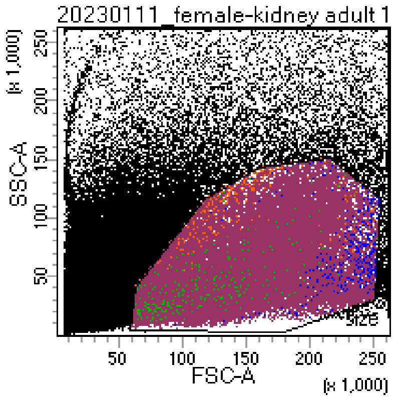

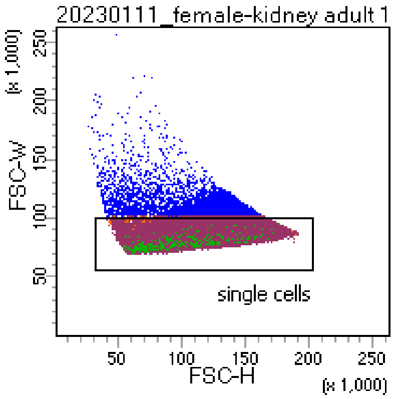

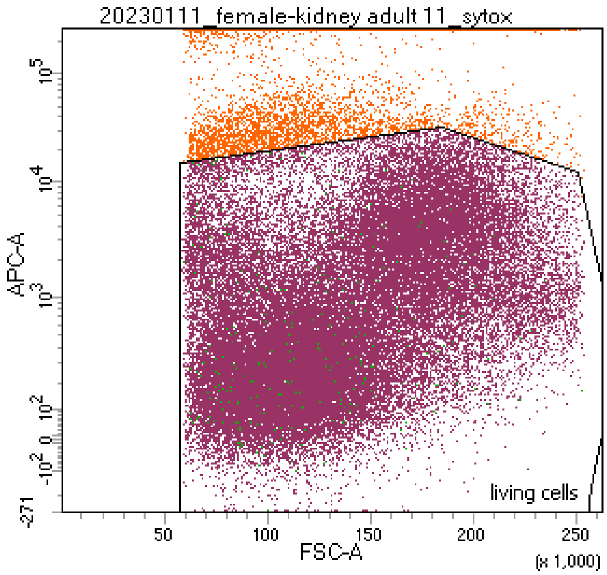

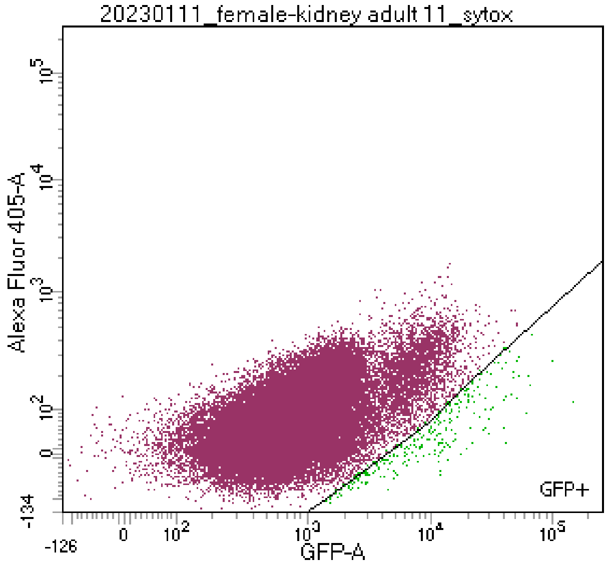

Tube: kidney adult 11_sytox

Population	#Events	%Parent	%Total
All Events	403,516	####	100.0
size	67,528	16.7	16.7
single cells	63,080	93.4	15.6
living cells	56,420	89.4	14.0
GFP+	270	0.5	0.1

Experiment Name: Johannes_Notho kidney
Specimen Name: 20230111_female
Tube Name: kidney adult 11_sytox

Population	#Events	%Parent	%Total	GFP-A Mean	GFP-A %CV
GFP+	270	0.4786	0.0669	14,370	115.2
living cells	56,420	89.4420	13.9821	1,397	176.2

BD FACSDiva 8.0

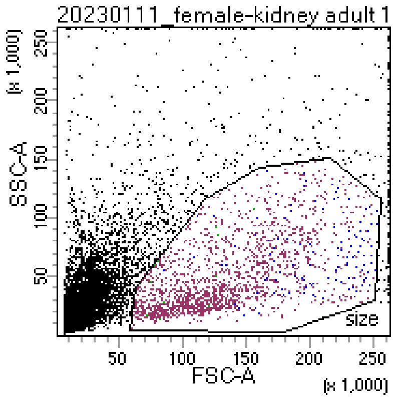

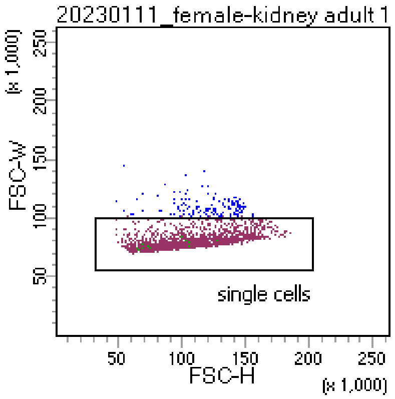

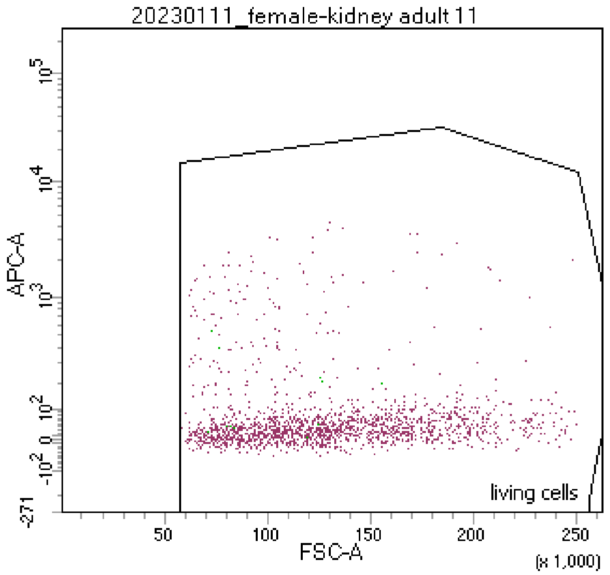

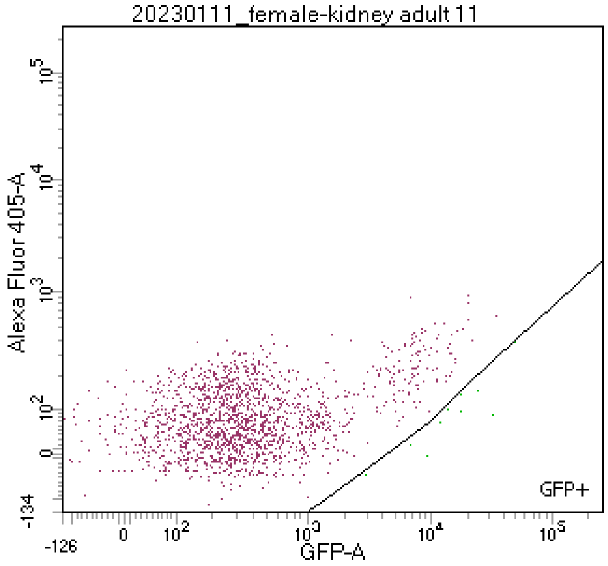

Tube: kidney adult 11

Population	#Events	%Parent	%Total
All Events	10,993	####	100.0
size	1,763	16.0	16.0
single cells	1,624	92.1	14.8
living cells	1,624	100.0	14.8
GFP+	11	0.7	0.1

Experiment Name: Johannes_Notho kidney
Specimen Name: 20230111_female
Tube Name: kidney adult 11

Population	#Events	%Parent	%Total	GFP-A Mean	GFP-A %CV
GFP+	11	0.6773	0.1001	17,036	82.0
living cells	1,624	100.0000	14.7730	1,090	273.5

BD FACSDiva 8.0

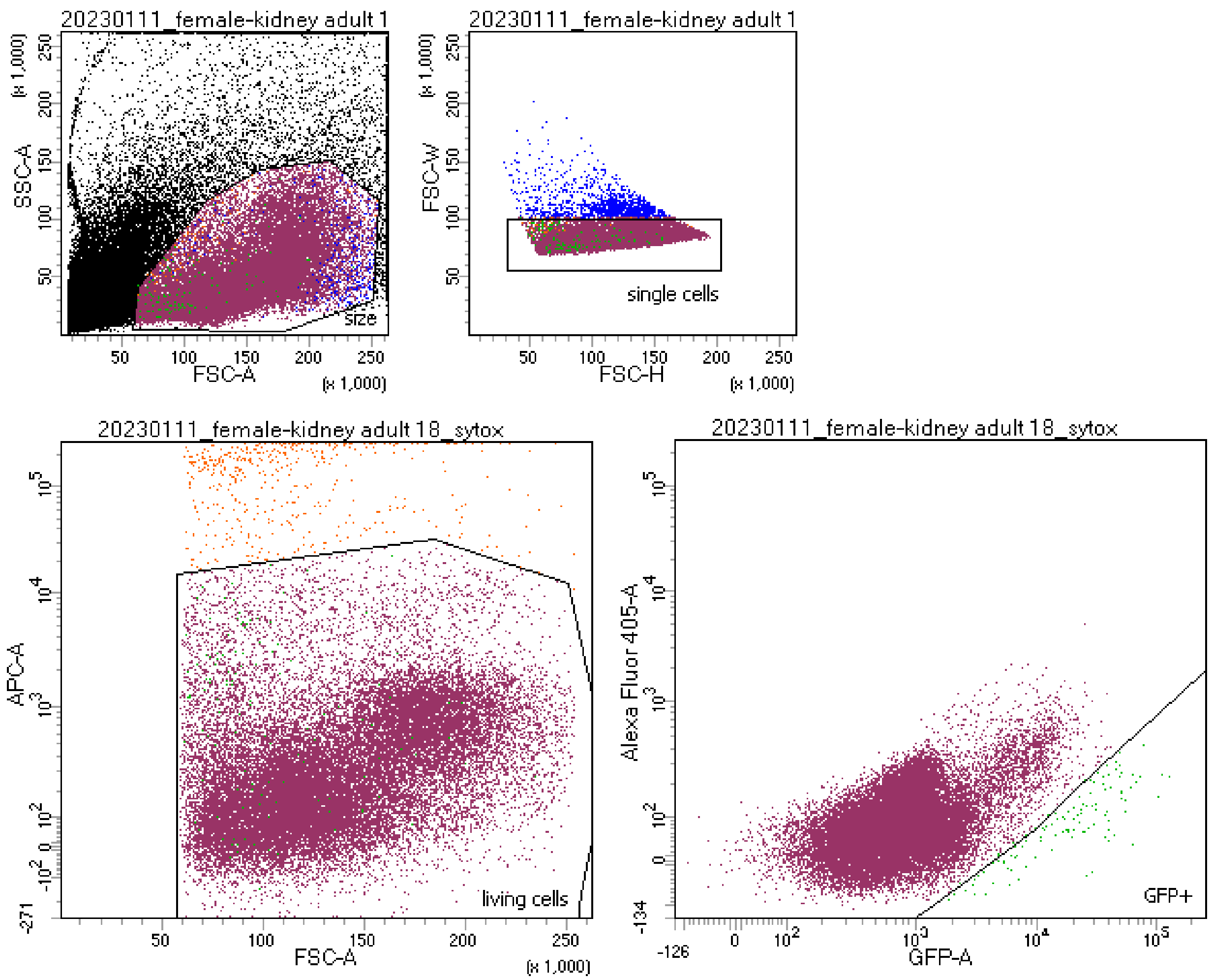

Tube: kidney adult 18_sytox

Population	#Events	%Parent	%Total
All Events	104,853	####	100.0
size	32,188	30.7	30.7
single cells	30,995	96.3	29.6
living cells	30,545	98.5	29.1
GFP+	120	0.4	0.1

Experiment Name: Johannes_Notho kidney
Specimen Name: 20230111_female
Tube Name: kidney adult 18_sytox

Population	#Events	%Parent	%Total	GFP-A Mean	GFP-A %CV
GFP+	120	0.3929	0.1144	25,140	94.0
living cells	30,545	98.5482	29.1313	1,287	224.1

BD FACSDiva 8.0

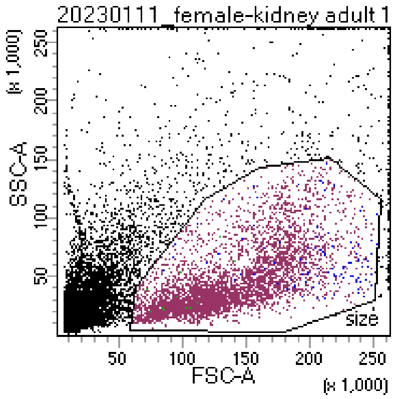

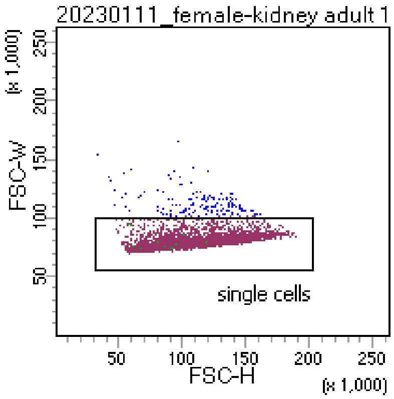

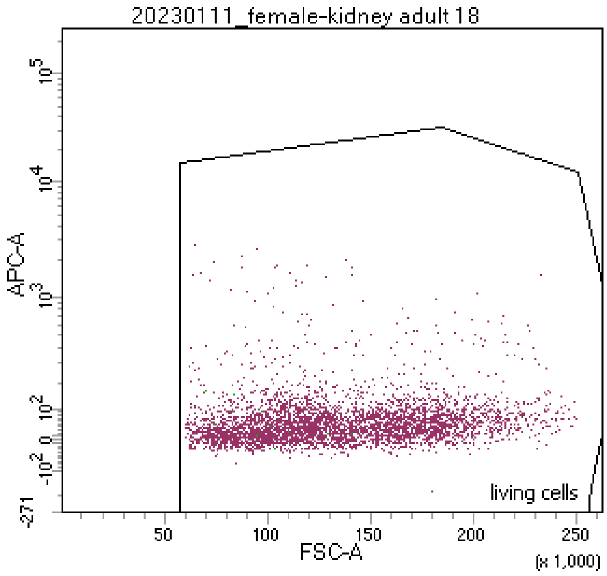

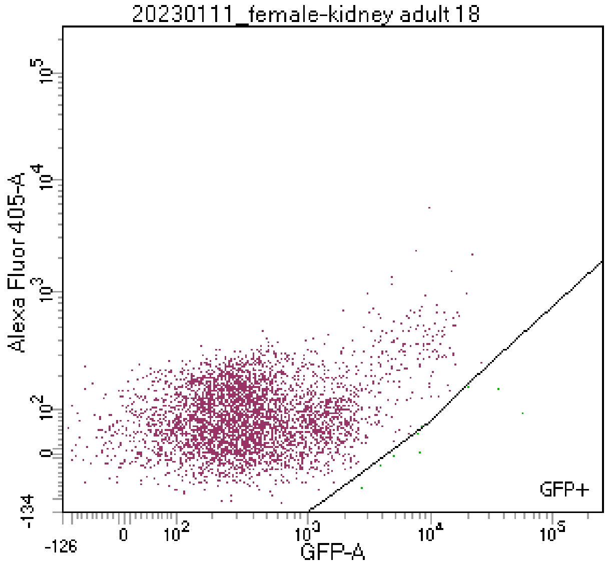

Tube: kidney adult 18

Population	#Events	%Parent	%Total
All Events	14,248	####	100.0
size	4,305	30.2	30.2
single cells	4,168	96.8	29.3
living cells	4,168	100.0	29.3
GFP+	11	0.3	0.1

Experiment Name:	Johannes_Notho kidney
Specimen Name:	20230111_female
Tube Name:	kidney adult 18

Population	#Events	%Parent	%Total	GFP-A Mean	GFP-A %CV
GFP+	11	0.2639	0.0772	15,335	111.6
living cells	4,168	100.0000	29.2532	832	245.8

BD FACSDiva 8.0

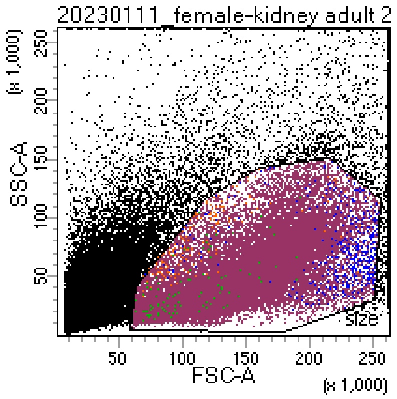

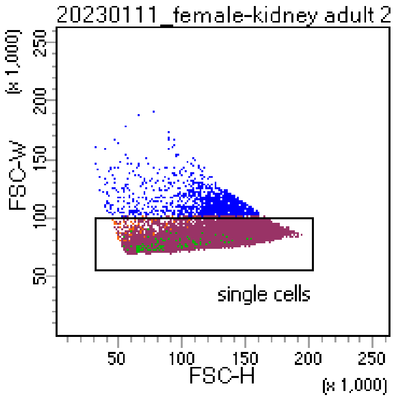

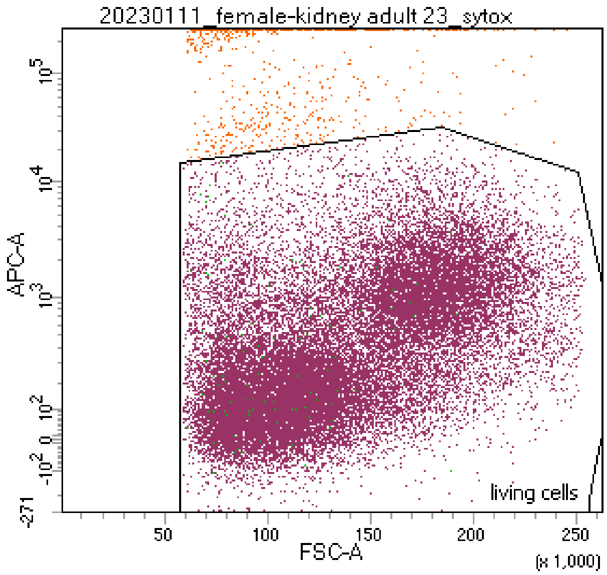

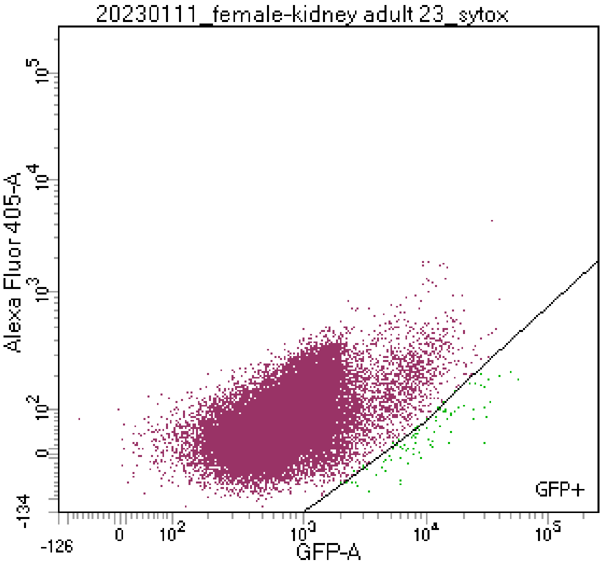

Tube: kidney adult 23_sytox

Population	#Events	%Parent	%Total
All Events	106,760	####	100.0
size	32,970	30.9	30.9
single cells	31,440	95.4	29.4
living cells	30,744	97.8	28.8
GFP+	93	0.3	0.1

Experiment Name: Johannes_Notho kidney
Specimen Name: 20230111_female
Tube Name: kidney adult 23_sytox

Population	#Events	%Parent	%Total	GFP-A Mean	GFP-A %CV
GFP+	93	0.3025	0.0871	12,196	86.2
living cells	30,744	97.7863	28.7973	1,156	153.7

BD FACSDiva 8.0

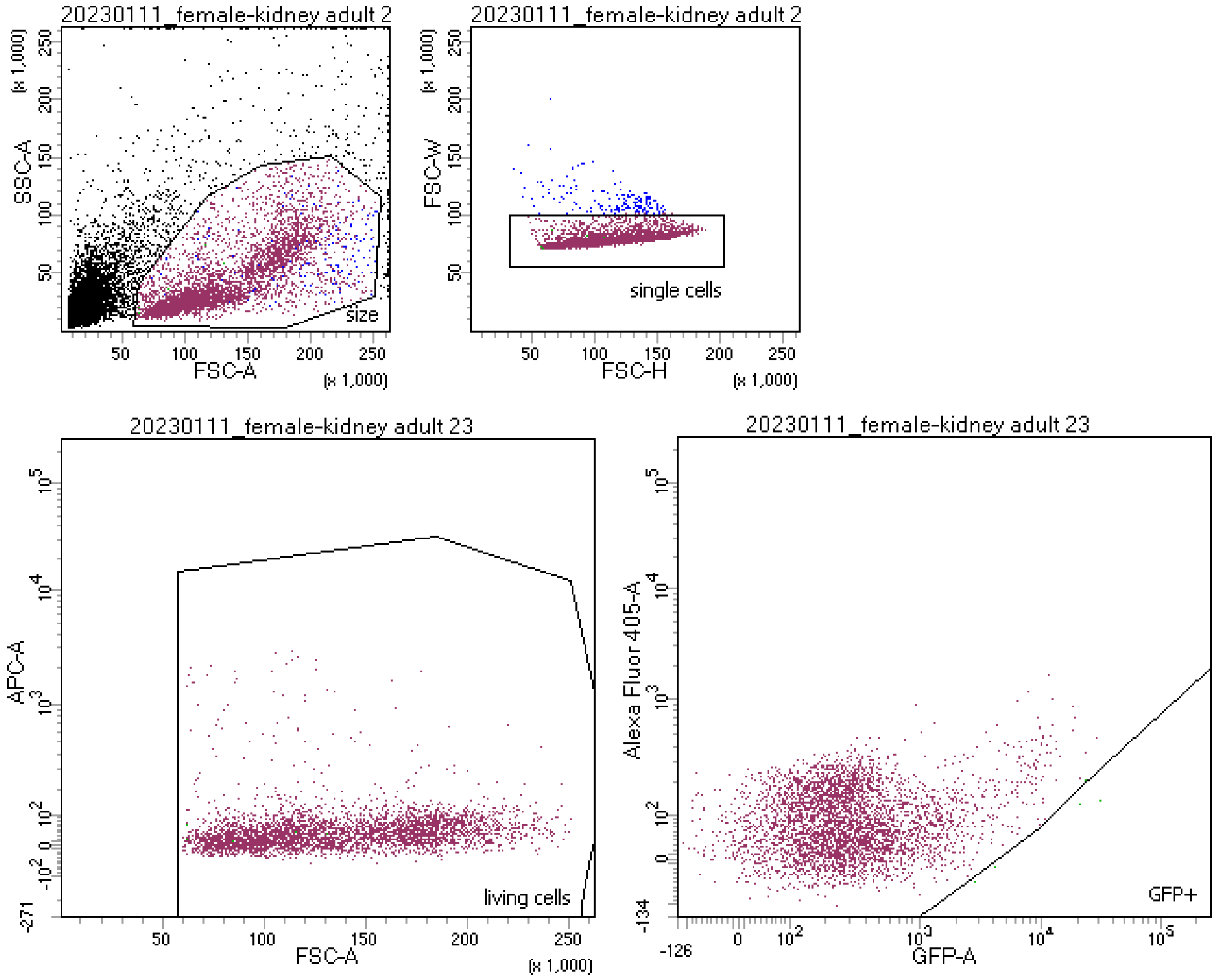

Tube: kidney adult 23

Population	#Events	%Parent	%Total
All Events	12,064	####	100.0
size	4,039	33.5	33.5
single cells	3,870	95.8	32.1
living cells	3,870	100.0	32.1
GFP+	5	0.1	0.0

Experiment Name: Johannes_Notho kidney
Specimen Name: 20230111_female
Tube Name: kidney adult 23

Population	#Events	%Parent	%Total	GFP-A Mean	GFP-A %CV
GFP+	5	0.1292	0.0414	16,473	75.9
living cells	3,870	100.0000	32.0789	652	263.0

BD FACSDiva 8.0

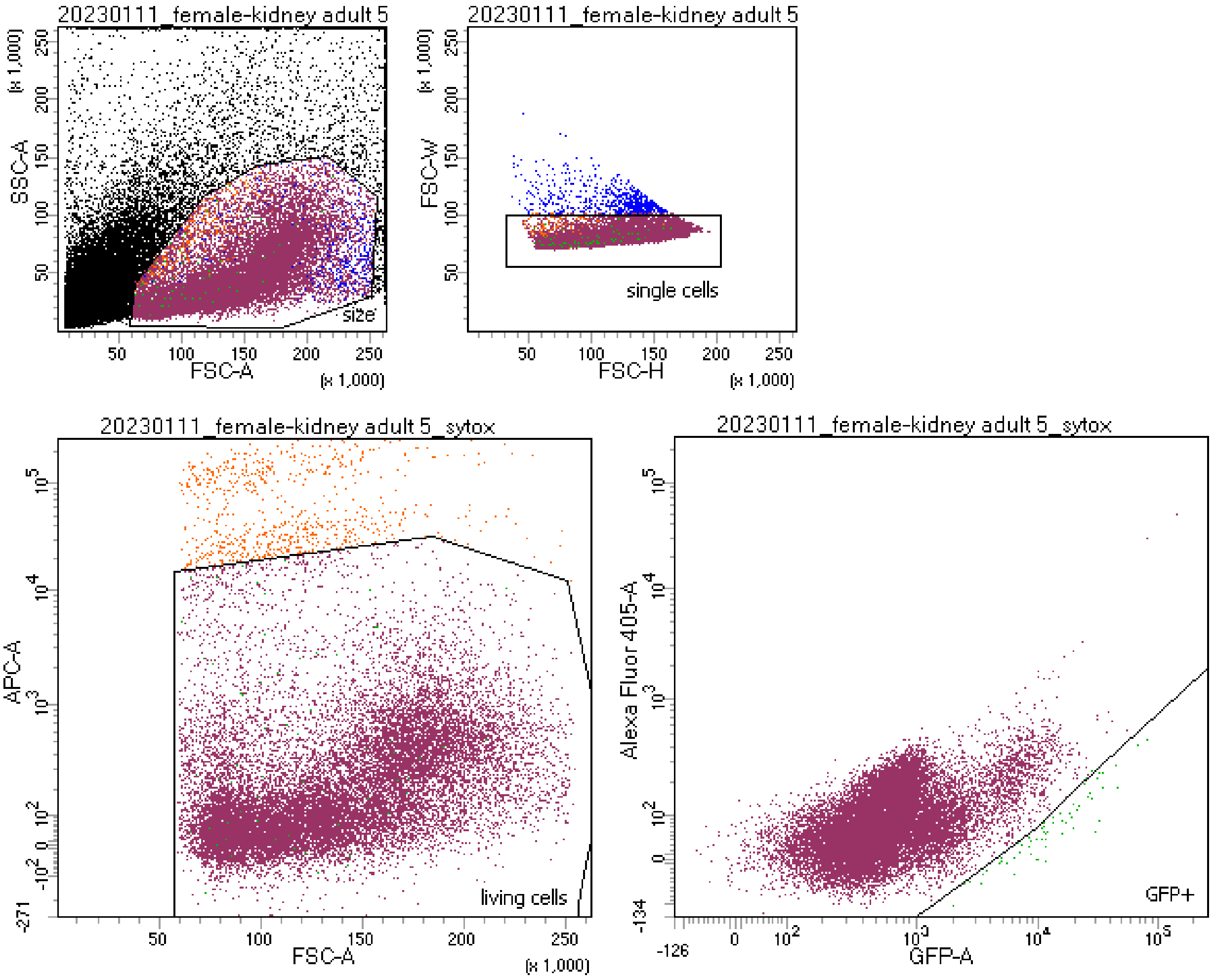

Tube: kidney adult 5_sytox

Population	#Events	%Parent	%Total
All Events	74,856	####	100.0
size	20,584	27.5	27.5
single cells	19,915	96.7	26.6
living cells	19,232	96.6	25.7
GFP+	62	0.3	0.1

Experiment Name:	Johannes_Notho kidney
Specimen Name:	20230111_female
Tube Name:	kidney adult 5_sytox

Population	#Events	%Parent	%Total	GFP-A Mean	GFP-A %CV
GFP+	62	0.3224	0.0828	17,532	86.6
living cells	19,232	96.5704	25.6920	1,097	235.2

BD FACSDiva 8.0

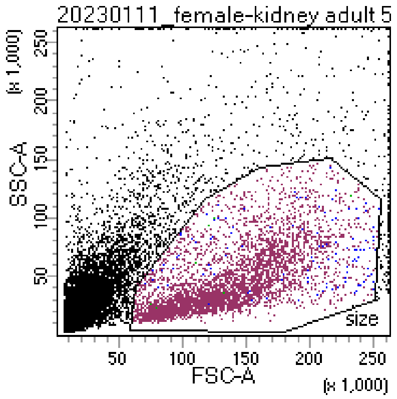

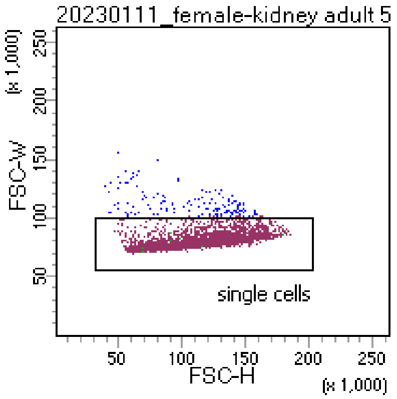

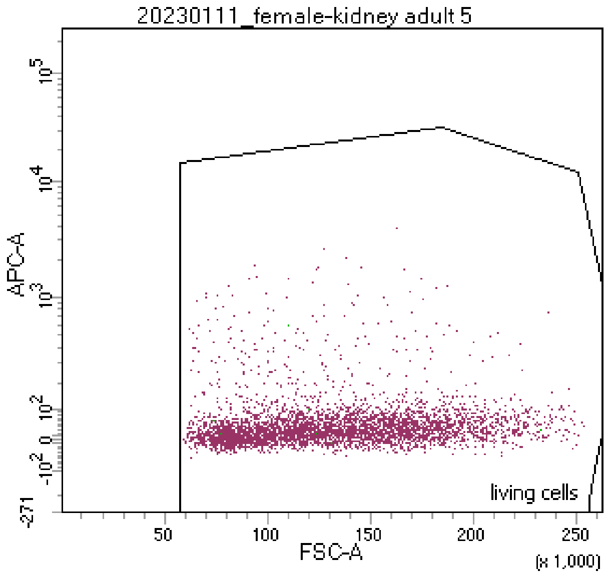

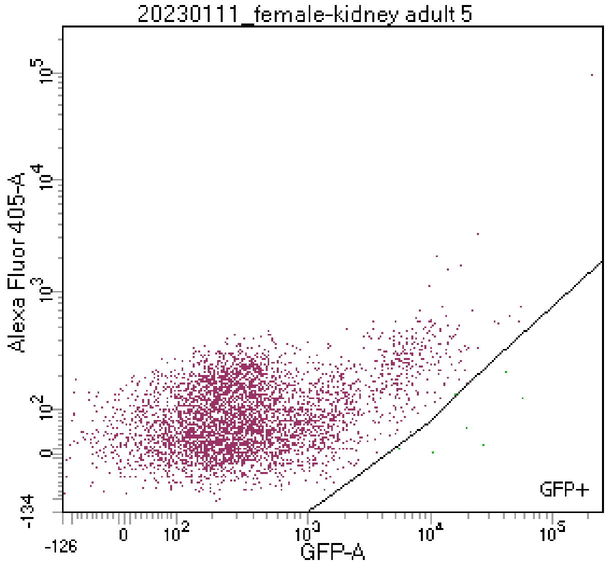

Tube: kidney adult 5

Population	#Events	%Parent	%Total
All Events	15,548	####	100.0
size	4,398	28.3	28.3
single cells	4,252	96.7	27.3
living cells	4,252	100.0	27.3
GFP+	7	0.2	0.0

Experiment Name:	Johannes_Notho kidney
Specimen Name:	20230111_female
Tube Name:	kidney adult 5

Population	#Events	%Parent	%Total	GFP-A Mean	GFP-A %CV
GFP+	7	0.1646	0.0450	25,326	73.4
living cells	4,252	100.0000	27.3476	1,070	411.2

BD FACSDiva 8.0

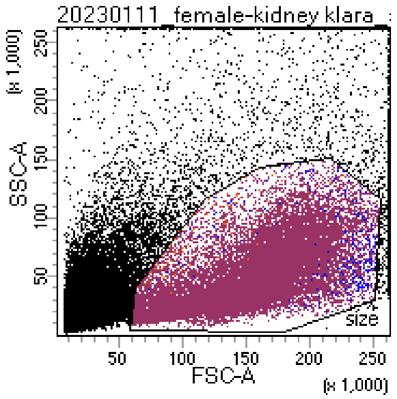

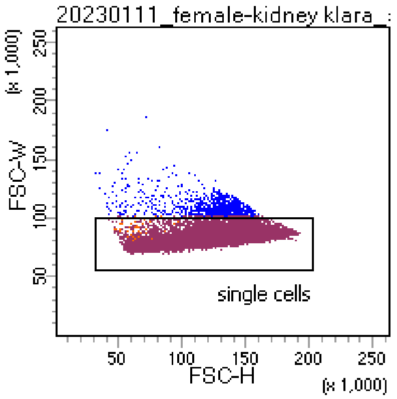

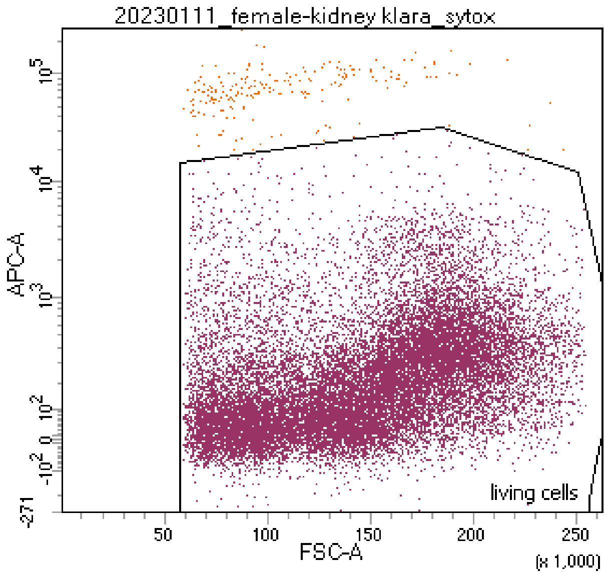

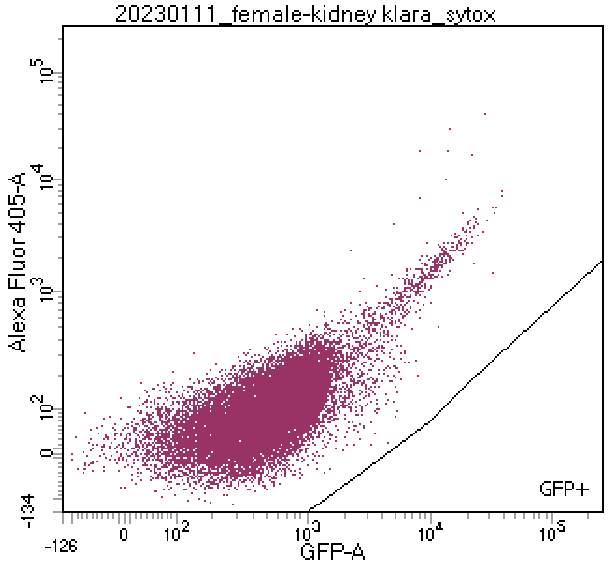

Tube: kidney klara_sytox

Population	#Events	%Parent	%Total
All Events	70,503	####	100.0
size	23,023	32.7	32.7
single cells	22,117	96.1	31.4
living cells	21,910	99.1	31.1
GFP+	0	0.0	0.0

Experiment Name: Johannes_Notho kidney
Specimen Name: 20230111_female
Tube Name: kidney klara_sytox

Population	#Events	%Parent	%Total	GFP-A Mean	GFP-A %CV
GFP+	0	0.0000	0.0000	####	####
living cells	21,910	99.0641	31.0767	823	198.7

BD FACSDiva 8.0

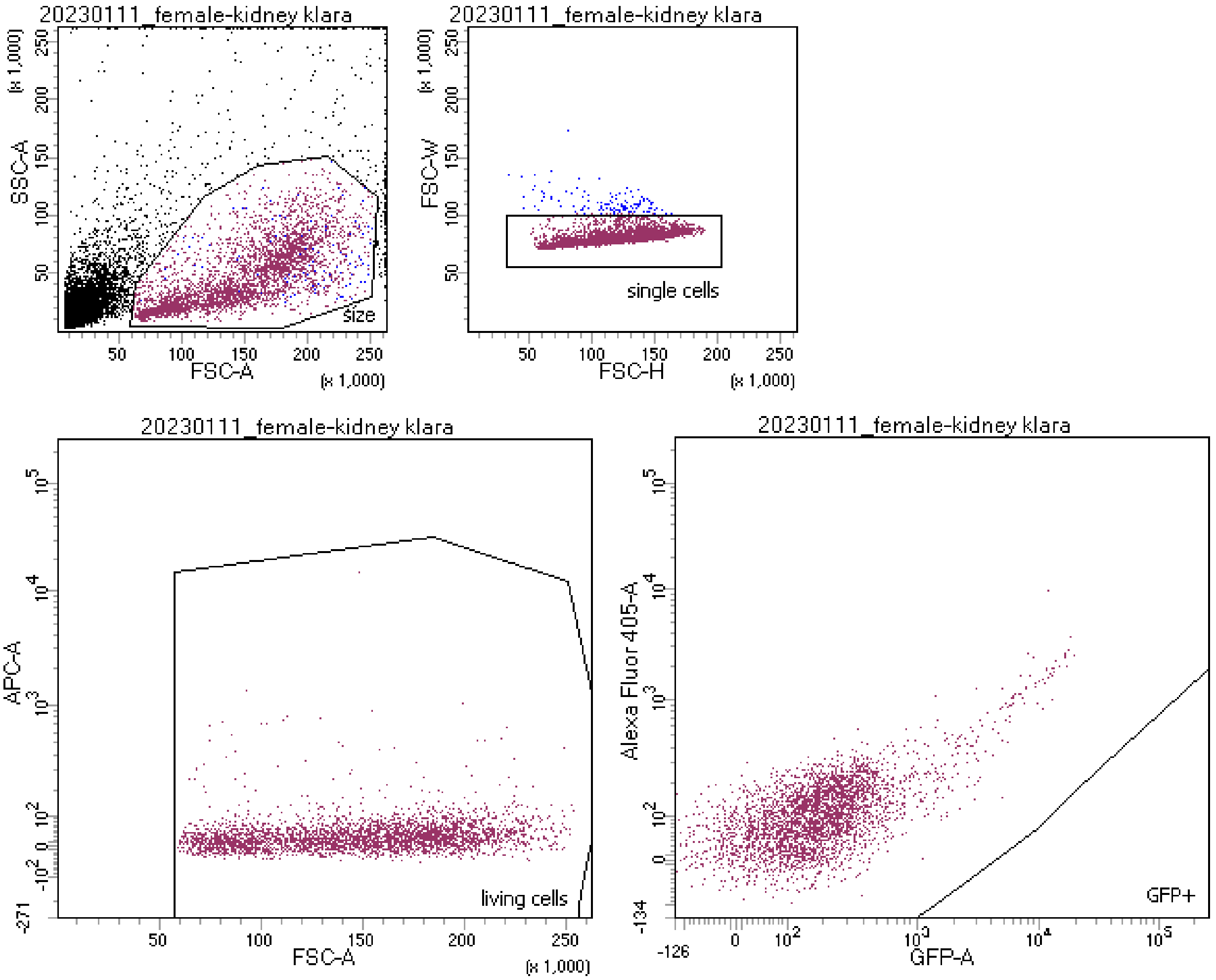

Tube: kidney klara

Population	#Events	%Parent	%Total
All Events	10,000	####	100.0
size	3,309	33.1	33.1
single cells	3,179	96.1	31.8
living cells	3,179	100.0	31.8
GFP+	0	0.0	0.0

Experiment Name: Johannes_Notho kidney
Specimen Name: 20230111_female
Tube Name: kidney klara

Population	#Events	%Parent	%Total	GFP-A Mean	GFP-A %CV
GFP+	0	0.0000	0.0000	####	####
living cells	3,179	100.0000	31.7900	426	333.8

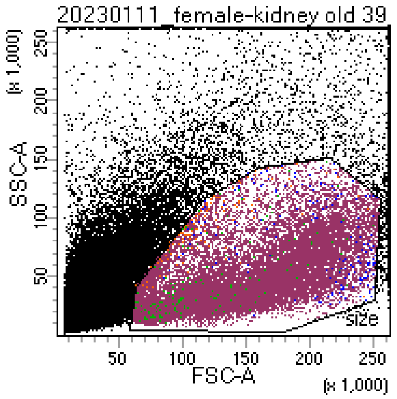

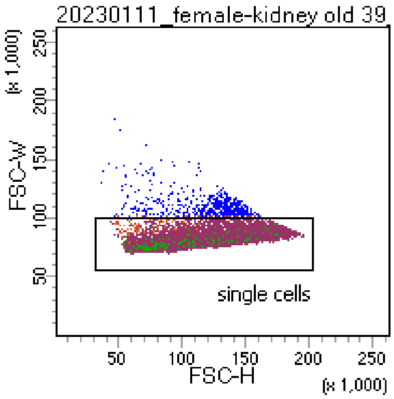

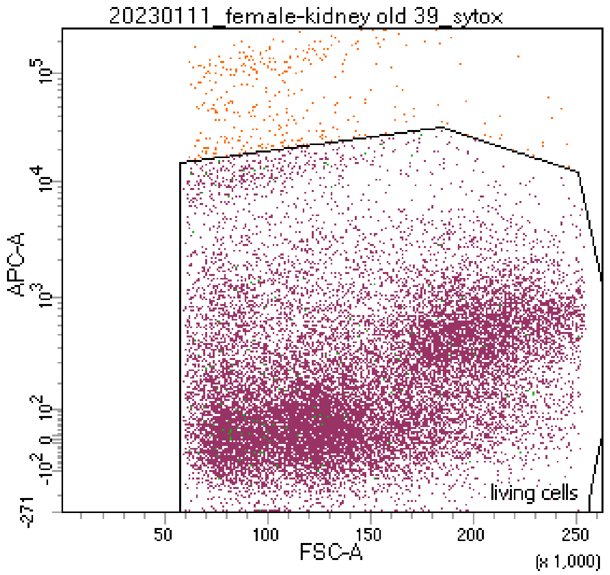

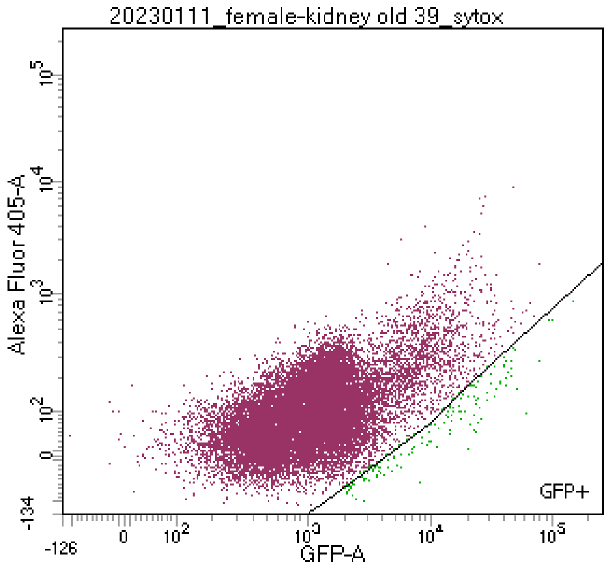

Tube: kidney old 39_sytox

Population	#Events	%Parent	%Total
All Events	112,169	####	100.0
size	20,751	18.5	18.5
single cells	20,195	97.3	18.0
living cells	19,839	98.2	17.7
GFP+	146	0.7	0.1

Experiment Name: Johannes_Notho kidney
Specimen Name: 20230111_female
Tube Name: kidney old 39_sytox

Population	#Events	%Parent	%Total	GFP-A Mean	GFP-A %CV
GFP+	146	0.7359	0.1302	18,024	116.1
living cells	19,839	98.2372	17.6867	1,981	201.9

BD FACSDiva 8.0

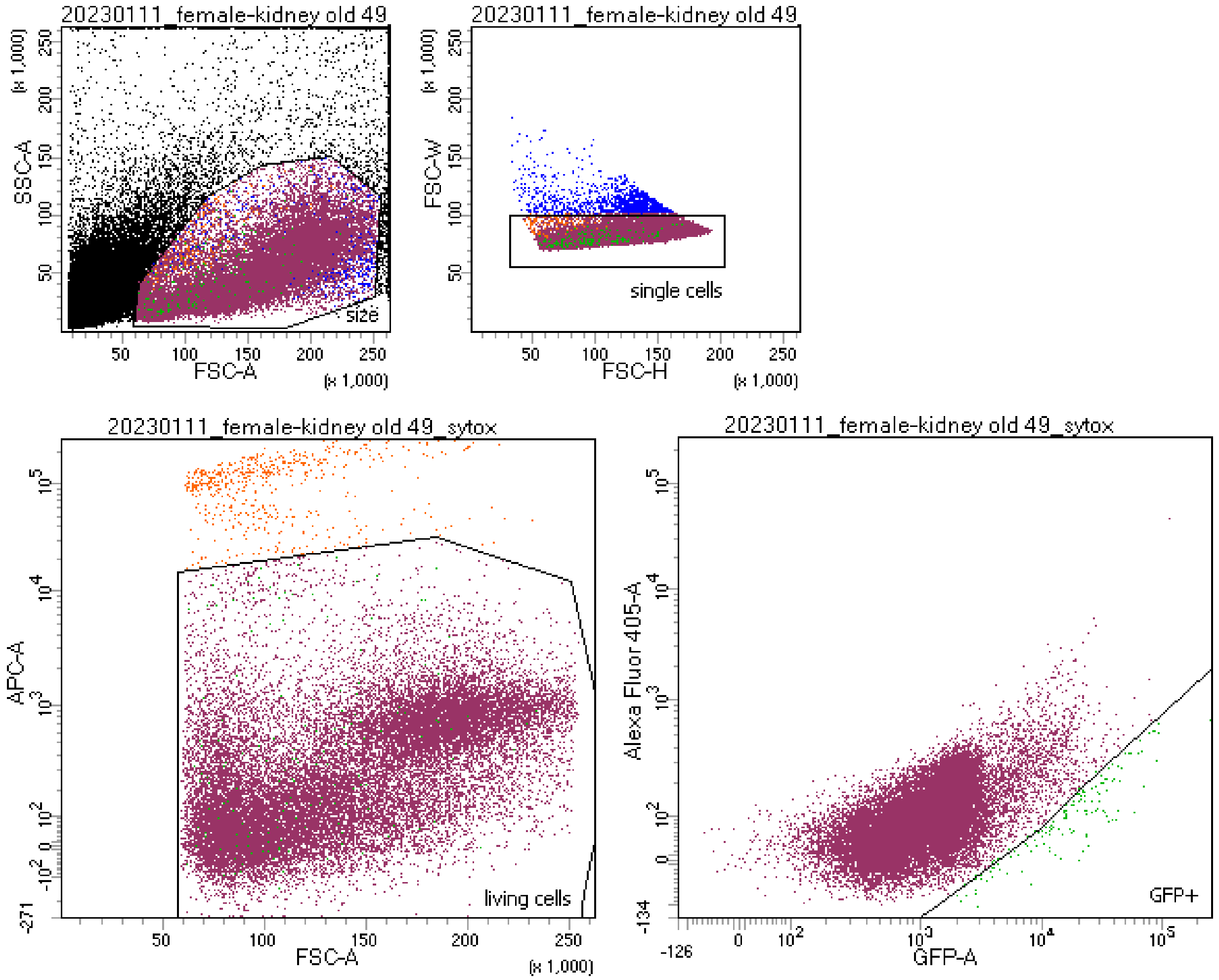

Tube: kidney old 49_sytox

Population	#Events	%Parent	%Total
All Events	95,664	####	100.0
size	26,950	28.2	28.2
single cells	25,772	95.6	26.9
living cells	25,226	97.9	26.4
GFP+	149	0.6	0.2

Experiment Name: Johannes_Notho kidney
Specimen Name: 20230111_female
Tube Name: kidney old 49_sytox

Population	#Events	%Parent	%Total	GFP-A Mean	GFP-A %CV
GFP+	149	0.5907	0.1558	23,827	117.8
living cells	25,226	97.8814	26.3694	1,826	213.8

BD FACSDiva 8.0

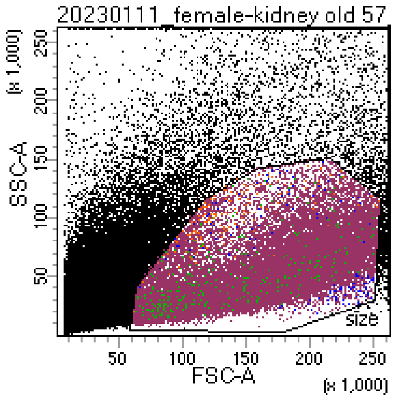

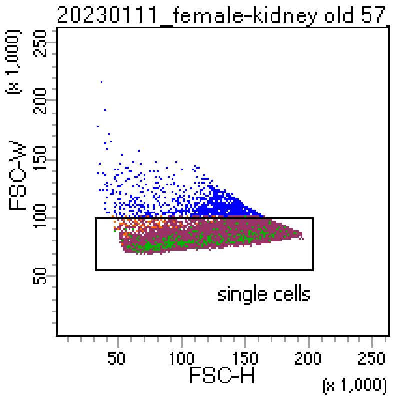

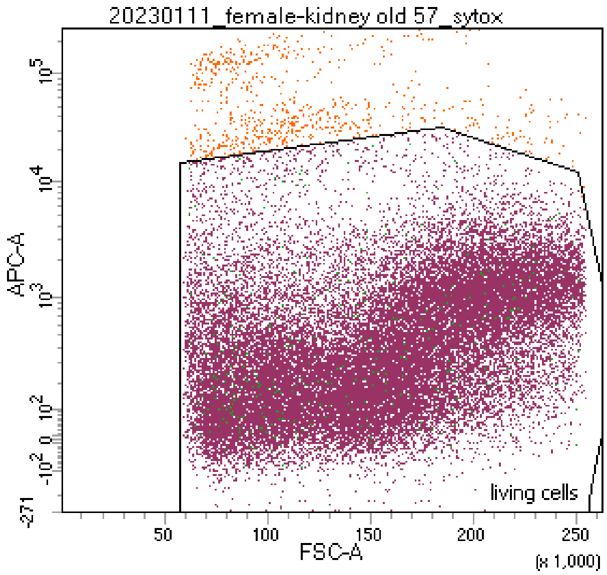

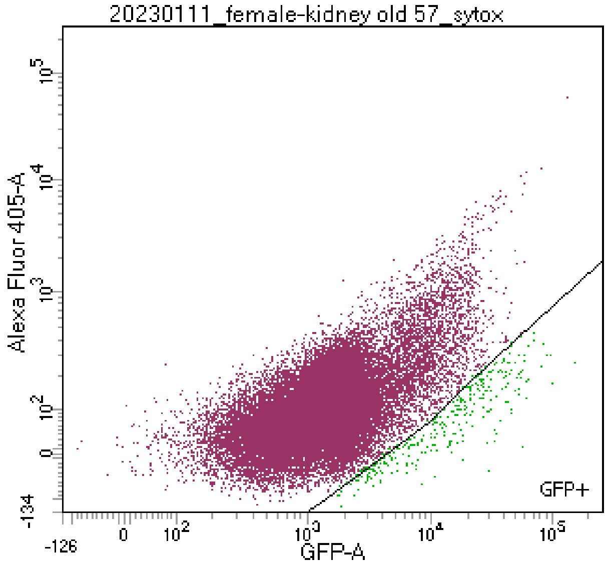

Tube: kidney old 57_sytox

Population	#Events	%Parent	%Total
All Events	156,800	####	100.0
size	36,916	23.5	23.5
single cells	35,841	97.1	22.9
living cells	35,133	98.0	22.4
GFP+	315	0.9	0.2

Experiment Name: Johannes_Notho kidney
Specimen Name: 20230111_female
Tube Name: kidney old 57_sytox

Population	#Events	%Parent	%Total	GFP-A Mean	GFP-A %CV
GFP+	315	0.8966	0.2009	20,173	98.9
living cells	35,133	98.0246	22.4063	2,341	178.0

BD FACSDiva 8.0

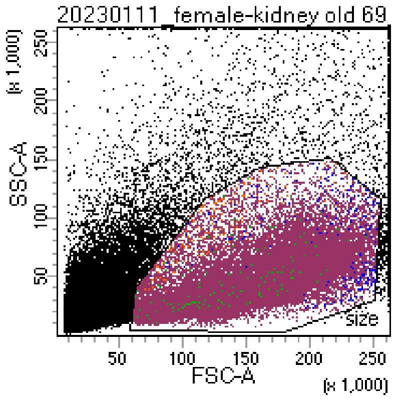

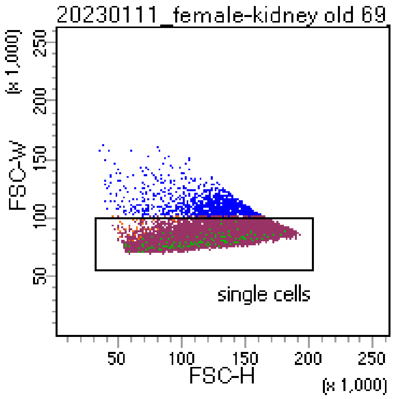

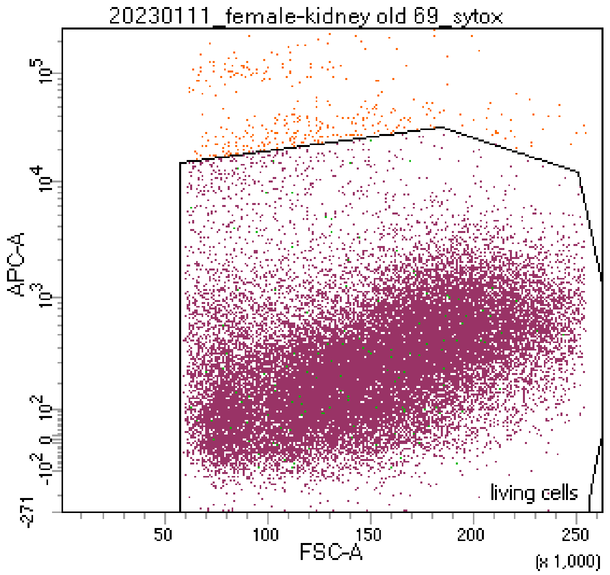

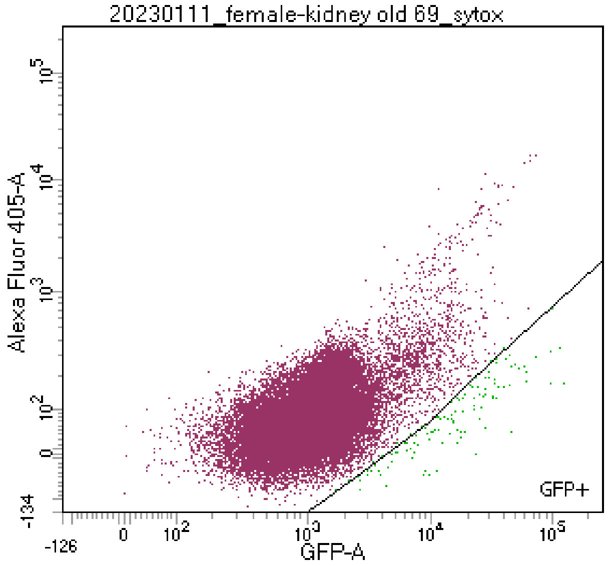

Tube: kidney old 69_sytox

Population	#Events	%Parent	%Total
All Events	86,908	####	100.0
size	34,423	39.6	39.6
single cells	33,239	96.6	38.2
living cells	32,882	98.9	37.8
GFP+	128	0.4	0.1

Experiment Name: Johannes_Notho kidney
Specimen Name: 20230111_female
Tube Name: kidney old 69_sytox

Population	#Events	%Parent	%Total	GFP-A Mean	GFP-A %CV
GFP+	128	0.3893	0.1473	21,833	107.6
living cells	32,882	98.9260	37.8354	1,603	184.4

BD FACSDiva 8.0

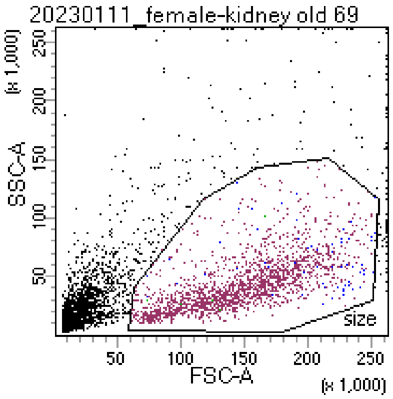

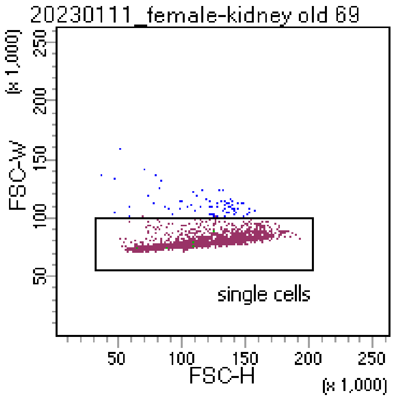

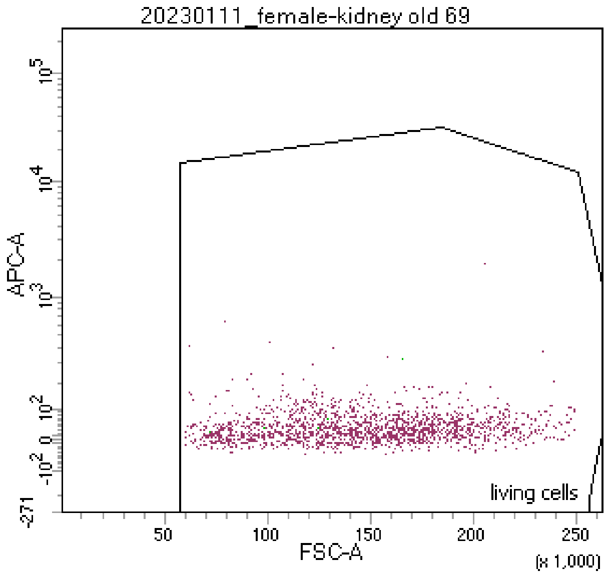

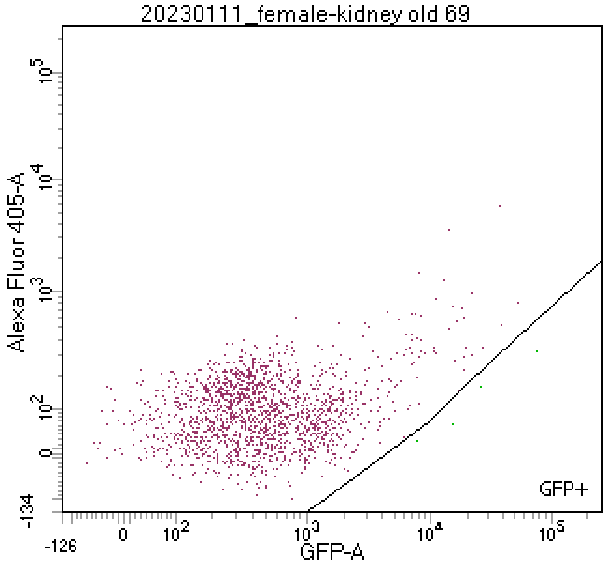

Tube: kidney old 69

Population	#Events	%Parent	%Total
All Events	4,108	####	100.0
size	1,731	42.1	42.1
single cells	1,659	95.8	40.4
living cells	1,659	100.0	40.4
GFP+	5	0.3	0.1

Experiment Name: Johannes_Notho kidney
Specimen Name: 20230111_female
Tube Name: kidney old 69

Population	#Events	%Parent	%Total	GFP-A Mean	GFP-A %CV
GFP+	5	0.3014	0.1217	26,142	116.1
living cells	1,659	100.0000	40.3846	1,062	320.2

BD FACSDiva 8.0

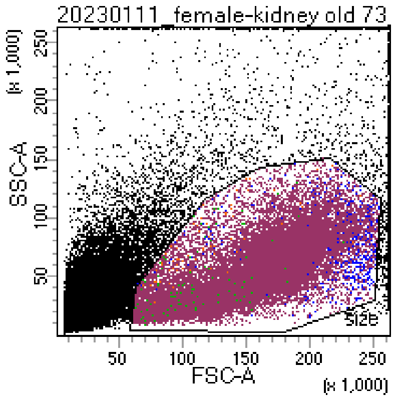

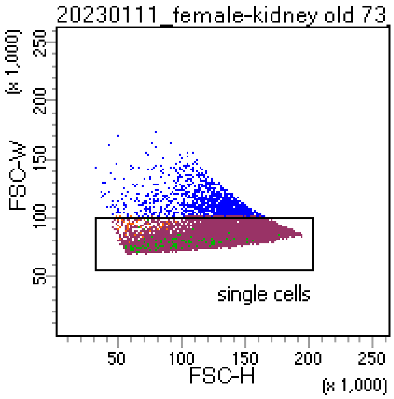

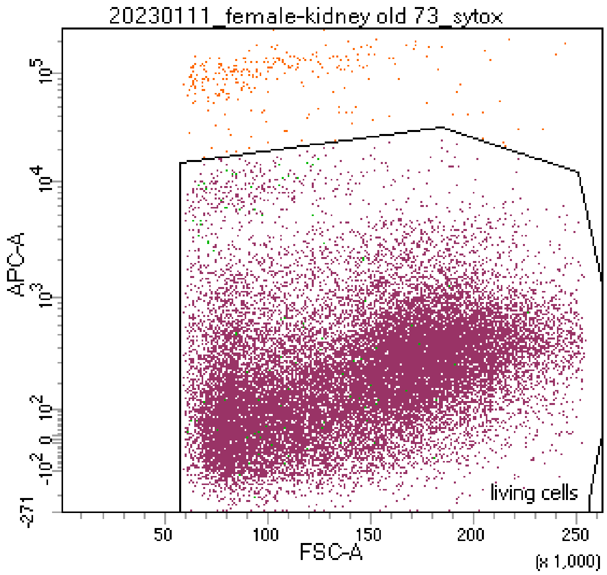

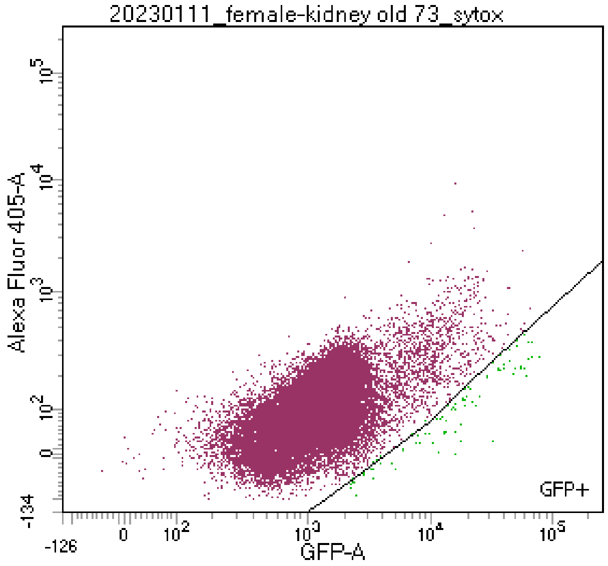

Tube: kidney old 73_sytox

Population	#Events	%Parent	%Total
All Events	69,285	####	100.0
size	28,433	41.0	41.0
single cells	27,175	95.6	39.2
living cells	26,914	99.0	38.8
GFP+	92	0.3	0.1

Experiment Name: Johannes_Notho kidney
Specimen Name: 20230111_female
Tube Name: kidney old 73_sytox

Population	#Events	%Parent	%Total	GFP-A Mean	GFP-A %CV
GFP+	92	0.3418	0.1328	22,272	91.7
living cells	26,914	99.0396	38.8453	1,751	169.7

BD FACSDiva 8.0

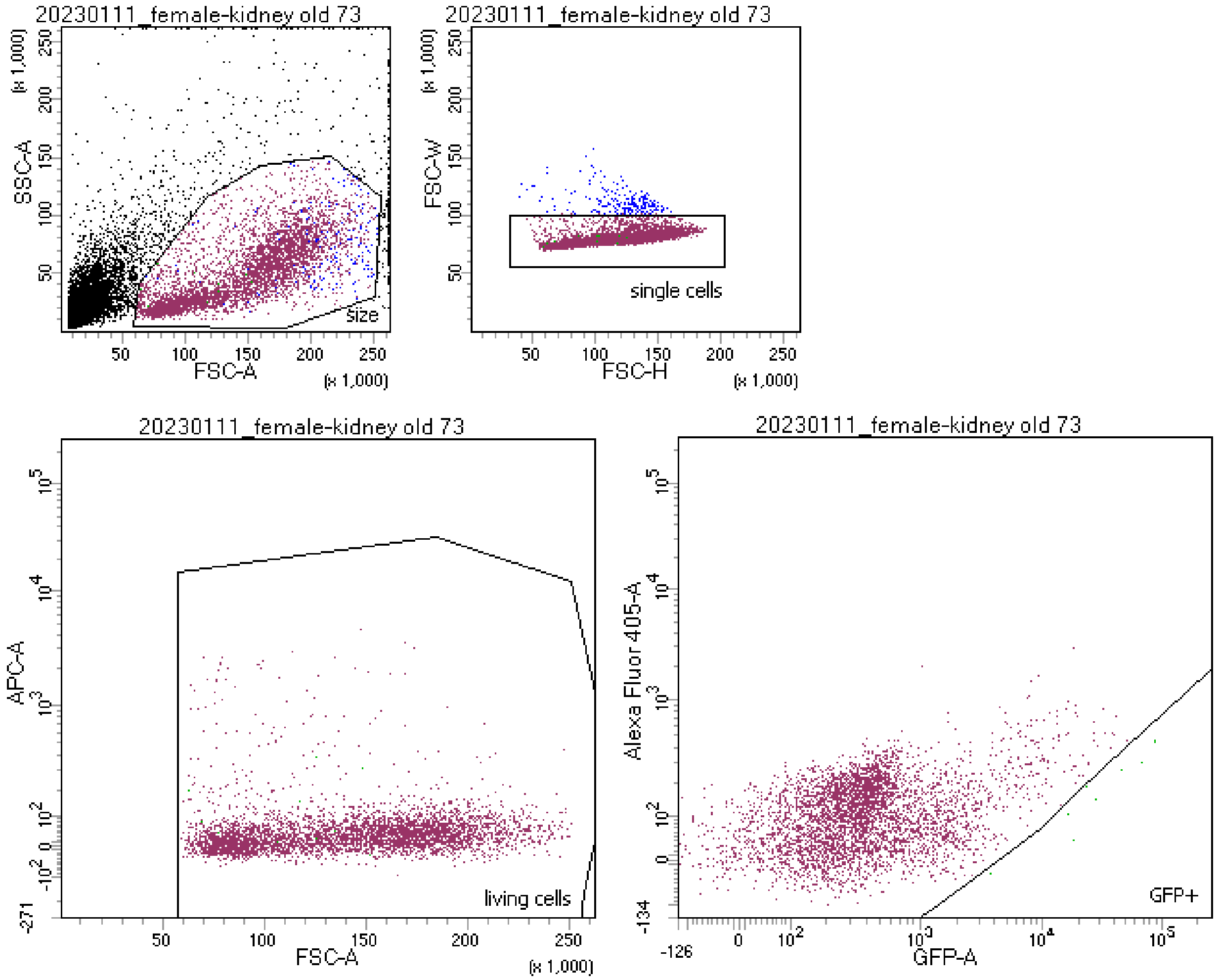

Tube: kidney old 73

Population	#Events	%Parent	%Total
All Events	11,336	####	100.0
size	4,471	39.4	39.4
single cells	4,257	95.2	37.6
living cells	4,257	100.0	37.6
GFP+	10	0.2	0.1

Experiment Name: Johannes_Notho kidney
Specimen Name: 20230111_female
Tube Name: kidney old 73

Population	#Events	%Parent	%Total	GFP-A Mean	GFP-A %CV
GFP+	10	0.2349	0.0882	40,692	75.8
living cells	4,257	100.0000	37.5529	987	356.0

BD FACSDiva 8.0

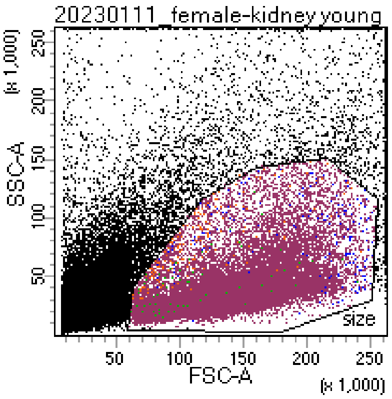

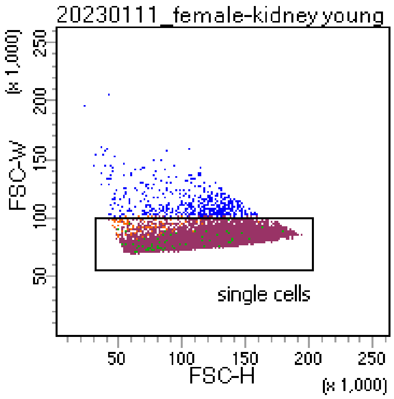

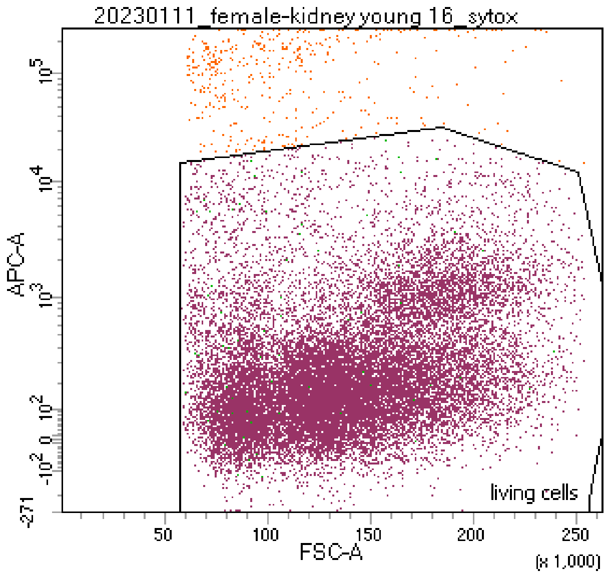

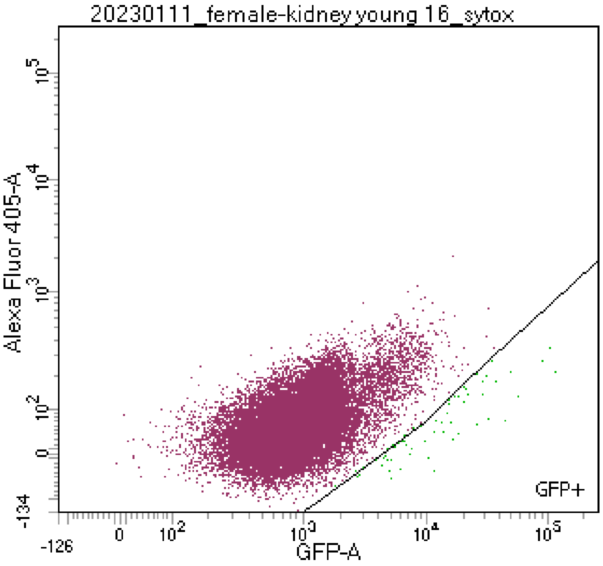

Tube: kidney young 16_sytox

Population	#Events	%Parent	%Total
All Events	69,255	####	100.0
size	24,195	34.9	34.9
single cells	23,702	98.0	34.2
living cells	23,276	98.2	33.6
GFP+	63	0.3	0.1

Experiment Name: Johannes_Notho kidney
Specimen Name: 20230111_female
Tube Name: kidney young 16_sytox

Population	#Events	%Parent	%Total	GFP-A Mean	GFP-A %CV
GFP+	63	0.2707	0.0910	19,256	121.5
living cells	23,276	98.2027	33.6091	1,258	162.3

BD FACSDiva 8.0

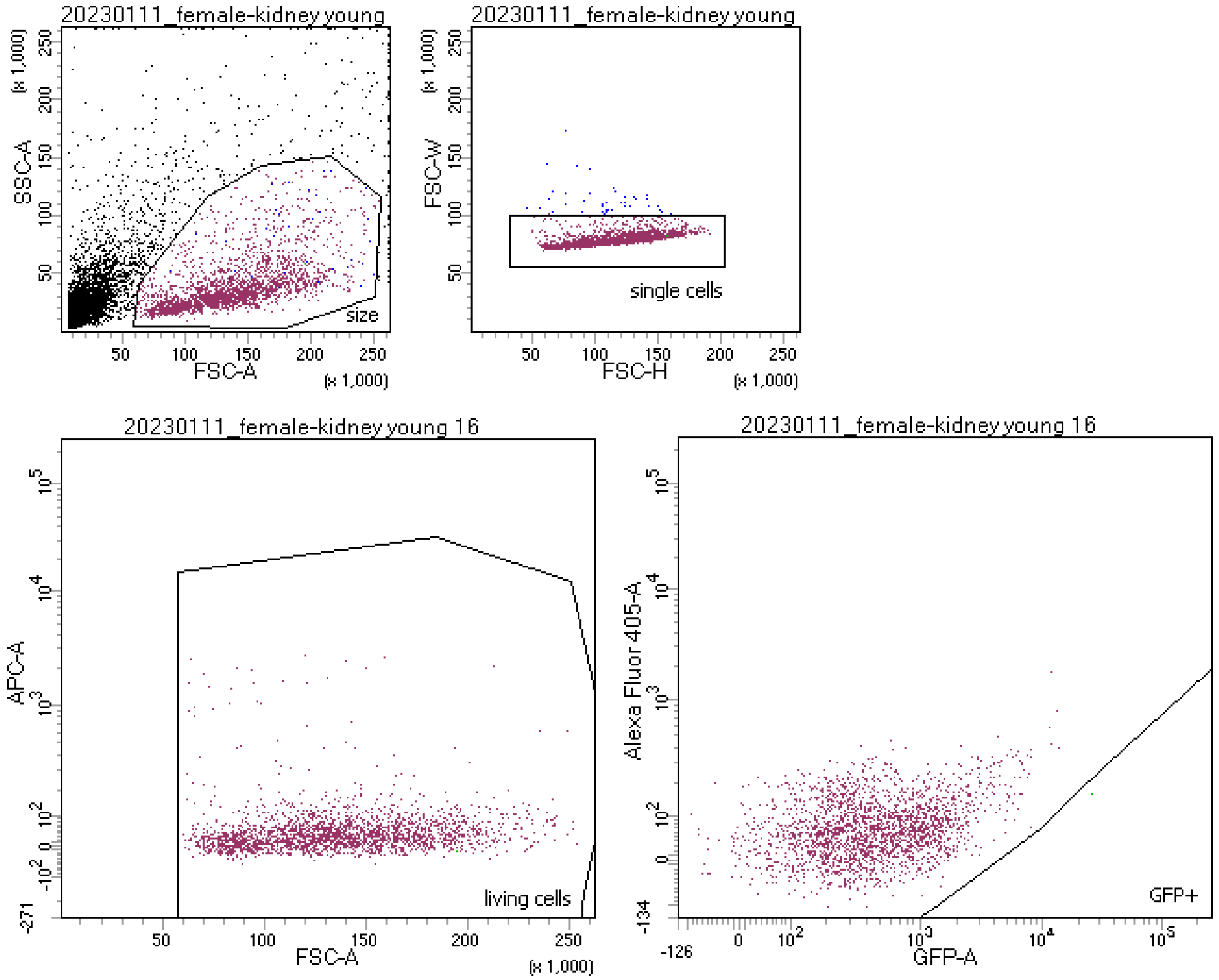

Tube: kidney young 16

Population	#Events	%Parent	%Total
All Events	6,508	####	100.0
size	2,272	34.9	34.9
single cells	2,233	98.3	34.3
living cells	2,233	100.0	34.3
GFP+	1	0.0	0.0

Experiment Name: Johannes_Notho kidney
Specimen Name: 20230111_female
Tube Name: kidney young 16

Population	#Events	%Parent	%Total	GFP-A Mean	GFP-A %CV
GFP+	1	0.0448	0.0154	26,114	####
living cells	2,233	100.0000	34.3116	765	160.2

BD FACSDiva 8.0

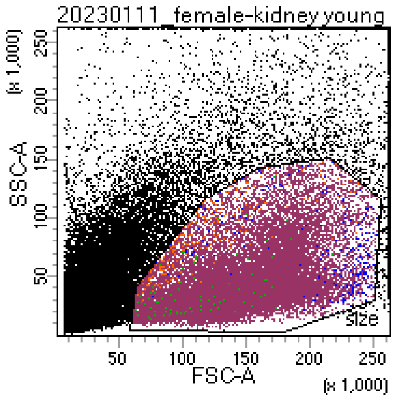

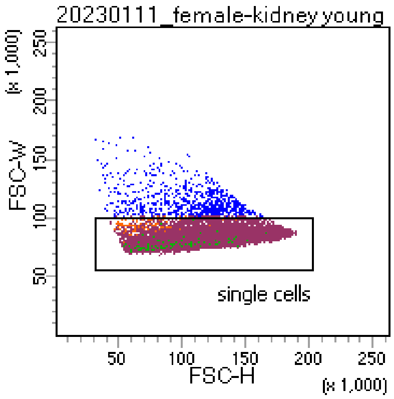

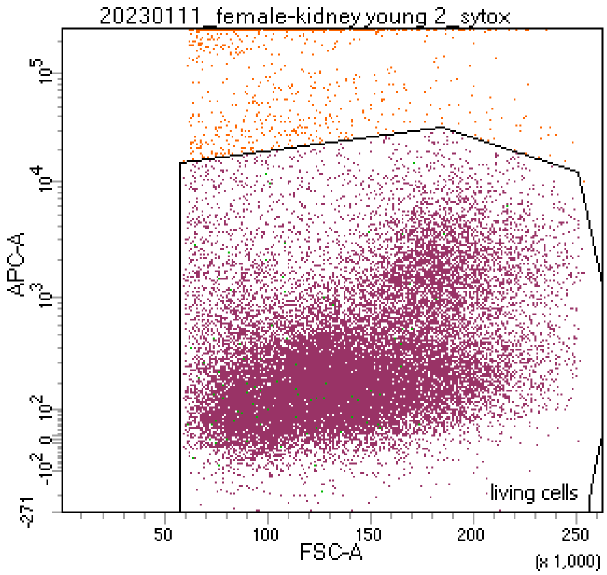

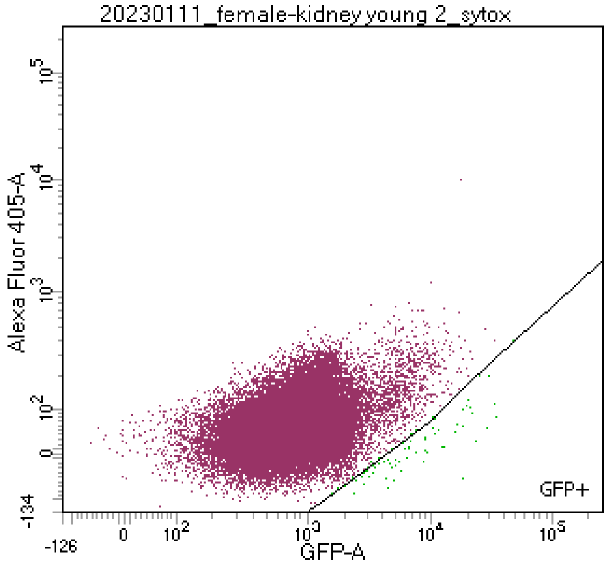

Tube: kidney young 2_sytox

Population	#Events	%Parent	%Total
All Events	107,451	####	100.0
size	30,202	28.1	28.1
single cells	29,355	97.2	27.3
living cells	28,516	97.1	26.5
GFP+	81	0.3	0.1

Experiment Name: Johannes_Notho kidney
Specimen Name: 20230111_female
Tube Name: kidney young 2_sytox

Population	#Events	%Parent	%Total	GFP-A Mean	GFP-A %CV
GFP+	81	0.2841	0.0754	9,139	98.3
living cells	28,516	97.1419	26.5386	1,078	127.4

BD FACSDiva 8.0

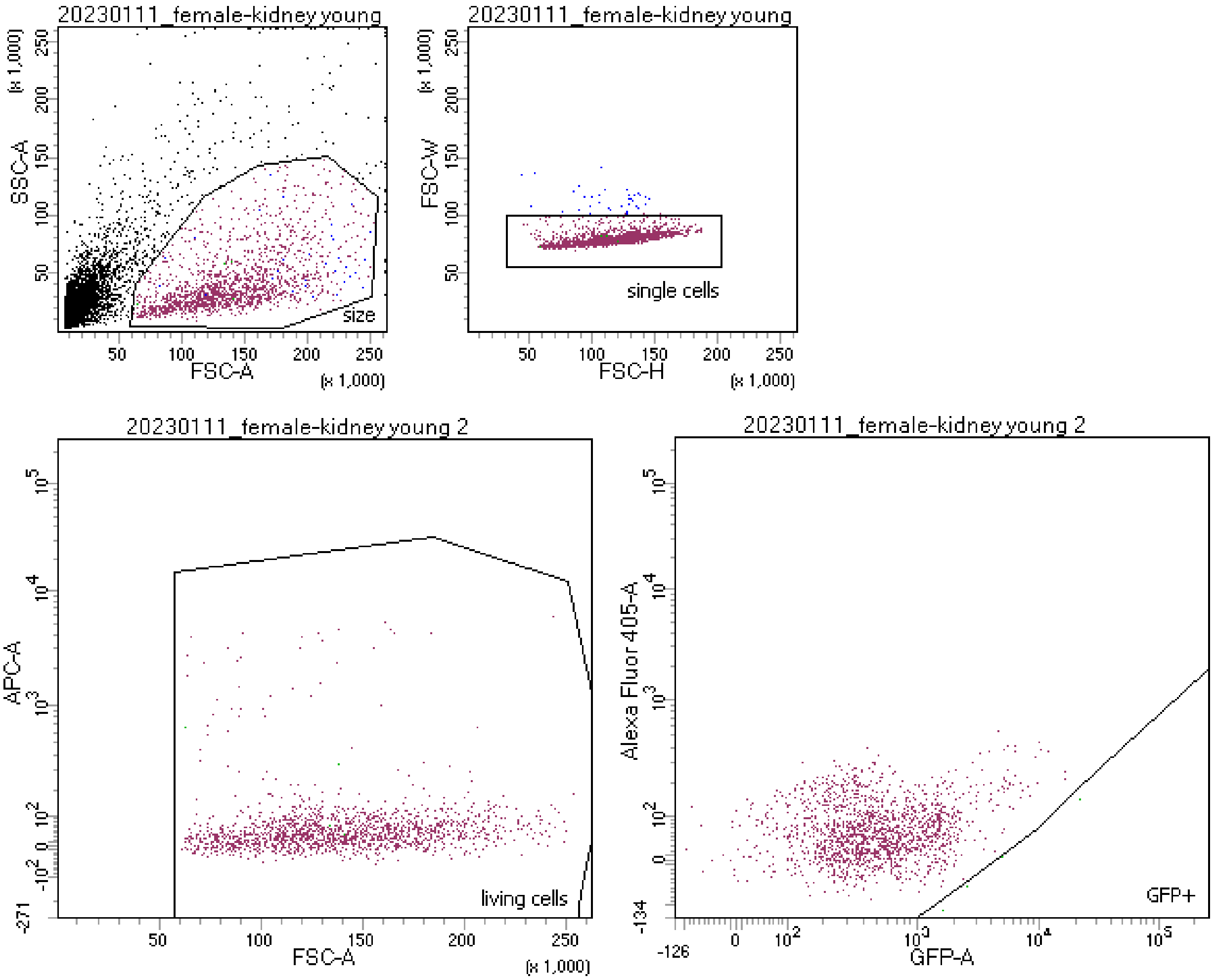

Tube: kidney young 2

Population	#Events	%Parent	%Total
All Events	5,158	####	100.0
size	1,554	30.1	30.1
single cells	1,518	97.7	29.4
living cells	1,518	100.0	29.4
GFP+	4	0.3	0.1

Experiment Name: Johannes_Notho kidney
Specimen Name: 20230111_female
Tube Name: kidney young 2

Population	#Events	%Parent	%Total	GFP-A Mean	GFP-A %CV
GFP+	4	0.2635	0.0775	7,709	124.2
living cells	1,518	100.0000	29.4300	819	163.0

BD FACSDiva 8.0

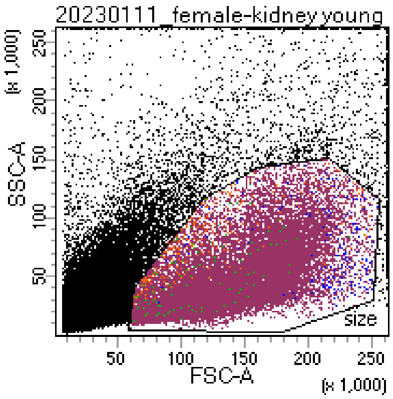

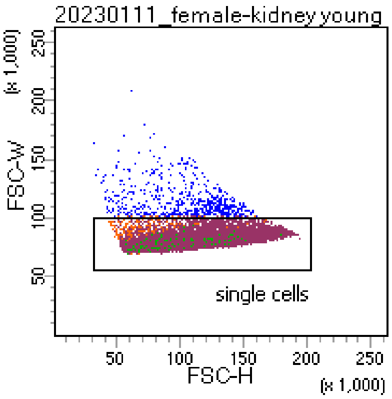

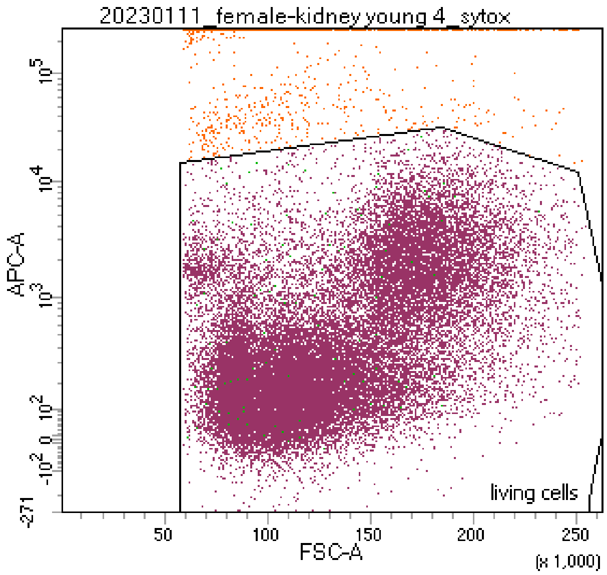

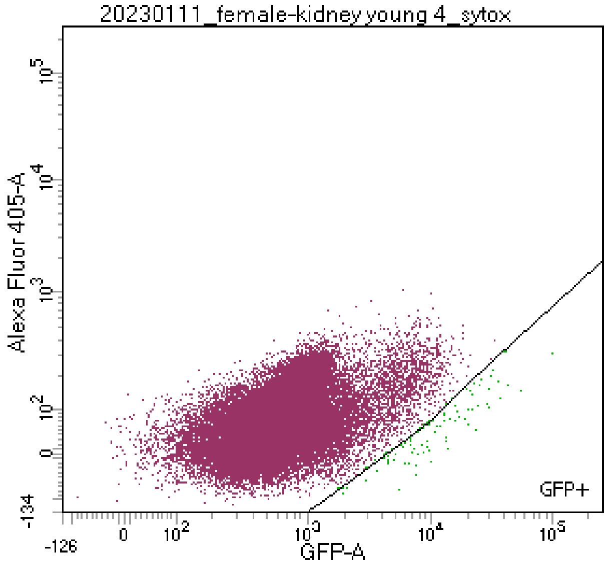

Tube: kidney young 4_sytox

Population	#Events	%Parent	%Total
All Events	101,628	####	100.0
size	36,000	35.4	35.4
single cells	35,400	98.3	34.8
living cells	34,207	96.6	33.7
GFP+	97	0.3	0.1

Experiment Name: Johannes_Notho kidney
Specimen Name: 20230111_female
Tube Name: kidney young 4_sytox

Population	#Events	%Parent	%Total	GFP-A Mean	GFP-A %CV
GFP+	97	0.2836	0.0954	13,818	100.8
living cells	34,207	96.6299	33.6590	985	168.6

BD FACSDiva 8.0

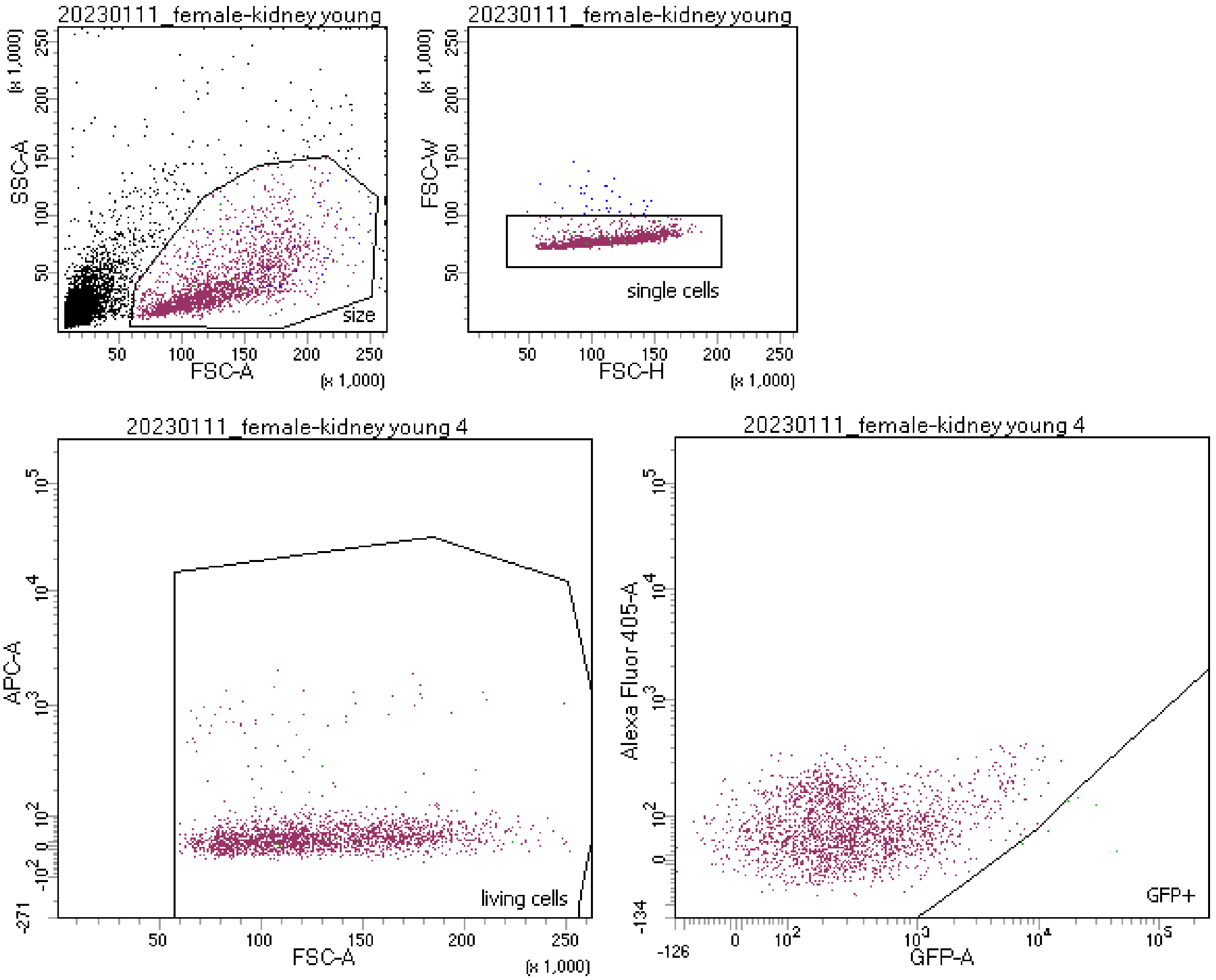

Tube: kidney young 4

Population	#Events	%Parent	%Total
All Events	5,980	####	100.0
size	2,215	37.0	37.0
single cells	2,182	98.5	36.5
living cells	2,182	100.0	36.5
GFP+	7	0.3	0.1

Experiment Name: Johannes_Notho kidney
Specimen Name: 20230111_female
Tube Name: kidney young 4

Population	#Events	%Parent	%Total	GFP-A Mean	GFP-A %CV
GFP+	7	0.3208	0.1171	23,269	55.4
living cells	2,182	100.0000	36.4883	706	268.6

BD FACSDiva 8.0

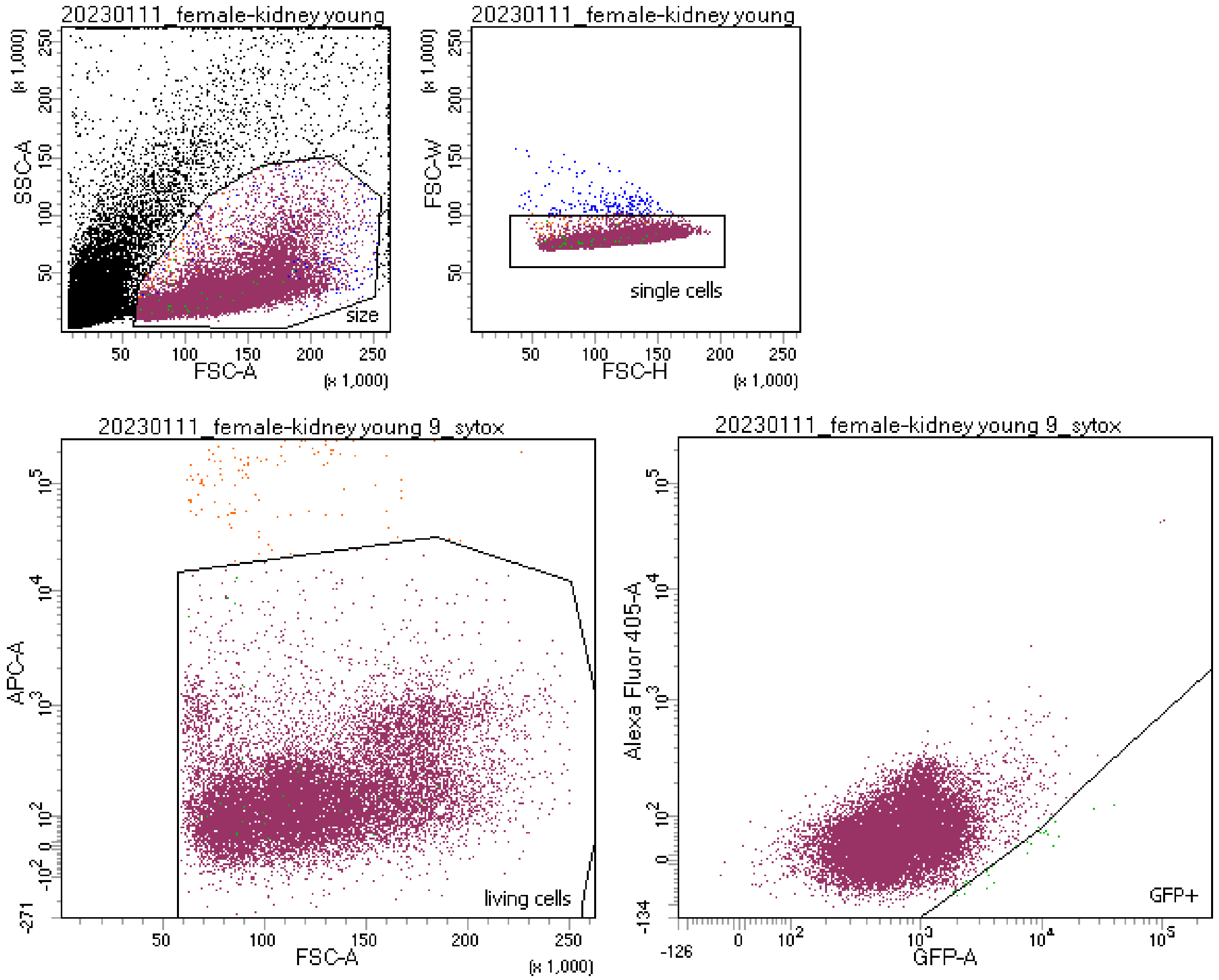

Tube: kidney young 9_sytox

Population	#Events	%Parent	%Total
All Events	36,295	####	100.0
size	19,213	52.9	52.9
single cells	19,013	99.0	52.4
living cells	18,908	99.4	52.1
GFP+	31	0.2	0.1

Experiment Name: Johannes_Notho kidney
Specimen Name: 20230111_female
Tube Name: kidney young 9_sytox

Population	#Events	%Parent	%Total	GFP-A Mean	GFP-A %CV
GFP+	31	0.1640	0.0854	7,759	103.0
living cells	18,908	99.4477	52.0953	907	159.8

BD FACSDiva 8.0

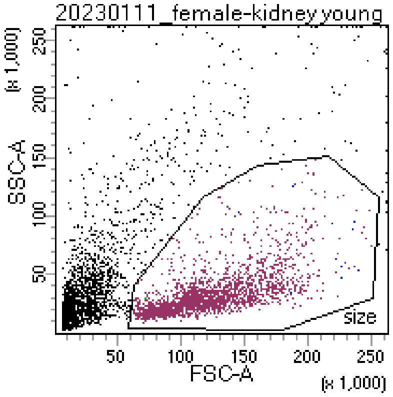

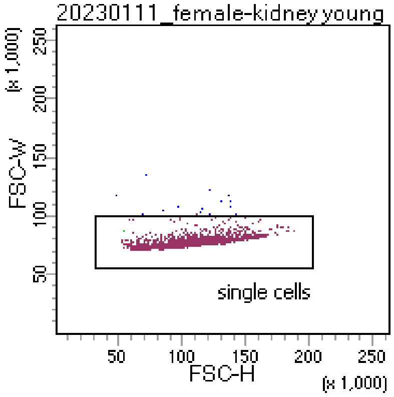

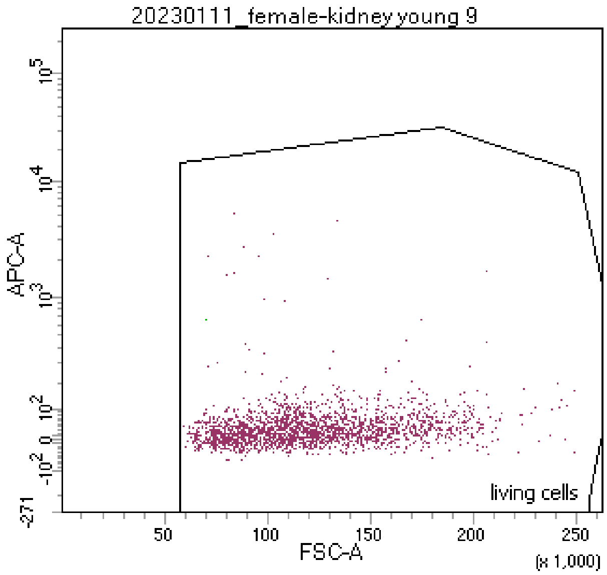

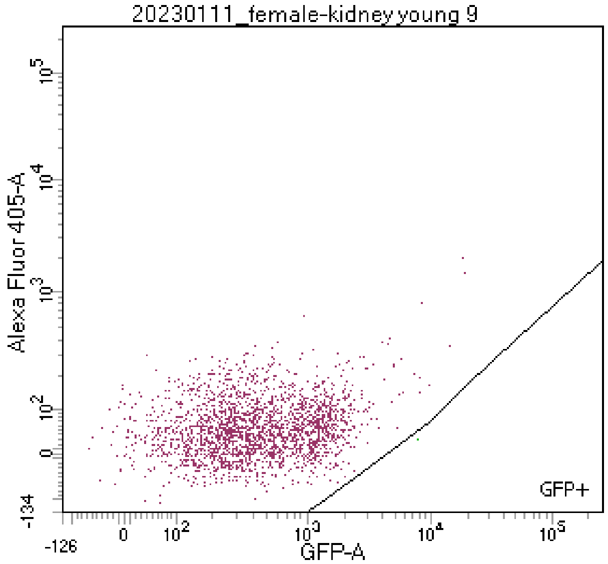

Tube: kidney young 9

Population	#Events	%Parent	%Total
All Events	4,137	####	100.0
size	2,203	53.3	53.3
single cells	2,188	99.3	52.9
living cells	2,188	100.0	52.9
GFP+	1	0.0	0.0

Experiment Name: Johannes_Notho kidney
Specimen Name: 20230111_female
Tube Name: kidney young 9

Population	#Events	%Parent	%Total	GFP-A Mean	GFP-A %CV
GFP+	1	0.0457	0.0242	7,575	####
living cells	2,188	100.0000	52.8886	656	146.8
