# Supplementary material for: Generation of a transparent killifish line through multiplex CRISPR/Cas9mediated gene inactivation
Source: eLife. 2023 Feb 23;12:e81549. doi: 10.7554/eLife.81549 (PMC10010688; doi:10.7554/eLife.81549)
Supplement: Figure 5—source data 1. [file elife-81549-fig5-data1.zip › Figure_5_source_data/Figure_5_panel_I_source_data/FACS_data_analysis/kidney_male-Batch_Analysis_23012023124440.pdf]

BD FACSDiva 8.0

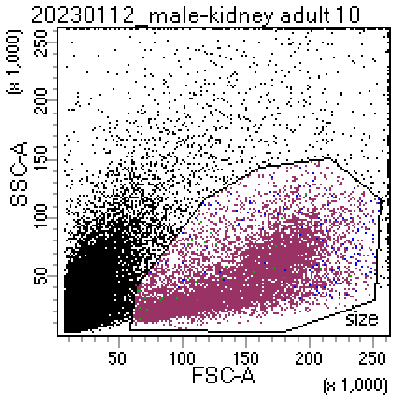

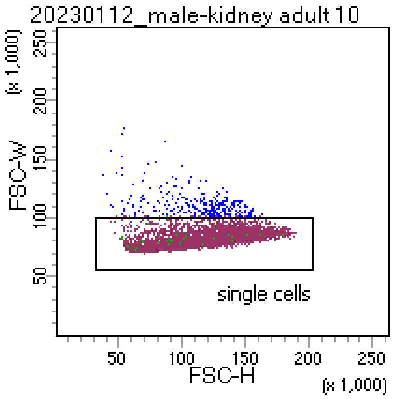

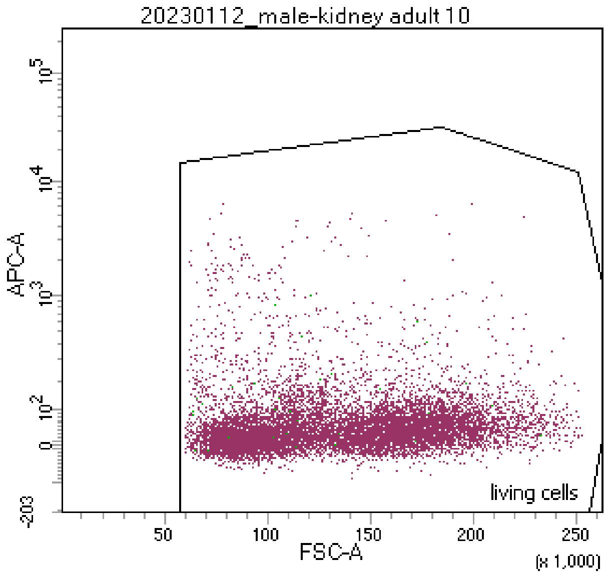

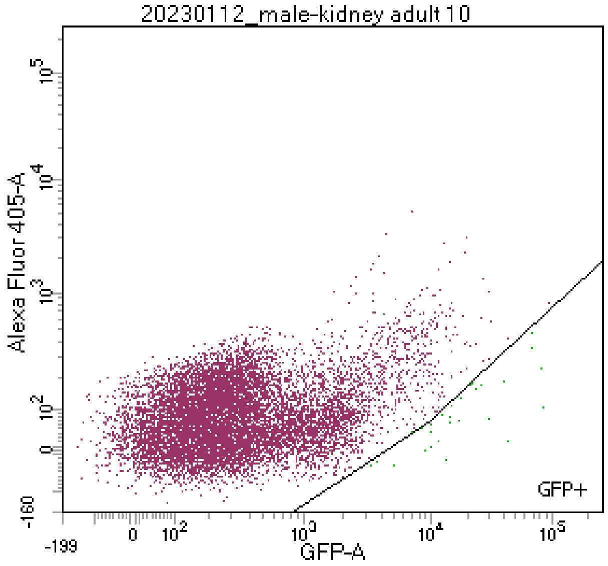

Tube: kidney adult 10

Population	#Events	%Parent	%Total
All Events	39,175	####	100.0
size	11,262	28.7	28.7
single cells	10,975	97.5	28.0
living cells	10,975	100.0	28.0
GFP+	31	0.3	0.1

Experiment Name:	Johannes_Notho kidney
Specimen Name:	20230112_male
Tube Name:	kidney adult 10

Population	#Events	%Parent	%Total	GFP-A Mean	GFP-A %CV
GFP+	31	0.2825	0.0791	23,687	96.4
living cells	10,975	100.0000	28.0153	773	330.4

BD FACSDiva 8.0

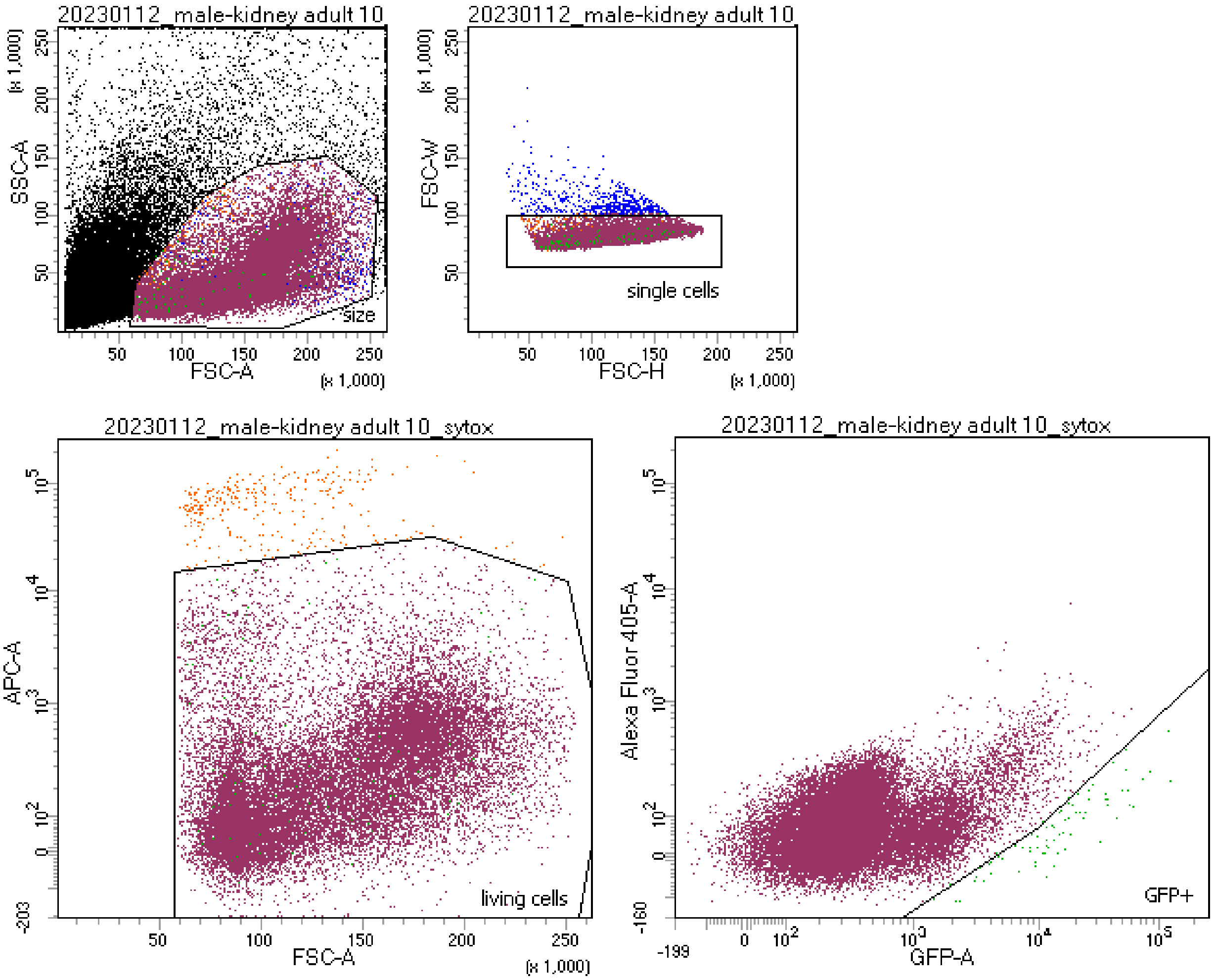

Tube: kidney adult 10_sytox

Population	#Events	%Parent	%Total
All Events	90,548	####	100.0
size	25,602	28.3	28.3
single cells	25,005	97.7	27.6
living cells	24,696	98.8	27.3
GFP+	80	0.3	0.1

Experiment Name: Johannes_Notho kidney
Specimen Name: 20230112_male
Tube Name: kidney adult 10_sytox

Population	#Events	%Parent	%Total	GFP-A Mean	GFP-A %CV
GFP+	80	0.3239	0.0884	21,712	111.8
living cells	24,696	98.7642	27.2739	832	301.7

BD FACSDiva 8.0

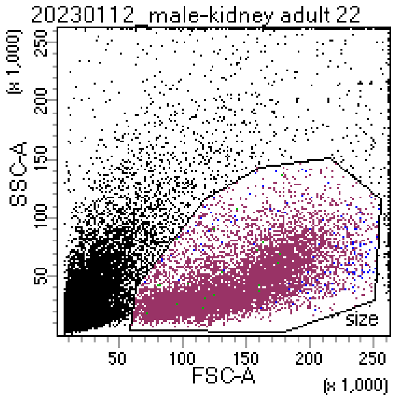

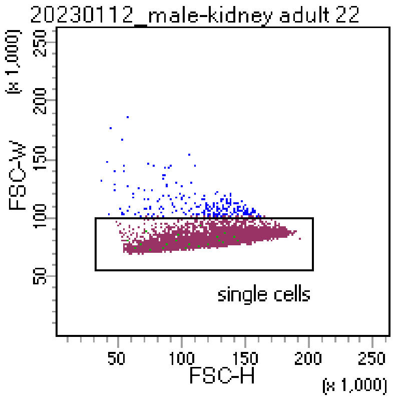

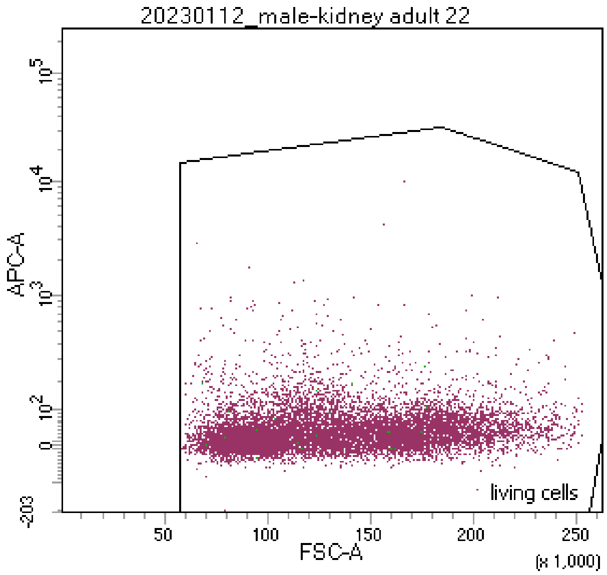

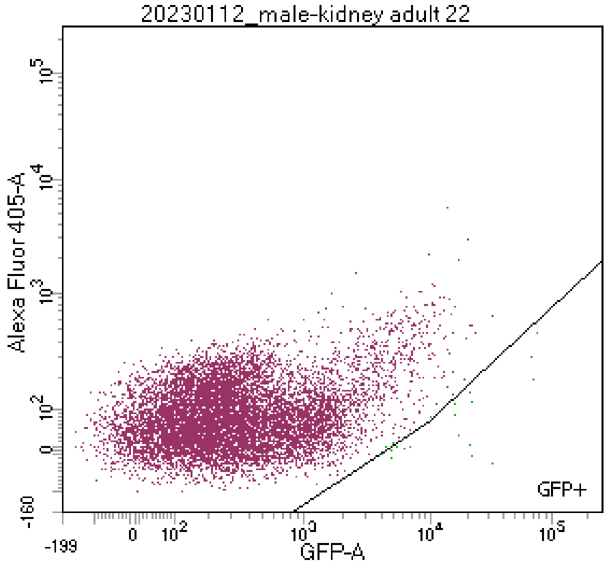

Tube: kidney adult 22

Population	#Events	%Parent	%Total
All Events	28,387	####	100.0
size	10,912	38.4	38.4
single cells	10,687	97.9	37.6
living cells	10,687	100.0	37.6
GFP+	20	0.2	0.1

Experiment Name: Johannes_Notho kidney
Specimen Name: 20230112_male
Tube Name: kidney adult 22

Population	#Events	%Parent	%Total	GFP-A Mean	GFP-A %CV
GFP+	20	0.1871	0.0705	21,700	107.4
living cells	10,687	100.0000	37.6475	664	286.4

BD FACSDiva 8.0

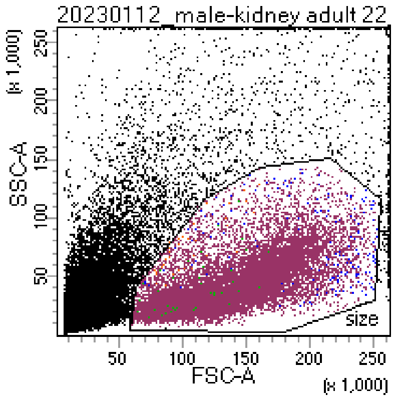

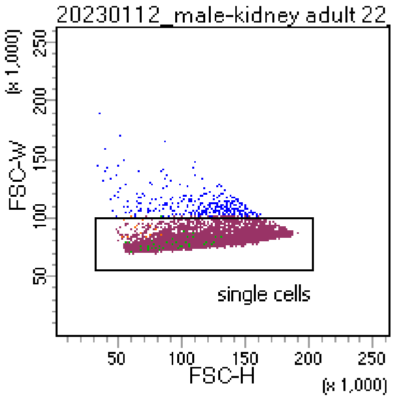

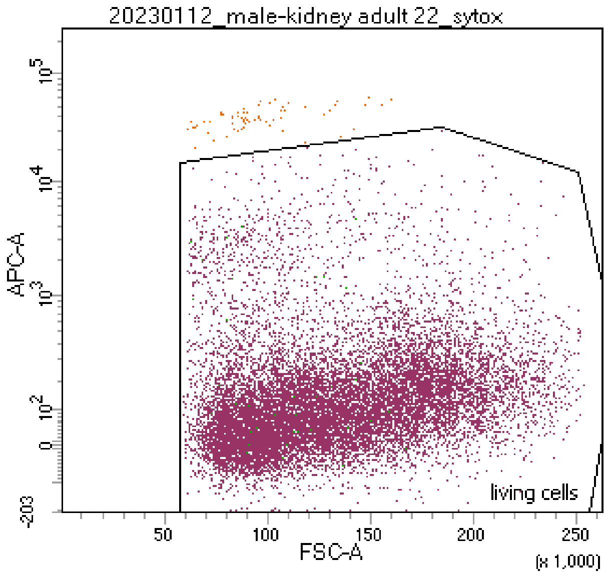

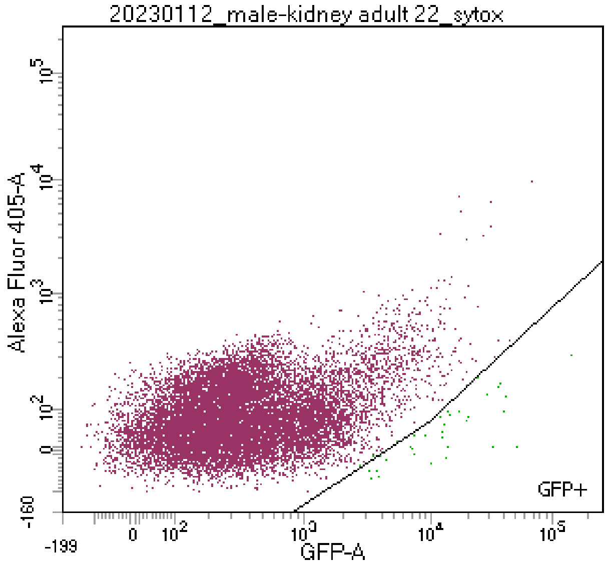

Tube: kidney adult 22_sytox

Population	#Events	%Parent	%Total
All Events	41,222	####	100.0
size	15,792	38.3	38.3
single cells	15,479	98.0	37.6
living cells	15,413	99.6	37.4
GFP+	36	0.2	0.1

Experiment Name: Johannes_Notho kidney
Specimen Name: 20230112_male
Tube Name: kidney adult 22_sytox

Population	#Events	%Parent	%Total	GFP-A Mean	GFP-A %CV
GFP+	36	0.2336	0.0873	19,711	128.9
living cells	15,413	99.5736	37.3902	726	303.3

BD FACSDiva 8.0

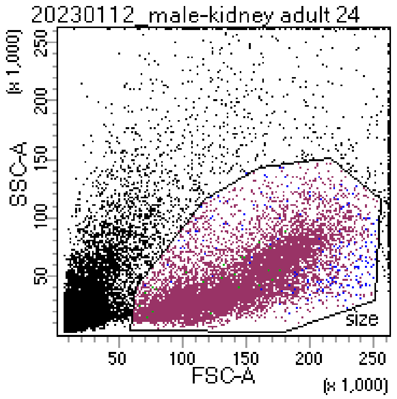

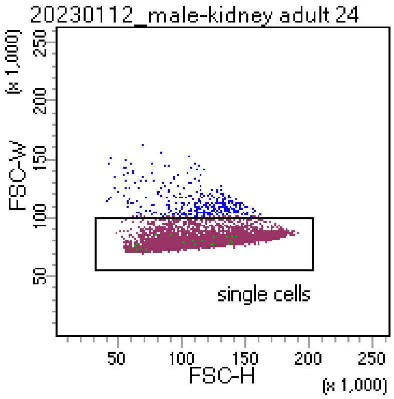

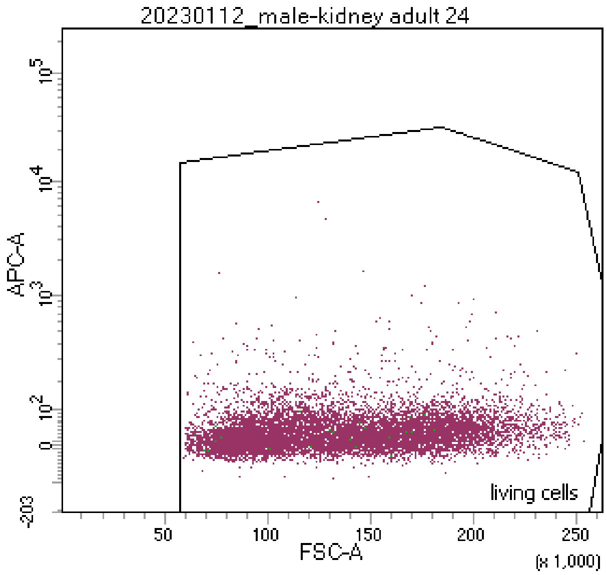

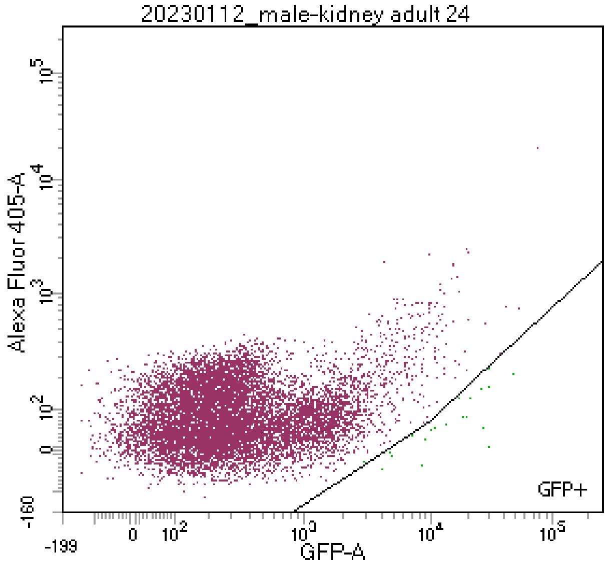

Tube: kidney adult 24

Population	#Events	%Parent	%Total
All Events	21,757	####	100.0
size	10,866	49.9	49.9
single cells	10,543	97.0	48.5
living cells	10,543	100.0	48.5
GFP+	22	0.2	0.1

Experiment Name: Johannes_Notho kidney
Specimen Name: 20230112_male
Tube Name: kidney adult 24

Population	#Events	%Parent	%Total	GFP-A Mean	GFP-A %CV
GFP+	22	0.2087	0.1011	18,532	76.4
living cells	10,543	100.0000	48.4580	661	294.9

BD FACSDiva 8.0

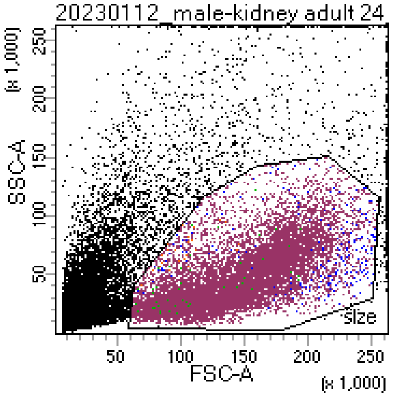

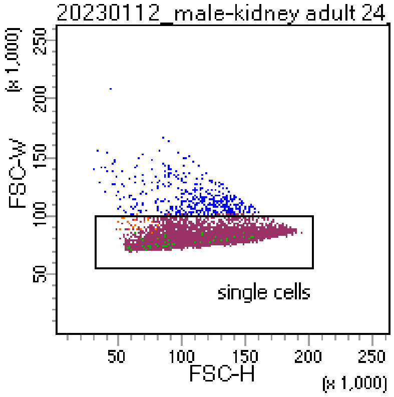

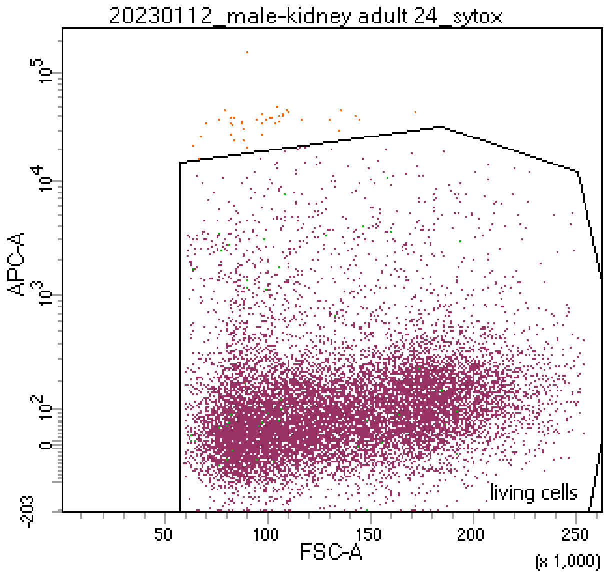

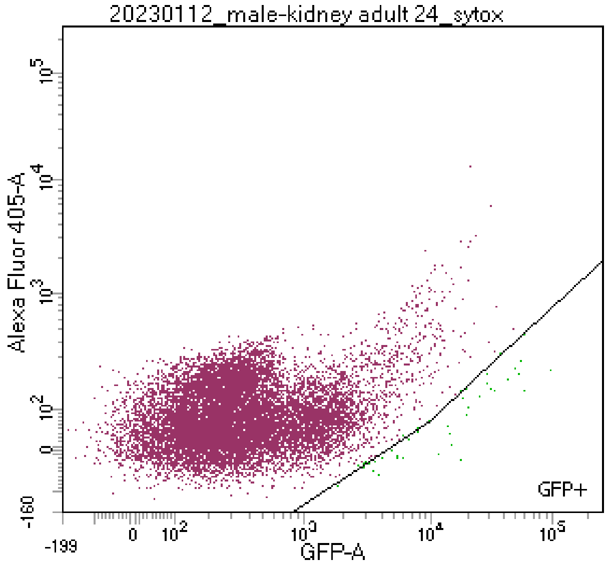

Tube: kidney adult 24_sytox

Population	#Events	%Parent	%Total
All Events	30,495	####	100.0
size	14,960	49.1	49.1
single cells	14,506	97.0	47.6
living cells	14,465	99.7	47.4
GFP+	45	0.3	0.1

Experiment Name: Johannes_Notho kidney
Specimen Name: 20230112_male
Tube Name: kidney adult 24_sytox

Population	#Events	%Parent	%Total	GFP-A Mean	GFP-A %CV
GFP+	45	0.3111	0.1476	19,416	107.8
living cells	14,465	99.7174	47.4340	704	303.4

BD FACSDiva 8.0

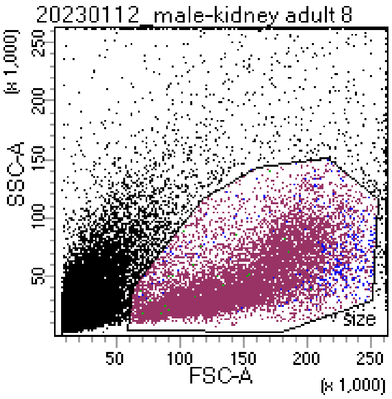

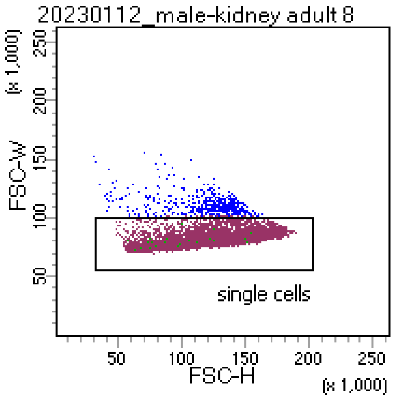

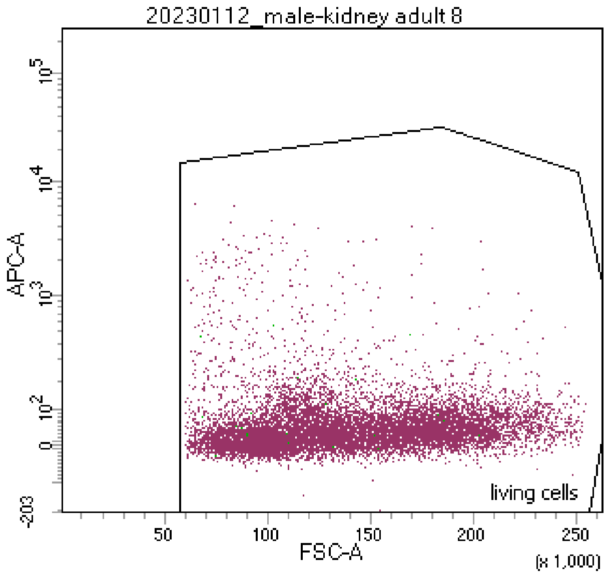

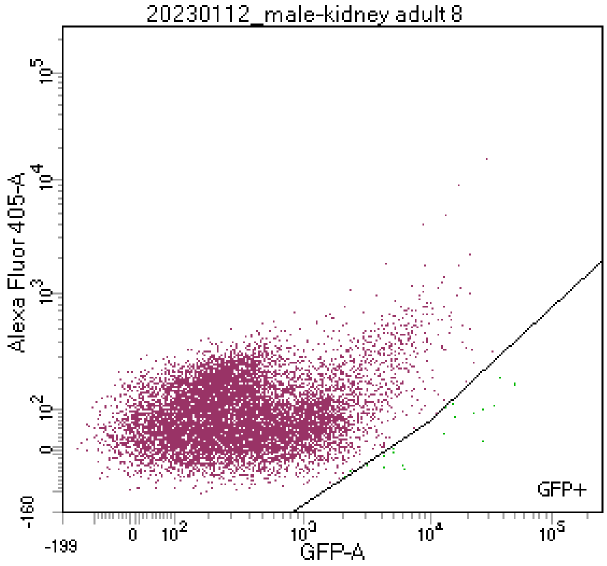

Tube: kidney adult 8

Population	#Events	%Parent	%Total
All Events	37,360	####	100.0
size	11,364	30.4	30.4
single cells	10,843	95.4	29.0
living cells	10,843	100.0	29.0
GFP+	21	0.2	0.1

Experiment Name:	Johannes_Notho kidney
Specimen Name:	20230112_male
Tube Name:	kidney adult 8

Population	#Events	%Parent	%Total	GFP-A Mean	GFP-A %CV
GFP+	21	0.1937	0.0562	16,299	95.1
living cells	10,843	100.0000	29.0230	705	239.0

BD FACSDiva 8.0

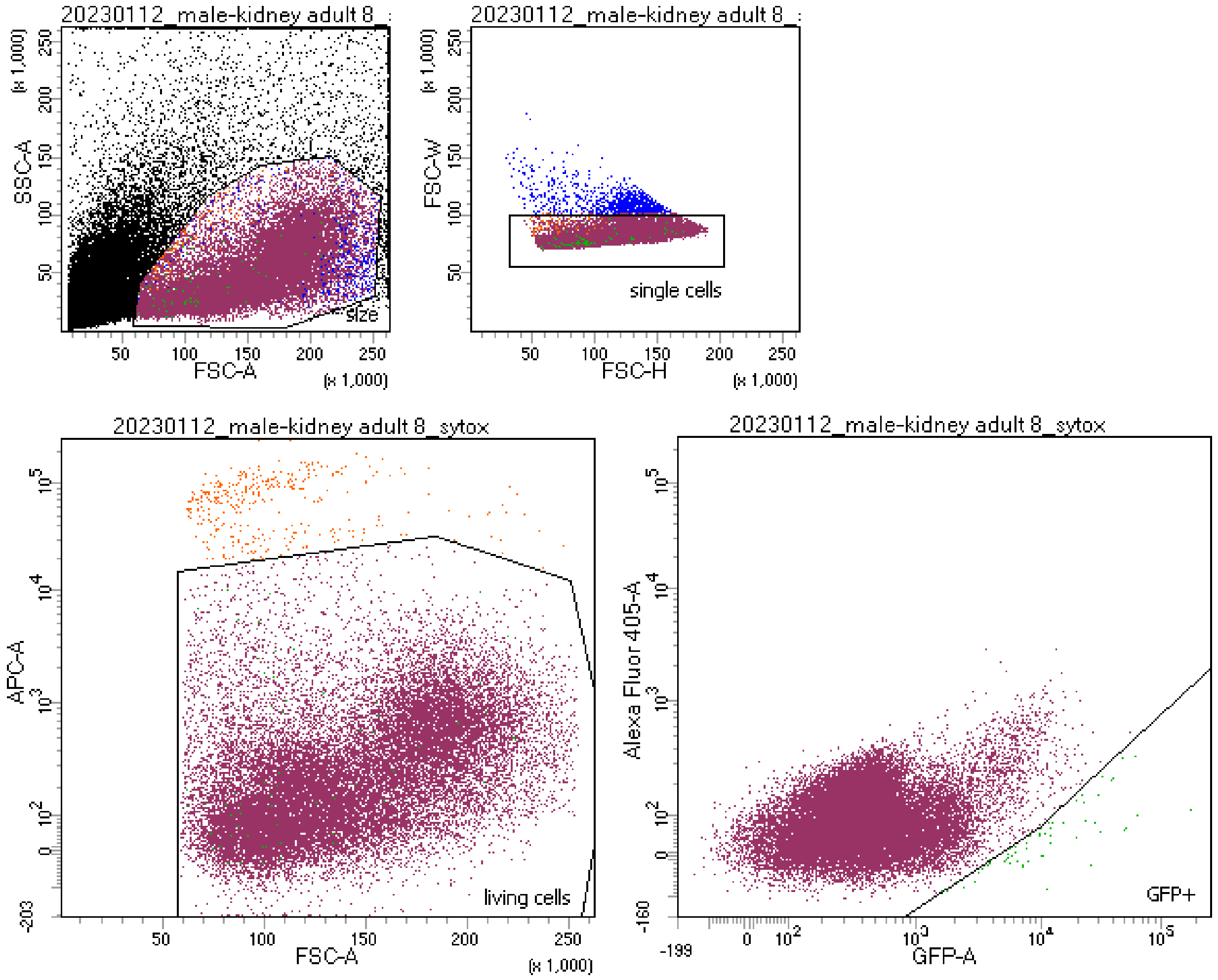

Tube: kidney adult 8_sytox

Population	#Events	%Parent	%Total
All Events	89,148	####	100.0
size	26,147	29.3	29.3
single cells	25,059	95.8	28.1
living cells	24,713	98.6	27.7
GFP+	62	0.3	0.1

Experiment Name: Johannes_Notho kidney
Specimen Name: 20230112_male
Tube Name: kidney adult 8_sytox

Population	#Events	%Parent	%Total	GFP-A Mean	GFP-A %CV
GFP+	62	0.2509	0.0695	17,926	147.7
living cells	24,713	98.6193	27.7213	769	270.1

BD FACSDiva 8.0

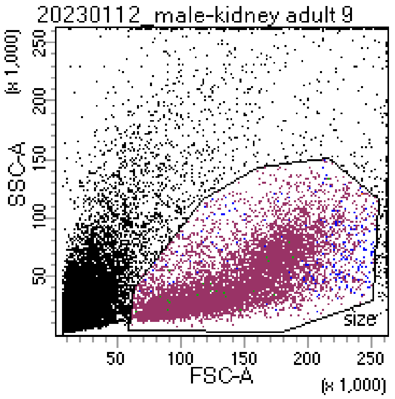

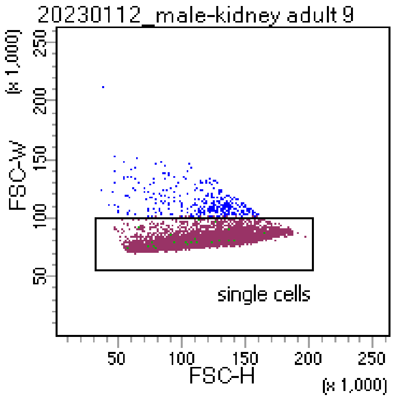

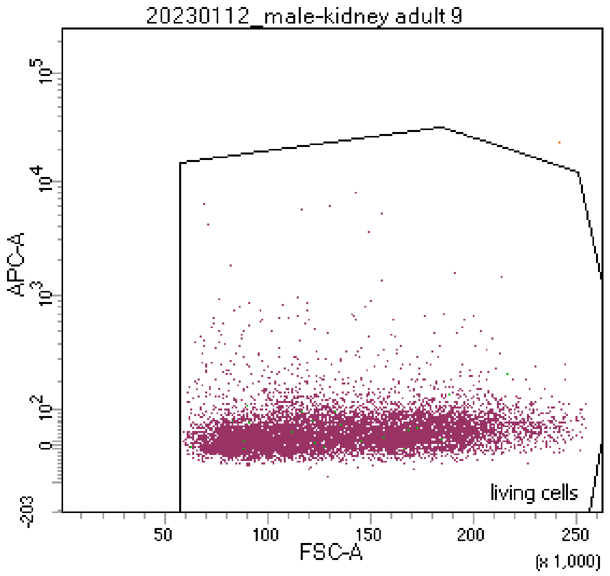

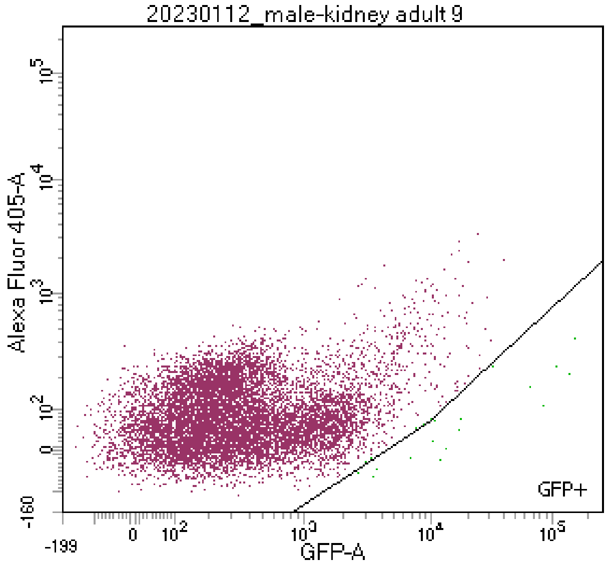

Tube: kidney adult 9

Population	#Events	%Parent	%Total
All Events	27,410	####	100.0
size	10,840	39.5	39.5
single cells	10,497	96.8	38.3
living cells	10,496	100.0	38.3
GFP+	21	0.2	0.1

Experiment Name: Johannes_Notho kidney
Specimen Name: 20230112_male
Tube Name: kidney adult 9

Population	#Events	%Parent	%Total	GFP-A Mean	GFP-A %CV
GFP+	21	0.2001	0.0766	34,270	139.7
living cells	10,496	99.9905	38.2926	762	396.0

BD FACSDiva 8.0

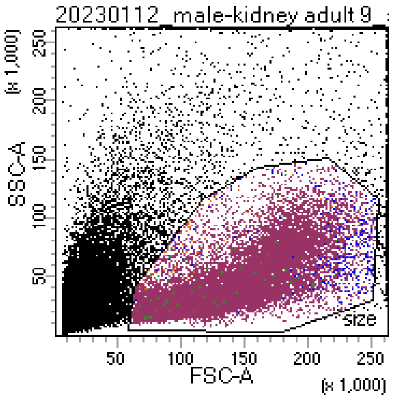

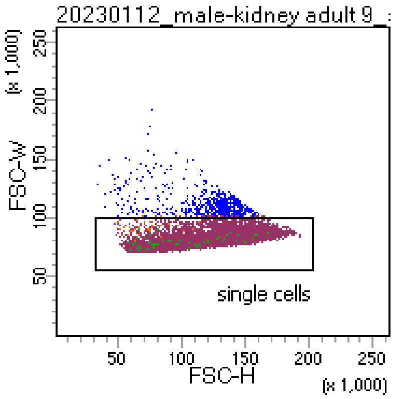

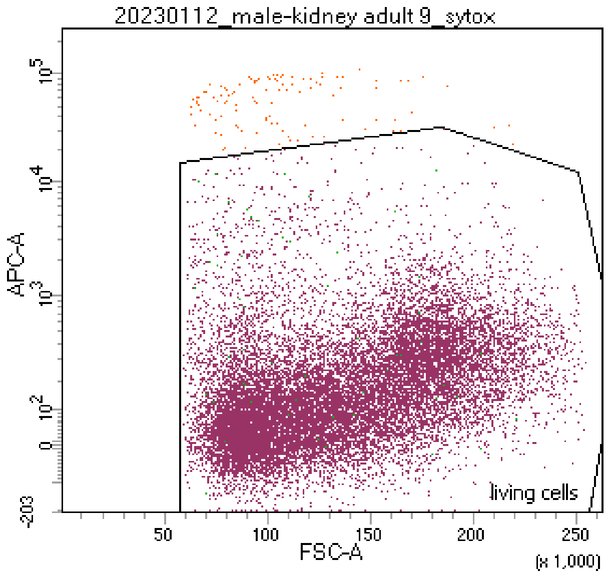

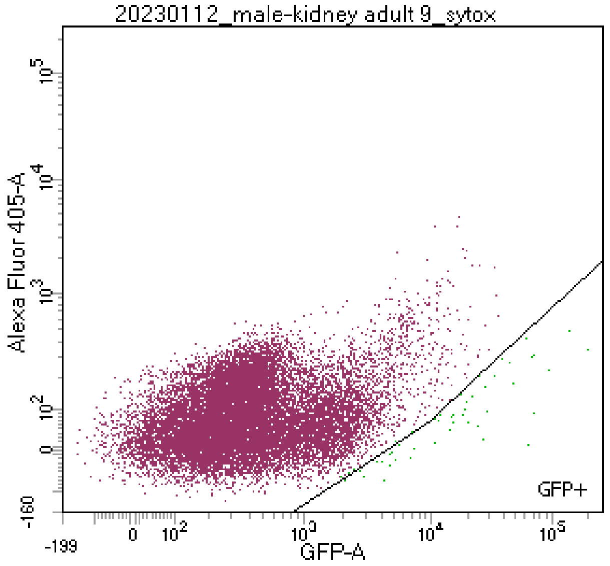

Tube: kidney adult 9_sytox

Population	#Events	%Parent	%Total
All Events	48,152	####	100.0
size	19,248	40.0	40.0
single cells	18,668	97.0	38.8
living cells	18,562	99.4	38.5
GFP+	54	0.3	0.1

Experiment Name: Johannes_Notho kidney
Specimen Name: 20230112_male
Tube Name: kidney adult 9_sytox

Population	#Events	%Parent	%Total	GFP-A Mean	GFP-A %CV
GFP+	54	0.2909	0.1121	26,179	139.4
living cells	18,562	99.4322	38.5488	800	348.0

BD FACSDiva 8.0

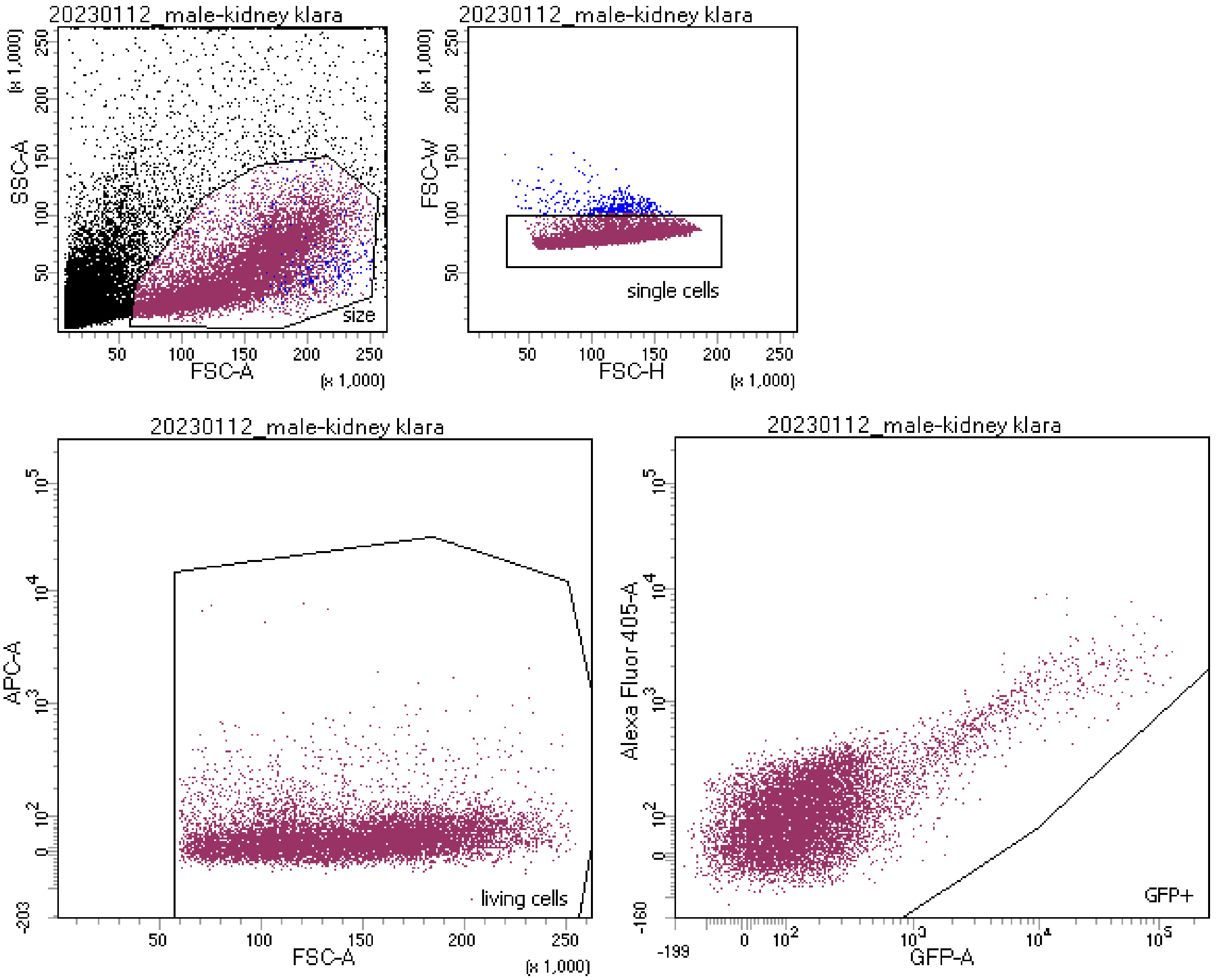

Tube: kidney klara

Population	#Events	%Parent	%Total
All Events	29,598	####	100.0
size	11,138	37.6	37.6
single cells	10,659	95.7	36.0
living cells	10,659	100.0	36.0
GFP+	0	0.0	0.0

Experiment Name: Johannes_Notho kidney
Specimen Name: 20230112_male
Tube Name: kidney klara

Population	#Events	%Parent	%Total	GFP-A Mean	GFP-A %CV
GFP+	0	0.0000	0.0000	####	####
living cells	10,659	100.0000	36.0126	826	651.8

BD FACSDiva 8.0

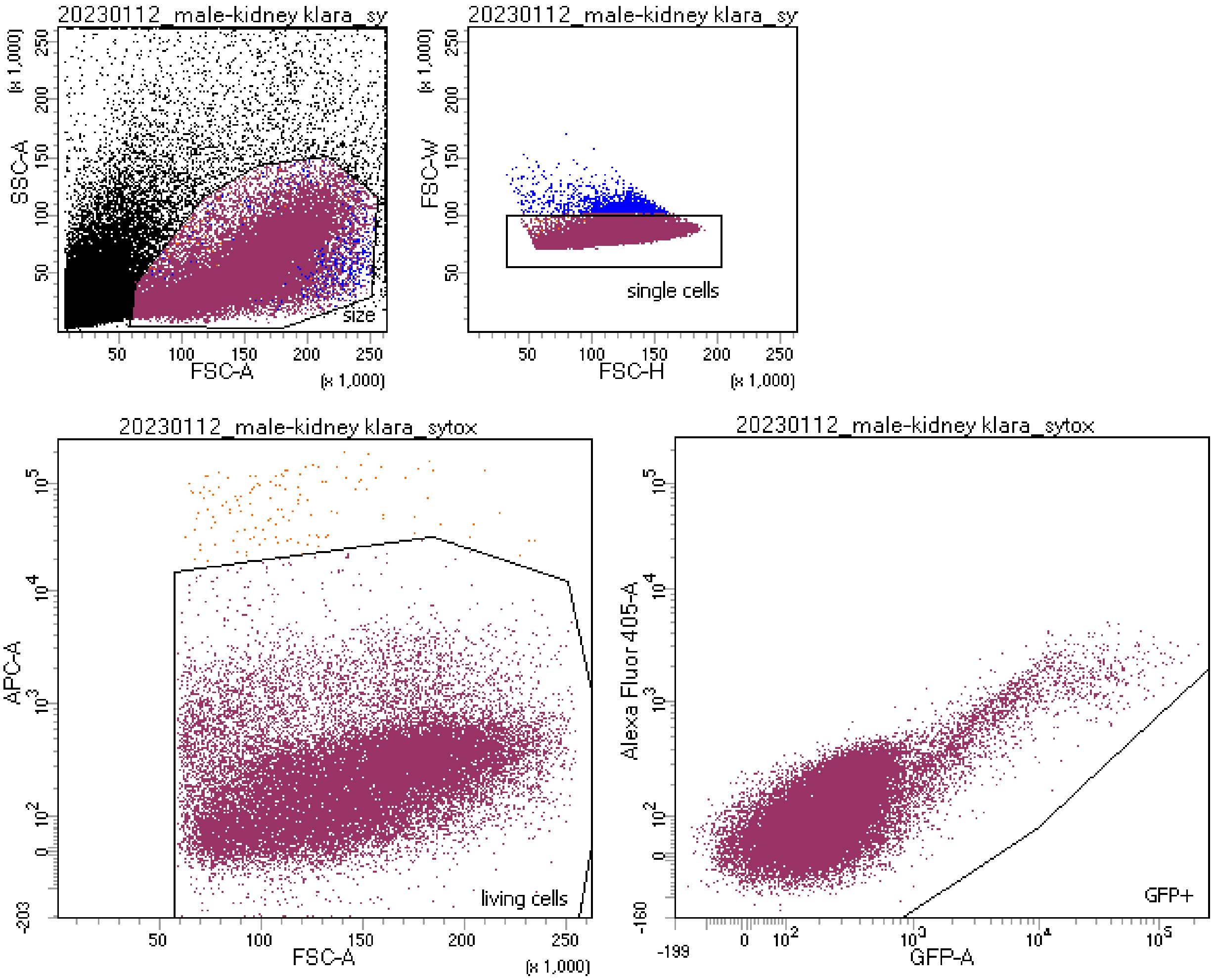

Tube: kidney klara_sytox

Population	#Events	%Parent	%Total
All Events	83,100	####	100.0
size	31,640	38.1	38.1
single cells	30,198	95.4	36.3
living cells	30,067	99.6	36.2
GFP+	0	0.0	0.0

Experiment Name: Johannes_Notho kidney
Specimen Name: 20230112_male
Tube Name: kidney klara_sytox

Population	#Events	%Parent	%Total	GFP-A Mean	GFP-A %CV
GFP+	0	0.0000	0.0000	####	####
living cells	30,067	99.5662	36.1817	811	606.1

BD FACSDiva 8.0

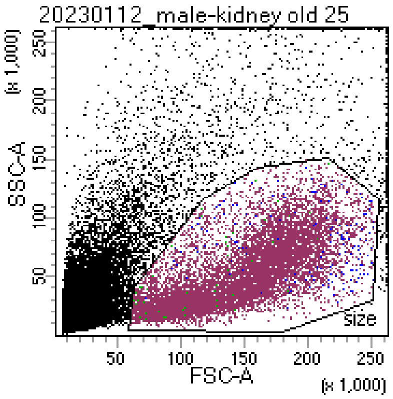

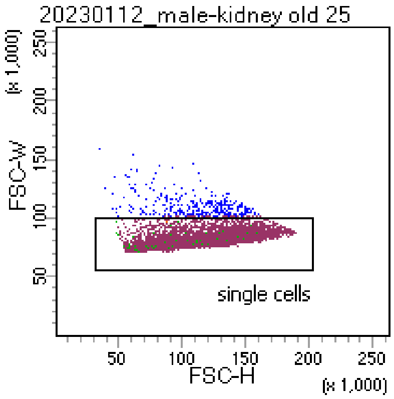

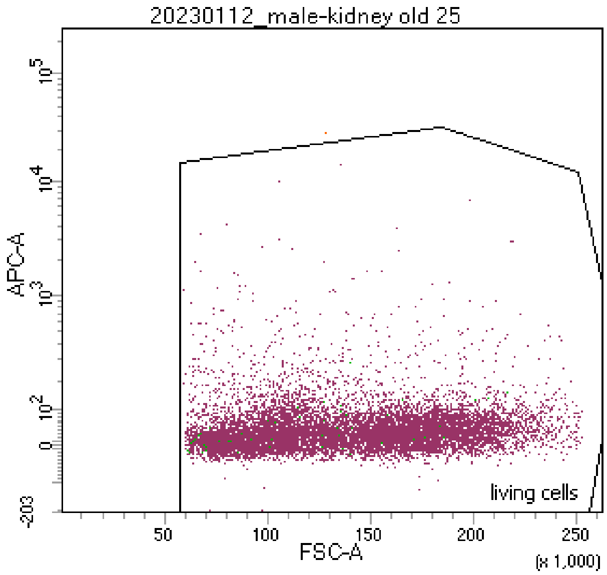

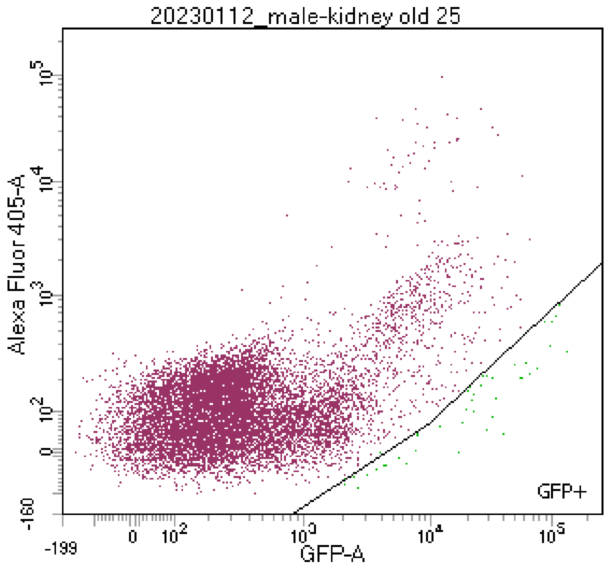

Tube: kidney old 25

Population	#Events	%Parent	%Total
All Events	30,409	####	100.0
size	11,059	36.4	36.4
single cells	10,747	97.2	35.3
living cells	10,746	100.0	35.3
GFP+	47	0.4	0.2

Experiment Name: Johannes_Notho kidney
Specimen Name: 20230112_male
Tube Name: kidney old 25

Population	#Events	%Parent	%Total	GFP-A Mean	GFP-A %CV
GFP+	47	0.4374	0.1546	33,815	103.6
living cells	10,746	99.9907	35.3382	1,094	394.5

BD FACSDiva 8.0

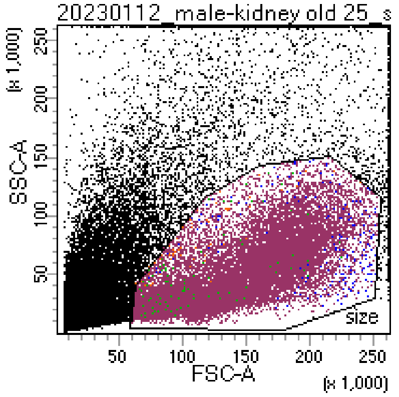

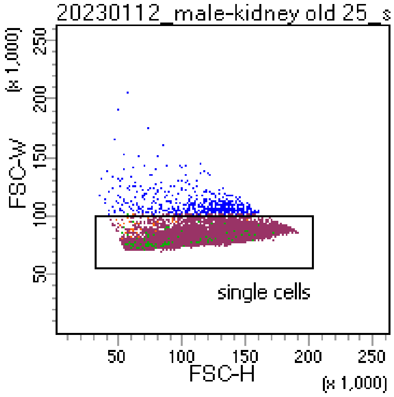

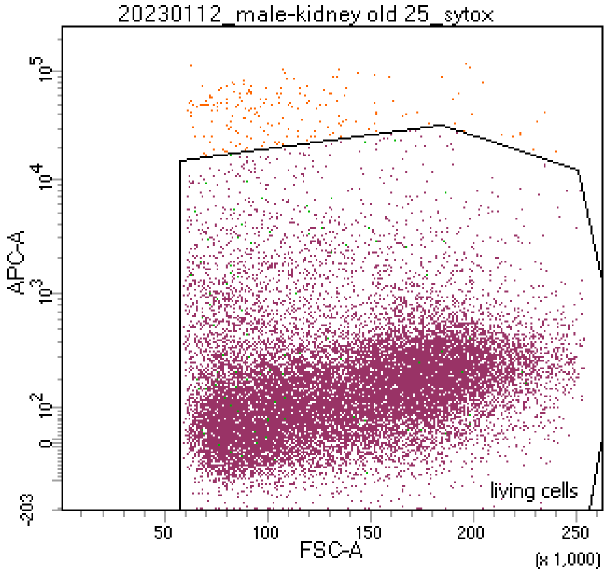

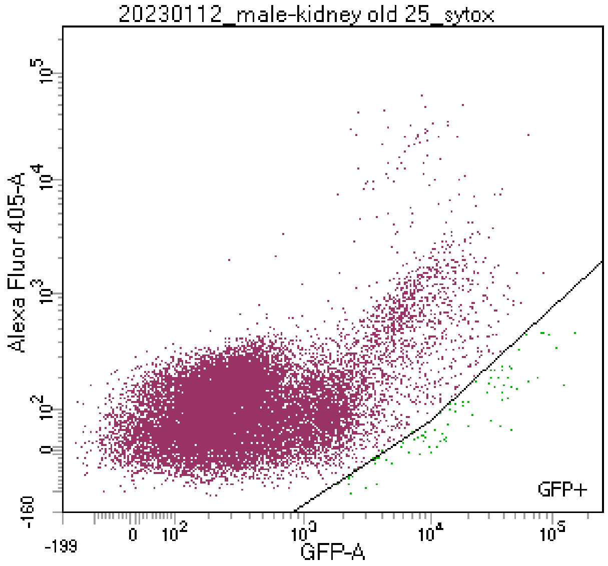

Tube: kidney old 25_sytox

Population	#Events	%Parent	%Total
All Events	59,388	####	100.0
size	21,123	35.6	35.6
single cells	20,564	97.4	34.6
living cells	20,370	99.1	34.3
GFP+	91	0.4	0.2

Experiment Name: Johannes_Notho kidney
Specimen Name: 20230112_male
Tube Name: kidney old 25_sytox

Population	#Events	%Parent	%Total	GFP-A Mean	GFP-A %CV
GFP+	91	0.4467	0.1532	25,451	113.9
living cells	20,370	99.0566	34.2999	1,080	348.5

BD FACSDiva 8.0

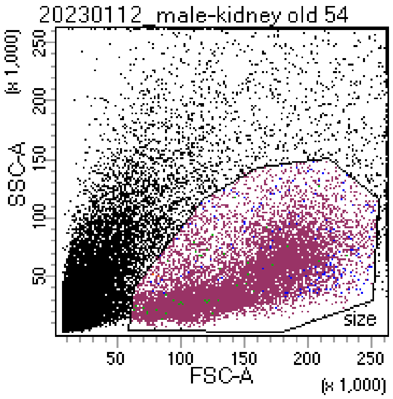

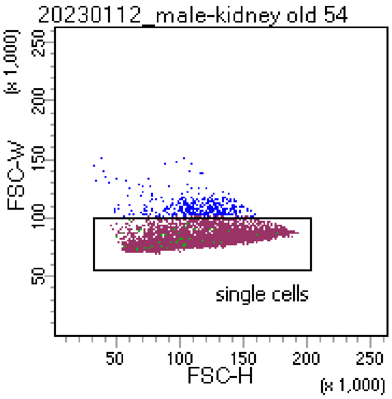

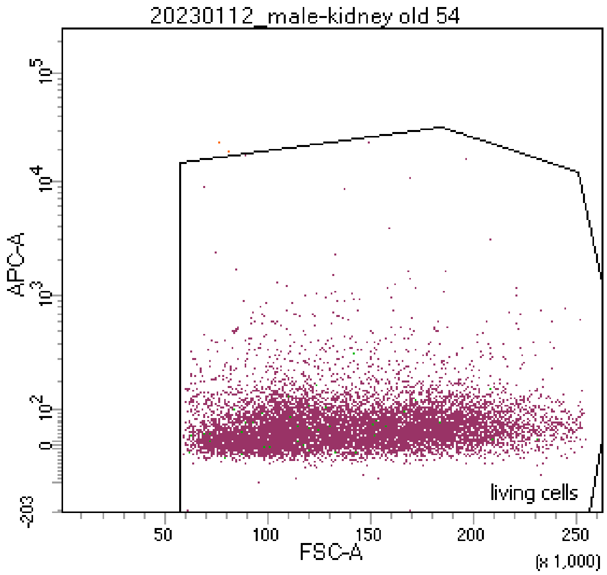

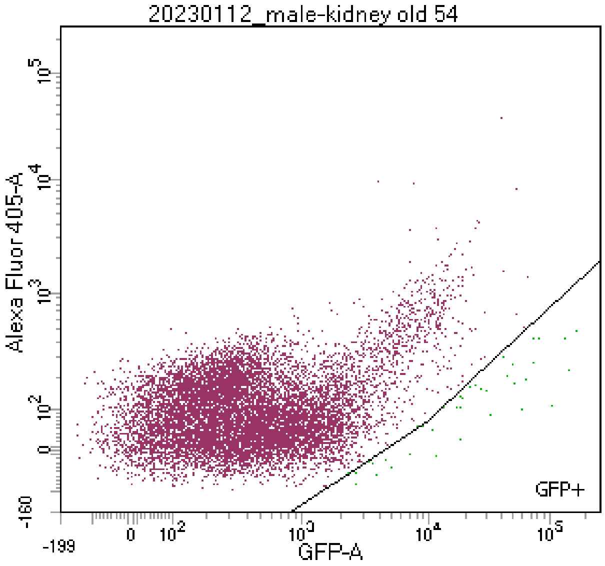

Tube: kidney old 54

Population	#Events	%Parent	%Total
All Events	33,047	####	100.0
size	11,371	34.4	34.4
single cells	10,982	96.6	33.2
living cells	10,979	100.0	33.2
GFP+	39	0.4	0.1

Experiment Name:	Johannes_Notho kidney
Specimen Name:	20230112_male
Tube Name:	kidney old 54

Population	#Events	%Parent	%Total	GFP-A Mean	GFP-A %CV
GFP+	39	0.3552	0.1180	36,569	116.7
living cells	10,979	99.9727	33.2224	1,077	376.4

BD FACSDiva 8.0

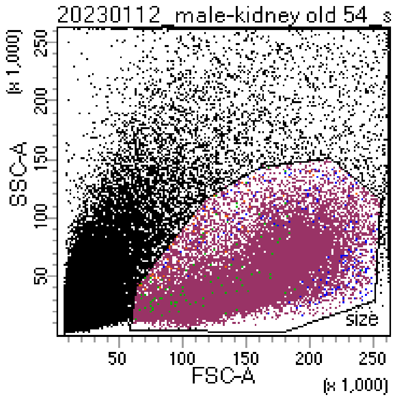

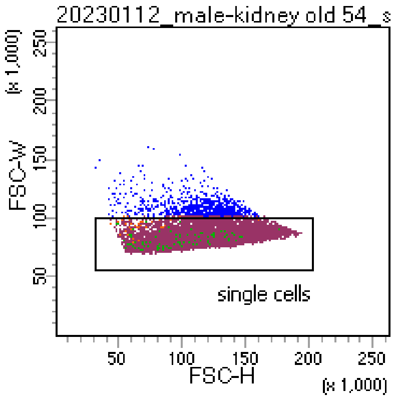

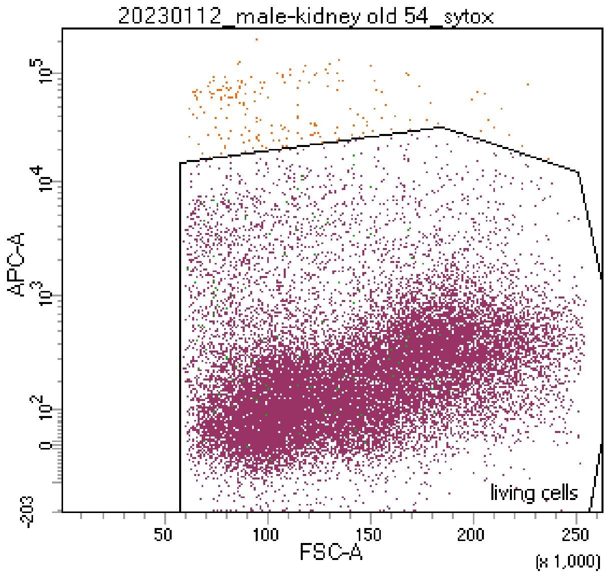

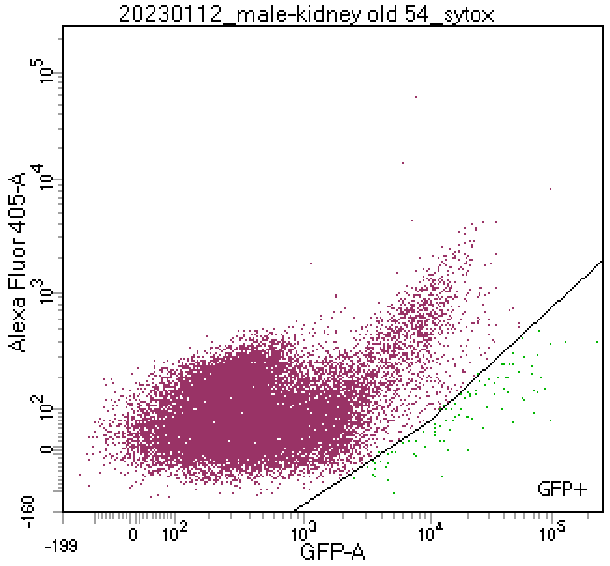

Tube: kidney old 54_sytox

Population	#Events	%Parent	%Total
All Events	71,787	####	100.0
size	24,514	34.1	34.1
single cells	23,720	96.8	33.0
living cells	23,527	99.2	32.8
GFP+	98	0.4	0.1

Experiment Name: Johannes_Notho kidney
Specimen Name: 20230112_male
Tube Name: kidney old 54_sytox

Population	#Events	%Parent	%Total	GFP-A Mean	GFP-A %CV
GFP+	98	0.4165	0.1365	30,053	114.2
living cells	23,527	99.1863	32.7733	1,126	332.7

BD FACSDiva 8.0

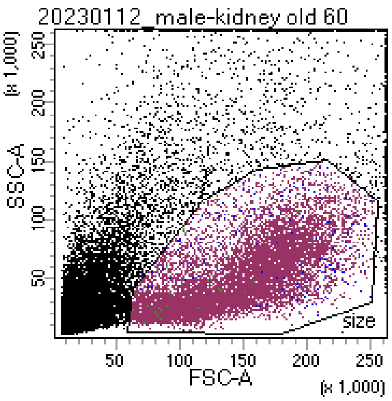

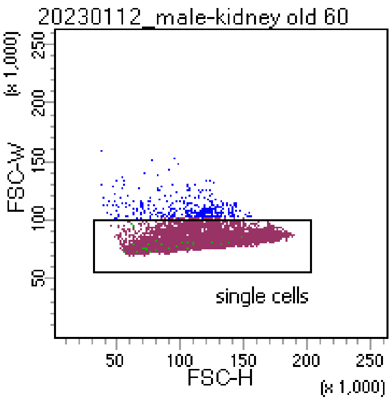

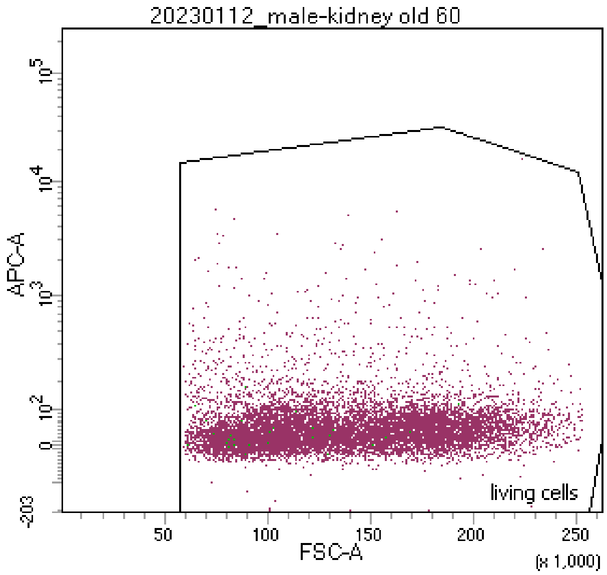

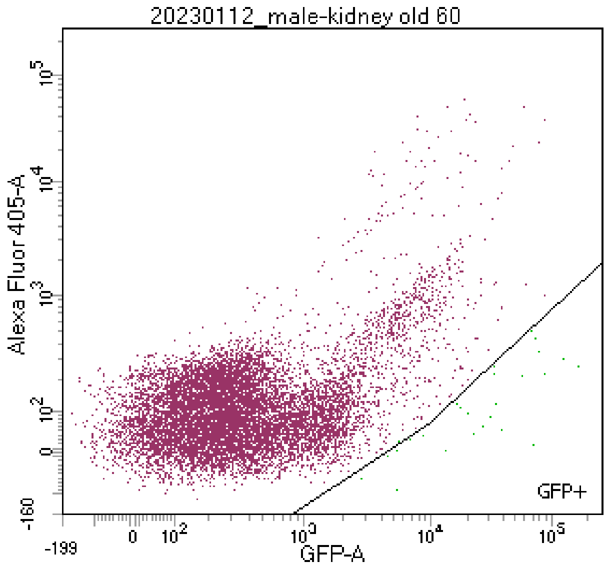

Tube: kidney old 60

Population	#Events	%Parent	%Total
All Events	28,619	####	100.0
size	11,327	39.6	39.6
single cells	10,944	96.6	38.2
living cells	10,944	100.0	38.2
GFP+	27	0.2	0.1

Experiment Name: Johannes_Notho kidney
Specimen Name: 20230112_male
Tube Name: kidney old 60

Population	#Events	%Parent	%Total	GFP-A Mean	GFP-A %CV
GFP+	27	0.2467	0.0943	39,482	104.3
living cells	10,944	100.0000	38.2403	1,050	384.1

BD FACSDiva 8.0

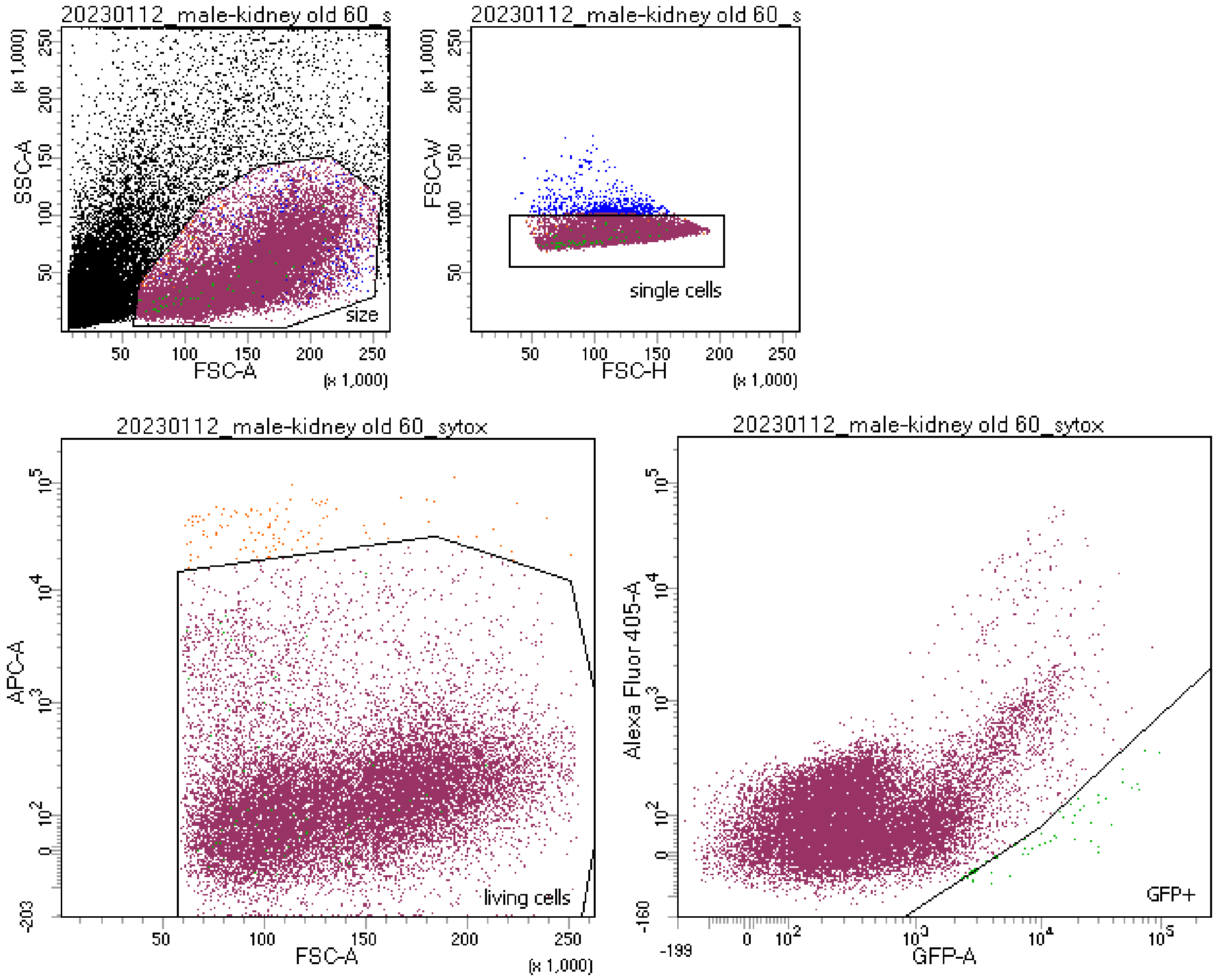

Tube: kidney old 60_sytox

Population	#Events	%Parent	%Total
All Events	52,205	####	100.0
size	20,756	39.8	39.8
single cells	20,102	96.8	38.5
living cells	19,990	99.4	38.3
GFP+	57	0.3	0.1

Experiment Name: Johannes_Notho kidney
Specimen Name: 20230112_male
Tube Name: kidney old 60_sytox

Population	#Events	%Parent	%Total	GFP-A Mean	GFP-A %CV
GFP+	57	0.2851	0.1092	18,954	109.5
living cells	19,990	99.4428	38.2914	934	289.1

BD FACSDiva 8.0

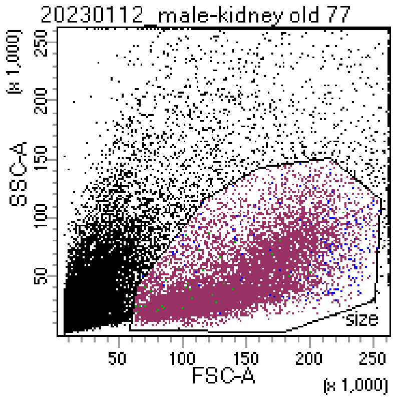

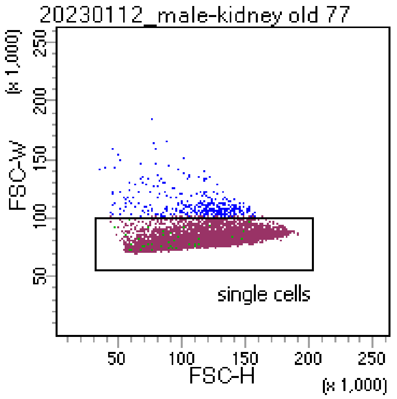

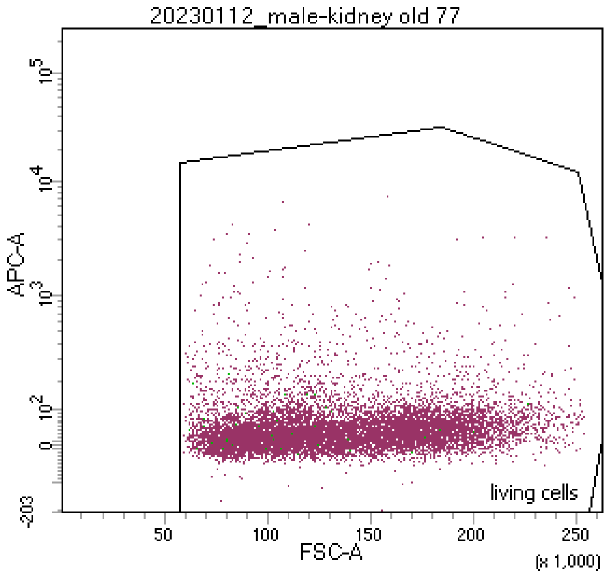

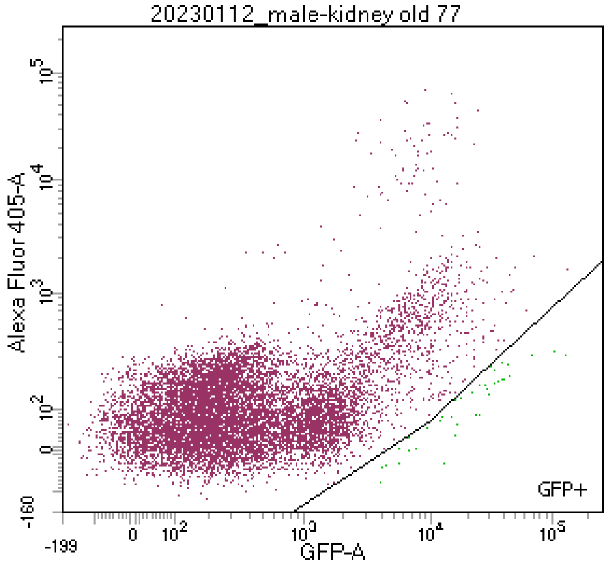

Tube: kidney old 77

Population	#Events	%Parent	%Total
All Events	32,601	####	100.0
size	11,300	34.7	34.7
single cells	10,941	96.8	33.6
living cells	10,941	100.0	33.6
GFP+	37	0.3	0.1

Experiment Name: Johannes_Notho kidney
Specimen Name: 20230112_male
Tube Name: kidney old 77

Population	#Events	%Parent	%Total	GFP-A Mean	GFP-A %CV
GFP+	37	0.3382	0.1135	27,165	100.3
living cells	10,941	100.0000	33.5603	1,220	323.1

BD FACSDiva 8.0

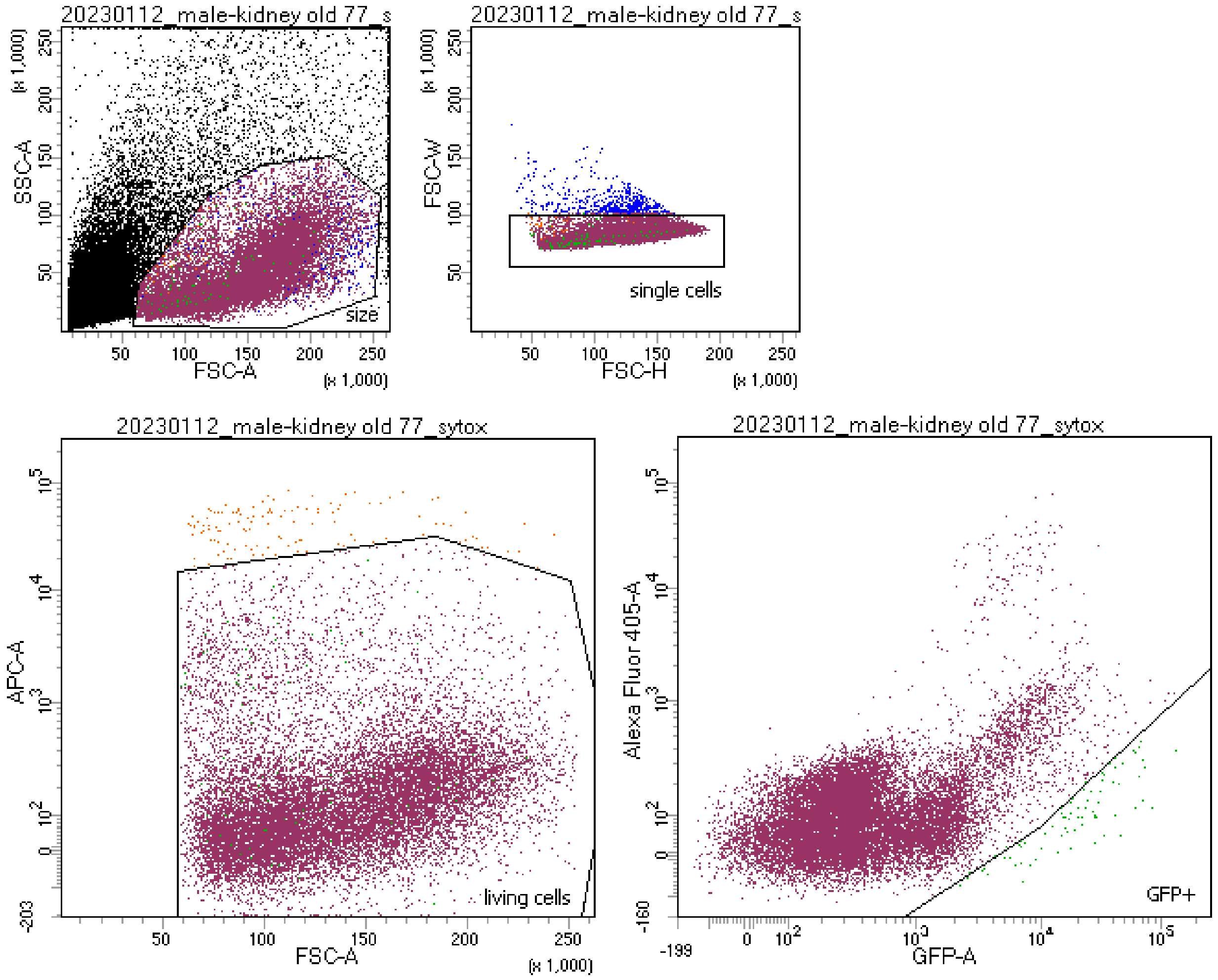

Tube: kidney old 77_sytox

Population	#Events	%Parent	%Total
All Events	52,348	####	100.0
size	18,441	35.2	35.2
single cells	17,907	97.1	34.2
living cells	17,783	99.3	34.0
GFP+	80	0.4	0.2

Experiment Name: Johannes_Notho kidney
Specimen Name: 20230112_male
Tube Name: kidney old 77_sytox

Population	#Events	%Parent	%Total	GFP-A Mean	GFP-A %CV
GFP+	80	0.4499	0.1528	26,827	88.2
living cells	17,783	99.3075	33.9707	1,266	322.5

BD FACSDiva 8.0

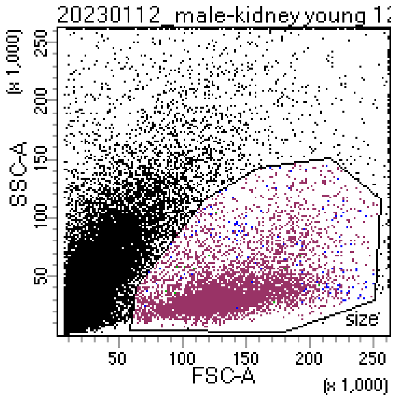

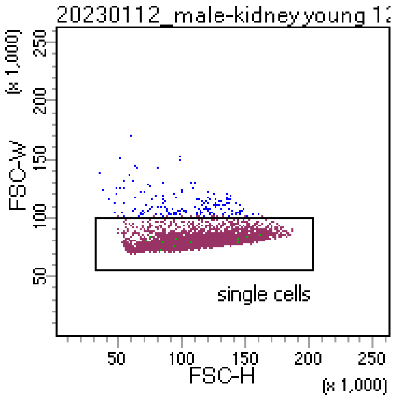

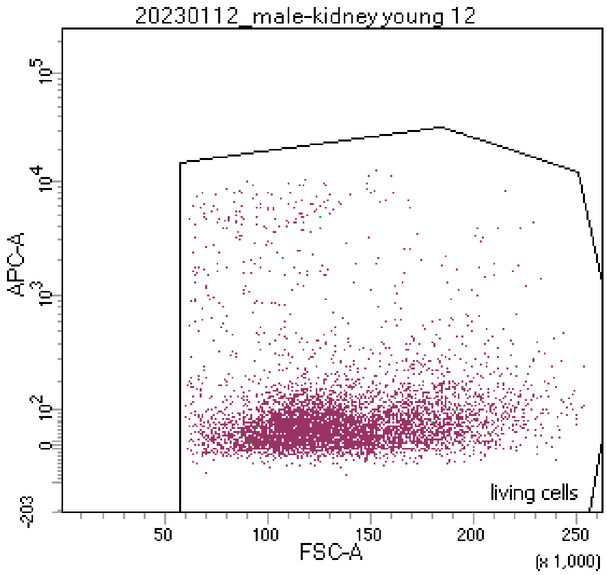

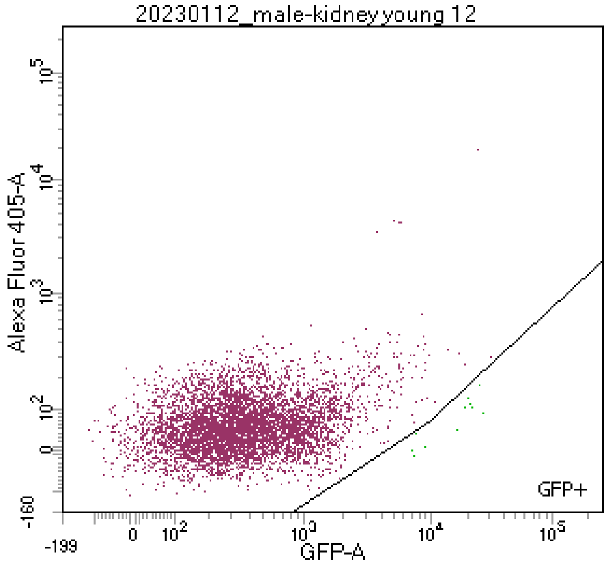

Tube: kidney young 12

Population	#Events	%Parent	%Total
All Events	58,459	####	100.0
size	5,972	10.2	10.2
single cells	5,813	97.3	9.9
living cells	5,813	100.0	9.9
GFP+	11	0.2	0.0

Experiment Name: Johannes_Notho kidney
Specimen Name: 20230112_male
Tube Name: kidney young 12

Population	#Events	%Parent	%Total	GFP-A Mean	GFP-A %CV
GFP+	11	0.1892	0.0188	16,434	46.2
living cells	5,813	100.0000	9.9437	595	210.0

BD FACSDiva 8.0

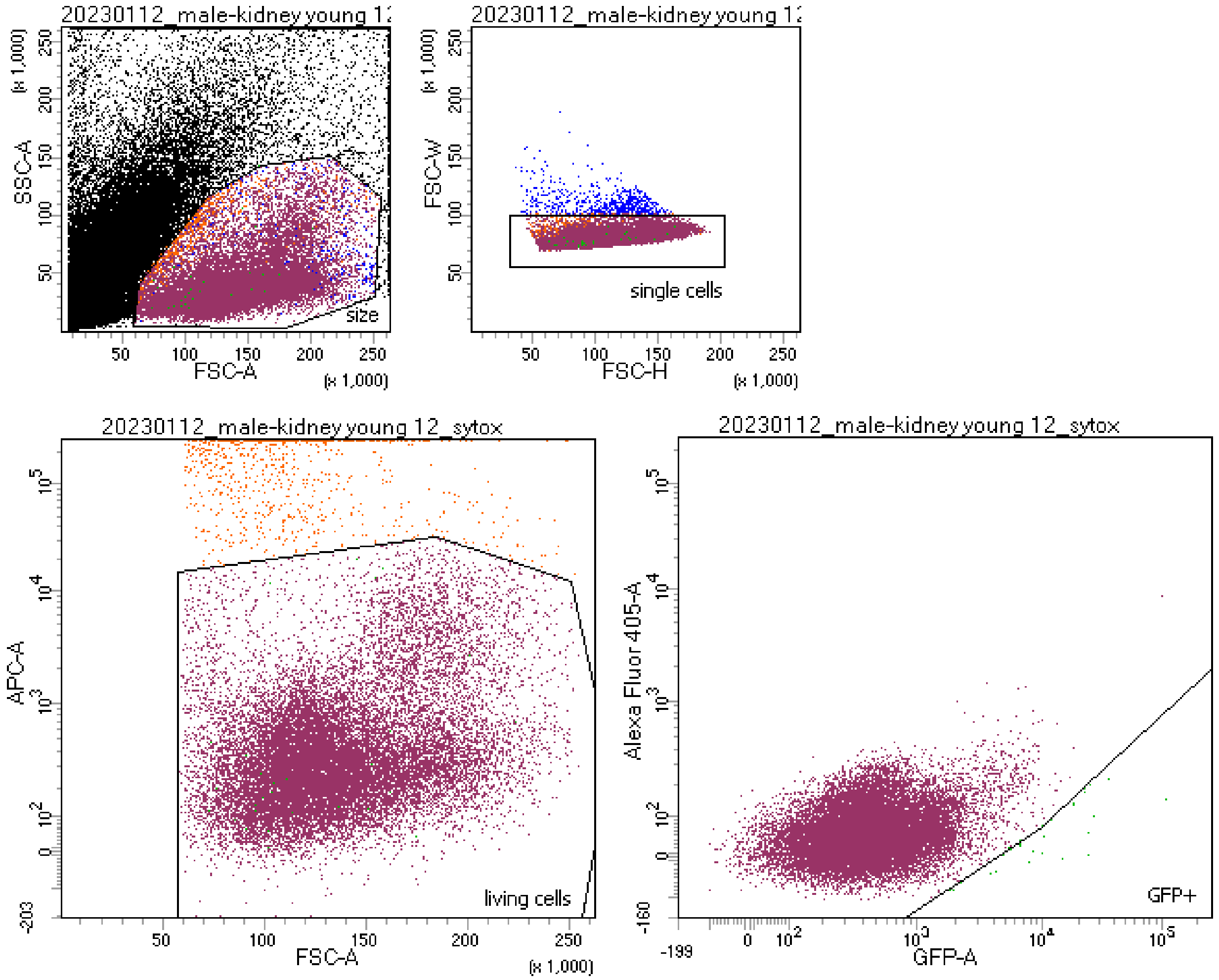

Tube: kidney young 12_sytox

Population	#Events	%Parent	%Total
All Events	210,179	####	100.0
size	22,645	10.8	10.8
single cells	22,084	97.5	10.5
living cells	21,262	96.3	10.1
GFP+	28	0.1	0.0

Experiment Name: Johannes_Notho kidney
Specimen Name: 20230112_male
Tube Name: kidney young 12_sytox

Population	#Events	%Parent	%Total	GFP-A Mean	GFP-A %CV
GFP+	28	0.1317	0.0133	16,158	127.5
living cells	21,262	96.2778	10.1161	644	220.3

BD FACSDiva 8.0

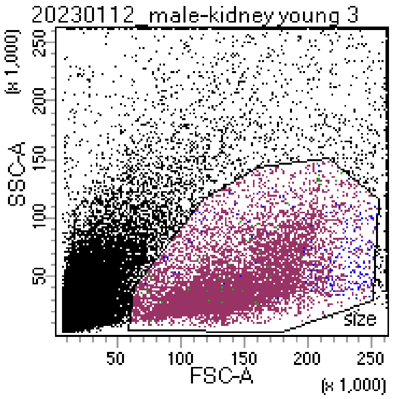

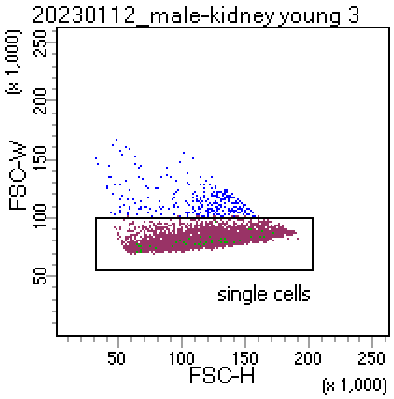

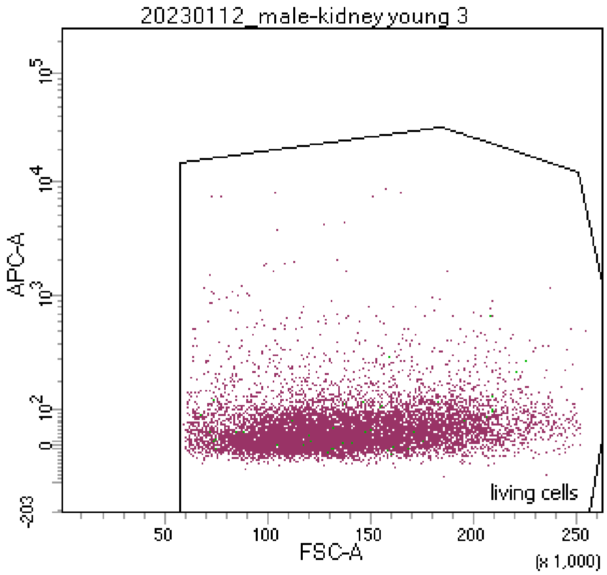

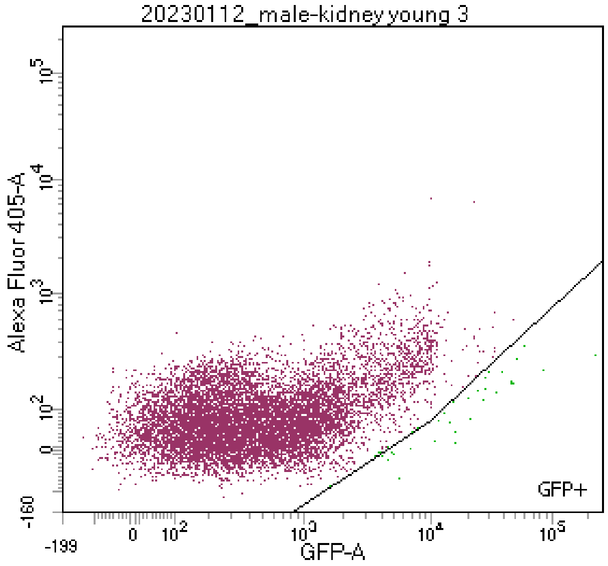

Tube: kidney young 3

Population	#Events	%Parent	%Total
All Events	40,830	####	100.0
size	10,994	26.9	26.9
single cells	10,711	97.4	26.2
living cells	10,711	100.0	26.2
GFP+	41	0.4	0.1

Experiment Name: Johannes_Notho kidney
Specimen Name: 20230112_male
Tube Name: kidney young 3

Population	#Events	%Parent	%Total	GFP-A Mean	GFP-A %CV
GFP+	41	0.3828	0.1004	25,651	150.5
living cells	10,711	100.0000	26.2332	1,067	321.0

BD FACSDiva 8.0

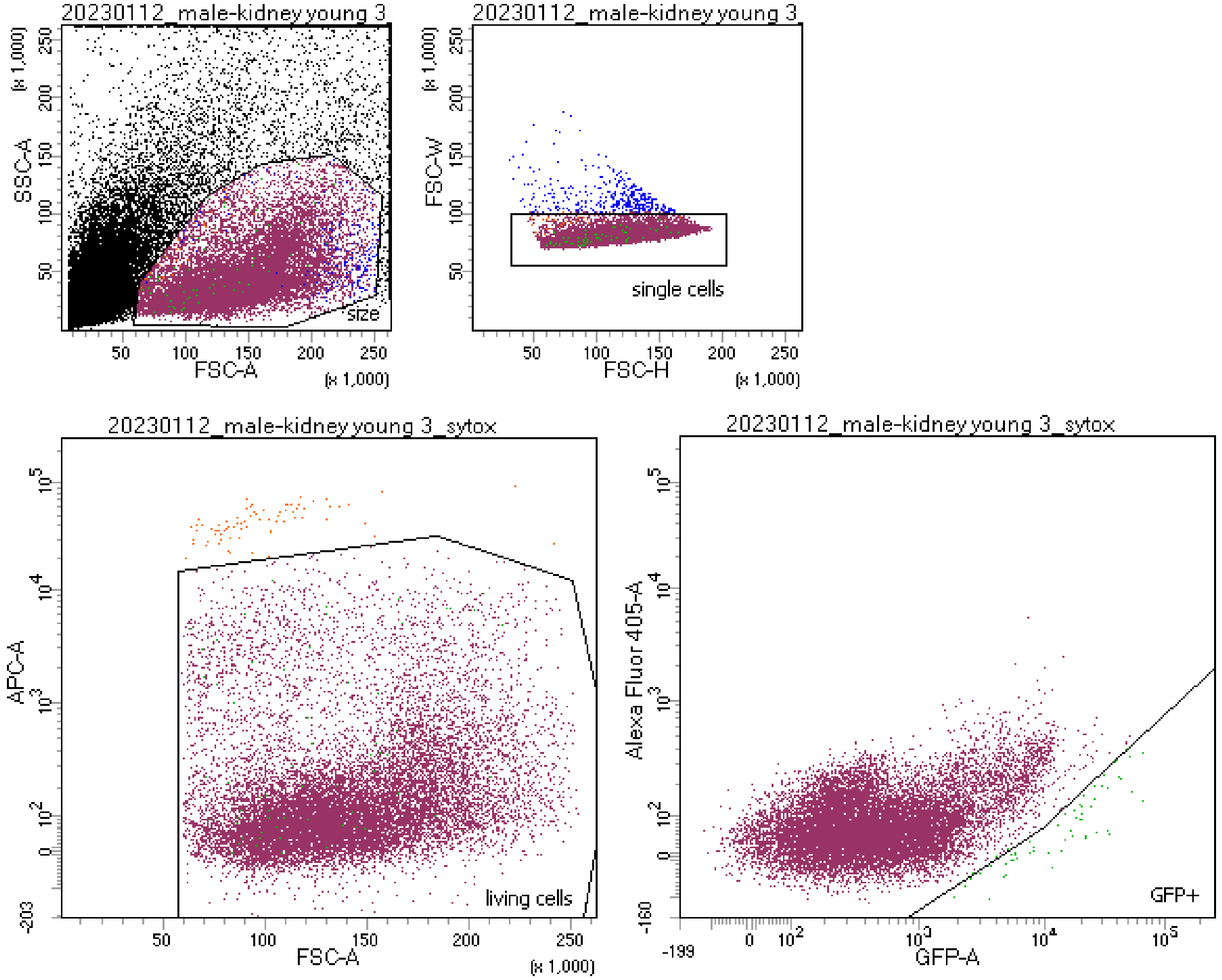

Tube: kidney young 3_sytox

Population	#Events	%Parent	%Total
All Events	61,096	####	100.0
size	16,302	26.7	26.7
single cells	15,904	97.6	26.0
living cells	15,828	99.5	25.9
GFP+	64	0.4	0.1

Experiment Name: Johannes_Notho kidney
Specimen Name: 20230112_male
Tube Name: kidney young 3_sytox

Population	#Events	%Parent	%Total	GFP-A Mean	GFP-A %CV
GFP+	64	0.4043	0.1048	18,021	90.9
living cells	15,828	99.5221	25.9068	1,044	232.9

BD FACSDiva 8.0

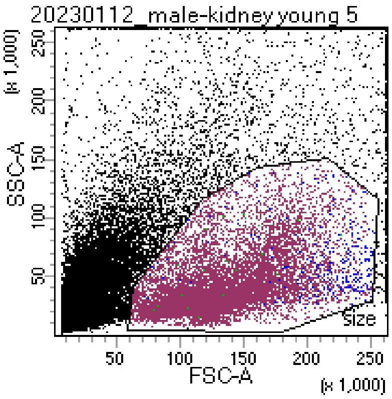

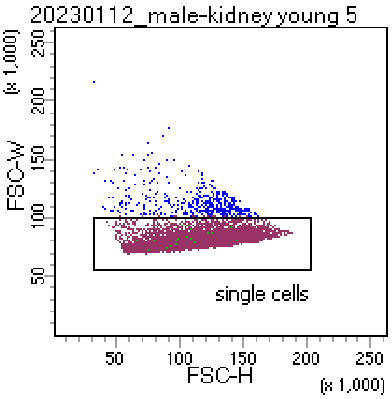

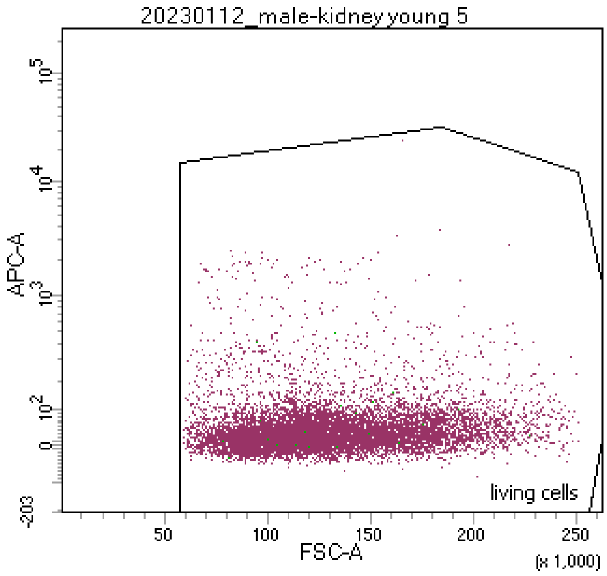

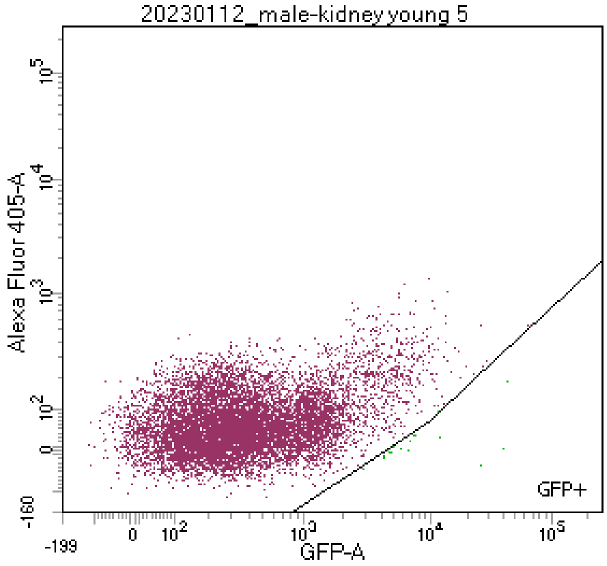

Tube: kidney young 5

Population	#Events	%Parent	%Total
All Events	42,323	####	100.0
size	11,112	26.3	26.3
single cells	10,644	95.8	25.1
living cells	10,644	100.0	25.1
GFP+	20	0.2	0.0

Experiment Name: Johannes_Notho kidney
Specimen Name: 20230112_male
Tube Name: kidney young 5

Population	#Events	%Parent	%Total	GFP-A Mean	GFP-A %CV
GFP+	20	0.1879	0.0473	11,098	106.1
living cells	10,644	100.0000	25.1494	713	222.2

BD FACSDiva 8.0

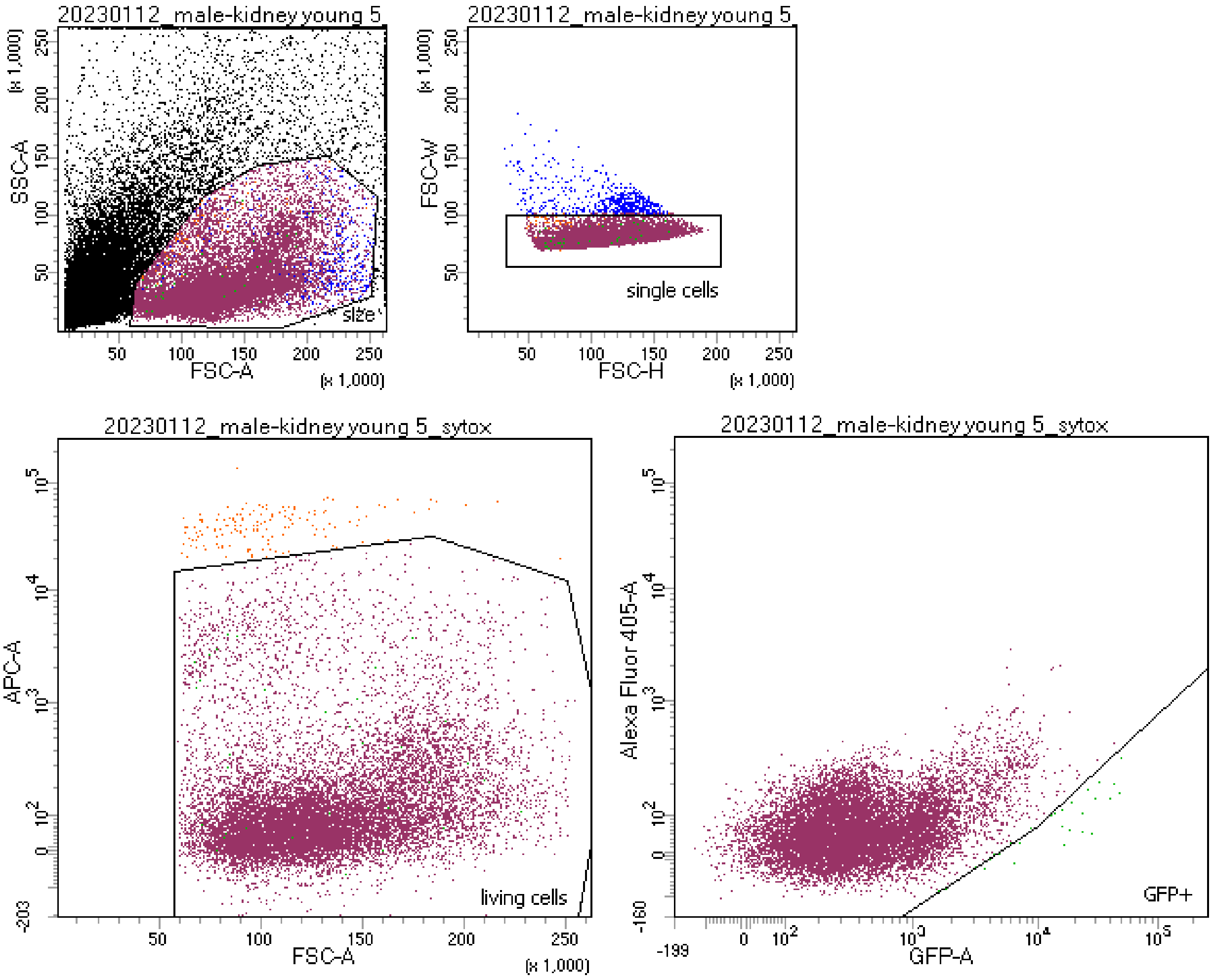

Tube: kidney young 5_sytox

Population	#Events	%Parent	%Total
All Events	58,062	####	100.0
size	15,082	26.0	26.0
single cells	14,499	96.1	25.0
living cells	14,348	99.0	24.7
GFP+	33	0.2	0.1

Experiment Name: Johannes_Notho kidney
Specimen Name: 20230112_male
Tube Name: kidney young 5_sytox

Population	#Events	%Parent	%Total	GFP-A Mean	GFP-A %CV
GFP+	33	0.2300	0.0568	17,701	79.8
living cells	14,348	98.9585	24.7115	738	223.2

BD FACSDiva 8.0

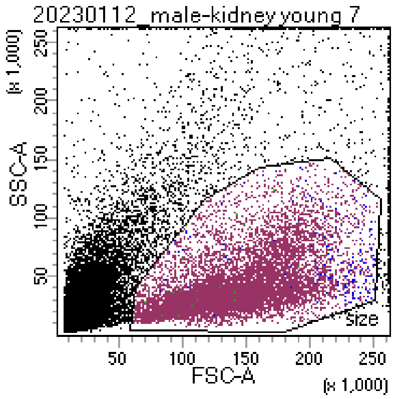

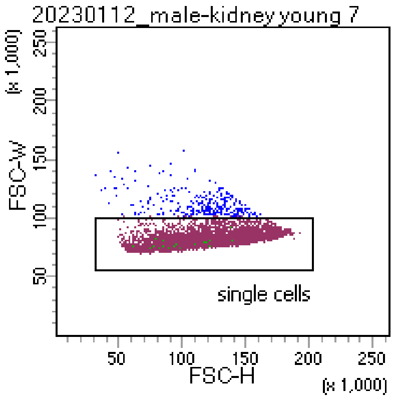

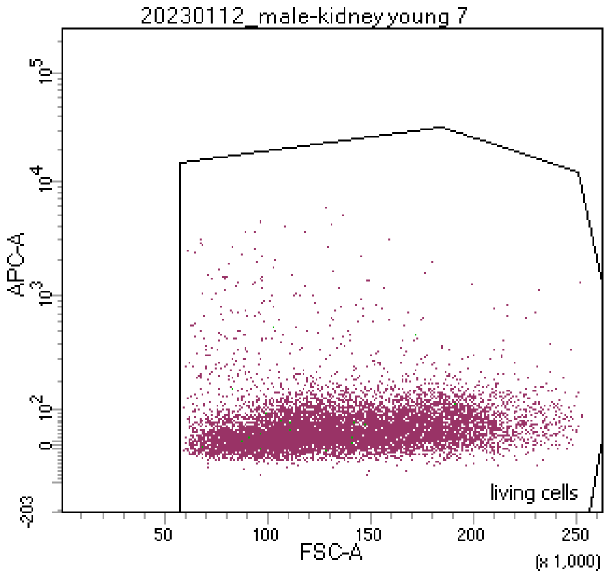

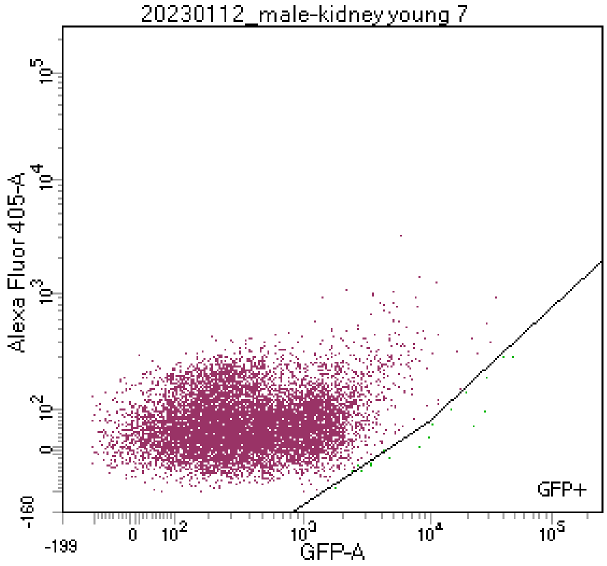

Tube: kidney young 7

Population	#Events	%Parent	%Total
All Events	30,663	####	100.0
size	10,932	35.7	35.7
single cells	10,641	97.3	34.7
living cells	10,641	100.0	34.7
GFP+	18	0.2	0.1

Experiment Name: Johannes_Notho kidney
Specimen Name: 20230112_male
Tube Name: kidney young 7

Population	#Events	%Parent	%Total	GFP-A Mean	GFP-A %CV
GFP+	18	0.1692	0.0587	14,122	99.6
living cells	10,641	100.0000	34.7031	652	198.7

BD FACSDiva 8.0

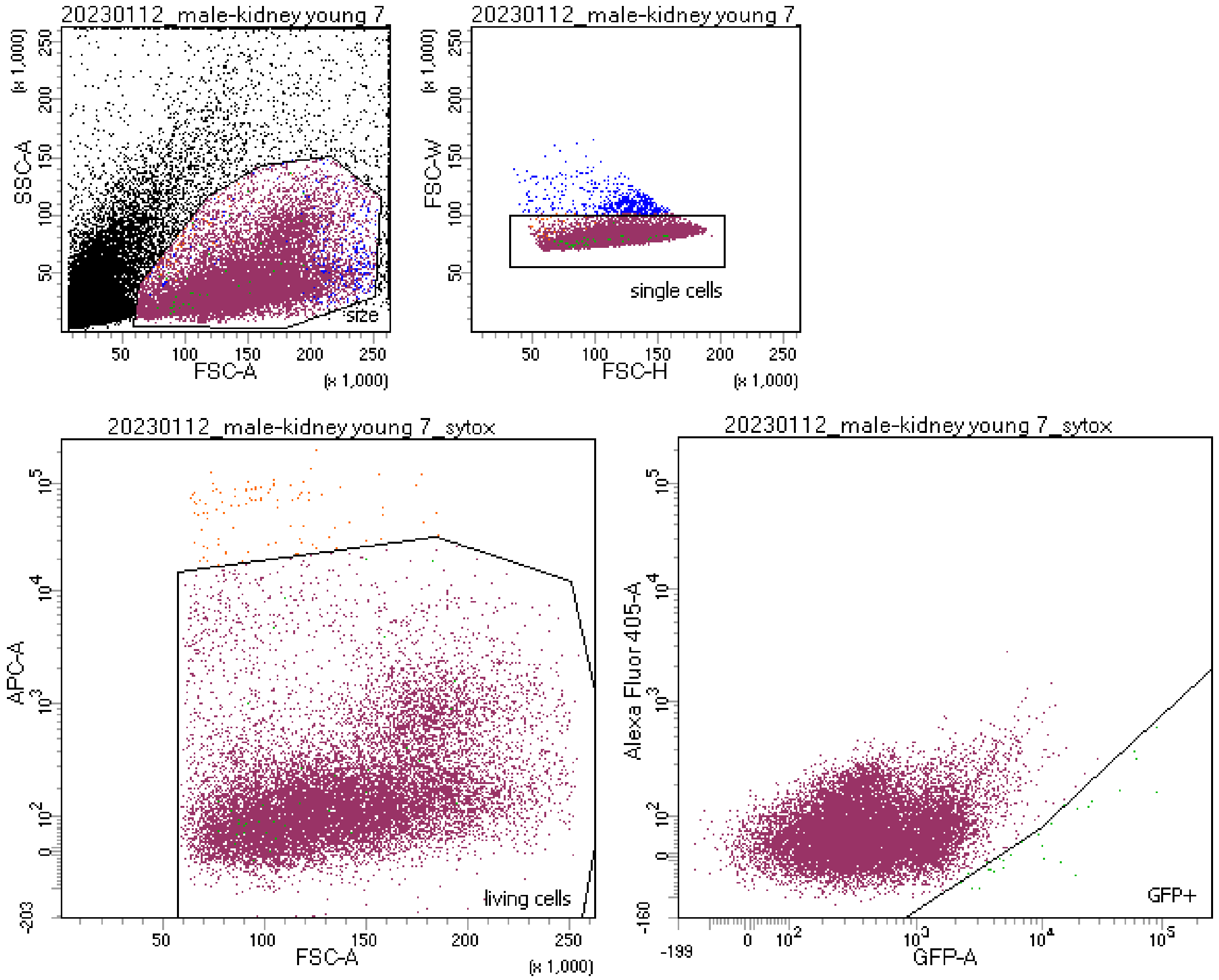

Tube: kidney young 7_sytox

Population	#Events	%Parent	%Total
All Events	52,491	####	100.0
size	18,095	34.5	34.5
single cells	17,562	97.1	33.5
living cells	17,461	99.4	33.3
GFP+	36	0.2	0.1

Experiment Name: Johannes_Notho kidney
Specimen Name: 20230112_male
Tube Name: kidney young 7_sytox

Population	#Events	%Parent	%Total	GFP-A Mean	GFP-A %CV
GFP+	36	0.2062	0.0686	16,218	146.6
living cells	17,461	99.4249	33.2648	700	222.8

BD FACSDiva 8.0

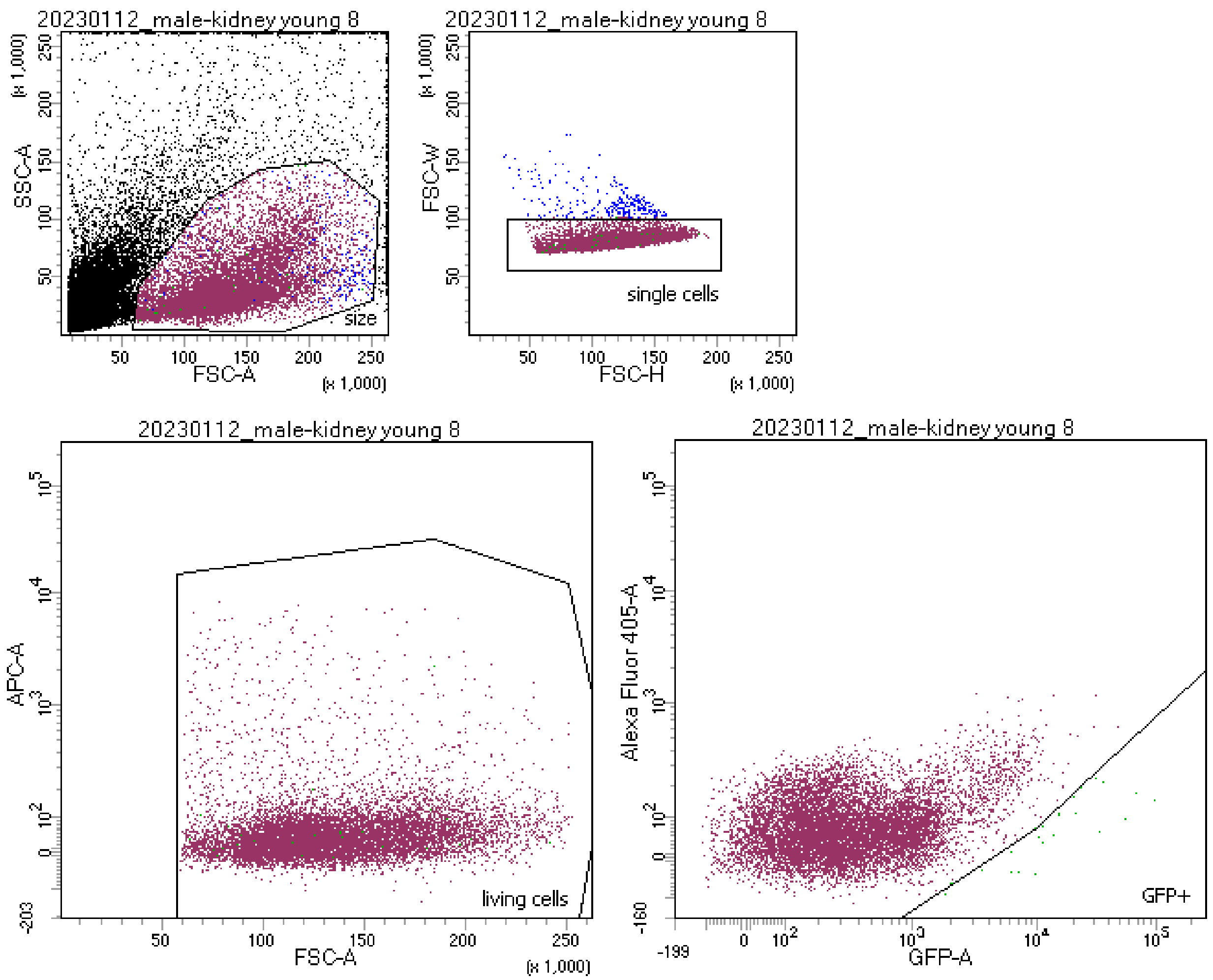

Tube: kidney young 8

Population	#Events	%Parent	%Total
All Events	38,263	####	100.0
size	11,133	29.1	29.1
single cells	10,851	97.5	28.4
living cells	10,851	100.0	28.4
GFP+	26	0.2	0.1

Experiment Name: Johannes_Notho kidney
Specimen Name: 20230112_male
Tube Name: kidney young 8

Population	#Events	%Parent	%Total	GFP-A Mean	GFP-A %CV
GFP+	26	0.2396	0.0680	20,484	111.3
living cells	10,851	100.0000	28.3590	682	288.9

BD FACSDiva 8.0

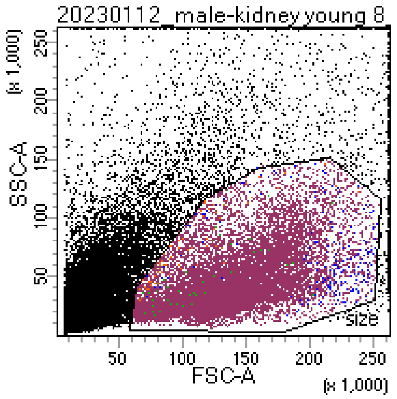

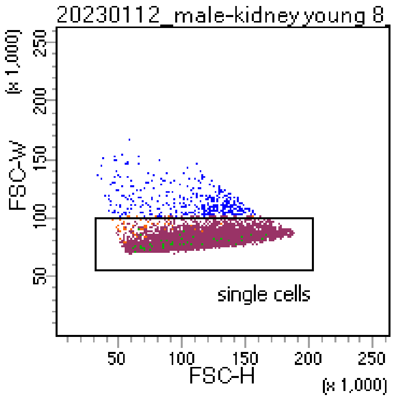

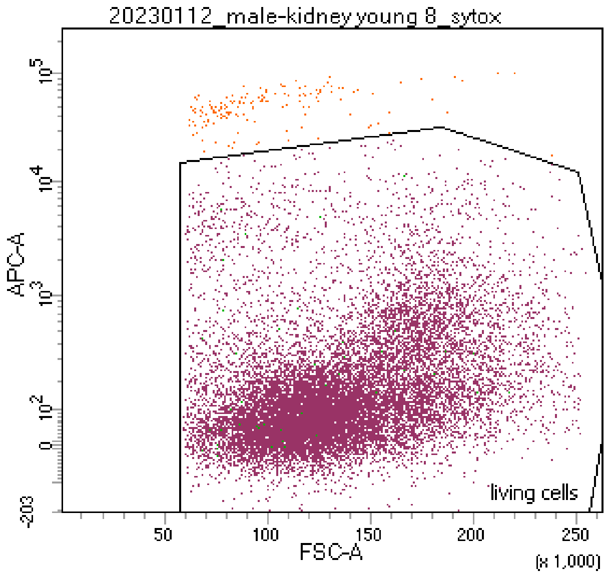

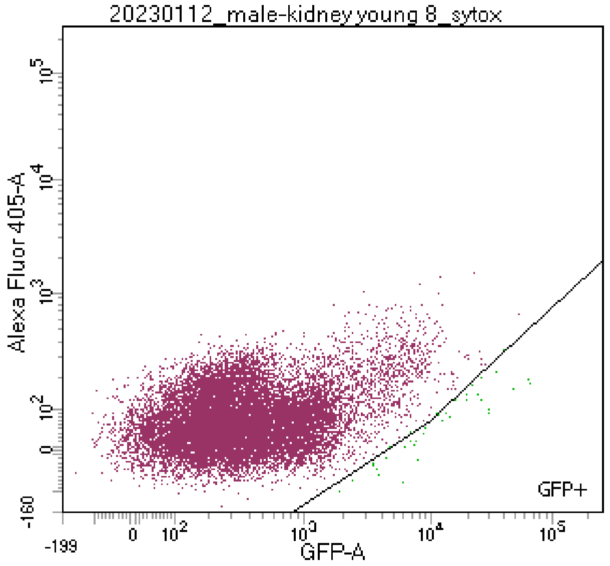

Tube: kidney young 8_sytox

Population	#Events	%Parent	%Total
All Events	58,074	####	100.0
size	16,650	28.7	28.7
single cells	16,247	97.6	28.0
living cells	16,097	99.1	27.7
GFP+	42	0.3	0.1

Experiment Name: Johannes_Notho kidney
Specimen Name: 20230112_male
Tube Name: kidney young 8_sytox

Population	#Events	%Parent	%Total	GFP-A Mean	GFP-A %CV
GFP+	42	0.2609	0.0723	16,552	95.3
living cells	16,097	99.0768	27.7181	707	248.9
