# Supplementary material for: Generation of a transparent killifish line through multiplex CRISPR/Cas9mediated gene inactivation
Source: eLife. 2023 Feb 23;12:e81549. doi: 10.7554/eLife.81549 (PMC10010688; doi:10.7554/eLife.81549)
Supplement: Figure 5—source data 1. [file elife-81549-fig5-data1.zip › Figure_5_source_data/Figure_5_panel_I_source_data/FACS_raw_data/Female_kidney_p21ki-Batch_Analysis_11012023180021.pdf]

BD FACSDiva 9.0.1

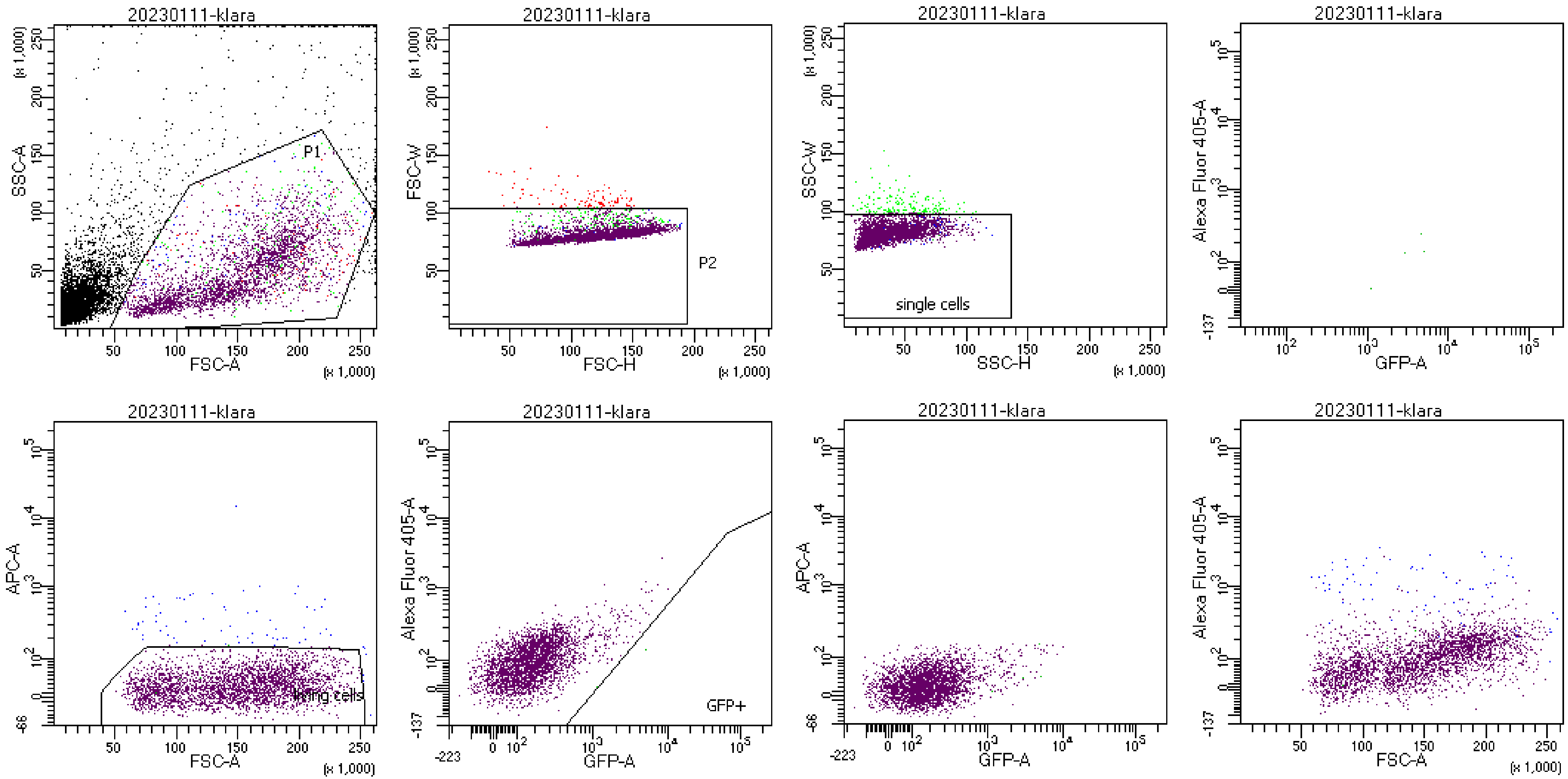

Tube: klara

Population	#Events	%Parent	%Total
All Events	10,000	####	100.0
P1	3,355	33.6	33.6
P2	3,253	97.0	32.5
single cells	3,085	94.8	30.8
living cells	3,014	97.7	30.1
GFP+	4	0.1	0.0

BD FACSDiva 9.0.1

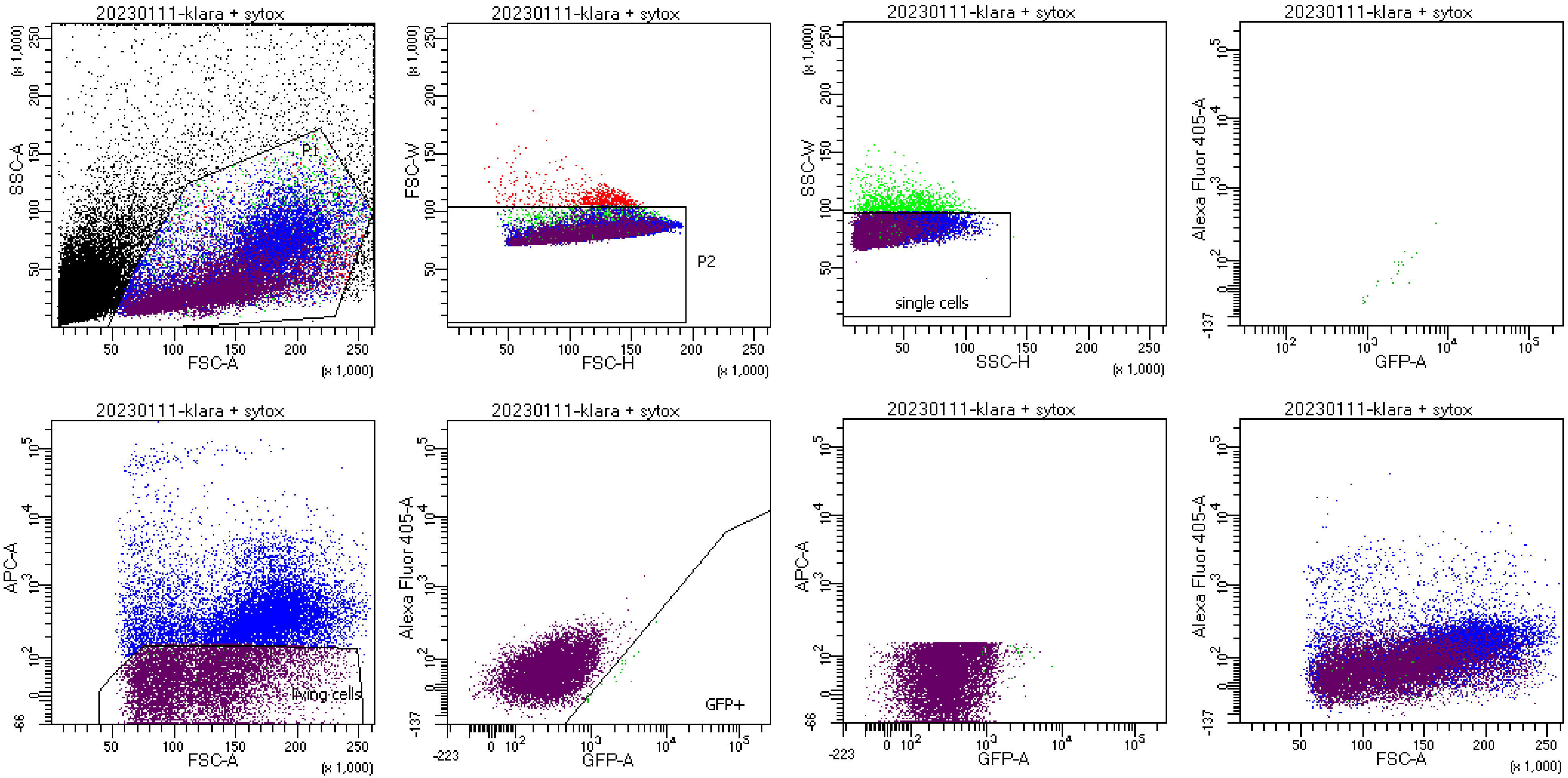

Tube: klara + sytox

Population	#Events	%Parent	%Total
All Events	70,503	####	100.0
P1	23,403	33.2	33.2
P2	22,688	96.9	32.2
single cells	21,503	94.8	30.5
living cells	9,999	46.5	14.2
GFP+	22	0.2	0.0

BD FACSDiva 9.0.1

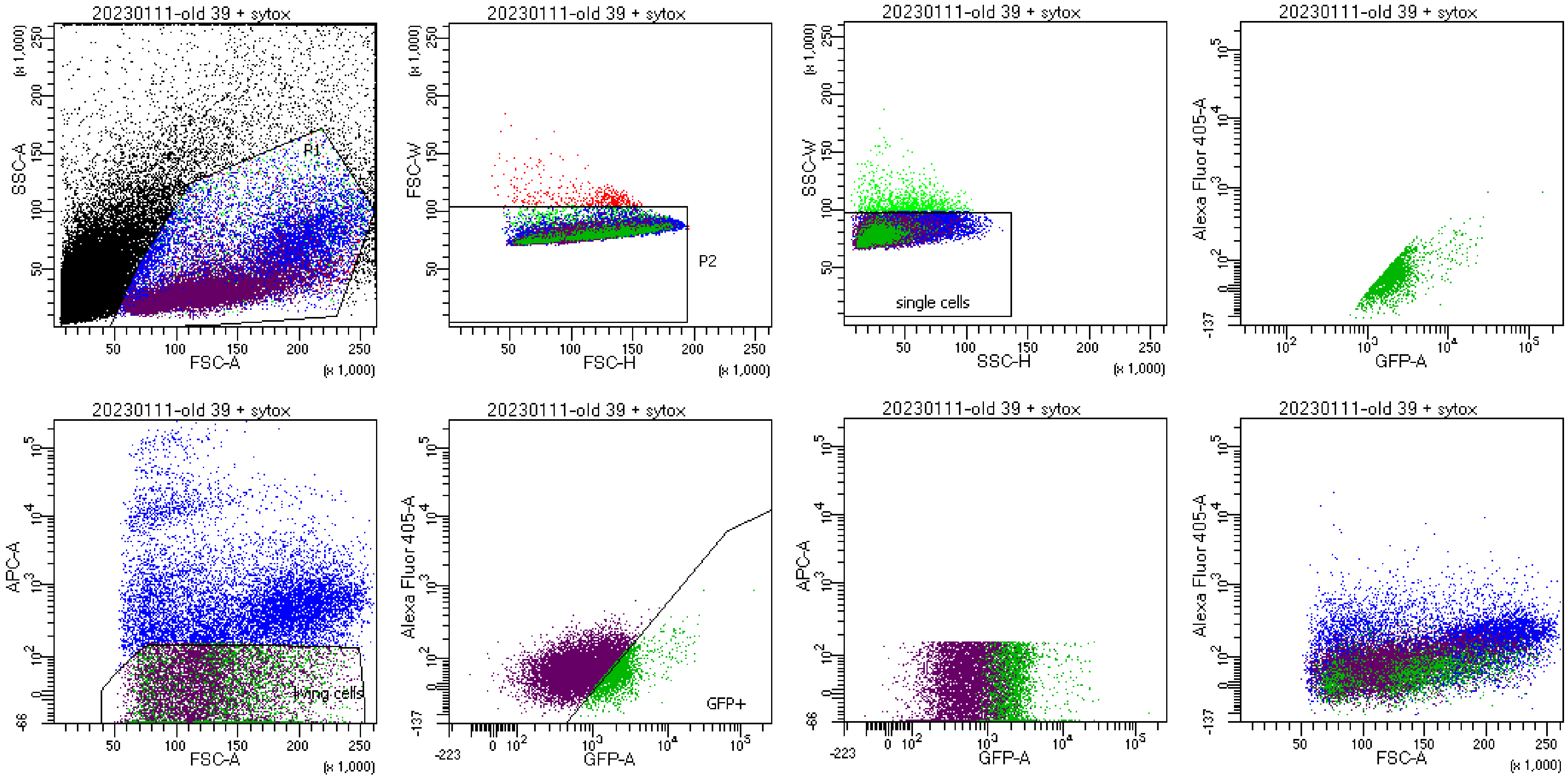

Tube: old 39 + sytox

Population	#Events	%Parent	%Total
All Events	112,169	####	100.0
P1	21,406	19.1	19.1
P2	20,955	97.9	18.7
single cells	19,909	95.0	17.7
living cells	9,986	50.2	8.9
GFP+	1,908	19.1	1.7

BD FACSDiva 9.0.1

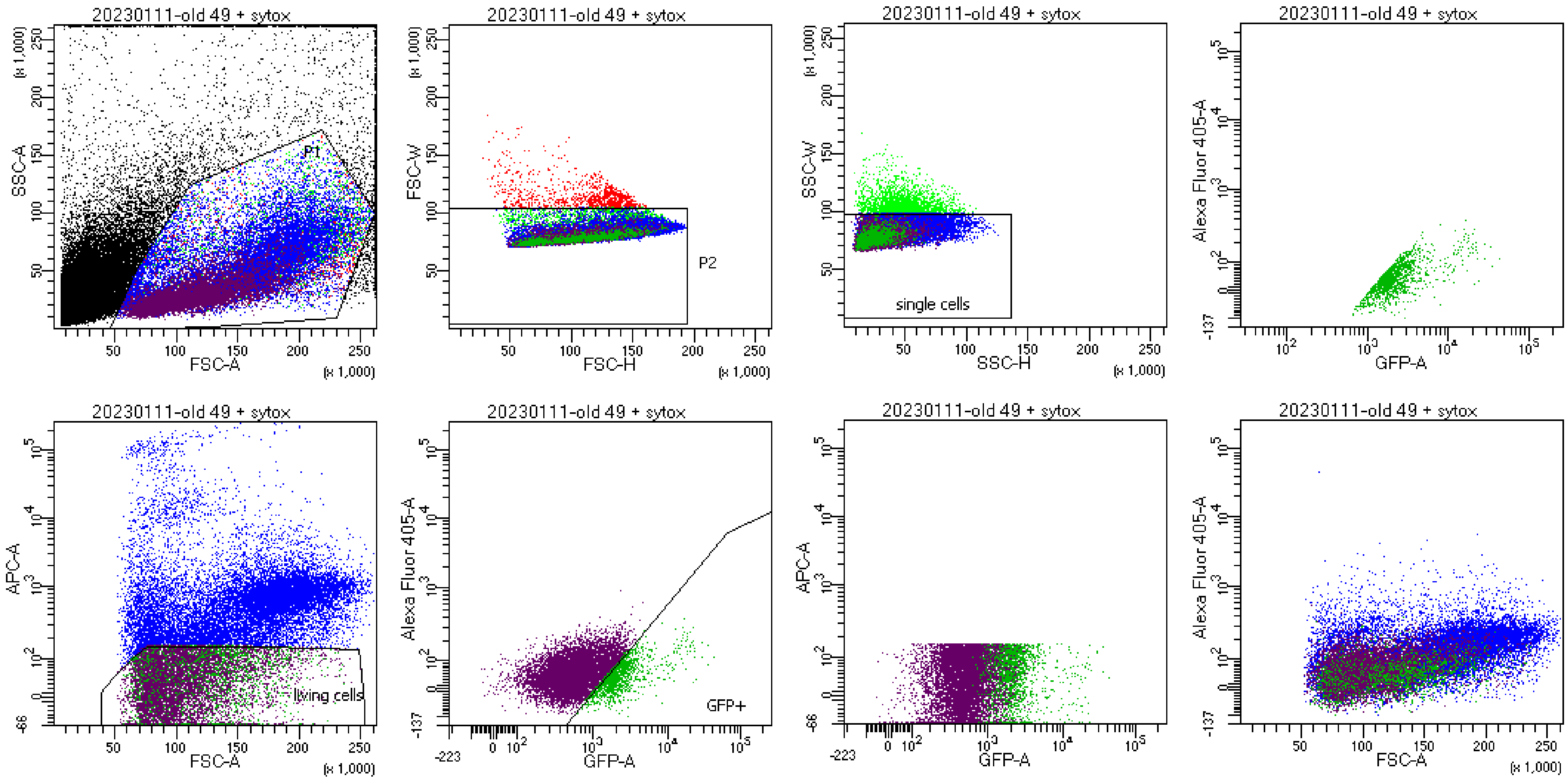

Tube: old 49 + sytox

Population	#Events	%Parent	%Total
All Events	95,664	####	100.0
P1	27,525	28.8	28.8
P2	26,554	96.5	27.8
single cells	24,544	92.4	25.7
living cells	9,994	40.7	10.4
GFP+	1,285	12.9	1.3

BD FACSDiva 9.0.1

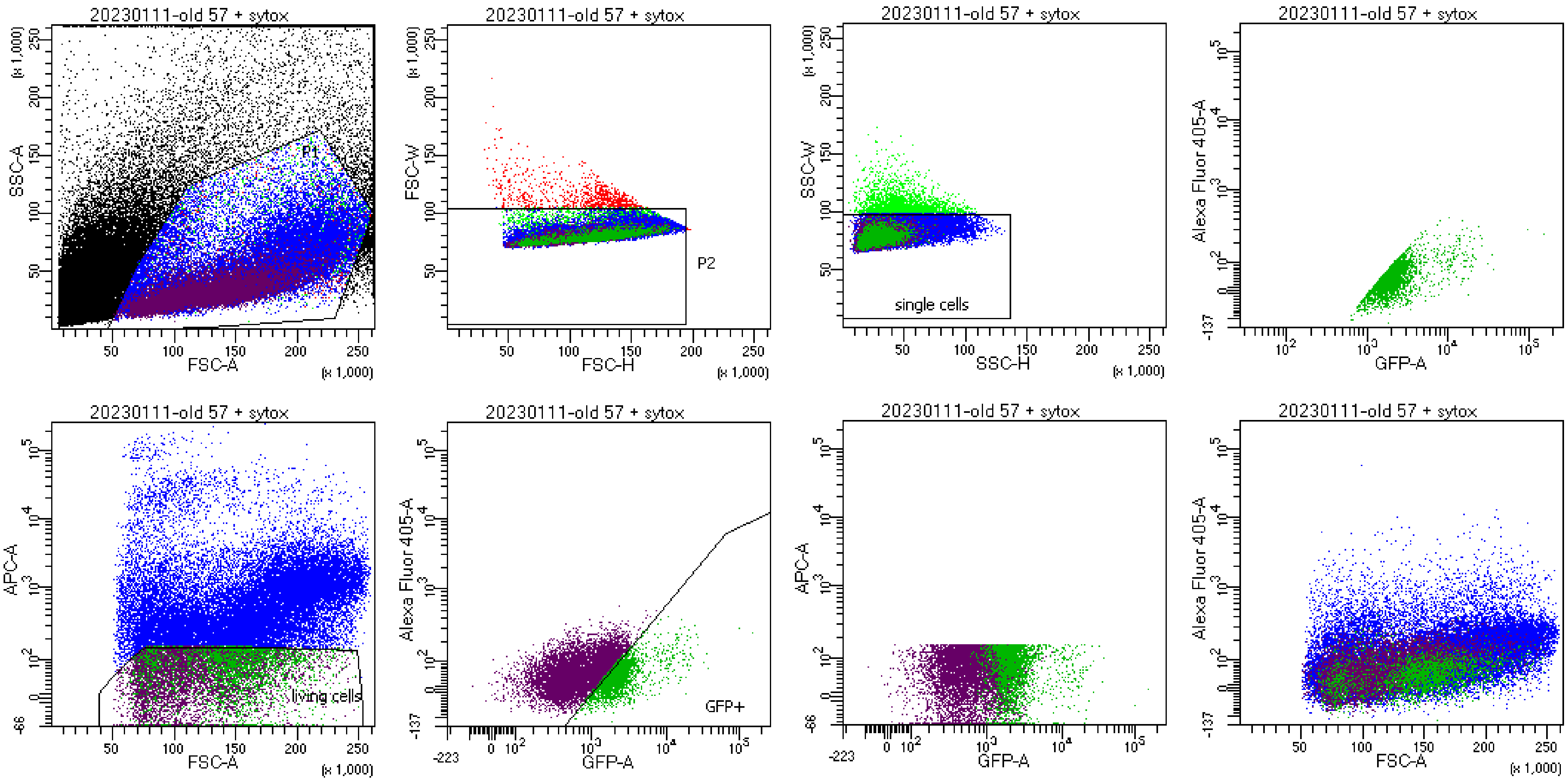

Tube: old 57 + sytox

Population	#Events	%Parent	%Total
All Events	156,800	####	100.0
P1	38,013	24.2	24.2
P2	37,134	97.7	23.7
single cells	34,882	93.9	22.2
living cells	9,987	28.6	6.4
GFP+	2,441	24.4	1.6

BD FACSDiva 9.0.1

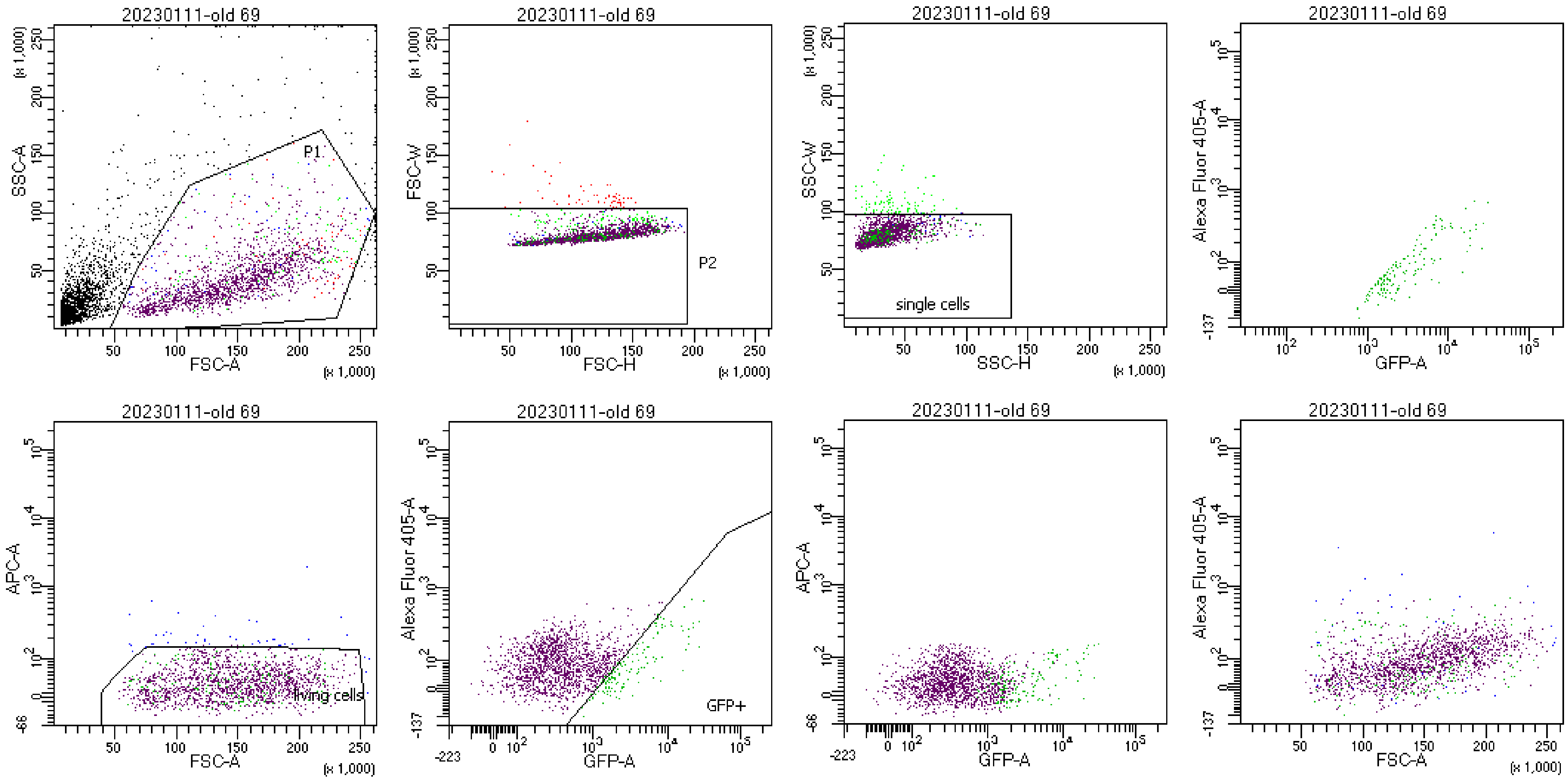

Tube: old 69

Population	#Events	%Parent	%Total
All Events	4,108	####	100.0
P1	1,764	42.9	42.9
P2	1,704	96.6	41.5
single cells	1,610	94.5	39.2
living cells	1,565	97.2	38.1
GFP+	141	9.0	3.4

BD FACSDiva 9.0.1

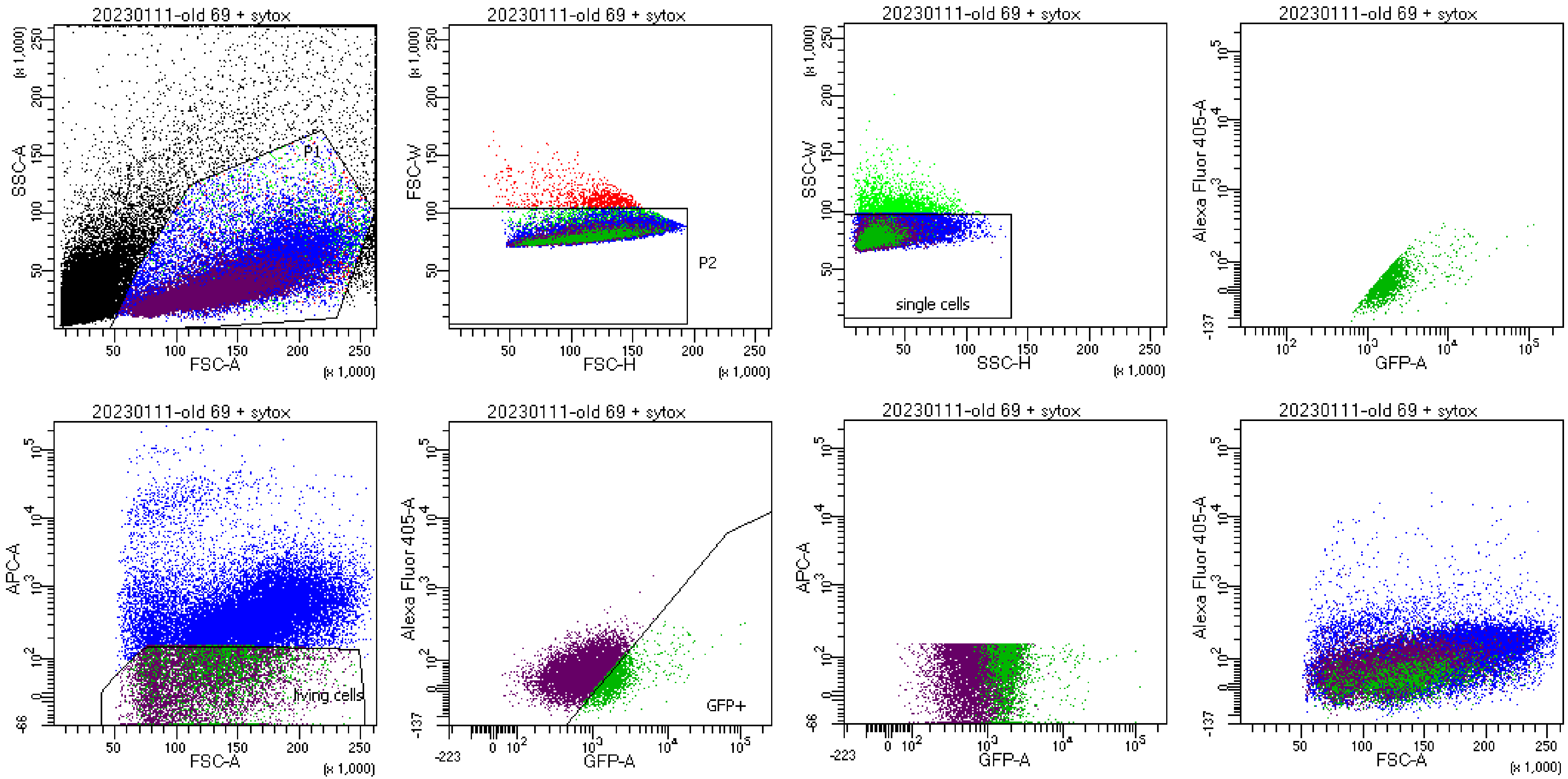

Tube: old 69 + sytox

Population	#Events	%Parent	%Total
All Events	86,908	####	100.0
P1	34,921	40.2	40.2
P2	33,970	97.3	39.1
single cells	31,848	93.8	36.6
living cells	9,986	31.4	11.5
GFP+	1,724	17.3	2.0

BD FACSDiva 9.0.1

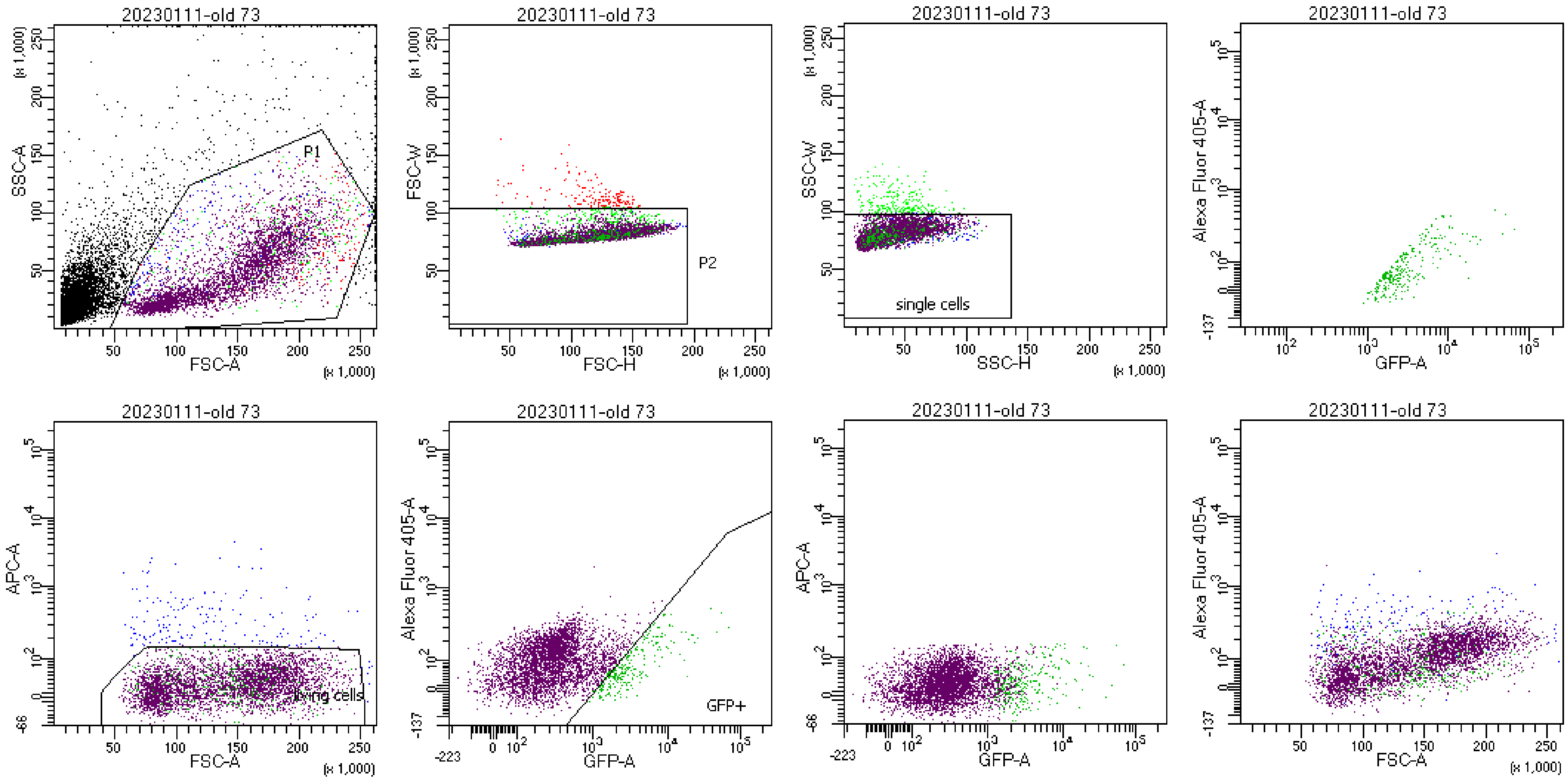

Tube: old 73

Population	#Events	%Parent	%Total
All Events	11,336	####	100.0
P1	4,540	40.0	40.0
P2	4,365	96.1	38.5
single cells	4,123	94.5	36.4
living cells	3,950	95.8	34.8
GFP+	266	6.7	2.3

BD FACSDiva 9.0.1

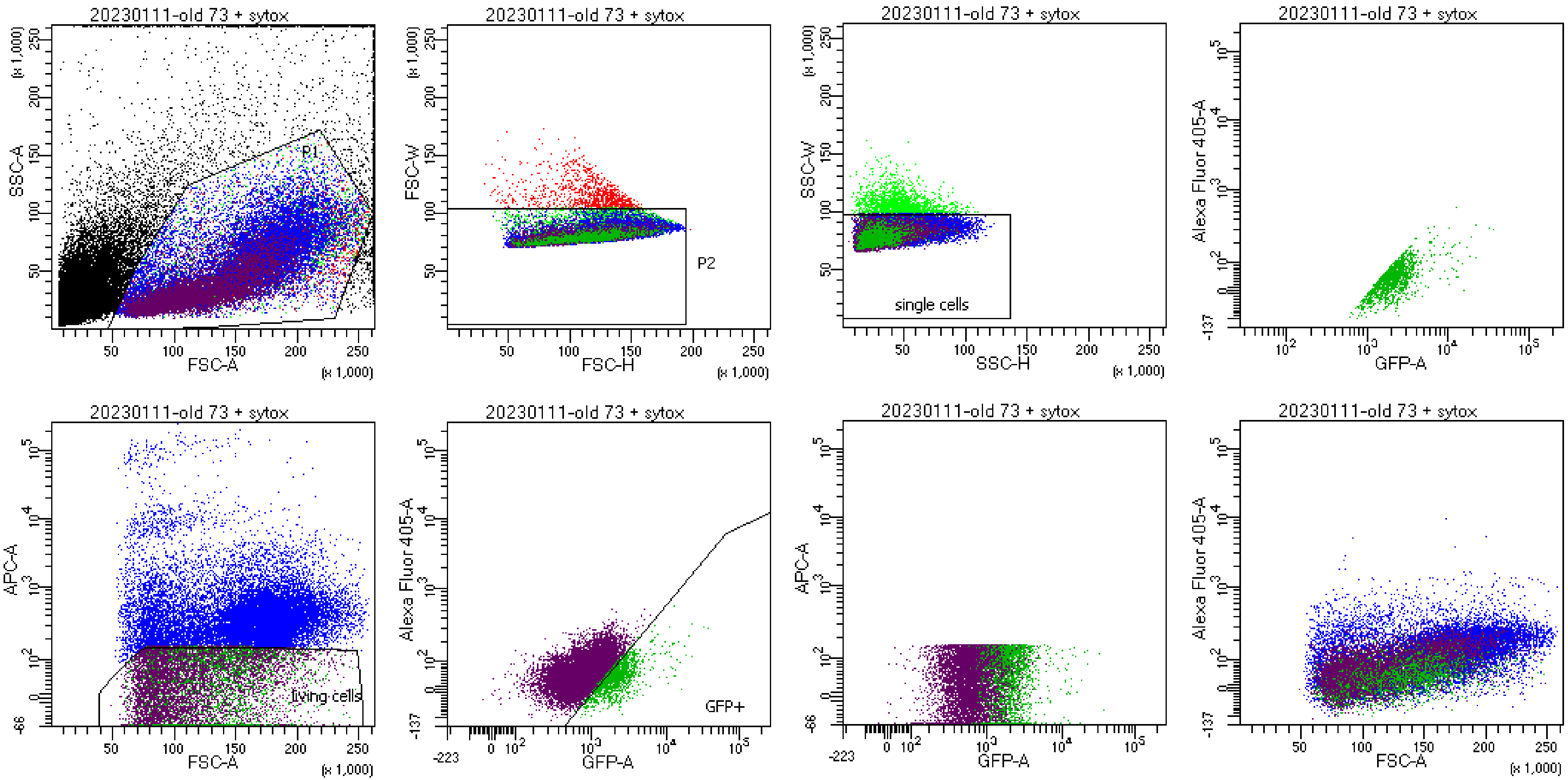

Tube: old 73 + sytox

Population	#Events	%Parent	%Total
All Events	69,285	####	100.0
P1	28,843	41.6	41.6
P2	27,808	96.4	40.1
single cells	26,109	93.9	37.7
living cells	9,975	38.2	14.4
GFP+	1,438	14.4	2.1

BD FACSDiva 9.0.1

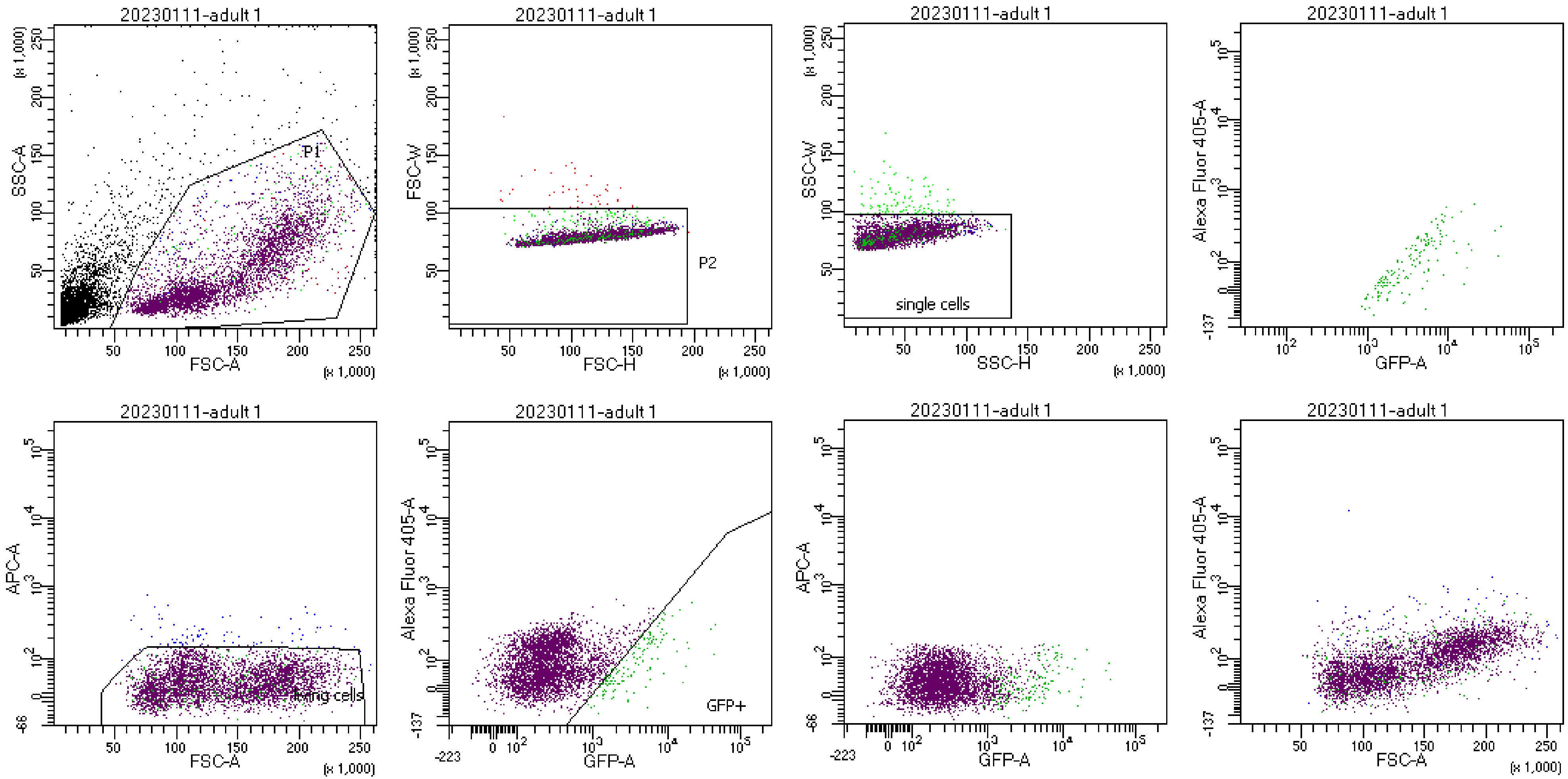

Tube: adult 1

Population	#Events	%Parent	%Total
All Events	8,320	####	100.0
P1	4,274	51.4	51.4
P2	4,231	99.0	50.9
single cells	4,127	97.5	49.6
living cells	4,041	97.9	48.6
GFP+	151	3.7	1.8

BD FACSDiva 9.0.1

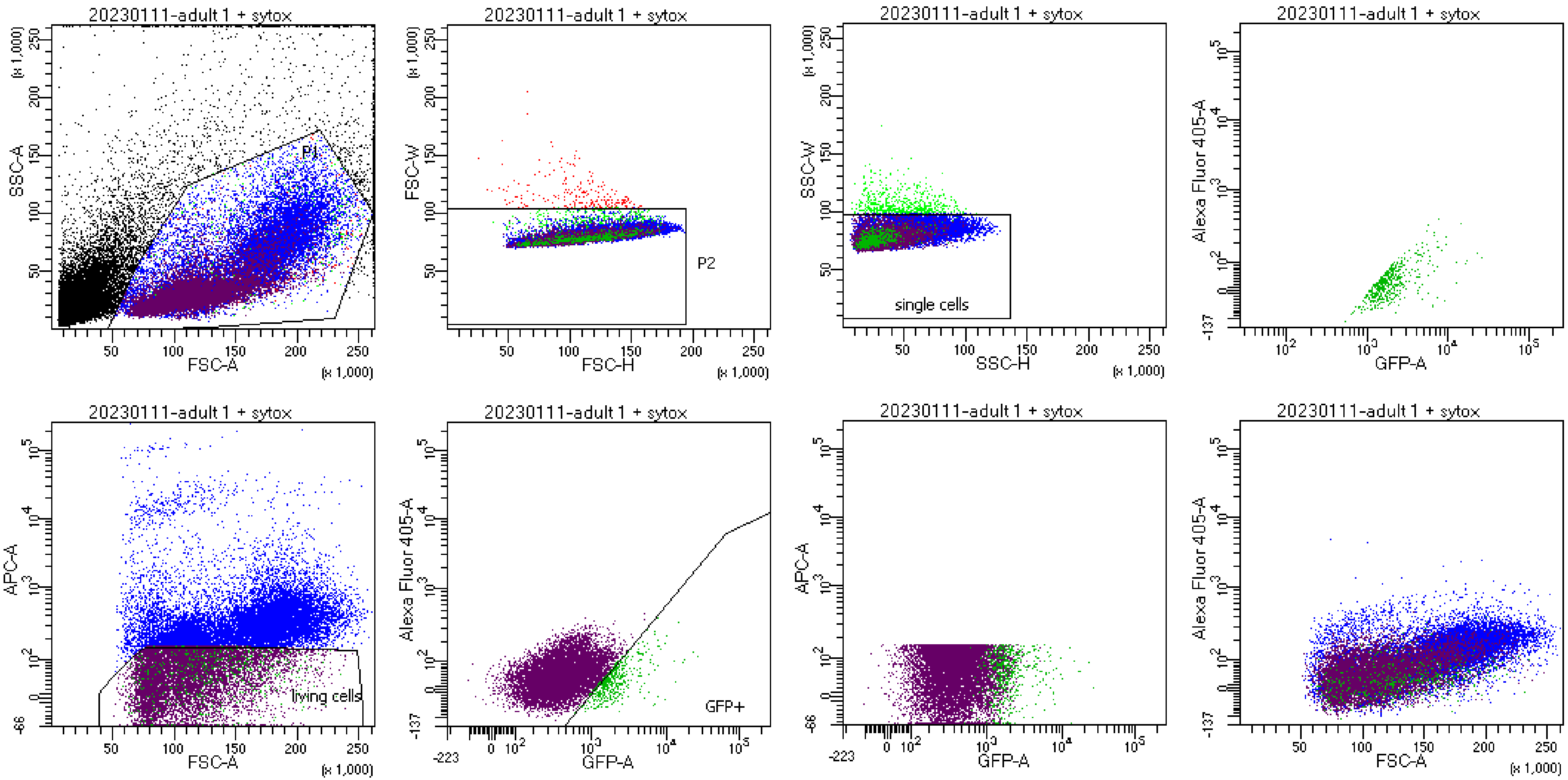

Tube: adult 1 + sytox

Population	#Events	%Parent	%Total
All Events	48,620	####	100.0
P1	24,154	49.7	49.7
P2	23,949	99.2	49.3
single cells	23,400	97.7	48.1
living cells	10,012	42.8	20.6
GFP+	498	5.0	1.0

BD FACSDiva 9.0.1

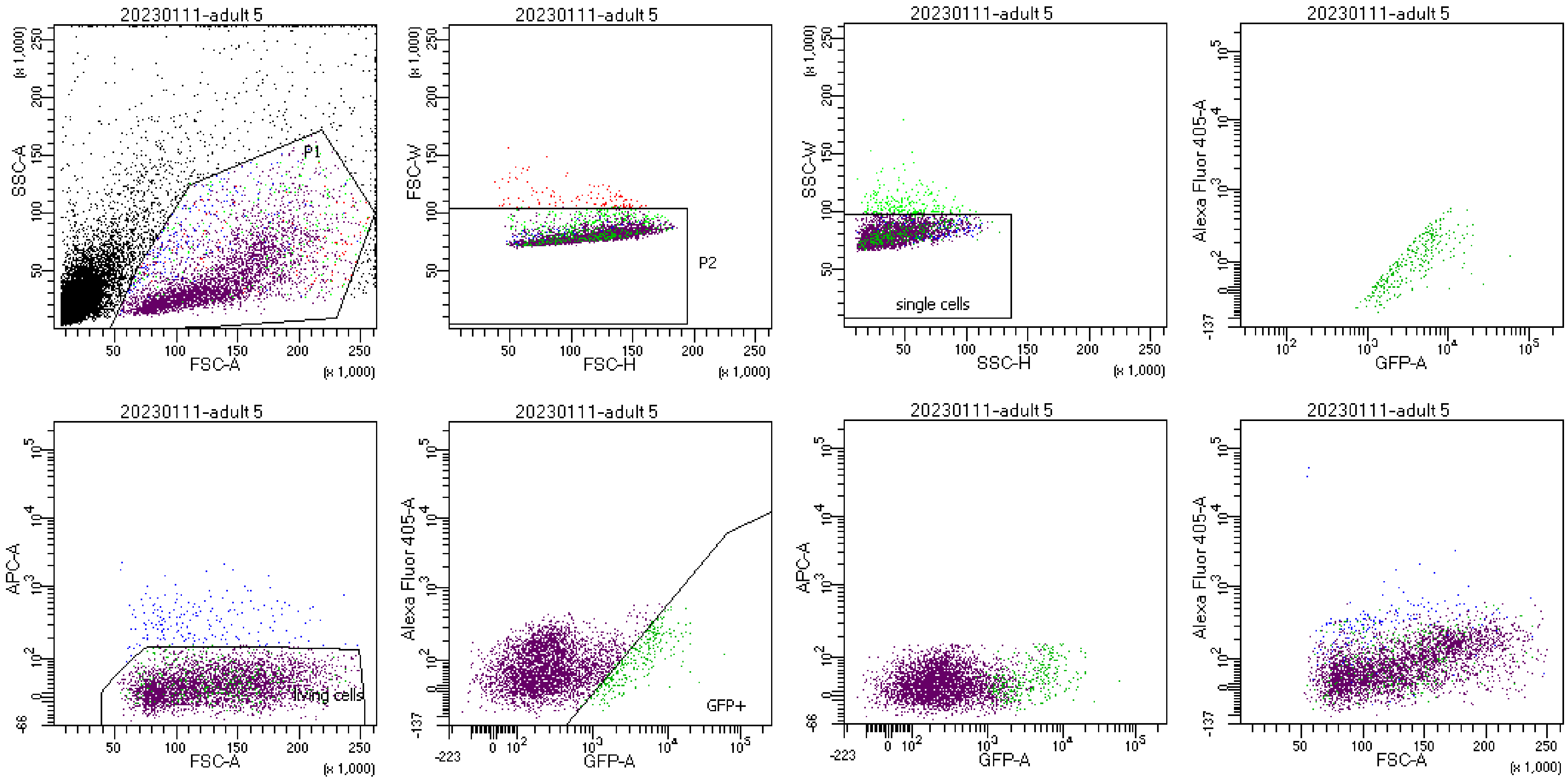

Tube: adult 5

Population	#Events	%Parent	%Total
All Events	15,548	####	100.0
P1	4,490	28.9	28.9
P2	4,374	97.4	28.1
single cells	4,145	94.8	26.7
living cells	3,943	95.1	25.4
GFP+	298	7.6	1.9

BD FACSDiva 9.0.1

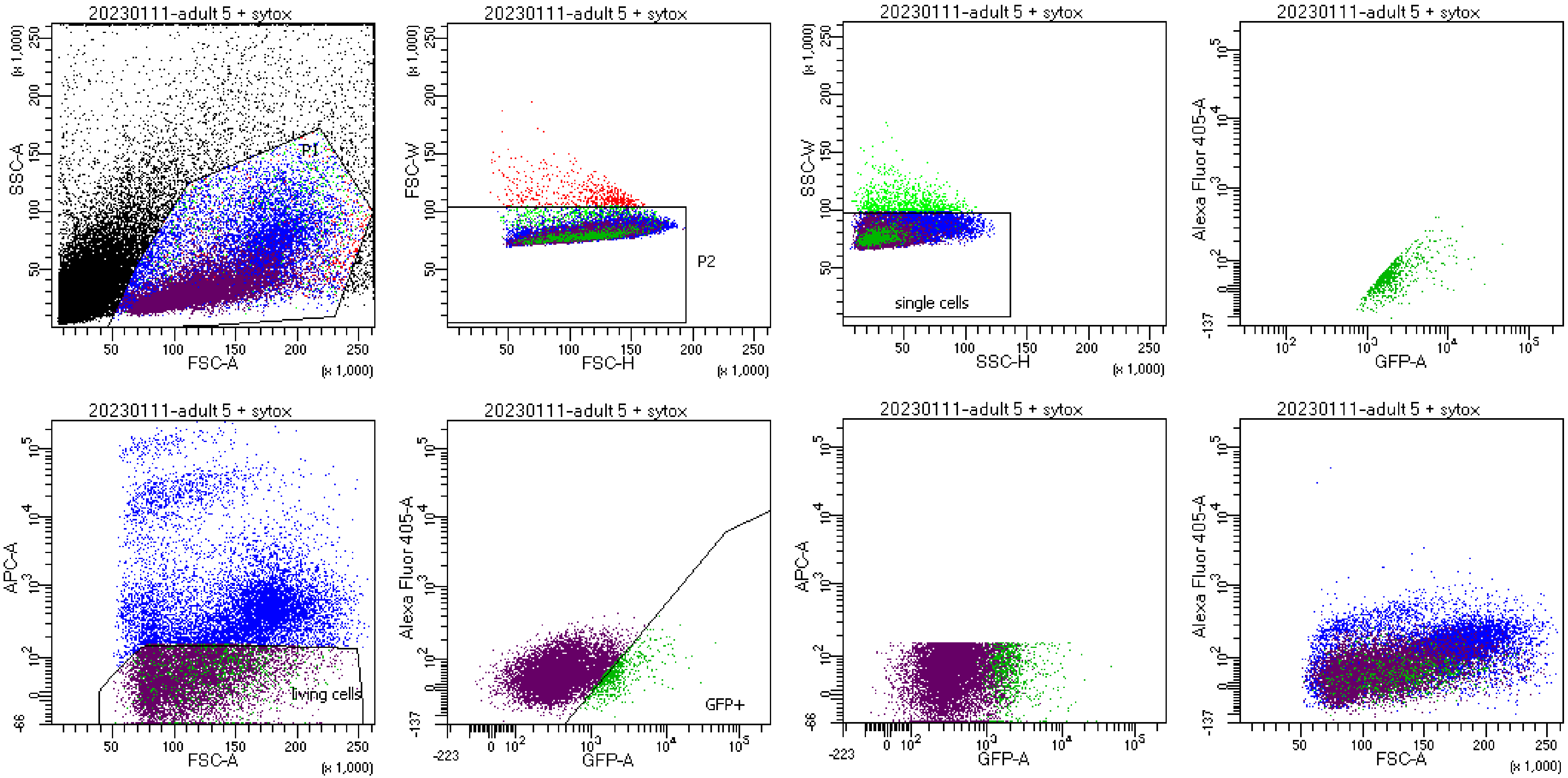

Tube: adult 5 + sytox

Population	#Events	%Parent	%Total
All Events	74,856	####	100.0
P1	21,069	28.1	28.1
P2	20,514	97.4	27.4
single cells	19,550	95.3	26.1
living cells	9,988	51.1	13.3
GFP+	695	7.0	0.9

BD FACSDiva 9.0.1

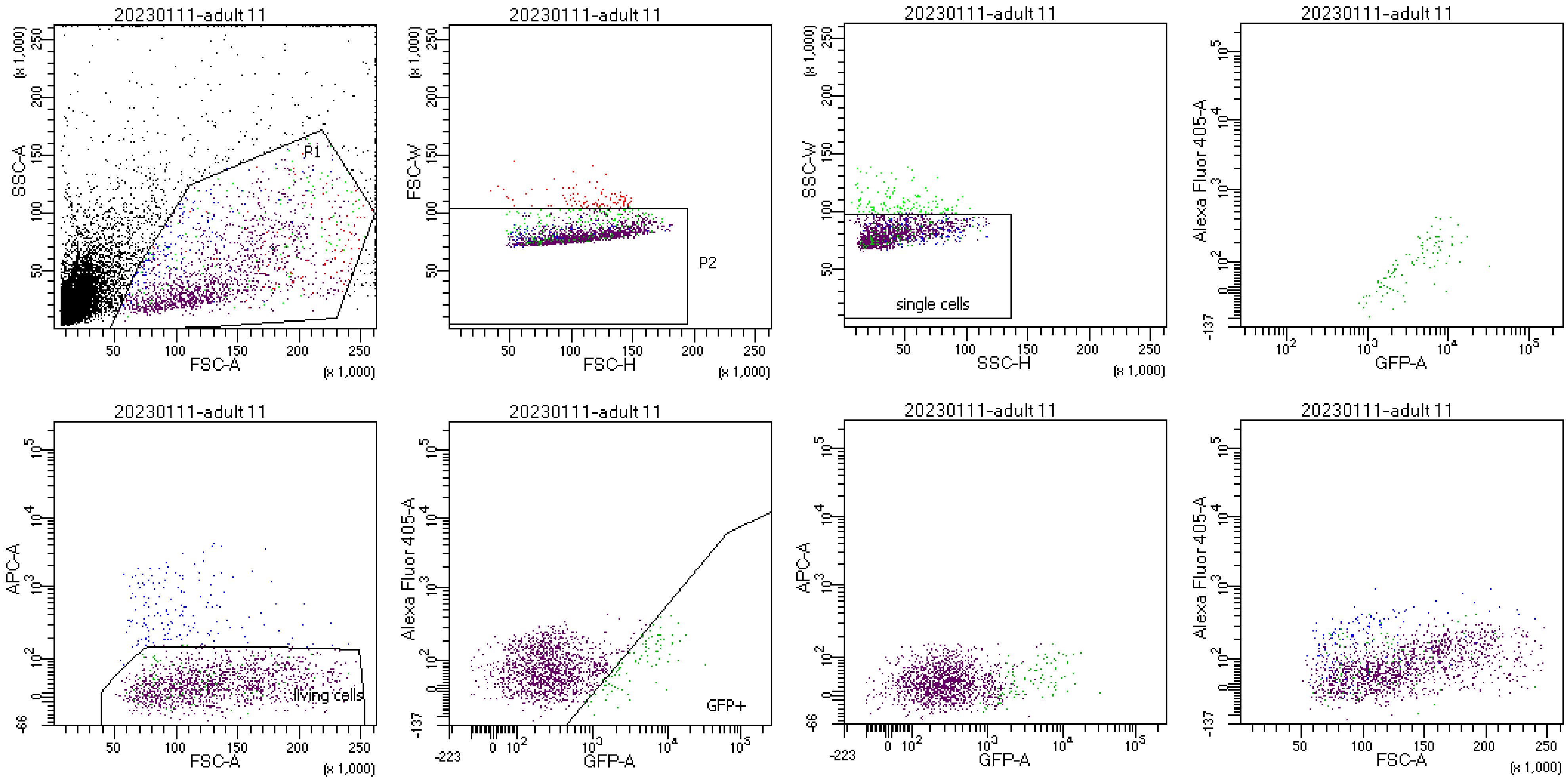

Tube: adult 11

Population	#Events	%Parent	%Total
All Events	10,993	####	100.0
P1	1,827	16.6	16.6
P2	1,722	94.3	15.7
single cells	1,588	92.2	14.4
living cells	1,427	89.9	13.0
GFP+	93	6.5	0.8

BD FACSDiva 9.0.1

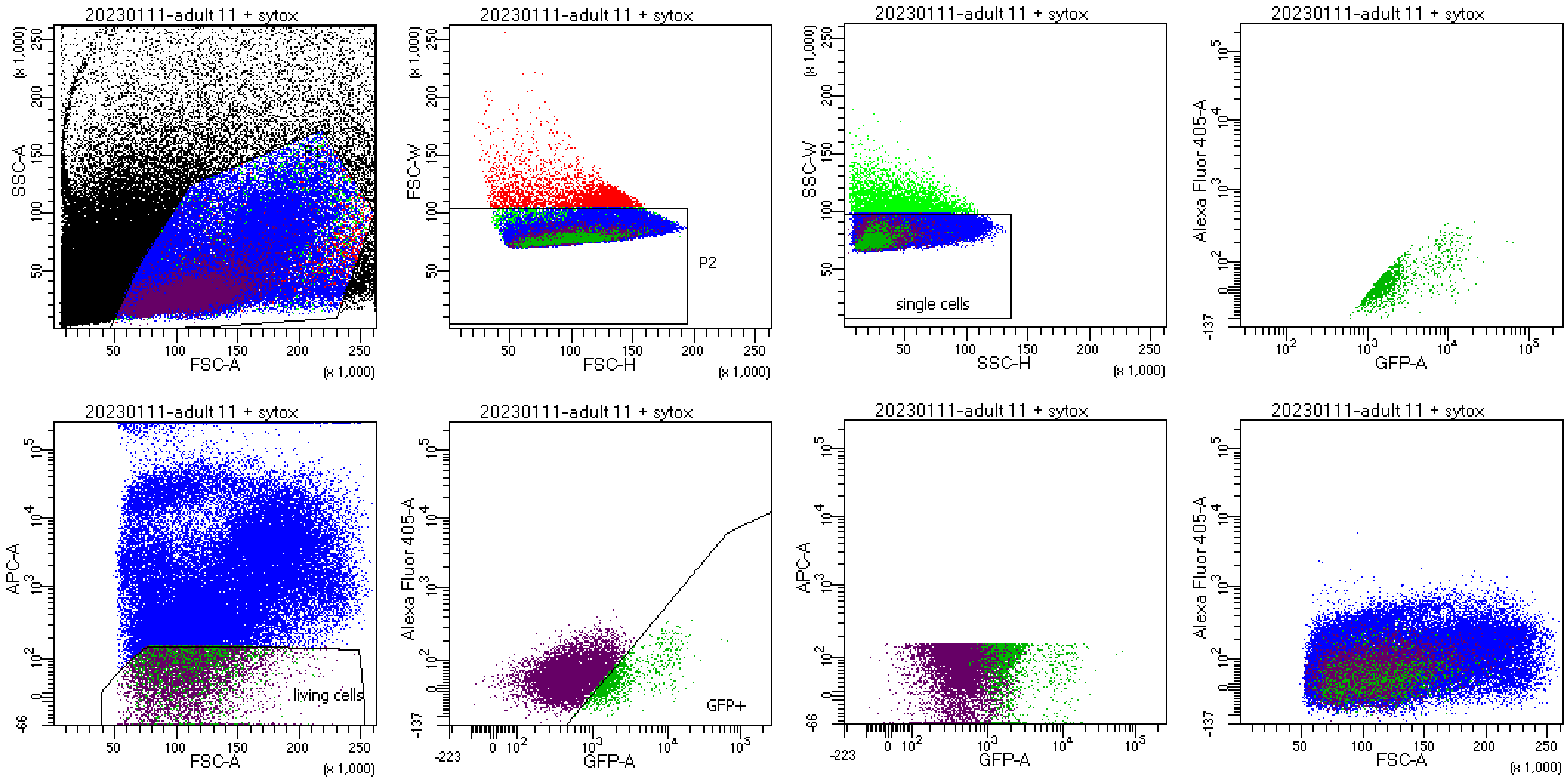

Tube: adult 11 + sytox

Population	#Events	%Parent	%Total
All Events	403,516	####	100.0
P1	70,179	17.4	17.4
P2	66,625	94.9	16.5
single cells	62,108	93.2	15.4
living cells	9,580	15.4	2.4
GFP+	1,143	11.9	0.3

BD FACSDiva 9.0.1

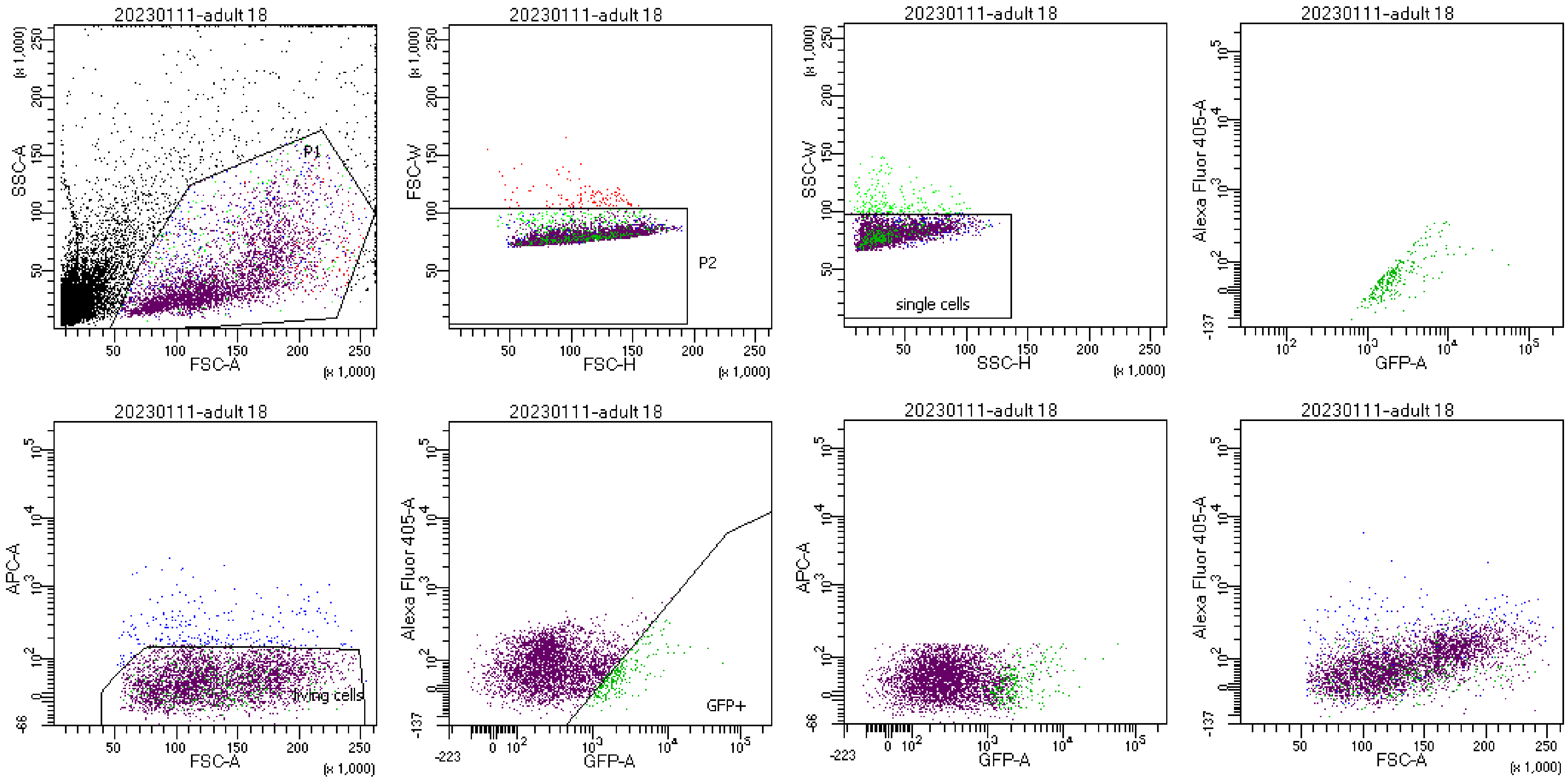

Tube: adult 18

Population	#Events	%Parent	%Total
All Events	14,248	####	100.0
P1	4,411	31.0	31.0
P2	4,294	97.3	30.1
single cells	4,083	95.1	28.7
living cells	3,850	94.3	27.0
GFP+	263	6.8	1.8

BD FACSDiva 9.0.1

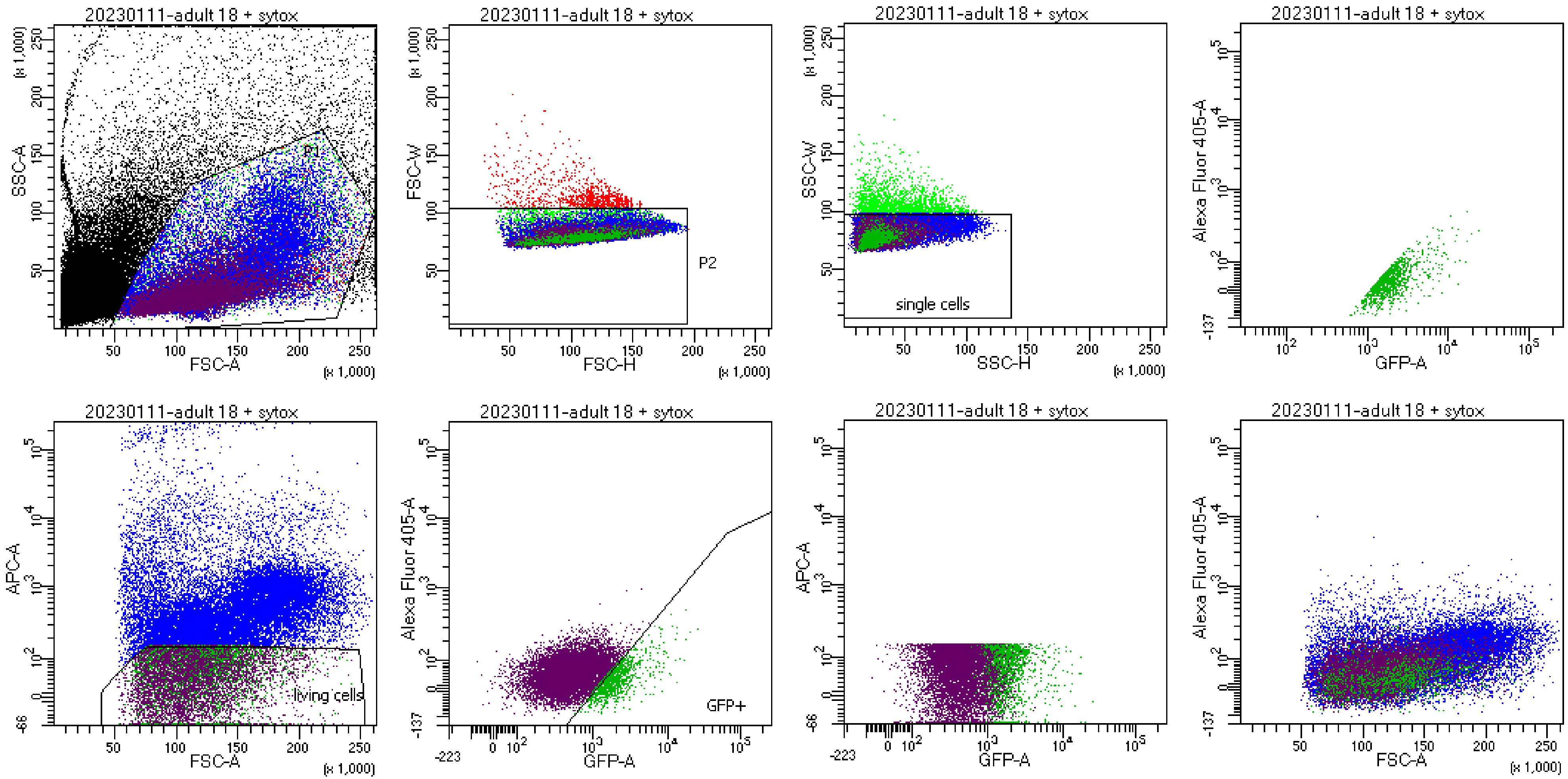

Tube: adult 18 + sytox

Population	#Events	%Parent	%Total
All Events	104,853	####	100.0
P1	32,989	31.5	31.5
P2	32,025	97.1	30.5
single cells	30,076	93.9	28.7
living cells	10,000	33.2	9.5
GFP+	1,144	11.4	1.1

BD FACSDiva 9.0.1

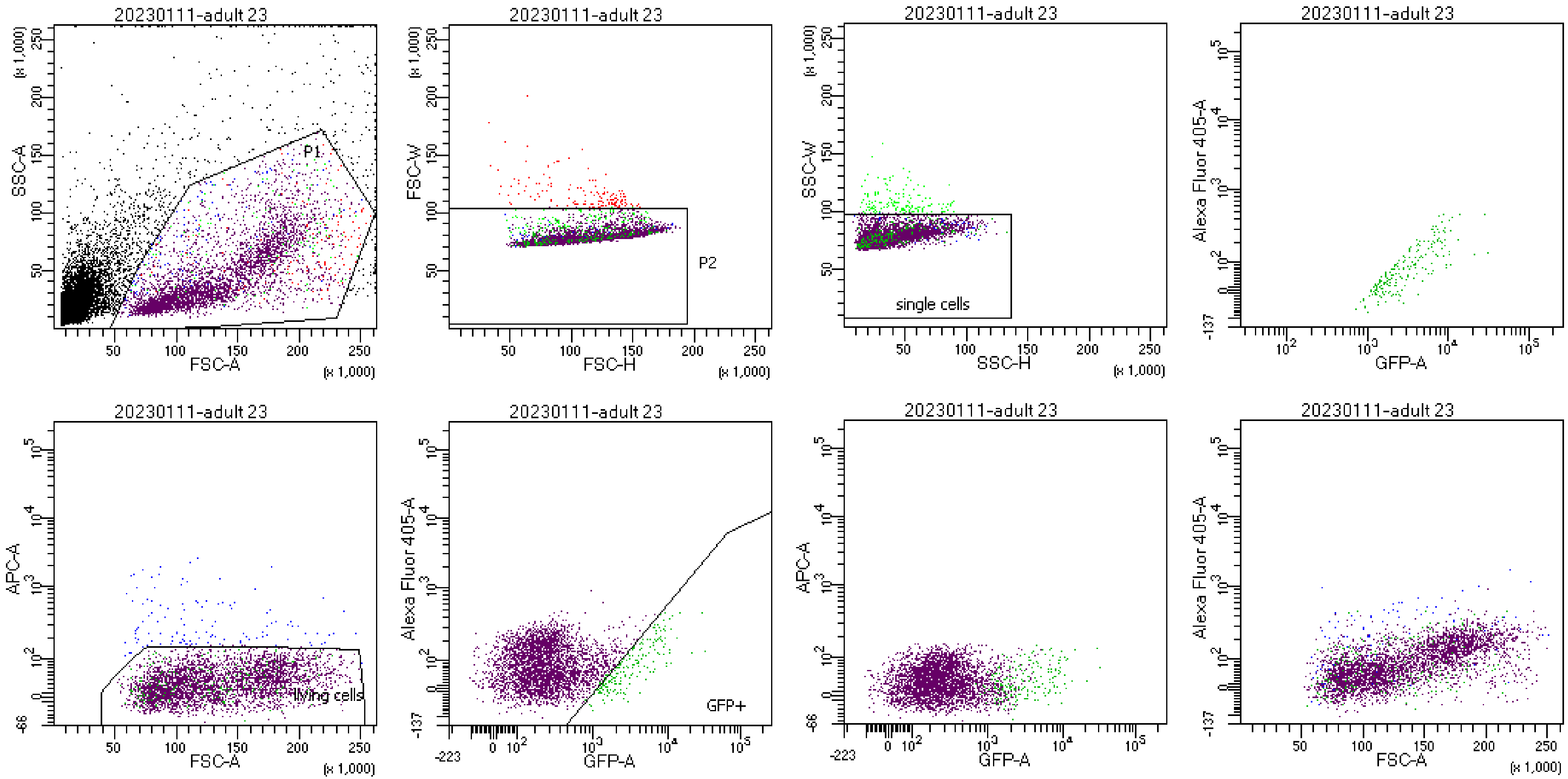

Tube: adult 23

Population	#Events	%Parent	%Total
All Events	12,064	####	100.0
P1	4,118	34.1	34.1
P2	3,969	96.4	32.9
single cells	3,813	96.1	31.6
living cells	3,694	96.9	30.6
GFP+	195	5.3	1.6

BD FACSDiva 9.0.1

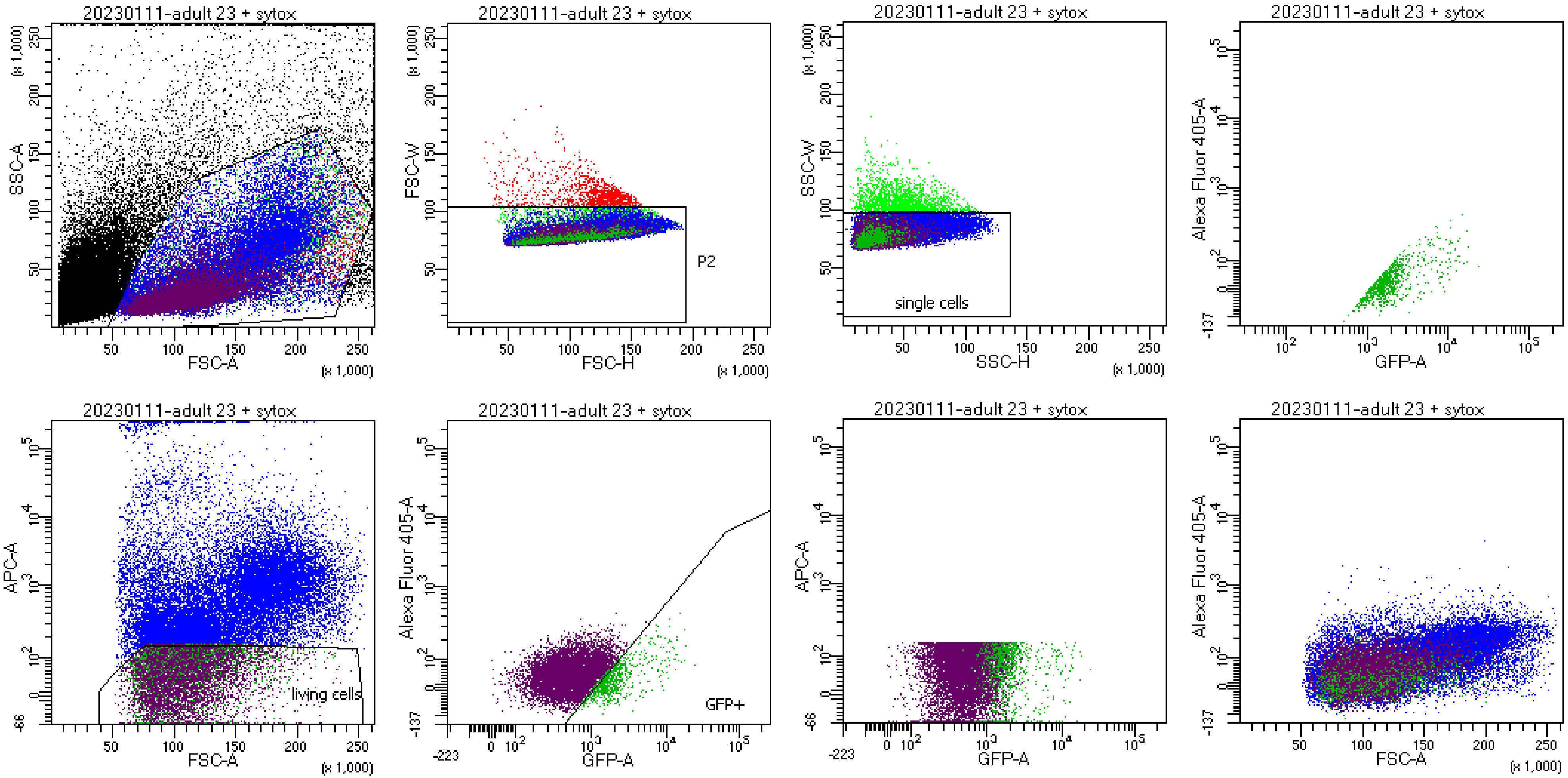

Tube: adult 23 + sytox

Population	#Events	%Parent	%Total
All Events	106,760	####	100.0
P1	33,713	31.6	31.6
P2	32,502	96.4	30.4
single cells	30,571	94.1	28.6
living cells	10,000	32.7	9.4
GFP+	834	8.3	0.8

BD FACSDiva 9.0.1

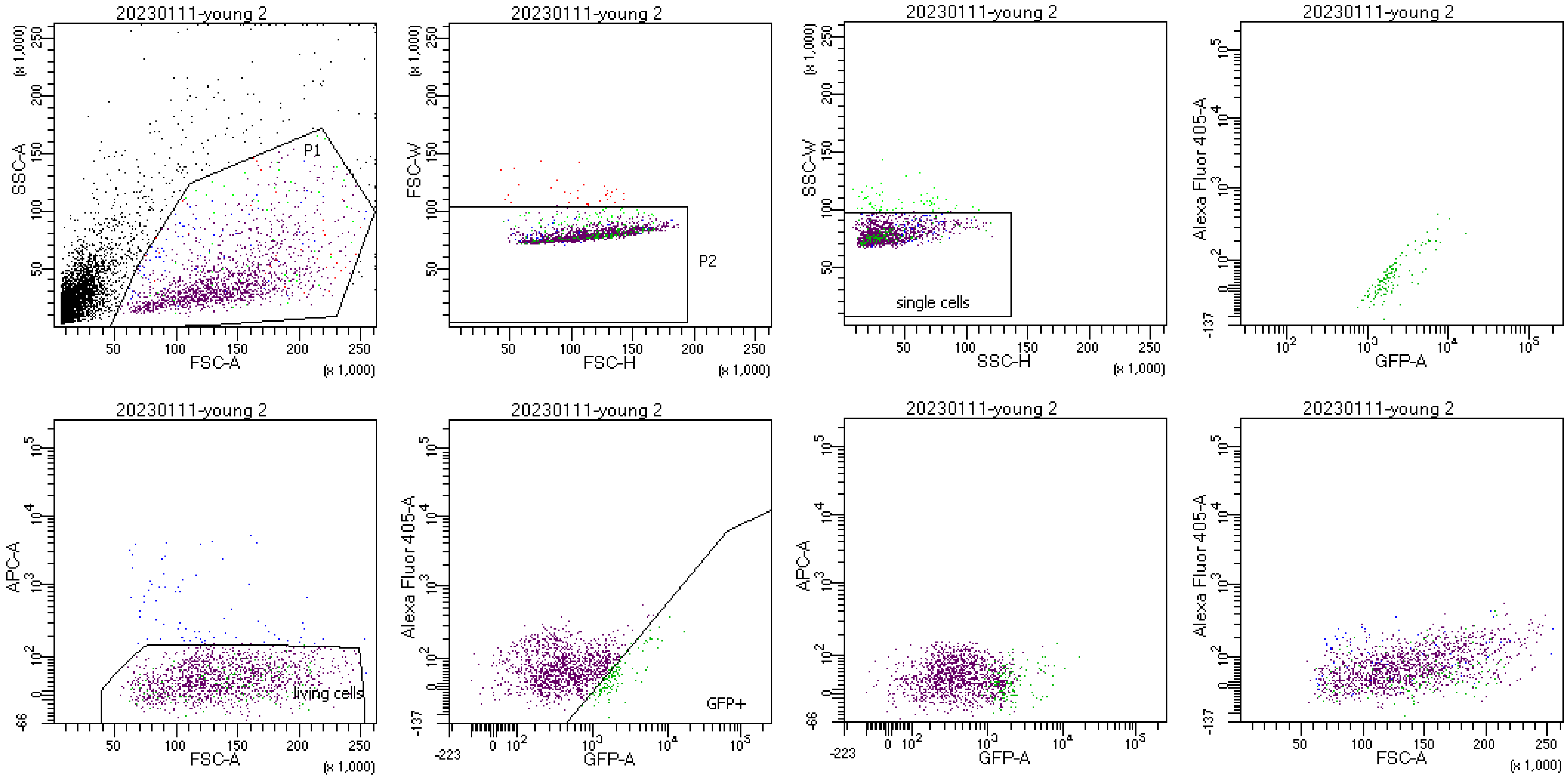

Tube: young 2

Population	#Events	%Parent	%Total
All Events	5,158	####	100.0
P1	1,578	30.6	30.6
P2	1,545	97.9	30.0
single cells	1,485	96.1	28.8
living cells	1,415	95.3	27.4
GFP+	126	8.9	2.4

BD FACSDiva 9.0.1

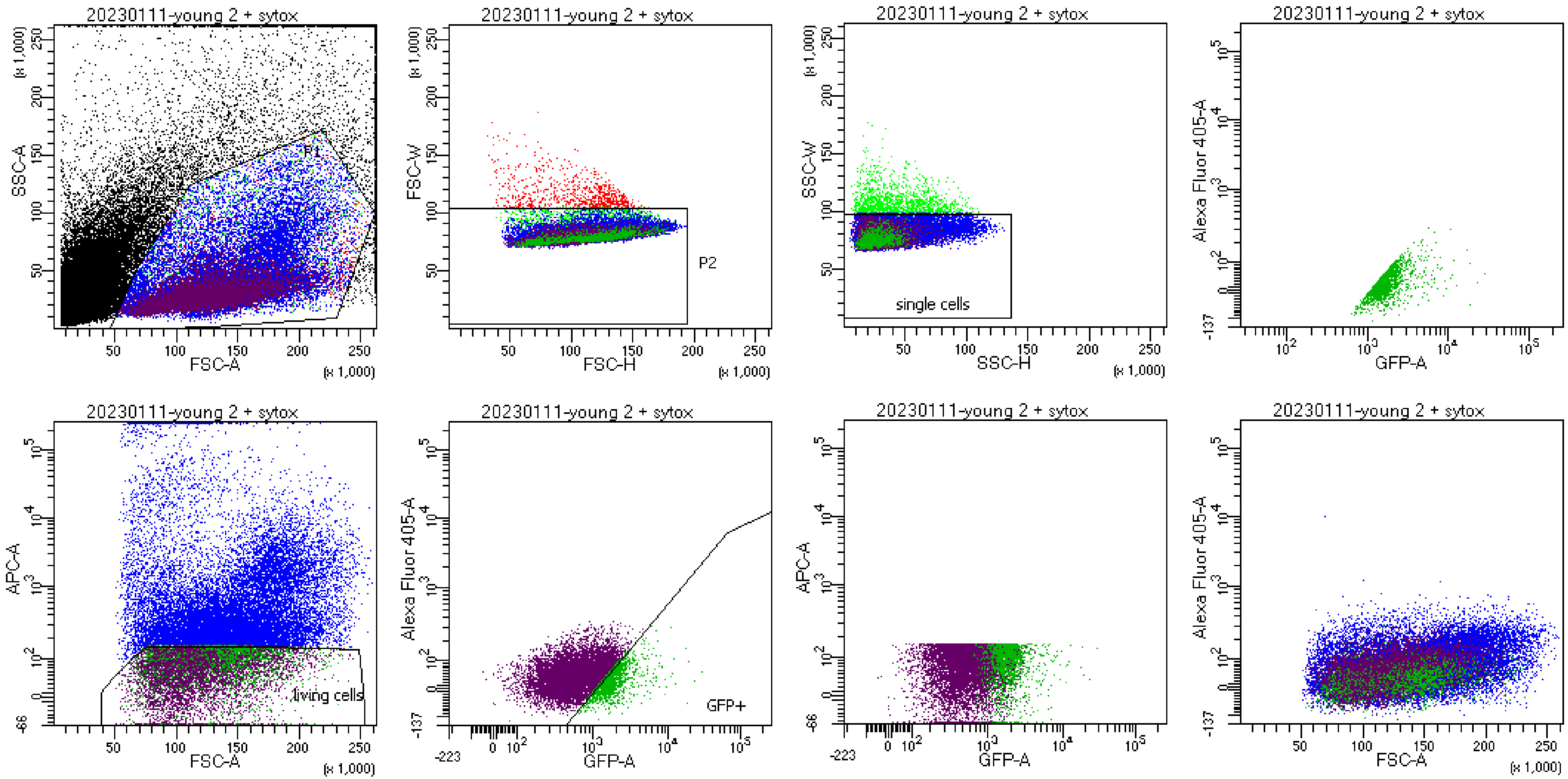

Tube: young 2 + sytox

Population	#Events	%Parent	%Total
All Events	107,451	####	100.0
P1	30,878	28.7	28.7
P2	30,152	97.6	28.1
single cells	28,817	95.6	26.8
living cells	10,000	34.7	9.3
GFP+	1,343	13.4	1.2

BD FACSDiva 9.0.1

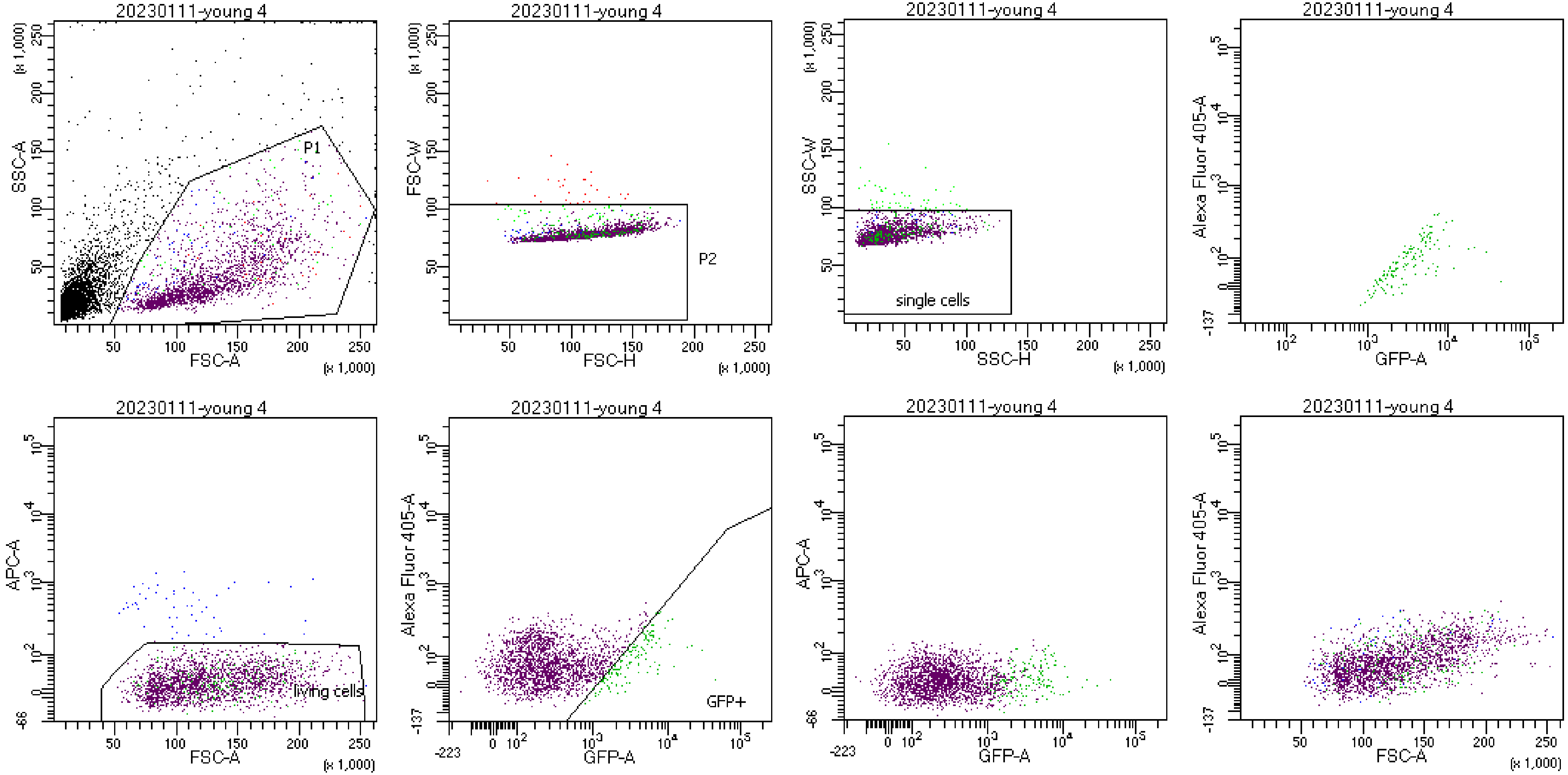

Tube: young 4

Population	#Events	%Parent	%Total
All Events	5,980	####	100.0
P1	2,256	37.7	37.7
P2	2,227	98.7	37.2
single cells	2,176	97.7	36.4
living cells	2,124	97.6	35.5
GFP+	142	6.7	2.4

BD FACSDiva 9.0.1

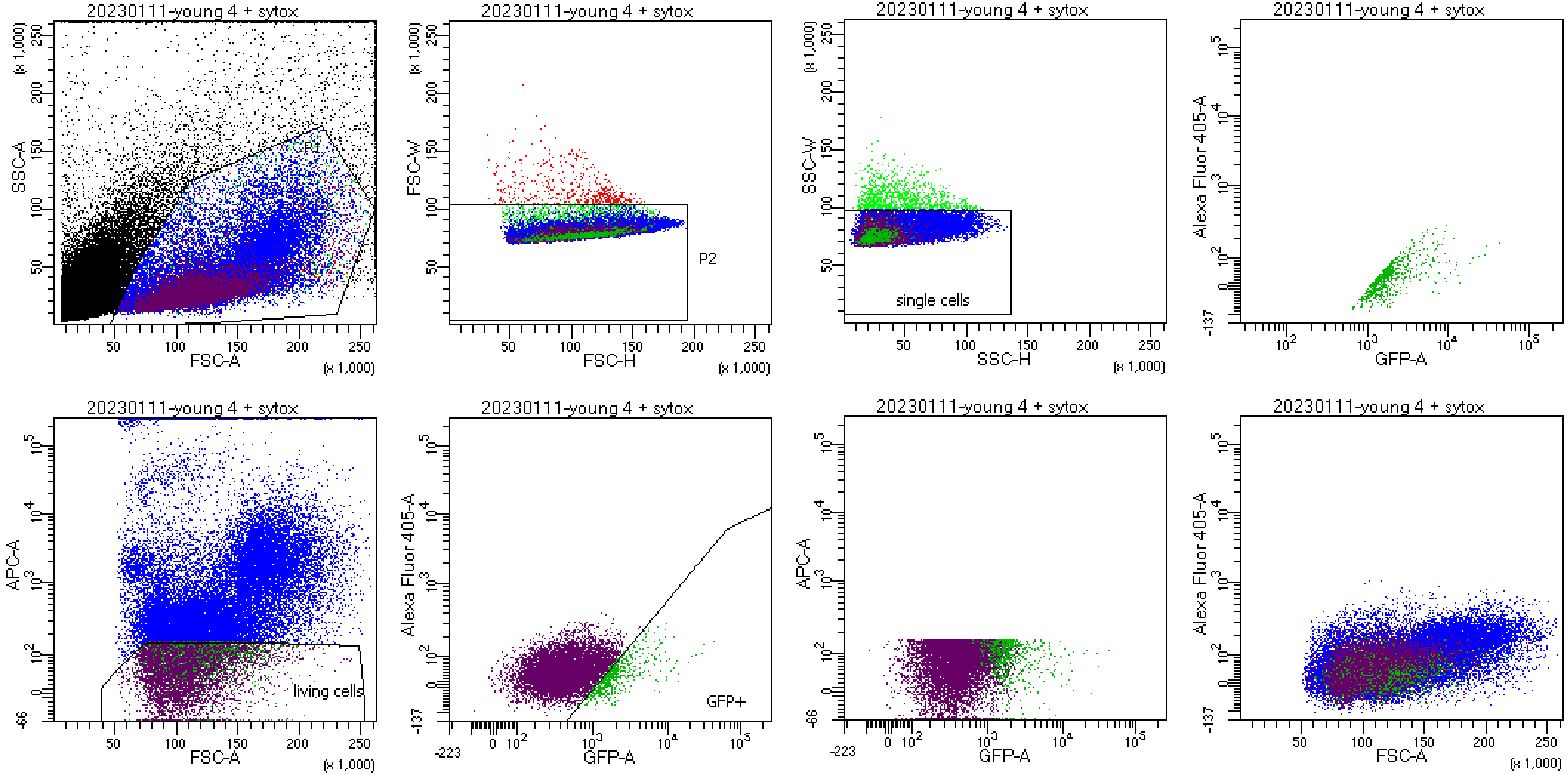

Tube: young 4 + sytox			
Population	#Events	%Parent	%Total
All Events	101,628	####	100.0
P1	36,706	36.1	36.1
P2	36,230	98.7	35.6
single cells	35,211	97.2	34.6
living cells	10,000	28.4	9.8
GFP+	632	6.3	0.6

BD FACSDiva 9.0.1

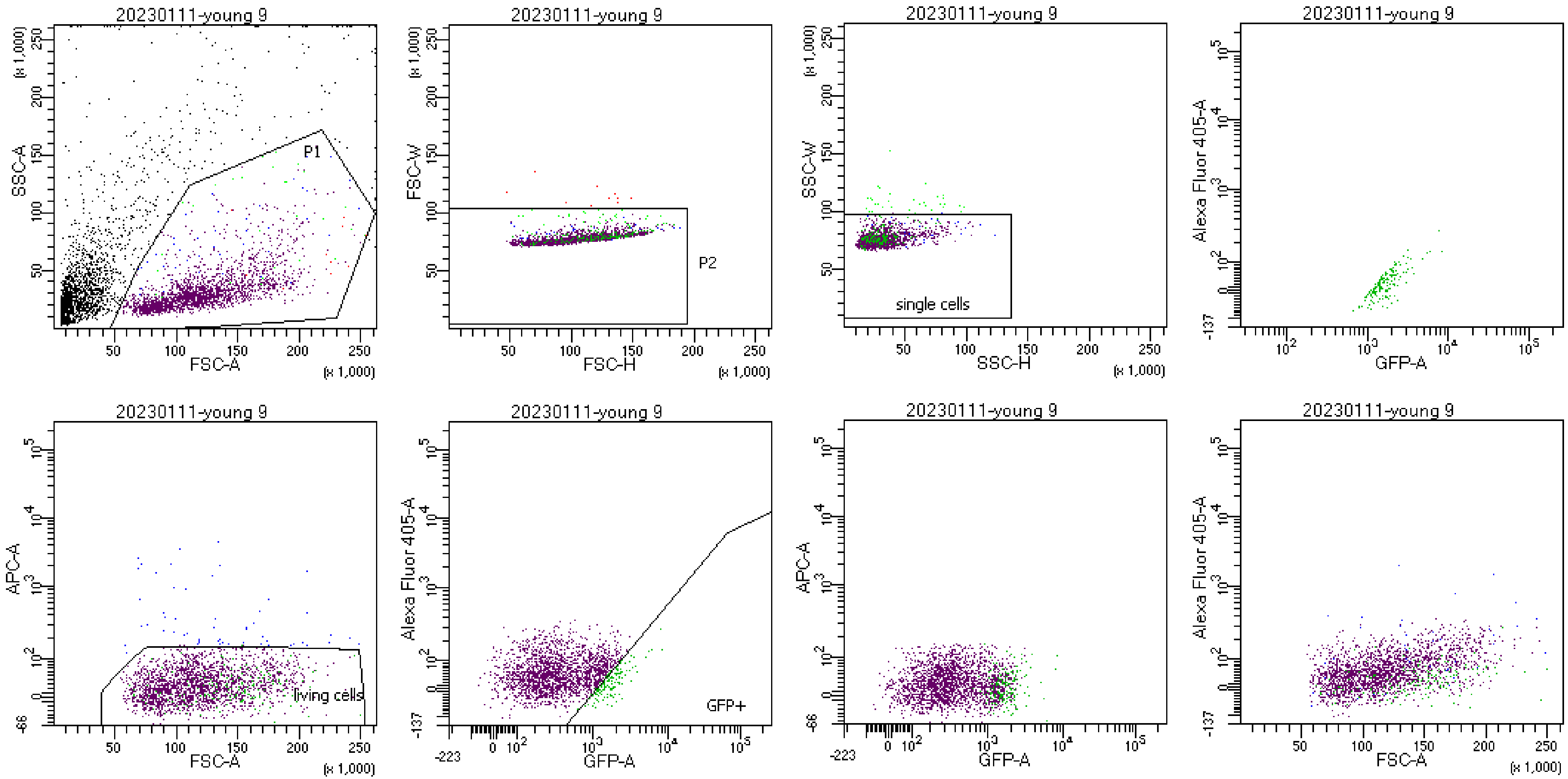

Tube: young 9

Population	#Events	%Parent	%Total
All Events	4,137	####	100.0
P1	2,241	54.2	54.2
P2	2,230	99.5	53.9
single cells	2,198	98.6	53.1
living cells	2,144	97.5	51.8
GFP+	160	7.5	3.9

BD FACSDiva 9.0.1

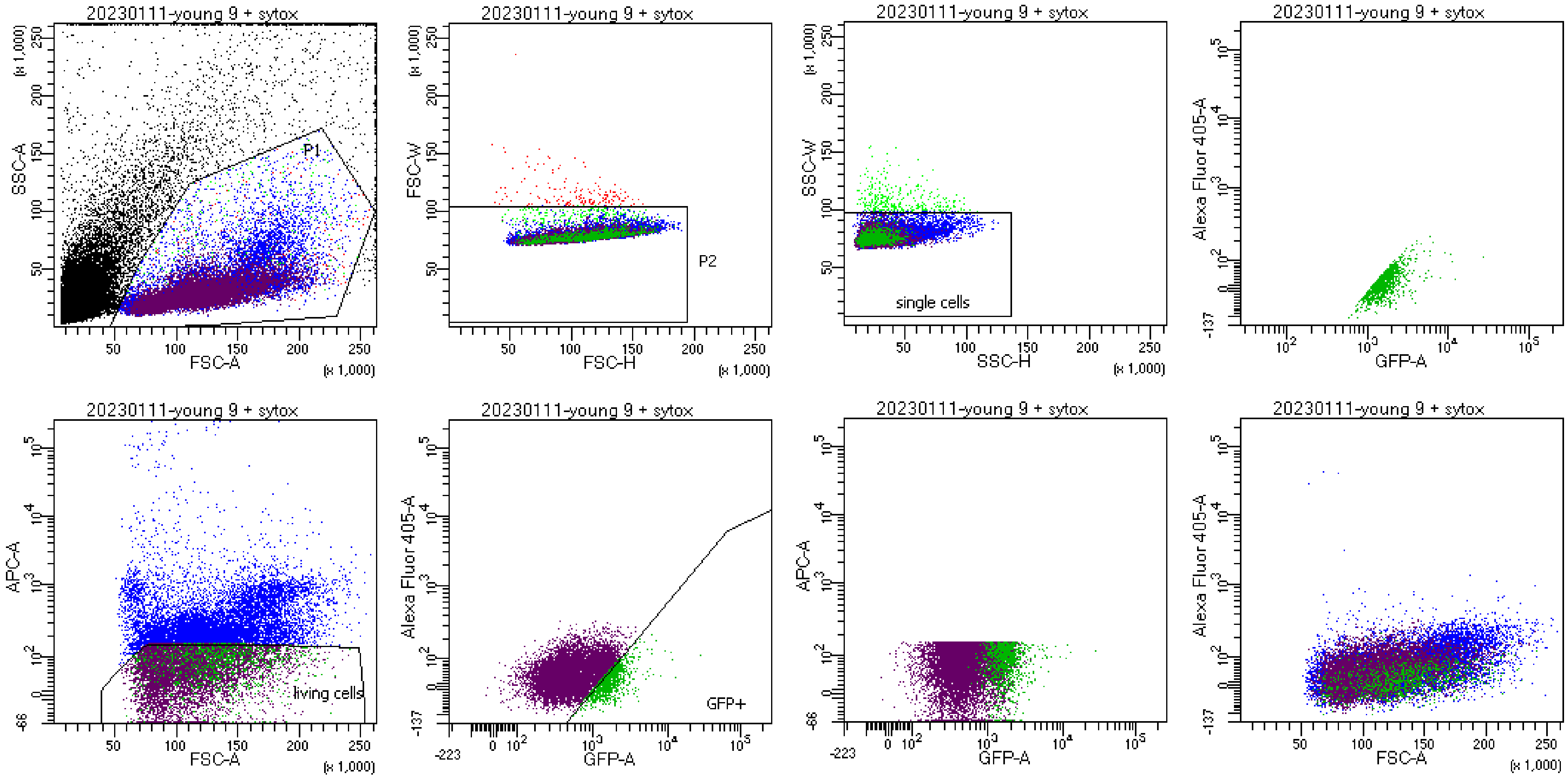

Tube: young 9 + sytox

Population	#Events	%Parent	%Total
All Events	36,295	####	100.0
P1	19,434	53.5	53.5
P2	19,276	99.2	53.1
single cells	18,950	98.3	52.2
living cells	10,000	52.8	27.6
GFP+	1,050	10.5	2.9

BD FACSDiva 9.0.1

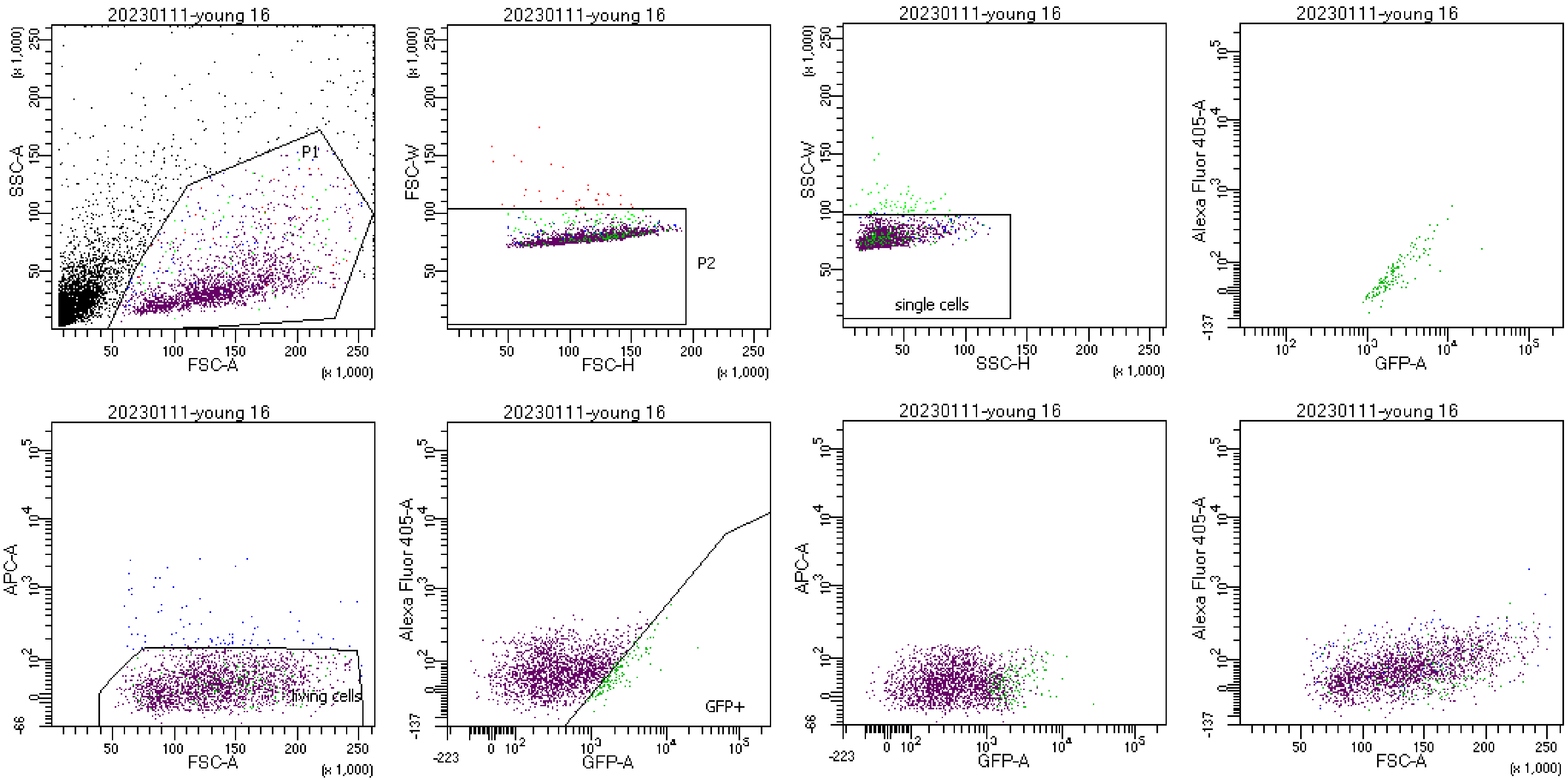

Tube: young 16

Population	#Events	%Parent	%Total
All Events	6,508	####	100.0
P1	2,310	35.5	35.5
P2	2,277	98.6	35.0
single cells	2,213	97.2	34.0
living cells	2,130	96.2	32.7
GFP+	140	6.6	2.2

BD FACSDiva 9.0.1

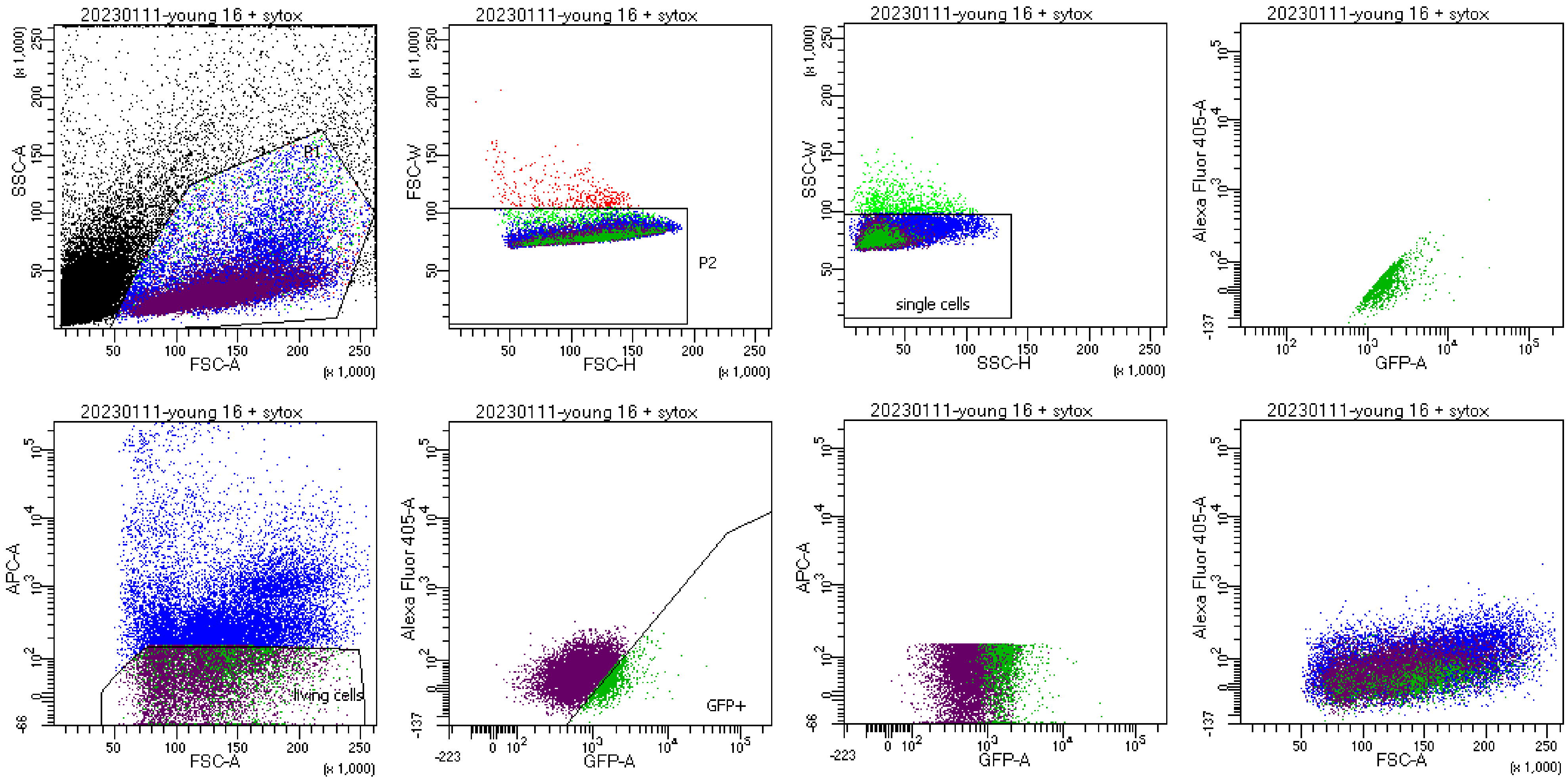

Tube: young 16 + sytox

Population	#Events	%Parent	%Total
All Events	69,255	####	100.0
P1	24,712	35.7	35.7
P2	24,298	98.3	35.1
single cells	23,371	96.2	33.7
living cells	10,000	42.8	14.4
GFP+	1,176	11.8	1.7
