# Supplementary material for: Generation of a transparent killifish line through multiplex CRISPR/Cas9mediated gene inactivation
Source: eLife. 2023 Feb 23;12:e81549. doi: 10.7554/eLife.81549 (PMC10010688; doi:10.7554/eLife.81549)
Supplement: Figure 5—source data 1. [file elife-81549-fig5-data1.zip › Figure_5_source_data/Figure_5_panel_I_source_data/FACS_raw_data/Male_kidney_p21ki-Batch_Analysis_12012023171103.pdf]

BD FACSDiva 9.0.1

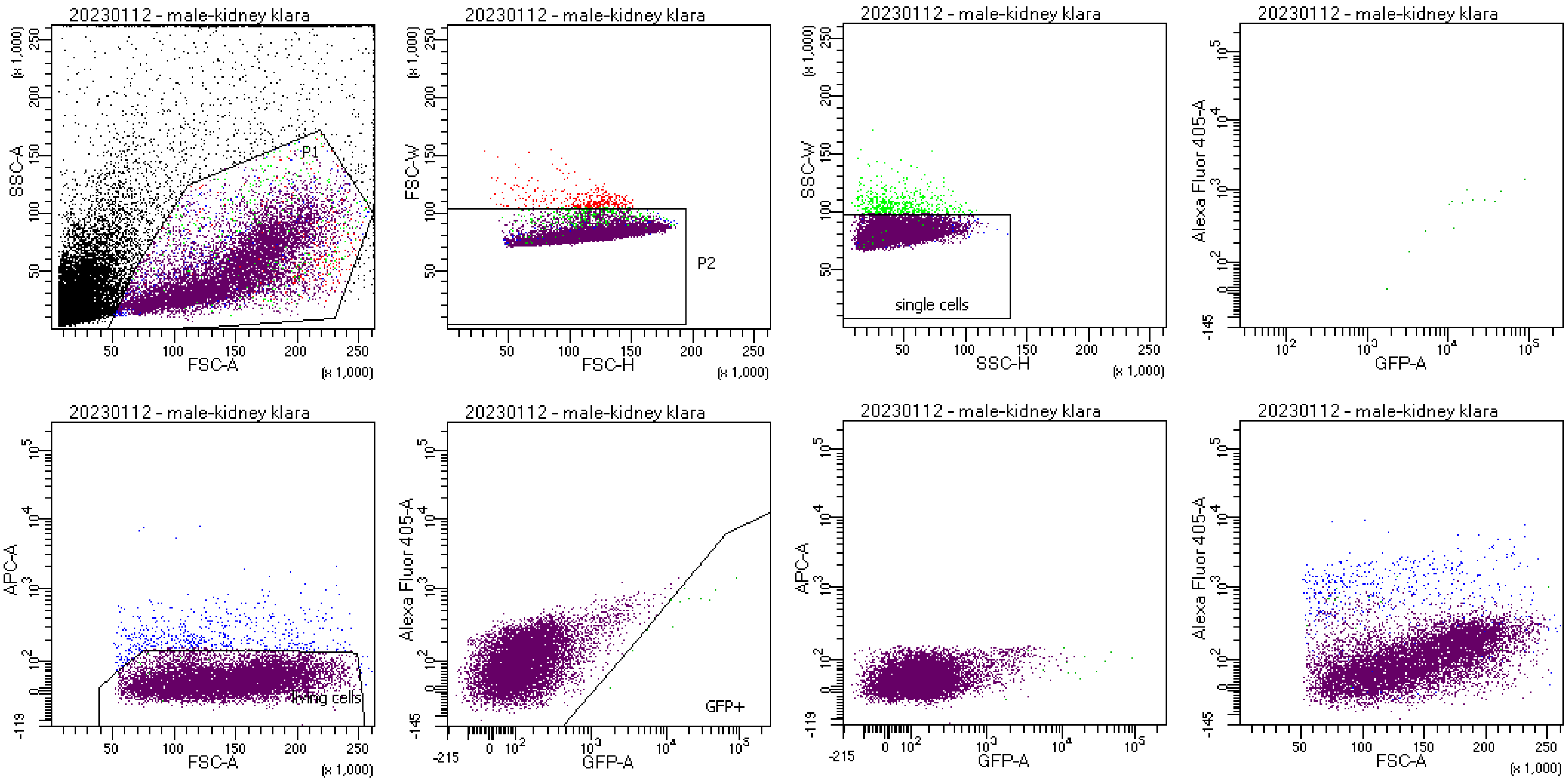

Tube: kidney klara

Population	#Events	%Parent	%Total
All Events	29,598	####	100.0
P1	11,384	38.5	38.5
P2	11,030	96.9	37.3
single cells	10,522	95.4	35.5
living cells	10,000	95.0	33.8
GFP+	13	0.1	0.0

BD FACSDiva 9.0.1

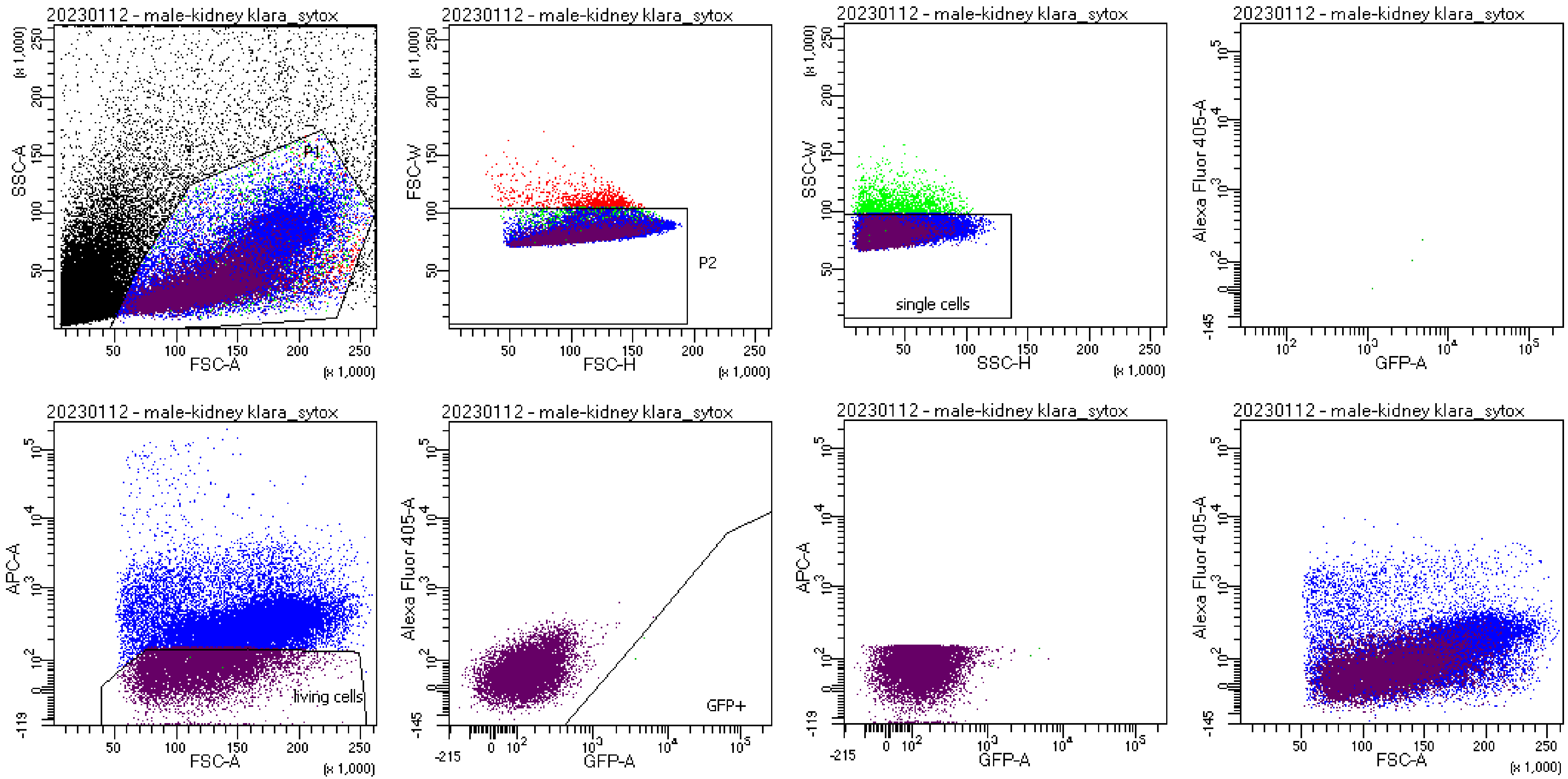

Tube: kidney klara_sytox

Population	#Events	%Parent	%Total
All Events	83,100	####	100.0
P1	32,230	38.8	38.8
P2	31,177	96.7	37.5
single cells	29,477	94.5	35.5
living cells	10,000	33.9	12.0
GFP+	3	0.0	0.0

BD FACSDiva 9.0.1

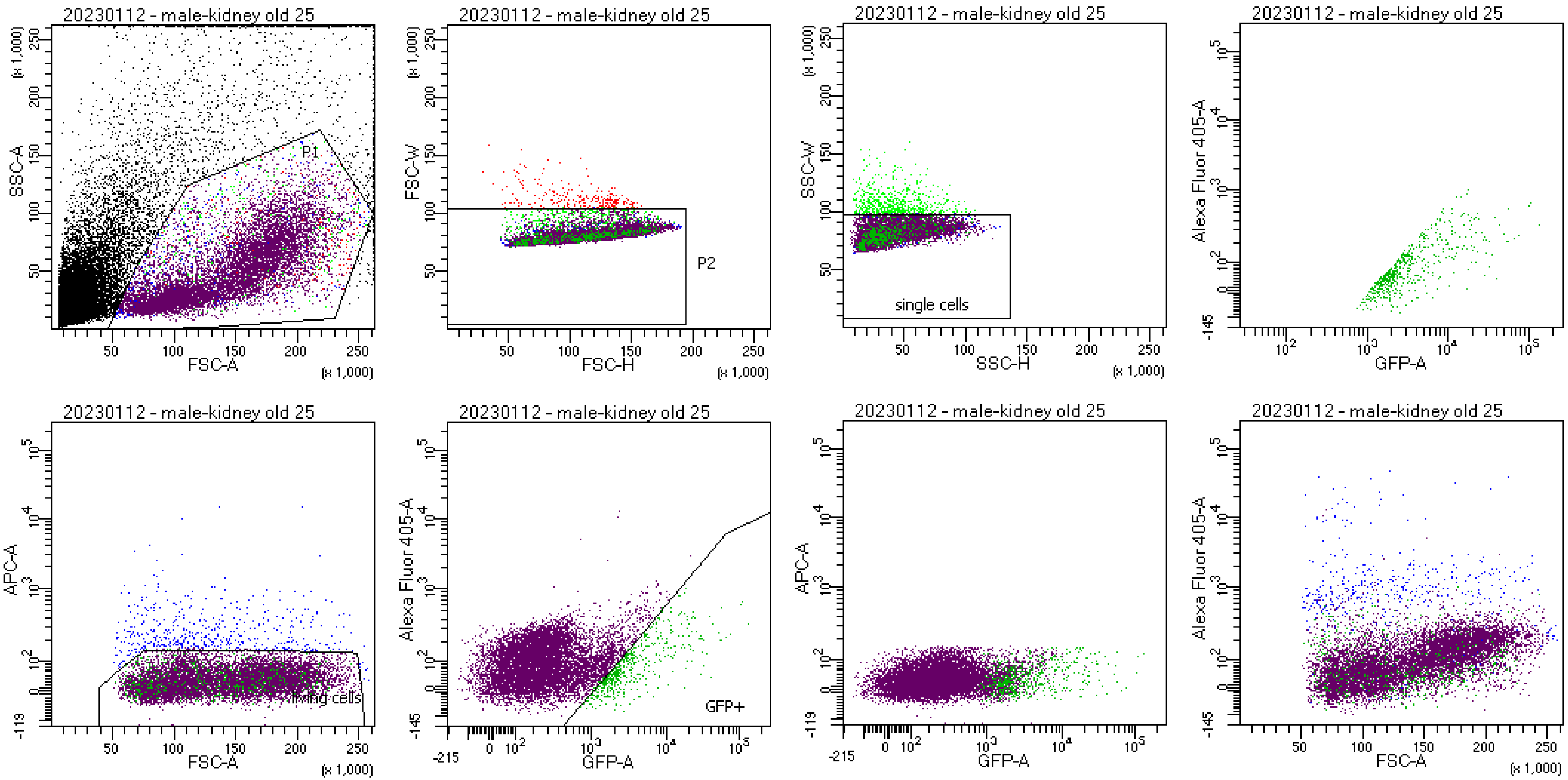

Tube: kidney old 25

Population	#Events	%Parent	%Total
All Events	30,409	####	100.0
P1	11,282	37.1	37.1
P2	11,041	97.9	36.3
single cells	10,486	95.0	34.5
living cells	10,000	95.4	32.9
GFP+	529	5.3	1.7

BD FACSDiva 9.0.1

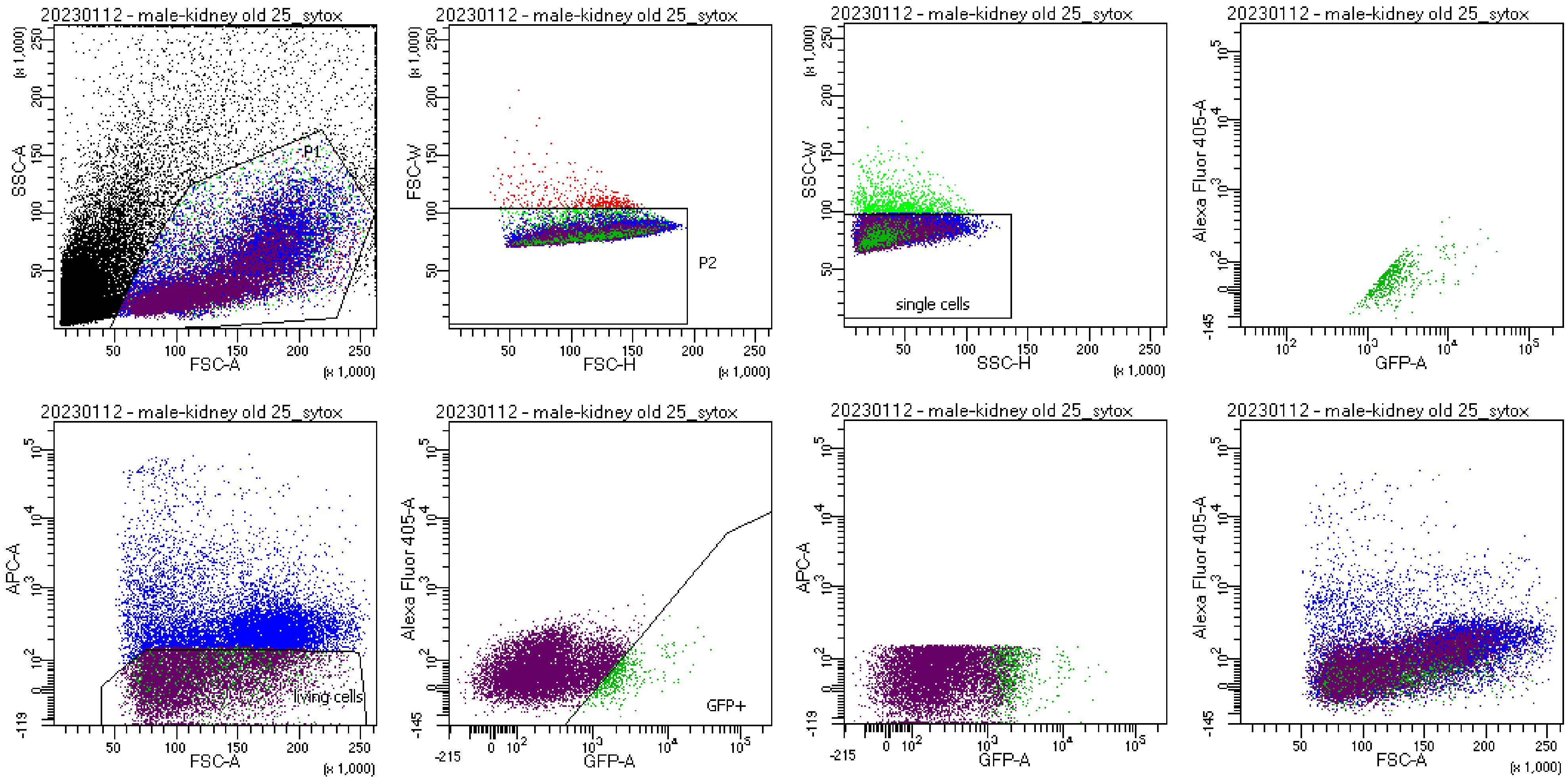

Tube: kidney old 25_sytox

Population	#Events	%Parent	%Total
All Events	59,388	####	100.0
P1	21,528	36.2	36.2
P2	21,111	98.1	35.5
single cells	20,017	94.8	33.7
living cells	10,000	50.0	16.8
GFP+	530	5.3	0.9

BD FACSDiva 9.0.1

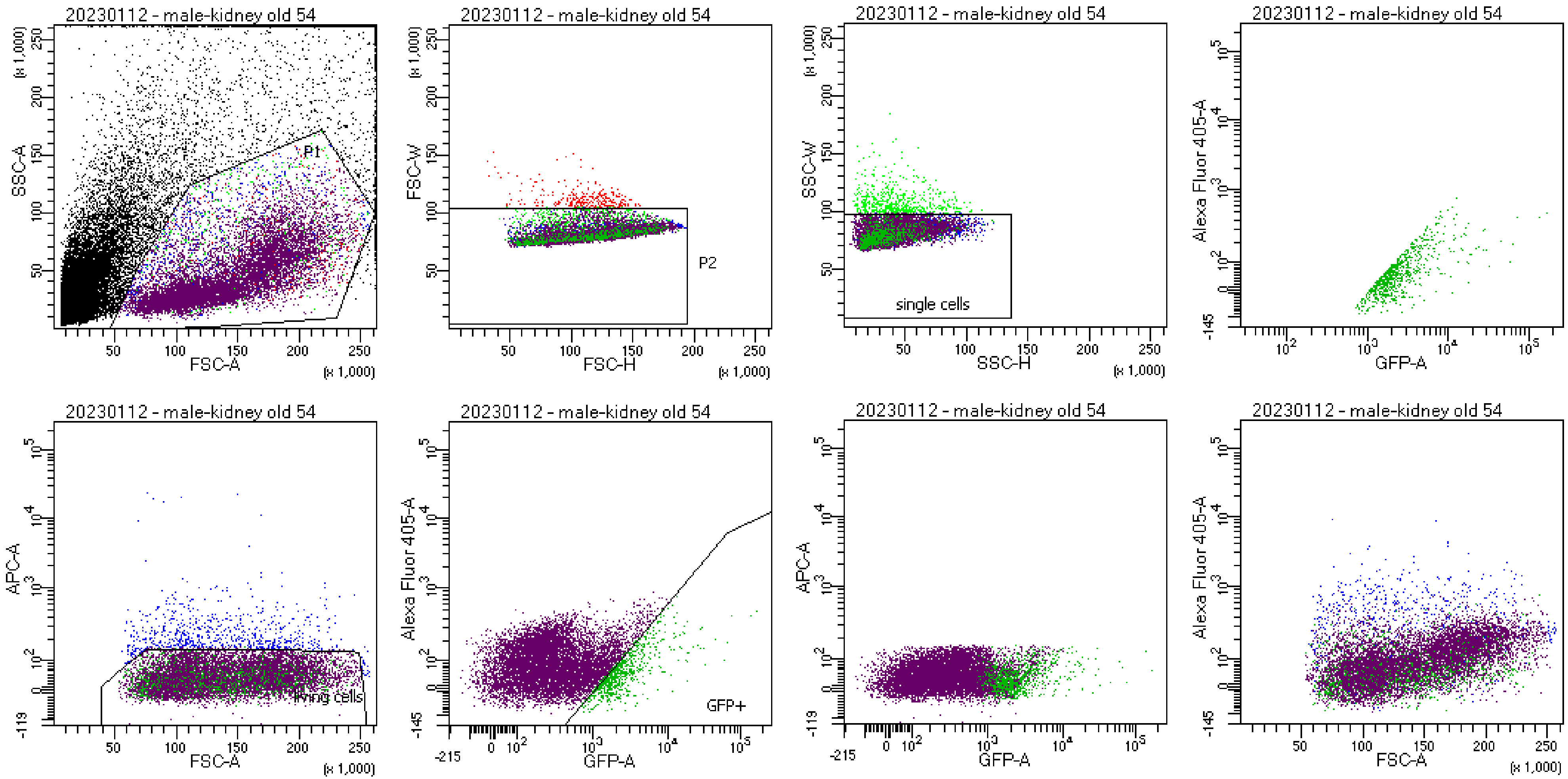

Tube: kidney old 54

Population	#Events	%Parent	%Total
All Events	33,047	####	100.0
P1	11,589	35.1	35.1
P2	11,301	97.5	34.2
single cells	10,709	94.8	32.4
living cells	10,000	93.4	30.3
GFP+	746	7.5	2.3

BD FACSDiva 9.0.1

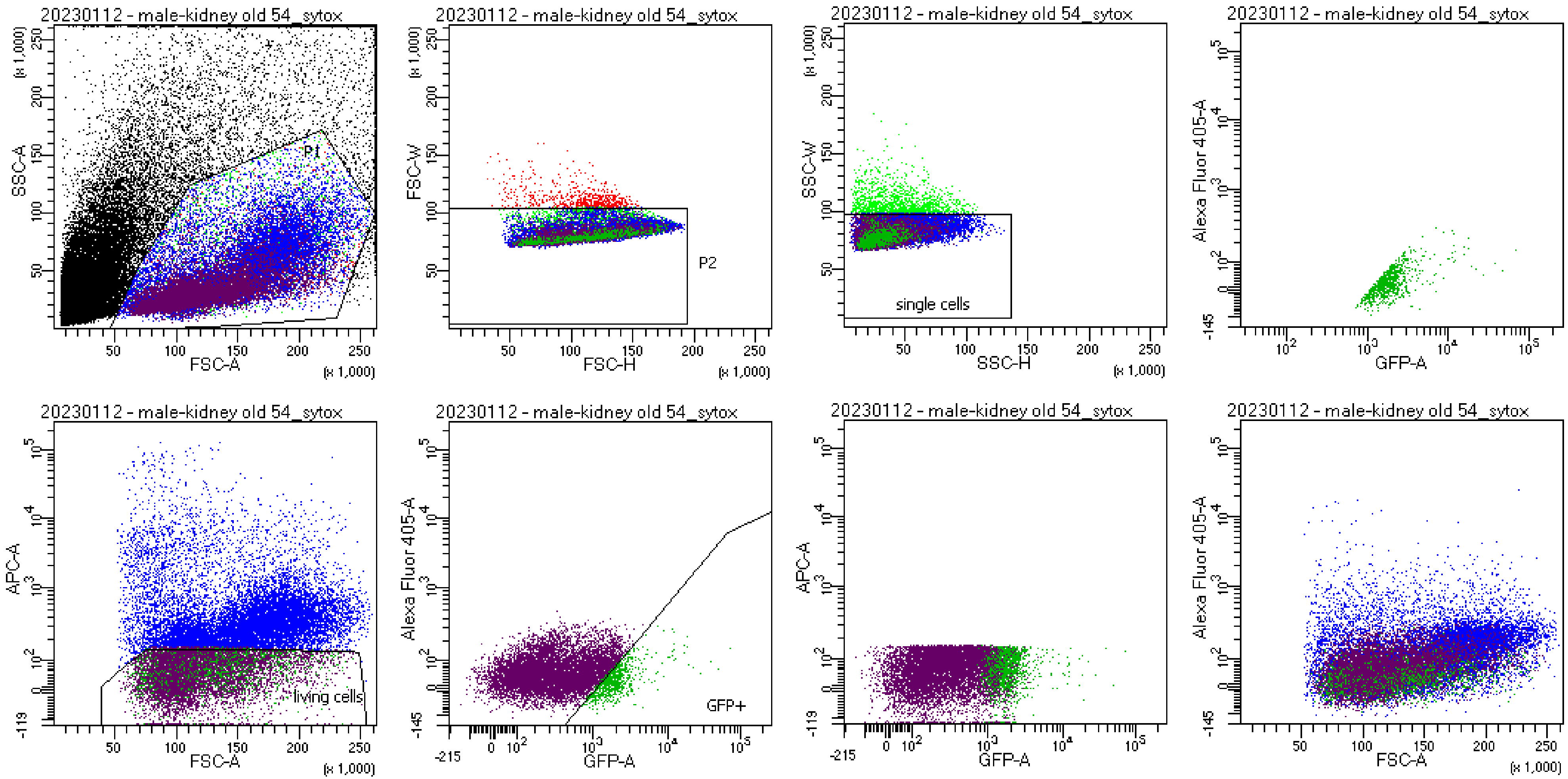

Tube: kidney old 54_sytox

Population	#Events	%Parent	%Total
All Events	71,787	####	100.0
P1	25,011	34.8	34.8
P2	24,393	97.5	34.0
single cells	23,079	94.6	32.1
living cells	10,000	43.3	13.9
GFP+	871	8.7	1.2

BD FACSDiva 9.0.1

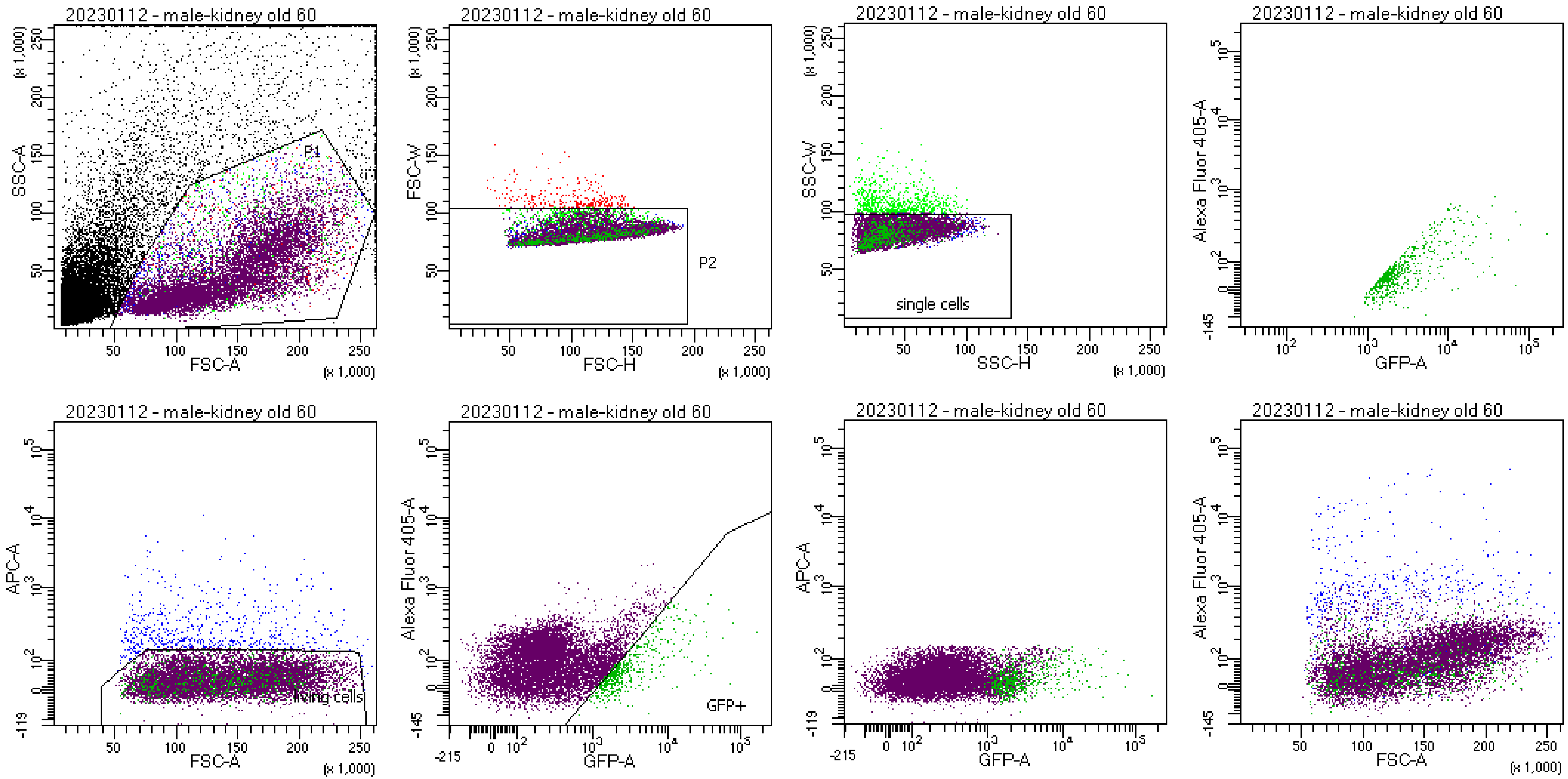

Tube: kidney old 60

Population	#Events	%Parent	%Total
All Events	28,619	####	100.0
P1	11,551	40.4	40.4
P2	11,267	97.5	39.4
single cells	10,551	93.6	36.9
living cells	10,000	94.8	34.9
GFP+	560	5.6	2.0

BD FACSDiva 9.0.1

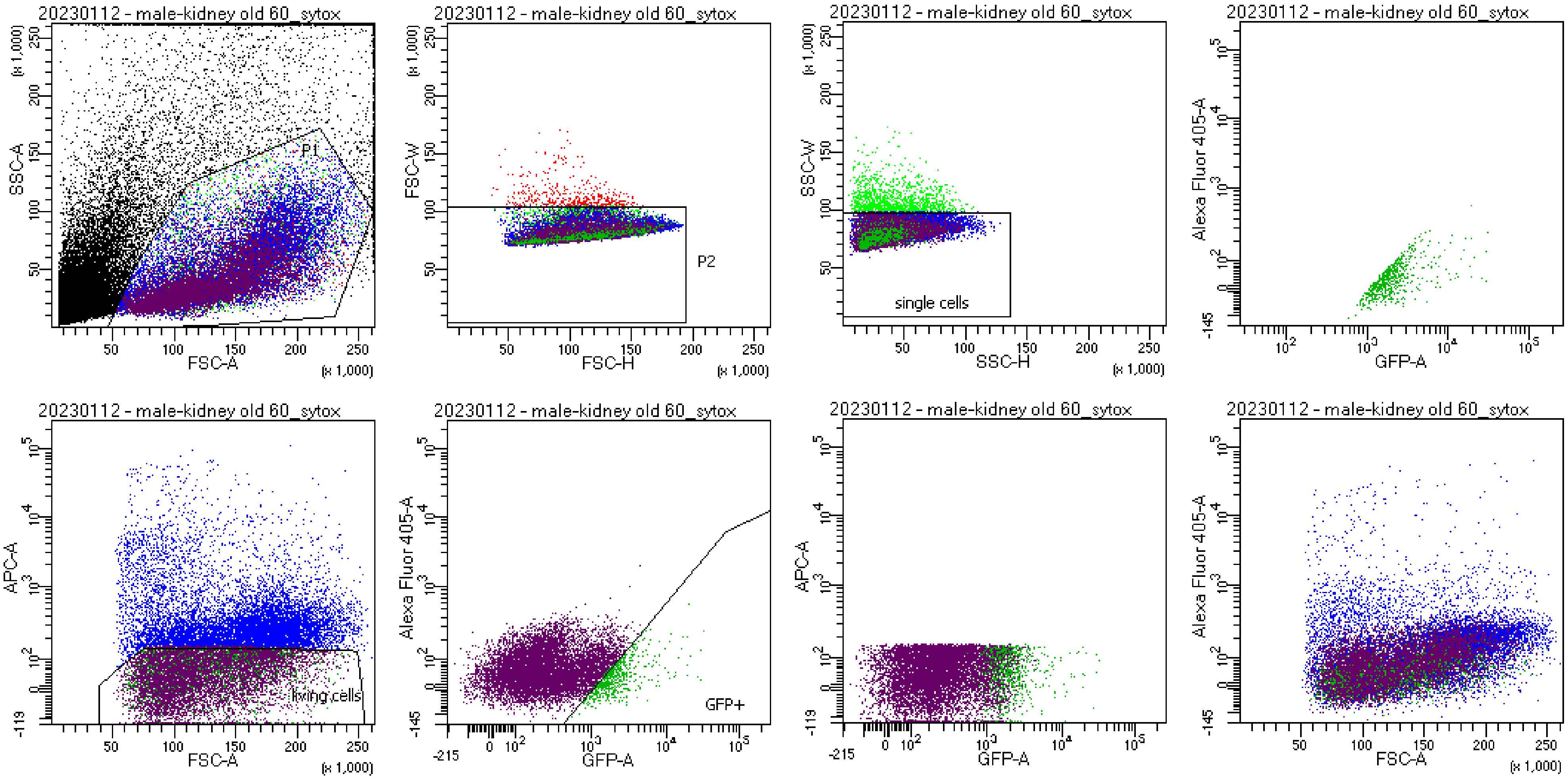

Tube: kidney old 60_sytox

Population	#Events	%Parent	%Total
All Events	52,205	####	100.0
P1	21,197	40.6	40.6
P2	20,735	97.8	39.7
single cells	19,370	93.4	37.1
living cells	10,000	51.6	19.2
GFP+	597	6.0	1.1

BD FACSDiva 9.0.1

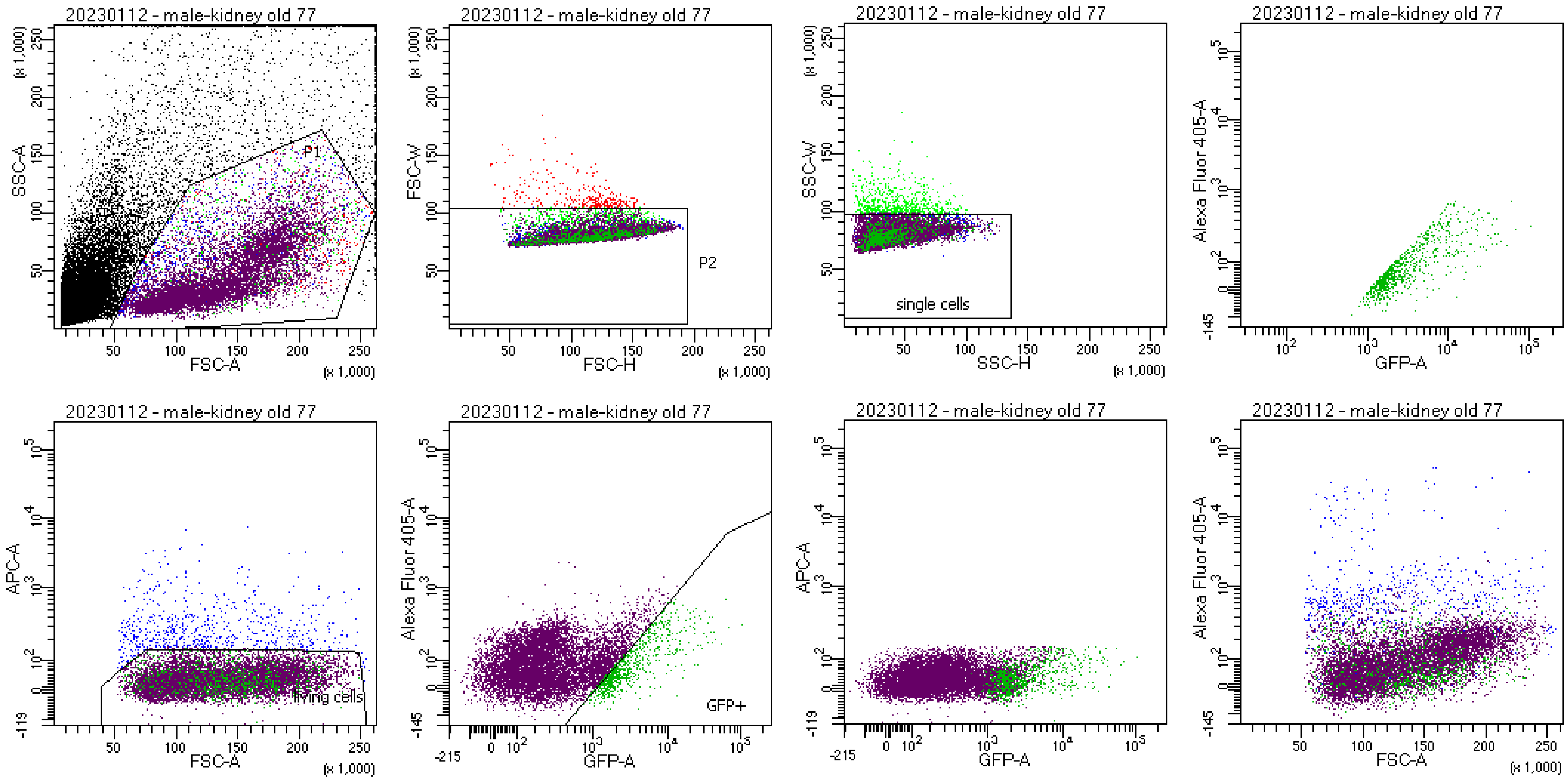

Tube: kidney old 77

Population	#Events	%Parent	%Total
All Events	32,601	####	100.0
P1	11,583	35.5	35.5
P2	11,276	97.3	34.6
single cells	10,634	94.3	32.6
living cells	10,000	94.0	30.7
GFP+	759	7.6	2.3

BD FACSDiva 9.0.1

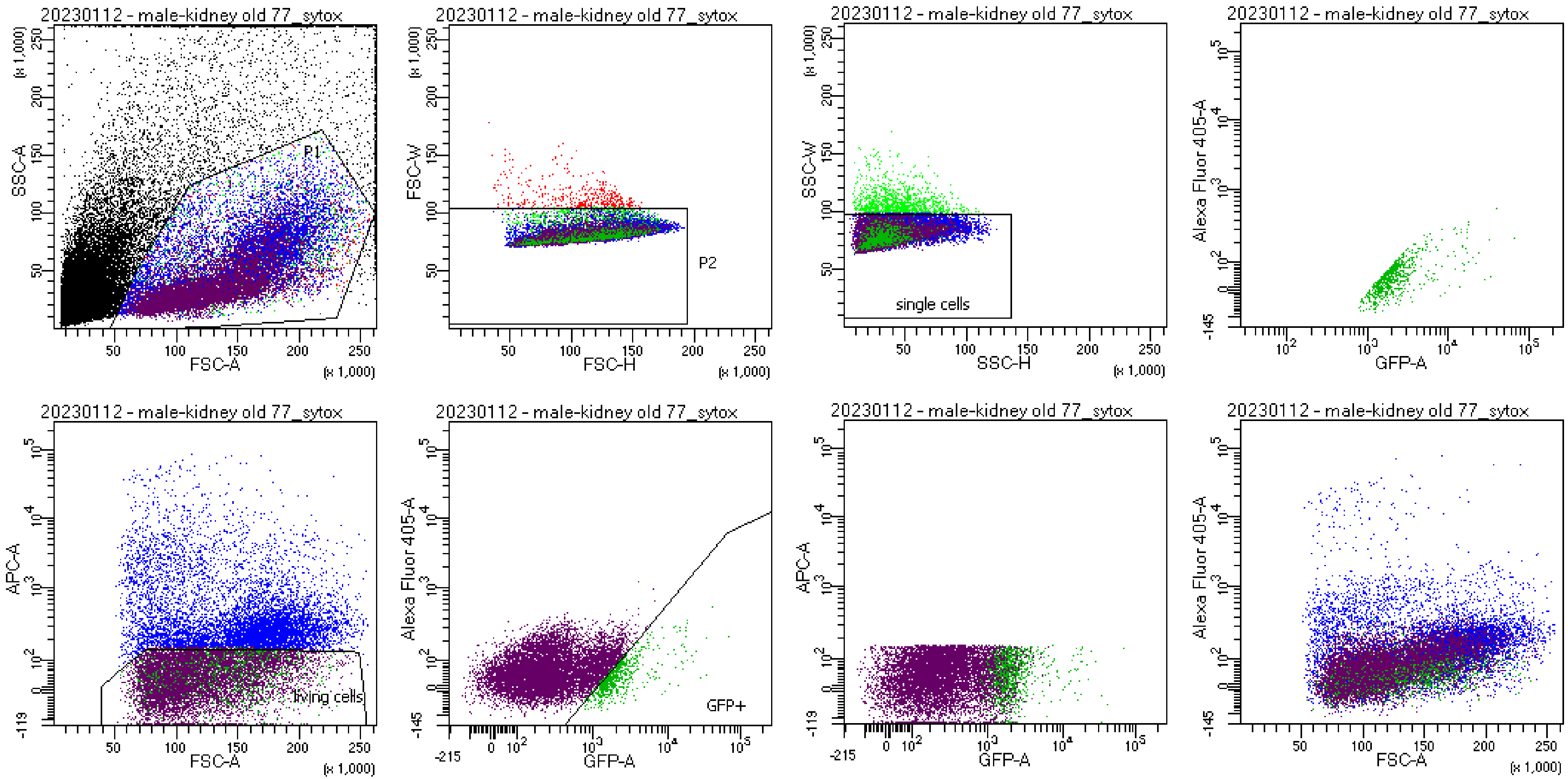

Tube: kidney old 77_sytox

Population	#Events	%Parent	%Total
All Events	52,348	####	100.0
P1	18,827	36.0	36.0
P2	18,423	97.9	35.2
single cells	17,369	94.3	33.2
living cells	10,000	57.6	19.1
GFP+	713	7.1	1.4

BD FACSDiva 9.0.1

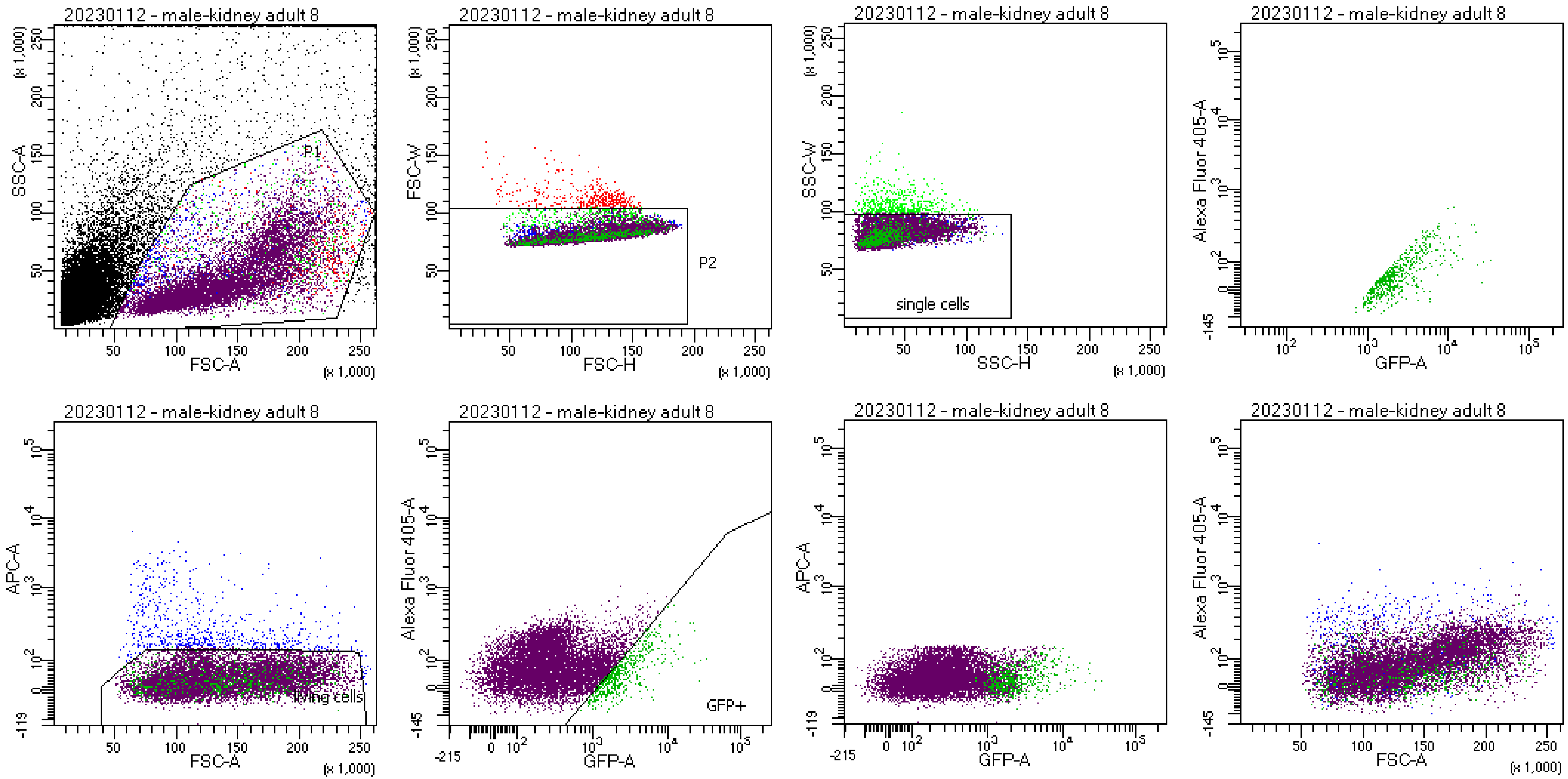

Tube: kidney adult 8

Population	#Events	%Parent	%Total
All Events	37,360	####	100.0
P1	11,587	31.0	31.0
P2	11,155	96.3	29.9
single cells	10,662	95.6	28.5
living cells	10,000	93.8	26.8
GFP+	567	5.7	1.5

BD FACSDiva 9.0.1

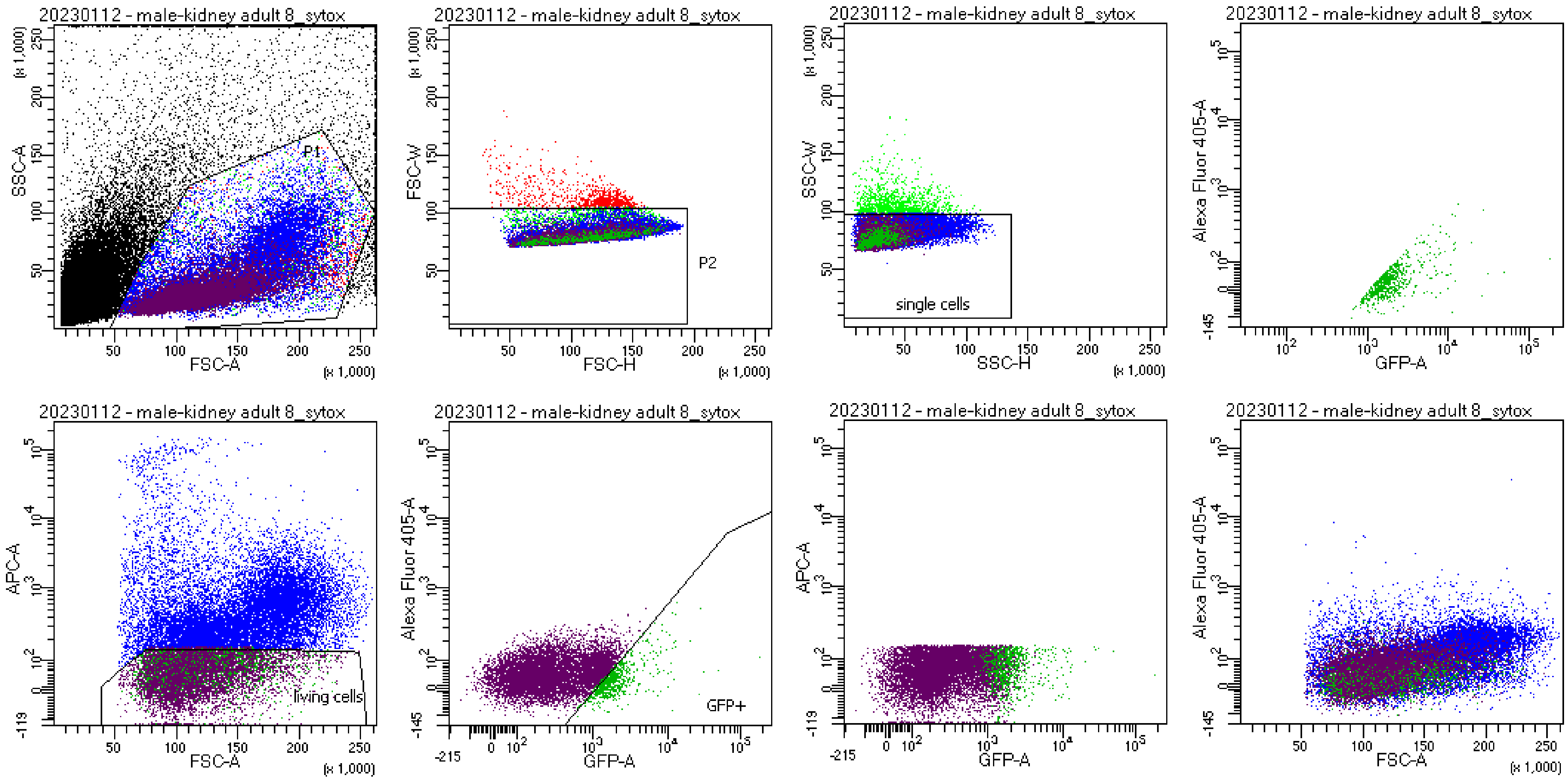

Tube: kidney adult 8_sytox

Population	#Events	%Parent	%Total
All Events	89,148	####	100.0
P1	26,640	29.9	29.9
P2	25,753	96.7	28.9
single cells	24,598	95.5	27.6
living cells	10,000	40.7	11.2
GFP+	690	6.9	0.8

BD FACSDiva 9.0.1

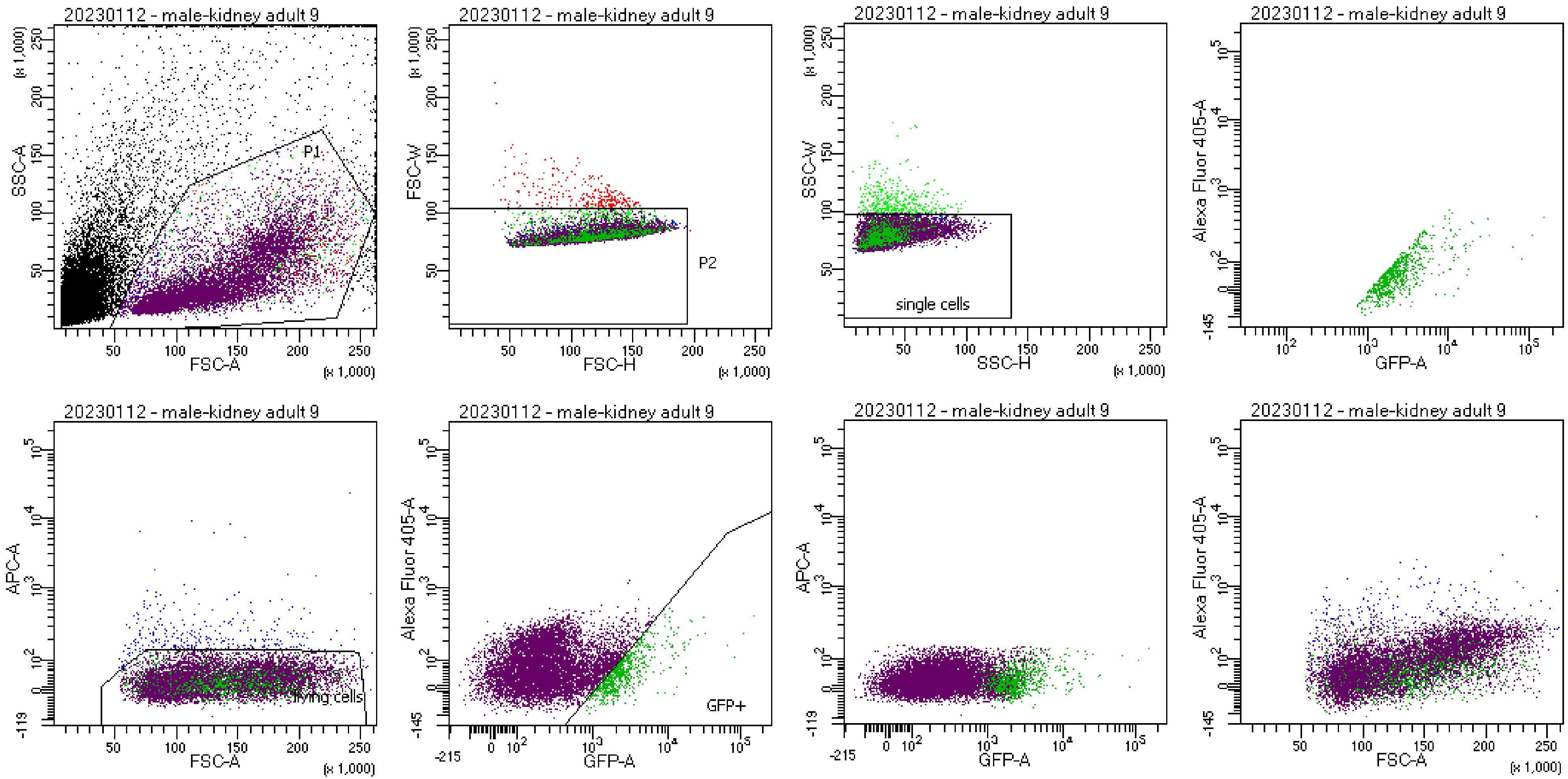

Tube: kidney adult 9

Population	#Events	%Parent	%Total
All Events	27,410	####	100.0
P1	10,975	40.0	40.0
P2	10,684	97.3	39.0
single cells	10,282	96.2	37.5
living cells	10,000	97.3	36.5
GFP+	795	8.0	2.9

BD FACSDiva 9.0.1

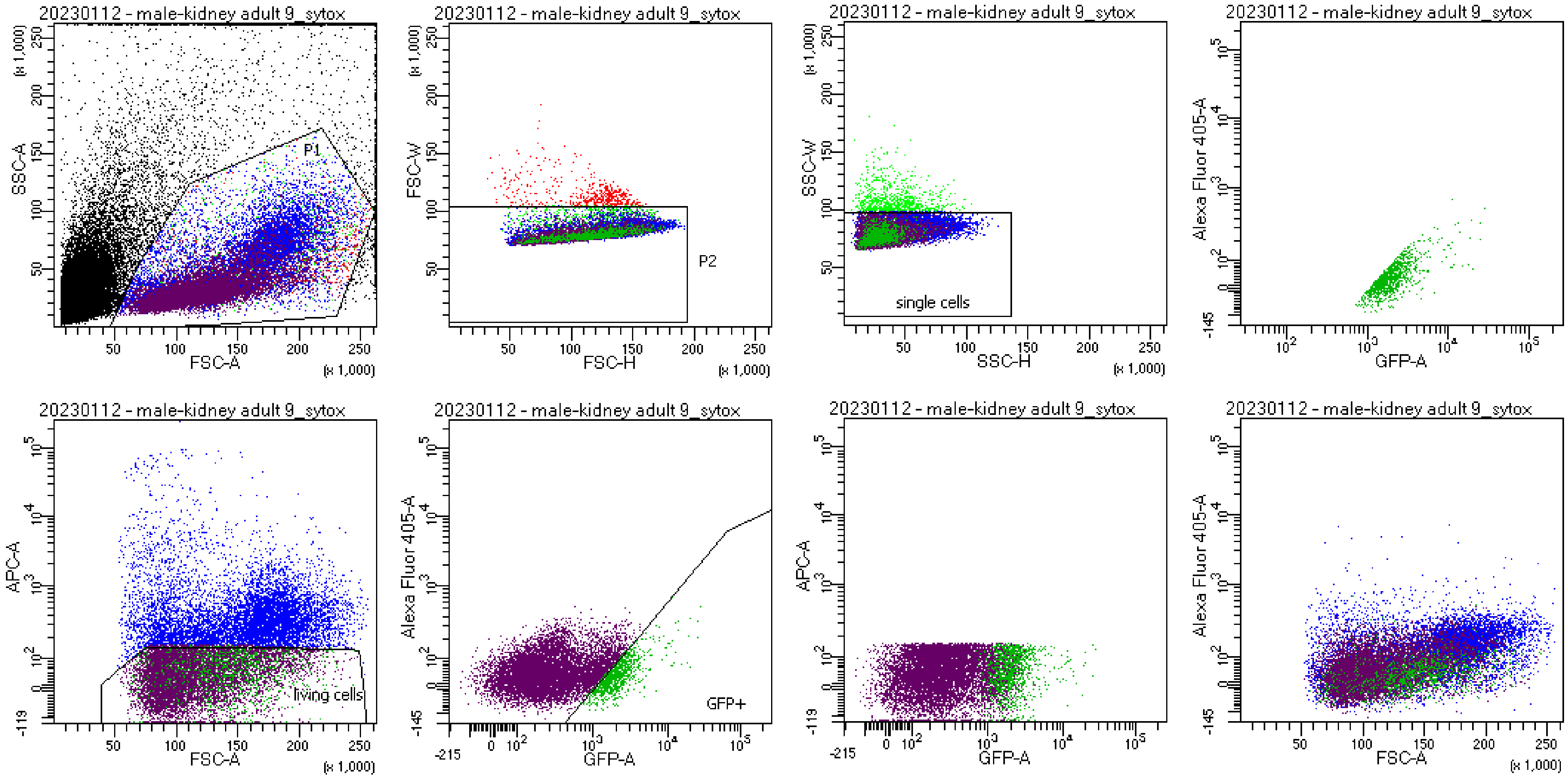

Tube: kidney adult 9_sytox

Population	#Events	%Parent	%Total
All Events	48,152	####	100.0
P1	19,459	40.4	40.4
P2	18,994	97.6	39.4
single cells	18,253	96.1	37.9
living cells	10,000	54.8	20.8
GFP+	993	9.9	2.1

BD FACSDiva 9.0.1

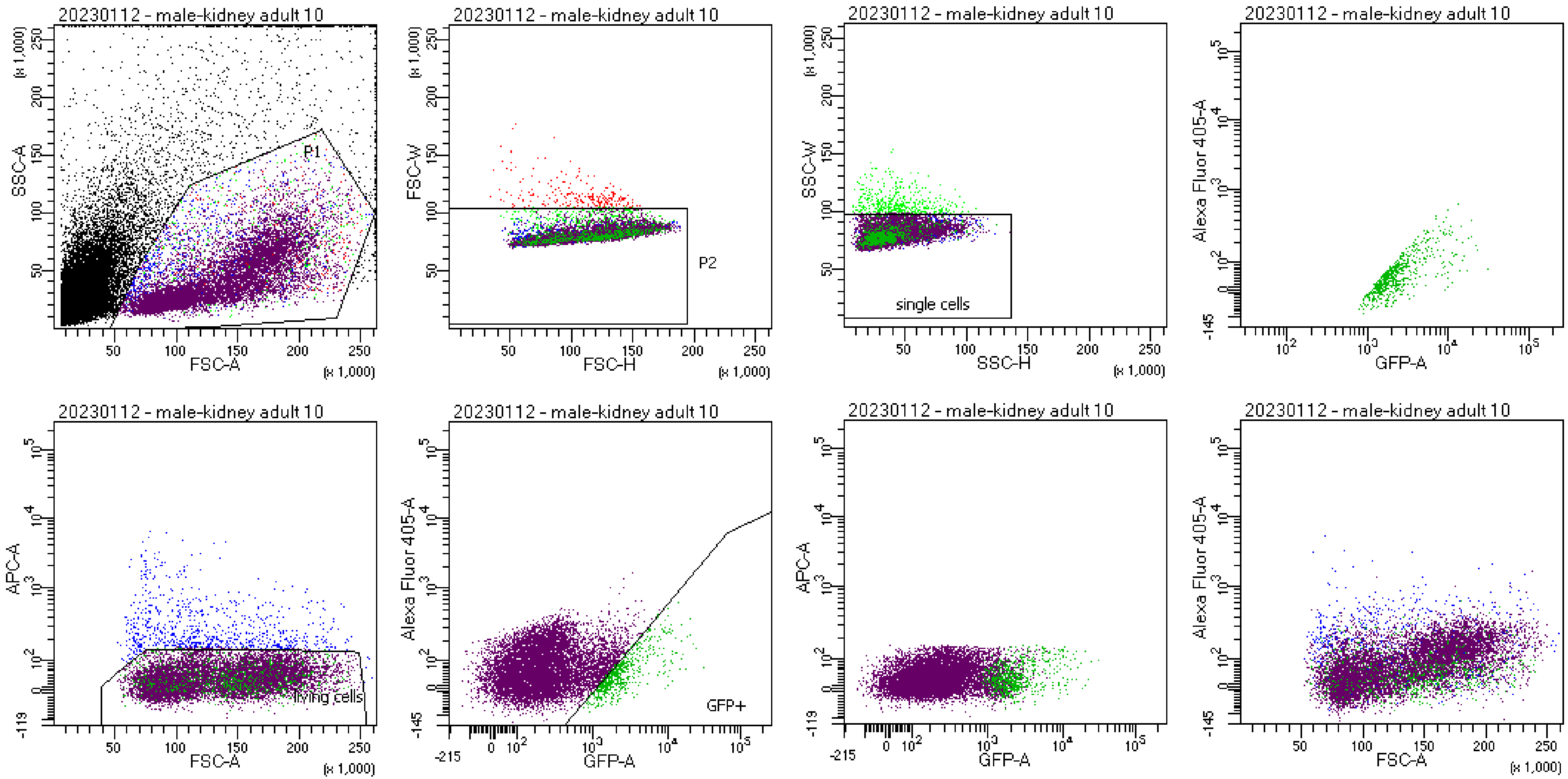

Tube: kidney adult 10

Population	#Events	%Parent	%Total
All Events	39,175	####	100.0
P1	11,437	29.2	29.2
P2	11,206	98.0	28.6
single cells	10,771	96.1	27.5
living cells	10,000	92.8	25.5
GFP+	644	6.4	1.6

BD FACSDiva 9.0.1

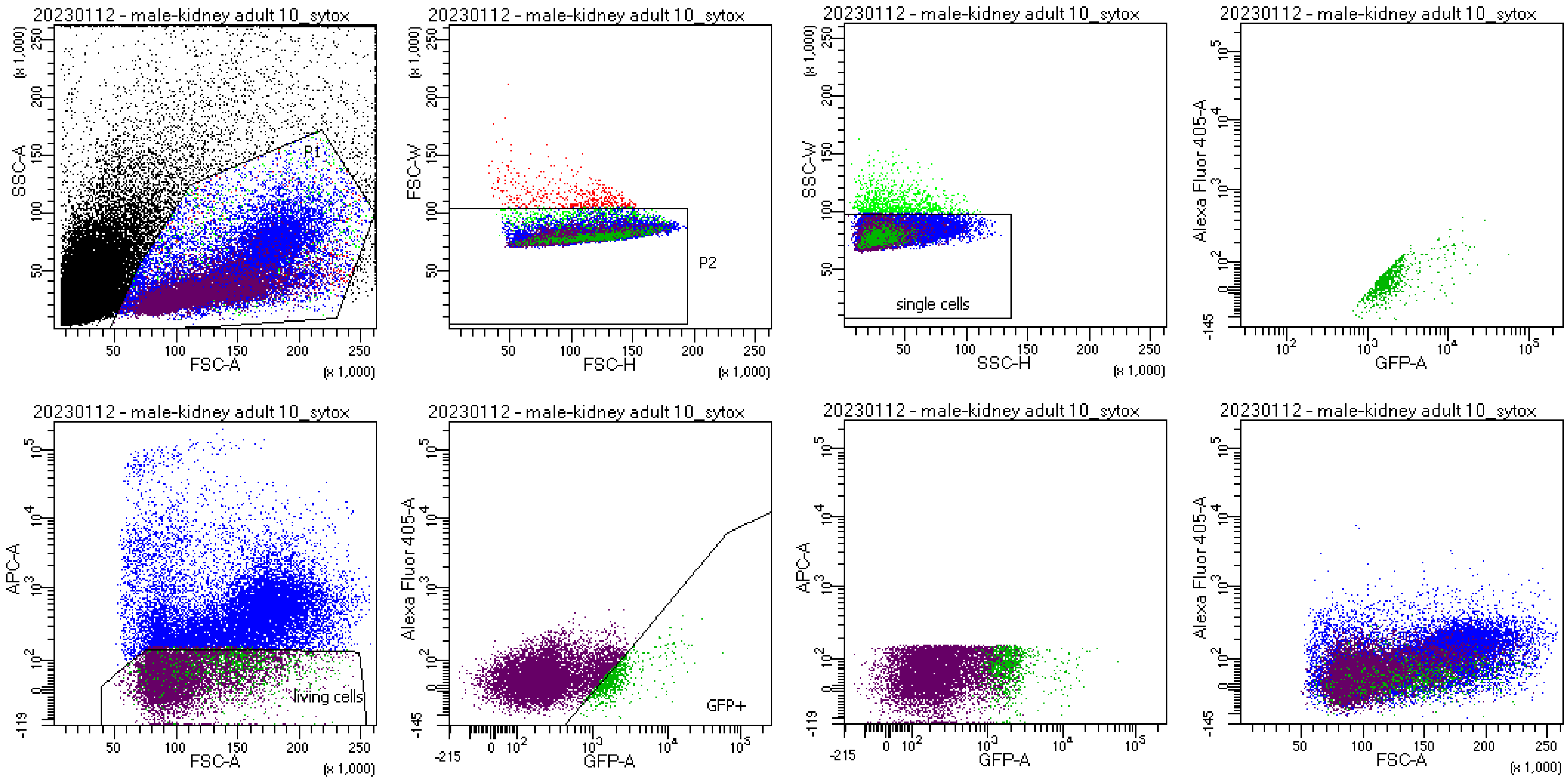

Tube: kidney adult 10_sytox

Population	#Events	%Parent	%Total
All Events	90,548	####	100.0
P1	26,108	28.8	28.8
P2	25,639	98.2	28.3
single cells	24,673	96.2	27.2
living cells	10,000	40.5	11.0
GFP+	751	7.5	0.8

BD FACSDiva 9.0.1

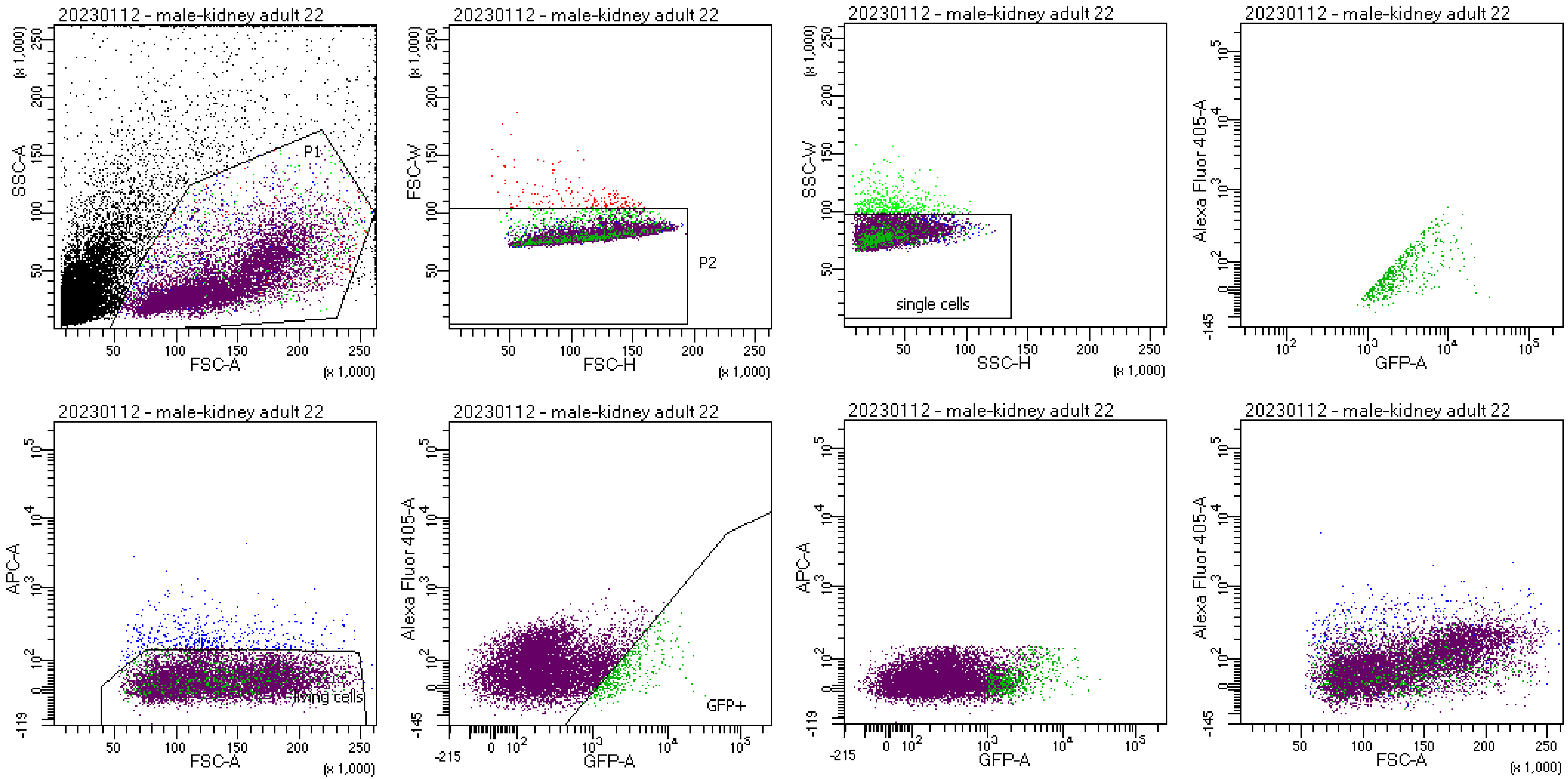

Tube: kidney adult 22

Population	#Events	%Parent	%Total
All Events	28,387	####	100.0
P1	11,046	38.9	38.9
P2	10,880	98.5	38.3
single cells	10,432	95.9	36.7
living cells	10,000	95.9	35.2
GFP+	487	4.9	1.7

BD FACSDiva 9.0.1

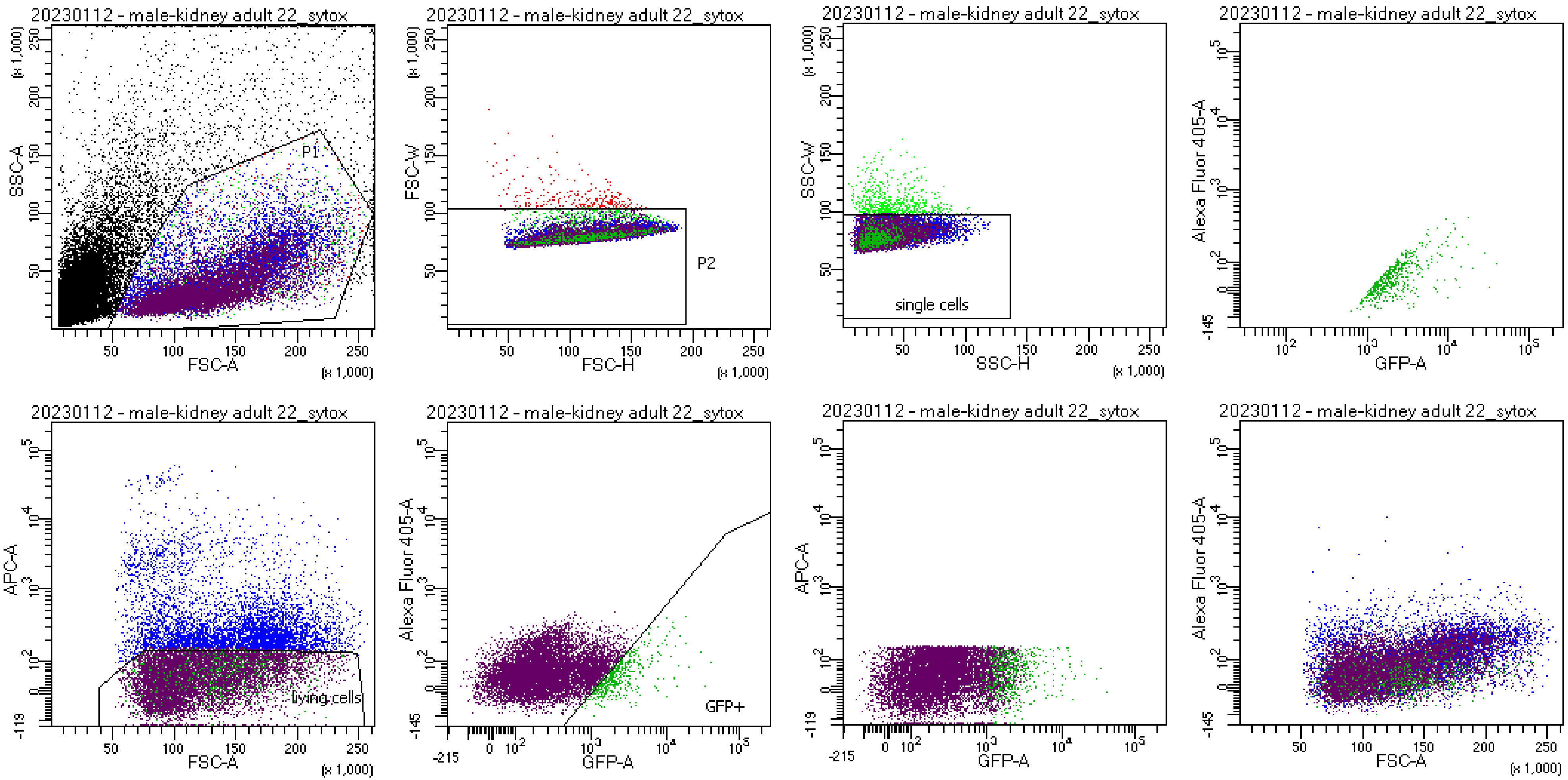

Tube: kidney adult 22_sytox

Population	#Events	%Parent	%Total
All Events	41,222	####	100.0
P1	16,019	38.9	38.9
P2	15,768	98.4	38.3
single cells	15,219	96.5	36.9
living cells	10,000	65.7	24.3
GFP+	506	5.1	1.2

BD FACSDiva 9.0.1

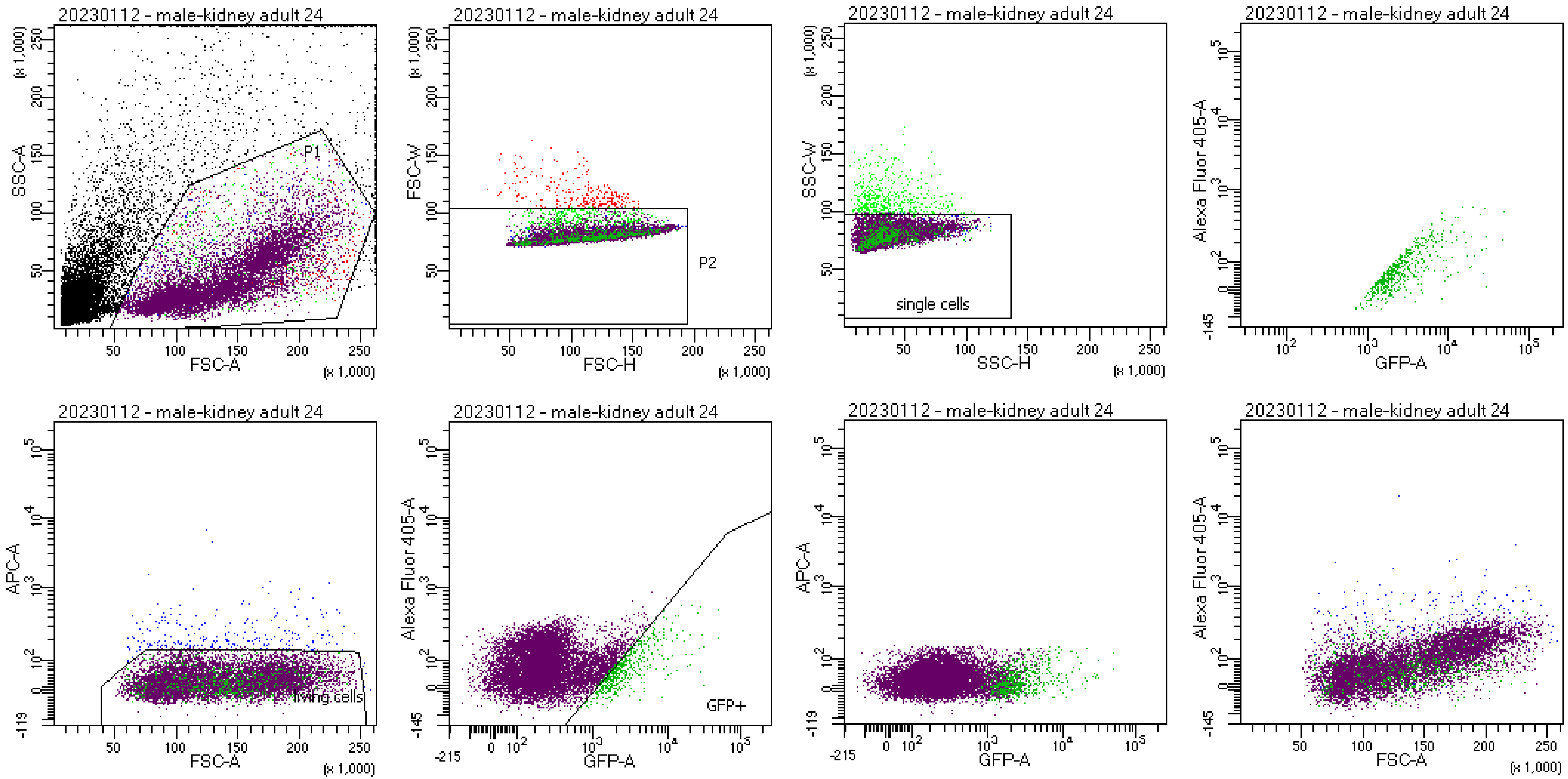

Tube: kidney adult 24

Population	#Events	%Parent	%Total
All Events	21,757	####	100.0
P1	11,007	50.6	50.6
P2	10,740	97.6	49.4
single cells	10,226	95.2	47.0
living cells	10,000	97.8	46.0
GFP+	544	5.4	2.5

BD FACSDiva 9.0.1

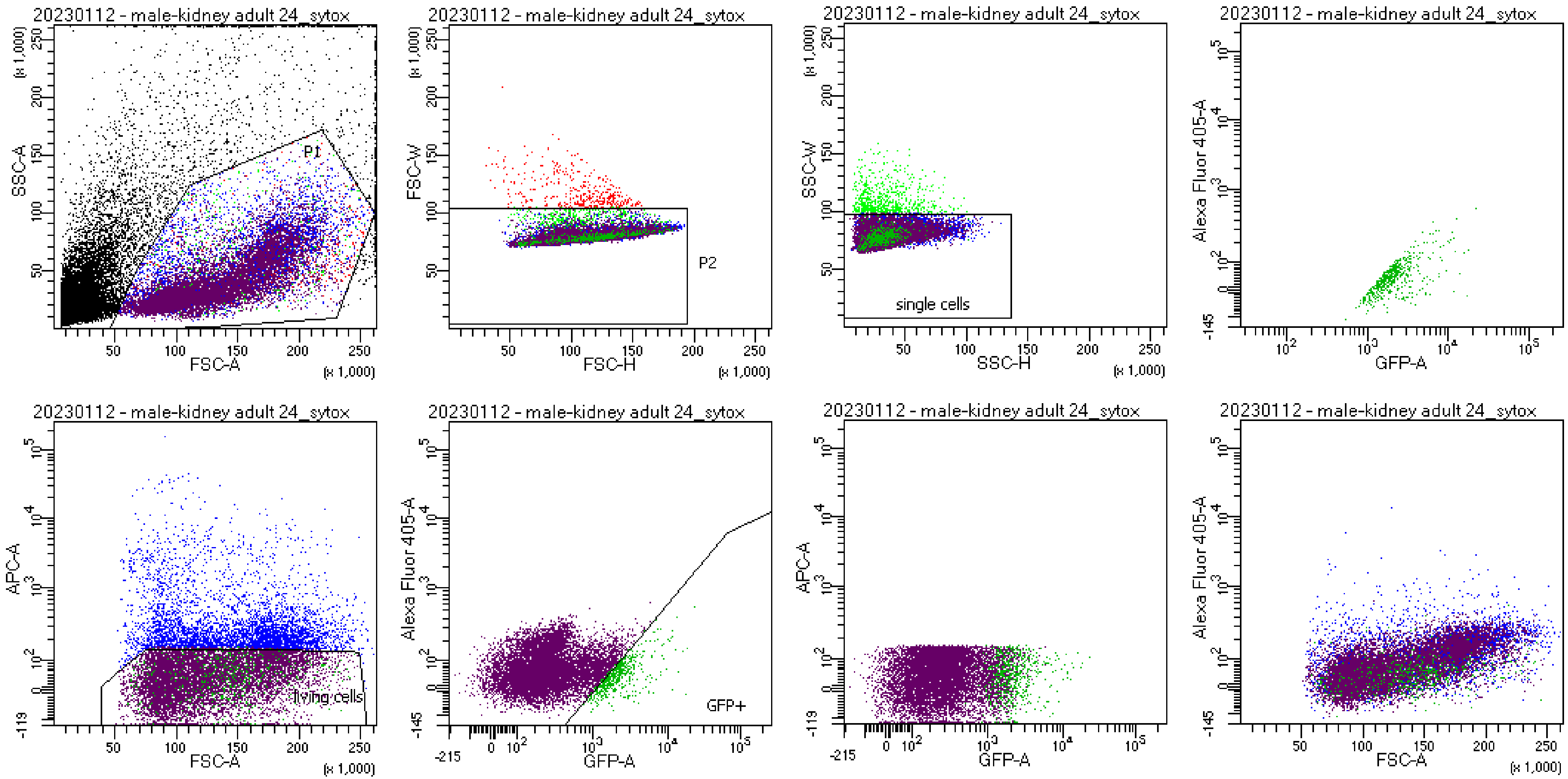

Tube: kidney adult 24_sytox

Population	#Events	%Parent	%Total
All Events	30,495	####	100.0
P1	15,188	49.8	49.8
P2	14,804	97.5	48.5
single cells	14,173	95.7	46.5
living cells	10,000	70.6	32.8
GFP+	522	5.2	1.7

BD FACSDiva 9.0.1

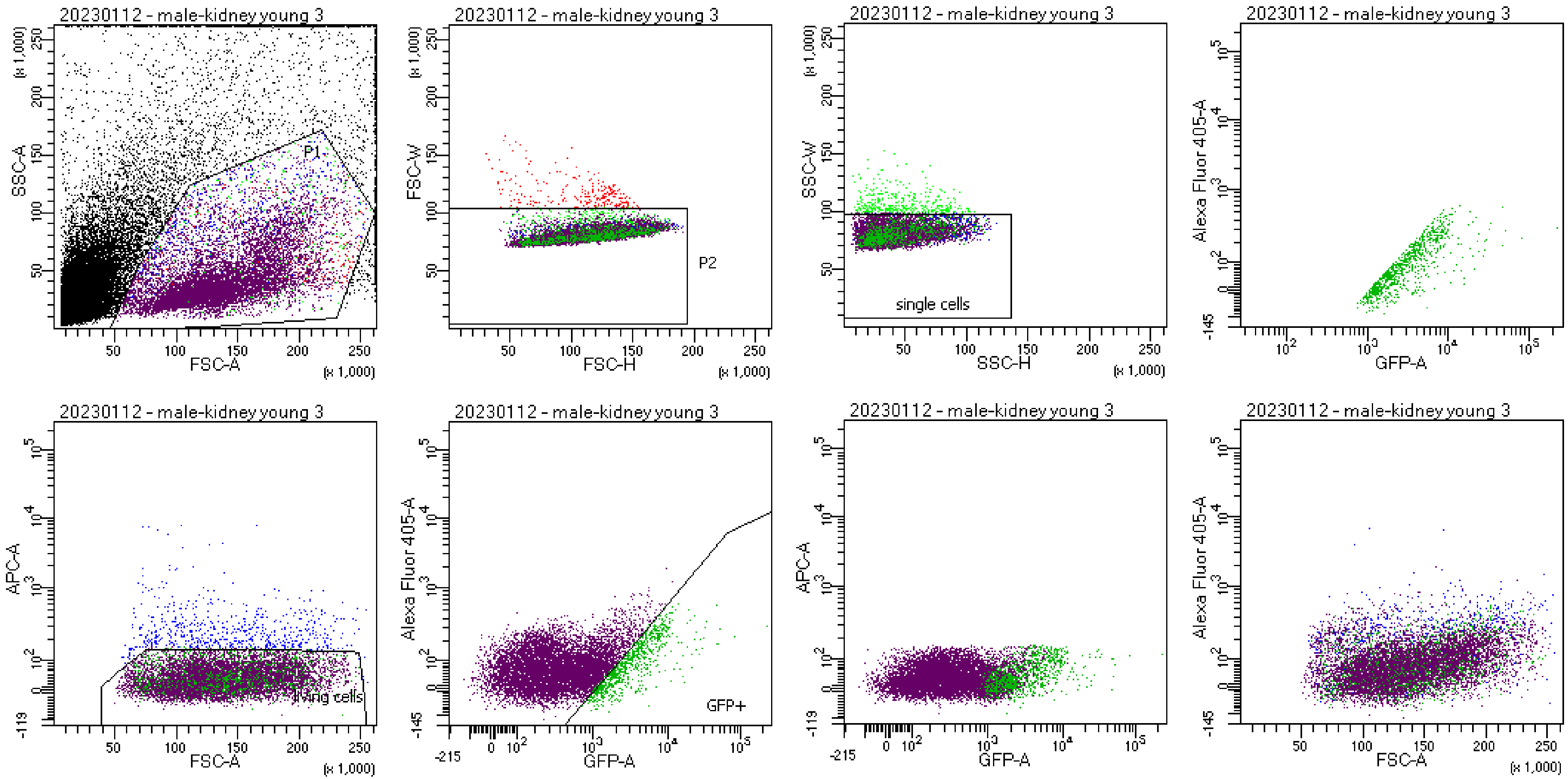

Tube: kidney young 3

Population	#Events	%Parent	%Total
All Events	40,830	####	100.0
P1	11,250	27.6	27.6
P2	11,011	97.9	27.0
single cells	10,608	96.3	26.0
living cells	10,000	94.3	24.5
GFP+	834	8.3	2.0

BD FACSDiva 9.0.1

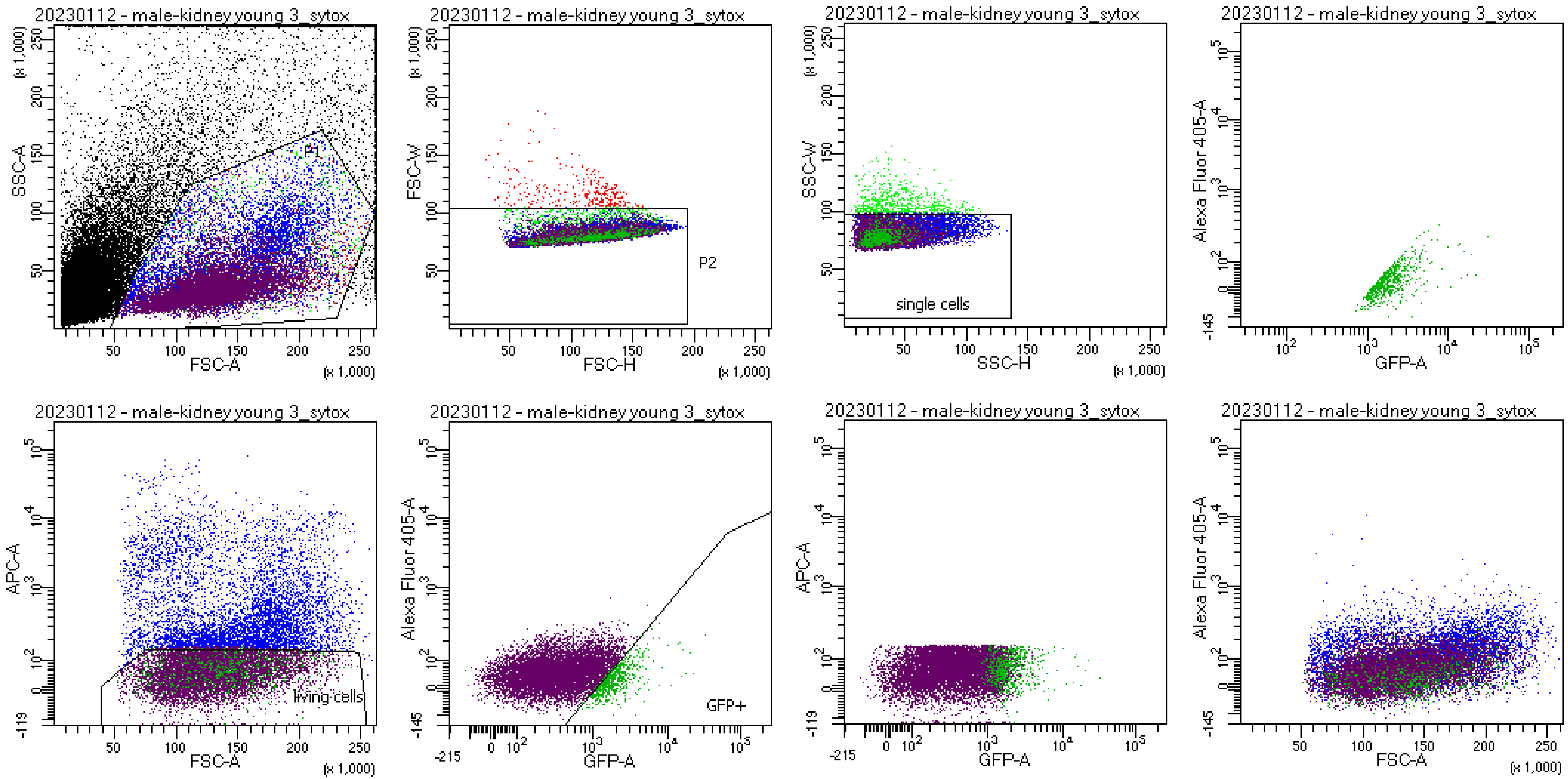

Tube: kidney young 3_sytox

Population	#Events	%Parent	%Total
All Events	61,096	####	100.0
P1	16,762	27.4	27.4
P2	16,438	98.1	26.9
single cells	15,817	96.2	25.9
living cells	10,000	63.2	16.4
GFP+	635	6.4	1.0

BD FACSDiva 9.0.1

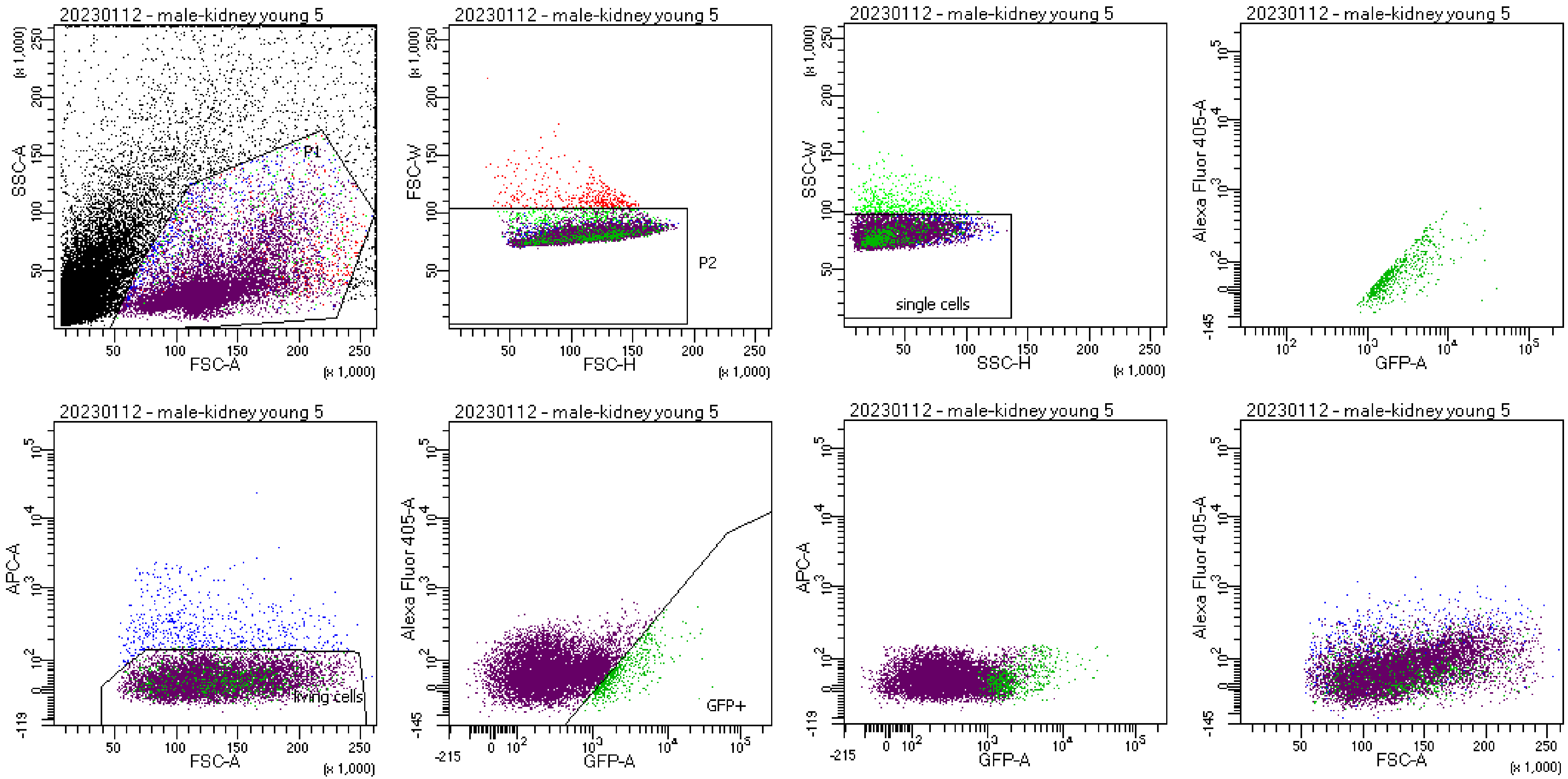

Tube: kidney young 5

Population	#Events	%Parent	%Total
All Events	42,323	####	100.0
P1	11,424	27.0	27.0
P2	11,028	96.5	26.1
single cells	10,586	96.0	25.0
living cells	10,000	94.5	23.6
GFP+	571	5.7	1.3

BD FACSDiva 9.0.1

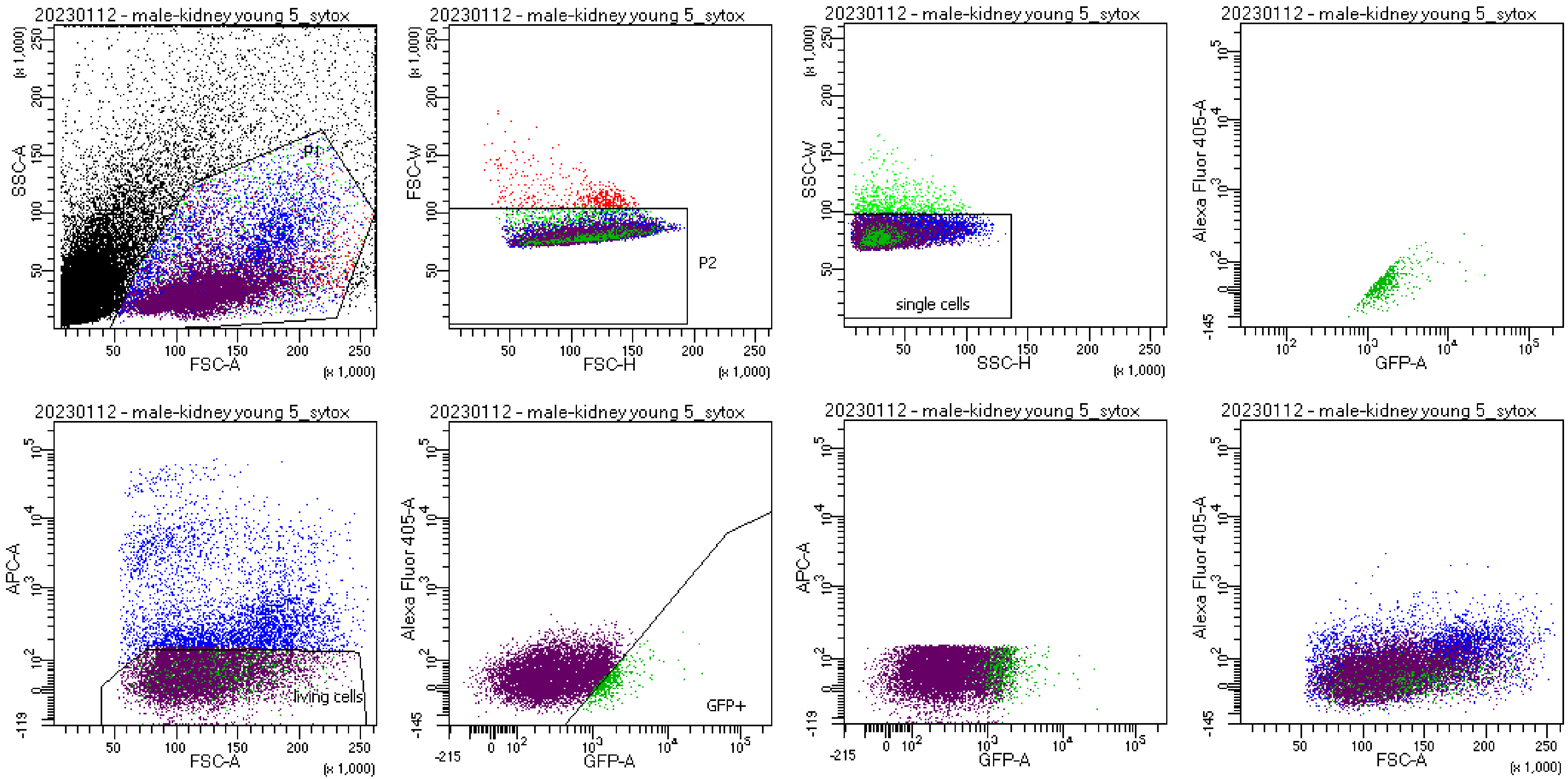

Tube: kidney young 5_sytox

Population	#Events	%Parent	%Total
All Events	58,062	####	100.0
P1	15,480	26.7	26.7
P2	15,002	96.9	25.8
single cells	14,340	95.6	24.7
living cells	10,000	69.7	17.2
GFP+	479	4.8	0.8

BD FACSDiva 9.0.1

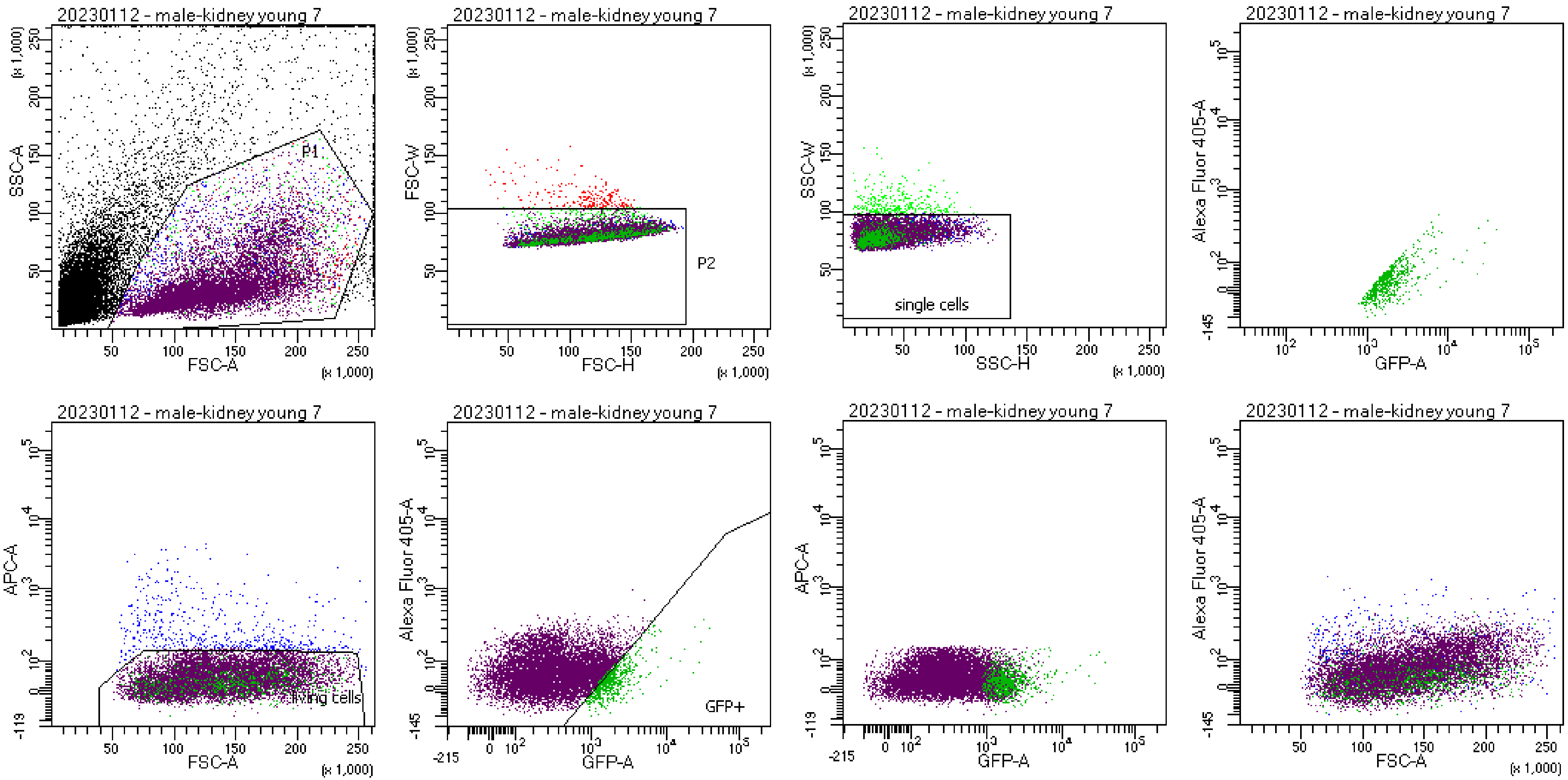

Tube: kidney young 7

Population	#Events	%Parent	%Total
All Events	30,663	####	100.0
P1	11,081	36.1	36.1
P2	10,860	98.0	35.4
single cells	10,534	97.0	34.4
living cells	10,000	94.9	32.6
GFP+	686	6.9	2.2

BD FACSDiva 9.0.1

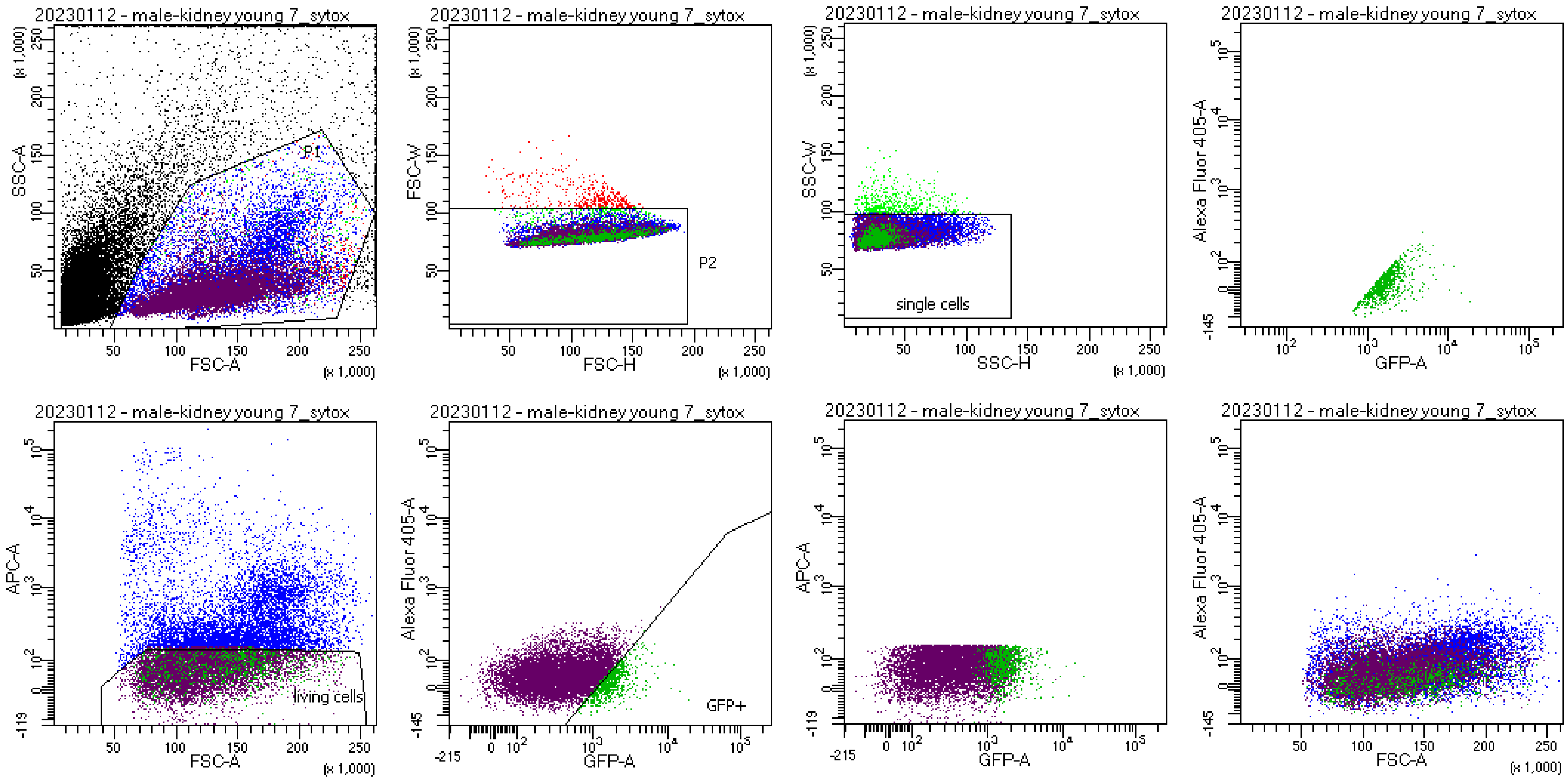

Tube: kidney young 7_sytox

Population	#Events	%Parent	%Total
All Events	52,491	####	100.0
P1	18,389	35.0	35.0
P2	17,966	97.7	34.2
single cells	17,453	97.1	33.2
living cells	10,000	57.3	19.1
GFP+	879	8.8	1.7

BD FACSDiva 9.0.1

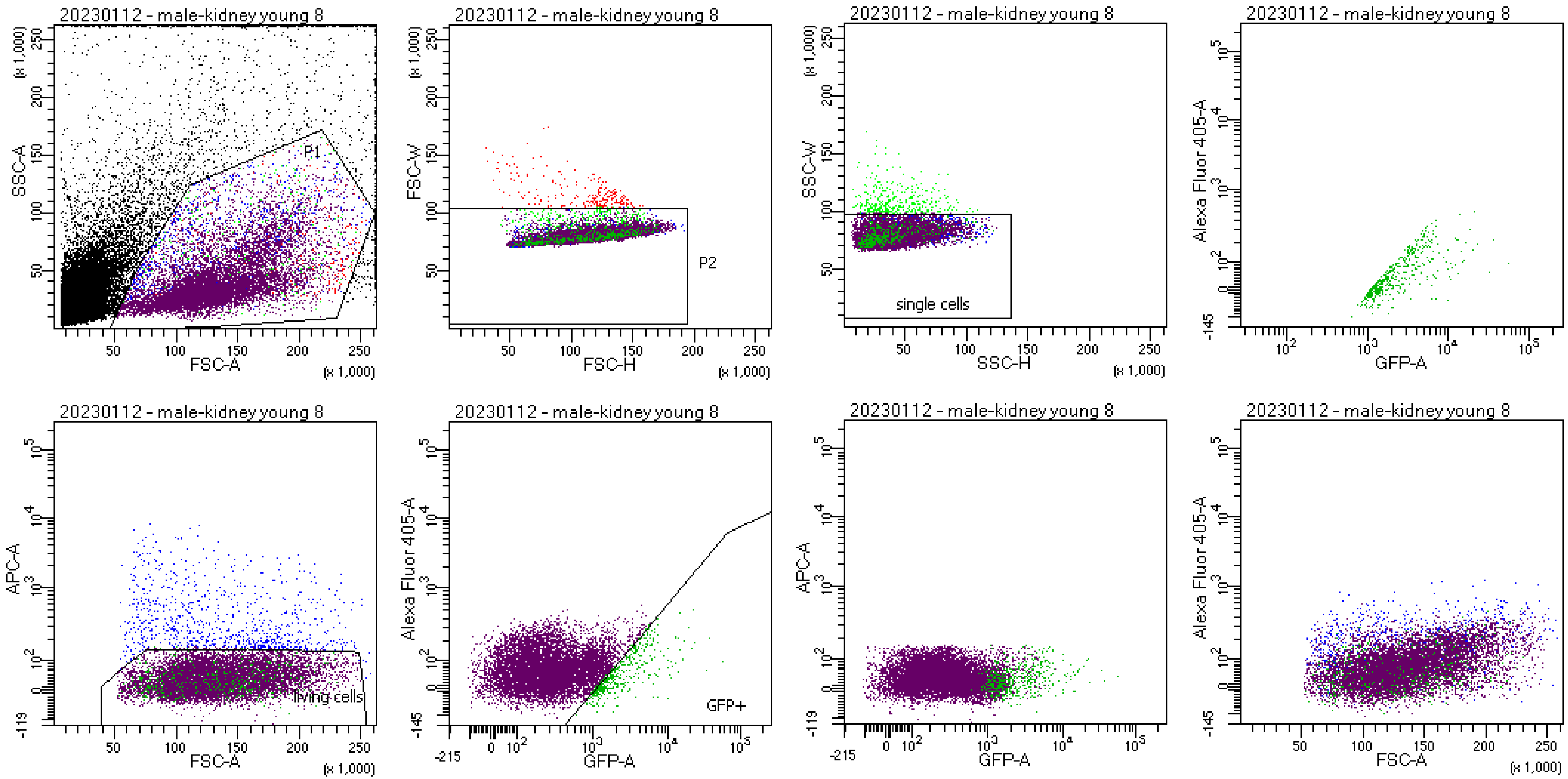

Tube: kidney young 8

Population	#Events	%Parent	%Total
All Events	38,263	####	100.0
P1	11,377	29.7	29.7
P2	11,146	98.0	29.1
single cells	10,793	96.8	28.2
living cells	10,000	92.7	26.1
GFP+	426	4.3	1.1

BD FACSDiva 9.0.1

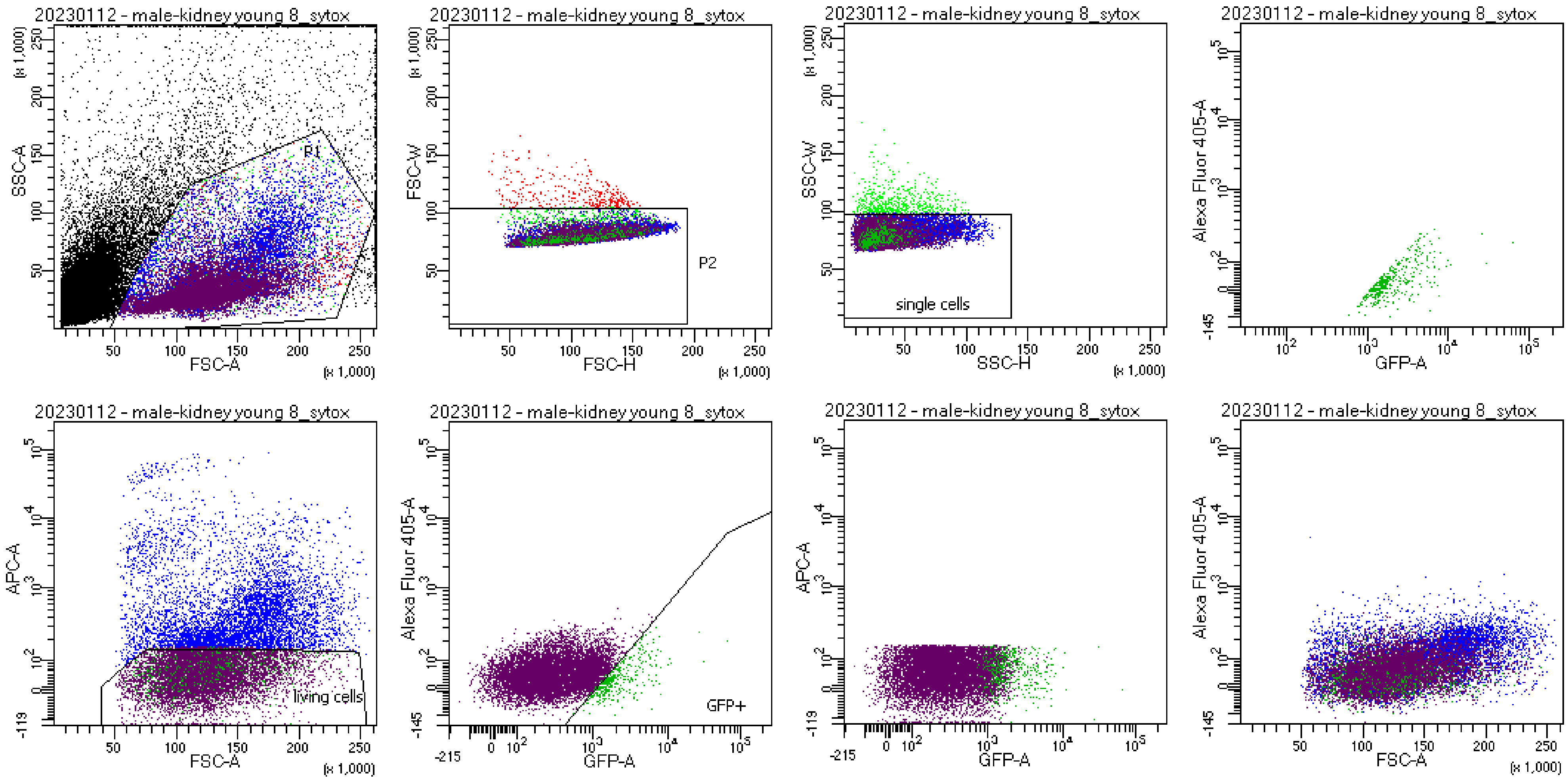

Tube: kidney young 8_sytox

Population	#Events	%Parent	%Total
All Events	58,074	####	100.0
P1	17,067	29.4	29.4
P2	16,732	98.0	28.8
single cells	16,167	96.6	27.8
living cells	10,000	61.9	17.2
GFP+	409	4.1	0.7

BD FACSDiva 9.0.1

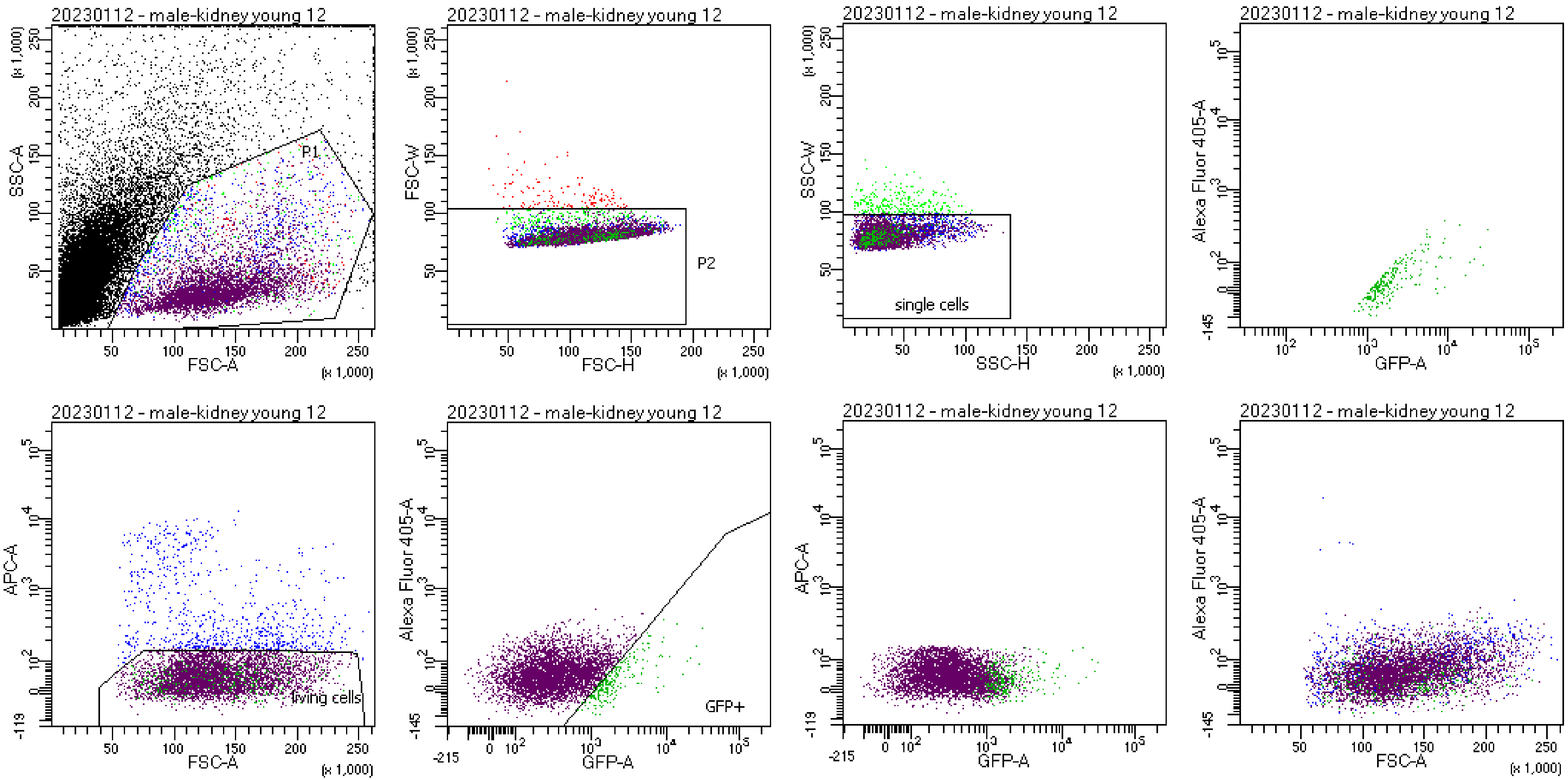

Tube: kidney young 12

Population	#Events	%Parent	%Total
All Events	58,459	####	100.0
P1	6,181	10.6	10.6
P2	6,048	97.8	10.3
single cells	5,773	95.5	9.9
living cells	5,003	86.7	8.6
GFP+	256	5.1	0.4

BD FACSDiva 9.0.1

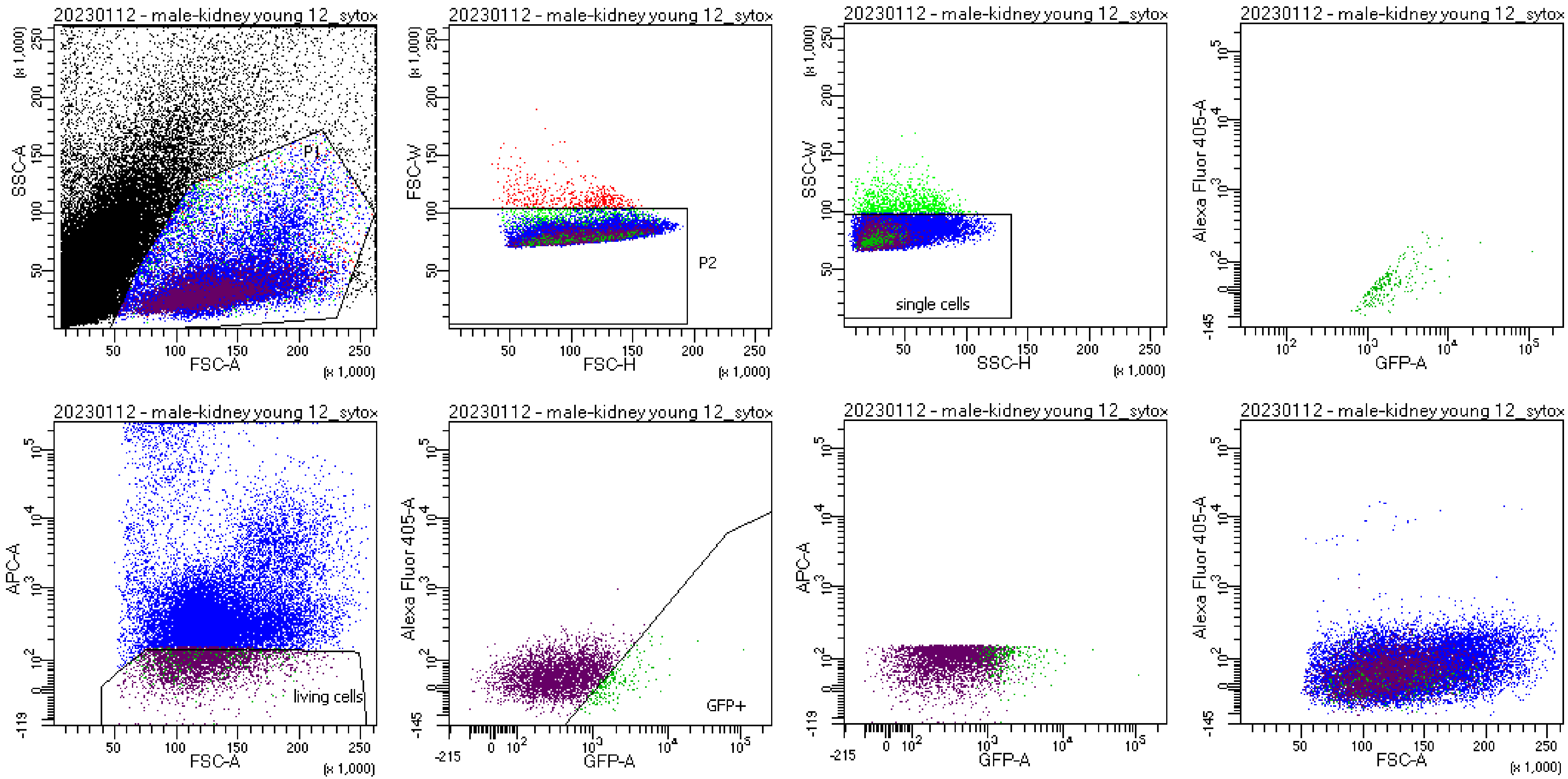

Tube: kidney young 12_sytox

Population	#Events	%Parent	%Total
All Events	210,179	####	100.0
P1	23,356	11.1	11.1
P2	22,885	98.0	10.9
single cells	21,899	95.7	10.4
living cells	4,072	18.6	1.9
GFP+	220	5.4	0.1
