# Supplementary material for: Generation of a transparent killifish line through multiplex CRISPR/Cas9mediated gene inactivation
Source: eLife. 2023 Feb 23;12:e81549. doi: 10.7554/eLife.81549 (PMC10010688; doi:10.7554/eLife.81549)
Supplement: Figure 5—figure supplement 1—source data 1. [file elife-81549-fig5-figsupp1-data1.zip › Figure_5_figure_supplement_1_source_data/Figure_5_figure_supplement_1_panel_A_source_data/Originals_Sequencing_data/cdkn1a_10/p21_#10_F.pdf]

Clip. 1 BQ 20 WL 10 Sequence: p21_#10_F

Clipped length:	1206	Samples:	18935	Quality: 0 - 9	
Left clip:	17	Bases:	1469	10 - 19	
Right clip:	1222	Average spacing:	13.0	20 - 29	
Avg. qual. in clip.:	47.47	Average quality >=	10: 135, 20: 159, 30: 1035	>= 30	

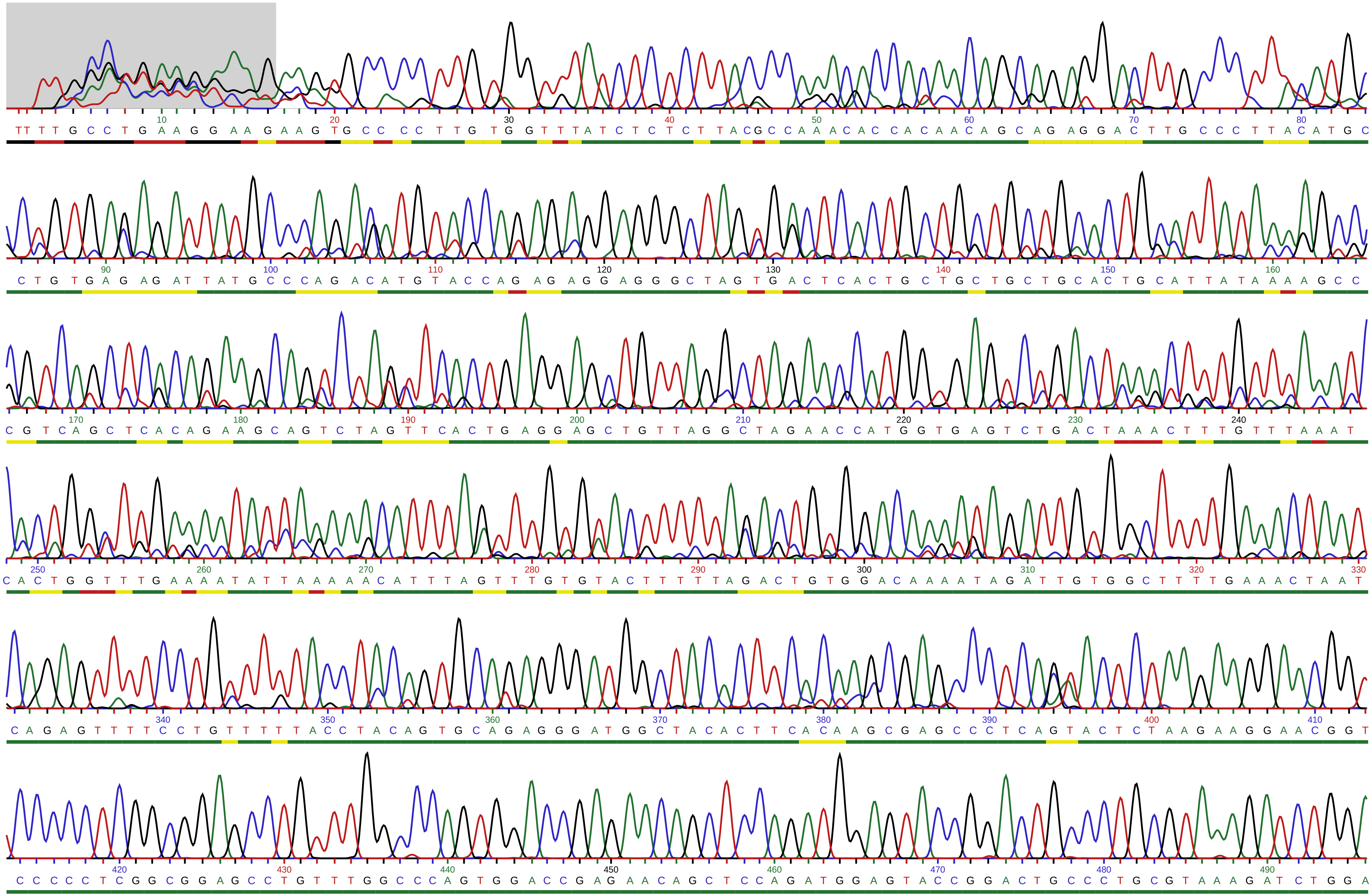

Clip. 1 BQ 20 WL 10 Sequence: p21_#10_F

Clipped length: 1206
Left clip: 17
Right clip: 1222
Avg. qual. in clip.: 47.47

Samples: 18935
Bases: 1469
Average spacing: 13.0
Average quality >= 10: 135, 20: 159, 30: 1035

Quality: 0 - 9, 10 - 19, 20 - 29, >= 30

06.08.2020

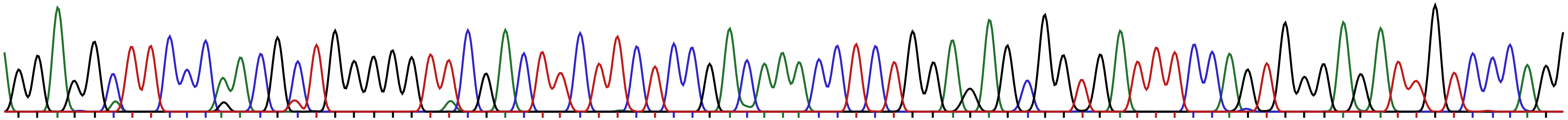

500 510 520 530 540 550 560 570

G G A G G C T T C C C A A C G C T G G G G G T T C G A C T T C T T C T C C G A C A A A C C T C T G G A G A G C G G T G A T T T C C A G T G G G A G A T T G T C C C A G

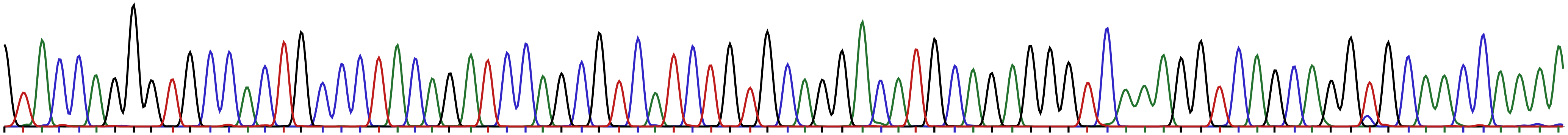

580 590 600 610 620 630 640 650 660

G T A C C A G G G T G C C A C T G C C C T A C A G A T C C A G C G T C A T C T G T G C A G G A C A T G C A G A G G G T C A A A G G T C A G C A G G T G C A A C C A A A A

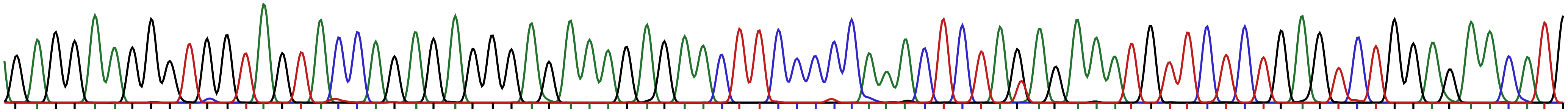

670 680 690 700 710 720 730 740

G A G G A A G G G T G G T A G T A C C A G A G A G G G A G A A A G A G A A C T T C C C C C A A A C T C T A G A G A A A T G T T C T C T G A G T C T G G A G A A C A T G

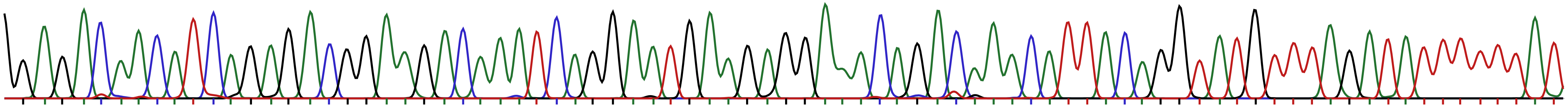

750 760 770 780 790 800 810 820

G A G A C A A C A T C A G G A G A C G G A A G A C A A A T C A G G A A T G A A G A G G A A A C A G A C A A A C A T T A C A G G T A T G T T T A G A T A T T T T T A T

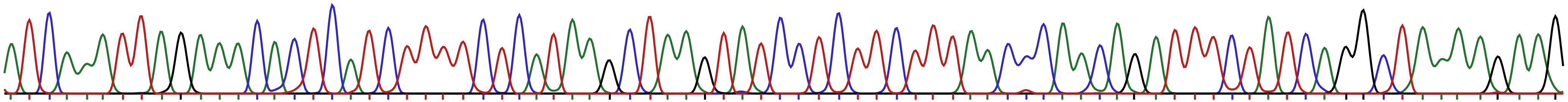

830 840 850 860 870 880 890 900 910

A T C A A A T T A G A A A C A C T C A T C T T T T C T C A T A A G C T A A G T A T C C T C T T C T T T A A C C C A A C A G A T T T C T A T C A G G C T A A A A G A A G

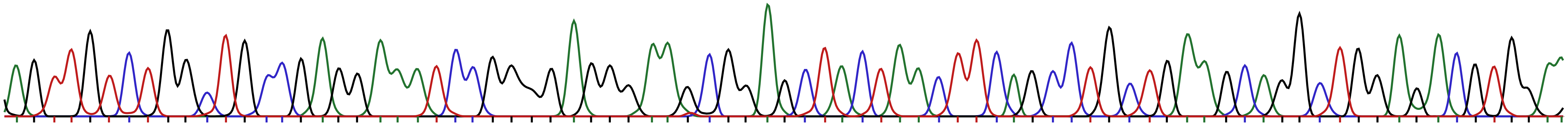

920 930 940 950 960 970 980 990

A G T T G T C T G G C T G C C G A G G A A A T C C G G G G A G G G A A G C G G A G C T A C T A A C T T C A G C C T G C T G A A G C A G G C T G G A G A C G T G G A A

Clip. 1   BQ 20 WL 10   Sequence: p21_#10_F

Clipped length:	1206	Samples:	18935	Quality:	0 - 9
Left clip:	17	Bases:	1469		10 - 19
Right clip:	1222	Average spacing:	13.0		20 - 29
Avg. qual. in clip.:	47.47	Average quality >=	10: 135, 20: 159, 30: 1035		>= 30

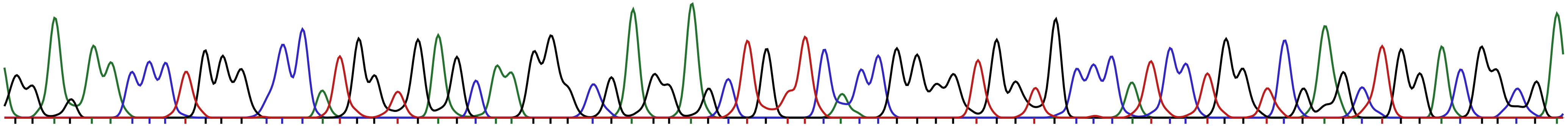

1000 1010 1020 1030 1040 1050 1060 1070

G G A G A A C C C T G G G C C C A T G G T G A G C A A G G G C G A G G A G C T G T T C A C C G G G G T G G T G C C C A T C C T G G T C G A G C T G G A C G G C G A

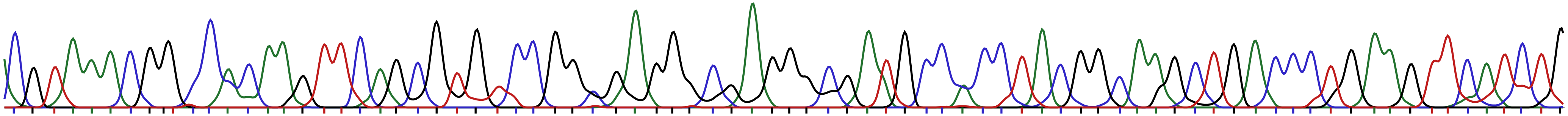

1080 1090 1100 1110 1120 1130 1140 1150

C G T A A A C G G T C C A C A A G T T C A G C G T G T C C G G C G A G G G C G A G G G C G A T G C C A C C T A C G G C A A G C T G A C C C T G A A G T T C A T C T G

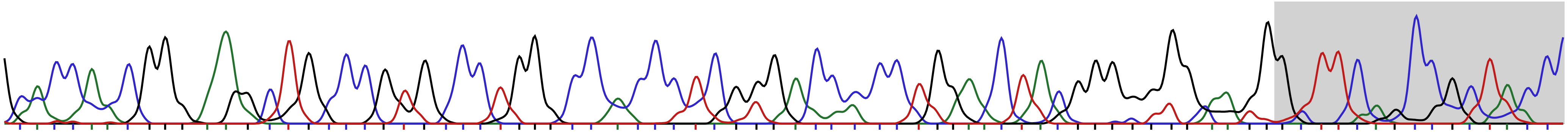

1160 1170 1180 1190 1200 1210 1220 1230

C A C C A A C G G G A A G C T G C C C G T G C C C T G G G C C A C C C T C G G G A C C C C T G G A A C T A C G G G G G G G A A G G G C T T C A G C C G C T A C C

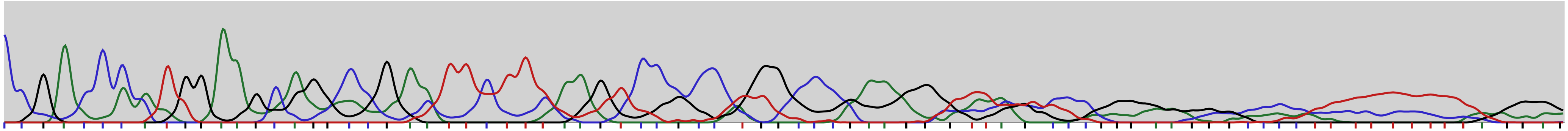

1240 1250 1260 1270 1280 1290 1300 1310 1320

C C G A C C C A T G G A A G C A G G C C G A A T T C T T T A A G T C C G C C T G G C C C G A A G G G T T A G C C C G G G A A G G G C C C C T T T T T T T T T A G G A G

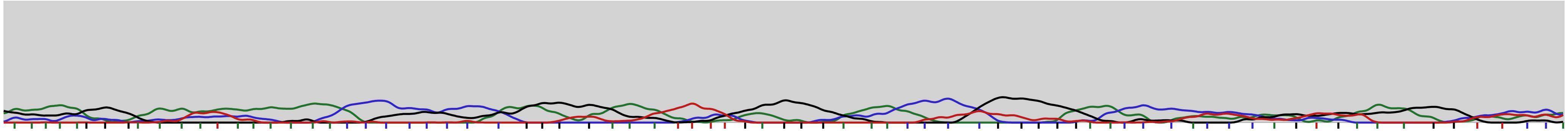

1330 1340 1350 1360 1370 1380 1390

A A A A A G G G A A A A T A A A A A A C C C C C C C C G G G G A A A T T T T G A G G G A A A C C C C G G G G A A C C C A C C C A G T G A A A G G T T C C A

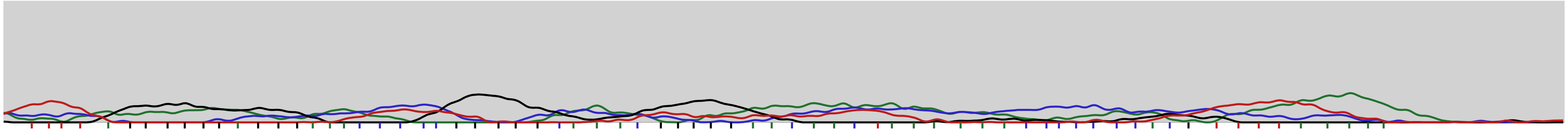

1400 1410 1420 1430 1440 1450 1460

T T T T A G G G G G G A G G G A A A C C C C C G G G G G C A A A C G G G G G A A C A A C T A A A A A A C C C C C C C A C G T T T T A A A A A

Clip. 1 BQ 20 WL 10 Sequence: p21_#10_F

Clipped length:	1206	Samples:	18935	Quality:	0 - 9
Left clip:	17	Bases:	1469		10 - 19
Right clip:	1222	Average spacing:	13.0		20 - 29
Avg. qual. in clip.:	47.47	Average quality >=	10: 135, 20: 159, 30: 1035		>= 30
