# Supplementary material for: Generation of a transparent killifish line through multiplex CRISPR/Cas9mediated gene inactivation
Source: eLife. 2023 Feb 23;12:e81549. doi: 10.7554/eLife.81549 (PMC10010688; doi:10.7554/eLife.81549)
Supplement: Figure 5—figure supplement 1—source data 1. [file elife-81549-fig5-figsupp1-data1.zip › Figure_5_figure_supplement_1_source_data/Figure_5_figure_supplement_1_panel_A_source_data/Originals_Sequencing_data/cdkn1a_sequencing.docx]

***cdkn1a* sequencing**

*>cdkn1a* donor template sequence

TCTTACACCAAACACCACAACAGCAGAGGACTTGCCCTTACATGCCTGTGAGAGATTATGCCCAGACATGTACCAGAGAGGAGGGCTAGTGACTCACTGCTGCTGCTGCACTGCATTATAAAAGCCCGTCAGCTCACAGAAGCAGTCTAGTTCACTGAGGAGCTGTTAGGCTAGAACCATGGTGAGTCTGACTAAACTTTGTTTAAATCACTGGTTTGAAAATATTAAAAACATTTAGTTTGTGTACTTTTTAGACTGTGGACAAAATAGATTGTGGCTTTTGAAACTAATCAGAGTTTTCCTGTTTTTACCTACAGTGCAGAGGGATGGCTACACTTCACAAGCGAGCCCTCAGTACTCTAAGAAGGAACGGTCCCCCTCGGCGGAGCCTGTTTGGCCCAGTGGACCGAGAACAGCTCCAGATGGAGTACCGGACTGCCCTGCGTAAAGATCTGGAGGAGGCTTCCCAACGCTGGGGGTTCGACTTCTTCTCCGACAAACCTCTGGAGAGCGGTGATTTCCAGTGGGAGATTGTCCCAGGTACCAGGGTGCCACTGCCCTACAGATCCAGCGTCATCTGTGCAGGACATGCAGAGGGTCAAAGGTCAGCAGGTGCAACCAAAAGAGGAAGGGTGGTAGTACCAGAGAGGGAGAAAGAGAACTTCCCCCAAACTCTAGAGAAATGTTCTCTGAGTCTGGAGAACATGGAGACAACATCAGAGACGGAAGACAAATCAGGAATGAAGAGGAAACAGACAAACATTACAGGTATGTTTAGATATTTTTTATATCAAATTAGAAACACTCATCTTTTCTCATAAGCTAAGTATCCTCTTCTTTAACCCAACAGATTTCTATCAGGCTAAAAGAAGAGTTGTCTGGCTGCCGAGGAAATCCGGGGAGGGAAGCGGAGCTACTAACTTCAGCCTGCTGAAGCAGGCTGGAGACGTGGAGGAGAACCCTGGGCCCATGGTGAGCAAGGGCGAGGAGCTGTTCACCGGGGTGGTGCCCATCCTGGTCGAGCTGGACGGCGACGTAAACGGCCACAAGTTCAGCGTGTCCGGCGAGGGCGAGGGCGATGCCACCTACGGCAAGCTGACCCTGAAGTTCATCTGCACCACCGGCAAGCTGCCCGTGCCCTGGCCCACCCTCGTGACCACCCTGACCTACGGCGTGCAGTGCTTCAGCCGCTACCCCGACCACATGAAGCAGCACGACTTCTTCAAGTCCGCCATGCCCGAAGGCTACGTCCAGGAGCGCACCATCTTCTTCAAGGACGACGGCAACTACAAGACCCGCGCCGAGGTGAAGTTCGAGGGCGACACCCTGGTGAACCGCATCGAGCTGAAGGGCATCGACTTCAAGGAGGACGGCAACATCCTGGGGCACAAGCTGGAGTACAACTACAACAGCCACAACGTCTATATCATGGCCGACAAGCAGAAGAACGGCATCAAGGTGAACTTCAAGATCCGCCACAACATCGAGGACGGCAGCGTGCAGCTCGCCGACCACTACCAGCAGAACACCCCCATCGGCGACGGCCCCGTGCTGCTGCCCGACAACCACTACCTGAGCACCCAGTCCGCCCTGAGCAAAGACCCCAACGAGAAGCGCGATCACATGGTCCTGCTGGAGTTCGTGACCGCCGCCGGGATCACTCTCGGCATGGACGAGCTGTACAAGGGAAGCGGAGCTACTAACTTCAGCCTGCTGAAGCAGGCTGGAGACGTGGAGGAGAACCCTGGGCCCATGGACATCATCTCTGTGGCTCTGAAGAGACATAGCACAAAGGCTTTTGATGCCAGCAAGAAACTGACCCCTGAGCAGGCTGAGCAGATCAAGACACTGCTGCAGTACAGCCCAAGCAGCcagAACAGCCAGCCTTGGCATTTTATTGTGGCTTCTACAGAGGAGGGAAAGGCTAGAGTGGCTAAGTCTGCTGCTGGAAATTATGTGTTCtctGAGAGAAAGATGCTGGATGCCTCTCATGTGGTGGTGTTCTGTGCTAAGACAGCCATGGATGATGTGTGGCTGAAACTGGTGGTGGACCAGGAGGATGCTGATGGAAGATTTGCCACACCTGAGGCTAAGGCTGCTAATGATAAGGGAAGAAAGTTCacaGCTGATATGCACAGAAAGGACCTGCATGATGATGCTGAGTGGATGGCTAAGCAGGTGTATCTGAATGTGGGAAACTTCCTGCTGGGAGTGGCTGCTCTGGGACTGGATGCTGTGCCTATTGAGGGATTTGATGCTGCTATCCTGGATGCTGAGTTTGGACTGAAGGAGAAGGGATACACCAGCCTGGTGGTGGTGCCAGTGGGACACCACTCTGTGGAGGATTTTAATGCTACACTGCCTAAGAGCAGACTGCCTCAGAACATCACCCTGACAGAGGTGTAATATTTCCTTTGAAGGTGCCAAAACTCCCAAGAGAACCAGACAAAGAACCAGCACCTCCTTCAGGAATCAGTTGTTCCACACCGAGCAGCGGGAGTGCTGAATCATCCAACAAAAAAAAAACAAAAAAAAGAAAAAAAAAACCCAGAAGGCACCAGGCTAATTAAGATGCTGCATGTGTGAGGGGTCTGGTGTTTTCTGTCCACGCTGCAGTTAAGAAGGTGACATTTGAGCAGAGAAGATTAACAGTAATCTCTGGAATCAGTGGATTTATACTCATTTGGACTGAGAGCTCGCCAAATTGCTGACTGACATTTGTTACGGCGCGTCACACAGCTCTTAACCCTCTCGGTCTCGTTTTGATAAAAGGACGAACAACTAAGTCCTTCAGGGGTGCTTCTGAGTTAAAAAATGCTCATGAGATACTTGTGTGGAGTAACCAGGTAGGTAGGTTTTAACTGTTGCAGATCTGCGATGCCTGCCTCCAGAGGGTTAAAACACAATCACATCTCAGTTTGGTGGGACTTCTGCCTGTTTGAGGATGAAAAGCAGACGTGCGAACAAATATCAGGACTCAGATTAATACGTTTCTGTGGCAAAGCCGCAGGAAAGCTAAAAACAGGAGAGCTTTGTCTATTTTGTTCAAATCTATAATTGTTTTTCTACGTCTTTTTATAGGAGTTTTTTTTAAGTGCATTATATTTTACATCGGGGGTTCCCAATCCTGGCCCTGGAGGGCTGGTATCCAGCAGGTTTTGTGGTTTTTCTGCTCCAACAGACTTGATTCAGTGGTTGAAACGCCTGTGCAGCAGCTCATCAGGCTCTGCAGAAGCCTGTTAATGAGCTGCTGATTGAAATCAGGTGTGTTGAAACAGAATTCAAACTAAAACATGCAGGATACCGG

>GFP-positive_#10

AAGTGCCCCTTGTGGTTTATCTCTCTTACGCCAAACACCACAACAGCAGAGGACTTGCCCTTACATGCCTGTGAGAGATTATGCCCAGACATGTACCAGAGAGGAGGGCTAGTGACTCACTGCTGCTGCTGCACTGCATTATAAAAGCCCGTCAGCTCACAGAAGCAGTCTAGTTCACTGAGGAGCTGTTAGGCTAGAACCATGGTGAGTCTGACTAAACTTTGTTTAAATCACTGGTTTGAAAATATTAAAAACATTTAGTTTGTGTACTTTTTAGACTGTGGACAAAATAGATTGTGGCTTTTGAAACTAATCAGAGTTTTCCTGTTTTTACCTACAGTGCAGAGGGATGGCTACACTTCACAAGCGAGCCCTCAGTACTCTAAGAAGGAACGGTCCCCCTCGGCGGAGCCTGTTTGGCCCAGTGGACCGAGAACAGCTCCAGATGGAGTACCGGACTGCCCTGCGTAAAGATCTGGAGGAGGCTTCCCAACGCTGGGGGTTCGACTTCTTCTCCGACAAACCTCTGGAGAGCGGTGATTTCCAGTGGGAGATTGTCCCAGGTACCAGGGTGCCACTGCCCTACAGATCCAGCGTCATCTGTGCAGGACATGCAGAGGGTCAAAGGTCAGCAGGTGCAACCAAAAGAGGAAGGGTGGTAGTACCAGAGAGGGAGAAAGAGAACTTCCCCCAAACTCTAGAGAAATGTTCTCTGAGTCTGGAGAACATGGAGACAACATCAGAGACGGAAGACAAATCAGGAATGAAGAGGAAACAGACAAACATTACAGGTATGTTTAGATATTTTTTATATCAAATTAGAAACACTCATCTTTTCTCATAAGCTAAGTATCCTCTTCTTTAACCCAACAGATTTCTATCAGGCTAAAAGAAGAGTTGTCTGGCTGCCGAGGAAATCCGGGGAGGGAAGCGGAGCTACTAACTTCAGCCTGCTGAAGCAGGCTGGAGACGTGGAAGGAGAACCCTGGGCCCATGGTGAGCAAGGGCGAGGAGCTGTTCACCGGGGTGGTGCCCATCCTGGTCGAGCTGGACGGCGACGTAAACGGTCCACAAGTTCAGCGTGTCCGGCGAGGGCGAGGGCGATGCCACCTACGGCAAGCTGACCCTGAAGTTCATCTGCACCAACGGGAAGCTGCCCGTGCCCTGGGCCACCCTCGGGACCCCCTGGAACTACGGGGGGGAAGG
