# Supplementary figures and images for: Generation of a transparent killifish line through multiplex CRISPR/Cas9mediated gene inactivation (part 2 of 2)
Source: eLife. 2023 Feb 23;12:e81549. doi: 10.7554/eLife.81549 (PMC10010688; doi:10.7554/eLife.81549)

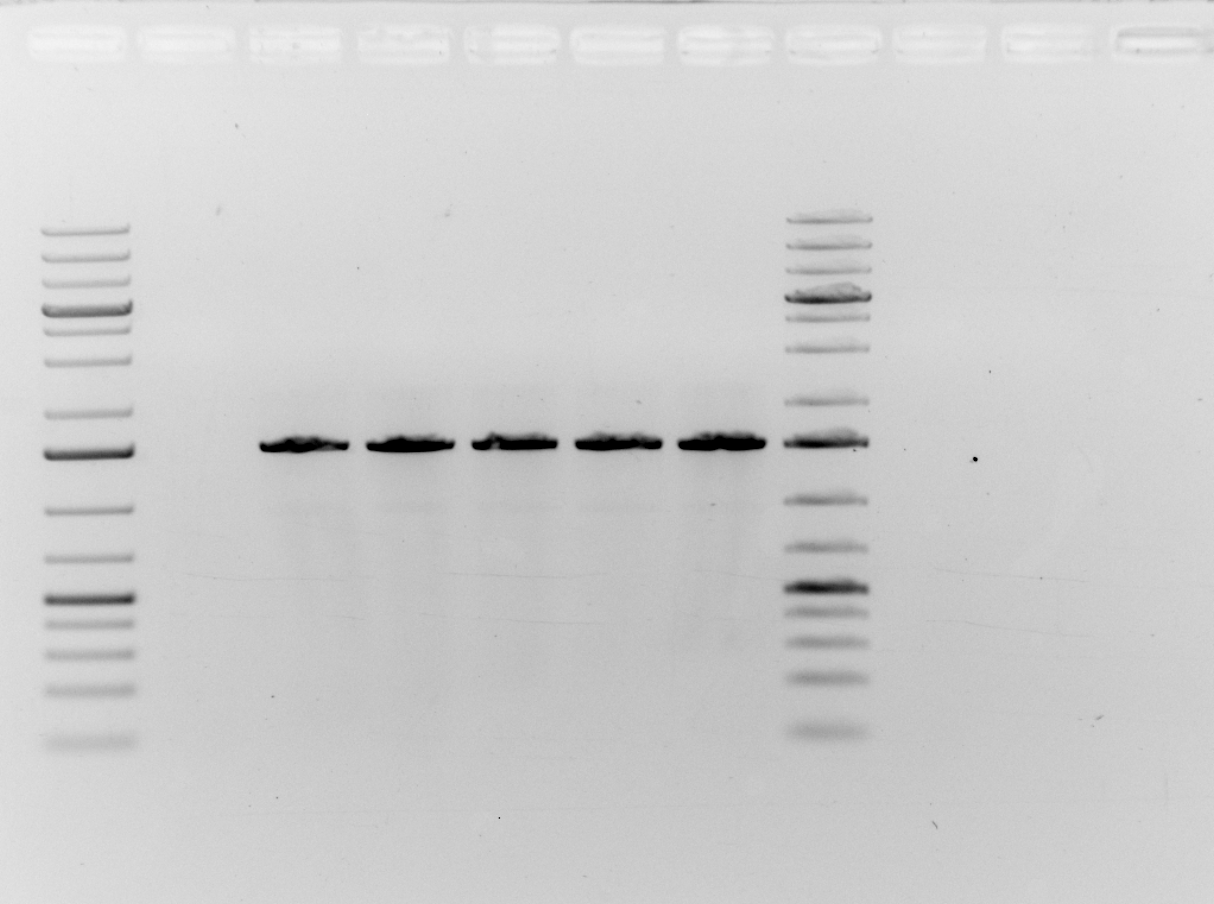

Supplement: Figure 5—figure supplement 1—source data 1. [file elife-81549-fig5-figsupp1-data1.zip › Figure_5_figure_supplement_1_source_data/Figure_5_figure_supplement_1_panel_B_source_data/Figure_5_figure_supplement_1_panel_b(1)_source_data.tif]

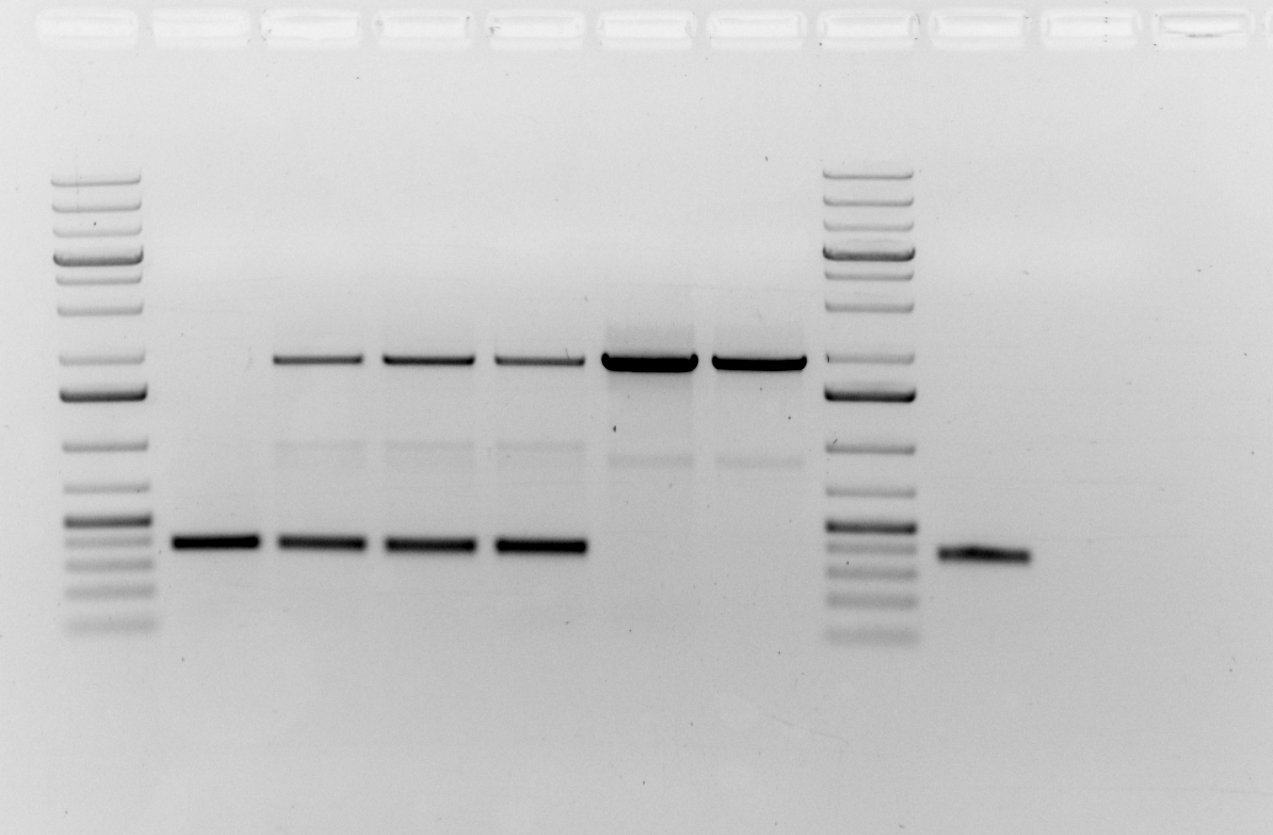

Supplement: Figure 5—figure supplement 1—source data 1. [file elife-81549-fig5-figsupp1-data1.zip › Figure_5_figure_supplement_1_source_data/Figure_5_figure_supplement_1_panel_B_source_data/Figure_5_figure_supplement_1_panel_b(2)_source_data.tif]

## Supplement Figure 5

panel b

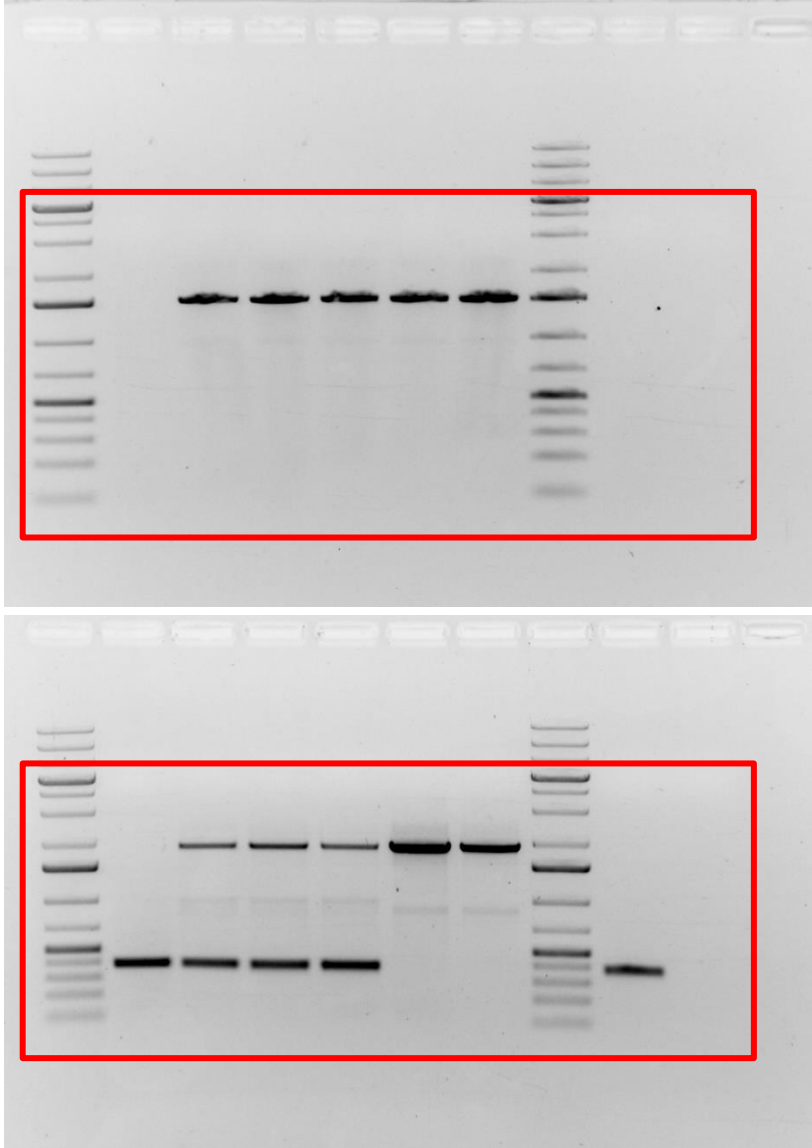

The red box surrounds the lanes shown in the respective panel.

Supplement: Figure 5—figure supplement 1—source data 1. [file elife-81549-fig5-figsupp1-data1.zip › Figure_5_figure_supplement_1_source_data/Figure_5_figure_supplement_1_panel_B_source_data/Figure_5_figure_supplement_1_panel_b_source_data.pdf]

## Slide 1
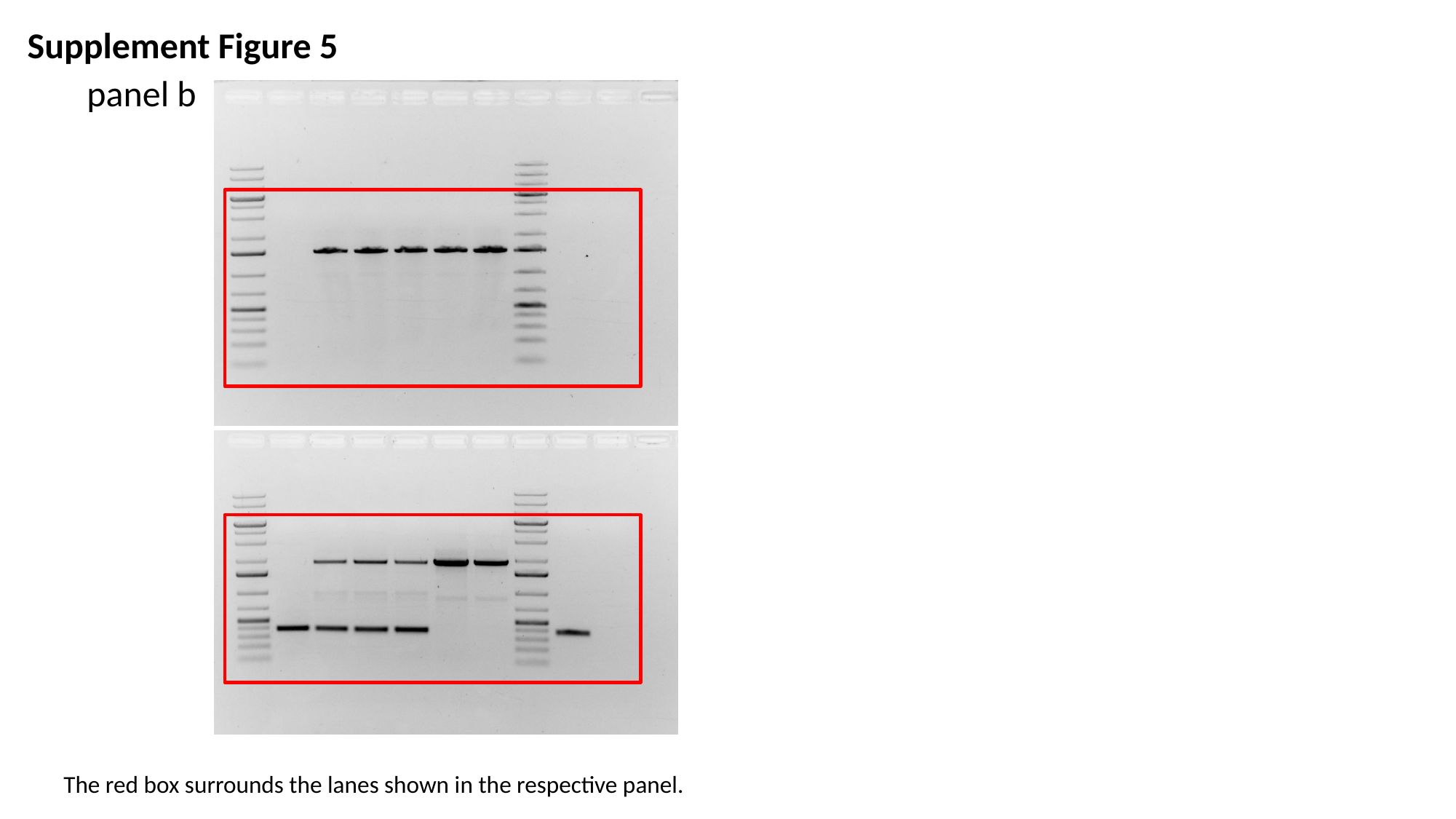

Supplement Figure 5
panel b
The red box surrounds the lanes shown in the respective panel.

Supplement: Figure 5—figure supplement 1—source data 1. [file elife-81549-fig5-figsupp1-data1.zip › Figure_5_figure_supplement_1_source_data/Figure_5_figure_supplement_1_panel_B_source_data/Figure_5_figure_supplement_1_panel_b_source_data.pptx]
