# Supplementary material for: Generation of a transparent killifish line through multiplex CRISPR/Cas9mediated gene inactivation
Source: eLife. 2023 Feb 23;12:e81549. doi: 10.7554/eLife.81549 (PMC10010688; doi:10.7554/eLife.81549)
Supplement: MDAR checklist [file elife-81549-mdarchecklist1.docx]

**Materials Design Analysis Reporting (MDAR)**

**Checklist for Authors**

The [MDAR framework](https://osf.io/xfpn4/) establishes a minimum set of requirements in transparent reporting mainly applicable to studies in the life sciences.

*eLife* asks authors to **provide detailed information within their article** to facilitate the interpretation and replication of their work. Authors can also upload supporting materials to comply with relevant reporting guidelines for health-related research (see [EQUATOR Network](http://www.equator-network.org/%20)), life science research (see the [BioSharing Information Resource](http://biosharing.org/)), or animal research (see the [ARRIVE Guidelines](http://www.plosbiology.org/article/info:doi/10.1371/journal.pbio.1000412) and the [STRANGE Framework](https://doi.org/10.1038/d41586-020-01751-5); for details, see *eLife*’s [Journal Policies](https://reviewer.elifesciences.org/author-guide/journal-policies)). Where applicable, authors should refer to any relevant reporting standards materials in this form.

For all that apply, please note **where in the article** the information is provided. Please note that we also collect information about data availability and ethics in the submission form.

**Materials:**

| **Newly created materials** | **Indicate where provided: section/figure legend** | **N/A** |
| --- | --- | --- |
| The manuscript includes a dedicated "materials availability statement" providing transparent disclosure about availability of newly created materials including details on how materials can be accessed and describing any restrictions on access. | - Plasmid containing the *cdkn1a-P2A-eGFP-P2A-NTR* cassette for HDR-mediated integration into the *N. furzeri* genome (Fig. 5B). - *klara* fish line (Fig. 1M-M’).   Materials can be accessed upon request. |  |
|  |  |  |
| **Antibodies** | **Indicate where provided: section/figure legend** | **N/A** |
| For commercial reagents, provide supplier name, catalogue number and [RRID](https://scicrunch.org/resources), if available.   - a-GFP antibody (Thermo Fisher Scientific Inc., United States: A-11122, rabbit) - anti-rabbit Alexa Fluor® 546 antibody (Thermo Fisher Scientific Inc., United States: A-11071, goat) | Fig. 5H  Fig. 5H |  |
|  |  |  |
| **DNA and RNA sequences** | **Indicate where provided: section/figure legend** | **N/A** |
| Short novel DNA or RNA including primers, probes: Sequences should be included or deposited in a public repository.  sg_mitfa_1: 5’-TAGGAACATCAAAAGGGAATTCAC-3’  sg_mitfa_2: 5’-AAACGTGAATTCCCTTTTGATGTT-3’,  sg_ltk_1: 5’- TAGGAACATCAAAAGGGAATTCAC-3’  sg_ltk_2: 5’-AAACGTGAATTCCCTTTTGATGTT-3’  sg_csf1ra_1: 5’-TAGGCAGAGACACTTTTTCCATGG-3’  sg_csf1ra_2: 5’-AAACCCATGGAAAAAGTGTCTCTG-3’  sg_slc45a2_1: 5’- TAGGTGACTACTGCCGCTCACAGT-3’  sg_slc45a2_2: 5’- AAACACTGTGAGCGGCAGTAGTCA-3’  bio_*cdkn1a*_fw: 5’-TCTTACACCAAACACCACAA-3’ bio_*cdkn1a*_rv: 5’-TAAAACATGCAGGATACCGG-3’  sg_*cdkn1a*_1: 5’-TAGGAATATCACTCCCCGGATTTC-3’ sg_*cdkn1a*_2: 5’-AAACGAAATCCGGGGAGTGATATT-3’  mitfa_fw: 5’-TGCTTCACATACGTTTGCAG-3’  mitfa_rv: 5’-CAAAGGTCTGAGGGCTTTCC-3’  ltk_fw: 5’-TGTTCTGTCACCACCCTTGT-3’  ltk_rv: 5’-ACACTGCTATTACCAGGTTTGAC-3’  csf1ra_fw: 5’-CATAGATACCGTGCAAGCCTG-3’  csf1ra_rv: 5’-AGCCCAGGTATGAAATCCGT-3’,  slc45a2_fw: 5’-GGATTTGGTGTTTTGGCCCT-3’ slc45a2_rv: 5’-GTAACTCGGCTCTAATCGTGC-3’  HRMA_mitfa_fw: 5’-CCTCACGAGTCTCTCTATCA-3’  HRMA_mitfa_rv: 5’-GCCCCATGAACCCAATATAA-3’  HRMA_ltk_fw: 5’-CCACAGACTCTTCCAGAAAT-3’  HRMA_ltk_rv: 5’-CTGATTATGAGGTGCGACTA-3’  HRMA_csf1ra_fw: 5’-AGTGTGTGGCTTTCAATTTG-3’  HRMA_csf1ra_rv: 5’-TTTCTGGTGAGTGTTTGTTA-3’.  q_mitfa_fw: 5’-TGAAGCAAGTACTGGACAAG-3’  q_mitfa_rv: 5’-TCCAGTAGAGTCAGAAGTCC-3’  q_ltk_fw: 5’-CTGGGAGGAATCCGCTTA-3’  q_ltk_rv: 5’-AGTGAGACCAGTGCAGAG-3’  q_csf1ra_fw: 5’-AGTTCAAATGTATCAGAGACCT-3’  q_csf1ra_rv: 5’-TATCCTGCTCCGAGAATCAT-3’,  q_gfp_fw: 5’-AAGGGCATCGACTTCAAGGA-3’  q_gfp_rv: 5’-GGCGGATCTTGAAGTTCACC-3’  q_ntr_fw: 5’-CTTTTGATGCCAGCAAGAAA-3’  q_ntr_rv: 5’-GAAGCCACAATAAAATGCCA-3’  q_cdkn1a_fw: 5’-ATGTGCAGAGGGATGGCTAC-3’  q_cdkn1a_rv: 5’-CCTCCAGATCTTTACGCAG-3’  q_rpl13a_fw: 5’-ACTGTCAGAGGCATGCTTCC-3’  q_rpl13a_rv: 5’-TGCTCTGAAAATTGTGCGCC-3’ | See: Design and synthesis of single-guide RNAs (sgRNAs) (Online Methods)  See: Design and synthesis of DNA donor templates for HDR  (Online Methods)  See: Restriction enzyme digest (Online Methods)  See: High-resolution melting analysis (HRMA)  (Online Methods)  cDNA synthesis and gene expression analysis  (Online Methods) |  |
|  |  |  |
| **Cell materials** | **Indicate where provided: section/figure legend** | **N/A** |
| Cell lines: Provide species information, strain. Provide accession number in repository OR supplier name, catalog number, clone number, OR RRID. | - - - |  |
| Primary cultures: Provide species, strain, sex of origin, genetic modification status. | Primary cells from *klara;cdkn1a^kiki^* fish (Fig. 5 – fig. suppl. 2)  Species: *N. furzeri*  Strain: *klara-cdkn1a-ki-eGFP-NTR*  Sex: male  Genetic mod.: Knock-in of the *P2A-eGFP-P2A-NTR* cassette into the *cdkn1a* locus of *klara* |  |
|  |  |  |
| **Experimental animals** | **Indicate where provided: section/figure legend** | **N/A** |
| Laboratory animals or Model organisms: Provide species, strain, sex, age, genetic modification status. Provide accession number in repository OR supplier name, catalog number, clone number, OR RRID. | Experiments were performed using fish of the species *Nothobranchius furzeri*.  Fig 1A-C  Species: *N. furzeri*  Strain: MZCS-08/122  Sex: male + female  Age: 1, 2, 3 and 6 weeks  Genetic mod.: wild type  Fig 1E-F  Species: *N. furzeri*  Strain: MZCS-08/122  Sex: male + female  Age: 25 days/21 days  Genetic mod.: wild type  Fig 1G-L’  Species: *N. furzeri*  Strain: *M08-mitfa^-^,ltk^-^,csf1ra^-^*  Sex: male + female  Age: 25-38 days  Genetic mod.: Knockout of *mitfa*, *ltk* and *csf1ra*  Fig 1M-M’  Species: *N. furzeri*  Strain: *klara*  Sex: male + female  Age: 39 days  Genetic mod.: Knockout of *mitfa*, *ltk* and *csf1ra*  ______________________________________  Fig 2B  Species: *N. furzeri*  Strain: *M08-mitfa^-^,ltk^-^,csf1ra^-^*  Sex: male + female  Age: 312 days  Genetic mod.:   - *mitfa^-/-^* - *ltk^-/-^* - *csf1ra^+/+^, csf1ra^+/-^ or csf1ra^-/-^*   Fig 2C-C’  Species: *N. furzeri*  Strain: *klara*  Sex: male + female  Age: 42 or 239 days  Genetic mod.: Knockout of *mitfa*, *ltk* and *csf1ra*  ______________________________________  Fig 3A  Species: *N. furzeri*  Strain: *klara* and MZCS-08/122  Sex: male + female  Age: 14 weeks  Genetic mod.: Knockout of *mitfa*, *ltk*,*csf1ra* and wild type  Fig 3B-C  Species: *N. furzeri*  Strain: *klara* and MZCS-08/122  Sex: male + female  Age: 18 weeks  Genetic mod.: Knockout of *mitfa*, *ltk*,*csf1ra* and wild type  _____________________________________  Fig 4A  Species: *N. furzeri*  Strain: *klara* and MZCS-08/122  Sex: male + female  Age: 20/21 days  Genetic mod.: Knockout of *mitfa*, *ltk*,*csf1ra* and wild type  Fig 4c  Species: *N. furzeri*  Strain: *klara;slc45a2^-^*  Sex: female  Age: 32 days  Genetic mod.: Knockout of *mitfa*, *ltk*,*csf1ra* and *slc45a2*  *________________________________________*  Fig 5H  Species: *N. furzeri*  Strain: *klara-cdkn1a-ki-eGFP-NTR*  Sex: male  Age: 4 and 30 weeks  Genetic mod.: Knock-in of the *P2A-eGFP-P2A-NTR* cassette into the *cdkn1a* locus of *klara*  Fig 5I  Species: *N. furzeri*  Strain: *klara-cdkn1a-ki-eGFP-NTR*  Sex: male and female  Age: 4.5, 9.5 and 20.5 weeks  Genetic mod.: Knock-in of the *P2A-eGFP-P2A-NTR* cassette into the *cdkn1a* locus of *klara*  Fig 5J-M  Species: *N. furzeri*  Strain: *klara* and *klara-cdkn1a-ki-eGFP-NTR*  Sex: unknown (fish too young)  Age: 4 and 17 days  Genetic mod.: Knockout of *mitfa*, *ltk*,*csf1ra* and knock-in of the *P2A-eGFP-P2A-NTR* cassette into the *cdkn1a* locus of *klara* |  |
| Animal observed in or captured from the field: Provide species, sex, and age where possible. | - - - |  |
|  |  |  |
| **Plants and microbes** | **Indicate where provided: section/figure legend** | **N/A** |
| Plants: provide species and strain, ecotype and cultivar where relevant, unique accession number if available, and source (including location for collected wild specimens). | - - - |  |
| Microbes: provide species and strain, unique accession number if available, and source. | - - - |  |
|  |  |  |
| **Human research participants** | **Indicate where provided: section/figure legend) or state if these demographics were not collected** | **N/A** |
| If collected and within the bounds of privacy constraints report on age, sex, gender and ethnicity for all study participants. | - - - |  |

**Design:**

| **Study protocol** | **Indicate where provided: section/figure legend** | **N/A** |
| --- | --- | --- |
| If the study protocol has been pre-registered, provide DOI. For clinical trials, provide the trial registration number OR cite DOI. | - - - |  |
|  |  |  |
| **Laboratory protocol** | **Indicate where provided: section/figure legend** | **N/A** |
| Provide DOI OR other citation details if detailed step-by-step protocols are available. | **Molecular Sexing of *N. furzeri*** (Fig. 2 – fig. supp. 1B):  Richter A, Krug J, Englert C. Molecular Sexing of *Nothobranchius furzeri* Embryos and Larvae. Cold Spring Harb Protoc. 2022 Dec 1;2022(12):630-640. doi: 10.1101/pdb.prot107782. PMID: 36167675.  **Genotyping via HRMA** (Fig. 1M,M’; 3B)  Krug J, Richter A, Englert C. Rapid Genotyping of *Nothobranchius furzeri* Fish and Larvae via High-Resolution Melt Analysis (HRMA). Cold Spring Harb Protoc. (in press). doi:10.1101/pdb.prot107744. |  |
|  |  |  |
| **Experimental study design (statistics details) *** | | |
| **For in vivo studies: State whether and how the following have been done** | **Indicate where provided: section/figure legend. If it could have been done, but was not, write “not done”** | **N/A** |
| Sample size determination | not done |  |
| Randomisation | not done |  |
| Blinding | not done |  |
| Inclusion/exclusion criteria | not done |  |
|  |  |  |
| **Sample definition and in-laboratory replication** | **Indicate where provided: section/figure legend** | **N/A** |
| State number of times the experiment was replicated in the laboratory. | Due to restrictions in the amount of animals, experiments were not repeated. However, sample sizes (biological replicates) were kept large to provide more accurate mean values. Wherever possible, technical replicates were included (e.g. qPCR). |  |
| Define whether data describe technical or biological replicates. | - For all qPCR analyses (e.g. Fig. 1A-C), each fish used represents one individual biological replicate. Each sample was measured in triplicates (= technical replicate). - For FACS analyses (Fig. 2B + 5I) each fish represents one individual biological replicate. - For measurements of body size and weight (Fig. 3C + Fig. 2 – fig supp. 1B) each fish represents one individual biological replicate. - For the analysis of lifespan (*klara* vs wild type) each fish represents one individual biological replicate. |  |
|  |  |  |
| **Ethics** | **Indicate where provided: section/submission form** | **N/A** |
| Studies involving human participants: State details of authority granting ethics approval (IRB or equivalent committee(s), provide reference number for approval. | - - - |  |
| Studies involving experimental animals: State details of authority granting ethics approval (IRB or equivalent committee(s), provide reference number for approval. | All animal licences required to perform the work with *Nothobranchius furzeri* in  this manuscript were approved by the “Thüringer Landesamt für Verbraucherschutz“ in Bad  Langensalza, Germany.  Animal licences:   - §11 J-003798 - FLI-17-016 - FLI-20-001 - FLI-20-102 |  |
| Studies involving specimen and field samples: State if relevant permits obtained, provide details of authority approving study; if none were required, explain why. | - - - |  |
|  |  |  |
| **Dual Use Research of Concern (DURC)** | **Indicate where provided: section/submission form** | **N/A** |
| If study is subject to dual use research of concern regulations, state the authority granting approval and reference number for the regulatory approval. | - - - |  |

**Analysis:**

| **Attrition** | **Indicate where provided: section/figure legend** | **N/A** |
| --- | --- | --- |
| Describe whether exclusion criteria were pre-established. Report if sample or data points were omitted from analysis. If yes, report if this was due to attrition or intentional exclusion and provide justification. | No sample or data points were omitted from analyses in this manuscript. |  |
|  |  |  |
| **Statistics** | **Indicate where provided: section/figure legend** | **N/A** |
| Describe statistical tests used and justify choice of tests. | Fig 1A-C   - Used tests: Student’s or Welch’s t-tests - Comparison of two groups.   Depending on equal or unequal variance (determined via F-test) use of Student’s or Welch’s t-test, respectively.  Fig 2B   - Used test: One-way ANOVA followed by Tukey’s post hoc test - Determining of sig. differences between the means of three groups (*csf1ra^+/+^, csf1ra^+/-^* and *csf1ra^-/-^*).   Fig 3A   - Used tests: Student’s or Welch’s t-tests - Comparison of two groups.   Depending on equal or unequal variance (determined via F-test) use of Student’s or Welch’s t-test, respectively.  Fig 3C   - Used tests: Student’s or Welch’s t-tests - Comparison of two groups.   Depending on equal or unequal variance (determined via F-test) use of Student’s or Welch’s t-test, respectively.  Fig 5E-G   - Used test: Student’s t-test - Comparison of two groups.   Due to equal variance (determined via F-test) use of Student’s t-test.  Fig 5I   - Used test: One-way ANOVA followed by Tukey’s post hoc test - Determining of sig. differences between the means of three groups (4.5, 9.5 and 20.5 wph).   _______________________________________  Fig 1 – fig. suppl. 1A-C   - Used tests: Student’s or Welch’s t-tests - Comparison of two groups.   Depending on equal or unequal variance (determined via F-test) use of Student’s or Welch’s t-test, respectively.  Fig 2 – fig. suppl. 1A   - Used test: Log-rank test   Fig 2 – fig. suppl. 1B   - Used test: Student’s or Welch’s t-test - Comparison of two groups.   Depending on equal or unequal variance (determined via F-test) use of Student’s or Welch’s t-test, respectively.  Fig 5 – fig. suppl. 2C   - Used test: One-way ANOVA followed by Tukey’s post hoc test - Determining of sig. differences between the means of four groups. |  |
|  |  |  |
| **Data availability** | **Indicate where provided: section/submission form** | **N/A** |
| For newly created and reused datasets, the manuscript includes a data availability statement that provides details for access (or notes restrictions on access). | - - - |  |
| When newly created datasets are publicly available, provide accession number in repository OR DOI and licensing details where available. | - - - |  |
| If reused data is publicly available provide accession number in repository OR DOI, OR URL, OR citation. | - - - |  |
|  |  |  |
| **Code availability** | **Indicate where provided: section/figure legend** | **N/A** |
| For any computer code/software/mathematical algorithms essential for replicating the main findings of the study, whether newly generated or re-used, the manuscript includes a data availability statement that provides details for access or notes restrictions. | - - - |  |
| Where newly generated code is publicly available, provide accession number in repository, OR DOI OR URL and licensing details where available. State any restrictions on code availability or accessibility. | - - - |  |
| If reused code is publicly available provide accession number in repository OR DOI OR URL, OR citation. | - - - |  |

**Reporting:**

The MDAR framework recommends adoption of discipline-specific guidelines, established and endorsed through community initiatives.

| **Adherence to community standards** | **Indicate where provided: section/figure legend** | **N/A** |
| --- | --- | --- |
| State if relevant guidelines (e.g., ICMJE, MIBBI, ARRIVE, STRANGE) have been followed, and whether a checklist (e.g., CONSORT, PRISMA, ARRIVE) is provided with the manuscript. | - - - |  |

* We provide the following guidance regarding transparent reporting and statistics; we also refer authors to [Ten common statistical mistakes to watch out for when writing or reviewing a manuscript](https://doi.org/10.7554/eLife.48175).

**Sample-size estimation**

- You should state whether an appropriate sample size was computed when the study was being designed
- You should state the statistical method of sample size computation and any required assumptions
- If no explicit power analysis was used, you should describe how you decided what sample (replicate) size (number) to use

**Replicates**

- You should report how often each experiment was performed
- You should include a definition of biological versus technical replication
- The data obtained should be provided and sufficient information should be provided to indicate the number of independent biological and/or technical replicates
- If you encountered any outliers, you should describe how these were handled
- Criteria for exclusion/inclusion of data should be clearly stated
- High-throughput sequence data should be uploaded before submission, with a private link for reviewers provided (these are available from both GEO and ArrayExpress)

**Statistical reporting**

- Statistical analysis methods should be described and justified
- Raw data should be presented in figures whenever informative to do so (typically when N per group is less than 10)
- For each experiment, you should identify the statistical tests used, exact values of N, definitions of center, methods of multiple test correction, and dispersion and precision measures (e.g., mean, median, SD, SEM, confidence intervals; and, for the major substantive results, a measure of effect size (e.g., Pearson's r, Cohen's d)
- Report exact p-values wherever possible alongside the summary statistics and 95% confidence intervals. These should be reported for all key questions and not only when the p-value is less than 0.05.

**Group allocation**

- Indicate how samples were allocated into experimental groups (in the case of clinical studies, please specify allocation to treatment method); if randomization was used, please also state if restricted randomization was applied
- Indicate if masking was used during group allocation, data collection and/or data analysis
